# Distinguishing and phenotype monitoring of traumatic brain injury and post-concussion syndrome including chronic migraine in serum of Iraq and Afghanistan war veterans

**DOI:** 10.1371/journal.pone.0215762

**Published:** 2019-04-26

**Authors:** Jay S. Hanas, James R. S. Hocker, Megan R. Lerner, James R. Couch

**Affiliations:** 1 Department of Biochemistry, University of Oklahoma College of Medicine, Oklahoma City, Oklahoma, United States of America; 2 Department of Surgery, University of Oklahoma College of Medicine, Oklahoma City, Oklahoma, United States of America; 3 Veterans Administration Hospital, Oklahoma City, Oklahoma, United States of America; 4 Department of Neurology, University of Oklahoma College of Medicine, Oklahoma City, Oklahoma, United States of America; 5 Department of Neurology, Veterans Administration Hospital, Oklahoma City, Oklahoma, United States of America; University of Arizona, UNITED STATES

## Abstract

Traumatic Brain Injury (TBI) and persistent post-concussion syndrome (PCS) including chronic migraine (CM) are major health issues for civilians and the military. It is important to understand underlying biochemical mechanisms of these conditions, and be able to monitor them in an accurate and minimally invasive manner. This study describes the initial use of a novel serum analytical platform to help distinguish TBI patients, including those with post-traumatic headache (PTH), and to help identify phenotypes at play in these disorders. The hypothesis is that physiological responses to disease states like TBI and PTH and related bodily stresses are reflected in biomolecules in the blood in disease-specific manner. Leave one out (serum sample) cross validations (LOOCV) and sample randomizations were utilized to distinguished serum samples from the following TBI patient groups: TBI +PTSD + CM + severe depression (TBI “most affected” group) vs healthy controls, TBI “most affected” vs TBI, TBI vs controls, TBI + CM vs controls, and TBI + CM vs TBI. Inter-group discriminatory *p* values were ≤ 10^−10^, and sample group randomizations resulted in *p* non-significant values. Peptide/protein identifications of discriminatory mass peaks from the TBI “most affected” vs controls and from the TBI plus vs TBI minus CM groups yielded information of the cellular/molecular effects of these disorders (immune responses, amyloidosis/Alzheimer’s disease/dementia, neuronal development). More specific biochemical disease effects appear to involve blood brain barrier, depression, migraine headache, autoimmunity, and autophagy pathways. This study demonstrated the ability for the first time of a novel, accurate, biomarker platform to monitor these conditions in serum, and help identify biochemical relationships leading to better understanding of these disorders and to potential therapeutic approaches.

## Introduction

Traumatic brain injury (TBI) is a major health issue for civilians and for the military. Approximately 1.7 million TBIs are reported annually in the United States, with 275,000 hospitalizations, and 53,000 deaths [1]. A significant number of TBIs likely go unreported. TBI is problematic for the military with 15–20% of Soldiers deployed to Afghanistan and/or Iraq wars experiencing a deployment-related TBI (D-TBI) [2, 3]. A majority of TBIs, including in the military, are mild with loss of consciousness (LOC) <30 minutes [1–4]. TBI is often associated with the post-concussion syndrome (PCS) which can include chronic daily headache (CDH), post-traumatic stress disorder (PTSD), and/or depression, all of which are health concerns for both military personnel and civilians [5–7]. These PCS conditions can occur for extended periods of time after the D-TBI [1–4, 7–9]. Headache (HA) is the most frequent PCS symptom (referred to as post-traumatic headache-PTH), and is a problem that can persist/reoccur for months to years after the TBI [8–12]. Studies of veterans at 2–11 years after D-TBI, 98% reported continuing HA of which 45% had CDH (occurring ≥ 15 days/month); two thirds of these had chronic migraine (CM) [headache occurring ≥ 15 days/month with ≥ 8 of these being migraine]) [4, 13]. The prevalence of CM in deployed veteran controls (8%) was about four times the reported prevalence of CM in the civilian population of 1–2% [14, 15]. In these analyses 2–11 years after D-TBI, PTSD was found in about 60% of TBI patients and about 10% of controls [4]. Severe depression (SDep) was noted in 43% of the D-TBI patients and only in 6% of controls. D-TBI subjects with a combination of PTSD and/or severe depression was found in approximately 60% of those with CM. In patients with D-TBI and CM, the addition of PTSD and/or SDep make the headache severity worse and the diagnosis and treatment more complicated. These observations indicate in studies of TBI and PTH (including CM), especially associated with deployed veterans, consideration should be given to these related factors (PTSD and/or SDep) in an effort to better understand TBI PCS patient symptoms and their persistence and potential biochemical/physiological inter-relationships.

It is important to develop accurate molecular aids useful for monitoring these conditions, and for revealing mechanistic clues that could possibly aid in the development of therapies for individuals with these conditions, especially in their persistent state. Peripheral blood, plasma, and/or serum are ideal bodily fluids to learn detailed information about TBI and PCS disorders including PTH since minimally invasive procedures are used to procure this biomaterial in a quick, efficient and accurate manner. Molecular information gleaned from such material is hypothesized to be able to help classify these conditions, understand any underlying associations/biochemical mechanisms, and provide possible targets for future biomarkers and therapeutic interventions. A number of studies have provided evidence indicating the analysis of peripheral blood (e.g., serum, plasma, blood cells) holds promise as minimally invasive aid or tool to study and understand TBI and associated PCS sequelae. In a study of acute TBI followed up to 90 days post-TBI using plasma immuno-analysis, increased levels of glial fibrillary protein, Tau, and Amyloid beta were observed in mild TBI patients versus healthy controls, providing evidence for physiological connections between TBI and Alzheimer’s disease (AD) [16]. A plasma micro RNA (miRNA) study of acute TBI identified several specific miRNA molecules whose levels decreased over 30 days after the TBI [17]. PTSD was found to be associated with increased blood levels of cytokines and suggests neuro-inflammation may be a key component in developing/maintaining this disorder [18]. Higher levels of serum inflammatory marker, C-reactive protein, were associated with severe depression (SDep) suggesting an inflammatory component of SDep [19]. Several blood biomarkers were reported to be associated with migraine headaches including CM. The calcitonin gene-related peptide (CGRP) is one such serum marker reported for acute migraine and CM [20]. An inflammatory connection to migraine has been alluded to with the observations that serum acute phase proteins pentraxin-3 and fibrinogen are found associated with migraine duration [21].

Currently there are no biomarkers in clinical use approved to assist in distinguishing and monitoring patients with TBI and related PCS symptoms including CM, PTSD, and/or SDep. Evidence indicates these potential post-concussion effects of TBI can be observed >30 years after initial brain concussion [4, 22]. Most molecular studies on TBI and PCS biomarkers are performed in the early acute phases of the disorders and have ignored long-term variations. The present study is unique in that TBI and PCS including CM are being studied in a 5–14 year window after the initial concussion event. In this regard, it is important to see if such conditions can be monitored in an extended time frame in an accurate, robust, and straight-forward manner. In addition, molecular analysis of such a prolonged window may yield possible mechanisms to help explain the persistence of these disease states over time. One unique aspect, of this study, is the patient recruitment strategy which seeks out a narrow source of patients (veterans deployed to Mideast war theaters) with a large swath (plus or minus a D-TBI) of symptoms but with no recruiting emphasis on PTH and PCS. This created two pools of subjects from which subgroups based on occurrence of D-TBI, severity of TBI, type of headache, and presence of PTSD and/or depression could be created. A third unique feature of this study Is the use, for the first time in the TBI and headache field, of a minimal serum electrospray ionization mass spectrometry (ESI-MS) platform as a potential tool to help monitor and understand TBI and PTH. This platform has had success in the cancer field being able to distinguish both early-stage lung cancer and pancreatic cancer as well as provide mechanistic information about the diseases [23, 24]. The large number of different identifiers (serum mass peaks) used by this methodology, which differs from a number of other biomarker platforms which use far fewer identifiers, appear to provide the highly specific disease discrimination and elucidation ability of this platform. It is anticipated that this unique property of this platform will be successful in this present study [23, 25, 26].

## Materials and methods

### Patients and clinical samples

**Fig 1:**
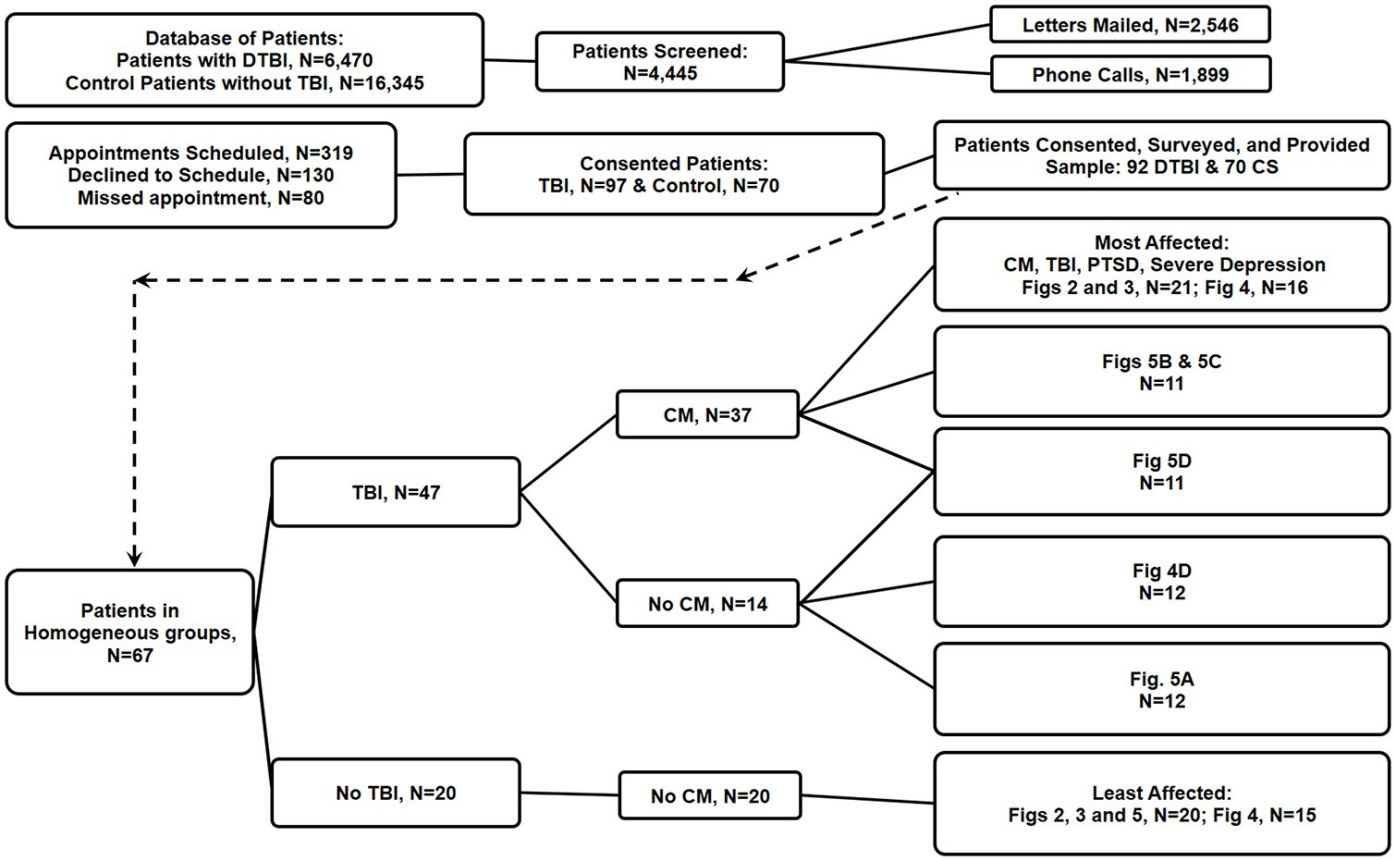
Study volunteer numbers, health characteristics, groupings, and figure inclusions. The number of patients available for recruitment, number of patients recruited, number of patients utilized, and the identification of the figures where these patients were utilized, are indicated.

**Fig 2:**
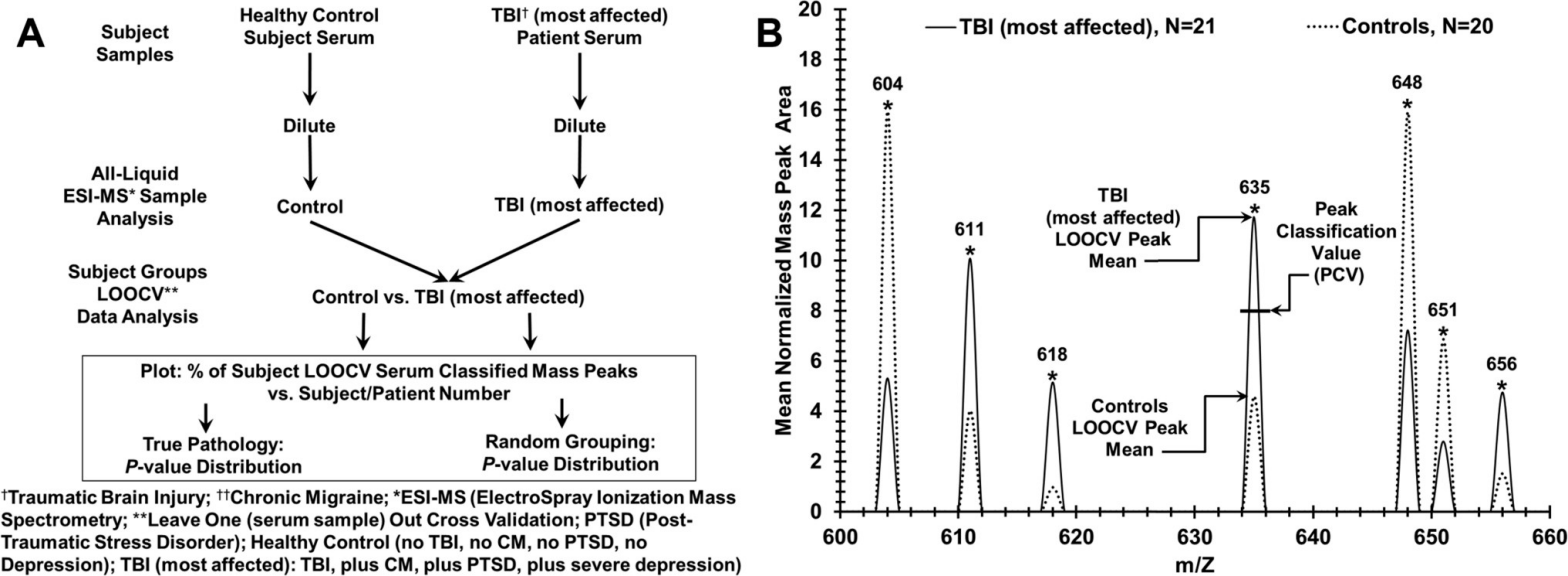
Experimental approach for discriminating sera from control individuals and patients with TBI and post- concussion syndrome (PCS) sequelae. (A) Flow chart for serum sample handling and mass spectrometry for binary patient/subject group analysis. Distinguishing control samples from TBI “most affected” samples is exhibited. (B) Peak Scoring for LOOCV (leave [one serum sample] out cross validation) procedure to classify mass peaks either “most affect” or control from a “left out” sample, over a narrow range (600–660 m/Z is displayed) of significant group discriminatory mass peaks. The PCV (peak classification value) example is exhibited on peak 635 which is used to classify “left out” peaks as either most affected (peak area above this PCV) or control (peak area at or below this PCV).

**Fig 3:**
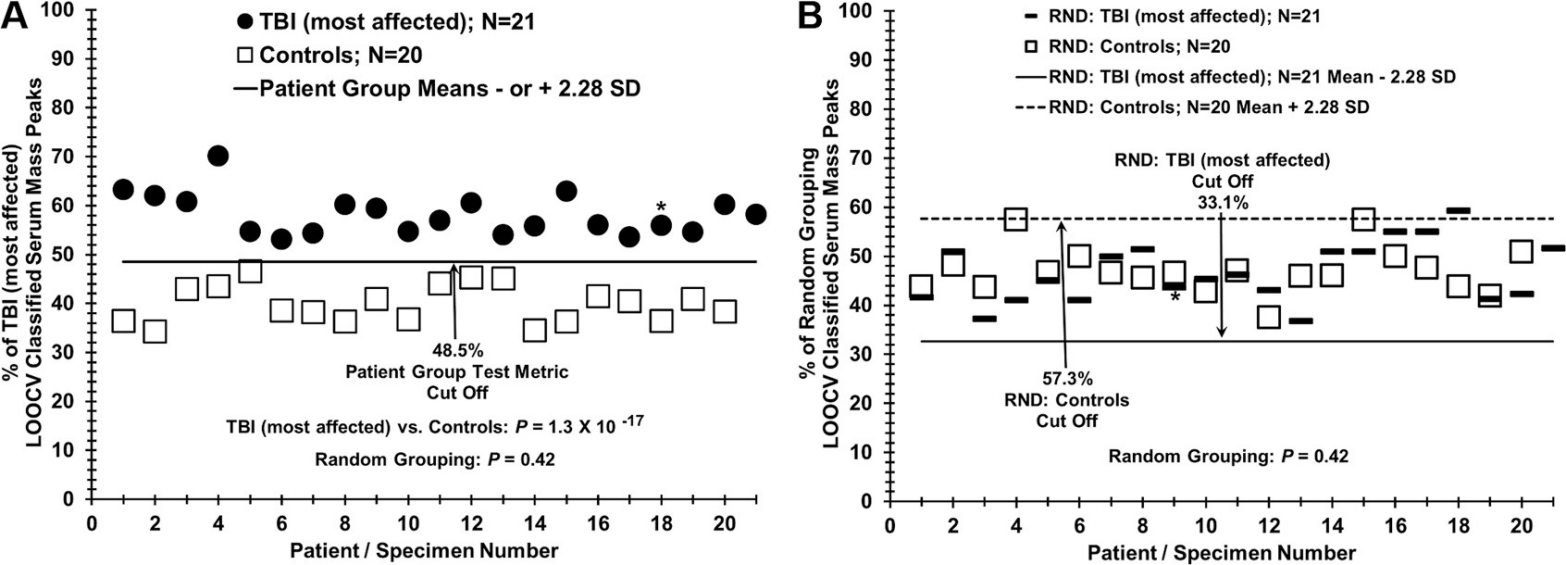
Distinguishing sera from TBI “most affected” patients versus controls using LOOCV and sample randomization analyses. Male veterans of the United States Iraq and Afghanistan Wars were age-matched selected for these two different groups with similar war theater experiences but either having mild TBI and post-concussion sequelae PTSD and CM and SDep (most affected group) or lack of all these maladies (control group). (A) Serum discrimination of TBI most affected patients (dark circles) from controls (squares) by % of LOOCV classified mass peaks. A cut off value is present (- or + SDs from the most affected or control groups respectively) to determine test metric values (e.g. true positives). (B) Non-serum sample discrimination when the two different sample groups are mixed together randomly followed by the same LOOCV mass peak analysis. An * indicates a female volunteer and no experimental segregation is observed from the male volunteers in this analysis.

**Fig 4:**
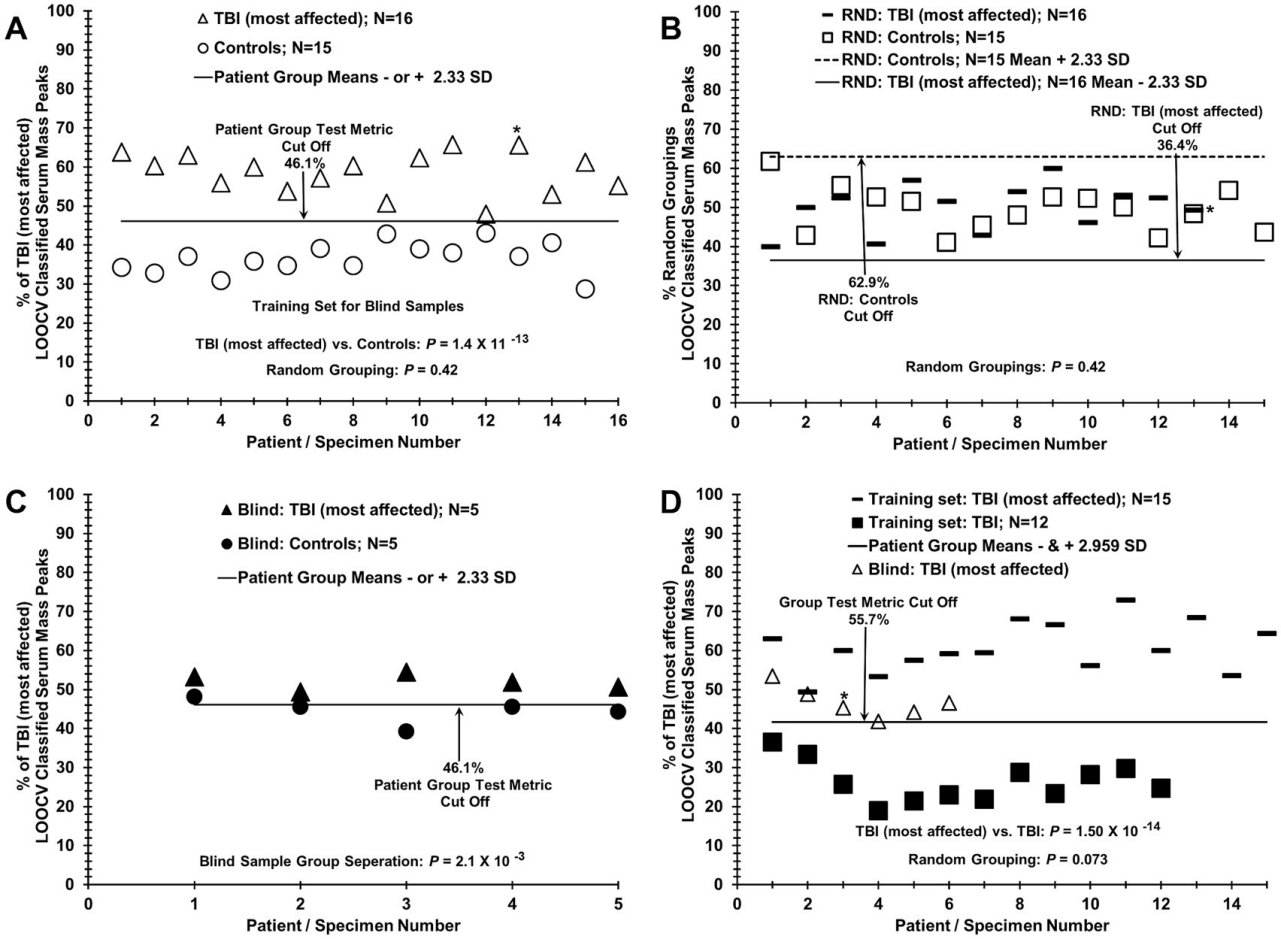
Analysis of blinded sera samples: Comparing patients with TBI+PCS versus controls or TBI alone. (A)Training set of serum discrimination of TBI most affected patients (triangles) from controls (circles) by % of LOOCV classified mass peaks; correct true positives and true negatives are observed. (B) Non-serum sample discrimination observed when the two different sample groups in panel A are mixed together randomly followed by the same LOOCV mass peak analysis. (C) Assessing the ability of the training set in panel A to correctly discriminate a blinded group of ten samples; 9 out of 10 samples were correctly identified. (D) Discrimination of sera from TBI most affected patients (dashes) from patients with TBI alone (dark squares), and assessment of 6 “left out” group of TBI most affected patients (triangles); 5 out of 6 blinded most affect patients were identified. An * indicates a female volunteer and no experimental segregation is observed from the male volunteers in this analysis.

**Fig 5:**
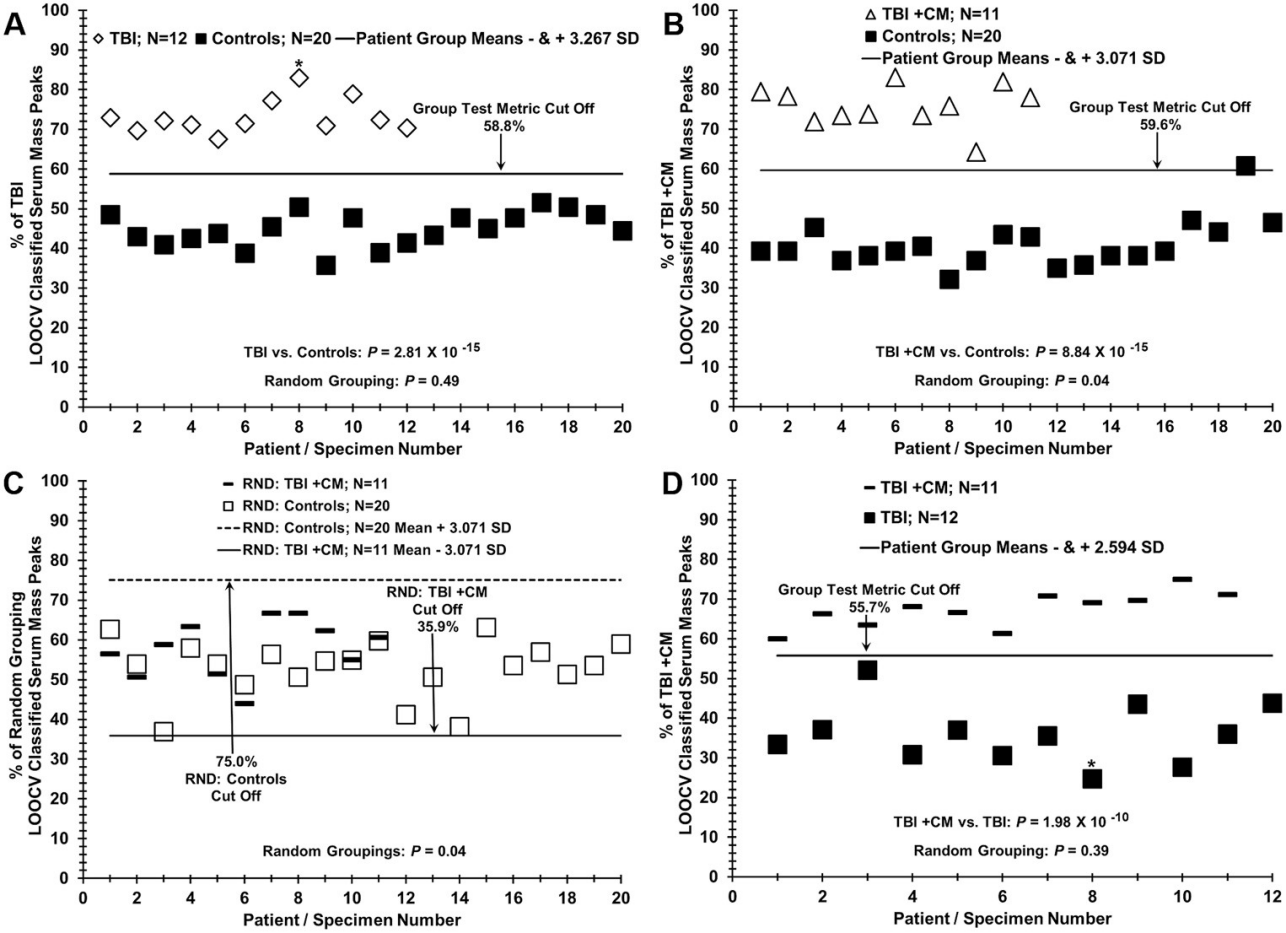
Distinguishing sera of TBI patients with and without chronic migraine (CM). (A) Sera discrimination of patients with TBI alone (diamonds) versus sera from control individuals (dark squares) by % of LOOCV classified mass peak analysis. (B) Sera discrimination of patients with TBI plus CM (triangles) versus sera from control individuals (dark squares). (C) Non-serum sample discrimination observed when the two different sample groups in B are mixed together randomly followed by the same LOOCV mass peak analysis. (D) Sera discrimination of patients with TBI plus CM (dashes) versus TBI alone (dark squares) by % of LOOCV classified mass peak analysis. An * indicates a female volunteer and no experimental segregation is observed from the male volunteers in this analysis.

All study subjects were recruited from a listing of Operation Enduring Freedom (OEF) and Operation Iraqi Freedom (OIF) veterans in the Oklahoma City Veterans Administration Medical Center (VAMC) catchment area. This list was constructed from the VA Veterans Integrated Service Network (VISN-16 & VISN-19) Data Repository and contained 6,470 OEF/OIF veterans who had suffered a confirmed D-TBI and 16,345 possible control subjects who had not suffered a D-TBI (Fig 1). Of these totals, 4445 Subjects who lived within 100 miles of the Oklahoma City VA Medical Center or, if located farther away but received medical care at the OKC VAMC, were identified and contacted randomly to request their participation in the study. No other criteria were applied in selection for initial contact. The major inclusion criteria for this study were: (a) deployment to a war zone, (b) absence of major organ disease or infectious/inflammatory disease at the time of the D-TBI and at the time of interview, and (c) occurrence of a D-TBI for the TBI subjects, and absence of a D-TBI for controls. 167 subjects were recruited into the study from the above VISN list. Participants provided written consent (at the time of the blood draw) via a signed consent document approved by the University of Oklahoma Health Sciences Center Institutional Review Board of Human Subjects Research (IRB # 6839). During the scheduled appointment the individuals were interviewed, completed the required questionnaires (described in the next section and in S1 Appendix), and provided a peripheral blood sample from an arm vein. These study volunteers resulted from 319 scheduled appointments of individuals who fulfilled all the requirements of the study and who responded to our initial contacts (mailings or phone calls). Not all individuals scheduling appoints appeared for the consenting, interview, questionnaire, and blood draw process. 162 volunteers completed this entire process. Six of the 162 patients were females, and two were included in the present analysis of 65 volunteers, analysis method outlined in Fig 2, and with one female in Figs 3 and 4, and a different female in Fig 5. The remaining study subjects were males. The study breaks down with 47 subjects in the D-TBI group and 20 subjects in the control, non D-TBI group. The final N values for the experiments and respective figures depicted in this study are also given in Fig 1. D-TBI subjects were recruited from veterans seen and diagnosed in the Oklahoma City VAMC (Veterans Administration Medical Center) TBI clinic. All TBI subjects had suffered their D-TBIs between 5 and 14 years prior to entering this study. The Volunteers consented to participate in the study and their blood samples were collected between January 1, 2017 to December 31, 2017.

Specific and pertinent patient/subject characteristics (e.g., PTSD and depression presence) for the 65 veteran serum samples used in the group comparison figures are given in Table 1.

**Table 1:** Patient characteristics.

Group characteristics and Figure identifier	Age in years	TBI (N)	PTSD (N):	Depression (N)	Headache (N)	Years since TBI
	Mean ±SD (range)	n, v-M, M, Mod	n, Poss, Prob	n/m, M, Mod, SDep	n, FH, CM	Mean ±SD (range)
All Patients						
All Patients, N = 65	43.29 ±8.38 (29–64)	20, 19, 21, 5	22, 9, 34	14, 27, 4, 20	33, 1, 31	10.89 ±2.48 (5–14)
All Controls, N = 20	44.05 ±8.8 (34–64)	20, 0, 0, 0	10, 0, 10	0, 20, 0, 0	20, 0, 0	na
All TBI, N = 45	42.96 ±8.26 (29–61)	0, 19, 21, 5	12, 9, 24	14, 7, 4, 20	13, 1, 31	10.89 ±2.48 (5–14)
Fig 3 panel A & Fig 3 panel B						
TBI (MA), N = 21	41.24 ±8.51 (29–61)	0, 7, 11, 3	6, 2, 13	0, 1, 0, 20	0, 1, 20	11.67 ±2.33 (6–14)
Controls, N = 20	44.05 ±8.8 (34–64)	20, 0, 0, 0	10, 0, 10	0, 20, 0, 0	20, 0, 0	na
RND: TBI (MA), N = 21	42.62 ±8.74 (30–64)	10, 5, 4, 2	8, 1, 12	0, 11, 0, 10	10, 1, 10	11.09 ±2.39 (6–14)
RND: Controls, N = 20	42.6 ±8.81 (29–61)	10, 2, 7, 1	8, 1, 11	0, 10, 0, 10	10, 0, 10	12.3 ±2.21 (7–14)
Fig 4 panel A & Fig 4 panel C						
TS TBI, (MA), N = 16	41.25 ±9.58 (29–61)	0, 7, 6, 3	4, 2, 10	0, 1, 0, 15	0, 1, 15	11.56 ±2.56 (6–14)
TS Controls, N = 15	43.87 ±10.23 (34–64)	15, 0, 0, 0	8, 0, 7	0, 15, 0, 0	15, 0, 0	na
TS RND: TBI (MA), N = 16	42.31 ±9.67 (29–64)	8, 1, 5, 2	6, 0, 10	0, 8, 0, 8	8, 0, 8	12.25 ±2.25 (7–14)
TS RND: Controls, N = 15	42.73 ±10.32 (30–61)	7, 6, 1, 1	6, 2, 7	0, 8, 0, 7	7, 1, 7	10.88 ±2.8 (6–14)
Blinds TBI (MA), N = 5	41.2 ±4.21 (37–47)	0, 0, 5, 0	2, 0, 3	0, 0, 0, 5	0, 0, 5	12 ±1.58 (10–14)
Blinds Control, N = 5	44.6 ±1.34 (43–46)	5, 0, 0, 0	2, 0, 3	0, 5, 0, 0	5, 0, 0	na
Fig 4 panel D						
TS TBI (MA), N = 15	43.73 ±8.15 (33–61)	0, 3, 10, 2	5, 1, 9	0, 0, 0, 15	0, 0, 15	12.33 ±1.54 (9–14)
TS TBI, N = 12	44.58 ±7.93 (30–57)	0, 7, 3, 2	3, 7, 2	7, 4, 1, 0	12, 0, 0	10.5 ±2.68 (5–14)
TS RND: TBI (MA), N = 15	44.07 ±7.1 (30–51)	0, 7, 5, 3	6, 3, 6	4, 2, 1, 0	7, 0, 8	10.87 ±2.47 (5–14)
TS RND: TBI, N = 12	44.17 ±9.15 (34–61)	0, 3, 8, 1	2, 5, 5	3, 2, 0, 7	5, 0, 7	12.33 ±1.78 (8–14)
Blinds TBI (MA), N = 6	35 ±6.16 (29–43)	0, 4, 1, 1	1, 1, 4	0, 1, 0, 5	0, 1, 5	10 ±3.22 (6–14)
Fig 5 panel A						
TBI, N = 12	45.25 ±6.85 (37–57)	0, 6, 4, 2	4, 7, 1	7, 4, 1, 0	12, 0, 0	11.17 ±2.12 (8–14)
Controls, N = 20	44.05 ±8.8 (34–64)	20, 0, 0, 0	10, 0, 10	0, 20, 0, 0	20, 0, 0	na
RND: TBI, N = 12	47 ±6.28 (37–57)	6, 3, 2, 1	8, 2, 2	3, 8, 1, 0	12, 0, 0	10.83 ±1.94 (8–13)
RND: Controls, N = 20	43 ±8.72 (34–64)	14, 3, 2, 1	6, 5, 9	4, 16, 0, 0	20, 0, 0	11.5 ±2.43 (8–14)
Fig 5 panel B & Fig 5 panel C						
TBI with CM, N = 11	44.91 ±8.37 (34–59)	0, 5, 6, 0	2, 0, 9	7, 2, 2, 0	0, 0, 11	9.64 ±2.16 (6–13)
Control, N = 20	44.05 ±8.8 (34–64)	20, 0, 0, 0	10, 0, 10	0, 20, 0, 0	20, 0, 0	na
RND: TBI with CM, N = 11	46.27 ±10.32 (34–64)	7, 1, 3, 0	3, 0, 8	3, 8, 0, 0	7, 0, 4	11.25 ±1.5 (10–13)
RND: Control, N = 20	43.3 ±7.44 (34–58)	13, 4, 3, 0	9, 0, 11	4, 14, 2, 0	13, 0, 7	8.71 ±1.98 (6–12)
Fig 5 panel D						
TBI with CM, N = 11	44.91 ±8.37 (34–59)	0, 5, 6, 0	2, 0, 9	7, 2, 2, 0	0, 0, 11	9.64 ±2.16 (6–13)
TBI, N = 12	45.25 ±6.85 (37–57)	0, 6, 4, 2	4, 7, 1	7, 4, 1, 3	12, 0, 0	11.17 ±2.12 (8–14)
RND: TBI with CM, N = 11	43.18 ±7.48 (34–56)	0, 5, 5, 1	0, 4, 7	6, 4, 1, 0	5, 0, 6	11.36 ±2.06 (8–14)
RND: TBI, N = 12	46.83 ±7.27 (36–59)	0, 6, 5, 1	6, 3, 3	8, 2, 2, 0	7, 0, 5	9.58 ±2.11 (6–13)

MA (most affected); TS (training set); SD (standard deviation); TBI (traumatic brain injury); PTSD (post-traumatic stress disorder); n (none); v-M (very mild); M (mild); Mod (moderate); Poss (possible); Prob (probable); n/m (none/minimal); SDep (severe depression); FH (frequent headache); CM (chronic migraine); na (not applicable)

Patients are partitioned with respect to sera presence in the respective figures (far left column). Individuals and their sera in figure column were deemed appropriate on the basis of having the most homogeneity for the following sample groupings: a) patients with TBI plus PTSD plus SDep plus CM (most affected), b) healthy control individuals (least affected: no TBI, no PTSD, no SDep, no CM), c) TBI alone (with minimal/no PTSD, SDep, CM contributions), d) TBI plus CM (with minimal/no PTSD, SDep, CM contributions). The 20 healthy controls used in this study out of the total of 70 were selected for best age-matching to the TBI “most affected” patients. All TBI subjects and control subjects were administered the same questionnaires (QS) which included: (1) TBI QS, (2) headache QS, (3) PTSD (civilian) QS, and (4) Beck Depression Inventory 2 (BDI 2). TBI was graded by duration of loss of consciousness (LOC) as mild—LOC of 1–30 minutes; moderate—LOC 30–360 minutes, and severe—LOC >6 hours. The majority of the D-TBI subjects in this study were graded “mild”. Headache type was classified by criteria of International Classification of Headache disorders as migraine, tension, cluster, probable migraine or no headache [13]. PTSD *civ* score ranges assessed were ≤ 35, none, 36–49, possible PTSD, ≥ 50, highly probable PTSD. BDI 2 score ranges were ≤ 11, none, 12–19, mild depression, 20–28, moderate depression, ≥ 29, severe depression (SDep). Additional comprehensive patient characteristics are provided in S1–S6 Tables. Peripheral blood was obtained from consented control subjects or D-TBI patients in identical fashion at the Veterans Administration (VA) Hospital in Oklahoma City before any treatments. Blood (5 cc) was collected in no additive non-separator red stopper vacutainer tubes. Samples were allowed to clot at room temperature for 30 to 60 minutes and then spun at 4 °C for 10 minutes at 2,500 rpm. Serum aliquots of 250 μl were stored at -80 °C and thawed only once [27]. Patient protocols were reviewed and approved (# 6839) by a Human Subjects Institutional Review Board (IRB), at the University of Oklahoma Health Sciences Center and the Oklahoma City VA Hospital.

#### Electrospray mass spectrometry of sera from TBI and PCS veteran patients and from control veterans

**Fig 6:**
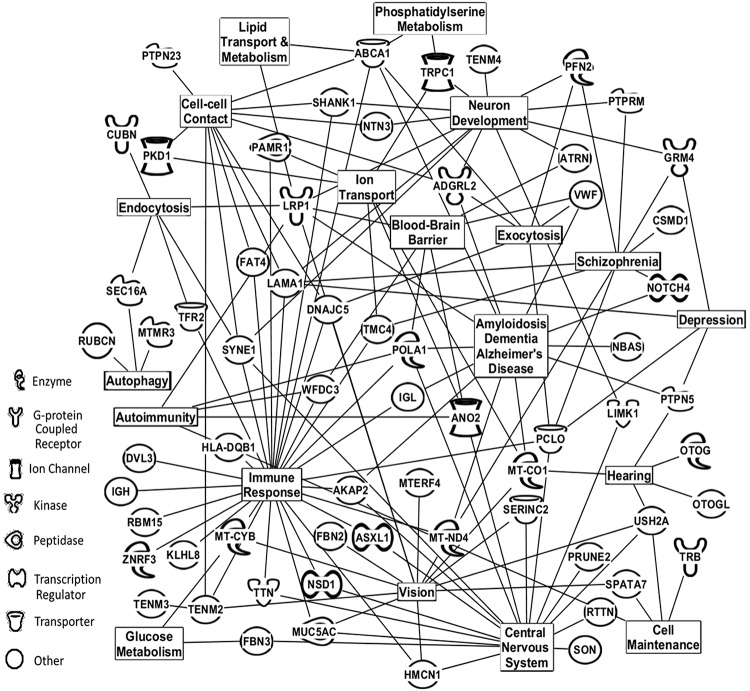
Physiological/cellular pathways of serum proteins found to distinguish TBI most affected patients from controls. Affected physiological/cellular pathways and serum protein assignments from Table 3 (top panel) that were found to distinguish TBI most affected patients from control individuals. The next top 58 proteins for each group (TBI most affected or control) not exhibited in Table 3 were added to the 48 in this table for this analysis. Analysis performed by using Ingenuity Pathway Analysis (IPA) bioinformatics software (Qiagen, Inc.).

**Fig 7:**
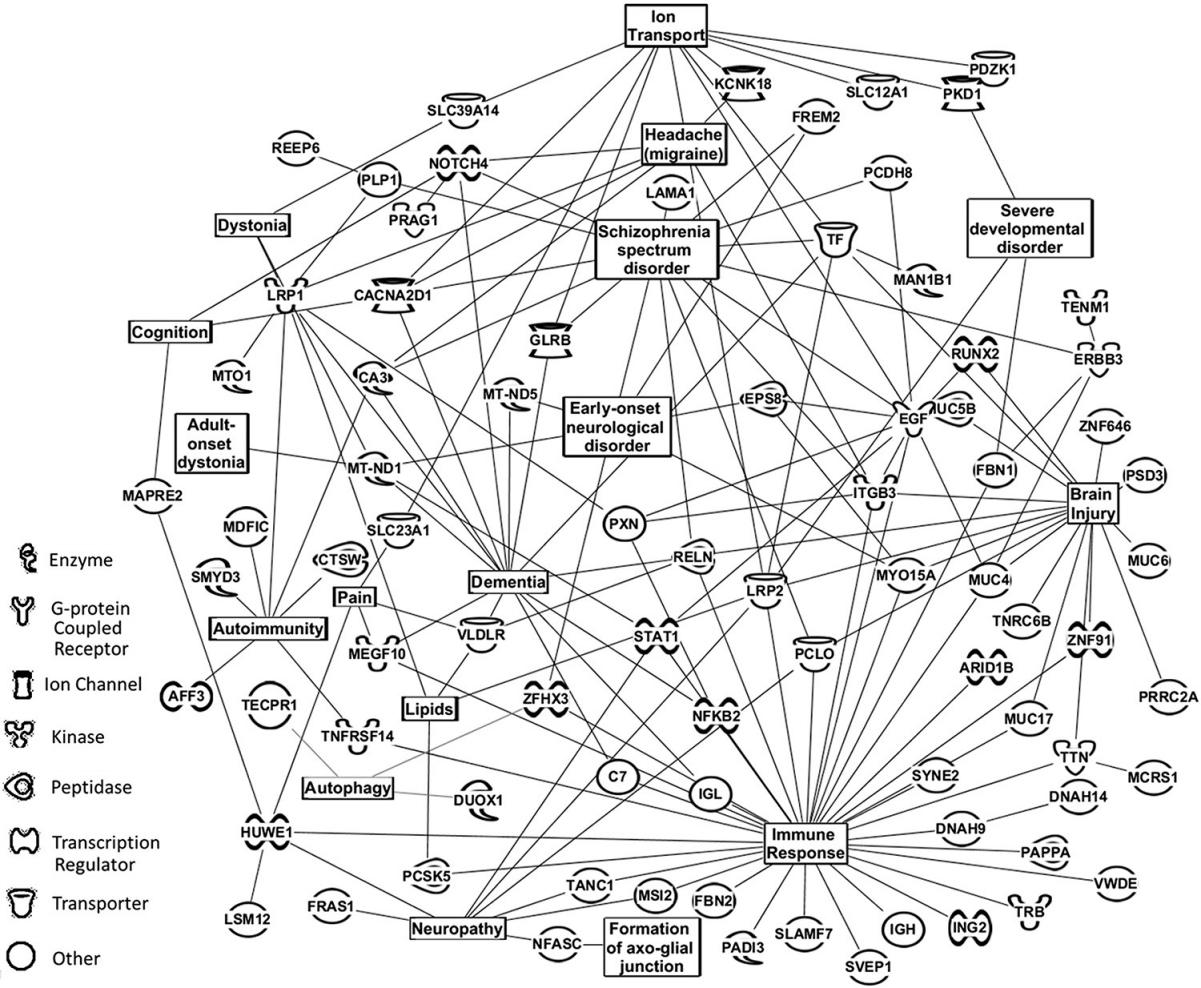
Physiological/cellular pathways of serum proteins found to distinguish TBI with CM from TBI only patients. Affected physiological/cellular pathways and serum protein assignments from Table 3 (bottom panel) that were found to distinguish patients with TBI alone versus patients with TBI plus CM. The next top 58 proteins for each group (TBI alone and TBI plus CM) not exhibited in Table 3 were added to the 48 in this table for this analysis. Analysis performed by using Ingenuity Pathway Analysis (IPA).

A serum sample (4 μl) was diluted 1 to 300 into a solution of 50% methanol and 2% formic acid and separated into 3 aliquots. Triplicate mass spectra (20 minute averages) were collected from each TBI patient or control individual sera in random fashion. Spectra were sampled at a m/Z (mass divided by charge) resolution of two hundredths over a m/Z range of 400 to 2000 as previously described [24, 28] and in S2 Appendix. For tandem MS/MS structural analysis, ten serum samples from each patient/control group (TBI “most affected”, control, and TBI plus CM, TBI minus CM) were selected randomly and analyzed in the ion-trap MS instrument as described previously [23, 24] and in S2 Appendix. Peak peptide/protein identifications were determined as described previously [23,24] and in S2 Appendix. Identified sequences were also searched using Basic Local Alignment Search Tool (BLAST) against NCBI human non-redundant database. Sequences were also compared against a *Taenia solium* database to help rule out non-specific identifications. For Ingenuity Pathway Analysis (IPA, QIAGEN Redwood City, “www.qiagen.com/ingenuity”), identified gene/protein names, when present in 3 or more sera samples out of 10, and their corresponding numbers of Identified MS/MS peptide sequence “hits”, were imported as log2 ratios for the control versus most affected TBI disorder comparison following similar work [23] and S3 Appendix. Proteins were also manually inspected for protein function and disease relatedness using PubMed/Medline. For IPA exhibited in Figs 6 and 7 in the main text, a neurological/immunological overview was incorporated into the IPA parameters used for those analyses. For IPA S1 and S2 Figs and in S3 Appendix, a TBI overview alone was incorporated for those figures. MS acquisition files are provided in S1–S17 Data. MS/MS acquisition files are provided in S18 Data.

### Statistical and data processing/analysis

Data analyses are described here and in more detail in S2 Appendix. Mass spectral data were exported into Microsoft Excel in a format providing rounded unit m/Z and intensity values, and normalized/scaled to the highest m/Z sum intensity value in segments of 10 m/Z from 400–2000. Leave one out [serum sample] cross validation (LOOCV, described previously, was used to distinguish mass peaks in serum samples between the various binary groups, for example to distinguish mass peaks between the “most affected”patient group vs the control individual group (“least affected) [24]. LOOCV is employed to help mitigate “over-fitting” of large data sets [29, 30]. Mass peak area differences between the various binary group comparisons used in the LOOCV analysis were identified as differing significantly at individual m/Z values using Student’s *t -*tests (one-tailed, unequal variance, significance designated at *p* < 0.05, see Fig 2 panel B). A peak classification value (PCV) metric is assigned at the area midpoint between the two group means of each m/Z peak differing significantly in the “left in” binary peak comparison dataset (e.g., peak 635, Fig 2 panel B). This midpoint is used to assign the group LOOCV classification for any “left out” peak in the LOOCV analysis. For example, if a “left out” samples identical m/Z peak has an area above this midpoint then that peak is classified as the larger peak area class. In the case of peak 635 this would be the most affected classification; if the area is equal to or below this value the peak classification would be “control”. In this way a % of significantly changed mass peaks classified (e.g., as most affected) is assigned each “left out” sample and plotted on the y axis vs the individual serum samples on the x-axis (e.g., in Fig 3 panel A). Randomization of serum samples from subject groups compared in binary fashion (another method to assess data over-fitting) was obtained using the RAND function in Excel 2016 but retaining group number and age ratios [31]. Upon randomization, the identical mass peak LOOCV database was created and the same analysis described above was performed. To obtain potential statistical powers for group sample sizes (ability to detect type II errors-false negatives), Cohen’s *d* effect size values are calculated from the binary group % LOOCV means and standard deviations in Table 2 [32, 33]. Statistical power using given sample sizes is calculated as described [33]. S1–S19 Tables provide spectral data, pre-averaged raw values and MS/MS data. Processing step result values are in S16, S17, S18 and S19 Tables.

**Table 2:** LOOCV serum mass profiling test metrics.

Group 1 vs. Group 2	Mean (SD) Group 1	Mean (SD) Group 2	TP	TN	FP	FN	*P*-Value, Cohen’s *d*	Random Grouping: *P*-Value	Figure #: panel
TBI (most affected), N = 21 vs. Controls, N = 20	58.16% (4.24%)	39.87% (3.80%)	21/21 (100%)	20/20 (100%)	0/20 (0%)	0/21 (0%)	1.3 x 10−^17^, 4.54	0.42	3: A & B
Training Set A: TBI (most affected), N = 16 vs. Controls, N = 15	58.50% (5.33%)	39.87% (4.07%)	16/16 (100%)	15/15 (100%)	0/15 (0%)	0/16 (0%)	1.4 x 10−^13^, 3.92	0.42	4: A & B
Blinded Set A: TBI (most affected), N = 16 vs. Controls, N = 15	51.90% (2.00%)	44.56% (3.28%)	5/5 (100%)	4/5 (80%)	1/5 (20%)	0/5 (0%)	2.1 x 10−^3^, 2.70	na	4: C
Training Set B: TBI (most affected), N = 15 vs. TBI, N = 12	60.80% (6.49%)	26.28% (5.18%)	15/15 (100%)	12/12 (100%)	0/12 (0%)	0/15 (0%)	1.50 x 10−^14^, 5.87	0.07	4: D
Blinded Samples B: (most affected), N = 6	46.71% (4.06%)	na	5/6 (83%)	na	na	1/6 (17%)	na	na	4: D
TBI; N = 12 vs. Controls, N = 20	73.13% (4.38%)	44.77% (4.31%)	12/12 (100%)	20/20 (100%)	0/20 (0%)	0/12 (0%)	2.81 x 10−^15^, 6.52	0.49	5: A
TBI +CM; N = 11 vs. Controls, N = 20	75.81% (5.28%)	40.91% (6.09%)	11/11 (100%)	20/20 (100%)	0/20 (0%)	0/11 (0%)	8.84 x 10−^15^, 6.12	0.04	5: B
TBI +CM; N = 11 vs. TBI; N = 12	67.41% (4.49%)	35.99% (7.62%)	11/11 (100%)	12/12 (100%)	0/12 (0%)	0/11 (0%)	1.98 x 10−^10^, 5.02	0.39	5: C

Mean of the % Total LOOCV Sera Mass Peaks (Standard Deviation [SD]); True Positives (TP); True Negatives (TN); False Positives (FP); False Negatives (FN); Traumatic Brain Injury (TBI); Chronic Migraine (CM); Post Traumatic Stress Disorder (PTSD); not applicable (na); TBI Most Affected (TBI, plus CM, plus PTSD, plus severe depression); Controls (minus TBI, no CM, minus PTSD, minus Depression); TBI +CM (TBI, plus CM, minimal/no PTSD, minimal/no Depression); TBI (TBI, minimal/no PTSD, no CM, plus minimal/no depression)

### Test metrics

The diagnostic value of a test/procedure is defined by its sensitivity, specificity, predictive value, and efficiency [34, 35]. Test sensitivity was determined from TP/(TP+FN) where TP was the number of true positives for disease presence, and FN was the number of false negatives for disease presence. Specificity was calculated from TN/(TN+FP) where TN is the number of true negatives and FP is the number of false positives. For example, TBI “most affected” and control TP, FP, TN, and FN values in Table 2 were determined using cutoffs of 2.28 standard deviations (SD) below the mean “% of TBI most affected LOOCV classified serum mass peaks, or 2.28 SD above the mean % control mass peaks (both cutoffs are represented by the single line in Fig 3 panel A).

## Results

### Using ESI-MS serum mass peak profiling to help distinguish “most affected” TBI patients from healthy control individuals

TBI in military veterans can occur in a clinical presentation involving other PCS sequelae including PTSD, SDep, and/or CM. It is important to study TBI and these sequelae together and individually to better understand these conditions and their interrelatedness and contributions to the overall clinical presentation. One goal of the present study is to examine whether the ESI-MS serum mass peak profiling methodology that has been successful in distinguishing early stage cancer patients from healthy controls as well as from different cancers, is able to distinguish TBI with PCS sequelae from healthy controls as well as distinguish TBI plus and minus PCS sequelae.

Fig 2 panel A is a flow chart outlining how serum mass peak profiling is employed to distinguish control individuals (least affected) from subjects with TBI, PTSD, SDep, and CM (most affected). The procedure requires a serum sample dilution (no pre-treatment or fractionation), and is a straight-forward biomolecule profiling platform. The complete mass peak database used here in Fig 2 (and also in Figs 3, 4 and 5, mean area values normalized) is provided in S6 Table. “Leave one out [serum sample] cross validation” (LOOCV) and group randomizations are employed to help mitigate and assess possible “over-fitting” of large data sets [29, 30]. Fig 2 panel B illustrates a small number of the significant ESI- MS mass peaks from 600 to 660 in m/Z units (mass divided by charge) able to discriminate sera from TBI “most affected” patients (solid line) from control individuals (dash line). This m/Z region is one of many analyzed (total range 400–2000 m/Z); the large number of significant peak differences likely is contributing to the disease discrimination ability of this technology [23, 25, 26]. These individual peak area means differ significantly (*p* value < 0.05) between 21 “most affected” patient sera samples and 20 control-“least affected” individual sera samples. Peak 635 exhibits a Peak Classification Value (PCV) metric area midpoint used for all peaks in the “left in” LOOCV database and is used to classify the group designations (either “most affected” or control-least affected) for the mass peaks in individual “left out” serum samples. A “left out” peak area above this midpoint is assigned to the higher value classifier (“most affected” for this peak, 635) and a “left out” peak equal to and below this midpoint is assigned to the lower value classifier (control for this peak). In this way individual “left out” serum samples are assigned a “% of total TBI most affected LOOCV classified mass peaks” or a “% of total control classified mass peaks” and that % value is plotted on the y-axis for each patient (x-axis, e.g., in Fig 3 panel A).

Fig 3 panel A illustrates the application of this LOOCV process for distinguishing patients with TBI “most affected” from healthy control individuals. When this “% of TBI most affected patient classified mass peaks value” (y axis) is plotted versus subject number (x-axis), a distribution plot is obtained (in which a clear demarcation is observed between most affected patients (darkened circles) versus controls (open squares). Individual LOOCV mass peaks identified and scored, on a per patient basis used in all the LOOCV binary group comparisons, are provided in S7–S10 Tables. The *p* value for this group discrimination is very low (10^−17^ range), and that value becomes non-significant (0.42) when these two different subject groups are mixed together in random fashion followed by the identical LOOCV mass peak analysis (Fig 3 panel B). This very large increase in *p* value upon inter-group sample randomization is consistent with minimal over-fitting of the original data set and with the presence of a physiological basis for the original TBI most affected versus control discriminations.

### Distinguishing sera of TBI “most affected” patients from controls via blinded “test” sample analyses

A blinded validation experiment of the discriminatory power of ESI-MS in distinguishing sera from most affected TBI patients from that of healthy controls was performed by removing randomly 5 TBI most affected patient sera and 5 control individual sera (10 total) from each of the N = 21 and 20 subject groups. A “training set” was then assembled with N = 16 for the “most affected” and N = 15 for the control group (“most affected” vs control) with significant peaks selection using the LOOCV procedure. Fig 4 panel A exhibits the ESI-MS discrimination of the training set for these two groups based on % of TBI most affected patient sera LOOCV mass peaks (y axis) classified for each TBI and each control serum sample (x axis). For the training database, a very low discriminatory *p* value is obtained (10^−13^ range), and a *p* value of 0.42 (non-significance) is observed upon sample randomization of the known pathology group with the control group followed by the same LOOCV analysis. The *p* value 0.42 random “non-discrimination” is exhibited in panel B, and results of this “leave 10 serum samples out” blinded pathology” test validation in panel C. As can be observed, 9 out of the blind group of 10 “left out” serum samples were classified correctly with only one false positive for “most affected” (solid circle, above cut off value of 46.1% of “most affected” classified mass peaks). Another type of blinded analysis was performed by randomly removing 6 of the “most affected” samples and then test those samples against the same selected peak and area values from the training database constructed using the previously described LOOCV method on a “most affected” (N = 15) and TBI only (N = 12) patient set. Resulting data of the blinded sample testing are reported in Fig 4 panel D along with the training database patient scores (“most affected” in solid bars; TBI only solid squares) against the cutoff value of 55.7%. Five out of six samples were identified correctly (open triangles).

### ESI-MS serum discrimination of patients with TBI plus or minus chronic migraine from each other and from control individuals

In order to examine the contribution of CM to the overall TBI clinical presentation, and conversely, to single out TBI and the CM phenotype from each other, the ability of ESI-MS serum mass profiling to discriminate these groups as well as controls was assessed. Exhibited in Fig 5 are binary ESI-MS LOOCV comparisons between serum samples from 20 control individuals, 12 TBI patients with no CM, and 11 TBI patients plus CM. Panel A of Fig 5 depicts ESI-MS distinguishing the TBI no CM patient serum sample set from the control serum set. A very low “true pathology” discrimination is obtained (*p-*value 10^−15^) with a random grouping value of 0.49. Discriminating serum samples from patients with TBI plus CM from control individuals is exhibited in panel B. One control false positive is observed (darkened square). The true pathology group discriminatory *p* value is very low (10^−15^) but the random grouping value is slightly significant (0.04). Plotting this still significant group value in panel C indicates no real group discrimination is taking place (samples are indicated as true positives for the random (RND) TBI + CM group if above the solid line and also indicated as true negatives for the RND Control group if below the dashed line), as samples are identified as members of both random groups. Importantly, the ESI-MS profiling methodology is able to discriminate serum from TBI patients with CM (dashes) from TBI patients without CM (darkened squares, panel D). The true pathology *p* value is quite low (10^−10^) and the random grouping value is non-significant (0.39).

### Test metrics

Table 2 is a summary of the test metrics for the discriminatory mass peak data exhibited in Figs 3, 4 and 5. These metrics include the % LOOCV classified mass peak means and standard deviations (SD) for all the group comparisons (far left column) with respect to the specific figure panels (far right column). Nomenclature from predictive value theory is presented, e.g., test sensitivity, specificity, etc., as well as true pathology and random grouping *p* values [34, 35]. The pathological groups tested in binary fashion from these Figures are listed in the far-left column. The “% LOOCV MS peaks” means and their standard deviation (SD) are all well separated and have narrow SD boundaries for all the groups tested. Test sensitivities (true positive rate) range from 1.0 to 0.83 and test specificities (true negative rate) range from 1.0 to 0.80 for all groups. Physiological values in the original distribution differences are indicated by the very large increases in *p* values when the groups are randomized. A Cohen’s *d* effect size value is provided in Table 2. “Effect size” refers to the mean differences for the binary group LOOCV discriminations observed in Figs 3, 4 and 5, taking into consideration the SD values [32]. This Cohen’s *d* value is an indirect measure of statistical power (ability to detect type II errors-false negatives) of the sample sizes employed in a study. The large Cohen’s *d* values exhibited here bolster the reliability and power (estimated from these effect sizes to be > 0.90) for the sample sizes used in this study (Table 2).

### Serum peptide/protein identifications using MS/MS of LOOCV TBI “most affected”, CM, and control group discriminatory mass peaks and bioinformatics cell pathway analysis

Table 3 exhibits MS/MS identified peptides listed by their protein name/abbreviations, their serum presence, and their numbers of MS/MS “hits” for the Fig 3 panel A TBI “most affected” versus control “least affected” binary LOOCV comparison (panel I, top 48 proteins) and for the Fig 5 panel D TBI plus and minus CM comparison (pane II, top 48 proteins). Individual selected LOOCV mass peaks identified and analyzed by MS/MS, on a per patient basis used in all the LOOCV binary group MS/MS comparisons, are provided in S11–S15 Tables. Notable serum peptide/protein changes (levels up or down in the two comparative groups) observed in panel I [shaded] with respect to post-traumatic neurological issues include GRM4 (glutamate receptor-4) and PCLO (protein Piccolo) which were previously suggested as biomarkers for major depression and possibly PTSD [36–38]. VWF (Von Willebrand Factor) was previously shown to have a role in maintaining blood brain barrier (BBB) flexibility, and ATRN has roles in myelination events which have been shown to be important in BBB protection and maintenance [39–41]. LRP1 is a major regulator of blood-brain barrier integrity [42]. Genetic evidence also indicates LRP1 is a susceptibility factor for migraine headache [43]. In addition, changes in LRP1 may have roles in dementia and Alzheimer’s disease (AD) progression, as does NOTCH4 [44, 45]. TBI has previously been shown to increase risks for dementia including AD in some cases 30 plus years after the initial injury [16, 25]. Of added interest, an autoimmunity pathway is present in Panel I with proteins LRP1 and POLA1 [46, 47]. Panel II exhibits the peptides/proteins identified by MS/MS whose serum levels are changing in TBI plus CM versus TBI minus CM patient comparison. A number of protein similarities are observed with Panel I including LRP1, a marker for migraine headache, and PCLO a possible marker for depression [37, 38, 43]. Also with respect to migraine is ITGB3 which was shown to influence serotonin blood levels, serotonin being previously implicated in migraine etiology [48, 49]. Dementia and AD potential markers are present in panel II, RXFP1, RELN, EGF and the previously mentioned LRP1 [50, 51]. TBI and dementia associations were previously demonstrated [16, 22]. HUWE1 mediated Notch signaling is involved in neuroprotection after injury [52]. Like in panel, I autoimmunity appears to be in play with proteins AFF3, SMYD3, and LRP1 [46, 53, 54]. Also of interest in panel II is autophagy with DUOX1, a protein involved in autophagic degradation pathways [55].

**Table 3:** Peptides/proteins identified using LOOCV discriminatory mass peaks in TBI+PTSD+CM+SDep versus controls and TBI+CM versus TBI.

**Least affected vs. Most affected (no. sera/10, no. MS/MS “hits”-least: no. MS/MS “hits” -most)**			
IGH (8, 9: 38)	SYNE1 (3, 57: 0)	TENM2 (3, 23: 11)	SIMC1 (3, 0: 15)
TTN (5, 54: 57)	FBN2 (3, 17: 32)	ATRN (3, 27: 0)	TNRC18 (3, 15: 0)
SSPO (4, 36: 44)	IGK (3, 21: 27)	PRUNE2 (3, 27: 0)	TCF20 (3, 13: 0)
NOTCH4 (4, 38: 36)	FAT4 (3, 13: 33)	FBN3 (3, 18: 8)	POLA1 (3, 8: 0)
IGL (4, 39: 26)	GRM4 (3, 0: 41)	ITGA8 (3, 0: 22)	DNAJC5 (2, 155:18)
PCLO (4, 4: 37)	LRP1 (3, 7: 33)	MALRD1 (3, 0: 19)	ADGRL2 (2, 171:0)
TRB (4, 15: 11)	MT-ND4 (3, 23: 16)	CSMD1 (3, 0: 17)	DNAJC5B (2, 6:151)
MUC19 (4, 0: 17)	HECTD4 (3, 23: 15)	PKD1 (3, 0: 16)	OTOGL (2, 38:90)
SON (3, 95: 0)	ZNRF3 (3, 36: 0)	C18orf15 (3, 15: 0)	EBF4 (2, 62:38)
LAMA5 (3, 39: 52)	NSD1 (3, 0: 35)	CNNM1 (3, 0: 15)	MUC2 (2,0:62)
MUC5AC (3, 24: 49)	USH2A (3, 19: 16)	PTPRM (3, 0: 15)	VWF (2, 13:45)
ZNF268 (3, 28: 34)	FCGBP (3, 22: 2)	SERINC2 (3, 0: 15)	TENM4 (2, 32:26)
**TBI—CM vs TBI + CM (no. sera/10, no. MS/MS “hits”-CM: no. MS/MS “hits” + CM)**			
IGH (5, 31: 5)	MUC5B (3, 0:16)	PCLO (2, 0: 38)	DNAH9 (2, 20: 0)
MUC19 (4, 13: 12)	LSM12 (3, 9:0)	MAN1B1 (2, 36: 0)	IGFN1 (2, 6: 20)
EBF4 (3, 9: 107)	PCDH8 (3, 16: 0)	DKC1 (2, 32: 0)	ITGB3 (2, 19:0)
SYNE2 (3, 19: 75)	TRB (3, 14: 6)	SMYD3 (2, 21: 9)	SBF1 (2, 18: 0)
FBN2 (3, 76: 12)	RUNX2 (3, 13: 0)	RNF219 (2, 0: 28)	RELN (2, 0:18)
MT-ND5 (3, 12: 75)	MUC6 (3, 10: 0)	ARID1B (2, 28: 0)	PLOD1 (2, 0: 16)
IGL (3, 23: 46)	AFF3 (2, 200: 0)	FREM2 (2, 26: 0)	SLC12A1 (2, 16: 0)
TTN (3, 13: 41)	MT-ND1 (2, 20: 85)	CCDC18 (2, 24: 0)	DUOX1 (2, 0: 15)
HUWE1 (3, 33: 0)	PXN (2, 0: 45)	PADI3 (2, 24: 0)	TANC1 (2, 15: 0)
RXFP1 (3, 0: 21)	MAPRE2 (2, 42: 0)	ZKSCAN7 (2, 0: 23)	EGF (2, 5: 14)
TNRC6B (3, 34:14)	LAMA1 (2, 0:41)	ZNF571 (2, 0: 23)	LRP1 (2, 14: 4)
CCDC148 (3, 29:15)	ING2 (2, 40: 0)	PSD3 (2, 22: 0)	PLP1 (2, 0:14)

Shaded cells indicate neurological relationship

Fig 6 exhibits cellular/biochemical pathways that the bioinformatic software Ingenuity Pathway Analysis (IPA, QIAGEN Redwood City) indicated are being affected by including the list of 48 proteins in the TBI “most affected” versus “controls” exhibited in Table 3, panel I plus the next 58 ranked proteins not listed in that table. Neurological and immunological focuses were programmed into the IPA software pathway search for both Figs 6 and 7. Also included in this pathway analysis as well as in Fig 7 are related functional inputs from literature searches on the 108 proteins described above using Medline and PubMed. Major pathways affected (by numbers of connections) in this TBI “most affected” versus control comparison include immune responses, central nervous system, neuron development/vision, and dementia/Alzheimer’s disease. Also a depression pathway is identified involving aforementioned proteins GRM4, PCLO, plus protein PTPN5/STEP which has previously been associated with major depression and neuroinflammation [56]. As noted above a blood brain barrier phenotype is present as well as autoimmunity and autophagy with ANO2, HLA-DQB1, MTMR3, and SEC16A [57–60]. IPA analysis of the 48 proteins listed in panel II plus the next 58 ranked proteins for the TBI plus and minus CM comparison (Fig 5 panel D) is displayed in Fig 7. Major pathways identified as affected in this binary group comparison include immune responses, brain injury, dementia, and ion transport, similar to that observed in Fig 6. In addition, a migraine headache pathway of related proteins also appears in this comparison, most notably ITGB3, KCNK18, NOTCH4, and LRP1 which could be interacting with glutamate receptors [43, 48, 61–63]. Also the autoimmunity connections are present with AFF3, SMYD3, and LRP1 as well as autophagy with DUOX1, TECPR1, and ZHHX3 [64, 65]. It is noted that the IPA analysis illustrated in Figs 6 and 7 also include protein inputs from literature searches on disease relatedness of the proteins exhibited in Table 3 as well as the next top 58 proteins. IPA analysis including these proteins described above with a TBI focus is exhibited in S1 and S2 Figs. A TBI focus alone yields fewer pathway connections but possibly more direct information concerning the TBI condition. Additional IPA results and discussion and detail are provided in S4–S6 Appendices.

## Discussion

Much research in the TBI and PCS field is focused on identifying short-term changes associated with TBI, while less attention has been paid to the long-term effects on patients. By observing individuals 5–14 years after the initial TBI, this present study focuses on the long-term physiological changes of PCS potentially induced by the TBI. By studying such long-term effects, it might be possible to obtain clues about mechanisms responsible for long-term persistence of PCS symptoms like chronic migraine headache. There are a number of unique aspects of this molecular and translational long-term study. The current experiments employ well-controlled subject group comparisons. The initial goal was to provide groups of D-TBI subjects who were recruited randomly from a listing, provided by the VA VISN 19 Data Repository, of approximately 6,000 Operation Enduring Freedom/Operation Iraqi Freedom (OEF/OIF) veterans who had suffered a confirmed D-TBI, and control veterans drawn from the same repository of approximately 12,000 OEF/OIF individuals who did not have a D-TBI. Thus, the non-TBI group of deployed veterans experienced the same war theater conditions as the veteran volunteers who suffered a deployment-related TBI. This also holds for the deployed veterans who did not manifest PCS associated PTSD, severe depression, and/or CM versus those deployed veterans who did. And as described above, these D-TBI veterans are in the persistent phases of their PCS disorders (5–14 years after their D-TBI) versus most previous studies on these disorders were in the earlier initial acute phase (less than 90 days). Therefore, veteran sera biomolecule analyses performed in this study should be able to provide additional clues concerning the underlying mechanisms for maintaining such persistence. Patient health and demographic data are provided in Table 1 in the main text and in S1–S3 Tables. The minimal serum mass profiling platform utilized in this study, employing leave one out (serum sample) cross validation [LOOCV] was able to uniquely discriminate groups of TBI and PCS sequelae patients from each other and from controls, as well as provide a set of discriminatory LOOCV serum mass peaks whose identification should provide additional mechanistic clues about the biochemical nature of these disorders.

With respect to these patient groupings, it is important to develop such robust, accurate, and minimally invasive testing aids to assist researchers and clinicians in diagnosing, classifying, and monitoring individuals who sustained a TBI and developed PCS sequelae. Such aids will allow more vigilant screening of these conditions, helping with patient prognoses and treatments, and assist in the understanding of these disorders. This study reports progress in distinguishing individual serum samples from patients with D-TBI and post-concussion sequelae including PTSD, CM, and severe depression from each other and from control individuals, in retrospective and blinded fashion with high sensitivity and specificity test metrics (Table 2) 5–14 years after the initial TBI. In looking for possible independent variable effects on our LOOCV binary group comparisons, we assessed a number of such variables using R^2^ variance analysis. We were not able to demonstrate a consistent R^2^ association (observed range 0.0001 to 0.145) between the number of TBIs per patient and influence on the LOOCV binary comparison results. The one comparison with the higher R^2^ value (0.749) was the blind analysis in Fig 4 panel C containing a small sample size which likely influenced the results. This information is provided in S4 Table. An R^2^ analysis was performed showing no effect of age on any of the LOOCV binary comparisons in the study (observed range 0.0014 to 0.1998, S6 Table). Six of the 162 patients were females, and two were included in the present analysis of 65 volunteers in the Figures (one in Figs 3 and 4, and a different one in Fig 5). The remaining study subjects were males. The two female volunteers with TBI segregated with the male volunteers with TBI in the binary analyses. There were not enough females to make a direct male to female comparison. It was difficult to accumulate large N values for the matched groups in this study. However, the large “effect sizes” for these binary group comparisons (differences in mean mass peak areas/standard deviations for the two different groups in question which is proportional to statistical power-ability to detect false negatives) does lend credibility to the success of these discriminations even at the reduced sample sizes, and helps establish their validity and portends well for future studies with larger sample sizes [32]. Although the present study is a retrospective analysis, experiments were performed to begin to address the ability of this test procedure to discriminate blinded samples against a training set. In one blind test set (equal numbers of TBI “most affected” samples and controls) an analogous training set was able to correctly identify 9 out of 10 samples (Fig 4 panel C).

With respect to identifying underlying physiological process at play in these disorders, this ESI-MS platform also has the ability to “target” the unique binary group discriminatory set of LOOCV mass peaks for MS/MS structural determination which can lead to biochemical and phenotype elucidations. This represents a third and another unique aspect of this ESI-MS methodology: disease understanding through identification of a wide variety of biomolecules involved in disease mechanisms which can be performed with a very small sample of minimally-invasive bodily fluid. The identification of such biomolecules and biochemical pathways can aid in further biomarker and therapeutic development. Such analyses are presented in Table 3 and in Figs 6 and 7 for the TBI “most affected” versus controls and the TBI plus and minus CM comparisons, respectively. The biochemical/cellular pathway phenotypes identified playing a role in these disorders include inflammation/immune responses, neurological issues, and dementia/Alzheimer’s disease inferences in Fig 6 and Fig. These observations are in line with previous evidences that TBI and associated post-concussion sequelae are triggering neuro-inflammation events, and the dementia/AD similarities observed in this study were noted before. Observing these known phenotypes lends credence to the ability of this serum profiling methodology and platform to help decipher these pathologies. These results indicate that we are separating/distinguishing these different groups based on actual physiological and pathological differences which is consistent with the serum mass profiling hypothesis guiding these studies. Such disease differences reflected in the peripheral blood likely stem from systemic processes in response to such disease states as well as direct diseased tissue inputs [66, 67].

More novel and specific biochemical/cellular pathway affects observed in this study include a potential blood brain barrier (BBB) phenotype in the TBI “most affected” vs control and in the TBI plus and minus CM comparisons (Table 3, Figs 6 and 7). These observations about BBB effects in the present TBI study are in line with a previous analysis concerning effects of concussion on the BBB in humans and in rats [68]. Notably, a depression phenotype was observed in the TBI “most affected” vs control comparison (Table 3, Fig 6), and a migraine headache phenotype was observed in the TBI plus vs minus CM comparison (Table 3 and Fig 7). With respect to the persistence phenomenon associated with studies on the long-term pathologies of patients with TBI and associated PCS, the autoimmunity phenotype appearing in both the TBI “most affected” versus controls and the TBI plus versus minus CM IPA analyses (Figs 6 and 7) could provide a potential explanation for such persistence. Autoimmunity has recently been associated with stress-related disorders, and is known to have roles in other chronic diseases [69]. The long term persistence of TBI and associated PCS sequelae can be described as chronic diseases. Also the autophagy phenotype (cell degradation of intra-cellular constituents) is appearing in both Figs 6 and 7 IPA analyses, and autophagy was previously reported as a possible mechanism underlying TBI [70]. And of added interest, autophagy and autoimmunity are linked biochemically and physiologically [71]. This combination would be an ideal mechanism(s) to promote the persistence seen in long-term patients with TBI PCS related sequelae. By providing initial evidence of potential autoimmunity and autophagy mechanisms, this study provides basic observations which could open up new avenues of thought and future possible research concerning TBI and associated PCS sequelae including chronic migraine.

## Supporting information

S1 Fig IPA analysis of TBI “most affected” patients versus control subjects. Affected physiological/cellular pathways and serum protein assignments from Table 3 (main text) that were found to distinguish TBI most affected patients from control individuals. The next top 58 proteins for each group (TBI most affected or control) not exhibited in Table 3 were added to the 48 in this Table for this analysis. Analysis performed by using Ingenuity Pathway Analysis (IPA) bioinformatics software (Qiagen, Inc.) using a “TBI” focus in the software.
(TIF)

S2 Fig IPA analysis of TBI plus CM patients versus TBI minus CM patients. Affected physiological/cellular pathways and serum protein assignments from Table 3 that were found to distinguish patients with TBI alone versus patients with TBI plus CM. The next top 58 proteins for each group (TBI alone and TBI plus CM) not exhibited in Table 3 were added to the 48 in this Table for this analysis. Analysis performed by using Ingenuity Pathway Analysis (IPA) using a “TBI” focus in the software.
(TIF)

S1 Appendix Methods continued. TBI and PCS patient assessment and classification.
(DOCX)

S2 Appendix Methods continued. Electrospray mass spectrometry of sera from patients with TBI and PCS and healthy controls.
(DOCX)

S3 Appendix IPA figures and legends. S1 and S2 Figs. IPA analysis of TBI “most affected” patients versus control subjects with legends.
(DOCX)

S4 Appendix IPA results continued. Cell pathways incorporating serum protein changes identified from MS/MS of LOOCV mass peaks discriminating TBI “most affected”, CM, and control subject groupings using IPA software focus on “TBI”.
(DOCX)

S5 Appendix Discussion continued. (DOCX)

S6 Appendix Materials and methods continued. Patient characteristics.
(DOCX)

S1 Data MS raw acquisition files ThermoFinnigan/ThermoFisher raw file format (1 of 17). (ZIP)

S2 Data MS raw acquisition files ThermoFinnigan/ThermoFisher raw file format (2 of 17). (ZIP)

S3 Data MS raw acquisition files ThermoFinnigan/ThermoFisher raw file format (3 of 17). (ZIP)

S4 Data MS raw acquisition files ThermoFinnigan/ThermoFisher raw file format (4 of 17). (ZIP)

S5 Data MS raw acquisition files ThermoFinnigan/ThermoFisher raw file format (5 of 17). (ZIP)

S6 Data MS raw acquisition files ThermoFinnigan/ThermoFisher raw file format (6 of 17). (ZIP)

S7 Data MS raw acquisition files ThermoFinnigan/ThermoFisher raw file format (7 of 17). (ZIP)

S8 Data MS raw acquisition files ThermoFinnigan/ThermoFisher raw file format (8 of 17). (ZIP)

S9 Data MS raw acquisition files ThermoFinnigan/ThermoFisher raw file format (9 of 17). (ZIP)

S10 Data MS raw acquisition files ThermoFinnigan/ThermoFisher raw file format (10 of 17). (ZIP)

S11 Data MS raw acquisition files ThermoFinnigan/ThermoFisher raw file format (11 of 17). (ZIP)

S12 Data MS raw acquisition files ThermoFinnigan/ThermoFisher raw file format (12 of 17). (ZIP)

S13 Data MS raw acquisition files ThermoFinnigan/ThermoFisher raw file format (13 of 17). (ZIP)

S14 Data MS raw acquisition files ThermoFinnigan/ThermoFisher raw file format (14 of 17). (ZIP)

S15 Data MS raw acquisition files ThermoFinnigan/ThermoFisher raw file format (15 of 17). (ZIP)

S16 Data MS raw acquisition files ThermoFinnigan/ThermoFisher raw file format (16 of 17). (ZIP)

S17 Data MS raw acquisition files ThermoFinnigan/ThermoFisher raw file format (17 of 17). (ZIP)

S18 Data MS/MS raw acquisition files ThermoFinnigan/ThermoFisher raw file format (1 of 1). (ZIP)

S1 Table Patient characteristics continued. (DOCX)

S2 Table Patient health history. (DOCX)

S3 Table Years since D-TBI. (DOCX)

S4 Table Relationship between D-TBI number and % LOOCV Patient scores. (DOCX)

S5 Table Volunteer specifications by pay grade. (DOCX)

S6 Table % Patient LOOCV score vs age. (DOCX)

S7 Table Normalized peak area mean for each patient by m/Z used for figures and tables. (DOCX)

S8 Table Patient % classified LOOCV mass peak scores plotted in presented figures. (DOCX)

S9 Table Samples used in each figure. (DOCX)

S10 Table Mass peaks selected by LOOCV process. MS peaks used by figure number.
(DOCX)

S11 Table LOOCV MS/MS peaks utilized. Mass peaks selected for MS/MS analysis in Figs 6 and 7.
(DOCX)

S12 Table Peptides identified using MS/MS by patient: TBI most affected vs control (least affected). (DOCX)

S13 Table Peptides identified using MS/MS by patient TBI + CM vs TBI. (DOCX)

S14 Table Proteins identified MSMS analysis TBI (most affected, N = 10) vs control (least affected, N = 10) MS/MS results. (DOCX)

S15 Table Proteins identified MSMS analysis TBI + CM (N = 10) vs TBI (N = 10) MS/MS results. (DOCX)

S16 Table Normalized peak data for averages. (XLS)

S17 Table Peaked data. (XLS)

S18 Table Normalized raw spectral data. (XLS)

S19 Table Raw spectral export. (XLS)
