# Supplementary material for: Distinguishing and phenotype monitoring of traumatic brain injury and post-concussion syndrome including chronic migraine in serum of Iraq and Afghanistan war veterans
Source: PLoS One. 2019 Apr 26;14(4):e0215762. doi: 10.1371/journal.pone.0215762 (PMC6485717; doi:10.1371/journal.pone.0215762)
Supplement: S1 Appendix — TBI and PCS patient assessment and classification. (DOCX) [file pone.0215762.s003.docx]

**S1. Appendix Methods continued. *TBI and PCS patient assessment and classification.***

D-TBI was graded by duration of loss of consciousness (LOC) as follows: very mild (VMTBI) – dazed only; mild (MTBI) – LOC of 1-30 minutes; moderate – LOC 30-360 minutes, and severe – LOC >6 hours. As there were relatively few subjects with LOC > 30 minutes, all DTBI subjects with LOC > 30 minutes were put in a group labelled Moderate-Severe TBI (MSTBI). The majority of the D-TBIs occurred in a combat situation and were graded as mild TBI. Initial TBI assessment was from a Medic or other non-physician medical personnel and generally not by a physician. Glasgow Outcome Scores are not available. The duration of LOC was obtained from self-assessment of the subject through inquiry concerning his/her recollections about the incident, including estimated time of LOC as reported by others to the patient, and an estimation of the duration of LOC based upon when the subject felt his/her memory returned to normal. Self-inquiry included asking whether the subject remembered the blast or direct head trauma (DHT), recalled participating in post-TBI activities such as setting up a defense perimeter around a disabled vehicle, recalled returning to base, and recalled any medical evaluation and recommendations when the subject returned to full duty. Exact determination of the duration of post-traumatic amnesia was not always possible unless provided by information from an observer or a specific notation in the medical record. Inclusion criteria for TBI subjects participation in this study included: (a) deployed to OEF/OIF, (b) occurrence of a TBI while deployed (D-TBI), (c) absence of any major organ disease chronic degenerative or inflammatory disease, or morbid obesity (Diabetes, hypertension and hyperlipidemia were allowed if treated and under control), and (d) willing to participate in the study. Inclusion criteria for control subjects (CS) were the same as (a), (c), and (d) above, as well as absence of any D-TBI.

Headache classification followed criteria of the International Classification of Headache Disorders (ICHD) and included migraine, probable migraine, tension-type, or no headache (HA) ^1^. CM is defined as ≥15 HA/month with ≥8 days/month of migraine with any other HA days usually classified as Tension-Type HA (TTH) or probable migraine ^2^. Chronic Tension-Type Headache (CTTH) is defined as occurrence of ≥15 HA/month with ≤7 migraine days/month and with the majority being TTH. Infrequent Headache (IFH) is defined as 2-9 HA/month of migraine and TTH type. Very infrequent Headache (VIFH) is ≤1 HA/month. For PTSD and depression assessments, PTSD civ score ranges were assessed as ≤ 35- no or minimal PTSD, 36-49- possible PTSD, and ≥ 50 - highly probable PTSD. For depression, the BDI 2 score ranges employed were ≤ 11 - no or minimal depression, 12-19 - mild depression, 20-28 - moderate depression, ≥ 29 - severe depression (SDep). The Breslau 7 question PTSD questionnaire was also employed and analyzed for compatibility of PTSD scoring. The Breslau QS has 7 questions which are answered in a yes/no manner. To score this questionnaire, the number of “yes” answers are tabulated as follows: 1-3 yes – no PTSD, 4, 5 yes – possible PTSD, and 6, 7 yes – Highly probable PTSD. In a preliminary analysis of our data using 125 subjects, the correlation between the PTSD civ and The Breslau QS was 0.72 (Pearson coefficient). The control subjects, without D-TBI, were recruited from the dataset provided by the VISN 16 and the VISN 19 Data Repositories. All D-TBI subjects in the recruited patients experienced D-TBIs between 4 and 15 years prior to entering this study. Demographic and health information recorded included age, sex, BMI (body mass index), hypertension, tobacco use, alcohol use, prescription drugs, headache type and frequency, hyperlipidemia, and other health issues (e.g., diabetes, arthritis, cancers, cardiovascular disease). Subjects with major organ disease, significant vascular disease, cancer, chronic inflammatory or autoimmune diseases or morbid obesity found on screening available medical records or found on the interview were excluded from this study. Subjects with history of diabetes, hypertension or hyperlipidemia which were under treatment and under control were allowed in the study.

**Bibliography:**

1. Bes A, Kunkel R, Lance JW, Nappi G, Pfaffenrath V, Rose FC, et al. The International Classification of Headache Disorders, 3rd edition (beta version). Cephalalgia. 2013 Jul;33(9):629-808. PubMed PMID: WOS:000320368000002. English.

2. Olesen J. Headache Classification Committee of the International Headache Society (IHS) The International Classification of Headache Disorders, 3rd edition Asbtracts. Cephalalgia. 2018 Jan;38(1):1-211. PubMed PMID: WOS:000425149500001. English.
