# Supplementary material for: Distinguishing and phenotype monitoring of traumatic brain injury and post-concussion syndrome including chronic migraine in serum of Iraq and Afghanistan war veterans
Source: PLoS One. 2019 Apr 26;14(4):e0215762. doi: 10.1371/journal.pone.0215762 (PMC6485717; doi:10.1371/journal.pone.0215762)
Supplement: S2 Appendix — Electrospray mass spectrometry of sera from patients with TBI and PCS and healthy controls. (DOCX) [file pone.0215762.s004.docx]

**S2 Appendix. Methods continued: Electrospray mass spectrometry of sera from patients with TBI and PCS and healthy controls.**

Triplicate mass spectra were collected from diluted sera of TBI patients or control individuals in random fashion. Spectra were sampled at a m/Z (mass divided by charge) resolution of two hundredths over a m/Z range of 400 to 2000. Positive ion mode spectra were collected in a 30 min time-frame for each injection. Raw spectral data were extracted using the manufacturer's software "Qual Browser" version 1.4SR1. For tandem MS/MS, sera were selected randomly and analyzed in the ion-trap MS instrument via Selected Reaction Monitoring (SRM) MS/MS without chromatographic separation of sera. Thirty six LOOCV discriminatory mass peaks in the m/Z range 539-995 were analyzed for the binary comparison TBI most affected versus controls, and thirty six LOOCV mass peaks in the range 509-1076 were analyzed for the TBI plus or minus CM comparison. Peak peptide/protein identifications were determined using SEQUEST Proteome Discoverer 1.0 (ThermoFisher) using the “no cleavage” setting on a Homo sapiens database created through the Discoverer software from a non-redundant database downloaded from NCBI on 07/08/2014. MS/MS identification of peptides/proteins from samples required a minimum of 2 unique peptides and a cross correlation minimum X-correlation of 1.7 or greater, in line with previous studies.

**Statistical and data processing/analysis**

Mass spectral data were exported into Excel in a format providing rounded unit m/Z and intensity values. Data were locally normalized/scaled to the highest m/Z sum intensity value in segments of 10 m/Z from 400-2000. MS spectral peak assignments were calculated as centroid m/Z peak area values (valley to valley) using Mariner Data Explorer 4.0.0.1 software (Applied BioSystems). Centroid area is defined as the area of the peak calculated from its geometrical m/Z center. It is possible to identify an m/Z value with a peak area = 0. These values are retained in the calculations and not considered missing data. Leave one [serum sample] out cross validation (LOOCV) was used to distinguish serum samples between binary groups, for example, “most affected“ patients vs control individuals (least affected). In this LOOCV analysis, the collective serum spectra mass peak areas between groups (e.g., most affected vs control-least affected) were analyzed for significant differences at individual m/Z values using Student’s *t -*tests (one-tailed, unequal variance, significance designated at p < 0.05), leaving out a different sample (most affected or control) in succession to build each unique N – 1 LOOCV “left in” significant mass peak dataset. All significant peaks utilized for these separations were at least 0.3% of the normalized maximum peak area. The mass peaks of each “left out” sample are then compared using peak area to all the “left in” mass peaks in their unique N-1 LOOCV dataset. This area comparison involves the use of a peak classification value (PCV) metric at each significant “left in” peak of the LOOCV dataset. Whether a “left out” peak area falls above or below this midpoint metric determines its classification. For example, in Fig 2 peak 635 is classified as a “most affected” peak in the “left in” database. If the 635 peak from the “left out” sample has a peak area above the PCV then it is classified as a “most affected peak”. If it falls below this PCV then the “left out” peak is classified as a “control, least affected peak”. Such peak classifications are performed for all “left out” peaks in all “left out” serum samples against their respective N-1 “left in” LOOCV mass peak databases. Of note, a control patient sample can have a certain percentage of “most affected peaks” because some of the control peaks at particular m/Z values can have areas above PCV values at particular “most affected” classified m/Z peaks. In this way a % of mass peaks classified (e.g., as most affected) is assigned each “left out” sample and plotted on the y axis vs the individual serum samples on the x-axis (e.g., in Fig 3). Randomization of serum samples from subject groups being compared in binary fashion was obtained using the RAND function in Excel and manually balanced to retain age distribution and sample number in each of the random groups. Upon randomization, the identical mass peak LOOCV analysis was performed as described above. To obtain potential statistical powers for group sample sizes (ability to detect type II errors-false negatives), Cohen’s *d* effect size values are calculated from the binary group % LOOCV means and standard deviations in Table 2. Statistical power using given sample sizes is calculated as described.
