# Supplementary material for: Distinguishing and phenotype monitoring of traumatic brain injury and post-concussion syndrome including chronic migraine in serum of Iraq and Afghanistan war veterans
Source: PLoS One. 2019 Apr 26;14(4):e0215762. doi: 10.1371/journal.pone.0215762 (PMC6485717; doi:10.1371/journal.pone.0215762)
Supplement: S3 Appendix — S1 and S2 Figs. IPA analysis of TBI “most affected” patients versus control subjects with legends. (DOCX) [file pone.0215762.s005.docx]

**S3 Appendix. IPA figures and legends:
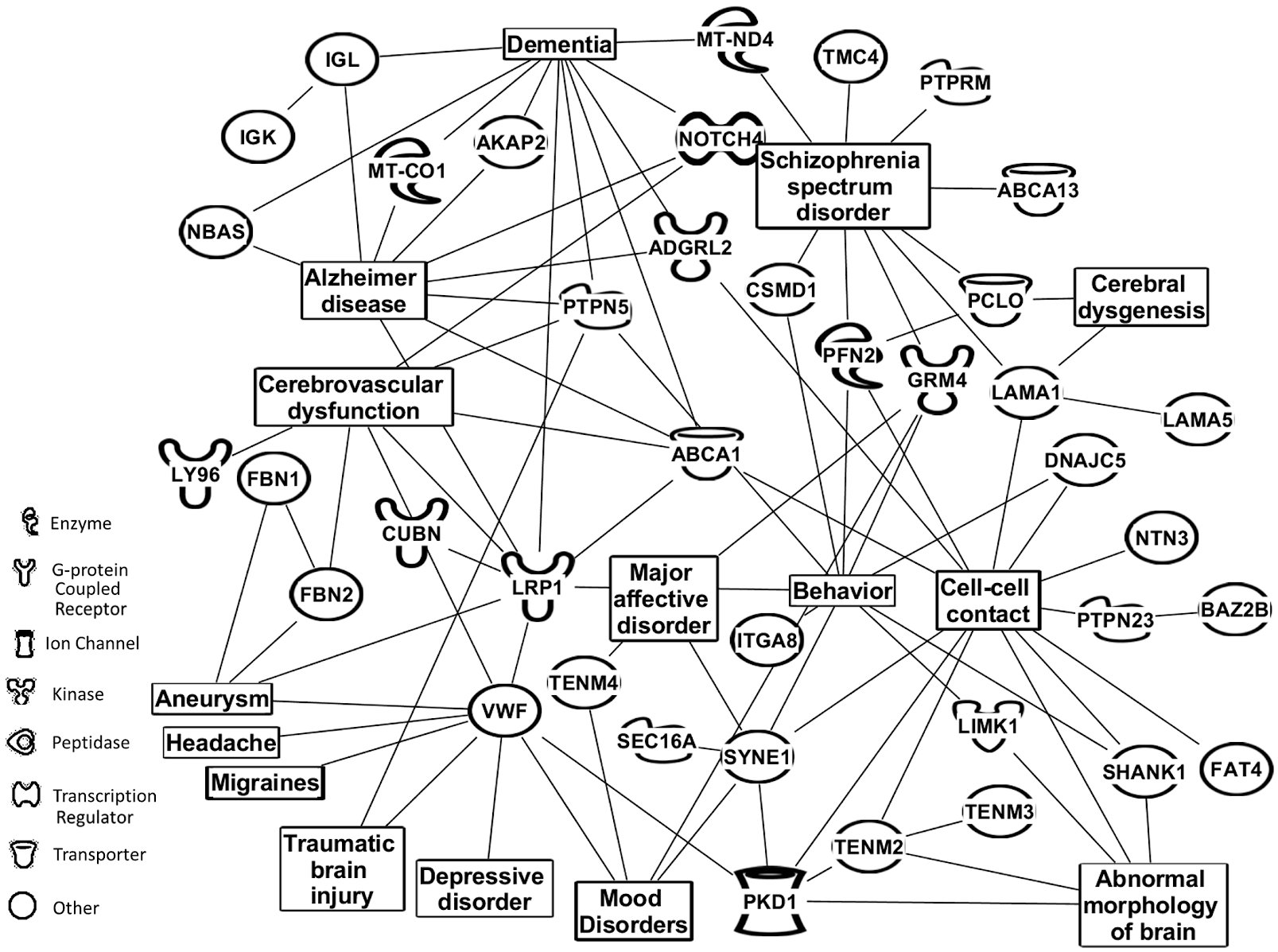
S1 and S2 Figs. IPA analysis of TBI “most affected” patients versus control subjects with legends.**

**S1 Fig. IPA analysis of TBI “most affected” patients versus control subjects.** Affected physiological/cellular pathways and serum protein assignments from Table 3 (main text) that were found to distinguish TBI most affected patients from control individuals. The next top 58 proteins for each group (TBI most affected or control) not exhibited in Table 3 were added to the 48 in this Table for this analysis. Analysis performed by using Ingenuity Pathway Analysis (IPA) bioinformatics software (Qiagen, Inc.) using a “TBI” focus in the software.


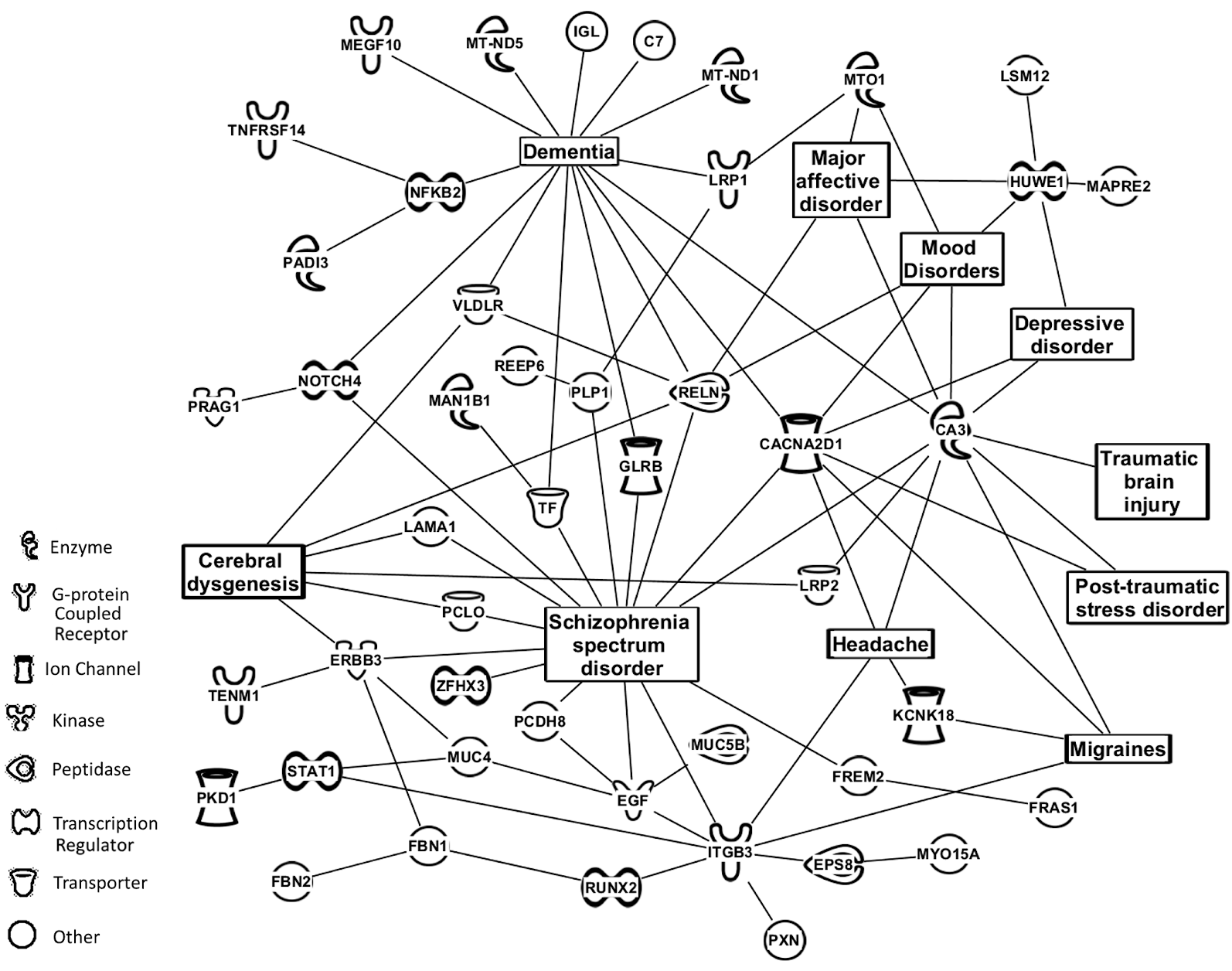
**S2 Fig. IPA analysis of TBI plus CM patients versus TBI minus CM patients.**

**S2 Fig. IPA analysis of TBI plus CM patients versus TBI minus CM patients.** Affected physiological/cellular pathways and serum protein assignments from Table 3 that were found to distinguish patients with TBI alone versus patients with TBI plus CM. The next top 58 proteins for each group (TBI alone and TBI plus CM) not exhibited in Table 3 were added to the 48 in this Table for this analysis. Analysis performed by using Ingenuity Pathway Analysis (IPA) using a “TBI” focus in the software.
