# Supplementary material for: Distinguishing and phenotype monitoring of traumatic brain injury and post-concussion syndrome including chronic migraine in serum of Iraq and Afghanistan war veterans
Source: PLoS One. 2019 Apr 26;14(4):e0215762. doi: 10.1371/journal.pone.0215762 (PMC6485717; doi:10.1371/journal.pone.0215762)
Supplement: S4 Appendix — Cell pathways incorporating serum protein changes identified from MS/MS of LOOCV mass peaks discriminating TBI “most affected”, CM, and control subject groupings using IPA software focus on “TBI”. (DOCX) [file pone.0215762.s006.docx]

**S4 Appendix. Results continued: Cell pathways incorporating serum protein changes identified from MS/MS of LOOCV mass peaks discriminating TBI “most affected”, CM, and control subject groupings using IPA software focus on “TBI”.**

S1Fig exhibits cellular/biochemical pathways that the bio-informatic software Ingenuity Pathway Analysis (IPA, QIAGEN Redwood City) indicated are being affected according to the list of 48 proteins in the TBI “most affected” versus “controls” exhibited in Table 3, top panel, plus the next 58 ranked proteins not listed in that Table. Unlike the similar Figs. in the main text (Figs. 6 and 7), S1 Fig as well as S2 Fig just have a “TBI” IPA focus rather than “neurological and immunological” focuses. Also functional information from PubMed/Medline searches of the top 106 proteins used here was not included like was included for the similar figures in the main text. Major biochemical pathways affected in this TBI “most affected” versus control comparison include typical traumatic sequelae including TBI, depression, headache, migraine. Psychological disorders are also evident with mood disorders, dementia, Alzheimer’s disease, and schizophrenia spectrum disorders. Similar pathways are observed in the IPA “alone” comparison of the top 48 proteins plus the next 58 from Table 3 (bottom panel) of the TBI minus CM versus TBI plus CM comparison (S2 Fig). The blood brain barrier (BBB), immune responses, autoimmunity, and autophagy pathways observed in the IPA analysis in Fig 6 and Fig 7 are not present in S1Fig and S2Fig. This suggests that these responses were possibly gleaned from literature searches of the proteins exhibited in Table 3 in the main text (and the next 58 top hits), and that this approach is a possible way to complement traditional IPA analysis.
