# Supplementary material for: Distinguishing and phenotype monitoring of traumatic brain injury and post-concussion syndrome including chronic migraine in serum of Iraq and Afghanistan war veterans
Source: PLoS One. 2019 Apr 26;14(4):e0215762. doi: 10.1371/journal.pone.0215762 (PMC6485717; doi:10.1371/journal.pone.0215762)
Supplement: S5 Appendix — (DOCX) [file pone.0215762.s007.docx]

**S5 Appendix. Discussion continued.**

This study deals with the long term or chronic effects of TBI in a military population at 2-14 years after injury with comparison to controls matched for age, sex, race and time since deployment. At this long term follow-up, the major symptoms found which were potentially related to TBI were headache, PTSD, and severe depression. Difficulty with dizziness, vertigo, balance problems or coordination were rarely noted to be problems for the consented TBI subjects, and essentially absent in the consented control subjects. Minor difficulty with memory was reported in a minority of TBIS but major difficulty was rare in this group. This will be the subject of a future report. To minimize bias, the TBI subjects and the matched controls were initially selected by minimal criteria. Both groups were subjected to the same study-related questionnaires. The D-TBI subjects were recruited randomly from a listing, provided by the VA VISN 19 Data Repository, of 6470 OEF/OIF Veterans who had suffered a confirmed D-TBI. These were matched to controls (by age and time of deployment ± 2 years) drawn from the same repository of 16,345 OEF/OIF veterans who did not have a D-TBI and who had experienced the same war theater conditions as the D-TBI subjects. Due to difficulty in recruiting controls, we were not able to recruit a unique control for each TBIS, however, each control subject in this study matched to at least one, if not more of the of TBIS. The criteria for recruiting a TBIS were minimal and the control subject only had to match to a TBIS by age, sex, race and time of deployment. The recruitment strategy employed provided controls matched to the TBIS with regard to their military experience, but minimized any other bias related to subject selection. In selection of subjects or controls, there was no consideration given to presence of headache, chronic migraine, PTSD or severe depression, prior to deployment. Secondly, it was necessary to identify relatively homogenous subgroups to carry out binary comparisons with the Mass Spectrometry (MS) procedure. To accomplish this, four conditions were employed which included TBI, CM, PTSD and severe depression. Employing these, four groups of Veterans were identified including “most affected” (all four conditions), least affected (none of the four), TBI+CM (no PTSD or severe depression) and TBI alone (no CM, PTSD or severe depression) were selected from the 142 recruited veterans. The 64 Veterans in these four subgroups represent 44.4% of the group who were recruited for this study.

Many of the recent studies dealing with effects of TBI in the military deal with the periods of up to 6-24 months after injury. The current report encompasses a prolonged chronic phase of 2-14 years. There has been little attention devoted to persistence of CM, PTSD and severe depression over prolonged periods after a TBI. The persistence of these symptoms suggests there is an ongoing process that is active and regenerative in producing them. These Veteran sera biomolecule analyses should provide clues about underlying mechanisms of maintaining persistence of the major symptoms mentioned. As noted, subgroups of subjects with specific combinations of the major problems of TBI, CM, PTSD, and severe depression were selected from the group of 92 TBIS and 50 CS which comprised the group of Veterans recruited into the study. The initial emphasis was on evaluating 21 D-TBI subjects with a combination of CM, PTSD and severe depression compared to 20 deployed subjects with none of the above. This showed a separation of MS profiles that was highly significant by the LOOCV analysis. The next step was to identify an additional 11 subjects with TBI+CM but no PTSD or severe depression and compare them to 12 subjects with TBI but no CM, PTSD or severe depression. Again, a significant difference in MS profiles was seen in binary comparisons of the 11 subject and 12 subject group vs the 20 control subject group. Finally, binary comparison of the TBI+CM vs the TBI without CM subgroups demonstrated a significant difference in the MS profiles.

Analysis of the MS peaks for these four binary comparisons suggested there are significant differences in the proteins involved in TBI and CM as well as PTSD and severe depression. Ingenuity Pathway Analysis of these proteins suggests pathways involving auto-immune disease, neuro-inflammation, autophagy, maintenance of blood-brain barrier, serotonin metabolism, migraine, and Alzheimer Disease/dementia could be involved. As these serum samples were obtained from subjects 2-14 years after the TBI, the implication of these results is that one or more of these pathways or mechanisms remains active sustaining pathophysiological activity over a number of years. Determining which, or what combination of these mechanisms following a TBI is important in maintaining of CM, PTSD, and severe depression for prolonged periods will be a major task for the future. A major question is whether these suggested pathophysiological pathways lead to neurodegenerative conditions or to alteration of processes that control headache, depression or PTSD. Such work will be important in defining the pathophysiology of TBI, identifying biomarkers, and providing possible therapeutic targets for these disabling conditions. A strength of the study is the strong statistical discrimination between the MS profiles of the selected groups. The study began with minimal selection criteria for the TBI subjects and control groups with subsequent subgroup selection based on four conditions found to be prominent in the TBI population. There were insufficient numbers to determine if the changes in MS profiles changed for the 4th to the 15th year after TBI. Earlier studies of the clinical course of CM, PTSD and severe depression suggested that there were no significant changes in these symptoms in cohorts 2-7 years and 8-11 years after the TBI. This suggests that the biochemical profile underlying these changes may not change to a significant degree over time.
