# Supplementary material for: Distinguishing and phenotype monitoring of traumatic brain injury and post-concussion syndrome including chronic migraine in serum of Iraq and Afghanistan war veterans
Source: PLoS One. 2019 Apr 26;14(4):e0215762. doi: 10.1371/journal.pone.0215762 (PMC6485717; doi:10.1371/journal.pone.0215762)
Supplement: S6 Appendix — Patient characteristics. (DOCX) [file pone.0215762.s008.docx]

**S6 Appendix. Materials and methods continued: patient characteristics.**

S1Table provides additional patient/subject characteristics (e.g., BMI, listed along top of Table) not provided in Table 1 of the main text (Methods and Materials). The figure and panel identification for each patient group are specified on the left side of this Table and refer to figures in the main text. S2Table lists the paygrades of all the Veteran participants in this study. E-1 through E-9 are increasing grades for enlisted personnel. S3Table details the breakdown for how Veteran study subjects group by numbers for “Years since TBI”. Pertinent Figures and respective panels are in the far left column. In general, these demographic characteristics exhibited in Table 1 and S1-S4 Tables are distributed fairly evenly among the subject groups used in the LOOCV binary comparisons, thus suggesting minimal effects of these potential confounders for Fig 3, Fig 4, and Fig 5 in these demographic tables. S4Table provides the “% of LOOCV classified serum mass peaks” scores (y axis of Fig 3, Fig 4, and Fig 5) for each patient/subject serum sample.
