# Supplementary material for: Distinguishing and phenotype monitoring of traumatic brain injury and post-concussion syndrome including chronic migraine in serum of Iraq and Afghanistan war veterans
Source: PLoS One. 2019 Apr 26;14(4):e0215762. doi: 10.1371/journal.pone.0215762 (PMC6485717; doi:10.1371/journal.pone.0215762)
Supplement: S1 Table — (DOCX) [file pone.0215762.s027.docx]

**S1 Table. Patient characteristics continued.**

| **Group characteristics and Figure identifier** | **BMI** | **Female (N)** | **DM, HLD, HTN (N)** | **Weight (kg)** | **Tobacco (N)** | **EtOH (N)** | **Patients with Headache prior to TBI (N)** | **Headaches per month mean ±SD (range)** |
| --- | --- | --- | --- | --- | --- | --- | --- | --- |
|  | **Mean ±SD** |  |  | **mean ±SD (range)** | **Smk, pSMK, pk/d, 2^nd^** |  |  |  |
| All Patients | | | | | | | | |
| All patients, N=65 | 30 ±5.44 | 2 | 11, 31, 22 | 95.26 ±18.07 (61-151) | 27, 23, 0.68, 19 | 11 | 2 | 12.31 ±11.5(0-32) |
| All Controls, N=20 | 30.03 ±5.62 | 0 | 0, 6, 5 | 95.45 ±19.48 (65-151) | 6, 6, 0.75, 4 | 3 | 0 | 0.78 ±0.9(0-4) |
| All TBI, N=45 | 29.99 ±5.42 | 2 | 11, 25, 17 | 95.18 ±17.64 (61-145) | 21, 17, 0.67, 15 | 8 | 2 | 17.43 ±10.23(1-32) |
| Fig 3 A,B | | | | | | | | |
| TBI (MA), N=21 | 29.77 ±8.51 | 1 | 5, 11, 9 | 93.52 ±18.88 (61-132) | 9, 8, 0.8, 7 | 6 | 0 | 22.86 ±6.43(12-32) |
| Controls, N=20 | 30.03 ±8.8 | 0 | 0, 6, 5 | 95.45 ±19.48 (65-151) | 6, 6, 0.75, 4 | 3 | 0 | 0.78 ±0.9(0-4) |
| RND: TBI (MA), N=21 | 29.42 ±8.74 | 0 | 3, 5, 7 | 93.29 ±17.99 (65-134) | 10, 7, 0.83, 3 | 5 | 0 | 11.88 ±11.47(0-32) |
| RND: Controls, N=20 | 30.39 ±8.81 | 1 | 2, 12, 7 | 95.7 ±20.32 (61-151) | 5, 7, 0.75, 8 | 4 | 0 | 12.3 ±12.99(0-32) |
| Fig 4 A,B,C | | | | | | | | |
| TS TBI, (MA), N=16 | 28.58 ±6.01 | 1 | 3, 8, 7 | 90.69 ±19.37 (61-132) | 8, 6, 0.83, 4 | 1 | 0 | 23.63 ±6.2(15-32) |
| TS Controls, N=15 | 30.3 ±6.25 | 0 | 0, 5, 3 | 96.67 ±22.2 (65-151) | 5, 5, 1, 3 | 1 | 0 | 0.77 ±1.02(0-4) |
| TS RND: TBI (MA), N=16 | 29.61 ±5.71 | 1 | 1, 5, 5 | 96.69 ±20.21 (65-134) | 7, 6, 0.83, 4 | 1 | 0 | 13.22 ±13.25(0-32) |
| TS RND: Controls, N=15 | 29.2 ±6.66 | 0 | 2, 8, 5 | 90.27 ±21.31 (61-151) | 6, 5, 1, 3 | 1 | 0 | 11.87 ±11.92(0-32) |
| Blinds TBI (MA), N=5 | 33.6 ±4.24 | 0 | 2, 3, 2 | 102.6 ±15.52 (82-122) | 1, 2, 0.75, 3 | 5 | 0 | 20.4 ±7.27(12-32) |
| Blinds Control, N=5 | 29.2 ±3.51 | 0 | 0, 1, 2 | 91.8 ±7.46 (84-102) | 1, 1, 0.5, 1 | 2 | 0 | 0.8 ±0.45(0-1) |
| Fig 4D | | | | | | | | |
| TS TBI (MA), N=15 | 29.97 ±6.27 | 0 | 4, 8, 6 | 92 ±17.77 (61-122) | 7, 7, 0.8, 6 | 5 | 0 | 22.8 ±6.36(12-32) |
| TS TBI, N=12 | 28.94 ±4.35 | 1 | 3, 8, 3 | 90.25 ±13.32 (70-108) | 9, 4, 0.55, 4 | 1 | 2 | 4.67 ±3.7(1-11) |
| TS RND: TBI (MA), N=15 | 30.25 ±5.52 | 0 | 5, 8, 6 | 92.33 ±16.4 (70-122) | 10, 6, 0.56, 3 | 2 | 2 | 15.13 ±10.56(1-32) |
| TS RND: TBI, N=12 | 28.59 ±5.39 | 1 | 2, 8, 3 | 89.83 ±15.32 (61-112) | 6, 5, 0.71, 7 | 4 | 0 | 14.25 ±11.05(1-32) |
| Blinds TBI (MA), N=6 | 29.27 ±5.62 | 1 | 1, 3, 3 | 97.33 ±22.75 (74-132) | 2, 1, 0, 1 | 1 | 0 | 23 ±7.21(15-30) |
| Fig 5A | | | | | | | | |
| TBI, N=12 | 28.28 ±4.17 | 1 | 3, 7, 3 | 88.92 ±12.59 (70-108) | 9, 5, 0.55, 4 | 1 | 2 | 4 ±3.36(1-11) |
| Controls, N=20 | 30.03 ±5.62 | 0 | 0, 6, 5 | 95.45 ±19.48 (65-151) | 6, 6, 0.75, 4 | 3 | 0 | 0.78 ±0.9(0-4) |
| RND: TBI, N=12 | 29.7 ±3.81 | 0 | 1, 7, 1 | 91.92 ±11.38 (70-108) | 5, 4, 0.35, 5 | 1 | 1 | 2.08 ±2.71(0-10) |
| RND: Controls, N=20 | 29.18 ±5.86 | 1 | 2, 6, 7 | 93.65 ±20.3 (65-151) | 10, 7, 0.75, 3 | 3 | 1 | 1.93 ±2.68(0-11) |
| Fig 5 B,C | | | | | | | | |
| TBI with CM, N=11 | 31.95 ±5.5 | 0 | 3, 6, 5 | 104.36 ±18.12 (79-145) | 3, 4, 0.63, 4 | 1 | 0 | 22.41 ±7.38(10-32) |
| Control, N=20 | 30.03 ±5.62 | 0 | 0, 6, 5 | 95.45 ±19.48 (65-151) | 6, 6, 0.75, 4 | 3 | 0 | 0.78 ±0.9(0-4) |
| RND: TBI with CM, N=11 | 29.82 ±6.21 | 0 | 0, 3, 1 | 96.55 ±21.5 (65-145) | 4, 3, 1, 4 | 3 | 0 | 9.18 ±11.35(0-30) |
| RND: Control, N=20 | 31.2 ±5.28 | 0 | 3, 9, 9 | 99.75 ±18.3 (83-151) | 5, 7, 0.34, 4 | 1 | 0 | 8.05 ±11.66(0-32) |
| Fig 5D | | | | | | | | |
| TBI with CM, N=11 | 31.95 ±5.5 | 0 | 3, 6, 5 | 104.36 ±18.12 (79-145) | 3, 4, 0.63, 4 | 1 | 0 | 22.41 ±7.38(10-32) |
| TBI, N=12 | 28.28 ±4.17 | 1 | 3, 7, 3 | 88.92 ±12.59 (70-108) | 9, 5, 0.55, 4 | 1 | 2 | 4 ±3.36(1-11) |
| RND: TBI with CM, N=11 | 30.12 ±5.97 | 1 | 2, 7, 3 | 95.45 ±19.99 (72-145) | 7, 5, 0.71, 5 | 2 | 1 | 12.91 ±9.57(1-30) |
| RND: TBI, N=12 | 29.97 ±4.41 | 0 | 4, 6, 5 | 97.08 ±14.73 (70-118) | 5, 4, 0.4, 3 | 0 | 1 | 12.71 ±12.43(1-32) |

MA (most affected); TS (training set); BMI (body mass index); SD (standard deviation); TBI (traumatic brain injury); DM (diabetes mellitus); HLD (hyperlipidemia); HTN (hypertension); Smk (current smoker); pSMK (former smoker); pk/d (estimated packs per day); 2nd (second hand smoke exposure); EtOH (ethanol use)
