# Supplementary material for: Distinguishing and phenotype monitoring of traumatic brain injury and post-concussion syndrome including chronic migraine in serum of Iraq and Afghanistan war veterans
Source: PLoS One. 2019 Apr 26;14(4):e0215762. doi: 10.1371/journal.pone.0215762 (PMC6485717; doi:10.1371/journal.pone.0215762)
Supplement: S2 Table — (DOCX) [file pone.0215762.s028.docx]

**S2 Table. Patient health history.**

| De-ID | approx. age at sample collection | TBI severity | PTSD | depression beck | Sex | TBI | Diabetes | HLD (High Cholesterol) | HTN (Hypertension) | Height (cm): | Weight (kg) | BMI | Smoking History | Year Quit | Current: | Packs Per Day | Second Hand Exposure: | ETOH Hx: | headache category | time since TBI | # current headaches per month | duty rank class | #TBI |
| --- | --- | --- | --- | --- | --- | --- | --- | --- | --- | --- | --- | --- | --- | --- | --- | --- | --- | --- | --- | --- | --- | --- | --- |
| control 1 | 46 | None | No PTSD | Minimal/No | Male | Control | FALSE | FALSE | FALSE | 165 | 84 | 30.9 | FALSE | 0 | FALSE | 0 | TRUE | TRUE | no CM | - | 0 | e-4 | 0 |
| control 2 | 54 | None | No PTSD | Minimal/No | Male | Control | FALSE | FALSE | FALSE | 185 | 103 | 30.1 | FALSE | 0 | FALSE | 0 | FALSE | TRUE | no CM | - | 0 | e-3 | 0 |
| control 3 | 39 | None | No PTSD | Minimal/No | Male | Control | FALSE | TRUE | FALSE | 168 | 94 | 33.3 | TRUE | 0 | TRUE | 0 | TRUE | FALSE | no CM | - | 0 | e-7 | 0 |
| control 4 | 34 | None | No PTSD | Minimal/No | Male | Control | FALSE | FALSE | FALSE | 170 | 65 | 22.5 | TRUE | 2016 | FALSE | 0 | FALSE | FALSE | no CM | - | 1 | e-6 | 0 |
| control 5 | 46 | None | No PTSD | Minimal/No | Male | Control | FALSE | TRUE | FALSE | 183 | 93 | 27.8 | TRUE | 2005 | FALSE | 0 | FALSE | FALSE | no CM | - | 1 | e-7 | 0 |
| control 6 | 51 | None | No PTSD | Minimal/No | Male | Control | FALSE | FALSE | FALSE | 170 | 75 | 26 | TRUE | 0 | TRUE | 1 | TRUE | FALSE | no CM | - | 4 | e-4 | 0 |
| control 7 | 37 | None | Probable PTSD | Minimal/No | Male | Control | FALSE | FALSE | FALSE | 175 | 83 | 27.1 | FALSE | 0 | FALSE | 0 | TRUE | FALSE | no CM | - | 1 | e-5 | 0 |
| control 8 | 34 | None | Probable PTSD | Minimal/No | Male | Control | FALSE | TRUE | FALSE | 178 | 96 | 30.3 | FALSE | 0 | FALSE | 0 | FALSE | FALSE | no CM | - | 1 | e-1 | 0 |
| control 9 | 54 | None | Probable PTSD | Minimal/No | Male | Control | FALSE | TRUE | FALSE | 173 | 83 | 27.7 | FALSE | 0 | FALSE | 0 | FALSE | FALSE | no CM | - | 1 | Officer | 0 |
| control 10 | 64 | None | Probable PTSD | Minimal/No | Male | Control | FALSE | FALSE | FALSE | 188 | 102 | 28.9 | FALSE | 0 | FALSE | 0 | FALSE | FALSE | no CM | - | 1 | e-5 | 0 |
| control 11 | 51 | None | No PTSD | Minimal/No | Male | Control | FALSE | TRUE | FALSE | 178 | 96 | 30.3 | FALSE | 0 | FALSE | 0 | FALSE | FALSE | no CM | - | 1 | e-6 | 0 |
| control 12 | 44 | None | Probable PTSD | Minimal/No | Male | Control | FALSE | FALSE | FALSE | 178 | 95 | 30 | FALSE | 0 | FALSE | 0 | FALSE | FALSE | no CM | - | 1 | e-6 | 0 |
| control 13 | 43 | None | Probable PTSD | Minimal/No | Male | Control | FALSE | FALSE | TRUE | 175 | 102 | 33.3 | TRUE | 0 | TRUE | 0.5 | FALSE | FALSE | no CM | - | 1 | e-8 | 0 |
| control 14 | 44 | None | Probable PTSD | Minimal/No | Male | Control | FALSE | FALSE | TRUE | 188 | 85 | 24 | FALSE | 0 | FALSE | 0 | FALSE | TRUE | no CM | - | 1 | e-4 | 0 |
| control 15 | 58 | None | No PTSD | Minimal/No | Male | Control | FALSE | FALSE | TRUE | 178 | 151 | 47.7 | FALSE | 0 | FALSE | 0 | FALSE | FALSE | no CM | - | 0 | e-4 | 0 |
| control 16 | 37 | None | No PTSD | Minimal/No | Male | Control | FALSE | FALSE | FALSE | 191 | 112 | 30.7 | TRUE | 0 | TRUE | 0 | FALSE | FALSE | no CM | - | 0.5 | e-4 | 0 |
| control 17 | 34 | None | No PTSD | Minimal/No | Male | Control | FALSE | FALSE | TRUE | 180 | 86 | 26.5 | TRUE | 0 | TRUE | 0 | FALSE | FALSE | no CM | - | 0 | e-4 | 0 |
| control 18 | 37 | None | Probable PTSD | Minimal/No | Male | Control | FALSE | FALSE | FALSE | 184 | 87 | 25.7 | FALSE | 0 | FALSE | 0 | FALSE | FALSE | no CM | - | 0 | e-6 | 0 |
| control 19 | 37 | None | Probable PTSD | Minimal/No | Male | Control | FALSE | TRUE | FALSE | 173 | 83 | 27.7 | FALSE | 0 | FALSE | 0 | FALSE | FALSE | no CM | - | 1 | e-5 | 0 |
| control 20 | 37 | None | Probable PTSD | Minimal/No | Male | Control | FALSE | FALSE | TRUE | 183 | 134 | 40 | TRUE | 0 | TRUE | 0 | FALSE | FALSE | no CM | - | 0 | e-5 | 0 |
| TBI 1 | 51 | Moderate: 30 Min to 6 Hours LOC | Probable PTSD | Severe | Male | TBI | TRUE | TRUE | TRUE | 168 | 79 | 28 | TRUE | 1977 | FALSE | 0 | FALSE | FALSE | CM | 9 | 18 | e-5 | 1 |
| TBI 2 | 54 | Mild: 1-30 Min LOC | Probable PTSD | Severe | Male | TBI | FALSE | FALSE | FALSE | 183 | 72 | 21.5 | TRUE | 0 | TRUE | 0 | FALSE | FALSE | CM | 13 | 20 | e-5 | 3 |
| TBI 3 | 51 | Mild: 1-30 Min LOC | Probable PTSD | Minimal/No | Male | TBI | FALSE | FALSE | FALSE | 180 | 111 | 34.3 | TRUE | 0 | TRUE | 1 | FALSE | FALSE | CM | 13 | 30 | e-4 | 1 |
| TBI 4 | 51 | Very Mild: Dazed Only | Probable PTSD | Minimal/No | Male | TBI | FALSE | TRUE | FALSE | 190 | 82 | 22.7 | TRUE | 0 | TRUE | 1 | FALSE | FALSE | no CM | 8 | 6 | e-6 | 1 |
| TBI 5 | 61 | Mild: 1-30 Min LOC | No PTSD | Severe | Male | TBI | FALSE | TRUE | FALSE | 180 | 84 | 25.9 | FALSE | 0 | FALSE | 0 | TRUE | FALSE | CM | 14 | 18 | e-5 | 1 |
| TBI 6 | 47 | Mild: 1-30 Min LOC | No PTSD | Severe | Male | TBI | FALSE | FALSE | FALSE | 180 | 122 | 37.7 | FALSE | 0 | FALSE | 0 | FALSE | TRUE | CM | 11 | 20 | e-4 | 0 |
| TBI 7 | 46 | Very Mild: Dazed Only | No PTSD | Moderate | Male | TBI | TRUE | TRUE | TRUE | 191 | 117 | 32.1 | FALSE | 0 | FALSE | 0 | FALSE | FALSE | CM | 9 | 14 | e-6 | 1 |
| TBI 8 | 36 | Mild: 1-30 Min LOC | Probable PTSD | Severe | Male | TBI | FALSE | TRUE | TRUE | 191 | 84 | 23 | FALSE | 0 | FALSE | 0 | FALSE | FALSE | CM | 12 | 18 | e-5 | 2 |
| TBI 9 | 37 | Very Mild: Dazed Only | No PTSD | Severe | Male | TBI | TRUE | TRUE | TRUE | 178 | 121 | 38.2 | TRUE | 0 | TRUE | 0 | FALSE | FALSE | CM | 14 | 26 | e-4 | 1 |
| TBI 10 | 37 | Mild: 1-30 Min LOC | Probable PTSD | Severe | Male | TBI | FALSE | TRUE | FALSE | 178 | 94 | 29.7 | TRUE | 0 | TRUE | 1 | TRUE | TRUE | CM | 13 | 12 | e-7 | 2 |
| TBI 11 | 38 | Mild: 1-30 Min LOC | No PTSD | Moderate | Male | TBI | FALSE | FALSE | FALSE | 185 | 88 | 25.7 | TRUE | 0 | TRUE | 0 | FALSE | FALSE | no CM | 13 | 2 | e-6 | 1 |
| TBI 12 | 33 | Mild: 1-30 Min LOC | Probable PTSD | Severe | Male | TBI | FALSE | TRUE | FALSE | 175 | 81 | 26.4 | TRUE | 0 | TRUE | 0.5 | TRUE | FALSE | CM | 12 | 20 | e-4 | 1 |
| TBI 13 | 49 | Mild: 1-30 Min LOC | Probable PTSD | Severe | Male | TBI | FALSE | FALSE | FALSE | 178 | 93 | 29.4 | TRUE | 0 | TRUE | 1 | FALSE | FALSE | CM | 14 | 32 | e-7 | 2 |
| TBI 14 | 49 | Very Mild: Dazed Only | No PTSD | Minimal/No | Male | TBI | FALSE | FALSE | FALSE | 180 | 91 | 28.1 | TRUE | 0 | FALSE | 0 | FALSE | FALSE | no CM | 12 | 10 | e-7 | 0 |
| TBI 15 | 44 | Very Mild: Dazed Only | No PTSD | Mild | Male | TBI | FALSE | FALSE | FALSE | 178 | 98 | 30.9 | FALSE | 0 | FALSE | 0 | FALSE | FALSE | CM | 6 | 30.5 | e-2 | 2 |
| TBI 16 | 38 | Mild: 1-30 Min LOC | Probable PTSD | Severe | Male | TBI | FALSE | FALSE | FALSE | 170 | 103 | 35.6 | FALSE | 0 | FALSE | 0 | TRUE | TRUE | CM | 14 | 32 | e-5 | 0 |
| TBI 17 | 44 | Mild: 1-30 Min LOC | No PTSD | Severe | Male | TBI | TRUE | TRUE | TRUE | 170 | 82 | 28.4 | FALSE | 0 | FALSE | 0 | FALSE | TRUE | CM | 12 | 18 | e-5 | 1 |
| TBI 18 | 34 | Very Mild: Dazed Only | Possible PTSD | Severe | Male | TBI | FALSE | FALSE | FALSE | 170 | 61 | 21.1 | TRUE | 0 | TRUE | 1 | FALSE | FALSE | CM | 13 | 32 | e-4 | 5 |
| TBI 19 | 36 | Very Mild: Dazed Only | Probable PTSD | Mild | Male | TBI | FALSE | FALSE | TRUE | 182 | 94 | 28.4 | TRUE | 0 | TRUE | 0.5 | FALSE | FALSE | CM | 7 | 32 | e-6 | 1 |
| TBI 20 | 50 | Moderate: 30 Min to 6 Hours LOC | No PTSD | Mild | Male | TBI | TRUE | TRUE | TRUE | 173 | 107 | 35.8 | TRUE | 2005 | FALSE | 0 | TRUE | FALSE | no CM | 11 | 1 | Officer | 1 |
| TBI 21 | 40 | Very Mild: Dazed Only | Possible PTSD | Minimal/No | Female | TBI | FALSE | TRUE | FALSE | 183 | 86 | 25.7 | FALSE | 0 | FALSE | 0 | TRUE | FALSE | no CM | 14 | 11 | e-4 | 1 |
| TBI 22 | 38 | Very Mild: Dazed Only | Possible PTSD | Mild | Male | TBI | FALSE | FALSE | FALSE | 165 | 72 | 26.4 | TRUE | 2016 | TRUE | 0.5 | FALSE | FALSE | no CM | 13 | 2 | e-4 | 0 |
| TBI 23 | 47 | Moderate: 30 Min to 6 Hours LOC | No PTSD | Severe | Male | TBI | FALSE | FALSE | TRUE | 163 | 107 | 40.3 | TRUE | 2015 | FALSE | 0 | FALSE | FALSE | CM | 13 | 26 | e-7 | 0 |
| TBI 24 | 34 | Mild: 1-30 Min LOC | Probable PTSD | Minimal/No | Male | TBI | FALSE | TRUE | TRUE | 190 | 93 | 25.8 | FALSE | 0 | FALSE | 0 | FALSE | FALSE | CM | 12 | 10 | e-4 | 5 |
| TBI 25 | 47 | Mild: 1-30 Min LOC | No PTSD | Mild | Male | TBI | FALSE | FALSE | FALSE | 180 | 106 | 32.7 | TRUE | 2015 | FALSE | 0 | TRUE | FALSE | no CM | 8 | 2 | e-6 | 0 |
| TBI 26 | 40 | Mild: 1-30 Min LOC | Probable PTSD | Severe | Male | TBI | TRUE | TRUE | TRUE | 175 | 112 | 36.6 | TRUE | 0 | TRUE | 0.5 | TRUE | TRUE | CM | 10 | 20 | e-7 | 2 |
| TBI 27 | 48 | Very Mild: Dazed Only | Probable PTSD | Severe | Male | TBI | FALSE | FALSE | FALSE | 175 | 85 | 27.8 | TRUE | 2017 | FALSE | 0 | TRUE | FALSE | CM | 11 | 30 | e-6 | 1 |
| TBI 28 | 45 | Mild: 1-30 Min LOC | Probable PTSD | Mild | Male | TBI | FALSE | TRUE | TRUE | 180 | 100 | 30.9 | TRUE | 2016 | FALSE | 0 | TRUE | FALSE | CM | 10 | 20 | e-5 | 1 |
| TBI 29 | 49 | Moderate: 30 Min to 6 Hours LOC | Possible PTSD | Mild | Male | TBI | FALSE | TRUE | FALSE | 178 | 86 | 27.1 | TRUE | 2016 | FALSE | 0 | FALSE | FALSE | no CM | 12 | 3 | e-4 | 1 |
| TBI 30 | 41 | Moderate: 30 Min to 6 Hours LOC | Probable PTSD | Severe | Male | TBI | TRUE | TRUE | TRUE | 196 | 132 | 34.4 | FALSE | 0 | FALSE | 0 | TRUE | FALSE | CM | 13 | 30 | e-4 | 3 |
| TBI 31 | 37 | Very Mild: Dazed Only | Probable PTSD | Mild | Male | TBI | FALSE | TRUE | FALSE | 180 | 106 | 32.7 | TRUE | 0 | TRUE | 1 | TRUE | TRUE | CM | 12 | 18 | e-4 | 1 |
| TBI 32 | 30 | Very Mild: Dazed Only | Possible PTSD | Severe | Male | TBI | FALSE | FALSE | FALSE | 188 | 74 | 20.9 | TRUE | 2015 | FALSE | 0 | FALSE | FALSE | CM | 6 | 20 | e-4 | 0 |
| TBI 33 | 29 | Mild: 1-30 Min LOC | Probable PTSD | Severe | Female | TBI | FALSE | TRUE | FALSE | 183 | 111 | 33.1 | FALSE | 0 | FALSE | 0 | FALSE | FALSE | CM | 7 | 28 | e-7 | 0 |
| TBI 34 | 43 | Very Mild: Dazed Only | Probable PTSD | Minimal/No | Male | TBI | FALSE | FALSE | FALSE | 180 | 83 | 25.6 | FALSE | 0 | FALSE | 0 | FALSE | TRUE | frequent HA | 9 | 15 | e-4 | 0 |
| TBI 35 | 49 | Mild: 1-30 Min LOC | Probable PTSD | Mild | Male | TBI | TRUE | TRUE | TRUE | 180 | 118 | 36.4 | FALSE | 0 | FALSE | 0 | FALSE | FALSE | CM | 9 | 30 | e-8 | 0 |
| TBI 36 | 37 | Very Mild: Dazed Only | No PTSD | Severe | Male | TBI | FALSE | TRUE | TRUE | 175 | 106 | 34.6 | FALSE | 0 | FALSE | 0 | FALSE | FALSE | CM | 14 | 30 | e-6 | 0 |
| TBI 37 | 30 | Very Mild: Dazed Only | Probable PTSD | Severe | Male | TBI | FALSE | FALSE | TRUE | 170 | 78 | 27 | TRUE | 0 | TRUE | 0 | FALSE | FALSE | CM | 11 | 15 | e-7 | 10 |
| TBI 38 | 30 | Very Mild: Dazed Only | Probable PTSD | Moderate | Male | TBI | FALSE | TRUE | FALSE | 176 | 104 | 33.6 | FALSE | 0 | FALSE | 0 | FALSE | FALSE | no CM | 5 | 10 | e-9 | 2 |
| TBI 39 | 56 | Very Mild: Dazed Only | Probable PTSD | Moderate | Male | TBI | TRUE | FALSE | FALSE | 168 | 87 | 30.8 | TRUE | 0 | TRUE | 0.025 | FALSE | FALSE | CM | 8 | 18 | Officer | 0 |
| TBI 40 | 37 | Mild: 1-30 Min LOC | Probable PTSD | Mild | Male | TBI | FALSE | FALSE | FALSE | 180 | 145 | 44.8 | TRUE | 2013 | FALSE | 0 | TRUE | FALSE | CM | 10 | 23 | e-6 | 30 |
| TBI 41 | 57 | Very Mild: Dazed Only | Possible PTSD | Minimal/No | Male | TBI | FALSE | TRUE | FALSE | 178 | 108 | 34.1 | TRUE | 0 | TRUE | 0.35 | FALSE | FALSE | no CM | 12 | 2 | e-6 | 2 |
| TBI 42 | 37 | Mild: 1-30 Min LOC | Possible PTSD | Mild | Male | TBI | TRUE | FALSE | TRUE | 178 | 89 | 28.1 | TRUE | 2016 | FALSE | 0 | FALSE | FALSE | no CM | 9 | 4 | e-9 | 1 |
| TBI 43 | 59 | Mild: 1-30 Min LOC | Probable PTSD | Mild | Male | TBI | FALSE | TRUE | FALSE | 180 | 79 | 24.4 | FALSE | 0 | FALSE | 0 | TRUE | FALSE | CM | 10 | 21 | e-6 | 2 |
| TBI 44 | 50 | Very Mild: Dazed Only | Possible PTSD | Mild | Male | TBI | FALSE | TRUE | FALSE | 175 | 70 | 22.9 | TRUE | 0 | TRUE | 0.35 | FALSE | FALSE | no CM | 9 | 4 | e-4 | 8 |
| TBI 45 | 37 | Mild: 1-30 Min LOC | Possible PTSD | Mild | Male | TBI | TRUE | TRUE | TRUE | 165 | 82 | 30.1 | TRUE | 1993 | FALSE | 0 | TRUE | TRUE | no CM | 13 | 1 | e-6 | 20 |
| TBI 46 | 37 | Mild: 1-30 Min LOC | Possible PTSD | severe | Male | TBI | FALSE | FALSE | FALSE | 183 | 72 | 21.5 | TRUE | 0 | TRUE | 0 | FALSE | FALSE | CM | 13 | 20 | e-5 | 2 |
| TBI 47 | 30 | Very Mild: Dazed Only | Probable PTSD | severe | Male | TBI | FALSE | TRUE | TRUE | 185 | 146 | 42.7 | TRUE | 2016 | FALSE | 0 | FALSE | FALSE | CM | 9 | 18 | e-4 | 2 |
