# Supplementary material for: Distinguishing and phenotype monitoring of traumatic brain injury and post-concussion syndrome including chronic migraine in serum of Iraq and Afghanistan war veterans
Source: PLoS One. 2019 Apr 26;14(4):e0215762. doi: 10.1371/journal.pone.0215762 (PMC6485717; doi:10.1371/journal.pone.0215762)
Supplement: S3 Table — (DOCX) [file pone.0215762.s029.docx]

**S3 Table. Years since D-TBI.**

| **Figs** | **Group Description** | **N** | **Years since TBI (N)** | | | | | | | | | | **R^2^** |
| --- | --- | --- | --- | --- | --- | --- | --- | --- | --- | --- | --- | --- | --- |
|  |  |  | **5** | **6** | **7** | **8** | **9** | **10** | **11** | **12** | **13** | **14** |  |
| 3 B,C and D | TBI (most affected) | 21 | 0 | 1 | 1 | 0 | 2 | 1 | 3 | 3 | 5 | 5 | 0.0001 |
| 4 A, B and C | TBI (most affected) | 16 | 0 | 1 | 1 | 0 | 2 | 0 | 2 | 2 | 4 | 4 | 0.0813 |
| 4D | Training set: TBI (most affected) | 15 | 0 | 0 | 0 | 0 | 1 | 1 | 2 | 3 | 4 | 4 | 0.0149 |
| 4D | Training set: TBI | 18 | 1 | 1 | 1 | 2 | 3 | 0 | 2 | 3 | 3 | 2 | 0.1454 |
| 4D | Blinded samples | 6 | 0 | 1 | 0 | 0 | 0 | 0 | 0 | 0 | 1 | 4 | 0.0522 |
| 5A | TBI | 12 | 0 | 0 | 0 | 2 | 2 | 0 | 1 | 3 | 3 | 1 | 0.0512 |
| 5 B and C | TBI + CM | 11 | 0 | 1 | 1 | 1 | 2 | 3 | 0 | 2 | 1 | 0 | 0.0139 |
| 5D | TBI +CM | 11 | 0 | 1 | 1 | 1 | 2 | 3 | 0 | 2 | 1 | 0 | 0.0003 |
| 5D | TBI | 12 | 0 | 0 | 0 | 2 | 2 | 0 | 1 | 3 | 3 | 1 | 0.0372 |
