# Supplementary material for: Distinguishing and phenotype monitoring of traumatic brain injury and post-concussion syndrome including chronic migraine in serum of Iraq and Afghanistan war veterans
Source: PLoS One. 2019 Apr 26;14(4):e0215762. doi: 10.1371/journal.pone.0215762 (PMC6485717; doi:10.1371/journal.pone.0215762)
Supplement: S4 Table — (DOCX) [file pone.0215762.s030.docx]

**S4 Table. Relationship between D-TBI number and % LOOCV Patient scores**

| group | Figure | # TBIs Mean (range) | Number of TBIs vs % LOOCV Patient Score R^2^ |
| --- | --- | --- | --- |
| 1 | 3 AB | 2.3 (1 - 11) | 0.0001 |
| 1 | 4 ABC | 2.6 (1 - 11) | 0.0813 |
| blinds | 4 ABC blinds | 1.4 (1 - 2) | 0.749 |
| 1 | 4d | 1.8 (1 - 5) | 0.052 |
| blinds | 4d blinds | 3.5 (1 - 11) | 0.0522 |
| 2 | 4d | 4.2 (1 - 29) | 0.1454 |
| 1 | 5A | 4.1 (1 - 29) | 0.0512 |
| 1 | 5BC | 4.2 (1 - 30) | 0.0139 |
| 1 | 5D TBI+CM | 4.2 (1 - 30) | 0.0003 |
| 2 | 5D TBI | 4.1 (1 - 29) | 0.0372 |
