# Supplementary material for: Distinguishing and phenotype monitoring of traumatic brain injury and post-concussion syndrome including chronic migraine in serum of Iraq and Afghanistan war veterans
Source: PLoS One. 2019 Apr 26;14(4):e0215762. doi: 10.1371/journal.pone.0215762 (PMC6485717; doi:10.1371/journal.pone.0215762)
Supplement: S5 Table — (DOCX) [file pone.0215762.s031.docx]

**S5 Table. Volunteer specifications by pay grade.**

| **Group characteristics and Figure identifier** | **Pay Grade** | |
| --- | --- | --- |
|  | **(N)** | **(%)** |
|  | **E-1-E9,Officer** | **E-1 to E-4 : E-5 to E-9 : Officer** |
| All Samples | | |
| All patients, N=65 | 1, 1, 1, 20, 11, 15, 9, 2, 2, 3 | 35.4% : 60% : 4.6% |
| All Controls, N=20 | 1, 0, 1, 6, 4, 4, 2, 1, 0, 1 | 40% : 55% : 5% |
| All TBI, N=45 | 0, 1, 0, 14, 7, 11, 7, 1, 2, 2 | 33.3% : 62.2% : 4.4% |
| Fig 3 A and B | | |
| TBI (MA), N=21 | 0, 0, 0, 7, 6, 2, 6, 0, 0, 0 | 33.3% : 66.7% : 0% |
| Controls, N=20 | 1, 0, 1, 6, 4, 4, 2, 1, 0, 1 | 40% : 55% : 5% |
| RND: TBI (MA), N=21 | 1, 0, 1, 8, 4, 4, 3, 0, 0, 0 | 47.6% : 52.4% : 0% |
| RND: Controls, N=20 | 0, 0, 0, 5, 6, 2, 5, 1, 0, 1 | 25% : 70% : 5% |
| Fig 4 A,B and C | | |
| TS TBI, (MA), N=16 | 0, 0, 0, 6, 4, 2, 4, 0, 0, 0 | 37.5% : 62.5% : 0% |
| TS Controls, N=15 | 1, 0, 1, 4, 4, 3, 1, 0, 0, 1 | 40% : 53.3% : 6.7% |
| TS RND: TBI (MA), N=16 | 0, 0, 1, 4, 5, 3, 3, 0, 0, 0 | 31.3% : 68.8% : 0% |
| TS RND: Controls, N=15 | 1, 0, 0, 6, 3, 2, 2, 0, 0, 1 | 46.7% : 46.7% : 6.7% |
| Blinds TBI (MA), N=5 | 0, 0, 0, 1, 2, 0, 2, 0, 0, 0 | 20% : 80% : 0% |
| Blinds Control, N=5 | 0, 0, 0, 2, 0, 1, 1, 1, 0, 0 | 40% : 60% : 0% |
| Fig 4D | | |
| TS TBI (MA), N=15 | 0, 0, 0, 4, 6, 1, 4, 0, 0, 0 | 26.7% : 73.3% : 0% |
| TS TBI, N=12 | 0, 0, 0, 4, 0, 4, 1, 0, 2, 1 | 33.3% : 58.3% : 8.3% |
| TS RND: TBI (MA), N=15 | 0, 0, 0, 5, 2, 2, 3, 0, 2, 1 | 33.3% : 60% : 6.7% |
| TS RND: TBI, N=12 | 0, 0, 0, 3, 4, 3, 2, 0, 0, 0 | 25% : 75% : 0% |
| Blinds TBI (MA), N=6 | 0, 0, 0, 3, 0, 1, 2, 0, 0, 0 | 50% : 50% : 0% |
| Fig 5A | | |
| TBI, N=12 | 0, 0, 0, 4, 0, 5, 1, 0, 1, 1 | 33.3% : 58.3% : 8.3% |
| Controls, N=20 | 1, 0, 1, 6, 4, 4, 2, 1, 0, 1 | 40% : 55% : 5% |
| RND: TBI, N=12 | 0, 0, 0, 2, 1, 4, 3, 0, 0, 2 | 16.7% : 66.7% : 16.7% |
| RND: Controls, N=20 | 1, 0, 1, 8, 3, 5, 0, 1, 1, 0 | 50% : 50% : 0% |
| Fig 5 B,C | | |
| TBI with CM, N=11 | 0, 1, 0, 3, 1, 4, 0, 1, 0, 1 | 36.4% : 54.5% : 9.1% |
| Control, N=20 | 1, 0, 1, 6, 4, 4, 2, 1, 0, 1 | 40% : 55% : 5% |
| RND: TBI with CM, N=11 | 1, 0, 1, 4, 1, 4, 0, 0, 0, 0 | 54.5% : 45.5% : 0% |
| RND: Control, N=20 | 0, 1, 0, 5, 4, 4, 2, 2, 0, 2 | 30% : 60% : 10% |
| Fig 5D | | |
| TBI with CM, N=11 | 0, 1, 0, 3, 1, 4, 0, 1, 0, 1 | 36.4% : 54.5% : 9.1% |
| TBI, N=12 | 0, 0, 0, 4, 0, 5, 1, 0, 1, 1 | 33.3% : 58.3% : 8.3% |
| RND: TBI with CM, N=11 | 0, 0, 0, 6, 1, 3, 0, 0, 0, 1 | 54.5% : 36.4% : 9.1% |
| RND: TBI, N=12 | 0, 1, 0, 1, 0, 6, 1, 1, 1, 1 | 16.7% : 75% : 8.3% |
