# Supplementary material for: Distinguishing and phenotype monitoring of traumatic brain injury and post-concussion syndrome including chronic migraine in serum of Iraq and Afghanistan war veterans
Source: PLoS One. 2019 Apr 26;14(4):e0215762. doi: 10.1371/journal.pone.0215762 (PMC6485717; doi:10.1371/journal.pone.0215762)
Supplement: S6 Table — (DOCX) [file pone.0215762.s032.docx]

**S6 Table. % Patient LOOCV score vs age.**

| **Group** | **Figure panel** | **Patient age vs % LOOCV patient score R^2^** |
| --- | --- | --- |
| TBI (most affected) | 3A | 0.1182 |
| Controls | 3A | 0.0419 |
| Training set: TBI (most affected) | 4A | 0.0071 |
| Training set: Controls | 4A | 0.0973 |
| Blinds: TBI (most affected) | 4C | 0.051 |
| Blinds: Controls | 4C | 0.0133 |
| Training set: TBI (most affected) | 4D | 0.0311 |
| Training set: TBI | 4D | 0.1519 |
| Blinds: TBI (most affected) | 4D | 0.0014 |
| TBI (most affected) | 5A | 0.0789 |
| Controls | 5A | 0.1671 |
| TBI+CM | 5B | 0.0073 |
| Controls | 5B | 0.0338 |
| TBI+CM | 5D | 0.1988 |
| TBI | 5D | 0.1372 |
