# Supplementary material for: Distinguishing and phenotype monitoring of traumatic brain injury and post-concussion syndrome including chronic migraine in serum of Iraq and Afghanistan war veterans
Source: PLoS One. 2019 Apr 26;14(4):e0215762. doi: 10.1371/journal.pone.0215762 (PMC6485717; doi:10.1371/journal.pone.0215762)
Supplement: S7 Table — (DOCX) [file pone.0215762.s033.docx]

**S7 Table. Normalized peak area mean for each patient by m/Z used for figures and tables.**

De-ID control 1 control 2 control 3 control 4 control 5 control 6 control 7 control 8 control 9 control 10 control 11 control 12 control 13 control 14 control 15 control 16 control 17 control 18 control 19 control 20 TBI 1 TBI 2 TBI 3 TBI 4 TBI 5 TBI 6 TBI 7 TBI 8 TBI 9 TBI 10 TBI 11 TBI 12 TBI 13 TBI 14 TBI 15 TBI 16 TBI 17 TBI 18 TBI 19 TBI 20 TBI 21 TBI 22 TBI 23 TBI 24 TBI 25 TBI 26 TBI 27 TBI 28 TBI 29 TBI 30 TBI 31 TBI 32 TBI 33 TBI 34 TBI 35 TBI 36 TBI 37 TBI 38 TBI 39 TBI 40 TBI 41 TBI 42 TBI 43 TBI 44 TBI 45 TBI 46 TBI 47

150 0 0 0 0 0 0 0 0 0 0 0 0 0 0 0 0 0 0 0 0 0 0 0 0 0 0 0 0 0 0 0 0 0 0 0 0 0 0 0 0 0 0 0 0 0 0 0 0 0 0 0 0 0 0 0 0 0 0 0 0 0 0 0 0 0 0 0

151 0 0 0 0 0 0 0 0 0 0 23.55 0 0 0 0 0 0 0 0 0 47.29658272 0 0 0 0 0 0 0 0 0 0 0 0 0 0 14.63284853 0 0 0 0 11.5206465 0 0 0 0 26.06919443 0 0 0 0 0 0 14.89115418 19.07249467 0 0 0 0 0 0 0 0 0 0 0 0 0

152 16.49852507 0 8.76242954 0 0 11.18051783 0 0 0 0 0 11.12227806 16.40762787 0 15.15799477 31.03558568 0 0 63.43930422 14.91124683 0 0 15.54639175 0 0 17.06 0 0 0 23.16807238 10.33082434 0 30.88185568 20.39670533 19.78423843 0 0 0 15.61813043 42.00521253 0 13.07573849 19.97799935 14.54250928 0 0 0 0 16.50623886 25.45924248 12.35836988 47.34972004 16.84939209 13.71174691 15.51111111 17.81808113 39.72992526 0 0 25.46758105 12.25333333 8.493333333 0 0 0 8.474576271 0

153 0 22.47661902 0 0 21.74170178 0 35.64384242 22.54185864 23.00242015 24.3367597 0 31.32682032 19.48056886 11.24609155 16.68981234 0 16.67067019 56.91556083 0 0 23.97 17.07278959 0 22.76311104 0 0 44.13121765 0 13.69123932 18.23723465 0 0 15.17006763 6.622369878 11.89502835 16.62615213 37.138075 33.33333333 0 0 0 0 20.29339853 42.61746111 20.90625424 0 50.69503546 29.92944755 0 15.73944211 0 6.96241528 13.12643678 27.15827338 20.24513722 0 0 33.33333333 33.33333333 0 26.74 0 14.35333333 34.59702468 14.24327307 25.82929996 34.72688754

154 0 0 0 0 0 23.72855215 0 23.53567625 0 0 13.79808323 0 26.70540383 0 0 0 29.71037211 0 0 0 0 0 18.8963652 0 33.33333333 0 0 10.76143691 33.33333333 0 0 33.33333333 0 0 0 0 0 0 13.24317739 0 14.48930392 11.09990757 0 0 50.22609819 56.05 22.12466781 33.33333333 12.71922452 0 0 0 0 0 22.3628692 0 18.47636164 41.33478378 33.33333333 30.79867236 0 0 0 22.14388439 0 0 0

155 66.58827527 0 60.83119841 26.65779552 33.33333333 34.33277546 0 0 0 0 0 0 0 51.34130146 0 52.91353383 16.66266314 21.24961809 0 33.33333333 0 18.22545022 0 0 66.66666667 66.66666667 33.46420123 0 33.33333333 0 13.98491575 33.33333333 0 41.46179402 33.33333333 34.45936147 0 0 33.33333333 0 45.75079662 33.33333333 0 0 0 0 0 0 16.82709447 0 44.17063213 0 0 16.01746524 0 12.17109365 5.789451097 0 33.33333333 0 9.63 35.18333333 24.52333333 0 0 0 0

156 0 33.33333333 0 33.33333333 0 0 64.35615758 21.71570984 24.17714975 33.33333333 0 0 0 22.08724179 33.33333333 6.290209919 0 9.904456709 0 18.4220865 0 15.3178885 40.50119261 0 0 0 0 50.1365882 19.64209402 16.05132978 0 0 0 0 0 0 13.35817968 33.33333333 11.52866464 0 0 8.497830043 31.12877778 42.84002962 0 0 0 25.29416605 0 34.51115357 20.97496345 14.20004108 18.44217916 0 10.83703704 48.84858554 8.364018937 0 0 7.865752286 5.153333333 0 11.50666667 24.87590147 52.4233936 25.88727495 33.33333333

157 0 33.33333333 0 0 44.92496489 9.604781184 0 0 25.29731909 6.607765478 43.11666667 0 13.85276447 15.32536521 34.81885955 0 17.2698199 0 19.18310759 33.33333333 19.30450592 33.33333333 14.43696814 33.33333333 0 0 0 0 0 0 0 6.963329213 51.49659904 0 34.98739988 8.680239036 33.33333333 33.33333333 0 50.38422332 0 0 0 0 28.86764757 0 15.97163121 0 33.33333333 17.34742912 11.88241899 10.09584023 16.48394125 0 13.08819611 21.16223968 7.74644865 0 0 35.8679943 0 27.19666667 0 7.193740512 33.33333333 14.95009176 31.93977913

158 12.20058997 0 24.57090379 0 0 0 0 0 27.52311101 0 19.5352501 43.84500332 23.55363497 0 0 0 19.68647466 0 0 0 0 0 0 0 0 16.27333333 0 22.57189643 0 15.06233178 0 0 0 0 0 0 16.17041199 0 20.09015595 0 18.84402942 22.23342576 28.59982434 0 0 10.61666667 0 11.44305307 20.61410881 0 0 0 0 14.26083866 0 0 0 0 0 0 0 0 0 0 0 24.85875706 0

159 4.712609682 10.85671431 5.835468261 40.00887115 0 21.15337337 0 32.20675527 0 35.72214148 0 13.7058983 0 0 0 9.760670564 0 11.93036438 17.3775882 0 9.428911362 16.05053835 10.6190823 43.90355562 0 0 22.40458113 16.53007847 0 27.48103141 75.68425991 26.37000412 2.451477649 31.51913077 0 25.60139883 0 0 6.18653826 7.610564151 9.395223547 11.7597648 0 0 0 7.2641389 11.20866552 0 0 6.942732725 10.61361555 21.39198337 20.20689655 9.779181136 17.95564932 0 19.89379442 25.33188289 0 0 10.34333333 0 9.816666667 11.18944895 0 0 0

160 0 0 0 0 0 1.748654283 0 0 0 0 0 0 0 0 0 0 17.5164967 0 0 0 20.45362 0 0 20.43333333 0 0 0 0 11.43964452 4.537729436 0 0 0 0 0 9.440478263 0 0 0 0 0 0 0 6.714060032 0 0 0 16.05302544 0 0 0 0 0 0 0 0 0 0 0 13.65593354 7.256666667 0 0 0 7.18116111 0 0

161 7.1859401 27.8902659 0 33.33333333 17.5205829 25.38146761 0 7.285873192 0 0 0 0 0 0 9.470359572 0 0 10.19026497 23.24796829 0 0 6.296666667 0 0 0 34.56957995 0 0 0 19.19907054 0 0 0 14.53881894 24.52241961 5.200875099 13.38534279 0 0 0 0 0 0 0 8.578840096 13.76779686 0 0 6.652135303 0 0 0 0 0 0 0 5.549060868 0 0 0 0 0 2.023333333 10.20416513 16.37974052 0 0

162 0 22.82663031 14.70102783 0 0 0 0 0 9.516664393 12.6781923 33.33333333 43.85489235 0 15.18205462 0 0 0 13.60133123 0 35.09862619 0 3.011648259 10.92657619 0 0 0 10.10402244 0 0 0 33.33333333 0 0 0 18.43902439 0 0 0 8.706346895 0 0 24.1945918 33.33333333 0 0 22.68097096 13.59207794 13.34026334 4.682293462 0 9.85051871 0 9.662034654 0 0 0 0 27.91002689 0 0 0 0 0 0 0 33.33333333 0

163 12.57626179 11.96253725 10.0811124 16.08648649 0 0 28.01332999 14.11820228 10.00773885 7.897378695 17.15863052 10.26895838 39.44662549 0 0 51.75200723 11.35851339 0 0 12.11386662 26.55629774 18.04018888 49.60052722 24.5007982 33.33333333 14.32618262 16.79859279 26.0520755 33.33333333 7.774500164 9.444955535 66.66666667 0 0 0 0 41.71610635 23.04952942 12.20274083 31.66213188 35.64398667 27.82791971 0 37.80125155 48.96859808 10.15705269 50.84756483 5.80714133 9.153945088 55.68524547 0 12.34507766 6.696502569 15.26349206 7.86687631 10.7946417 33.33333333 22.23184316 36.42450167 43.85224368 14.1 29.21 9.956666667 31.56477844 0 9.698727243 60.12526536

164 16.22921186 0 0 33.33333333 0 48.89669879 0 26.04746014 15.09736786 0 0 0 0 57.01074544 19.27076755 0 27.64976959 0 0 0 0 0 0 0 31.88839063 0 0 33.33333333 49.47713073 0 16.29241517 27.6524268 33.33333333 9.893541519 0 39.69922717 0 0 0 0 0 0 0 0 0 0 0 33.33333333 0 0 33.33333333 0 0 22.42047581 0 0 0 0 0 0 5.75 0 0 0 33.33333333 23.27666667 0

165 9.343596322 0 33.33333333 0 56.74457122 0 0 0 0 53.98847437 20.45319686 15.04734622 45.70664414 0 6.049562682 33.33333333 0 25.70089325 60.848839 0 0 0 7.797918446 33.33333333 0 18.08737829 8.430808565 29.33652313 0 0 0 0 0 0 0 0 11.02504944 46.78519616 0 33.33333333 33.33333333 0 66.66666667 33.33333333 42.45256182 0 0 0 0 0 0 33.33333333 42.37776634 19.94288434 11.90036088 66.66666667 0 7.588723051 0 0 0 0 0 0 0 12.30223941 23.56374808

166 0 18.92502281 18.6323055 0 0 0 55.64761905 21.24130402 18.01744759 25.43595464 0 0 0 0 33.33333333 0 21.71050863 42.87507047 15.90319271 21.21946672 0 57.35835174 0 0 0 10.22552255 39.76405144 0 0 28.7956039 23.8883778 5.680906538 33.33333333 33.33333333 14.89430894 0 19.94799054 0 33.33333333 27.43354505 15.10351186 27.15398442 0 0 0 33.33333333 15.81910183 31.46623655 28.65103987 0 0 0 26.63683076 18.06984127 20.46864232 0 33.33333333 29.13507855 11.37443573 0 16.73333333 14.19 0 16.00548902 26.15217222 0 0

167 30.67525292 10.50670302 0 0 0 7.951865723 16.33905096 0 23.81666894 0 29.0548393 2.504838893 11.72173037 0 14.06256579 14.91465944 5.683563748 0 0 31.56804047 45.18 15.29314445 7.383627608 12.9 34.77827603 0 0 0 0 7.857533049 17.04091816 0 33.33333333 26.1188579 0 6.099177937 0 10.28380392 0 7.570989736 15.91916814 20.82350407 0 22.15135508 0 0 19.74125539 0 24.17938825 11.12180001 56.81614796 54.321589 0 24.30330652 21.43297245 5.592404734 27.78427247 0 45.03784093 33.33333333 0 10.60666667 9.196666667 42.22556741 0 0 5.597470719

168 0 0 23.25222093 17.24684685 0 0 0 0 18.23596548 0 0 20.30693542 0 0 0 0 0 7.632440081 0 0 7.810082254 0 0 0 0 14.00970843 0 7.281257835 5.749891417 14.13426279 0 0 0 16.11544831 0 25.51224365 13.92551088 0 0 0 0 0 0 0 0 20.06084616 0 0 0 25.09076864 0 0 0 0 25.46645702 0 0 13.13432836 0 9.158489444 9.456666667 0 21.93333333 0 16.95359281 10.05666667 0

169 23.98973701 7.888840705 0 0 25.73484588 16.0213136 0 31.30716036 5.308146897 0 0 8.017028726 3.125 27.80719994 17.81341108 0 16.08114795 0 0 0 0 0 24.29135054 8.832535134 0 8.781628163 24.90252477 3.996810207 0 17.70130012 0 0 0 0 42.14424706 14.04799788 0 19.88147051 45.75757894 0 0 0 0 0 0 0 0 0 26.68119803 8.10218588 0 0 14.62686567 0 12.86469102 16.9462869 0 0 7.163221668 0 0 0 27.55333333 0 0 11.33236668 10.71351584

170 17.64255645 26.38736494 0 0 0 0 15.55628607 0 0 4.717898833 36.29932168 11.77534412 21.99104916 19.08973467 0 27.1702417 22.21600656 0 12.89494471 25.72486742 12.965098 6.762219772 33.33333333 0 0 0 0 19.18760021 24.26444067 0 0 16.60397281 2.600911458 0 0 22.04063986 6.709015937 13.63901169 33.33333333 13.6089318 25.51054018 0 42.01281391 12.01787995 33.33333333 0 22.02651106 0 0 18.56289505 0 0 15.04764832 16.0619469 0 0 10.87204236 33.43009335 0 18.53386809 15.23666667 32.20666667 0 0 17.44415649 17.29828042 28.97604298

171 0 0 0 17.99242341 0 0 12.55928854 14.43486486 27.13191247 12.26331796 0 0 0 0 33.33333333 14.48084554 15.33514002 22.34313598 0 18.5721177 4.109417937 7.090923618 0 13.09 12.26471024 0 0 6.420180621 9.132103389 18.11983009 0 12.21659634 11.92357153 0 0 0 35.91734159 0 0 19.65934253 25.46434573 22.34689639 0 0 21.06561421 15.84471069 38.07189808 47.68142119 19.882276 14.02575588 16.42231889 0 0 38.54835246 25.72100609 38.118426 44.26845805 9.599776099 37.22443498 0 24.66333333 6.266666667 8.086666667 42.37610958 8.65248227 0 0

172 1.385041551 0 0 0 11.29146855 19.49910253 33.33333333 0 0 0 0 0 9.498376793 0 0 17.40357479 0 30.26433549 11.52107642 0 17.48061543 7.453333333 0 0 30.55284235 6.978382424 15.42538855 0 3.064010517 22.01188843 14.10333333 0 0 32.25467313 14.88477073 0 0 21.57533027 0 0 0 12.71596628 0 0 0 23.7807377 0 0 0 0 0 58.83290498 14.01321398 0 0 0 0 0 0 26.75242171 0 0 0 0 0 14.63087667 0

173 12.03666667 0 0 24.92256148 12.58867652 13.23014205 0 0 0 0 15.62665537 15.90443457 21.20533493 13.53722583 43.58831193 0 0 0 23.80788076 11.02577684 0 0 9.787729607 8.790837243 0 26.64774519 9.073333333 15.17967088 0 0 17.58759175 8.026420445 0 0 11.90604509 43.60812425 8.183810691 0 33.33333333 19.95706715 0 21.3387185 0 55.24810274 30.31616675 0 5.592706904 0 0 33.33333333 40.87699504 0 34.75301883 0 8.234443452 0 0 18.45925437 21.17746005 0 0 7.76 0 18.30217006 30.88795234 0 14.99743742

174 0 13.40435681 20.98988681 14.57936428 11.1238972 0 7.243041858 0 33.33333333 0 18.47166512 0 0 0 0 6.163091635 33.33333333 0 0 0 6.520109918 0 0 53.57666667 14.66207203 26.35495091 0 0 13.10399038 15.21350324 0 0 25.93411403 16.45842467 0 0 2.727088402 4.836300513 0 0 17.56573164 0 27.07296849 0 0 0 0 0 0 0 0 0 0 0 0 0 13.84811314 0 0 4.180035651 0 0 0 0 0 31.11898292 0

175 17.11720317 0 22.42313731 9.812972271 0 0 0 0 0 0 5.502422851 21.55798922 0 0 23.07835474 4.863232557 14.35851918 33.35036412 8.471563981 11.05225989 20.36823534 26.57111356 15.60569926 0 36.11382431 0 0 6.306787814 0 0 11.15666667 0 19.91209945 20.32725821 7.606715768 4.120190229 14.53800371 0 0 5.887480722 7.822793149 3.820880213 15.73999732 0 0 33.44597086 11.30682228 19.13824233 5.694353608 6.734041814 16.91101445 0 9.287084399 29.5214669 6.998097747 0 18.15155358 5.004218285 0 0 9.106666667 0 0 0 0 0 33.33333333

176 0 12.24074074 0 18.75396905 35.32360786 0 20.7740448 41.85975556 16.80317269 61.94876783 0 0 0 0 0 0 0 0 14.19238254 0 38.55652338 0 0 0 0 0 33.33333333 0 39.33821548 33.33333333 49.07907492 0 0 0 0 0 0 19.69432165 0 0 17.41505969 0 15.17422027 6.251596424 0 0 0 33.18033649 57.66386675 0 0 0 0 0 5.388966867 13.73911292 4.246655032 0 18.36364578 18.79453392 0 12.16333333 0 0 0 0 0

177 0 37.96383381 36.56825272 13.93870951 0 52.19675878 0 33.33333333 0 0 24.09993497 0 35.17724071 53.12944084 0 0 0 10.99019735 0 6.402039832 0 25.88 23.54560373 24.54249609 6.40655106 33.33333333 0 18.15366245 11.09723956 0 0 63.1530104 14.27470075 30.95964399 65.60246841 0 18.44050671 11.75800306 33.33333333 13.6739908 0 22.98105178 0 0 0 0 23.00206168 0 7.756703728 19.30757745 18.43963102 29.92207477 26.89903446 15.86823373 0 48.14246108 0 0 11.0785859 0 23.05333333 15.63666667 17.88 31.2770867 15.88917685 36.95185999 5.229619864

178 36.98744355 0 10.91019602 0 20.74465682 0 10.53400541 0 13.58998884 0 0 0 12.12799841 14.24359866 0 13.98925524 14.7570009 3.051967055 29.11215159 18.86376252 0 26.24240972 0 0 0 0 42.16794478 34.75209802 0 0 8.073333333 0 25.35460279 0 0 30.23104566 0 28.49703282 0 13.37626618 6.221529609 16.79648684 0 15.06385696 15.2848857 17.3588555 0 0 0 0 0 11.24502025 0 0 0 0 8.613177827 33.5066579 12.15587328 21.11971104 0 0 15.16666667 8.044633664 27.12623206 0 0

179 14.83108861 10.0037037 9.108527132 0 8.927693064 15.07399664 0 10.37204625 9.141592666 21.07001537 0 50.7622321 0 0 0 15.92975854 0 0 0 8.359175798 0 0 17.72763407 0 0 6.685588143 0 0 0 11.3214449 0 0 0 0 0 0 13.48423297 0 0 13.83692081 0 0 0 11.41856392 0 9.569725246 0 0 9.002799914 8.036396471 7.350040608 0 0 0 53.65748584 0 0 0 0 10.61942959 6.486666667 0 0 0 0 0 17.4635664

180 0 0 0 0 11.29844719 0 0 6.843775058 0 0 5.384172051 0 12.93025163 0 0 0 0 0 0 0 0 11.32800609 0 8.116773279 28.40419948 0 7.033977975 0 0 0 0 0 0 0 0 0 0 0 7.030688311 0 0 11.13884926 0 0 13.70025402 0 7.200180852 0 0 0 0 0 0 0 0 6.575813707 0 16.79117293 0 0 0 0 0 0 0 8.460634548 0

181 0 9.664625255 0 0 0 0 13.18652355 0 0 0 5.48413813 0 0 0 10.41944075 8.155186065 36.30429866 24.64905213 0 0 1.96789424 4.045488441 0 0 0 0 0 0 35.08462406 0 15.38179515 0 0 0 10.3343893 0 0 8.37332602 22.11503207 8.259671134 5.559185451 0 13.93289329 11.21094548 0 5.277182965 0 14.11966944 7.676809782 0 9.833971903 0 8.044935677 0 0 0 4.453055952 0 3.449167442 0 4.14 0 0 0 0 16.06684982 0

182 0 0 0 19.34435327 0 0 0 0 0 0 0 0 0 0 19.75600157 0 0 0 0 0 0 0 0 0 0 0 0 31.32547227 0 0 0 0 0 0 0 15.14902926 0 0 0 18.68045477 0 0 0 0 0 0 16.05770739 0 0 0 16.39309316 0 0 19.74086823 0 0 0 0 0 0 0 0 17.57333333 0 0 0 0

183 24.24373848 51.94744471 92.80087746 14.36934335 48.47202466 17.99797776 52.69671645 64.34748602 30.71992722 50.72163933 49.85605054 62.5189414 33.33333333 80.2965388 11.11850866 33.33333333 16.6322314 39.39637453 33.33333333 79.51368189 75.35527379 0 32.93222381 48.61253159 28.11075418 62.31503851 34.06283311 11.14266243 11.8649755 37.41581175 13.86194714 0 51.20457783 60.56076078 0 50.6006006 66.66666667 91.62667398 25.9217698 25.7106104 66.66666667 55.5278174 0 55.25325506 29.56049947 0 0 61.6771143 86.12693884 56.04483518 56.83269476 33.48449142 0 61.80511962 84.35377374 77.60531285 54.76457743 40.47645646 66.66666667 27.13334366 50.84 49.57666667 29.97 23.29336155 60.72633354 0 51.76431286

184 49.96434713 17.50947798 0 0 0 33.33333333 0 0 50.05720371 30.02856841 26.79292929 33.33333333 13.86765094 0 25.76897059 58.5114806 33.33333333 21.33901112 59.22253259 0 0 62.62117823 22.66635681 0 0 0 33.33333333 4.75116766 14.01004079 33.33333333 52.88683486 78.98876568 33.33333333 16.51031895 75.79544121 0 33.33333333 0 39.14874238 0 27.77414788 33.33333333 86.06710671 22.12238786 0 94.72281704 33.33333333 0 0 28.4699151 0 22.89319249 84.66509633 0 0 0 0 33.33333333 29.88416589 66.66666667 0 19.10666667 18.75666667 62.97929192 33.33333333 50.59981685 20.37898712

185 0 0 0 33.33333333 22.03488614 33.33333333 0 0 0 0 0 0 33.33333333 0 0 0 5.134281201 0 0 0 0 22.00532725 8.200847907 0 38.67139805 0 0 0 0 0 0 0 0 0 0 0 0 0 0 17.52685106 0 0 0 0 48.46941868 0 43.40877843 0 0 0 16.94024018 15.91629148 0 13.5924651 0 0 28.88027738 0 0 0 0 0 0 0 0 24.87269879 0

186 16.70231953 14.71922196 0 28.57399835 11.76797534 15.33535558 26.4399093 14.56849837 0 15.94502734 0 0 0 0 17.71824895 0 0 14.61556223 0 0 0 0 0 43.27069513 0 37.68496149 19.82180294 20.05663936 5.99371503 0 7.785292848 0 15.46208884 0 0 34.25037014 0 0 0 14.65287857 0 0 0 0 0 0 0 0 0 0 0 4.504667269 0 0 0 0 5.974426808 0 0 0 0 0 0 0 0 0 0

187 0 0 7.199122545 0 0 0 7.67685071 11.38140431 4.682220947 0 6.54040404 0 0 7.944394259 7.564362739 0 0 0 0 0 0 0 25.8203383 0 0 0 0 0 33.04664462 8.855047941 4.089591041 0 0 6.105905885 0 0 0 0 0 0 0 0 0 0 8.26982783 0 0 13.26355711 6.196251378 4.332968835 0 0 7.289967989 4.861547046 5.054589567 15.81887345 0 9.399037269 0 6.199989675 0 5.846666667 0 0 5.940333122 0 27.85670002

188 0 6.159230096 0 0 6.426666667 0 0 2.858836244 7.324704878 0 0 4.14772527 6.535430764 11.75906694 0 0 8.595855404 0 0 0 8.829806475 0 0 0 0 0 0 18.57518656 0 0 5.994538962 10.19613234 0 16.82301438 0 0 0 0 5.78376744 15.16953407 0 0 0 0 0 0 0 10.93965914 0 11.15228089 0 10.44014085 0 0 8.435656195 0 5.927662425 0 0 0 0 0 2.423333333 10.03997178 0 0 0

189 9.089594853 0 0 4.378971693 0 0 0 0 7.215943244 3.304764928 5.942305942 0 0 0 7.654466745 0 0 0 7.444134078 20.48631811 13.8470255 0 10.38023317 0 4.813648294 0 5.748052646 14.14887172 0 20.39580698 0 10.81510197 0 0 13.87016949 0 0 0 0 0 0 0 0 11.41341161 0 0 0 0 0 0 0 12.7612165 0 0 2.155980502 0 0 0 0 0 3.916666667 0 0 3.687374749 0 0 0

190 0 0 0 33.33333333 8.171993444 0 10.05941198 0 0 0 0 0 0 0 2.21936395 3.192956069 0 36.7857834 0 10.58993201 0 9.862029655 8.390063299 11.43207417 0 39.83357556 0 0 0 7.178191769 6.907886958 0 9.930554023 4.197441175 0 16.90735821 0 13.6311849 0 0 13.76736111 9.238971662 9.5772471 0 8.043619629 12.5247836 8.078999485 0 5.782754517 0 0 0 0 10.63857123 5.84257975 11.94292415 9.693553711 5.69228491 3.935343843 5.330838679 0 6.256666667 0 0 3.620706527 0 0

191 11.66470376 24.96492853 11.86441309 11.31990502 0 2.740775669 7.201463428 0 6.705922954 0 7.562364958 48.73009512 7.831810094 8.183573742 0 3.001212611 3.233696352 0 9.078208049 0 24.01460404 0 0 0.388784166 15.81421887 0 13.24309979 0 30.1234575 3.93390568 0 3.787239483 5.608856089 0 3.154009872 0 6.478622493 7.787308395 15.09366397 5.80027273 6.101276843 13.04811935 3.386846434 5.722827247 0 4.708257359 0 9.567812109 0 0 16.16740322 6.302387749 11.63892929 0 0 0 3.896648631 8.478971299 16.19890746 0 0 0 13.24 5.935157332 2.353434794 9.453312098 9.142142471

192 0 0 7.571593018 0 11.57836818 6.846270116 0 9.029896278 0 5.436683368 0 0 0 0 0 7.567906156 4.144096919 0 6.104475811 0 4.885976545 0 0 0 22.49306671 0 6.837606838 6.463909666 0 0 33.33333333 0 0 0 0 0 0 0.922490522 2.797913234 11.31514919 0 0 0 0 0 4.419204956 0 33.33333333 5.579885006 16.91021955 0 0 0 0 0 0 0 0 0 18.56272838 8.233333333 5.333333333 0 0 0 0 0

193 13.32874907 12.65046385 0 0 9.37371603 10.97691141 4.818046582 29.13765896 21.46367654 9.462497445 44.46460914 33.33333333 0 3.159988994 0 0 0 18.09406086 0 0 0 13.76853906 0 9.709929245 0 2.313983643 9.429680665 0 18.08969953 0 13.11211949 8.15147475 0 12.25838633 7.369922565 0 15.65265706 0 0 8.150477688 19.77714406 0 11.6779021 0 2.403279529 20.80854974 6.777815013 0 0 0 0 9.863663962 0 0 0 0 0 0 0 0 0 0 0 0 0 0 0

194 0 0 5.719616074 18.70180626 0 7.192817007 10.30354449 25.1030816 0 0 0 6.908419374 6.530429927 0 33.33333333 9.704257613 7.087871064 0 0 0 7.097182208 0 11.11111111 0 0 0 12.02229299 0 15.58840845 26.88144776 5.737259075 12.61656767 12.91433734 9.105218549 0 7.148606892 0 19.70214844 10.95212452 0 23.26107766 0 0 0 11.58938124 0 0 0 15.57676079 0 23.01951441 0 9.198155047 0 0 8.400337502 6.312640701 5.500804277 0 0 0 17.43666667 0 0 25.28388621 27.0642186 7.337010324

195 42.9461559 16.25445512 4.242677436 0 5.014341323 0 6.649987894 24.30343706 18.85631982 33.33333333 0 0 8.39764669 9.812815094 15.23862145 20.09002063 23.27717696 15.40470632 16.47600024 27.2253052 5.989157284 11.25670299 39.22144787 11.21823201 0 19.14954886 0 6.092458005 0 0 0 7.600051144 5.845396916 0 0 0 14.07903835 10.09848304 7.66062603 10.27812133 0 0 5.310989552 34.38168379 10.07173969 21.44634479 6.42873101 33.33333333 4.653379808 47.34137698 0 28.67164297 12.12267069 29.03017876 22.2261022 14.53892151 15.43849918 12.00409524 17.68159287 25.13149775 53.21333333 26.13666667 30.60333333 18.17019484 0 0 25.43207762

196 10.10257292 0 0 0 23.9596173 0 0 0 0 32.12901449 24.46261779 11.02815217 19.9031453 8.929667492 0 0 0 0 19.08067557 0 0 45.31511542 0 11.1804901 0 0 7.752403846 0 6.684876788 0 0 0 27.36498309 0 47.4927818 0 0 13.2716988 0 0 0 35.28661877 0 0 0 0 0 0 0 0 0 0 0 0 0 0 0 0 14.42197852 14.77060495 0 0 0 0 10.09231171 0 0

197 6.187870217 0 0 10.09170556 5.848137085 0 0 12.42592611 11.8696568 0 13.35134923 0 0 0 0 0 0 0 0 0 6.037275848 0 5.520665593 0 5.046217049 0 31.97159322 11.74101329 4.3960689 21.53017236 0 11.32252038 0 25.00766699 0 28.53752079 12.03649615 0 0 5.841977206 13.55618927 0 8.758905137 6.892377382 0 0 19.06858098 23.76552122 0 0 0 0 0 0 12.49259332 25.77794145 0 0 0 0 0 0 0 0 0 11.05223146 0

198 0 25.44728302 0 0 0 0 0 0 0 0 0 0 0 0 0 0 0 0 0 0 0 0 0 0 0 0 0 0 0 0 0 0 0 0 0 0 0 0 0 0 0 0 0 0 0 0 0 0 0 0 0 0 0 0 0 0 0 0 0 0 0 0 0 0 0 0 0

199 15.76994814 20.68286948 70.60170038 26.55324983 36.05382663 72.2432258 60.96754563 0 41.10442389 19.63847136 10.15905888 0 57.33696799 69.91395468 49.20868127 56.44364692 62.25715871 29.71544941 49.26064033 62.18476279 51.97580407 19.79761287 35.75671212 56.0704903 56.64649737 38.70289194 18.74332265 75.70261903 25.11748883 40.47628243 40.90940114 56.52214657 38.33587254 49.43128695 41.98328576 47.40651412 51.75318595 34.5866859 63.49567224 58.61400186 23.53695105 42.42629022 61.28810968 53.00311158 67.89197992 36.09285956 59.64587351 0 68.40721988 35.74840347 60.81308237 55.16230532 67.04024497 60.33125 59.43872472 39.33987539 64.65865778 68.32384427 47.76217731 36.20433024 41.34 56.74333333 41.71666667 75.89464783 58.64966077 52.43023783 58.08876959

200 62.06241999 24.76843605 0 44.73095738 22.0454604 0 0 72.07365474 27.54742386 48.85566091 43.28583319 83.30094164 0 0 29.37597775 0 0 20.33250208 0 0 20.54318822 49.57895732 20.58591758 0 0 26.43869378 30.76258128 0 18.84966228 14.33921883 14.56313947 0 27.07887804 0 21.15905947 16.22235819 0 19.87720645 0 0 48.4361758 30.20402236 0 0 0 33.33333333 0 70.9901188 0 25.61983471 0 0 0 0 0 18.1687529 0 0 0 24.90442381 11.77333333 0 18.58666667 0 0 0 0

201 0 0 0 0 0 0 0 0 0 0 0 0 0 0 0 0 0 0 0 0 0 0 0 0 0 0 0 0 0 0 0 0 0 0 0 0 0 0 0 0 0 0 0 0 0 0 0 0 0 0 0 0 0 0 0 0 0 0 0 0 0 0 0 0 0 0 0

202 0 0 0 0 0 0 33.33333333 0 0 0 0 0 0 0 0 0 0 0 0 0 0 0 0 0 0 0 15.75811737 0 10.12151977 0 0 0 0 0 0 0 0 0 0 0 0 0 0 0 0 0 0 0 0 0 0 0 0 0 0 0 0 0 0 0 0 0 0 0 0 0 0

203 0 0 66.66666667 13.77666087 52.90271132 50.02932551 66.66666667 8.204285938 10.70532129 5.052063247 27.1601461 4.02047527 23.21825156 0 33.33333333 27.73809524 25.54377699 13.00083126 54.39189189 0 17.19568899 29.56258499 70.63108374 0 71.58555164 66.66666667 0 0 35.77926754 26.7305768 5.42541734 46.06739217 0 10.4899203 33.33333333 33.33333333 0 23.71271765 66.66666667 33.33333333 7.703703704 22.10433245 33.33333333 59.33240444 17.75046936 13.02035855 21.78561882 20.83295318 51.14558473 41.04683196 51.30294966 45.50227153 0 90.47923323 24.45740201 0 22.04560698 0 74.67564183 41.76224285 4.68 4.06 8.056666667 16.94043471 71.80605687 66.66666667 100

204 33.33333333 0 0 17.09401709 0 0 0 15.21836406 5.785909469 0 0 0 0 0 0 0 8.052434457 0 33.33333333 0 0 0 0 0 0 0 0 0 0 0 0 0 0 0 2.746887967 0 33.33333333 0 33.33333333 33.33333333 0 0 0 0 0 0 0 0 0 0 0 0 0 0 0 0 0 25.734482 0 0 4.23 8.646666667 5.923333333 0 0 0 0

205 4.604246678 0 0 0 2.314207017 0 0 0 22.62801205 5.191560244 0 4.471338011 0 0 0 0 0 33.33333333 12.27477477 33.33333333 0 0 0 59.5823544 0 0 33.33333333 0 23.21181357 0 33.33333333 0 13.76659679 56.17674637 33.33333333 22.86512521 6.410907859 0 0 0 5.154238145 3.129310971 33.33333333 0 33.33333333 0 33.33333333 0 33.33333333 33.33333333 5.799457995 0 22.68518519 0 0 0 0 0 18.72427984 33.33333333 0 0 1.02 8.677617388 0 33.33333333 0

206 0 0 0 0.742151262 0 0 0 0 0 7.567382269 0 1.468463191 43.4484151 0 3.957355583 33.33333333 0 33.33333333 0 0 28.92778946 8.689458689 0 0 10.10633623 0 20.14596802 66.66666667 0 33.33333333 0 0 33.33333333 0 0 0 33.33333333 0 0 0 0 0 0 19.36236392 0 0 0 0 0 0 0 0 33.33333333 0 33.33333333 0 0 0 0 0 5.713333333 0 0 0 0 0 0

207 0 0 0 0 0 16.63734115 0 4.503695263 33.33333333 33.33333333 16.71663473 1.552165387 0 33.33333333 0 5.595238095 7.789556341 0 0 33.33333333 33.33333333 12.168999 8.782998681 0 0 0 0 0 12.03773684 18.97568324 0 13.5920047 19.56673655 0 0 14.59080813 0 14.26191963 0 0 38.70588235 0 0 6.209446205 15.58286397 53.64630811 11.54771451 0 0 0 0 21.16439514 10.64814815 9.520766773 42.20926466 66.66666667 66.66666667 66.66666667 0 0 0 5.093333333 0 41.04861457 0 0 0

208 0 0 0 0 13.76395534 0 0 0 0 0 0 0 0 0 0 0 0 0 0 0 0 0 0 0 0 0 0 0 0 6.621187801 13.34477652 0 6.254455292 0 0 0 0 8.814822936 0 0 0 11.22900088 0 15.09578544 0 0 0 8.176928024 0 0 0 0 0 0 0 0 0 0 0 0 0 5.646666667 3.96 0 0 0 0

209 0 41.89823061 0 23.6562134 8.97366592 0 0 0 0 0 12.83738598 5.186616495 0 0 0 0 25.28089888 0 0 0 0 0 0 40.4176456 18.30811213 6.894639556 0 33.33333333 0 0 0 7.00726979 0 33.33333333 9.427385892 12.98837514 26.92242547 0 0 0 0 0 0 0 0 0 0 0 15.52108194 0 42.89759235 0 0 0 0 15.16458044 11.28772636 7.598851336 6.600078339 0 0 0 10.90333333 33.33333333 28.19394313 0 0

210 12.87655612 0 18.45812995 0 0 23.30499692 15.58323163 13.48484835 5.587130654 12.13333333 0 14.75136807 0 0 0 27.04603888 0 0 34.45640202 8.848120509 9.274916731 10.58818608 35.77761901 0 0 3.233185483 8.217854663 0 0 0 0 9.744950612 3.690925427 11.37721467 23.65640913 0 26.33167283 9.035336216 0 23.95421369 19.30654129 32.70688809 23.06446897 0 9.571855648 0 6.429260063 18.35526125 30.13241775 8.167313497 0 5.602647937 0 11.89279732 7.015658031 0 0 0 0 11.3295053 21.32666667 0 6.033333333 9.283500917 0 12.62605889 9.198677761

211 13.40550604 23.60520905 32.15590947 0 0 0 8.414903694 0 26.56013702 14.95610535 10.92773474 0 16.53868607 31.53590517 2.019794886 0 0 0 0 0 0 23.27628706 14.89886532 30.48759265 15.34552846 11.38814016 20.39886653 27.02774196 17.63541846 20.64156206 21.67968717 0 0 0 0 0 0 13.21886072 26.98635543 11.85336702 0 0 5.679625034 16.13695464 19.88711819 41.73112071 30.54949779 0 0 0 18.36178802 0 15.84300719 0 0 17.67073233 19.92071358 0 13.77676091 0 17.06333333 19.45 0 9.025481424 0 19.95472186 23.7680288

212 0 27.05458121 0 33.33333333 24.13956714 23.92129249 0 27.94014881 0 29.30245514 0 0 0 13.87976861 0 0 0 13.43371715 0 0 0 0 0 0 0 0 0 29.47304211 18.09239838 21.36517615 4.615711253 17.97333333 21.12315149 5.130004679 0 22.88277013 10.65058682 0 7.693284245 0 0 23.0206855 0 19.37823879 0 12.91856348 0 0 0 0 0 0 14.40696504 24.18531606 0 0 33.33333333 0 0 28.69614071 0 0 0 0 0 10.81864084 0

213 0 0 0 0 33.33333333 5.84663738 37.42265969 7.133580411 0 0 38.34907764 38.43412332 39.15219883 0 31.55984801 26.14989929 20.30009309 48.53022167 13.85393379 41.40351308 33.33333333 23.28499491 0 20.04666547 33.90282584 14.95138275 0 0 0 33.33333333 0 0 0 13.02791512 15.84793172 12.18401161 3.435766112 0 10.26088982 3.764066744 26.22207578 15.70326249 9.571730539 0 18.36553409 0 0 9.258390416 10.81786708 23.97589951 0 0 15.83796751 0 54.96127277 33.52329384 0 52.70578441 33.33333333 25.67145534 10.79333333 8.446666667 20.94666667 8.15726991 25.95146178 0 16.0606406

214 0 0 8.479378739 18.8486125 0 0 0 0 8.129401102 0 0 0 0 0 15.26714139 0 28.32410669 0 0 0 45.7820907 0 0 0 17.98780488 0 17.73616861 0 0 0 19.1724415 0 36.53123444 33.33333333 8.808461411 0 12.47676172 12.7103551 8.154542762 15.52526236 0 0 0 0 23.76147769 0 18.33574355 0 0 33.33333333 33.33333333 52.01628605 0 0 10.63097925 0 15.55228277 0 25.23101074 0 8.723333333 0 8.296666667 11.74350218 0 12.87737607 0

215 42.73967269 21.28191068 0 15.49631258 18.92000806 30.80407841 0 0 27.74620268 0 0 0 0 8.364787068 3.107730317 39.32999609 17.69470405 0 0 10.26810392 0 14.83852075 10.74861518 22.88333333 0 43.14083018 19.05322586 8.270443181 29.74722002 11.96815718 0 24.78263917 0 0 18.52546227 33.33333333 9.275857163 0 14.24743602 0 0 23.83018968 33.3346334 22.99753197 0 10.09271523 10.00984446 14.31086152 1.932192496 0 8.85257065 0 17.790141 28.16678133 0 0 0 7.348139973 19.55657242 22.00382803 0 24.47333333 0 0 21.89389202 10.64481417 17.77931432

216 0 0 0 0 0 0 23.22557041 8.161097454 12.22595217 0 0 9.445250979 16.79464727 0 0 0 0 0 28.08077464 20.3307347 0 0 0 0 0 0 12.9344668 0 0 0 0 15.1496388 4.924291168 0 0 31.59988492 0 24.29799712 0 0 17.6 0 11.39141623 17.1963787 0 9.295679946 0 0 0 0 11.07309487 0 11.98757492 0 0 16.63748943 7.466863034 10.10983194 0 0 8.416666667 0 0 0 26.59141519 0 0

217 0 0 10.07657383 17.83702075 0 9.41204084 0 27.01882994 13.43512515 21.2 31.56036454 18.58196526 0 36.64997875 31.23253436 0 0 27.19719465 0 0 11.60965924 17.95496493 35.45499172 0 32.76384083 0 0 6.305591377 26.12408942 12.69177127 28.71762208 5.143333333 0 28.20332865 9.974683544 0 0 40.73745084 23.52965118 21.20682965 29.365205 0 0 0 0 0 0 24.07494292 12.28886773 17.6325524 0 29.49160805 0 15.93671488 6.168446026 0 13.41261976 15.6043956 8.102322594 7.661877993 4.723333333 3.633333333 33.03666667 37.48239366 14.1237897 0 3.84893043

218 22.00743934 18.33017477 14.87520338 0 9.193766196 0 0 0 0 22.40810618 0 18.78729237 0 9.56956041 16.81295104 0 13.03324024 0 0 0 0 10.05704627 0 0 0 0 0 13.99422342 0 0 25.814538 0 21.52021563 0 0 0 22.68274651 0 0 14.3171409 0 0 10.2675643 0 0 20.41476985 26.10732441 0 16.1846228 0 0 12.88945796 10.32061913 0 0 0 0 14.23184807 0 0 17.16666667 10.70333333 0 24.30785191 0 19.01142332 11.20491534

219 8.97082581 9.728124286 15.95480463 14.48472084 14.41332527 6.710953963 15.35363458 16.26149504 6.316051225 0 19.16282308 0 27.51446783 0 0 7.474065742 20.64785592 10.83886653 23.60888955 19.14952778 0 0 3.119908765 26.58240855 0 27.28646142 21.65941754 14.92895795 8.400873714 0 0 27.20610475 12.21018184 8.928203543 23.18705192 0 15.14660884 0 9.127840548 9.379119642 7.506177924 4.738974241 6.690561529 24.2908959 28.41401438 5.547150782 8.568329718 34.0005439 28.64403215 16.89090125 28.37921312 0 13.81372522 19.81839042 21.22364392 32.1684844 10.31418753 0 0 4.637192619 3.82 6.696666667 0 0 11.43944132 14.06696484 18.13949275

220 0 0 0 0 0 3.812537673 11.33851106 0 0 0 0 0 0 0 0 0 0 0 0 0 9.670582001 0 10.30359101 0 0 0 16.2259743 0 0 18.85467693 0 0 13.63144731 0 9.308115099 11.67506808 0 5.36493047 0 0 0 0 14.17447051 0 12.80511811 0 0 0 0 0 3.854451913 4.970717063 0 0 0 0 33.15297204 0 0 0 0 0 0 0 0 0 0

221 5.7 11.15966659 12.32463524 19.91386736 7.868124901 12.65639855 0 5.171898356 0 0 0 9.774555883 20.39575589 0 4.404945904 22.01616738 0 0 0 0 12.21508095 0 0 33.33333333 23.12510705 7.055540792 0 1.753408373 0 0 12.31944735 0 0 17.98708037 0 0 0 0 0 0 0 8.409559726 10.18421563 0 0 30.8342498 0 0 0 0 9.927212137 0 0 0 0 9.688888889 0 0 12.51566239 0 0 0 0 0 0 18.08278867 0

222 3.628187966 0 0 0 0 9.613928842 0 12.5483871 14.02755817 0 0 12.07072726 0 0 0 0 0 19.61706961 0 0 8.01854784 16.76930166 16.28447025 13.96290051 0 12.28559705 33.33333333 0 0 0 20.55460242 0 0 0 42.1098099 29.48853367 0 10.28942116 0 0 0 14.80913026 0 0 0 0 7.521974307 0 0 5.840488899 0 16.09823318 0 0 0 0 0 0 0 0 9.22 6.35 0 7.396101625 0 0 16.36323179

223 9.042664327 0 0 33.33333333 16.29276054 40.61415942 24.68963917 12.82006004 11.89417989 22.26392165 45.33711589 0 0 18.04246364 15.87667863 0 18.99108556 17.64545057 29.26893007 9.66976564 0 32.7439167 18.55955679 0 28.22631413 23.32482993 22.49431137 21.2101877 17.22976562 24.99201847 0 33.0158274 20.93298359 32.53923542 14.70740382 0 76.45547967 37.68318677 31.70590564 23.39022271 13.77433485 8.178272268 9.353388658 53.11150694 0 0 24.471435 17.9420541 0 6.689548422 0 32.09574772 3.53133638 23.85600554 39.27524184 0 0 33.58757394 44.63068806 25.11102179 21.37333333 5.946666667 0 13.80361757 18.30528978 0 0

224 0 36.89762123 0 0 0 0 28.53289807 10.93672147 0 15.27432378 0 0 16.96939119 0 0 0 15.28120713 0 0 33.33333333 0 0 0 0 0 0 0 13.5814852 0 0 0 0 22.88468789 0 0 0 0 0 18.58460023 12.80411613 0 0 12.01085903 0 28.19387026 0 0 12.30538922 0 18.42875052 4.297114794 0 0 0 0 0 36.14006515 0 0 0 0 8.956666667 9.81 0 0 0 0

225 0 0 30.1678117 13.41946598 17.97922568 0 0 0 0 0 6.511111111 34.19185736 12.93757745 7.040380712 0 56.82529405 0 20.71219812 16.51299246 33.33333333 48.30167385 8.960663567 0 0 17.34340573 0 7.810342997 13.30311615 0 0 23.12998403 5.807597704 0 21.97361517 19.63857123 22.04848852 0 0 0 10.57160903 0 28.39586262 0 19.45780718 0 41.0490835 26.3218109 0 72.0981243 0 0 0 52.79977769 33.33333333 0 90.31111111 10.30143881 19.05649555 0 33.33333333 0 9.413333333 0 47.29058022 16.40826873 19.64373767 17.03024555

226 24.29066901 0 21.00869809 33.33333333 33.61055219 0 0 41.29821953 52.6391085 0 0 0 33.33333333 0 0 0 0 0 48.56527977 0 0 0 0 0 0 0 20.13603799 0 33.33333333 34.45378191 18.03759095 7.917656374 0 16.14035088 0 11.08407208 10.56910569 46.6624616 0 0 38.41061241 18.52420307 0 0 0 17.29807945 0 0 0 0 18.50380669 9.235998839 0 0 49.64465892 0 9.272529859 26.55999171 0 0 11.45666667 8.11 18.46666667 0 0 15.25054466 10.8197672

227 57.3384787 36.31569662 15.09475735 0 15.35410765 33.30297551 26.79525754 17.2247135 21.43915344 11.06941168 48.151773 31.03633463 16.36394215 47.78500945 33.33333333 11.31716595 65.7277073 26.33739893 0 23.66356769 0 33.92274997 49.34198041 52.70376616 21.09694681 26.27779254 0 12.12314564 49.43690104 0 0 44.4624023 30.15053147 0 4.918285538 15.12964448 0 0 34.96076103 37.81087196 33.33333333 0 54.27706617 12.22649162 13.42519685 0 32.82288146 69.75255668 16.91736667 61.61677978 48.58788782 7.60510396 43.66888594 42.81066113 0 0 11.13299415 20.79593879 33.43291156 18.96528343 15.58666667 5.546666667 33.39666667 11.63307494 65.28644148 47.02292899 43.7367161

228 0 0 21.40409762 0 0 0 8.643694164 0 0 18.05900955 0 0 0 27.1321462 46.38504213 0 0 0 0 0 9.179672774 7.603368104 0 0 10.20822628 21.04773629 0 17.99843976 0 15.44806479 13.16725979 0 12.40034974 0 0 0 0 0 14.74873311 15.42318017 0 0 0 15.20419426 0 10.81858725 8.861898336 0 0 0 0 12.26438309 0 0 0 0 0 0 0 22.59036145 0 0 0 19.87662565 0 0 0

229 0 15.62701557 0 0 8.895229038 0 0 0 0 33.33333333 0 12.92652487 0 0 0 9.841372612 0 15.68788276 5.652797704 0 12.61444258 0 5.510401548 0 0 10.0085034 0 20.03021719 0 6.251457896 12.79111547 8.796516231 0 11.35971816 9.31781442 10.57419317 12.97541464 0 0 0 14.48171941 21.68297206 0 0 45.57581478 0 0 0 10.98450903 7.424432372 14.82952665 17.72981615 0 0 11.08009924 0 0 0 9.420737992 0 0 4.326666667 8.936666667 0 0 0 12.05003935

230 26.79140128 0 0 30.94641373 0 0 0 0 0 0 0 0 19.36825686 0 0 0 9.113894279 0 11.34128167 20.81184437 3.755500858 7.565518967 7.522804704 26.20014331 0 13.06010929 0 10.54594387 0 0 14.72153972 0 10.75459866 6.654180897 9.636897462 0 8.782975816 11.40111141 23.11344166 0 0 9.413987426 0 12.18523261 0 0 11.99740597 0 0 0 0 0 0 14.45348569 19.29616449 13.97183562 2.53317914 0 0 14.66843167 0 10.48 8.72 0 3.268667273 7.500506791 9.100941253

231 0 0 11.89637379 0 0 5.616403464 0 0 14.64257242 15.60582361 10.0081367 6.663479924 15.11880669 0 0 33.64794772 0 7.799957752 0 0 6.939361904 7.13713495 0 23.93984221 24.10479028 0 36.60146661 0 0 0 0 5.883333333 0 8.048792201 0 10.63132643 0 14.60913845 0 1.679821898 12.33816532 3.87742339 42.59451007 0 0 23.45822365 16.01175483 23.17341541 14.48623165 0 0 0 24.42760943 8.930012683 0 0 12.69516356 14.74542422 9.259826442 5.995913478 20.27333333 11.25333333 0 7.802764798 0 0 0

232 0 0 0 19.14310197 11.74484053 10.32830343 17.75533109 0 0 7.106429228 0 16.5855499 0 0 0 0 0 0 0 0 0 8.928914505 0 0 0 0 0 0 0 9.687721789 0 0 0 0 0 33.33333333 16.03183627 0 0 0 8.927363722 0 0 4.848484848 0 9.857859001 0 0 8.819035145 18.55633647 31.80718199 20.47139217 0 0 0 5.859271083 0 0 7.590996169 0 0 6.45 0 0 0 0 0

233 23.53690747 12.56606496 26.98884885 7.915702904 0 20.74604401 9.635229182 13.66462386 0 0 0 0 0 0 18.86751675 15.20481928 46.03398601 41.42479185 12.7648814 0 14.32865731 0 18.16054716 0 0 16.7183827 11.84969091 0 0 10.70116896 15.73212439 0 14.69595669 0 27.88024338 0 13.13967411 21.2390493 0 0 33.33333333 31.96420733 23.74440276 26.46541094 16.53696498 0 0 12.80404107 0 0 0 0 9.164908049 24.01897983 47.37050217 0 0 9.657534247 0 0 0 0 0 20.42396094 23.98979244 4.549634043 24.23239208

234 0 12.59589691 9.362656133 0 0 0 0 13.27033537 11.74671241 21.60977767 16.08826559 8.56277884 7.626864553 0 15.46175373 33.33333333 0 0 0 22.58518255 10.49451779 16.37884801 52.25299483 0 22.95187271 0 0 0 16.95041816 0 37.47249984 0 0 0 0 20.25441329 0 0 33.33333333 0 0 8.608321377 0 0 0 8.865678892 15.62363412 24.95389063 0 46.60243583 0 0 0 0 13.34801762 36.3566896 0 0 0 0 0 15.02666667 7.023333333 0 0 17.31461189 0

235 0 0 0 14.19023136 12.99919727 0 10.32717383 33.33333333 22.62151578 0 0 0 0 25.30377051 24.05660377 11.16329726 0 25.53337558 40.84451068 33.33333333 0 26.19619838 0 0 0 8.100042034 0 0 24.48577411 0 0 53.56666667 33.33333333 17.26532509 22.5118479 22.7020069 23.93936709 0 6.844650471 0 8.631182915 0 0 0 0 15.1054554 0 0 18.60206071 14.77699686 11.68875297 33.33333333 33.33333333 14.21076905 0 7.209364193 19.57156231 16.3364841 0 0 0 11.64666667 0 46.12644667 24.53631139 0 46.87902188

236 19.21568627 26.4784161 0 0 0 23.00502991 0 19.66870948 0 0 33.33333333 50.08111677 0 0 0 0 33.33333333 0 0 0 0 17.7857829 0 0 37.20324552 0 12.02299328 89.45405613 0 23.64561154 12.39560964 27.45 0 0 0 0 0 18.72419488 15.17024507 31.65351143 0 0 0 47.79408543 33.33333333 9.156672378 21.33592737 0 24.51429819 0 23.17073171 12.86194116 0 9.314353499 0 0 53.97150311 0 49.81584406 0 15.34333333 0 14.3 0 0 16.89378057 0

237 30.45600498 27.62218561 20.46406872 0 54.92182614 0 0 0 32.29843848 26.22690411 17.24506774 0 13.96507648 66.66666667 27.14830916 6.65060241 11.51878638 14.47389178 14.48087432 4.627583061 26.39397143 0 22.06365331 28.95913877 0 49.94828397 32.40970542 0 49.71624851 55.96549771 8.69964421 0 41.21611132 15.82020491 33.33333333 13.07892004 0 12.47608636 0 48.7931808 0 46.13606047 8.435631099 8.706786172 50.12970169 0 35.03127771 26.89602894 14.73127263 13.93915025 0 33.33333333 24.16842528 0 0 4.473062088 0 27.93151642 33.33333333 33.33333333 0 9.653333333 0 12.90937239 0 0 0

238 0 0 19.2137491 8.662941191 20.33413606 40.30421919 17.07463403 20.06299796 18.69076092 11.72355566 0 0 33.33333333 0 14.46581658 0 0 0 20.56845193 18.64205669 38.08799071 0 0 0 0 12.17318201 0 0 0 0 0 13.1 0 52.2114969 0 0 0 0 0 0 24.40596961 0 0 0 0 0 0 12.17262395 0 0 0 0 8.905723906 0 19.98531571 0 11.22859188 0 0 18.66490166 0 0 19.86666667 0 27.89734263 28.78369929 19.78764479

239 0 20.73743643 12.07430341 19.14160884 0 0 45.20763187 0 0 17.72750973 23.32519664 18.10707457 10.58766209 8.029562821 0 0 0 10.76798303 0 0 0 16.00760228 0 20.90087572 15.74009149 0 7.116143777 0 8.847559223 0 10.9785822 0 0 0 6.637677928 0 38.10614672 21.55041959 21.53832947 17.87348587 12.3639851 0 25.22545607 0 0 33.55611067 0 0 18.84710168 6.125080593 0 0 0 29.07239924 0 32.12977742 0 31.32904102 0 27.33741985 23.92666667 0 0 12.7374552 20.30788627 24.95776742 0

240 0 0 0 0 0 0 0 0 0 0 9.426169591 0 0 0 0 0 14.90951841 0 22.3427255 33.33333333 0 0 0 0 0 10.19188596 11.27784784 0 10.34786438 16.26451405 0 25.05644682 0 0 0 0 0 0 11.79115828 0 0 5.370181695 0 0 6.789748573 0 21.81818182 0 0 0 15.77259728 20.2239695 12.18598196 0 28.9700375 14.95030051 0 0 18.74571805 0 0 0 12.10666667 33.33333333 0 0 10.13307368

241 46.16011654 36.15836351 27.52524654 26.08908468 14.91114191 18.26454439 55.27003047 43.35984212 0 18.02497162 16.60925479 19.65348399 61.85690531 39.85384836 47.32684391 70.34684902 17.61382519 21.20915945 37.18361212 42.44091151 0 33.33333333 61.50591716 0 17.30513939 37.56683977 0 18.14799504 0 0 17.18972546 0 0 21.27733306 46.52985564 38.73987537 0 36.4447233 12.81481481 52.98138182 24.86894432 0 20.42230991 25.61483163 40.80869328 0 20.7866436 33.58041989 54.56098265 33.35833108 20.29739777 50.02831134 31.42317069 19.86101771 0 0 19.83566839 33.71689365 0 47.41205396 37.59 20.31666667 21.40666667 26.84754847 35.49121805 6.446540881 20.7597405

242 0 0 17.68543342 12.05581209 22.11392884 0 0 13.54177207 0 11.82823872 30.72237587 33.33333333 0 0 23.22712418 0 0 19.9379845 0 0 22.08555928 39.23745166 0 6.575712881 0 0 26.98992925 24.69514594 0 0 33.33333333 0 45.49618115 10.22700402 0 0 50.07398754 17.21143811 0 0 33.33333333 0 22.67898037 42.95433536 0 21.68274717 17.40213111 18.25897551 0 14.34741531 23.48840852 0 0 0 21.83515483 0 0 19.62028359 0 0 0 26.76666667 20.52 0 0 0 20.9745158

243 0 0 0 0 0 55.4517134 0 0 58.19987691 12.80945536 18.5124269 0 0 33.33333333 0 0 26.78025339 16.77404922 0 0 15.45823364 0 0 0 56.73030863 0 12.62295082 29.28103236 44.24195112 0 16.14360788 9.292765625 0 0 0 33.33333333 0 0 11.16622028 0 0 47.99576922 0 0 0 17.2451091 0 0 0 0 8.296501719 0 26.36970679 31.57326084 8.975996594 12.78133489 50.1874439 0 72.31093544 0 0 10.89 10.52 11.93904963 0 20.39345712 11.2041545

244 0 0 0 26.50923094 0 0 0 0 0 0 0 17.32953124 14.65987632 0 0 0 4.089515892 0 8.360411589 0 0 0 0 14.86958928 0 0 0 0 0 27.44717164 27.74483378 22.16797206 0 15.58946738 12.28506938 0 0 7.55804101 0 0 33.33333333 0 0 0 13.25559739 0 0 0 5.73969557 0 0 0 13.06009769 0 0 24.66173793 0 0 0 12.56656017 4.45 0 0 0 6.912490808 3.966417468 0

245 9.907798093 18.15308389 12.27528624 0 8.487565067 0 0 4.856282799 0 0 0 0 0 0 0 9.610158373 0 4.858977939 5.289921846 0 0 0 0 33.33333333 0 23.14144737 9.676720442 0 0 0 0 13.9826567 0 11.9135041 7.209900354 10.85013146 0 0 20.51851852 0 0 0 33.33333333 0 0 12.60253542 0 0 5.991639573 7.135465875 0 0 0 0 7.878728734 0 8.673040654 4.057971014 0 0 0 0 0 0 4.879996243 0 0

246 0 0 0 7.244248654 27.91226276 0 17.00741119 19.79156127 0 5.429436247 2.610957461 0 0 10.90230574 0 11.44671429 0 0 0 11.09838361 0 0 6.571985519 0 0 0 5.031525851 0 0 8.66017373 0 0 10.73151901 0 4.432559826 0 0 10.64898963 19.71104231 14.34572569 0 22.17830989 0 0 0 0 0 8.434886499 0 5.712873734 5.297974983 13.10936383 0 0 0 0 0 0 0 10.54812834 0 0 0 6.485784868 0 0 0

247 0 34.02691611 15.45756716 0 0 0 0 0 11.6069003 0 22.11881538 0 0 0 0 0 15.71950814 13.39534884 0 7.013927066 17.87509969 0 10.81831278 0 9.936358037 8.029046499 0 4.05230097 8.79568472 0 0 19.44224778 0 5.967356274 0 0 0 12.01491273 0 11.06902357 0 16.99691766 10.65435297 10.07793128 0 11.65058616 11.51515152 0 6.769283393 0 0 0 4.691186676 0 0 0 0 0 0 0 0 0 14.11333333 0 33.33333333 43.47632609 0

248 20.50321713 0 27.05646663 6.82410239 26.57510143 0 27.72255834 18.45054175 30.19322279 20.52387797 0 29.68365143 23.48321837 0 17.57996145 8.596278317 6.553079948 23.82448006 6.112916328 0 44.58110738 27.42921501 14.6988834 0 16.02819394 0 30.00001697 8.638187397 20.092502 39.21949502 5.588499551 0 16.32979898 35.02533517 14.86660238 7.070817008 33.33333333 16.12189522 0 21.60386892 8.464389009 0 0 7.718501703 13.28798737 36.81902214 15.93120222 15.07435782 26.93839882 0 9.844924812 16.63835533 12.2698562 13.47231562 0 13.84650948 13.6490839 0 8.943346509 0 14.18333333 2.87 0 0 11.57684631 12.93987622 24.93241036

249 23.42886824 11.66163649 0 21.27752125 0 26.28374222 0 0 0 31.38402008 0 0 0 15.91051257 11.86607045 0 14.33429903 0 20.71041262 6.113444487 0 0 6.404901142 45.22136451 0 21.0707804 4.401008827 15.18533829 16.52199778 8.40864555 0 10.057911 27.44250086 0 14.67601243 10.00584283 16.59267913 0 23.99824579 0 0 7.458821532 12.91102343 13.63440003 25.85797339 0 12.54668973 24.65136028 0 39.445914 17.00219492 0 0 35.09340583 32.34008235 33.7601172 7.654763159 42.60485175 0 29.47325753 0 12.97 4.956666667 21.3942837 7.80611526 12.77738222 11.99610516

250 0 0 0 5.766369048 0 15.93484625 0 0 0 0 14.1688253 10.1370779 0 0 0 7.640755893 0 0 0 0 0 17.17870808 0 9.287402904 14.10001742 4.087586456 0 0 0 0 0 0 0 0 0 8.708876474 0 0 0 0 0 0 5.042479276 0 0 0 0 0 0 0 0 0 12.41179464 0 0 0 0 0 11.06126738 0 0 14.08666667 0 18.05299586 0 0 0

251 0 8.700980392 4.557612068 0 0 0 11.59124187 0 10.05233058 0 8.661257606 5.965085098 20.97911495 0 0 0 7.862113461 0 0 0 17.73212081 0 9.971959341 0 0 9.421611761 2.836361105 10.30277979 0 0 13.74666667 0 0 10.22291421 7.089139571 7.847862531 12.66444904 5.139060994 0 0 20.41833806 0 0 0 0 15.05721994 0 6.813467369 0 0 5.061583322 0 0 0 5.281666329 0 0 0 6.690223793 8.345833902 12.27666667 0 0 0 3.62586507 4.968383017 0

252 18.97341326 6.298076923 0 6.74731431 0 9.662316597 7.371175065 9.491859009 0 19.76016435 0 0 0 0 0 0 0 0 14.38251971 16.47304141 0 9.207587019 0 0 0 0 5.733775811 11.87320778 0 0 0 0 0 0 0 0 0 0 0 0 0 17.73292414 0 0 0 0 0 0 0 0 3.269558969 0 0 0 0 0 0 0 0 0 0 0 0 0 0 0 0

253 0 16.48673441 8.567032122 0 0 0 0 0 0 10.3486386 0 0 0 6.372800273 0 0 0 0 0 0 0 0 0 0 0 0 8.470801142 0 0 28.8237632 34.91827936 3.915677733 0 0 15.89519297 0 0 0 23.09409034 0 0 0 5.52034165 8.059981256 0 0 0 0 0 0 0 0 0 0 0 7.925575166 7.52456589 0 0 0 0 7.606666667 0 0 11.63118171 9.109070815 0

254 0 0 0 0 37.2796212 0 0 24.69177952 21.33560842 0 0 0 19.89699571 0 0 0 0 33.33333333 33.33333333 0 20.44957852 0 0 46.2785631 0 0 0 0 49.62909515 0 0 0 49.20073512 10.57763275 0 0 0 33.33333333 33.33333333 0 0 0 0 0 0 0 0 0 0 0 61.60508334 33.33333333 0 0 33.33333333 12.20731707 18.19025295 0 0 0 33.13 0 0 0 17.67078504 0 17.70623742

255 21.79354839 0 46.48176311 0 0 0 25.96215827 23.84147432 29.77217733 15.26890876 58.00540906 51.26664881 0 81.82448131 80.90133533 66.66666667 92.13788654 66.66666667 42.24865164 66.66666667 16.14553902 59.28447376 55.04713663 0 85.89998258 42.90159888 0 54.79345889 0 50.11241569 38.68219043 0 33.33333333 56.14660312 59.5775271 51.25623288 82.48959312 45.2784498 33.33333333 33.33333333 66.98290777 64.48733232 89.43717907 85.7522041 100 70.9356666 94.4086965 50.99937506 100 76.99507077 30.06377436 60.40906995 77.82733131 33.33333333 35.62413659 37.508936 59.14210078 84.66917136 76.53590577 72.18664071 18.92 39.39 79.88 48.6136708 45.713642 61.69828365 66.66666667

256 59.23303835 24.63235294 0 82.95222475 53.41358928 41.06950382 55.0754248 23.05518004 26.84215876 13.57316898 0 0 25.79055601 0 0 25.69257744 0 0 0 0 23.45265975 0 0 24.04593043 0 0 33.33333333 23.03055355 33.33333333 16.18831107 0 62.75098893 0 18.82841938 0 25.4854708 0 0 10.23924299 66.66666667 0 0 0 0 0 0 0 33.33333333 0 0 0 0 0 31.23362937 0 0 0 0 0 0 0 0 0 33.33333333 0 24.22426252 0

257 0 43.88185534 24.76630121 0 0 33.33333333 0 0 11.99772492 41.04911931 19.16450804 27.36824824 33.33333333 0 0 0 0 0 0 16.86029193 12.88375481 0 14.27736885 20.38810356 0 29.24574688 38.77447226 0 13.09426786 0 0 33.33333333 7.38236416 0 17.43814036 0 0 0 0 0 12.59875418 0 0 0 0 0 0 0 0 0 0 0 0 0 0 0 15.14308038 0 0 0 0 12.62666667 0 0 12.03668323 0 5.287279231

258 0 0 0 4.534091889 0 0 0 8.641553811 0 0 0 0 0 9.625893232 0 0 0 0 10.03549532 0 0 14.32923114 20.70353518 0 0 14.34345602 0 0 3.943303653 0 12.65286354 0 0 0 0 0 0 0 0 0 0 0 0 0 0 0 0 0 0 0 0 0 9.760874047 0 0 0 0 15.33082864 0 14.51884177 0 0 0 0 9.321842965 0 0

259 0 0 15.62729149 0 9.306789514 0 0 10.27815329 0 0 0 5.262939959 0 2.176825184 19.09866467 0 0 0 0 0 9.336347084 0 0 0 0 0 10.85125635 0 0 4.875510034 0 0 10.08356739 4.22443054 0 6.701557314 4.845957841 16.24915587 0 0 0 17.77974353 0 6.187814643 0 14.00711346 5.5913035 8.853824238 0 23.00492923 0 6.257596714 0 35.4330373 25.76086375 42.35817176 0 0 5.712603062 4.948683623 6.56 0 0 0 0 0 10.33981668

260 0 0 0 0 5.254204736 0 2.696772548 0 0 0 1.532481124 0 0 0 0 0 0 0 0 0 0 0 0 0 4.348118732 0 0 0 0 0 0 4.487389453 0 0 0 0 0 9.043010753 0 11.42680165 7.140044353 0 0 0 0 0 0 0 0 0 6.169576407 2.685810442 0 2.805044308 0 0 0 0 0 1.074190178 0 8.75 0 4.209621993 0 0 0

261 0 0.566225419 0 0 0 0 0 0 3.488711156 0 0 17.35903255 0 0 6.947601562 7.473417338 0 7.688642224 6.966981485 5.482185831 2.466632877 6.138040042 0 0 0 0 0 11.2534281 5.597745103 0 1.952002704 0 0 18.10165731 25.65838647 2.821116984 10.28074933 0 4.309358964 0 16.79817521 0 35.60569021 0 2.729996384 15.36870047 0 15.42124542 10.89873185 0 3.643125819 0 10.18673994 0 17.16755058 0 0 0 0 0 0 0 0 0 0.722066604 12.40076821 0

262 12.48424544 0 10.97634022 0 0 0 0 0 5.252927861 0 0 0 0 4.897424032 0 0 0 0 0 0 0 0 0 6.595972985 0 0 7.079386062 0 0 0 0 1.640724744 0 0 4.604505838 0 0 0 0 17.90382244 0 0 0 5.141338016 0 0 0 11.8371221 0 0 4.980502493 0 0 0 0 0 0 0 0 0 0 0 4.313333333 0 0 0 0

263 0 21.03559871 1.957081075 0 0 0 0 0 0 0 0 16.93076374 5.865048722 0 0 0 49.6551367 33.33333333 4 0 0 0 0 0 0 6.390149952 0 0 2.328851887 0 3.252531451 0 18.81399229 0 0 47.76334776 0 0 0 0 0 0 21.37076378 0 0 0 0 0 0 0 0 0 0 0 0 0 3.137403615 0 0 0 0 0 3.113333333 0 8.879015223 0 0

264 0 3.68879531 0 0 5.181049536 0 10.31171019 0 0 0 0 0 0 0 33.33333333 2.565557952 0 0 0 0 0 0 45.86745658 0 0 0 0 0 0 0 0 0 0 9.417293658 0 0 0 0 5.085602514 0 0 9.0360265 6.151195548 33.33333333 33.33333333 5.644689074 6.287676826 0 7.109505554 0 0 10.46492423 0 22.68041237 33.33333333 0 0 1.999292286 7.550909289 0 0 12.14666667 0 8.470828372 0 5.26339147 11.67107489

265 0 0 0 22.39458026 0 0 8.243398393 0 0 43.00992471 0 0 0 14.81383741 9.787177441 15.47169752 23.64496555 0 0 9.407162939 0 50.4720926 0 0 0 0 0 13.26436447 20.7647876 4.848342592 0 0 0 25.77554926 14.37535982 0 37.99411957 3.61235518 0 8.418341061 26.15112655 19.4730922 0 7.077876149 4.380391549 0 13.24559071 9.16413603 6.096029787 25.89318869 3.357846245 7.90132926 8.746410582 0 23.89509291 3.922628702 0 33.33333333 0 0 4.03 0 0 6.504219811 0 31.75004462 15.30954745

266 45.65879812 5.476426389 22.10774817 0 0 12.69035533 0 0 12.76391312 0 0 10.99069809 27.46828461 10.68526607 0 0 0 0 8.217028193 61.18448084 0 0 0 33.33333333 10.9165023 26.94318338 0 10.9976755 0 0 9.993316146 0 0 0 5.90728864 0 0 24.29032258 9.515061728 0 0 0 0 0 0 0 33.33333333 0 0 0 10.20379773 0 32.62595658 0 0 7.055924361 0 0 0 0 0 14.59666667 0 0 33.33333333 0 0

267 20.84908789 23.91555798 22.35699311 54.19323429 25.9595561 46.4283026 61.10811376 51.46732033 14.3254137 23.65674196 35.76408015 35.28484473 66.66666667 0 0 0 5.125255624 5.936764211 0 0 97.53336712 10.05653403 10.36651339 35.68564772 21.28289695 66.66666667 59.58728061 46.45198893 12.56854573 49.08426984 15.79231827 69.52450852 25.66299064 5.113084344 7.219928067 27.59167994 23.052584 7.088038016 33.33333333 15.42951089 7.693423698 0 11.96256955 33.33333333 11.46124773 32.63525678 0 17.91208791 22.43460148 49.7339718 0 0 0 51.21188483 0 33.33333333 45.08369778 23.83069475 66.66666667 41.94338962 16.50666667 12.19666667 10.78666667 7.533546064 4.931167601 3.703457152 0

268 0 0 0 0 0 0 0 0 0 0 0 0 0 0 0 0 0 0 0 0 0 0 0 0 0 0 0 0 0 0 0 0 0 0 0 0 0 0 0 0 0 0 0 0 0 0 0 0 0 0 0 0 0 0 0 0 0 0 0 0 0 0 0 0 0 0 0

269 21.00786855 45.3173962 42.60183742 23.41218545 63.60518963 40.88134207 17.6400051 48.53267967 64.16903416 33.33333333 62.70343873 19.43466089 0 69.60347248 49.93188766 74.48932719 21.57464213 53.04126023 80.81599032 23.92617039 0 33.33333333 43.76603003 24.38504596 63.45248202 0 33.33333333 18.032543 58.74006968 46.06738757 69.00983143 24.34737729 55.52301707 41.59241543 42.23453117 21.82385531 28.6725471 55.96627347 47.75664346 46.82152396 42.21723019 71.4908813 24.90978091 21.11411917 48.095031 46.35135368 47.13339913 45.66540853 53.46113133 24.37283951 71.6451513 78.94793607 48.4408929 23.30265849 25.60402318 55.6881136 51.7788986 40.83667963 25.78242404 56.9824202 84.42 65.27333333 125.4633333 73.28178376 52.13441724 46.88233855 73.01937765

270 65.36048266 29.63393991 31.51179771 62.0447588 0 33.33333333 61.33311131 31.80752527 0 65.82015504 0 64.56637317 93.58290571 0 31.03133556 0 63.13908146 33.33333333 0 59.86666546 96.27351725 64.73844763 0 62.23510566 0 100 58.59861207 66.66666667 28.82066767 30.26456237 0 63.29921866 32.48830226 33.33333333 30.02835864 62.26951283 63.87038931 31.25915568 27.57591532 33.33333333 31.73225499 0 63.60562349 63.58537794 29.81232782 29.65473698 33.33333333 28.82091809 31.73512714 57.76105168 0 0 30.85084252 63.39011745 62.47749096 33.33333333 29.45505171 22.93183322 64.92606471 0 0 25.40666667 0 0 33.33333333 30.81837447 0

271 0 0 0 0 0 0 0 0 0 0 0 0 0 0 0 0 0 0 0 0 0 0 0 0 0 0 0 0 0 0 0 0 0 0 0 0 0 0 0 0 0 0 0 0 0 0 0 0 0 0 0 0 0 0 0 0 0 0 0 0 0 0 0 0 0 0 0

272 0 0 0 0 0 0 0 0 0 0 0 0 0 0 0 0 0 0 0 0 0 0 0 0 0 0 0 0 0 0 0 0 0 0 0 0 0 0 0 0 0 0 0 0 0 0 0 0 0 0 0 0 0 0 0 0 0 0 0 0 0 0 0 0 0 0 0

273 0.566798387 0 0 0 0 0 0 0 0 0 0 0 0 0 0 0 0 0 0 0 0 0 0 0 0 0 0 0 0 33.33333333 0 0 0 0 0 0 12.45254366 0 0 0 0 0 0 0 1.687327824 0 0 0 0 0 0 0 0 0 0 0 0 0 0 0 0 0 0 0 0 0 0

274 0.739385624 0 0 0 0 0 33.33333333 33.33333333 0 0 0 0 2.795387981 0 0 0 0 0 0 0 2.426547744 0 0 0 0 0 3.073221662 0 18.86832589 3.068770967 0 0 51.53508772 0 16.68784413 0 0 22.4691358 0 0 0 0 0 0 33.33333333 33.33333333 33.33333333 0 0 0 0 0 0 0 1.202176915 0 0 0 0 0 0 3.5 4.223333333 0 0 0 0

275 0 0 33.33333333 33.33333333 0 0 0 0 33.33333333 0 33.33333333 0 1.506514658 33.33333333 17.68361582 33.33333333 0 0 0 9.307875895 0 1.928219034 0 4.431561006 0 0 33.33333333 0 0 0 0 0 0.845031078 11.35436789 0 0.861616306 0 0 0 7.112332112 0 0 0 0 0 0 0 0 1.598206192 0 21.74940898 16.75774135 0 33.33333333 0 33.33333333 33.33333333 0 0 0 0 0 0 0 14.42735043 0 12.21144968

276 0 33.33333333 0 1.74507355 100 0 1.331513497 1.525808063 33.33333333 33.33333333 0 1.241859761 0 0 35.6353311 33.33333333 3.527585211 33.33333333 0 0 0 0 0 0 0 0 0 0 33.33333333 0 33.33333333 34.21004365 0 0 3.304974689 33.33333333 2.796277357 2.074177653 0 0 1.601078344 0 35.00841279 0 0 37.01192968 33.33333333 0 0 33.33333333 33.33333333 33.33333333 5.555555556 3.276549219 33.33333333 33.33333333 0 0 33.33333333 0 3.633333333 0 0 0 33.33333333 2.514958863 33.33333333

277 33.33333333 0 1.821535624 0 0 0 4.002041858 33.33333333 0 0.846511628 66.66666667 34.19176707 2.115191652 33.33333333 0 0 33.33333333 33.33333333 33.33333333 3.656234476 1.299935003 0 0 33.33333333 0 0 0 0 18.97767311 0 66.66666667 0 15.13157895 0 0 1.002434484 20.88078967 10.86419753 39.09075134 0 0 33.33333333 1.38596372 34.92666667 35.16701102 0 0 4.51241524 33.33333333 4.571580831 11.58392435 16.57559199 35.81582415 0 2.98699879 0 37.21161496 66.66666667 0 0 0 2.23 3.366666667 66.66666667 0 33.33333333 0

278 0 37.03272676 0 0 0 0 0 0 0 0 0 0 0 0 0 0 0 0 0 24.02545744 0 0 0 0 0 0 4.994832931 0 0 0 0 0 0 21.97896544 0 2.533103051 0 0 0 0 0 0 0 0 0 0 0 0 0 0 0 0 0 0 0 0 0 10.40150011 0 0 0 0 0 0 18.90598291 0 21.12188366

279 0 0 33.33333333 2.876834321 0 0 0 0 0 0 0 0 0 0 15.64971751 0 0 0 0 3.143766732 0 0 0 0 33.33333333 0 0 0 0 0 0 2.490737684 0 0 16.6454892 0 0 0 0 26.22100122 0 0 0 1.487955393 0 0 0 33.33333333 0 4.334034156 0 0 27.77777778 0 0 0 0 0 1.740601958 0 0 0 0 0 0 0 33.33333333

280 0 0 0 0 0 0 0 0 8.712855297 5.223310412 10.93101474 0 4.809004093 0 0 0 0 0 0 0 13.38464358 30.02836798 20.05009299 6.578268164 0 0 0 7.954507775 0 13.39833075 12.93532338 0 8.388447972 0 0 0 14.2772637 14.35179237 0 0 12.22540489 0 8.50740215 0 0 0 0 0 0 0 0 0 0 9.823677582 0 19.02651782 3.187231786 0 0 7.618274234 0 6.9 7.963333333 0 0 0 0

281 23.08449351 0 0 14.26285325 6.123267688 26.14830071 30.91016548 7.82142154 0 5.9243506 12.20279397 0 0 0 0 0 0 0 5.884605565 10.75214538 8.78189203 8.992535665 0 19.88655536 0 0 13.01694915 0 12.51786565 7.834414581 0 0 0 0 0 15.82176624 36.54058923 0 0 0 33.33333333 0 0 0 0 13.31504537 3.937555303 0 0 0 0 0 0 0 0 0 0 0 6.617986487 0 0 2.13 0 0 0 5.987277036 0

282 0 15.88806139 16.06465122 17.90716999 0 0 0 17.39380573 11.86994179 30.04608997 0 56.56198447 18.42491256 27.70952299 0 19.24130968 0 0 16.39886183 29.06557665 12.53825785 0 16.45103324 0 88.64065138 0 28.45459373 0 0 16.64945712 15.51565847 9.622182943 8.852459016 0 33.33333333 31.94321016 0 0 18.36104812 0 0 47.8364815 0 0 0 0 15.88434924 0 21.8433267 0 0 30.2805193 0 0 23.2131126 0 0 0 0 0 13.60666667 8.14 11.42 0 0 0 39.30321391

283 33.33333333 18.9139997 63.73148887 0 62.00175421 33.33333333 33.33333333 14.57313038 33.33333333 0 0 33.33333333 0 26.37404241 82.27945404 48.32564497 66.66666667 34.38205798 0 33.33333333 0 0 18.74929355 0 0 33.33333333 16.47109442 18.9051059 61.0851862 0 33.33333333 0 43.74632569 66.66666667 33.33333333 0 0 63.49411427 54.10548087 66.66666667 8.404184598 0 8.084905023 82.37587192 54.79216784 77.30107471 56.15598534 66.66666667 61.25318066 0 33.33333333 60.63680482 77.52922453 33.58023238 48.91400009 43.88703607 13.22048295 8.042076688 42.89311343 0 28.31666667 17.93333333 10 72.26112018 0 43.31751113 30.44803414

284 0 23.38019369 0 10.16343207 18.71261853 0 0 25.51191179 24.62047804 55.51900565 49.0782344 0 60.0126767 0 0 0 33.33333333 33.33333333 33.33333333 0 24.5514413 24.34079767 0 35.25113473 0 0 0 23.24792126 8.916944577 45.43392134 20.39800995 0 0 33.33333333 0 0 0 0 0 0 0 0 33.33333333 0 0 0 0 0 0 33.33333333 0 0 0 0 0 0 0 33.33333333 33.33333333 53.94748714 0 12.02333333 22.92666667 0 66.66666667 27.3460563 0

285 43.58217316 25.64479064 17.26868211 39.71688034 0 40.51836596 33.33333333 27.28300874 21.46339155 0 22.40231859 0 14.90842078 32.06449415 0 9.623270118 0 24.28047404 44.38319927 22.58118795 40.74376524 36.63829868 25.98108026 26.75506517 0 66.66666667 34.35455988 39.80705299 0 16.68387622 17.81767486 90.37781706 24.48087432 0 29.42582746 45.34720308 42.27327462 18.98154097 27.53347101 33.33333333 22.58085809 50.0188984 50.07435949 0 26.82219197 0 7.087134735 33.33333333 6.393861893 62.09577792 66.66666667 0 15.38774472 46.18436355 0 26.19433928 83.59228526 58.62458998 13.70179193 33.33333333 16.00666667 19.17333333 17.58666667 15.6473606 33.33333333 0 16.9978995

286 0 0 0 0 0 0 0 0 0 0 5.385638298 0 0 0 0 8.971585172 0 3.074019145 0 0 0 0 0 0 0 0 0 0 0 0 0 0 0 0 0 0 0 0 0 0 18.22189566 0 0 0 0 0 0 0 5.413486005 0 0 0 0 0 0 0 0 0 0 0 0 0 0 0 0 0 0

287 0 0 0 8.907048008 8.497447119 0 0 0 0 0 0 7.672601511 0 0 17.72054596 0 0 0 0 0 0 0 0 0 11.35934862 0 0 10.08541207 0 0 0 0 14.531893 0 0 0 0 0 0 0 0 0 0 10.08062072 0 0 4.028383798 0 5.096144738 0 0 0 7.083030743 8.748099676 21.09724173 0 0 0 0 0 0 0 0 0 0 0 0

288 0 6.21981494 0 0 0 0 0 0 0 0 0 0 0 0 0 0 0 0 0 0 0 0 0 0 0 0 0 0 11.89852311 0 0 0 0 0 3.907505874 0 0 0 0 0 0 2.144620105 0 0 0 9.383879918 0 0 0 0 0 0 0 0 0 9.509921656 0 0 0 0 5.61 0 2.713333333 12.09151922 0 11.35971659 2.885299197

289 0 9.953139644 2.935177796 9.042616334 4.664912452 0 2.423167849 7.416721818 0 3.287243363 0 2.43208069 1.844985876 13.85194045 0 13.83819006 0 4.9301155 0 4.267756681 0 0 18.76849996 11.52897657 0 0 7.702802809 0 5.581480465 0 0 0 0 0 0 6.887820513 6.908872447 3.172552397 0 0 5.234323432 0 0 7.543507363 18.38564018 0 12.90659158 0 0 4.570888744 0 9.082675879 0 1.663626817 6.775645582 1.382185169 0 0 3.453774812 5.10090529 3.006666667 0 0 0 0 11.98943894 10.36555326

290 0 1.87907786 0 0 0 0 0 14.7723723 19.5026824 7.285791548 7.629137122 0 0 0 0 0 7.495006058 0 0 4.779756326 0 0 0 2.931266846 14.26564695 1.587941048 0 0 10.64848266 0 0 7.111890496 0 3.820750169 0 11.68894289 0 0 2.428718768 6.094621101 0 0 0 0 0 0 7.364064507 8.224637681 7.269632871 2.691690728 6.144574017 0 0 0 0 0 5.947668244 7.816524668 0 10.39313794 0 0 0.93 0 0 0 0

291 15.28171606 0 1.251780752 0 10.93256815 20.99216596 6.837160752 0 0 2.335902826 1.767765106 0 0 5.20592156 0 0 0 3.016949153 15.68102707 0 0 18.15106149 0 0 0 0 2.496556474 2.537144526 0 20.00286085 0 0 0 0 17.25873122 4.391111111 7.240583029 1.152698671 7.457180501 0 0 6.679028084 0 4.259776536 0 0 0 0 0 0 0 3.943081322 0 15.0594339 0 0 3.166732258 0 2.793946449 0 0 4.286666667 0 4.013125804 0 0 10.1886209

292 0 0 6.054598769 11.28375033 3.523793842 0 0 0 0 0 0 5.548824059 30.71800786 0 0 0 0 0 9.686472616 0 0.694719254 0 0 0 0 10.02436366 0 0 17.76802616 0 9.15 2.275047721 9.979949218 6.577365619 0 0 0 2.477503351 0 5.502159075 13.2066373 11.09832355 0 3.560682046 0 0 0 10.44116174 4.051510649 0 0 0 6.466711659 0 0 0 0 0 2.071326046 0 0 3.583333333 0 0 6.53535496 5.640725571 2.787037037

293 0 0 0 14.1253021 1.523311685 7.824019025 0 0 0 9.645435931 0 6.64616436 0 0 0 0 2.749973934 0 0 0 10.59112325 8.828945943 11.53677758 8.472316309 0 3.497248869 8.681903101 0 0 1.880434783 0 0 0 0 0 0 2.036109123 0 0 0 0 8.21493881 11.67914439 0 7.069344617 16.62982735 0 0 4.562751555 15.65069973 2.271673621 0 0 0 0 12.86650016 0 3.526785714 0 0 0 10.31333333 2.963333333 2.835754439 0 0 0

294 0 0 0 15.00114469 0 0 0 0 0 6.940688661 3.922666107 0 0 0 0 0 7.730257562 0 0 0 0 0 0 5.615453729 17.33610157 0 0 0 0 10.27317793 0 4.018705246 0 4.196798766 0 0 0 4.078519121 20.97241567 7.102862289 11.13161888 0 10.25609706 0 0 0 15.00867553 0 0 0 0 4.350877193 1.576004067 0 0 0 6.027699087 6.398725876 0 0 0 0 0 0 0 0 0

295 4.358820789 10.6673472 6.229488983 0 4.295369344 6.016646849 11.84044387 0 0 0 2.464060399 18.02647413 0 0 0 0 0 0 0 1.783838107 5.129529219 0 6.553996278 0 11.11284533 6.661537475 0 4.158383035 44.46102265 0 12.68793877 0 0 0 0 21.64439044 8.771458273 9.739041988 0 5.103774899 0 0 0 0 0 0 0 0 11.22429552 0 0 0 0 3.669415112 0 0 0 7.407040486 0 0 9.536666667 0 3.136666667 0 7.620589382 0 0

296 0 10.47411918 0 0 0 0 0 0 0 0 0 0 0 0 0 12.99310054 0 0 0 0 0 0 0 0 0 0 0 0 0 6.46178093 0 33.33333333 0 0 0 0 0 0 0 0 0 0 0 0 0 0 0 0 0 0 0 0 0 7.071068208 0 0 0 0 0 0 0 0 0 0 0 0 0

297 33.33333333 0 0 19.20803123 52.21030468 48.04124803 0 44.86014202 0 19.10685313 0 0 9.477846261 38.67707426 18.60692036 33.33333333 0 0 12.16139486 0 22.74221008 15.18227184 0 0 41.28817439 0 30.83677686 86.67869213 0 31.45289855 24.18333333 31.05828561 27.71275299 0 34.83021694 33.33333333 53.41450765 23.59429134 0 0 29.22335195 48.88931503 21.65418895 29.0735568 28.20048309 83.37017265 0 25.10869565 0 40.00555285 0 29.39025201 0 0 66.66666667 33.33333333 0 18.10976818 0 0 8.236666667 0 0 33.33333333 30.67345784 43.31045952 0

298 22.09023757 55.99931947 27.27873456 22.049583 0 17.12592014 54.8262228 24.03003278 80.4973176 54.68532791 27.06070948 54.47167825 21.53779573 33.33333333 0 33.33333333 49.61256383 0 23.64686072 33.33333333 60.84241819 24.50438739 55.12988908 29.91657686 0 31.74539229 57.98476357 0 11.43424599 29.92884695 20.64539456 0 28.97396446 33.33333333 27.09270652 28.94222222 0 58.95794552 38.2370705 22.06037825 22.20171445 25.11839452 23.07723627 0 33.33333333 0 15.3465582 30.28144239 72.8918094 24.17388515 66.66666667 33.33333333 0 47.93781766 0 53.80016651 30.16660108 29.80654762 30.53938688 33.33333333 0 54.34333333 0 0 55.17059782 33.33333333 56.47804576

299 24.93589224 20.98013629 59.18539693 18.33218864 27.5146523 0 26.49617258 16.33745291 0 0 57.15566178 15.30685921 38.26635015 22.78367085 81.39307964 20.34023279 32.41219861 63.64971751 38.82424473 60.10307223 0 0 26.77933706 53.06438626 15.99723176 46.48351666 0 6.625780309 15.68822254 0 33.33333333 22.20273759 33.33333333 52.07175211 20.81834532 0 28.53734192 0 30.90461457 54.13620439 24.23667741 0 33.33333333 63.10598462 31.39683896 0 62.28070175 25.94406253 0 17.47817155 24.9170857 28.98245614 91.95728427 26.26226513 33.33333333 0 54.69129934 26.93460746 64.59534062 56.27352873 59.78333333 40.36333333 90.3 59.81778642 0 17.71548158 30.5462963

300 0 0 7.540470602 0 4.058779268 0 0 0 1.234195511 0 0 0 0 0 0 0 0 24.59227044 0 0 0 50.14513332 0 0 0 0 0 0 22.90692047 0 0 0 0 0 0 17.30502507 0 0 0 0 5.094224924 0 0 0 0 0 0 0 0 0 0 0 0 0 0 0 0 0 0 0 0 0 0 0 0 26.63457682 0

301 8.413595757 33.20002458 0 16.6353893 0 0 0 28.20570571 0 0 0 0 33.33333333 0 0 13.33652389 0 0 0 0 17.42241518 4.074186683 0 26.16659981 0 8.981204613 15.60295506 0 0 54.40313235 0 30.64484127 0 19.59046245 0 0 0 0 10.76006671 0 0 30.38560509 0 0 0 0 13.8560095 0 0 0 0 21.83597271 0 14.38286164 0 30.93794803 0 0 0 0 0 0 0 16.39645151 6.787597745 0 52.57343835

302 7.965003977 0 0 12.34961589 7.887398752 0 12.60480452 15.61039895 15.81204788 22.14249058 15.30412786 0 0 5.530946965 0 0 0 0 0 0 0 0 0 0 0 0 0 25.47326004 0 0 0 0 0 0 0 12.57604516 0 0 0 8.175690367 8.461415306 11.43218271 0 0 0 14.67220957 0 12.98848711 0 0 0 0 0 0 0 0 0 0 0 0 0 0 0 0 0 0 0

303 5.573003908 0 0 0 0 34.8883586 0 0 6.329336651 0 0 14.22087133 2.1631644 0 11.98125837 0 0 0 11.77209229 0 0 0 12.1254098 33.33333333 0 0 12.9425734 0 38.44168414 0 23.03014669 0 0 0 33.67727809 0 8.046148491 0 0 32.17571634 0 0 17.4749179 0 0 0 8.419775554 7.849657396 22.04065976 0 0 18.66148926 0 0 0 0 0 0 0 9.262701363 0 3.53 0 0 0 12.97883505 0

304 0 18.1372549 6.46025155 33.33333333 24.46071753 0 0 0 0 0 0 6.707677748 0 26.08076393 16.50568182 0 8.631820996 0 16.1026276 14.5694777 0 0 0 0 0 0 15.09014571 0 23.17391696 0 0 0 4.769230769 0 0 8.702300935 7.655920613 7.451029011 35.52836254 0 17.53596758 0 0 0 33.33333333 0 0 0 13.35324232 33.33333333 13.75485302 0 0 0 0 10.32823509 17.24283305 15.6522376 9.233467451 0 0 0 0 0 2.936897618 0 14.49187607

305 0 10.64230343 16.15320626 0 0 0 0 0 0 12.97428433 23.13684628 0 33.33333333 0 0 48.30956865 33.33333333 0 8.882333661 0 0 15.40732665 7.274283269 23.70947465 0 15.79097252 17.73037827 0 5.318062053 3.813588024 33.33333333 24.72109061 0 13.1850307 12.24637681 15.3761506 0 0 0 0 33.33333333 0 0 46.86325528 0 0 33.19412 10.57240149 0 15.96491228 0 13.78535085 0 0 20.05509642 0 0 33.33333333 0 0 2.47 17.98 0 9.115937596 33.33333333 40.03208985 0

306 33.33333333 0 0 7.43567288 33.33333333 0 33.33333333 17.72293439 0 0 33.33333333 0 0 0 0 0 0 0 0 0 0 0 33.33333333 0 33.33333333 0 0 22.17138922 0 0 0 2.688492063 21.46054841 0 0 0 49.4554677 32.83702078 0 25.15764297 24.87191803 21.90115063 0 0 44.58408139 0 0 20.25281671 19.98009101 33.33333333 0 0 0 50.26552288 0 0 33.33333333 0 0 33.33333333 0 0 0 33.33333333 0 0 14.09322832

307 0 0 0 0 0 0 0 0 25.76980117 19.07051282 18.02920547 19.11246201 0 21.07404394 0 0 0 0 17.23070574 52.09718897 15.91091815 0 9.608902433 16.79059221 0 0 20.39075993 19.02201741 0 16.41915917 0 0 33.33333333 13.74287089 20.28566421 7.482760586 25.67741272 25.88230432 0 0 0 22.67429074 0 0 0 33.33333333 0 0 44.6260069 0 33.33333333 11.49736063 46.63947164 18.9504717 33.33333333 52.83360492 0 33.33333333 0 33.33333333 6.93 0 0 0 0 0 18.84145726

308 44.71506302 15.19607843 40.53581974 20.98371745 21.38715531 14.69078779 0 33.33333333 0 33.33333333 0 33.33333333 0 27.80238637 71.51305982 38.35390746 58.03484567 0 24.45099967 33.33333333 33.33333333 0 26.05905006 0 66.66666667 41.89448953 18.24318762 33.33333333 0 14.01815349 0 0 40.43688749 20.14830263 21.08695652 20.21840038 0 0 26.72615254 23.14515162 0 13.60677083 49.19174877 33.33333333 0 0 28.10737211 35.25612067 0 0 33.33333333 34.21982656 33.33333333 0 0 5.900211949 49.42383361 17.68109573 33.33333333 0 20.68 19.84333333 6.923333333 0 56.9421713 0 0

309 0 22.82433865 29.31025185 9.262271154 8.872615804 50.42085361 54.06186214 5.127627628 50.85461878 12.47937894 10.19648705 26.62565559 31.17016893 19.5118588 0 0 0 42.07439623 21.56124104 0 33.33333333 30.37335335 11.5990211 0 0 33.33333333 0 0 10.15941637 11.34596696 43.63651998 41.94557606 0 33.33333333 12.70372436 18.33931726 9.165050475 33.82964589 26.98541821 11.34579871 10.70314083 0 33.33333333 19.80341139 22.08258528 51.99445709 16.42272284 13.08051663 0 17.36842105 19.57848031 0 20.02719503 16.40114379 46.61157025 0 0 0 57.43319922 24.07063197 5.403333333 0 5.586666667 41.15427756 0 20.35449828 0

310 0 0 0 0 13.33406982 0 0 0 0 13.84068509 33.45228548 0 0 0 0 0 15.62099872 0 0 0 0 20.92635788 0 0 0 0 17.20143592 0 21.21915208 0 0 0 0 0 0 0 15.511735 0 0 0 0 0 0 0 0 0 0 0 30.54097994 24.79714912 0 7.039312533 0 0 0 0 0 0 0 0 0 0 0 0 0 0 13.75178036

311 28.11834205 0 0 9.615445987 0 16.8271892 0 13.31666485 0 0 7.515955361 0 57.66304749 0 0 6.977186312 0 29.35294347 0 0 6.93708875 0 15.77824022 61.42476997 44.23213739 22.44333261 0 18.49649504 16.41867624 2.955861793 0 41.33673904 19.73283337 9.881355353 9.161484831 0 0 0 0 34.67839002 33.33333333 8.713166076 0 8.671711579 20.75321568 0 11.78451178 18.0665396 0 0 0 0 0 0 0 12.13657373 0 0 0 24.70999961 6.803333333 13.82 6.436666667 0 25.41648023 19.09941363 0

312 40.50879053 0 0 20.68448011 7.732854148 0 19.2352208 58.48814378 0 23.68504061 0 61.20178938 9.952816278 0 13.23521818 0 15.11189795 0 0 9.008478568 16.31914456 16.03509427 0 0 0 33.33333333 15.98330378 0 33.33333333 0 19.97910891 0 0 37.810493 0 44.56545114 9.247603768 0 20.26090722 0 0 37.33464039 5.385984352 33.33333333 0 0 0 0 0 25.12134552 0 13.80260521 15.33939394 33.33333333 4.62458154 0 46.56363284 33.33333333 0 12.68955409 25.48333333 2.78 12.78333333 0 0 13.64151371 16.20013055

313 0 54.62191358 42.00201253 33.33333333 24.41015883 16.75374759 31.84453592 0 42.08494208 0 8.869914616 33.33333333 0 57.94009447 40.65087129 15.8271262 0 41.05969658 20.87751104 21.6055996 36.62732521 33.33333333 11.71953676 0 19.1150272 4.754346183 31.09107432 16.18852459 0 0 18.10047982 25.17593951 35.16985849 6.906691285 26.54162206 20.09731708 23.32637439 32.17162508 18.60661597 19.83094451 31.83702599 7.154428828 33.33333333 0 31.7703891 57.44680851 9.6482062 10.39439297 46.66843096 21.55336163 43.25567002 45.43227724 43.07094638 0 35.8089685 47.2303948 0 37.72231467 79.3361397 33.33333333 27.77 29.02 16.29333333 83.62839691 50.97465887 33.33333333 0

314 0 0 0 0 0 14.90605947 0 0 0 0 0 0 0 0 0 17.10392902 0 0 33.33333333 0 0 12.40697545 15.23284314 0 0 0 0 0 0 68.89299466 0 0 0 0 14.8746938 0 0 9.799622389 33.33333333 0 0 0 21.63014347 6.410729667 11.55147942 18.29084354 0 50.04229293 0 0 23.27837515 0 0 33.33333333 0 0 19.91527849 0 0 4.781565806 0 0 7.88 0 0 0 35.12742828

315 0 13.65676689 33.33333333 14.88201262 14.97708524 0 35.74622368 0 24.58172458 23.719097 9.476949406 0 0 0 20.09811516 20.63657007 33.33333333 0 0 45.06106707 0 0 0 20.69762777 18.77279014 27.34880646 0 33.33333333 12.11418126 0 25.30494566 0 13.60049996 20.44861338 0 0 25.02365379 9.467223352 0 16.90128433 0 46.79776471 10.31865955 33.33333333 8.0692067 0 0 0 7.51459144 20.31615591 0 16.04458918 36.46292424 23.72805245 12.21903396 0 0 0 0 0 0 0 0 0 15.10274713 16.11148878 0

316 5.214991284 0 0 0 13.94535278 2.358020591 13.17401961 20.01666848 0 0 24.34446657 0 23.38051705 10.43297746 0 0 26.52885566 3.980389868 16.34227858 0 17.01418877 0 17.55509311 0 0 0 27.73068716 0 6.895844023 0 0 21.33676093 7.07201889 0 19.24053368 0 0 0 0 0 0 0 0 0 0 9.219858156 45.23394868 0 0 0 4.479283315 0 0 0 0 24.18560849 0 0 20.6638603 0 0 9.766666667 0 0 0 14.2339197 0

317 0 0 7.774469493 0 0 0 0 0 0 17.15988183 0 0 9.003619174 0 10.33931224 17.50620713 0 0 0 24.32485477 6.616134908 0 0 0 0 0 0 14.83683829 0 0 15.23285351 0 0 0 0 0 0 5.914946669 27.79914347 0 0 0 0 0 15.72769953 0 33.33333333 0 0 8.211987809 6.377277599 0 0 0 0 8.36936534 0 0 0 3.841767921 0 0 6.62 7.24450194 8.506113769 0 34.92066081

318 0 19.67656644 16.89018465 0 25.60047919 0 0 8.178522882 0 9.648292721 0 0 0 0 0 9.252217997 0 25.60697009 24.16328322 0 16.4861178 0 18.1004902 0 0 12.12018141 0 0 0 14.33421781 8.028387669 12.15056052 0 24.95284698 20.88451092 13.23601625 10.00695894 0 0 13.50238883 34.82964067 0 0 0 12.12800957 0 0 14.05321929 5.281290532 0 0 14.1950769 0 0 16.18561539 0 20.98843572 0 0 0 0 0 0 0 0 0 0

319 26.15787614 12.04475309 0 21.48472795 0 49.15498315 0 0 33.33333333 11.94700275 16.34042857 5.464877282 0 31.62692807 15.67648314 12.69676327 9.404914334 0 5.283593827 0 0 17.29823906 21.61379658 17.87760226 17.88004527 0 7.993498818 17.14480874 10.01881307 13.81692573 13.35422442 0 24.42478928 0 9.297154701 22.10121553 16.88367411 42.64658251 0 15.08699231 0 0 29.3318793 18.25089209 0 15.04248979 0 7.443555209 9.994707128 0 22.60939392 3.486138945 5.126735448 9.605280884 31.16180061 8.078057636 12.53265294 28.94435199 0 20.64377924 18.56666667 14.77666667 13.83333333 9.12710115 0 3.580330841 0

320 0 0 0 24.2282697 11.48869684 0 32.0144421 0 0 0 0 16.85254027 0 0 8.019822006 0 0 0 15.80007263 35.00349174 10.74846861 17.10059425 12.08266316 0 0 22.27378003 0 0 0 17.82416836 0 0 17.88440567 0 15.69378931 0 0 0 0 0 13.56804809 13.90728477 24.23859103 0 0 0 14.84281484 0 0 5.016664502 18.47182765 18.21533923 17.11839615 21.43043592 0 13.42353952 0 19.12183483 11.76928483 0 0 6.873333333 3.86 0 7.972937119 0 10.16289593

321 25.89505329 20.89272708 32.87213429 0 6.342592593 0 21.5751181 20.36803432 35.96402076 0 17.32085608 7.446544008 18.48455375 0 6.054957194 0 25.05141325 24.96891775 0 0 0 20.11976718 0 25.27643125 10.70621746 0 23.78307896 0 31.03147869 10.86937138 16.99555688 8.039963669 0 12.62347699 0 0 0 8.366013072 14.83500381 0 0 5.95982064 0 33.33333333 22.29234353 16.9739401 0 22.62126287 14.10111452 38.14927232 0 19.01029892 0 17.66134857 0 0 9.276059989 0 9.287925697 0 9.89 9.766666667 11.25666667 23.9247331 10.16548463 16.63042088 20.5109127

322 0 6.731616028 0 0 0 0 0 10.56485356 0 0 0 33.33333333 0 8.980454305 0 0 0 0 19.47438079 0 0 6.733358984 0 33.33333333 0 21.08737398 7.522731172 0 0 0 22.41666616 0 0 14.31976102 0 0 15.6946132 0 0 10.73064504 0 11.44270833 0 0 0 15.26518704 7.484682895 9.402040553 18.25291706 0 0 0 21.38124698 0 0 0 22.4841259 0 0 0 0 0 0 0 0 0 0

323 20.54400603 33.33333333 0 12.63436672 20.56669054 14.84625781 0 15.72788267 0 38.77975312 0 0 26.29969982 41.14439748 28.03412485 26.99437716 0 9.063539851 0 7.084152675 0 0 21.49759262 5.420679508 42.61560201 14.40497077 0 21.46326179 18.4624086 8.902847571 0 33.93002846 14.72737561 0 21.97246485 42.92185314 16.37530427 7.406045752 26.56981821 18.77681897 36.37049842 18.56088691 12.83671433 14.4679364 25.52826846 15.90219041 0 0 7.403219728 13.6129507 35.81741025 13.9516129 0 41.8751231 31.28892071 41.33573704 8.088200124 42.60178117 29.260323 57.54303292 6.416666667 5.32 5.26 11.1915407 35.48264609 38.65176332 9.352943663

324 16.16487918 15.18604139 26.48223063 33.33333333 0 0 18.47679135 0 0 0 18.1751084 0 0 0 5.475006904 33.33333333 0 0 0 0 36.05949237 0 0 0 9.468618014 0 0 17.64056358 0 0 0 16.98978058 0 0 0 0 19.68867255 17.3235812 18.49832952 33.33333333 0 19.42604857 0 0 0 0 34.61657755 15.17500587 18.51127624 0 0 0 0 0 0 0 24.05727334 0 10.85995921 0 8.023333333 0 0 21.05682229 0 0 9.56561086

325 0 0 16.48532575 0 0 14.1386755 0 10.81200842 23.70704627 29.88635118 16.44183463 6.089942173 0 24.35287903 0 0 0 36.99512739 0 14.36174671 22.58486473 35.5506617 0 0 0 0 5.961497173 0 0 9.830278344 0 0 11.05052708 10.66030974 6.962877476 23.66723047 10.88159849 33.33333333 33.33333333 0 0 0 20.496619 45.7102918 6.837905527 0 0 0 0 13.87055567 6.220407197 0 27.50603766 0 15.39108141 24.8087997 0 0 0 18.7745154 11.41666667 8.893333333 14.19666667 0 0 0 12.82242063

326 0 0 0 0 19.51809764 13.24705387 11.79408026 11.39818689 0 0 0 25.88678933 35.42118663 25.52226919 14.072469 0 0 0 45.4008556 43.55060887 0 0 49.15923381 0 0 0 25.81060216 0 26.26257364 0 20.15361496 0 0 29.21695046 0 15.43947725 16.95802906 17.56127451 6.763515123 0 5.6568176 0 0 0 6.344841634 11.47413458 0 0 0 0 8.367526377 33.70475484 0 0 22.87432443 0 26.23639833 15.85877863 11.74428583 4.980692454 6.073333333 33.89 4.266666667 23.58076687 23.1678487 37.05502957 0

327 29.95778145 23.85628216 0 29.80403024 28.2686867 0 0 10.57887346 0 12.0276688 0 10.39085089 0 0 16.54025081 0 37.50838358 0 0 0 30.6071743 0 17.26051042 21.73622258 37.20956251 12.24595935 17.5227728 60.89617463 14.87092474 5.678886626 0 15.05453103 26.67378917 0 39.51781971 10.03455174 6.757121642 0 0 37.15920265 0 11.9375 42.42807564 0 29.79000065 35.49928921 43.05592471 23.93129278 4.774519079 10.5804133 19.71212121 0 17.77938202 7.130194997 0 0 0 14.2114985 17.52209381 0 3.86 0 12.20333333 7.969626002 23.21108345 0 0

328 7.43828004 0 9.529557724 0 0 37.68173335 4.381352962 0 27.02274672 19.3062269 48.06220089 0 19.7945598 0 21.80336924 39.67228951 24.48109021 17.86482509 0 0 0 17.84003248 0 0 0 16.20063868 0 0 0 24.43048576 24.09638554 16.34355275 0 23.12995519 0 0 0 16.00975213 0 0 15.34234028 0 0 0 0 0 0 18.15832746 0 0 0 15.1179941 16.21493719 11.90289742 0 20.43192375 0 0 0 0 0 0 0 12.27651104 0 0 0

329 0 0 14.6307516 0 13.81523569 20.08627946 11.75821523 20.55016069 13.30618625 0 0 0 0 0 0 0 12.95911296 11.10758992 19.32469099 0 0 2.655585404 0 14.23333333 0 13.78727718 19.39931773 0 9.372614338 22.46396196 16.33777645 9.642143506 29.66390247 10.0495466 15.85304866 7.936887401 13.64466078 0 0 0 29.0622956 18.76575078 0 6.488438465 9.206640187 4.885258654 0 10.71207046 36.95695338 18.77014351 11.41070731 0 0 0 30.44567346 0 9.857942316 8.20610687 9.556127623 18.70175922 0 0 0 0 0 7.662786235 37.58521622

330 5.430283224 16.17878502 4.993782975 20.73392073 22.1733392 0 0 10.25395304 0 0 0 0 0 13.96179254 17.7040677 0 0 0 18.82725205 12.08883026 18.01393504 9.563033651 0 0 0 0 0 0 16.02620489 11.67822291 0 11.83018868 0 0 0 0 18.31354935 0 32.09528942 0 0 0 5.439814815 0 0 0 13.70504465 0 0 0 0 0 15.08426042 0 0 14.9536188 14.68611469 0 0 0 17.12 8.39 13.99333333 33.33333333 24.60739445 0 23.34788937

331 9.180020457 14.20259057 0 2.942357903 0 0 42.061454 0 0 0 0 0 12.27113906 17.40291262 24.34833261 14.84902409 6.620977115 26.05876755 8.375016032 36.18534181 8.728271781 13.6477852 29.48898991 0 0 0 10.93683038 13.68497676 0 4.146671589 0 4.656666667 33.33333333 0 0 8.741403596 0 9.973573902 33.33333333 36.7946664 0 9.888899686 28.0799733 31.50727071 11.94000968 0 0 0 21.6062424 0 25.11227434 50.52834729 0 17.15059081 14.6484546 24.6658734 13.40806682 24.66223754 11.54981031 47.62202182 12.30666667 10.61 0 0 6.935612083 5.800235942 0

332 0 3.494421124 0 9.791292718 0 0 0 0 0 0 0 0 9.638361766 0 0 0 0 0 0 0 0 5.890379804 0 46.43478613 44.75428964 2.904981164 0 11.33744011 0 0 14.45188706 0 11.75048853 0 0 0 0 0 0 0 19.03581669 0 0 0 0 13.16291264 0 0 0 30.42999409 0 0 19.16786917 33.33333333 0 0 33.33333333 0 0 0 0 0 0 13.45437101 0 12.57706535 0

333 0 0 0 12.5994126 0 7.530834915 33.33333333 0 0 14.09662868 15.02890173 33.33333333 0 0 32.6171604 33.08457942 8.353260003 0 0 0 0 0 0 0 0 0 4.91572242 0 8.741390697 0 12.76265689 0 0 0 6.732819419 0 0 0 0 13.48815606 2.945723818 0 0 0 17.63642581 0 0 7.116465863 33.33333333 33.33333333 23.24437256 0 0 0 11.31413806 19.14643696 0 21.91176471 9.865307608 0 2.873333333 0 3.87 18.01263445 0 0 0

334 0 0 0 0 11.15999413 21.363355 0 7.546717777 0 12.78805316 20.82268053 0 0 0 0 0 0 33.33333333 14.50608128 0 12.5165108 11.88012724 6.1960475 0 0 24.61646666 0 33.33333333 4.359666024 0 0 21.50314465 33.33333333 5.956168257 22.47284879 0 0 0 0 15.54346309 0 21.58302766 0 0 0 33.33333333 10.04973799 0 6.362929418 0 8.596112311 10.54400662 0 17.13251809 27.40361152 0 0 0 0 13.98372586 4.863333333 0 0 0 0 0 0

335 0 36.28529108 0 8.385217915 45.4180724 17.8197065 0 18.40124004 28.06699647 47.79202279 51.63776493 0 54.3955276 15.93042071 0 13.53141681 33.33333333 0 33.33333333 7.805760401 26.25442206 5.786488621 13.59818388 17.85464148 17.11421411 0 22.39650296 19.64835657 0 9.330609068 18.27711759 0 0 0 13.09546049 6.181125197 24.07661307 56.69309276 0 0 25.63826579 17.42526479 13.51560086 7.139388052 15.23487773 0 33.33333333 33.33333333 0 15.9650082 0 4.319711144 0 0 0 0 8.314100451 0 0 0 0 13.30666667 0 19.87896232 0 6.265565605 33.33333333

336 0 0 44.84323016 0 0 0 12.06647186 0 0 0 0 0 0 33.33333333 8.985000728 0 24.98007333 22.779965 0 0 0 0 11.00777346 0 0 35.61860435 0 0 16.47325219 21.65511043 6.496497908 33.33333333 14.028592 16.41922941 0 10.25793356 17.65097765 0 0 0 0 0 25.0710925 19.72687265 0 20.17042069 7.091479604 0 0 20.27166437 0 0 0 0 0 0 18.64721865 11.42156863 23.46802573 0 0 0 9.703333333 0 0 13.97908389 0

337 40.09129561 0 0 0 0 11.96997833 0 48.86599585 0 20.54528018 0 33.33333333 23.69497157 19.3715408 16.34543856 18.37235877 0 0 0 43.92006754 34.48686031 0 0 0 38.13149625 0 0 0 0 29.18666174 18.88144627 28.67666667 0 55.96479036 10.86048454 33.33333333 0 22.19753478 19.50051525 19.84517727 6.327452981 14.41282632 0 15.43252331 0 11.90861302 0 25.20820475 19.61328095 0 32.95828002 16.9450431 51.93309512 0 40.70407401 0 11.61116606 0 55.11685635 11.81583275 0 20.23333333 20.69666667 0 68.45699347 0 0

338 24.15331288 0 50.16298686 45.54779813 0 25.80249842 0 14.9320933 33.33333333 0 12.5106528 33.33333333 0 0 0 6.624220207 26.71235622 7.274565786 24.9583173 0 0 53.23218549 0 0 0 8.716866675 28.41761091 0 54.39948619 0 14.07417854 0 0 0 46.83838676 18.41080454 15.01978398 0 15.070862 0 0 15.90806854 0 0 15.69690752 0 23.28359535 0 19.0842139 0 0 0 0 0 0 22.85435631 0 20.3505109 0 0 20.74666667 0 0 0 0 0 33.33333333

339 21.14508783 29.83891221 0 0 21.24859427 15.51362683 12.53874081 0 38.5996702 4.778015195 0 0 0 0 0 13.5384007 0 10.55336833 0 0 0 0 39.70900525 35.7105724 0 28.14308115 0 21.99589322 0 24.00272427 15.05621574 0 7.554252803 21.65981197 0 23.07539977 24.93907595 11.13579855 0 14.32853717 46.05274073 20.781913 27.89351852 26.19394528 39.49177926 21.42472031 12.53680908 34.34199605 0 0 10.08896078 17.66289184 13.81477529 32.38355776 5.929721816 18.37971454 0 21.65391823 0 26.57841957 0 8.833333333 0 15.32069888 0 61.37804921 9.985443959

340 0 0 17.53324753 21.59560665 27.42727441 11.31011244 47.38821606 15.00687176 0 0 0 30.16595455 19.03787103 0 34.68236694 10.17954342 33.50922758 0 0 0 47.2859783 0 0 0 31.10747918 0 0 42.72113118 21.1923833 11.91050052 0 30.70128159 20.47148954 0 0 0 0 18.28706933 23.16487737 0 0 0 0 0 0 13.50688376 0 33.33333333 19.69913661 18.27125791 0 36.92791182 0 14.7409556 0 0 34.43103954 10.62625394 33.33333333 19.75429574 0 3.71 0 22.4305835 16.66666667 0 0

341 0 48.55105725 0 16.89580729 24.36837816 0 0 37.81307624 23.46055164 0 38.90591038 16.74624226 0 73.25721517 17.12490633 0 0 33.33333333 19.06903257 40.22268344 26.84547666 0 19.66248901 46.22816415 16.67630336 0 77.44141937 0 0 33.33333333 21.48348742 26.58109631 26.72162949 0 0 0 0 23.71151484 52.25869195 47.84825902 0 14.66039368 0 49.61153814 18.57404631 21.31377377 28.85278972 0 0 46.79675072 51.00182864 0 11.4829067 0 42.26371747 18.63356846 6.91958704 0 31.45930322 25.8884127 29.81333333 18.72 29.22333333 30.72961465 18.67158672 7.323616852 22.889145

342 0 0 0 0 0 0 20.57453416 0 22.52553867 22.4137931 18.41333822 25.65399749 26.26467604 0 0 12.19135802 0 11.13288541 26.27203283 20.85402185 0 64.92293222 0 0 0 41.40701283 0 0 12.48366896 0 0 0 15.88277858 15.19497725 28.67742608 25.62379608 22.93853073 7.32369689 0 0 17.85020804 25.19118782 27.59112197 10.67979915 0 0 0 25.22522523 47.61946695 0 0 0 24.23105776 24.43020905 25.85861017 0 0 0 0 0 12.94333333 0 9.916666667 0 0 0 26.94004053

343 10.04898773 33.33333333 60.23353954 0 0 33.33333333 0 7.083201184 0 38.2444032 0 0 0 0 18.78803034 0 14.3002934 28.81328473 0 0 0 0 55.82636735 0 0 9.816908993 0 7.689743305 22.06792531 13.81457379 9.902185726 14.68859584 6.113424855 22.76751048 0 21.17771538 0 0 0 0 21.37888863 0 0 0 0 0 22.72499821 0 13.63419673 10.37852784 0 0 21.85042664 0 0 8.039242404 15.74560608 35.8133211 14.16499483 9.312185146 0 4.98 0 0 19.37433073 23.10667585 0

344 31.50893628 0 0 16.12310152 0 14.53320148 0 0 33.33333333 0 0 0 0 13.94332302 8.017753185 18.66114225 4.374352481 0 0 14.2791762 0 0 0 0 0 0 7.843927787 0 0 30.43719749 33.33333333 11.4379085 0 33.33333333 0 0 17.77708105 0 0 0 0 27.0525507 0 0 7.405809606 0 19.00566824 0 0 10.27953463 0 46.46929595 11.93917233 7.81564741 0 0 4.944349954 0 8.290048644 9.675774878 0 12.68 0 0 0 33.33333333 16.12776468

345 9.775870291 10.70106184 0 24.23451466 14.8710141 24.39885783 0 11.24326039 10.80779466 14.58691238 8.85074878 0 0 6.194927463 7.842531045 21.14197531 0 0 0 0 0 0 0 11.26683155 0 11.23855681 0 3.380503145 9.063169322 0 0 0 17.45055475 8.075571684 0 0 20.83150985 25.57340696 10.16845597 0 4.595469256 8.142145513 18.68660822 30.1954798 41.78466322 27.72533721 0 15.75986566 0 0 11.39978791 9.074733096 0 11.77542836 7.184669759 0 0 9.871765688 0 13.57903759 0 0 36.91333333 23.21251 12.56529096 0 21.9351505

346 0 0 6.593736594 0 0 0 0 0 0 0 0 3.167378785 33.33333333 2.848712992 0 0 37.97383345 0 25.25964198 0 0 0 0 21.0524224 25.64401899 0 0 8.352779939 0 0 15.16322505 0 6.748418942 0 15.20279799 0 0 0 0 18.81840765 7.47534969 0 5.742211362 0 0 12.01955957 0 0 0 0 0 0 0 25.15054195 0 0 16.24334655 0 0 4.95286548 8.093333333 0 0 0 13.9590026 14.29192952 0

347 0 0 0 0 0 0 32.03724978 0 0 0 0 0 14.2954623 0 8.190673814 33.33333333 9.842293082 22.20044793 15.13499186 16.35528048 25.86854504 15.17835403 0 21.45258191 16.65702997 0 10.67042607 0 0 7.608259018 0 5.907047958 0 0 33.33333333 0 25.95105488 0 14.40797471 13.16091954 0 0 33.33333333 0 0 16.73239437 4.480543611 0 13.92881658 4.147597254 32.87056443 0 0 0 7.474723158 48.03309821 21.71607084 15.66991191 0 0 0 4.85 0 6.85982562 0 0 2.793340486

348 0 0 15.63947633 0 33.33333333 7.490019412 0 0 0 10.91954023 25.65110735 24.26642691 7.068657293 0 5.353738342 0 0 0 14.26430076 8.288838037 0 8.05137412 24.51114364 0 0 0 4.044226774 4.522509103 20.84966437 0 20.11776847 10.6840698 6.611703847 20.62860725 22.78644259 45.48895129 0 5.826010136 0 0 10.88765603 6.54877785 0 9.513182908 25.92752373 0 24.93600022 0 0 5.34379085 4.727819017 7.528059129 30.49643658 16.08721762 17.21827943 25.29409093 0 23.46156765 5.665997933 16.83742846 3.916666667 12.87333333 0 11.33003158 14.66174662 21.94444444 0

349 48.6662057 7.414547569 0 21.15096987 0 8.934475503 0 28.85359043 9.872781698 13.83535108 8.178895271 0 0 3.755821356 0 4.492647656 0 4.520048603 0 0 0 11.84733963 0 0 9.915168496 37.53752137 0 33.33333333 14.34318873 2.896135841 0 0 0 0 0 7.709537249 12.50182349 19.27830184 0 20.17241379 37.81242834 18.40494443 14.64672512 0 6.307957133 8.702051329 0 25.68157578 5.118383131 4.782540798 0 0 0 0 0 0 0 4.557179708 7.086322038 0 0 4.77 0 5.437434646 4.101375708 0 9.314558808

350 0 11.09634748 4.530868951 6.185630589 3.171007928 2.977544474 0 0 0 0 0 0 0 9.543093222 0 9.657633545 0 11.90161154 0 4.223208072 0 0 0 0 0 0 19.2342513 0 0 0 4.809920052 0 0 8.828696327 0 0 0 0 11.88101255 0 0 13.48982384 0 0 0 0 0 8.088815677 10.56910569 2.548764629 0 14.88839286 0 0 0 0 0 0 0 0 0 7.636666667 0 0 0 0 0

351 0 0 8.069711008 0 10.25904517 0 1.416497634 0 20.34436333 0 0 13.92951477 3.212901097 0 19.63043576 4.680130054 0 0 0 0 4.954390363 0 7.100767307 0 11.5916955 0 0 5.442577856 0 11.46699267 0 5.981014688 5.870805918 6.70282959 0 0 0 0 0 8.420344882 9.760608971 0 0 12.01234397 9.169192284 0 5.493587333 10.76388889 0 0 3.38780581 10.95232377 2.347296071 0 22.30322821 0 7.353197445 0 0 0 11.33333333 0 0 0 17.69882384 0 0

352 0 0 0 0 7.676848875 1.956064947 6.37117036 0 7.328313377 0 0 0 13.32014005 0 0 0 0 0 0 0 0 0 0 0 0 0 9.013372644 7.740167634 9.26473305 0 0 0 0 0 0 7.315952202 0 0 0 8.699071889 0 9.811289245 0 0 0 0 0 0 0 0 0 0 0 0 0 0 0 0 0 0 0 0 0 0 0 16.44242309 9.844596521

353 0 0 7.593205383 0 0 0 0 0 0 0 0 0 0 0 0 0 0 0 0 3.690905449 2.131524767 0 0 0 0 0 0 0 0 0 0 0 0 0 0 0 0 20.94731906 0 0 0 0 0 0 0 9.402678084 12.20975857 0 4.915572233 0 0 0 0 0 0 0 0 0 0 0 0 4.92 0 13.32824427 0 12.38008913 0

354 0 0 0 0 0 0 0 0 0 0 0 0 0 0 0 0 0 23.26594789 0 0 0 33.33333333 0 0 0 0 23.03921569 0 11.93691336 0 0 0 0 24.50463701 0 0 33.33333333 23.64510662 7.691625659 12.23847727 22.7674105 0 0 17.1532069 0 0 0 0 25.63789773 0 0 0 0 16.78118797 14.08666903 0 0 0 0 0 0 11.62666667 0 0 0 22.17128977 0

355 92.97602358 88.90365252 69.72715235 93.81436941 74.30817747 92.2430859 92.21233201 93.37097154 62.80625583 92.39669421 95.57673771 86.07048523 83.46695885 90.45690678 57.17021166 75.58468916 90.45349731 49.971162 95.74451098 86.61441814 90.8216122 65.32599837 85.62889482 100 88.4083045 100 24.31996069 78.57418552 48.73452799 84.74153814 92.21435853 94.01898531 94.12919408 22.06703911 100 92.6840478 66.66666667 33.33333333 64.94497112 15.84796709 33.33333333 76.69888691 93.78218577 46.94855143 67.86038131 83.3888783 48.81307742 44.17622491 33.33333333 97.45123537 83.01837345 63.52576016 97.65270393 0 25.00351173 100 85.16446473 100 96.05403349 100 72.69 36.08 35.65333333 69.30235766 71.13959652 27.97427888 43.58634405

356 0 0 0 0 0 0 0 0 0 0 0 0 0 0 0 0 0 0 0 0 0 0 0 0 0 0 16.98002147 0 0 0 0 0 0 0 0 0 0 0 0 33.33333333 23.57272436 0 0 0 0 0 0 18.32938673 17.84865541 0 0 0 0 33.33333333 4.66280295 0 0 0 0 0 0 0 25.33333333 0 0 0 0

357 0 0 10.07906231 0 0 0 0 0 0 0 0 0 0 0 23.19935258 0 0 0 0 0 0 0 0 0 0 0 0 0 0 0 0 0 0 26.63050374 0 0 0 12.38601428 13.76069513 0 10.56592283 0 0 16.18012643 0 0 0 0 0 0 0 0 0 49.8854787 0 0 0 0 0 0 0 0 0 0 0 16.67022372 23.48873681

358 0 0 0 0 0 0 0 0 0 7.603305785 0 0 0 0 0 0 9.54650269 0 0 0 0 0 0 0 0 0 0 0 21.39641997 0 0 0 0 0 0 0 0 0 0 21.46080553 0 0 0 7.705771268 0 0 0 0 0 0 0 0 0 0 19.24666431 0 0 0 0 0 0 0 0 10.55470738 0 0 23.08032262

359 7.023976419 0 0 0 4.584920553 2.82330468 0 6.629028464 9.521067461 0 4.42326229 0 0 0 0 10.07754724 0 14.86127857 4.255489022 5.471468337 2.092472669 1.340668297 7.270337869 0 0 0 7.413178214 8.243068988 8.667405628 3.791469194 2.975721415 0 0 11.26629423 0 0 0 9.688226716 1.721695543 0 0 0 6.217814228 0 22.9704264 7.208443617 33.48357667 18.64168379 7.6954356 0 13.59382074 10.63352322 0 0 14.69712378 0 7.482337829 0 3.945966514 0 0 0 0 6.814690686 11.16157964 4.361695408 0

360 0 2.682899335 0 33.33333333 0 0 7.072744386 0 0 0 0 21.24799465 12.77729342 0 10.23367507 0 0 0 5.368690927 33.33333333 7.684208418 9.218345269 16.83338891 40.53365344 7.427910803 0 0 0 3.511977263 0 9.175124859 0 38.56829495 3.946415641 0 0 0 11.41770201 0 0 18.78139686 0 0 0 5.471035693 0 0 0 0 35.40153805 0 0 6.044789932 0 0 22.65630191 16.18589744 0 0 23.53054689 5.32 4.033333333 11.13666667 0 0 6.684282638 0

361 0 0 7.479784367 0 5.559152316 0 0 6.041951664 0 0 0 0 12.75994347 0 9.432864727 0 0 0 0 0 0 0 5.999607612 9.411982935 0 0 0 0 0 10.41274191 0 12.23230033 0 0 6.19343583 33.33333333 3.231158851 3.690534576 5.320437562 3.657496268 0 0 22.90745078 0 0 4.324061703 3.961969632 0 0 0 0 0 0 12.32418723 0 0 0 5.449900653 13.49972036 8.362245352 2.703333333 4.363333333 0 11.3317757 33.33333333 0 1.180020718

362 4.59121272 28.1841467 1.932626014 33.33333333 3.093031652 0 0 5.279883605 0 1.691536277 6.565726084 0 0 0 0 11.97412611 4.066079108 12.12293144 33.33333333 0 9.938974098 33.33333333 33.33333333 0 0 0 0 0 0 11.75698727 0 0 0 4.892561983 33.33333333 0 0 0 0 0 0 20.40380186 33.33333333 3.9839906 0 0 0 0 0 0 5.572867272 44.91200558 0 0 0 0 0 0 33.33333333 0 6.276666667 12.53 0 4.299352427 2.74490262 0 0

363 5.58663303 0 4.804132825 0 0 7.580786693 0 0 48.57544141 0 0 0 41.12942978 0 5.80031403 0 0 0 0 0 0 0 0 0 0 0 11.25787098 0 0 0 0 0 33.33333333 0 6.600783921 0 17.55693582 0 0 0 0 0 0 13.58782916 18.68792613 6.606670196 0 0 18.89878015 0 0 0 33.33333333 33.33333333 0 0 33.33333333 0 0 0 0 0 0 0 0 12.17688698 14.15397485

364 0 8.277909292 0 0 46.35134593 0 14.40895703 0 12.75733506 6.126003332 0 0 11.80726661 66.66666667 0 0 7.84676354 0 0 46.15541672 0 0 0 7.226859581 0 6.900538694 0 33.33333333 0 0 3.393123098 0 0 33.33333333 0 33.33333333 0 0 55.91706082 21.62383765 0 0 0 0 0 0 20.42045016 33.33333333 0 23.58667605 0 13.18115846 0 0 12.62758822 33.33333333 17.1474359 8.455608058 33.33333333 24.97108798 0 0 3.666666667 0 0 9.783074347 0

365 0 22.04284463 0 0 0 0 33.33333333 33.33333333 0 7.307692308 24.72258835 20.46461101 0 33.33333333 0 2.647191195 0 0 33.33333333 0 24.64955398 57.4483214 0 0 36.37575139 0 0 66.66666667 38.60738936 0 0 0 0 5.511304208 9.799004107 33.33333333 30.79255331 29.5296578 0 0 22.68616832 18.25677267 43.75921589 19.33196869 0 35.44475485 0 15.5533447 2.104400749 0 0 9.14793518 9.087543957 17.05677035 26.6442477 10.67703143 33.33333333 40.41931785 0 14.3407493 7.703333333 0 18.92666667 0 0 6.415149736 12.14631452

366 0 0 0 0 0 25.75254664 0 42.44013463 0 0 0 12.77308179 0 0 8.714830711 0 8.178682871 21.21040189 0 0 0 0 16.49994443 0 0 15.79031485 0 0 33.33333333 0 0 65.05651405 0 0 26.73254941 0 0 0 4.558245281 0 0 0 0 0 0 0 0 0 0 0 27.76046606 0 0 0 0 0 0 0 19.83361298 0 0 0 0 0 0 0 0

367 0 0 12.97918538 0 0 33.33333333 0 0 0 0 0 0 0 0 0 0 0 33.33333333 0 0 0 0 0 0 0 0 25.01506932 0 0 22.92059143 0 0 0 0 0 0 0 0 0 0 33.33333333 0 0 0 42.50770484 0 0 17.77998863 0 33.33333333 0 0 0 0 0 0 0 0 0 0 0 19.42 0 8.192172897 63.92176405 23.55025899 0

368 27.7467003 22.37252471 59.92990783 33.33333333 27.77418102 0 24.17305365 0 20.57599827 26.02564103 0 0 21.52606672 0 17.29934423 56.59917286 54.75382402 0 27.96464241 0 0 0 27.33372572 16.69449082 0 0 0 0 0 33.33333333 0 0 28.09837172 23.87561348 17.3408934 0 33.33333333 22.02877227 0 0 25.19910149 38.63254086 0 0 0 26.72666314 33.33333333 0 0 0 33.33333333 20.15217487 33.33333333 37.28570909 28.65607326 0 0 24.87772528 0 28.79537047 0 0 0 0 0 41.39034731 51.3326711

369 62.07545395 16.43967533 12.87436358 0 17.22228908 33.33333333 21.0119116 12.90469676 18.09122525 58.84912706 68.71168556 45.51431255 0 0 48.51897123 28.77950984 25.15465046 0 0 20.51124995 57.72726351 0 0 26.13301323 56.19633781 77.30914646 63.7270597 0 24.54730004 21.57634606 87.43175204 22.71118562 0 28.44077135 0 0 15.08601869 33.33333333 34.20425634 41.38533275 0 22.70688461 0 63.09621156 33.33333333 26.89785012 42.28424688 33.33333333 78.9968191 7.678452563 0 12.60672591 18.20099944 0 32.07209083 0 0 20.79744817 0 0 6.51 44.73666667 13.92333333 42.84336564 0 0 21.18701881

370 0 0 0 33.33333333 0 0 0 0 0 0 0 0 0 0 0 0 0 0 0 0 0 0 0 0 0 0 0 0 0 0 0 0 0 26.84915963 0 0 0 0 0 0 0 0 0 0 0 0 0 0 0 0 0 0 0 25.29479992 0 0 0 0 0 0 0 0 0 0 0 0 0

371 25.74657818 85.06103729 52.84857324 59.12250217 85.25778886 98.52715557 75.75025345 89.61993728 85.69057598 27.43290548 97.07235911 68.66587222 96.66555742 93.02313993 58.24145535 66.66666667 92.03750923 98.81062617 96.93262411 97.63908216 95.54010461 86.95574225 96.83819165 33.33333333 45.6577042 46.16849084 33.33333333 87.90673377 25.81395881 95.78874914 28.4446649 88.53225409 92.97022007 43.67427931 85.16975329 93.33833066 75.52973482 70.97518936 64.06634738 66.66666667 70.8326166 19.27670592 90.12134399 33.33333333 100 49.03211241 38.85130908 74.93560455 52.01656644 89.34189248 98.17246557 90.01459709 96.0621127 50.09308682 17.84456678 93.36981146 100 93.48911624 100 98.16309491 91.00333333 0 83.28 57.94162826 98.36623553 65.54884508 44.45073587

372 0 0 0 0 0 0 0 0 0 0 0 0 0 0 0 0 0 0 0 0 0 0 0 25.95009597 0 0 0 0 0 0 0 0 0 0 0 0 0 0 0 0 0 0 0 0 0 0 0 0 0 0 0 0 0 0 0 0 0 0 0 0 0 12.13666667 0 0 0 0 0

373 0 0 0 0 0 0 0 0 0 0 0 0 0 0 0 0 0 0 0 0 0 0 0 0 0 0 0 0 25.46390743 0 0 0 0 0 0 0 0 0 0 0 0 0 0 0 0 0 0 0 0 0 0 0 0 0 26.26995646 0 0 0 0 0 0 0 0 33.33333333 0 0 33.33333333

374 11.09747366 0 0 0 0 0 0 0 0 0 0 0 0 0 0 33.33333333 0 0 0 0 0 0 0 0 0 0 0 0 0 0 0 0 0 0 4.972804973 0 0 0 0 0 0 0 0 0 0 6.584143041 0 0 0 0 0 0 0 0 0 0 0 0 0 0 0 0 0 0 0 0 0

375 0 7.511085406 0 7.544164495 0 0 2.319109462 4.425333692 4.647435897 0 0 8.497871257 0 0 0 0 0 1.18937383 0 0 0 0 0 0 0 0 0 0 0 0 0 0 0 17.27361679 0 0 13.7254902 8.291974337 0 0 14.52446796 47.38996075 0 0 0 0 33.33333333 9.530924568 0 4.566972988 0 6.473283628 0 16.57357985 15.48876655 0 0 6.082191781 0 0 0 3.776666667 0 0 0 0 11.08480225

376 0 0 40.26560425 0 0 0 0 5.954729032 0 0 2.927640895 0 2.274426994 4.569723252 8.425211319 0 0 0 0 0 0 0 3.161808354 7.383237364 21.00896247 20.49817582 33.33333333 0 14.30463576 4.21125086 33.33333333 3.54327636 3.569116967 12.20294427 3.975088795 0 6.798394009 0 2.60031929 0 6.169594137 33.33333333 6.557807069 0 0 0 10.53572732 2.890357513 43.12440853 0 1.827534426 3.512119285 0 0 0 3.790985705 0 0 0 0 2.88 10.82333333 1.053333333 0 1.633764466 20.23738495 11.13112855

377 29.82261482 7.427877308 6.885822511 0 14.74221114 1.472844431 0 0 9.66198812 39.23376118 0 7.924657828 0 2.407136815 33.33333333 0 1.788726143 0 3.067375887 2.360917841 0 13.04425775 0 0 33.33333333 17.71904063 0 4.1545331 0 0 4.88866843 1.571546733 0 0 0 6.66166934 3.946380975 4.498230392 33.33333333 33.33333333 8.473321301 0 3.320848939 0 0 11.05041121 5.357142857 7.353414085 4.859025033 6.091134532 0 0 0 0 40.39671021 2.839202839 0 0.428691983 0 0 0 0 0 8.725038402 0 0 0

378 0 0 0 0 0 0 11.35712151 0 0 0 0 5.746771621 0 0 0 0 6.173764623 0 0 0 0 0 0 0 0 0 0 0 22.98261941 0 0 0 0 0 0 0 0 0 0 0 0 0 0 0 0 0 0 0 0 0 0 0 0 0 0 0 0 0 0 0 0 0 0 0 0 0 0

379 0 0 0 0 0 0 10.57351559 0 0 0 0 9.164827079 1.060015588 0 0 0 0 0 0 0 4.459895393 0 0 0 0 15.61429271 0 7.938733126 11.43487859 0 0 6.352922813 3.460662968 0 5.882352941 0 0 16.23460591 0 0 0 0 0 0 0 0 11.92248741 5.289699286 0 0 0 0 3.937887304 8.038533411 0 0 0 0 0 1.836905093 0 0 0 0 0 14.21376997 0

380 0 0 5.445745892 8.281519862 0 15.94939569 0 0 10.52470351 0 9.65900016 0 0 14.0840063 5.796930342 0 6.604369239 8.102884329 0 0 0 12.6206038 0 35.90476226 0 0 0 0 4.926517813 0 15.78156159 0 13.27423306 0 0 0 0 0 24.52735111 5.820287205 0 0 0 7.739824368 0 0 0 0 0 0 16.0544168 0 6.948676585 0 0 0 0 0 0 5.162105839 5.306666667 0 4.483333333 12.35536886 0 0 0

381 23.72566503 0 0 14.67572613 10.42265622 0 0 0 0 0 0 0 14.90548755 45.77339727 0 22.50534276 0 0 0 0 0 0 20.09557652 0 0 0 27.27834033 0 0 0 0 0 25.70698326 19.63754778 0 0 0 8.566279887 0 0 0 33.33333333 33.33333333 0 0 0 0 0 18.36060415 0 0 0 0 0 0 0 0 0 0 0 0 0 0 16.24328508 22.22734964 0 5.917480999

382 15.13779182 0 0 7.068221071 33.33333333 0 33.33333333 16.15419168 0 23.42247552 11.87828066 38.17719161 19.59367595 0 25.97659849 33.33333333 23.86209814 45.14570324 23.91674172 33.33333333 22.63064388 57.12560957 10.98644226 0 28.86409422 66.66666667 0 21.65760254 33.33333333 0 33.33333333 37.46893054 0 0 18.8682946 0 18.54012018 24.18897313 0 45.84207642 0 29.04651823 0 0 57.44493093 31.13905073 15.34978492 19.23293852 0 0 0 0 0 0 21.64080722 33.33333333 0 0 33.33333333 25.23517382 12.76666667 0 0 33.33333333 22.53914989 16.98625759 27.39909431

383 33.33333333 80.38708015 91.0993511 23.19424965 13.56624319 60.20820922 66.66666667 58.85429591 56.02631428 33.33333333 44.7252835 20.23303799 33.33333333 19.24932704 41.38170698 23.1754496 48.58853232 0 9.2591081 27.66708969 43.99859719 13.38313103 30.06353861 25.5742244 53.5678101 23.2459141 56.26130808 66.66666667 40.5119858 80.5350058 11.36677405 26.41597029 20.05910028 41.12855124 41.48185197 69.56135133 66.66666667 6.596500946 43.9925604 7.001104636 85.58998458 20.38426739 26.91194575 77.69085552 22.83793797 66.66666667 60.69314784 9.176915799 58.2853567 100 40.25257009 91.98898485 89.44248917 80.7527726 59.03394256 57.05549264 100 65.7347414 50.41176819 31.12958246 36.62 46.71666667 33.31666667 0 26.64348925 58.40915357 60.74918567

384 3.80071333 0 0 0 0 0 0 0 0 29.70406952 0 0 0 0 0 10.82799057 0 26.68254914 33.33333333 0 0 0 0 0 0 0 0 0 0 0 25.8313826 0 0 13.69578555 0 0 0 0 0 0 0 0 0 0 0 0 0 0 0 0 0 0 0 0 0 0 0 0 0 0 0 0 0 38.06801273 0 0 0

385 8.271019835 0 0 25.45153229 21.32269681 0 0 0 0 0 0 0 28.76172417 0 0 0 0 0 0 17.8520215 10.70268946 0 35.58464788 0 0 0 0 0 11.55226163 0 0 13.74785552 14.18845994 17.65090872 0 17.03358509 14.79321315 17.67317159 22.71989614 10.53974784 0 0 23.41353413 0 0 0 0 55.08904193 9.644430459 0 24.88957597 0 0 0 0 0 0 9.206586826 0 0 11.7 0 9.15 0 4.363431267 16.34707574 0

386 5.806954971 0 0 0 0 0 0 19.0269337 12.45897335 0 21.45505267 0 0 13.90521446 0 0 7.9976428 6.650784197 0 0 0 0 0 21.1369556 0 0 0 0 0 12.83224132 0 22.36724365 0 0 25.18481469 0 0 0 0 0 0 12.94906594 0 0 0 0 0 0 8.381309971 0 0 0 0 0 0 9.611174028 0 0 7.507732216 19.51781599 0 0 0 0 6.689844079 0 0

387 0 16.42276663 3.454903012 10.13908368 0 23.8423951 0 5.964578712 0 0 0 41.5897704 0 0 9.354584809 10.15788373 9.471235195 13.4180791 0 0 9.385908209 16.87065561 0 17.38405773 0 0 0 11.6757308 0 0 7.50195073 0 26.77122346 7.887206701 14.46503874 13.40506359 0 35.88119259 0 12.79818594 9.490206141 0 0 14.56932011 10.49539536 2.194282599 23.95706724 0 0 0 9.033789444 8.011015146 3.608834244 7.33973504 19.32525022 0 0 6.087824351 0 18.95532189 8.266666667 0 2.426666667 0 10.79418345 0 5.934239023

388 9.924521678 3.190153228 0 0 0 0 0 0 8.425433211 3.629263811 12.28238301 0 0 6.988054939 10.13344454 0 0 0 0 0 0 0 3.269794721 0 0 0 10.40535859 0 0 6.632752886 6.184997699 0 0 0 0 0 0 0 8.760192348 0 0 4.286815103 0 0 0 0 0 0 0 0 9.769647696 0 0 11.90749235 0 0 0 0 8.74716626 0 0 8.783333333 0 0 6.742552428 8.257513096 0

389 0 0 0 11.18966731 21.35507044 0 0 0 12.56457565 9.910857811 0 0 3.405778999 0 7.356734844 0 3.476122307 0 33.49081685 21.14755548 13.28216127 0 0 0 17.56809568 10.08741923 6.054993002 0 9.675901418 0 0 0 0 0 0 0 0 7.093881857 0 17.99859796 4.919809276 0 16.34118679 0 9.221735736 0 0 16.50110375 5.32829872 0 0 0 0 0 0 0 0 18.97084742 0 0 3.363333333 0 0 0 0 0 0

390 0 0 11.17051332 0 7.09261622 0 0 5.35701846 0 22.21506524 0 0 7.15278807 8.758021895 0 43.52727493 8.114833131 38.03854455 0 8.433466845 14.71983398 0 0 12.39432227 0 6.423726559 0 17.82525275 0 27.22878132 0 8.396651642 9.302949062 0 0 0 5.819145967 0 0 9.287769571 0 11.57765217 0 18.89278896 0 0 16.34075837 0 10.47059677 35.45711477 11.62503092 0 18.21434962 33.33333333 0 39.98375074 46.41146492 52.22584148 0 0 0 0 0 0 13.32261522 0 24.43129952

391 29.25451436 31.63488799 37.70255892 0 0 0 16.00536193 0 10.7951599 0 14.69543444 0 0 8.024691358 50.68287876 28.54926933 19.99720514 17.27560414 47.42383991 0 7.376830892 12.12679847 0 11.07761869 0 8.322998484 17.01625333 0 10.7374388 0 0 0 8.388127345 36.26439164 13.51160703 23.71264141 20.30811356 14.94151856 3.681346321 10.53300645 0 0 14.67585232 6.934979556 13.06147668 7.807977853 0 13.11492791 33.21974707 0 42.02251965 69.82380208 53.161627 55.96584132 20.31096648 20.30699901 0 0 43.88222784 56.28260802 8.496666667 20.84666667 28.14333333 18.08392689 33.41768114 16.51283186 27.69763776

392 0 0 0 0 10.71441895 34.18540061 0 23.84784142 0 0 0 7.844764012 15.12947854 0 0 0 14.17910448 0 0 29.82216142 0 12.94726316 19.98125084 0 0 0 7.277637486 22.28677053 6.945203981 20.99447514 0 7.959335482 0 0 20.07265506 14.77636806 0 6.44109254 12.32598313 0 18.82637391 5.083046964 6.131160264 14.59056146 0 9.685724903 10.67971724 15.3521634 0 8.522149017 0 0 0 0 43.7744521 0 28.31781361 0 33.33333333 0 24.47666667 0 12.57 17.62562332 0 10.91917791 0

393 0 33.33333333 0 21.88882085 5.15915857 8.572664828 26.57778511 4.636015326 13.07425497 0 6.810321894 9.417817493 8.159232072 0 0 11.71859973 3.94388977 0 18.48860974 14.32280077 17.18111346 0 0 0 0 0 0 9.212239583 0 0 43.98508405 6.915647135 0 5.381142916 0 0 10.76556062 6.771715067 0 0 26.38938903 0 17.92477994 0 17.66381766 18.47458318 0 10.45279383 0 14.48208865 11.46838156 11.36856369 15.11898372 0 0 10.54178916 11.32697245 26.33765371 0 9.001261323 0 0 0 0 25.36465057 0 12.79993798

394 9.757628428 0 13.4176555 33.33333333 0 0 0 0 11.95741196 17.9779701 7.952973721 33.33333333 12.09222941 29.54532504 0 0 0 0 0 0 31.06356594 0 23.521414 0 24.70573018 25.01033485 9.725713647 0 14.0763284 0 0 33.33333333 0 21.08153781 0 0 18.15982882 16.53881559 5.249919971 11.44136929 0 0 0 21.84453329 15.95436086 25.52535548 24.33388948 0 0 0 24.64414702 0 0 0 0 0 0 7.535287731 0 0 0 16.96 0 7.025593963 0 0 0

395 19.0951761 0 7.94291265 30.03236246 0 20.92843535 0 0 0 17.49913542 0 9.925024582 0 8.508748557 0 0 0 0 0 10.02764274 0 41.09797926 33.33333333 76.52805904 20.86134339 24.07085798 29.08996661 26.55464339 0 39.43788535 33.33333333 23.90581842 61.45376553 0 21.92404703 23.44276588 0 10.98994429 22.93388263 8.801719977 33.33333333 36.7343466 0 0 15.66951567 13.14965228 0 0 27.60678232 15.6676844 10.23992085 9.668067836 0 0 0 0 0 0 5.833608331 16.84356253 10.05666667 0 16.16 23.92688172 25.78664744 43.58287306 0

396 0 0 0 0 9.212549481 0 17.3279714 0 20.25907836 26.95246601 25.38035961 0 0 0 0 0 10.73225266 0 0 0 0 0 0 0 0 0 0 0 39.573459 0 0 0 0 0 0 0 23.56523278 0 0 0 0 0 25.76008139 11.99030032 0 0 10.19575856 20.21840543 0 4.0571536 0 9.139566396 0 0 9.899428121 0 0 0 0 0 17.62333333 0 0 0 0 0 0

397 24.03151674 26.62897146 21.02119509 0 20.63193964 2.97546802 25.66155886 7.079512434 8.715067704 0 28.67504577 0 19.13231696 28.36331973 15.88195155 16.20485601 21.27461043 28.62812212 19.24282676 10.57706572 0 0 9.811919329 0 0 0 18.98217977 24.12109375 0 0 22.68158262 0 0 0 0 19.19155266 12.02775971 0 24.40206704 16.66666667 14.50695942 10.96050479 0 0 0 0 25.99201881 40.86170943 28.70287384 12.67030965 0 0 0 0 12.99278645 8.844323114 5.015519721 6.995679628 0 17.87256813 0 4.726666667 0 15.70771001 0 12.13142071 12.10218242

398 0 8.402807216 8.745164515 11.44451249 0 0 0 39.78454462 21.37592138 0 0 23.91551584 18.20385479 0 33.43516968 0 0 0 0 3.511171911 16.15221987 12.25164123 0 0 0 0 17.90824916 0 11.37929828 12.3388582 0 0 20.85515807 0 35.79089183 18.87667199 0 28.41461745 0 12.60419448 6.943944304 28.25028637 27.20217307 0 20.27185666 0 0 0 0 0 0 0 13.50503966 10.70082534 13.02236686 20.32313797 8.928229298 0 16.95083049 0 0 0 8.223333333 17.6302641 0 0 0

399 17.86116437 0 0 3.300970874 47.18931714 33.33803119 14.4273227 19.29506774 13.82310573 15.35536323 16.48586456 15.56354474 20.13010015 16.79989342 0 0 21.75810439 16.05772919 14.84472359 23.30569059 13.50643586 21.57631788 13.3520825 0 54.43292643 36.17208213 0 0 17.28827154 0 0 19.48921398 0 37.27292764 8.700799053 0 9.354358545 15.90229651 31.4068009 30.66527358 0 7.39416311 8.305953012 25.74683641 17.37897247 25.3567063 12.45785753 0 0 9.14349992 0 0 0 0 0 0 0 6.905537459 0 0 6.076666667 31.18333333 5.99 0 2.108405635 16.85369647 22.96894232

400 20.84200631 23.85612032 22.10234757 23.5991125 0 0 0 0 17.64604366 20.87762565 21.51080541 0 6.77673616 0 12.71276299 20.82961482 0 0 9.748997053 0 12.03262835 0 34.61575813 12.13073301 0 0 14.59280098 13.24539212 0 12.53414963 0 10.44765622 13.30888697 0 0 7.162534435 0 0 0 0 27.92019321 0 0 0 0 0 22.11737439 25.85013705 22.40069212 25.00271612 0 15.29680365 14.84891951 21.37970764 0 0 20.64607851 0 0 31.23710466 0 0 0 0 15.52802092 26.66063712 10.06423183

401 13.08198159 0 0 0 0 12.07941739 5.095071073 6.885328783 0 0 0 37.0034861 0 12.94943418 0 0 13.23856457 32.06638806 12.65619994 58.49909981 32.32049201 30.66614881 0 13.33780911 11.45967014 19.03043875 33.33333333 17.69622215 16.83292891 24.10546054 35.80162868 23.9518939 0 22.83295576 0 11.34486908 15.894303 23.32867784 0 18.40465523 10.31435746 22.2540025 18.73738301 7.441364606 24.40503789 17.5486618 19.25377385 12.36942339 14.16166467 4.760725051 86.08350714 31.89513689 0 18.61610968 15.36096376 3.158506312 16.81202758 26.48691831 15.83580125 0 23.74 0 15.47666667 39.49691992 21.70020051 0 0

402 0 18.49114271 0 39.28887904 0 0 0 0 17.25506678 22.53400266 0 0 13.97066812 18.94073637 58.67211078 0 0 0 0 0 0 0 0 0 0 0 0 0 0 0 0 0 0 0 4.569892473 0 5.169172932 19.08712163 0 0 0 0 0 15.6226144 0 0 0 0 0 0 0 0 33.33333333 25.59052309 16.00616252 21.44624691 19.25589444 23.19761782 21.81729241 0 0 0 3.46 0 0 15.93642225 15.20838411

403 0 0 37.93103448 4.033895105 0 33.33333333 7.402621377 0 4.965915955 0 0 0 0 0 15.87700206 24.17488494 17.03020134 26.57769988 38.74392019 25.67472093 0 0 0 24.54265183 0 8.284486127 10.66178074 6.60703076 0 0 0 0 14.49439261 14.26900585 16.33420461 0 8.2734046 0 25.34402366 23.64952653 0 0 33.33333333 0 17.33512251 13.77724455 22.03052422 0 5.199353448 20.58235011 0 7.945158081 25.15836944 0 0 0 0 33.33333333 24.52447228 0 0 12.81333333 9.116666667 16.47112951 17.06269523 0 9.530211368

404 5.364936547 23.76408686 6.788646493 0 0 0 19.70606424 31.02991129 0 18.4376749 0 0 3.117144294 0 0 0 7.333838767 0 7.615080637 0 16.28815991 0 8.756504576 0 0 0 0 0 0 6.563312597 10.17612524 10.97520661 0 0 0 17.72509787 14.57374534 0 0 0 4.419709692 7.837791787 0 34.0670975 9.406081273 0 0 0 0 12.03956479 8.694229747 10.15396324 0 0 21.35441544 33.97678262 0 0 17.49753208 22.8676664 9.536666667 7.756666667 0 0 6.084491461 17.87177289 0

405 18.70652046 0 3.651635721 0 11.39774859 5.667439684 0 0 10.72137372 7.676389716 33.33333333 0 0 0 0 0 0 0 0 0 0 0 0 0 0 0 0 0 0 0 8.236832275 11.66086437 0 0 0 0 0 0 0 0 0 10.92466352 5.694403607 0 0 17.20454895 11.21595894 0 0 0 0 0 0 0 0 0 0 0 0 12.99589603 0 0 3.1 0 0 6.672696211 5.387882853

406 8.288762239 0 0 0 0 0 4.707402621 33.33333333 0 0 33.33333333 0 0 14.39259697 0 10.6077659 0 0 5.240174672 0 0 0 0 17.11052278 7.324492609 0 0 0 9.844893181 0 0 0 0 0 0 33.33333333 0 0 33.33333333 9.722826876 0 0 0 0 0 0 0 0 16.40386737 0 0 0 0 7.742810248 14.88204858 0 0 6.846415019 11.51604093 15.31677883 0 4.596666667 0 14.36344969 10.38232731 0 0

407 0 0 0 33.07811336 0 0 13.62726909 0 33.33333333 0 11.82252793 9.049551211 26.55659717 0 0 0 0 3.423802839 0 0 21.30070498 0 0 0 0 25.04884721 18.74053235 0 17.74636828 0 18.0205319 0 0 0 33.33333333 0 0 33.33333333 0 0 0 51.07233785 0 0 23.21068646 35.68487317 11.30280911 0 0 0 0 0 14.76743731 0 0 11.88708643 14.07743889 0 0 10.46566693 0 6.566666667 0 16.86220382 0 0 0

408 0 33.88865011 15.69786535 0 33.33333333 15.58647626 0 0 0 25.6949891 0 53.94696269 0 0 0 19.27074159 37.96979866 0 13.06205276 0 4.062144377 11.81043555 18.56553938 0 66.66666667 33.33333333 22.67155259 42.36341375 0 24.03764218 9.868604976 31.73956616 52.17227406 0 0 0 41.26592094 24.2508672 0 32.3375369 57.34573963 0 14.59595033 25.15820456 0 15.78467153 14.07955948 40.81652961 41.8344224 17.00946784 0 17.71549673 0 11.95362569 0 21.9774996 29.20856058 10.13571551 8.808861053 7.116887135 39.42666667 13.27333333 0 0 21.81927947 0 27.94545048

409 33.71579286 0 13.82847038 0 55.26891807 33.33333333 49.4615716 28.75142659 16.07826655 4.779317969 0 0 49.57885426 53.71723249 12.73812417 25.11699275 24.42759666 37.93210922 12.93357475 15.82617926 13.99587037 57.52341564 38.06219791 32.87828327 14.54917059 14.30289458 0 20.08794121 55.57580963 32.75943505 17.89627693 11.22481274 20.02444636 62.89803839 45.76256958 30.43416528 14.82345319 0 41.32264301 15.88545446 0 7.911204335 27.63892973 17.71071894 25.64307187 0 0 20.96390994 0 20.60517609 5.222263117 16.9934414 11.89194041 14.71722365 32.3964097 7.553878131 0 0 0 0 0 0 26.32333333 12.80629706 7.422985109 32.85847153 31.86383936

410 0 0 0 18.39368685 0 0 0 12.25422321 16.30525438 33.33333333 26.32126684 6.345661782 0 15.01021835 0 0 0 0 0 11.74813181 0 0 0 14.85184607 0 0 22.93399545 0 0 0 0 0 0 0 0 0 0 0 0 0 0 0 0 10.66258185 0 0 0 25.87810898 0 0 0 0 16.86938307 0 0 0 0 0 0 19.44761905 0 0 0 0 0 0 0

411 0 0 23.84972839 0 0 38.19447006 0 15.54868379 10.05340385 0 0 8.13648294 0 0 8.661575563 0 0 0 13.20967956 0 51.77019539 0 0 0 0 36.3082512 18.75057376 0 0 0 0 26.43216532 0 0 18.88310687 0 8.296110181 8.961804231 10.51090343 0 9.352096159 32.3369084 25.07111936 0 11.05110511 17.67623562 29.80388305 0 11.7740979 0 0 0 0 19.39905691 27.68661519 0 0 26.16039039 0 0 0 0 0 0 0 29.14801722 0

412 43.79700332 22.79749682 24.7369939 16.72508409 0 33.33333333 15.67604136 0 0 0 0 22.61880481 0 24.06181015 0 0 0 22.89886523 15.11843851 14.45254841 0 0 0 0 0 0 29.16973717 0 40.46357457 12.63806179 14.89658248 0 0 9.550816697 0 14.73396366 47.33819027 0 21.560237 68.47874657 17.77915377 0 0 0 0 0 12.11021854 0 11.60302831 16.19033577 0 15.54913913 0 13.48814229 0 42.13044542 0 33.33333333 39.95925722 0 0 21.78333333 8.15 38.15937332 66.66666667 0 0

413 25.65777856 44.3507332 0 29.35016543 57.16941276 7.183881283 16.47482851 15.62477311 56.61326282 47.12951927 41.26130407 44.57937503 9.732937685 39.27549594 14.70391211 45.08341354 27.27749803 20.7118968 8.407660928 0 26.77003611 65.81848889 57.57084706 33.29013779 74.50617424 27.45425979 0 28.05997908 13.17078422 47.6630735 0 21.99251115 43.10864237 0 0 33.33333333 0 54.75363012 19.05363307 0 0 0 15.49213061 47.09033191 40.41161602 42.7057644 23.39579784 37.78526808 39.09949364 20.95080155 71.69419782 49.31435772 53.25630252 0 0 12.29596487 47.38807047 11.05220228 24.62869392 60.14190961 12.91333333 5.593333333 9.23 16.11661166 25.9965338 17.56484344 0

414 0 0 0 11.52985717 0 0 9.685851655 0 0 0 0 0 57.01338826 0 20.87152729 0 25.42616554 33.33333333 0 14.70749418 0 0 0 0 0 0 14.58275957 59.86137366 0 0 66.66666667 14.19004525 33.33333333 0 28.56290996 33.33333333 0 0 22.82242991 0 48.74192639 29.27683616 0 0 18.19544 0 0 0 0 0 0 0 0 0 21.07147266 0 0 11.64748667 0 0 2.243333333 7.603333333 0 0 0 33.33333333 0

415 0 0 25.08810041 0 33.33333333 0 0 32.84568165 17.02807895 6.893126738 0 0 23.60039565 0 25.42170884 25.32785281 0 4.837470048 21.8069163 18.88078493 14.89647127 0 6.59513851 0 0 15.05028736 10.39933789 0 28.39426895 0 0 6.25119334 0 46.50240725 47.7835598 12.44438265 25.03722315 0 0 12.1610669 0 20.46692238 41.59554731 6.073298429 0 7.128434729 6.746134577 13.9530408 18.25143232 0 4.003558719 0 12.00418947 13.93427642 0 5.93079922 14.74451002 0 10.62418784 0 26.30333333 15.48333333 14.37333333 0 0 5.026158445 55.72192513

416 0 0 0 16.60824925 0 0 0 17.70856023 0 0 5.793028067 0 0 0 0 21.58325313 20.15191622 12.62143653 0 16.70137613 0 0 18.39447997 0 0 0 0 0 0 0 0 0 15.33042603 0 0 0 0 20.48212263 0 11.745244 0 0 10.12450082 0 0 0 9.937535491 0 0 13.44207223 0 17.7841942 0 0 5.646718147 20.73536782 10.27908015 9.332517673 0 0 0 0 0 17.21672167 0 0 26.31932209

417 7.675554771 0 21.85498946 3.409789316 0 21.28831532 16.85850483 0 0 0 0 0 0 12.38095238 12.46180605 0 0 5.596998056 30.68982318 0 0 0 0 0 0 5.879073539 0 0 5.346664789 24.48406197 0 9.241226563 0 23.78251664 0 0 7.786774629 12.18364492 11.77309634 0 0 0 0 0 0 12.95423156 3.529450283 0 0 12.38253179 0 17.35230895 0 33.33333333 33.33333333 0 0 0 0 13.88571429 7.21 0 0 15.09816142 7.336799538 14.92764757 0

418 15.81966334 32.85176998 4.47018784 0 9.497253908 0 23.64748168 6.018078021 0 12.64402066 0 7.605146918 0 0 0 0 27.14442022 0 0 18.62583916 0 18.69053797 17.43953446 41.1721858 20.38560065 15.30812811 4.163596168 6.805293006 12.62470748 10.95925082 0 10.5520362 8.227598263 20.16425942 0 0 0 0 14.27970026 0 8.572644118 13.86283589 0 0 0 8.52866298 14.47698022 11.47134196 19.27194784 0 7.567712523 0 13.41036415 19.84519104 0 7.680311891 0 0 16.08322161 6.524757057 0 0 9.913333333 0 0 0 10.94474153

419 7.05 0 0 3.983167903 0 0 17.65729197 0 0 0 26.62440102 10.71452852 9.653278407 9.271523179 17.87947015 8.005480525 0 0 10.76748152 4.883825391 6.56329722 15.49097314 0 10.68583034 5.108225108 0 0 5.273354256 0 4.255551909 18.43675085 11.34082218 0 0 4.770423375 6.154987023 11.54170177 3.618798101 0 7.614942529 15.55417957 4.056497175 7.716701903 36.17378782 30.34183886 11.00667071 0 10.91224018 0 37.03425867 16.73453094 0 4.459760795 0 12.26186068 11.22711078 27.58833935 8.474069646 8.704639412 0 0 0 11.92 13.40913193 0 0 7.014011243

420 4.538919696 0 2.804849061 2.498321805 10.22973559 25.33036009 0 0 7.897340754 5.734433209 0 0 11.92405559 0 0 0 0 0 0 7.634408602 15.83313003 0 0 0 0 0 32.71083699 0 0 0 8.669473995 0 0 11.4228302 7.259867671 12.98401714 0 12.85464397 0 0 26.09385327 0 0 0 0 0 0 0 0 4.902679371 5.787400202 7.60913637 0 0 12.50742821 1.323611522 0 0 15.78856971 0 9.343333333 5.166666667 0 13.08826584 0 17.98613849 0

421 0 11.19850591 0 0 0 0 0 22.32932785 0 0 0 0 38.23255342 0 10.82225697 9.09790578 23.25204863 0 0 0 0 9.398805847 0 0 0 0 0 0 6.13370625 4.827235772 5.730920314 5.959692462 14.90225672 0 0 0 4.741998142 0 0 5.133373648 0 11.82795699 22.34993175 0 0 0 0 0 5.675526024 0 0 3.141234747 4.064164135 4.666276183 0 0 0 0 0 11.76478071 0 0 0 17.37834644 24.16593188 0 0

422 0 15.9678505 0 4.321671095 15.51909052 0 0 20.22744221 0 10.41783774 5.300345007 27.93902916 0 12.97179452 0 0 0 4.765108196 0 11.12212708 0 0 8.80195599 0 0 7.164247869 0 16.92694547 0 20.61548291 0 33.33333333 0 26.82442111 0 0 0 0 17.3266275 0 19.53811909 0 12.43668499 16.45014245 3.464595053 9.164297153 12.31096244 24.24218612 0 3.208730353 0 19.52017448 12.1886903 0 0 6.16685792 12.99118267 10.43236455 0 0 0 0 0 0 6.200969904 11.35766807 2.62555834

423 8.320519392 0 14.91580442 13.16018825 0 12.95661929 22.37509491 0 18.30985915 6.338259442 0 14.62210572 0 38.32520078 6.169625247 33.33333333 0 19.47003467 0 0 0 39.64735889 33.33333333 29.14142874 0 33.33333333 0 33.33333333 35.03336915 0 0 9.776988356 0 0 3.230663929 20.64896755 0 19.68467837 0 0 0 6.382202772 0 0 0 6.873736446 27.38769347 0 11.12027775 33.33333333 14.26884007 0 5.571555556 36.16154855 6.391138698 0 7.99876467 0 14.68552881 20.18119338 7.823333333 11.14333333 6.57 7.248182762 14.37758629 8.207498716 42.60455405

424 16.93366194 0 30.52848427 27.26506031 16.16818379 0 0 0 25.43599258 0 0 0 0 0 8.242116237 0 18.15154038 0 0 8.324907314 17.5002033 19.62918178 0 0 0 0 25.54781379 0 0 43.44850949 19.03651617 0 24.13152755 0 23.60363704 26.89803927 15.74811291 0 0 8.996842035 0 21.57070841 0 0 29.86873828 0 0 0 17.69274619 11.09618047 9.23892201 16.44315573 9.663824365 0 18.47831331 44.42802247 11.01003032 0 0 17.03866168 0 0 0 14.57119655 0 0 0

425 27.71575906 17.45120894 22.72370324 0 32.84637469 36.15613532 33.33333333 0 33.33333333 21.0244243 55.28026484 7.557793866 41.01583178 0 17.73771899 29.64300978 13.81124698 33.33333333 26.45200759 22.42756836 8.176052179 0 33.33333333 0 23.67385141 0 0 0 27.19962708 0 13.75814295 0 29.23428397 18.10392085 12.65567254 0 39.59792533 23.96311718 22.6044505 46.31803629 0 12.21735959 23.7916033 13.05054445 16.48984199 26.10985704 11.96599066 32.33890159 14.13621262 7.868678846 8.201476793 25.72419696 0 16.60911721 9.151585162 8.24514429 16.57244717 32.58555226 20.38681426 0 15.59 25.38 31.40333333 0 0 30.15605645 24.63451424

426 0 0 0 0 0 0 5.291064982 0 0 0 0 0 0 7.821077863 13.58579882 0 0 0 19.39547525 0 0 0 14.28280359 41.29682498 33.33333333 59.5024188 0 0 0 0 0 0 0 0 0 0 0 0 0 0 0 0 0 16.88319088 33.33333333 10.54678563 33.33333333 12.60101766 0 0 0 13.81315885 0 0 0 0 0 0 0 17.50567752 18.75 0 0 0 0 20.99320324 0

427 0 0 0 17.79598465 18.30120145 0 0 13.10589113 15.02347418 0 0 0 0 0 14.26896013 0 15.18179295 0 0 0 33.33333333 0 0 0 0 0 41.74134921 49.73972119 16.92609855 0 19.57519038 27.37364087 0 0 0 26.78461026 0 0 26.19235421 0 7.239480062 14.73377097 19.26422617 7.557710551 0 0 5.945639864 17.37381601 21.77500639 0 0 0 27.76177778 0 0 20.91503268 0 0 18.64780452 0 0 5.106666667 0 15.9549869 28.1231485 0 0

428 0 0 0 20.17314508 0 0 0 0 0 14.48908994 0 13.48019932 0 0 0 14.51788462 8.614593378 0 0 35.33100666 0 17.62050193 0 0 0 0 0 0 0 0 18.33613917 0 0 0 32.57249803 12.68436578 0 0 23.14768495 39.55174803 47.12854758 0 0 0 0 26.45959689 0 0 0 0 25.13185654 0 0 22.83249158 14.85502002 18.92133112 29.10427216 0 0 25.46022924 9.79 0 8.986666667 0 0 11.29943503 0

429 42.4911399 55.38243465 29.027159 14.78562881 6.935413958 25.5568853 39.00050678 44.33733882 0 41.99595537 39.41939015 36.40087193 8.827559209 40.88192683 29.17352361 13.40786649 20.98877768 42.4315238 54.15251716 15.15998197 25.15728115 13.70415155 10.24857376 29.56174628 42.99281526 0 0 0 14.70719896 31.10877183 14.89361702 23.55634498 31.73193176 43.64882784 20.6776608 0 39.91196362 43.49756048 10.72888283 0 0 33.26800127 22.15755379 46.05841167 16.84349135 20.84572685 9.056380236 13.44407862 29.60023102 39.59039763 37.37150439 13.74894285 40.74998786 19.73056648 38.6165146 0 22.32330301 56.98208319 30.4912827 8.049457482 23.68333333 0 18.67 31.75902151 27.13236343 0 30.13537337

430 0 0 11.80956612 0 5.617230053 13.66861436 13.42406567 0 0 0 8.956738576 0 22.36082575 0 0 0 25.15321592 5.978509374 0 0 35.12002799 0 13.0258624 19.42421677 15.32640647 0 9.671322546 0 0 11.05271939 0 0 0 0 0 0 0 0 0 0 0 16.3075042 9.677055104 0 0 0 0 24.36056338 0 0 0 15.06672113 0 0 0 17.31143686 0 0 0 0 0 8.903333333 9.783333333 0 0 0 10.00494862

431 0 10.09546074 0 7.615951907 10.99300204 9.031066331 0 15.9928535 44.15926766 22.33956133 0 18.01104309 37.42559821 20.14022787 0 23.43002442 18.72340426 0 0 7.803634274 0 33.33333333 24.74221421 0 0 39.51878597 0 51.00620777 16.1105226 16.37627909 16.76466745 20.00592894 0 0 15.4668681 29.89859097 14.01315521 15.31634335 0 33.33333333 36.03331305 0 0 0 34.06821382 11.39885808 13.59838327 6.927528721 0 22.83477149 8.228656589 9.849797805 20.7012488 16.09640867 0 0 0 0 0 19.61307796 7.026666667 12.25 5.043333333 0 34.49617612 26.95001599 0

432 6.272260633 0 23.64601763 0 0 0 33.33333333 0 0 0 0 0 0 0 0 0 0 0 0 0 0 0 0 0 0 0 16.78274545 0 20.65324975 0 0 13.26819598 0 0 0 0 0 18.22633876 5.909870575 0 0 0 0 0 0 0 13.90509014 0 0 0 0 0 0 0 15.6005444 0 11.74599693 0 23.94340544 0 0 0 0 0 0 12.93194331 0

433 35.36841095 9.242152672 14.40841108 25.66375406 29.65726212 12.21453786 12.92287751 0 0 10.34660365 6.19363316 0 0 0 71.45015577 0 0 23.84519351 42.44056269 22.92879534 0 0 0 20.47213115 0 0 10.94501718 0 0 0 9.322678843 25.5994447 0 0 4.903053265 12.77165354 28.16317069 0 11.66902405 0 0 0 33.33333333 31.28902144 0 0 10.5677441 0 5.295356136 18.04258498 36.81128421 3.938589325 16.39480692 0 13.28835161 0 22.67117004 44.13307112 0 0 9.46 4.28 0 19.61037563 0 0 19.6589525

434 0 0 0 0 0 15.53631052 23.67519645 0 18.57816262 0 17.59162607 0 0 0 0 0 0 23.41106539 0 13.65725777 0 0 0 0 0 0 0 0 0 5.155155155 0 0 33.67550331 22.74861459 0 0 0 6.212755167 0 0 0 15.5792277 23.65627823 0 11.30078073 0 0 0 16.83074593 6.956429104 0 11.57206817 16.20799537 3.66146237 0 22.64669085 12.91971094 33.33333333 19.56379718 20.11120651 4.026666667 0 14.57333333 0 0 0 18.15462056

435 0 0 18.44343635 0 0 5.696053736 0 37.45421562 0 33.33333333 0 0 13.24494442 23.97620682 16.48021545 33.33333333 0 16.09356361 18.96576218 0 39.8262052 18.33594158 3.010337385 29.31875345 4.953578393 0 23.66201079 13.44894027 33.33333333 16.95705425 0 0 33.33333333 0 19.08297924 10.92110025 19.32017813 0 33.33333333 0 5.13341895 17.02582913 0 33.33333333 0 7.215198477 8.209614705 42.30610329 0 28.47054617 0 24.04804329 0 21.25200537 9.946693887 27.91272345 13.8078526 10.76696165 34.73600308 0 5.946666667 8.986666667 11.91666667 66.66666667 19.54273897 15.73152161 0

436 0 34.00175424 0 25.71738143 0 0 0 0 0 0 27.13970017 15.32229024 0 0 6.318522793 9.90330891 13.0699055 0 0 0 13.72098588 0 22.35067437 0 66.66666667 60.48121403 0 14.74217175 12.68008358 0 11.42078158 4.20822838 0 0 0 0 19.42762399 11.80423482 21.66430929 0 33.33333333 0 33.33333333 0 0 0 19.73495006 10.69793556 13.39005135 0 19.44871258 14.32802288 16.93852641 33.33333333 40.70377659 0 0 0 0 26.79277851 0 5.466666667 0 0 33.33333333 0 0

437 20.80962364 23.23787259 0 28.12938263 12.98490707 12.73729831 6.986390149 33.33333333 7.89532946 1.597617113 33.33333333 54.92091388 10.97250758 33.33333333 0 33.33333333 33.33333333 0 15.6784527 22.27697927 0 9.294080405 0 0 0 0 0 0 0 50.45879213 0 0 32.99116336 40.24452369 33.33333333 6.020541121 19.07587199 35.39489381 0 33.33333333 25.49993467 0 0 0 36.63585781 66.66666667 22.76558924 0 0 18.83288109 11.93259486 0 29.7574225 0 7.786095044 0 0 0 0 0 0 5.356666667 13.03333333 0 12.62775157 6.853821341 12.23765028

438 0 0 24.57721269 12.87352998 22.3403313 0 0 13.21959755 0 0 0 0 0 0 5.751105982 0 0 0 8.547651276 0 0 0 19.57377809 11.98522622 0 0 22.38831615 0 17.22281073 0 0 0 0 22.7826018 0 7.356134636 0 0 27.42346276 33.33333333 0 17.75410564 0 17.79807093 0 14.71927678 0 0 28.0379772 4.862787164 0 11.91146736 0 0 0 0 20.41362239 0 13.76953616 0 6.033333333 0 0 0 0 0 0

439 37.54970478 23.42275975 7.115356138 0 18.40726742 31.11611889 9.65813688 0 29.36724025 32.38288457 6.784968685 11.74575278 15.99612403 22.55023198 0 0 9.720140987 30.67166812 14.36757115 33.33333333 11.33278093 39.03664469 17.29713355 18.79967241 13.05334847 0 16.55058788 20.80268021 0 0 62.49187212 36.91820201 0 14.22425992 27.21376607 33.03197948 0 13.0454341 0 0 0 33.33333333 0 17.5795743 17.99514764 0 11.21862849 15.70786905 36.44586938 0 23.57875176 9.285290042 0 25.65679026 12.67453847 32.12914884 18.44164709 11.76663389 7.987258141 33.48293702 23.83666667 10.95 0 13.7229577 0 37.53269776 39.94382804

440 0 0 15.67083838 0 0 9.89602298 26.57437714 0 0 26.56996777 11.91621851 28.24000605 17.49721604 0 13.0734767 6.64542558 11.88752112 0 0 0 13.34240209 29.52882926 12.04814553 0 23.29873126 11.4491713 14.86467387 12.87374079 20.53684879 25.96809528 0 20.29175306 0 0 0 0 17.6899553 32.79636047 0 0 46.74074786 0 24.82374601 13.99854334 17.83729876 0 0 0 0 0 0 0 0 17.15222253 0 0 0 2.764055516 0 0 0 0 9.703333333 12.39641468 0 0 0

441 23.22367596 18.2364886 8.461057816 11.75160123 43.09172976 0 0 33.33333333 7.86746596 0 0 9.13884007 14.75309183 33.75245502 32.45752796 11.61565479 0 0 7.822691383 0 18.15451815 0 8.614908434 0 0 36.43131248 0 25.33501277 28.83210869 12.94498382 0 0 51.23454122 0 12.73340249 12.38158185 7.541197585 0 34.30246282 60.04224136 4.382943342 0 33.35625325 0 23.23643594 0 6.687205509 16.56797864 7.108906799 0 0 9.509118333 6.827376939 0 0 9.538811587 5.03360717 0 0 11.47546977 0 19.51 12.86 19.67696396 17.69890565 0 0

442 0 12.92443289 0 0 0 0 0 24.16477794 9.559120938 0 0 0 0 0 0 0 0 0 0 0 0 0 0 33.33333333 0 0 0 0 0 0 0 0 0 12.24665804 0 0 0 15.05157186 0 0 0 29.60665513 0 0 0 12.18584159 39.25586147 26.19639192 0 17.53746456 0 0 0 0 0 0 0 0 0 0 0 0 0 0 0 32.98480591 0

443 0 23.22911947 0 33.33333333 0 0 0 0 19.84841663 0 3.774082871 33.33333333 28.20691336 0 0 0 0 0 0 0 0 0 19.6767497 33.33333333 17.49876421 8.014719081 0 12.12237094 4.026392352 14.0542414 15.42341701 0 0 12.90872462 38.82006086 13.02564103 0 0 4.881629657 0 0 21.65083299 0 0 8.43219159 15.70279797 0 17.12467008 26.22442653 5.108278935 28.74452596 31.98257081 0 0 0 0 8.576432929 0 0 0 4.976666667 1.396666667 8.92 0 0 10.7849778 0

444 10.36813345 0 0 33.33333333 13.79695886 33.33333333 31.86183685 0 0 0 44.37150785 0 0 0 10.98598599 0 23.80952381 49.89166423 33.33333333 0 33.33333333 22.79049861 21.2851878 0 0 0 0 0 12.79648454 0 0 33.33333333 0 0 0 16.86521841 0 0 0 0 13.29928337 24.25319455 13.69664549 0 0 0 18.72659176 0 18.70730426 33.33333333 0 0 33.33333333 0 51.82867784 15.52566419 0 0 0 33.33333333 8.156666667 9.133333333 0 20.93691865 16.92820383 0 0

445 27.894013 9.915474642 70.93606236 21.58173211 36.61268074 42.25409933 19.27967126 24.67776931 32.45431733 0 0 19.09392697 0 32.91421164 27.03444161 55.54165475 21.44581221 13.28960725 33.33333333 92.56534582 19.99093125 12.17269966 24.7184249 12.86469345 25.8691712 12.37417137 43.9700235 28.45791311 0 20.38834951 17.10442837 28.41161526 29.22870821 21.33005453 16.63187196 41.25944379 62.33847529 16.8425314 49.31791766 13.74564264 14.5776743 11.68250034 8.509587319 78.84833148 45.61132902 6.278916061 18.87947269 7.601880878 18.12511219 26.68075117 39.50046253 50.45095404 47.53596314 76.84342738 0 57.12785508 33.33333333 93.72304556 66.66666667 55.1911969 17.61666667 15.37333333 24.16666667 18.3908046 40.51464064 32.7555934 66.66666667

446 0 0 0 0 0 0 0 0 0 25.24072612 29.55925046 0 8.154255475 18.91763212 0 0 33.33333333 0 16.21610024 0 0 3.804504073 0 0 33.33333333 13.85103468 0 0 21.62822111 19.27909193 33.33333333 0 0 20.42460871 0 0 0 0 11.49798986 0 0 0 0 0 0 15.36527887 0 0 0 0 0 1.350762527 0 0 14.89276222 0 28.29972616 0 33.33333333 0 0 0 0 0 0 0 0

447 0 15.09684474 0 0 0 0 22.28411475 0 0 33.33333333 0 5.093327288 0 0 0 0 0 20.04372609 0 0 15.17881518 0 13.65658363 0 0 0 0 0 12.17994451 7.365238051 0 0 0 21.08667529 0 16.46811493 0 25.61190947 0 13.29383166 0 0 0 0 0 21.14749174 8.5313325 7.136941414 14.62602908 0 14.08618634 0 0 0 14.83798883 0 24.7569004 0 0 0 0 0 0 13.65636937 0 9.495824312 33.33333333

448 23.49174631 10.49342581 0 0 0 0 0 0 0 0 0 0 20.96253727 0 9.268643644 0 0 0 0 7.434654183 0 21.16063367 0 20.46863989 0 13.86944295 8.045367819 21.2109624 0 0 17.90991633 5.111135363 19.53675058 0 31.8146647 0 5.587493696 9.697626792 0 0 20.99935114 3.726678207 0 0 4.88274469 11.3516193 0 16.20866325 0 0 0 0 0 0 0 0 0 0 0 0 0 3.22 3.343333333 14.94252874 8.453120379 0 0

449 15.02243129 10.10421386 4.932041448 0 6.498630646 14.51654435 0 17.82411942 30.27067914 14.85597277 10.37894031 5.100566296 10.42598602 14.41570121 7.179924096 26.19726488 9.523809524 16.77500244 9.29454171 0 0 10.54283472 0 0 0 4.01014813 33.11993481 0 0 0 16.22890496 12.85216298 0 12.00327881 0 0 6.84287812 0 0 12.91828434 0 9.080138783 19.61376793 7.15312518 0 17.96805446 7.919536064 9.163473819 15.20822115 17.340172 17.66882516 6.706594298 12.30332659 6.00435009 18.44057111 17.80766914 0 3.512898926 0 0 5.353333333 0 2.156666667 0 16.4051295 13.97879859 0

450 0 0 0 21.08651457 4.832239241 2.637348869 0 6.218279183 0 0 0 0 0 8.770267425 0 0 0 0 6.701268743 10.16419442 8.373114378 10.74301439 0 33.9316544 0 0 0 13.0221237 0 16.76274054 0 0 0 14.12671604 7.985089268 0 0 0 0 22.83818611 0 0 14.281404 0 0 0 6.409185804 6.896984925 15.68235391 19.68475554 7.02338816 7.381166592 12.88762639 0 0 0 0 15.56778634 15.36927389 17.68900445 6.623333333 0 0 13.670832 0 0 27.54406458

451 0 0 17.28457335 6.951475491 0 0 46.3173903 5.640423032 34.33783084 0 0 0 6.970364928 0 0 0 40.70761142 0 10.99307903 12.02199662 0 9.160749138 14.66990337 0 0 15.2086694 0 0 30.90612193 0 0 6.708070023 0 14.53923117 29.63349348 5.748976009 0 0 0 0 0 38.04753316 0 12.27761486 7.185900611 0 0 11.51898734 0 10.23914357 0 0 10.39459078 16.36909464 16.30262741 0 44.40151231 0 9.089731131 8.833108715 4.95 0 4.113333333 18.49699068 0 0 10.96533852

452 0 10.61501698 0 21.82467243 0 0 0 0 0 18.69565217 0 3.234842324 0 0 23.92950881 15.41777188 0 0 6.158407454 0 22.32377614 0 8.612283169 0 40.15930474 0 22.11437372 10.4185256 13.85230326 0 0 0 0 0 0 0 6.463195691 6.837204436 10.90353457 0 14.08949108 33.33333333 11.09887589 16.79347826 0 33.33333333 26.13005051 0 0 0 14.81522584 8.358866849 0 0 0 20.2418692 0 0 0 16.41337386 0 10.52 0 0 0 22.79759877 0

453 0 0 12.92792793 0 23.91323595 40.65429274 0 15.68417097 0 0 27.47693495 25.57010814 0 46.11401888 50.98259067 45.27593375 33.33333333 27.43350494 0 0 0 0 0 14.11267064 0 0 0 39.04499081 0 16.02899443 25.8530783 0 33.23087172 0 0 0 0 14.10824296 25.16795866 10.29269473 0 0 0 13.04511278 59.83124237 19.30032537 0 18.7294686 10.24925842 31.63892027 7.602887084 30.15199177 12.56938137 20.28156222 0 20.01856738 15.86511885 16.5304693 22.15036365 5.362758311 0 23.87 0 20.3567217 16.19140625 13.96759923 0

454 54.94676304 20.98714794 0 14.2033996 0 0 21.58607865 0 0 27.08089728 18.85328836 0 59.69630174 0 0 0 0 21.79049246 23.65634174 21.31133672 0 26.70442524 0 0 0 21.58983747 20.06633499 0 0 6.307742621 24.89993936 30.12266336 33.33333333 34.52755239 0 44.00021732 33.33333333 35.90206621 16.909645 16.74571555 38.86483215 0 0 0 0 0 0 26.43634841 0 0 0 0 0 6.725654395 33.33333333 0 0 15.39192399 0 0 8.743333333 0 0 0 0 0 26.93331522

455 22.40425061 15.74644699 20.40540541 0 0 21.95076114 0 12.5620389 35.17702053 0 0 45.46876723 15.77714467 0 0 0 6.958906458 0 0 23.55191978 12.81580558 0 18.66342996 0 0 39.63187104 6.480752061 0 14.70588235 0 0 33.33333333 7.099143207 0 6.965488215 0 56.30593451 0 11.451306 11.53846154 12.18967229 0 28.12998018 0 19.766386 8.570705153 53.60473208 0 0 9.621182331 8.390589215 0 32.94071692 0 17.03070592 32.26348708 3.904533653 22.04372066 0 0 16.54 7.013333333 40.01333333 0 17.84157908 8.920906116 10.52071698

456 0 33.33333333 33.33333333 0 0 0 0 27.6929103 0 0 39.18973172 20.50682797 0 10.62855338 0 0 19.00014879 0 0 23.16913891 0 17.52046288 0 22.67869071 20.50752278 0 21.27095268 0 9.285105825 25.41287879 12.62538914 0 0 0 25.34824407 27.58435732 0 5.157578535 5.382587925 0 0 15.97510373 0 0 0 0 0 0 20.01517122 0 12.82136648 13.05703381 0 18.40250067 0 13.31476595 17.46821448 0 27.06888581 36.53731641 7.733333333 0 10.31666667 19.66250134 33.33333333 10.67969871 0

457 10.92908272 12.3461854 16.04875998 5.876816505 71.25452481 26.01237392 15.70746419 0 10.95623501 35.18081489 0 0 0 18.72242729 16.59444466 0 0 4.309534412 27.17492588 0 45.47774671 7.661641958 58.0543835 0 15.89600359 11.74349587 20.30970927 20.77628397 21.05466238 17.30433891 29.12130495 0 8.506731946 7.546076883 30.06768497 15.15806008 0 14.88282067 19.14386744 0 21.14366104 0 19.05192933 0 0 2.81048234 0 36.41821073 33.33333333 13.06452443 0 0 31.20768454 23.29035541 0 0 13.6758538 16.80286404 26.32174552 0 0 22.26333333 0 14.83634265 15.49175425 21.56042749 0

458 11.71990363 6.971869367 0 0 0 0 16.38906687 0 11.30920937 19.04263566 14.48004497 0 17.55618867 0 0 17.91556145 0 24.05330917 10.53704598 0 0 0 0 29.27698424 0 0 0 0 0 18.18330472 0 11.57124235 0 9.748386817 0 7.508389262 0 0 0 21.9973243 0 12.64402977 14.17330039 57.8837941 0 0 0 0 0 11.72407839 33.33333333 19.54436451 0 14.93083267 0 0 4.684766902 0 0 0 7.056666667 0 0 12.97661164 0 0 5.976959904

459 0 0 0 30.05712141 0 8.745223329 0 32.20217762 8.219704244 0 0 5.21945433 0 15.76473303 8.493455862 21.39073292 0 22.41315902 14.77893118 9.781413555 11.0095572 28.20970639 0 0 23.43716889 11.82612623 9.75787728 16.73807592 10.19592425 0 7.500288251 18.26469093 17.8299198 19.5120367 0 0 3.897536463 23.11208718 11.0411004 16.58761778 13.71234344 0 13.26451021 0 13.21647102 35.98515381 13.85603161 0 20.71988312 4.027395465 16.01320989 21.50657647 0 0 0 14.16131039 0 13.66323568 0 15.16443825 5.503333333 18.71 8.393333333 0 17.14192708 22.07376968 18.05960479

460 4.137585991 6.183677407 0 0 0 0 0 0 8.291913215 0 0 0 6.496376812 5.279034691 11.99878361 12.84545314 22.52292127 0 0 10.11821744 0 0 0 0 0 0 0 0 12.21571421 0 9.380988885 7.806955287 0 0 0 0 0 0 11.10201855 0 8.55760508 9.855793138 0 0 15.05572812 9.507406132 0 0 12.00442745 0 0 0 0 0 16.03675287 0 0 0 0 10.23296593 0 9.766666667 0 0 0 0 0

461 0 18.87702787 27.13836539 0 8.383734081 0 17.83085121 0 0 6.150114155 17.6573825 0 0 0 8.368506071 0 8.571605397 33.33333333 13.31591322 19.47925608 0 0 9.369248787 6.055302765 0 33.4021602 0 0 0 0 0 0 18.38727995 0 12.40274068 10.43173351 6.640801811 0 0 18.83713126 20.81010777 0 0 0 0 0 0 25.28887364 0 6.273006135 0 0 12.89775138 19.72559742 0 26.14912155 23.23324789 4.205607477 0 0 20.21 0 0 8.416945373 0 0 3.288530466

462 0 0 0 21.56512113 11.01335186 25.23287269 0 0 15.06328549 0 0 18.74660388 33.33333333 0 17.77137368 4.320688727 0 0 0 0 8.735872022 0 14.3067196 6.344733359 0 0 19.34063706 0 0 0 18.29398 0 0 8.472961857 30.76018466 0 9.415826801 8.067940552 0 10.01680849 0 30.73983917 0 0 13.18728522 0 0 5.876083903 0 1.945996276 12.35615705 5.61971831 21.67922165 16.37891867 0 0 0 13.13826736 6.889218595 20.05094614 2.903333333 0 10.82 0 15.5787164 10.38064824 9.654717396

463 24.08401042 25.73667683 0 22.84359114 6.636155606 0 7.083333333 0 0 7.532803046 0 0 0 5.767012687 0 0 0 30.75145415 16.81436843 21.69258221 0 24.33615819 0 10.41884452 0 0 0 0 12.42801661 5.42252865 0 15.25524632 0 50.77264736 0 0 5.574189427 8.396892655 29.24750026 0 0 0 15.74524969 0 0 3.295441497 0 0 9.438518817 41.83026585 0 38.21194452 0 6.08974359 30.5513293 0 11.38991927 0 0 10.22704134 0 9.333333333 7.63 0 21.06046607 0 10.10304659

464 8.400250156 0 0 0 24.94959925 33.33333333 0 0 0 15.93079074 15.54364102 0 13.39492754 40.43350168 21.35904845 0 29.11036036 0 0 0 9.203330198 0 10.68904594 27.27803057 18.93997251 12.70332349 0 5.890081016 17.721463 49.69840465 0 33.33333333 0 0 0 66.66666667 0 0 0 2.802227412 6.803749622 0 26.7331434 17.81692407 22.8777681 17.16231359 0 0 26.95368292 0 20.36096725 10.14084507 7.711073398 0 0 0 16.47523774 39.33508567 39.9851632 23.1003674 0 12.44 11.31666667 13.24319639 0 12.80079738 12.90642272

465 0 0 33.33333333 0 0 0 33.33333333 28.64039898 5.191321499 0 20.95785441 18.20481965 0 0 0 0 0 0 0 25.49482838 26.87152977 14.64909238 23.96408455 26.98859997 0 17.13314358 12.70180899 19.30696977 0 0 45.22805673 0 25.05354629 0 8.382147838 4.654832347 0 28.22840825 0 9.8382183 0 0 33.33333333 19.85545335 0 17.28773458 85.51190643 21.22778382 0 12.81191806 16.24421761 0 0 0 0 37.23969763 0 3.174603175 4.70092865 0 0 0 0 18.19375464 22.78294455 33.33333333 0

466 0 10.66422913 15.85897087 21.08643588 0 0 0 0 25.40185846 0 30.07 47.92006279 0 0 15.56195965 33.33333333 31.57812145 0 13.14905058 0 24.59746131 0 19.02661373 0 47.29474082 0 33.33333333 0 20.90531673 0 0 25.52637805 0 0 15.53052297 0 11.3001118 24.93644068 37.46522342 24.96264121 12.52322557 12.33880325 0 11.80871499 0 0 0 0 0 0 0 0 0 16.95441466 15.88685666 14.49993953 21.94341407 0 0 0 12.08 4.396666667 0 9.395515917 0 13.58147047 0

467 33.33333333 33.41265721 12.86980232 11.7682122 10.40122044 41.43379398 23.20248213 33.33333333 26.20152272 27.18321918 0 0 46.77536232 26.23316498 24.94032853 20.48788019 0 24.90826143 41.71198576 23.21511589 0 52.01757429 22.6442874 0 0 20.63000984 23.9259129 33.33333333 15.61187033 27.91080468 15.03935333 0 33.33333333 0 14.44204686 0 50.61003806 0 14.52536825 7.905242147 0 15.34505121 0 0 35.42103441 0 0 27.45724943 33.33333333 0 0 28.45472215 0 17.08276965 0 0 0 0 28.63240468 36.38867919 5.506666667 13.21666667 23.30666667 20.09013694 30.02748419 0 19.94175627

468 9.249322909 0 0 22.73663965 0 0 18.55 20.25641026 0 43.20307288 15.77112207 0 0 0 0 0 0 0 0 0 6.461803561 8.997175141 0 22.91448881 14.39336083 16.13136289 10.69830772 41.46961588 21.11761912 16.96826202 0 18.07808702 0 40.75439079 13.08228731 18.24676748 0 9.899767936 0 20.06466679 0 0 0 15.51640927 0 36.70150544 0 0 0 0 28.52894949 17.57276995 12.72450856 0 0 12.20677883 10.10008544 0 19.79228487 0 0 0 0 15.13957869 0 20.53253595 44.10552655

469 20.79549719 5.125731558 10.79952809 0 38.61593876 0 0 17.76985743 19.85009862 0 0 15.12851368 0 22.28728596 0 29.01264461 8.216991524 11.00695109 15.00868201 0 24.13000314 0 0 0 19.37192584 0 0 0 0 0 12.05762106 0 23.22584042 0 5.400069678 0 16.4590321 20.47054992 7.659889515 5.573064398 51.30531196 31.72051324 24.18827358 35.00249832 13.45818415 16.04559875 14.48809357 20.1500092 18.27003748 37.13881368 22.5097086 0 44.98744502 23.76855601 37.52506117 9.90446245 16.85809559 40.14643632 0 0 7.436666667 0 16.39666667 15.52087204 10.55038878 9.371214624 0

470 12.37709459 24.18121095 23.78844083 6.483997585 0 12.39114918 17.3165346 0 0 0 0 6.963559249 0 0 19.31492245 0 0 15.27331878 14.68759854 10.48822448 0 7.318943147 0 0 0 0 21.36155357 0 0 0 0 15.44958175 18.84419787 0 0 9.589990042 0 0 34.26455526 0 0 0 0 0 0 0 0 14.9952919 0 0 0 0 0 0 14.39236968 0 7.778660312 19.2123966 12.87241625 25.55640829 10.17333333 2.4 15.79333333 0 0 0 0

471 0 15.64195298 18.40982059 18.18055362 16.3183846 0 8.258101302 0 0 0 33.33333333 26.64586583 33.22491141 31.69448203 0 8.76006106 18.79733666 18.58643605 0 24.40480122 0 0 42.02432046 0 56.90532638 37.845256 43.31998843 13.77228292 0 7.47311828 8.087571072 8.597817625 0 11.36356343 0 26.06547619 19.45674044 17.85006543 5.910567848 28.39729359 19.52981651 0 13.75714766 8.353557287 6.700610998 13.15952003 50.64102564 0 13.33524246 15.71533691 16.02112676 18.19877982 0 23.61917803 10.04552652 6.62976395 15.87370242 0 0 24.7176763 4.873333333 0 0 0 46.26681207 0 0

472 0 13.07565246 0 0 0 22.55338078 0 14.45609706 18.69918699 0 27.31581278 0 0 0 17.52992634 8.656021927 0 0 0 0 0 38.54605028 0 45.63073196 0 0 0 12.40374037 0 17.75503241 0 0 0 25.13745045 13.60227151 20.19451326 0 9.857431642 0 0 0 9.739573622 38.83254612 0 23.21917808 0 0 0 11.5694165 12.75500385 18.85840429 0 0 0 0 14.69962124 0 15.80725724 0 0 12.04333333 14.09 0 0 0 0 0

473 4.029912754 0 33.33333333 0 28.74724366 0 11.42519119 19.84126984 18.30335607 21.16141001 0 19.70483857 6.290785696 0 15.45055513 0 0 7.444030649 0 0 47.77576651 8.492569002 19.86952224 0 0 6.269293924 0 33.33333333 67.51554985 0 20.89216719 0 41.994122 4.909983633 29.71576231 0 19.52909519 0 0 0 35.16566483 34.97286785 0 19.37561422 5.486005089 3.195251 0 29.24382577 19.99809087 6.379498364 0 6.709554444 19.76052276 0 0 5.987147674 33.33333333 0 0 0 0 0 6.996666667 43.06451843 0 31.86552693 18.12375992

474 0 0 0 11.29227053 0 0 0 8.367843292 14.59658829 0 0 8.456201214 15.48487199 0 0 11.07122507 27.44276507 6.445511422 0 0 0 0 0 0 0 0 0 0 0 45.31498581 10.94838462 47.20459496 0 19.52369241 0 0 33.33333333 0 0 10.21643434 0 0 4.809956273 0 0 0 0 12.43956783 33.33333333 17.61799643 0 0 0 0 0 17.21474414 25.55467302 0 0 0 5.603333333 9.656666667 0 0 8.471631692 0 19.5329087

475 23.78854626 22.8780338 7.22190746 0 13.02340142 19.36337625 28.57859364 0 0 42.31147311 26.37367652 0 0 0 13.30536913 4.537885096 33.33333333 0 5.98134631 8.254828073 0 7.396294709 0 39.85983734 7.452036064 17.59561292 30.41887258 14.19600293 0 0 0 6.77801985 0 8.195882879 8.37351334 31.01120043 0 0 17.59907532 18.99647786 8.822629969 0 0 0 10.11415525 15.08653914 18.00328122 0 21.76391683 0 0 21.37531069 0 37.74097761 17.32936491 0 0 0 47.35264346 3.303125821 0 10.03333333 4.46 0 0 0 33.33333333

476 2.9705027 9.152122387 0 33.33333333 6.368985808 10.77995255 34.42157927 0 0 0 0 0 13.24066823 16.17028732 0 0 0 0 14.05908616 0 18.30090296 0 0 0 29.99378912 19.82021482 0 0 0 0 37.6869284 0 0 0 0 0 0 0 0 0 0 19.80068921 0 26.50100324 16.0556687 7.776487289 0 0 0 0 17.31220657 27.09257616 0 0 0 0 0 0 0 7.776925045 3.743333333 12.12666667 0 0 0 19.10638298 0

477 33.33333333 0 9.544892505 0 0 0 0 42.28235304 0 24.92294144 12.97717736 0 22.66431433 0 22.67663189 0 20.42656494 41.6347192 24.72058993 0 33.92333053 0 0 0 5.648848438 0 4.89958542 19.56105041 0 13.87856258 0 0 0 17.05978627 0 13.13882008 13.80423814 33.33333333 27.42276549 0 8.386742355 23.59375971 16.31289976 45.76982525 0 20.17381331 0 34.88597237 0 26.95383497 47.80826238 26.62377889 33.33333333 33.33333333 18.94096366 0 17.45963091 17.5260761 11.09527204 15.18907442 0 29.84333333 5.406666667 56.93548157 5.517993457 22.08623479 13.80042463

478 0 15.07102742 0 15.55706522 31.5504372 0 0 0 33.37089139 0 0 24.87713212 0 34.97218463 11.72259508 42.29749544 0 0 27.35198702 43.83989941 0 26.54692001 23.07277628 0 0 0 0 0 32.48445015 0 22.38494871 11.10573173 22.73258077 0 36.95090435 0 0 23.47590169 0 19.27289522 23.11425948 0 26.28745019 0 27.84732824 18.2467942 16.02564103 0 0 0 0 0 46.90614391 5.306511031 39.29177523 36.83501091 0 47.45427007 0 0 5.91 0 0 0 24.86170164 26.9418553 15.20957341

479 23.50061037 0 7.701605288 15.15277971 3.991547312 34.91214124 0 15.05243677 15.02997726 11.60417544 0 13.35240301 9.094448338 17.16304602 0 24.67731141 0 10.6159839 13.19939204 13.01224682 0 11.69922285 15.03338102 14.5094307 0 18.46962233 0 6.733590026 0 15.57830092 0 10.86425408 16.42909936 13.80964092 11.35754848 0 13.87659289 15.4832679 14.80303609 23.116899 4.98088685 11.89310961 0 0 10.57705363 22.36159504 15.33005211 8.435342122 0 20.57832948 0 0 0 0 0 18.63371209 0 0 28.67966825 23.45679012 7.54 0 20.34333333 0 14.88186114 0 0

480 0 12.8730742 0 0 0 0 0 0 0 0 26.08478749 0 7.027134086 0 3.911879761 0 29.23585736 0 0 0 0 0 0 0 0 0 0 14.62398504 0 0 0 33.33333333 23.50588235 19.43836268 0 13.02521008 0 0 0 0 8.14354035 0 0 0 15.28943404 0 0 7.176569185 14.80673555 0 0 13.09327167 0 19.28167631 0 0 7.168949772 0 19.0735568 8.622892182 0 17.04666667 8.223333333 21.43284475 0 0 8.370842711

481 0 20.61363093 22.63062656 25.0809945 0 22.89844509 15.99743308 7.577680628 20.30219513 32.47159876 0 22.55326077 10.1953602 14.64473756 12.126304 0 0 3.735598286 11.90379496 19.25694611 16.25693394 0 9.10250279 30.33930848 23.82828532 17.59429154 41.60411065 0 0 15.3543496 14.42629502 0 13.4508278 0 15.05026868 0 43.89018191 16.25072855 19.02062279 13.16216809 0 11.16963803 0 0 0 4.734754586 42.07448252 16.21189774 27.81149286 0 36.63540214 0 30.40813438 0 5.818852027 17.97173084 7.049833172 0 0 0 6.77 0 0 0 21.54703127 8.502132196 0

482 0 20.92411442 0 27.08921846 42.13105706 5.915675326 29.89845097 0 0 0 0 10.65987518 0 0 11.28753883 22.58110415 12.26035314 18.73887206 0 0 15.0070891 12.89920725 24.05446293 0 12.1592256 33.33333333 0 15.13558615 37.38655937 20.95524382 0 24.71732248 0 0 19.66272721 28.02884006 0 0 9.401596331 0 19.9050709 0 33.33333333 28.09848333 12.86975075 21.93758733 0 0 0 18.92853173 17.5662604 17.23614275 0 20.75036873 0 10.21118012 23.79283489 16.92270178 0 21.21881752 5.836666667 0 0 41.92205591 0 0 9.825021054

483 58.20382014 0 12.57579676 0 0 0 0 18.69976026 10.49072035 0 25.92766203 0 0 25.36073702 10.03088326 0 0 0 33.33333333 6.512524085 17.7035212 22.6974781 4.41575004 10.58352403 0 0 0 16.73636522 13.0952381 33.33333333 25.97471531 21.85473411 0 32.2584079 10.77270812 20.30812325 0 14.52312139 20.29480301 20.53276225 26.3878608 17.68173655 40.88435129 0 0 13.99355478 0 19.68301047 13.18804135 20.84200786 0 0 0 0 0 0 24.80980416 0 0 0 16.48333333 0 15.69333333 0 0 18.41684435 0

484 0 0 0 0 0 10.43488824 0 0 17.68081132 25.4613415 8.341401872 0 28.44115276 0 8.793670797 0 0 13.85710354 20.27236889 33.33333333 0 0 0 15.22813392 0 14.82315538 11.71326518 9.62462271 0 0 0 0 0 0 0 0 33.33333333 0 0 0 0 0 0 20.71752952 0 0 0 0 5.074569049 0 0 22.01858216 10.27119368 14.05165702 0 0 0 0 14.22528522 7.979901209 2.283333333 6.576666667 0 0 0 21.20579568 28.46644462

485 18.865606 0 38.4889502 18.60481841 29.61094212 0 0 55.28603628 0 0 0 17.58406235 11.57456922 33.33333333 5.856633299 19.8904555 17.01797993 40.51698931 0 0 28.00854377 32.33049361 19.23313153 0 27.78461791 3.620795107 9.406822472 0 25.36654647 6.746317355 33.33333333 0 0 0 4.645519154 18.20592134 4.243234213 23.23927816 0 8.439763002 9.872579512 41.16668079 0 0 37.56757468 16.70239077 40.48637317 7.2541166 8.637581324 0 4.499903269 0 0 0 29.63463516 0 8.115380476 43.39184306 0 0 5.87 17.87666667 18.82333333 7.867500156 16.19198312 33.33333333 11.85240635

486 0 0 0 0 0 50.63122117 10.19837623 0 16.34161686 0 7.558788439 0 0 26.66119209 19.39057031 0 0 0 21.42953838 4.70013612 0 0 19.8150805 0 0 0 10.92820513 0 0 0 10.58993956 0 33.33333333 0 0 20.43190526 0 0 16.55421793 0 0 0 0 33.33333333 0 0 0 17.65691853 0 0 14.81547619 20.24006167 0 0 10.97946288 25.72376702 0 16.41063155 33.33333333 15.42135038 0 12.50333333 10.81666667 0 11.78630206 0 0

487 22.93057385 12.7197024 26.30462648 8.25233883 0 0 17.33590026 0 0 12.42409587 10.72968929 14.07997425 0 0 0 29.28723391 27.51869448 0 0 0 0 18.67538515 0 34.6712558 10.13104175 12.11824669 13.59362812 16.24784616 19.54468803 11.23266638 0 0 0 48.30322942 13.67060612 0 9.056042202 0 0 23.85969281 13.42826244 14.33034784 0 0 16.39379578 9.424062138 17.43914432 0 7.669959326 47.73813493 15.21578833 16.09719058 40.99765054 18.22838312 0 15.3616025 9.540498442 0 0 7.988417646 0 0 0 12.52541684 0 0 15.07256513

488 0 12.40921891 0 20.9726298 15.55958812 0 18.29778065 0 19.53213432 0 0 14.08171592 23.13797314 0 28.60251974 22.58763026 0 15.74063151 0 36.19706035 15.62981213 0 9.278870398 0 15.05376344 0 0 16.59696811 4.606968039 12.37808951 0 20.09461008 0 0 36.19817072 0 0 35.89281673 13.03853032 11.73140224 11.35669623 15.65159678 15.24940001 12.61580381 8.903593716 11.395746 0 32.01748747 9.889016456 0 0 0 0 12.5829646 19.50886767 23.12215321 0 23.27482361 33.36782465 12.11451581 16.43666667 0 0 8.216173098 33.33333333 0 0

489 0 20.46025914 0 0 12.6984127 10.11977017 8.272058824 18.43652283 15.65252202 29.64296387 21.35767088 21.04111153 19.6238106 0 0 5.653576172 13.9671151 7.410805301 13.06096444 0 7.394099849 13.3974359 14.10020181 9.177777778 11.04306598 18.51017795 12.75396846 11.03462662 0 0 15.67571678 0 29.70995651 0 0 0 9.477208344 10.09405517 21.69022961 22.2742116 10.90598977 0 10.53291536 5.234850003 8.975851033 21.81190439 0 0 12.92260408 12.49132547 11.26716967 11.31475118 18.3230214 15.10495021 34.05818227 7.609566312 19.52269909 0 0 26.65410525 10.5 15.00333333 9.14 8.03600925 17.14135021 18.54189444 26.41272013

490 0 0 0 0 0 0 0 13.03505452 0 0 0 0 0 3.954574019 0 0 0 0 0 0 0 0 0 0 0 0 0 0 0 0 0 18.60516303 0 9.154647436 0 15.67377394 5.681819914 0 0 0 0 0 21.99395009 0 0 0 0 0 0 6.824374536 0 0 0 0 0 0 0 0 0 0 0 0 0 0 0 0 9.856194162

491 4.593856275 0 22.01504374 6.729828307 30.13382314 13.12202977 0 3.837891192 6.79087201 0 6.656895078 6.503340757 0 0 11.89377378 0 0 0 0 3.931642618 10.19840201 0 12.36216189 6.146887628 0 11.67196663 2.703577789 0 0 4.389491209 0 0 5.710889206 0 12.53199777 0 0 5.548435331 16.00251458 0 0 0 0 12.16918346 15.84315133 0 0 23.81031153 0 16.05164578 6.866991518 3.771566889 0 14.79897978 0 17.67871257 8.847284342 0 4.106666667 0 0 0 7.31 0 0 9.98269522 0

492 22.05059992 7.727713178 0 2.843323388 0 0 0 0 0 0 5.539869952 0 0 0 12.84818434 0 0 7.562479673 0 0 4.04290429 0 0 0 0 0 0 8.009219245 0 0 8.452660825 0 14.18336834 0 0 0 0 8.604206501 0 0 8.103331774 0 6.752396089 0 0 0 0 0 0 0 0 0 0 0 11.10734113 0 0 3.014633073 0 0 0 5.61 0 0 4.77712435 0 0

493 0 0 6.525706375 3.360759218 0 0 0 9.38091376 0 16.69087603 0 9.267836257 3.346132748 6.324625191 0 5.6455049 7.945405801 0 12.79484007 0 0 14.8986963 18.68116631 6.192515065 21.26396154 4.52982923 0 0 0 0 11.33866134 0 0 5.869132473 8.547955322 0 11.0519898 0 0 8.520437536 11.98280494 0 0 0 0 0 30.31704883 0 17.5646562 0 5.189997206 6.998777506 0 0 0 0 6.967088608 3.374605487 11.39956965 5.001819789 0 6.05 4.523333333 5.603574999 3.633025116 8.975336893 0

494 0 0 0 0 0 0 0 0 0 0 0 0 3.040668947 0 0 0 0 0 0 0 0 0 0 0 0 0 0 0 0 0 0 0 0 0 0 0 0 0 0 0 0 0 0 0 0 0 0 0 0 0 5.067338049 0 0 0 0 0 0 0 0 0 0 0 0 0 0 0 0

495 0 58.93895349 36.1933671 26.60350503 23.704154 59.12443874 33.33333333 52.11635604 39.55916106 27.69786747 27.79346338 26.82999258 0 45.25540892 20.485149 39.97477446 58.72126087 30.98489775 0 33.33333333 0 33.33333333 0 0 0 0 0 13.79582963 26.00643269 33.33333333 0 0 11.4148946 0 37.9628961 38.01119943 0 16.4648396 19.30342385 38.2579455 25.23000156 61.42083699 20.09860312 29.93970504 60.5811641 0 22.79798673 56.94983955 0 52.49585913 26.46634182 27.96107183 33.33333333 24.21287267 25.2205853 33.33333333 28.6036036 0 21.93376368 0 35.60333333 16.18 20.50333333 0 0 41.89786433 49.42784004

496 73.35554381 33.33333333 26.80762696 60.46258406 16.03985507 20.21130356 66.66666667 10.91736505 25.03417895 33.97461562 60.00977159 57.39883041 78.36360971 19.12469034 54.77289288 33.33333333 33.33333333 61.45262257 79.30418734 62.73502405 69.63947229 24.96277411 19.51590927 77.03180801 78.73603846 83.79820414 81.68836491 58.65744742 66.66666667 57.38011126 55.32800533 68.21801001 41.46971756 80.21147444 0 25.37053616 83.26619028 61.11823134 50.66415208 0 23.79974326 33.33333333 26.58093724 57.8911115 0 100 46.88496443 19.23984891 76.47470323 19.99741301 49.41976182 46.54621391 66.66666667 51.00279664 63.67207357 48.9879541 20.03457489 93.61076144 62.56 61.66484688 0 37.85666667 40.8 94.396425 91.58985053 24.35799644 23.47713917

497 0 0 0 0 23.61129822 0 0 0 0 0 0 0 0 0 0 0 0 0 0 0 0 0 24.91717352 0 0 0 0 19.53750371 0 0 24.88067251 0 0 0 33.33333333 0 0 0 0 24.8128958 11.2018628 0 11.33938325 0 0 0 0 0 0 0 0 0 0 0 0 0 26.36624473 0 0 33.33333333 8.573333333 0 0 0 0 0 0

498 0 0 0 0 0 7.542227924 0 0 13.22810973 0 0 0 0 0 0 0 0 0 0 0 0 0 24.52358901 0 0 0 0 0 0 0 0 0 0 0 0 0 0 0 0 14.35781787 0 0 0 0 0 0 0 0 0 0 0 0 0 0 0 0 0 0 0 0 0 0 0 0 0 0 8.485150986

499 0 0 8.458255827 0 6.510869565 0 0 10.71241944 15.38767825 21.63664088 0 0 15.24958859 25.34070153 0 21.04638731 0 0 7.900972591 0 16.11922141 26.80519626 0 10.6287893 0 0 15.6080573 0 7.326900647 4.897064198 0 13.17682696 27.22113029 4.764745655 7.623817474 20.94449047 0 8.264287232 14.02990948 14.0509033 19.68225566 5.245829675 13.23473021 0 23.57568458 0 0 0 5.960640573 4.630707541 6.989569596 14.72236986 0 9.985350912 0 0 9.181203835 0 0 0 0 0 0 0 0 14.78610711 8.753675639

500 0 0 0 11.49930878 9.963149993 0 0 12.44821092 0 0 7.579745236 13.91380827 0 21.63593725 0 0 0 6.802806314 0 9.67919918 0 0 0 14.4111329 0 22.85961684 0 10.57279381 12.69248917 24.09460458 0 0 0 0 0 0 12.17505312 0 5.286767062 0 0 13.83244504 0 0 0 1.495180682 9.594997313 13.09094035 12.77192982 0 10.90270172 8.45470565 13.26903231 0 0 0 0 12.07326755 0 0 9.236666667 0 0 7.135809114 0 15.65896554 0

501 26.52519894 14.26160338 20.55349195 18.81838074 6.146112057 22.9869631 51.27270346 12.24076884 0 5.841298741 23.1650285 22.30347349 16.49941353 0 25.06957297 18.16401469 11.76776205 10.40163099 16.18983829 14.20578381 20.46049803 14.49780336 33.04785144 0 20.01620746 30.77462668 17.03627372 8.967573925 10.94396195 6.19357904 9.77602108 9.115508202 0 0 21.75027228 8.581713463 7.039242632 23.70726431 19.45748565 19.0505676 12.69503546 0 19.30951563 7.024929094 0 0 10.09327812 15.53733623 13.29275624 21.85357828 0 8.310110754 35.47139947 36.2653634 0 0 12.97258123 35.26067075 0 12.94211131 10.09 5.38 16.84333333 16.2204885 22.45232407 0 16.05700904

502 12.148444 16.8999698 0 6.129012683 0 0 0 0 0 3.66091251 0 15.02648737 8.95458014 0 16.84147438 19.19032597 0 24.56800072 20.50853943 17.16505844 25.44870288 0 0 12.95430306 0 0 0 0 0 0 20.57290609 15.79418867 13.07722158 26.24923831 0 0 0 13.41021909 12.02749141 8.361914094 0 0 0 28.88980509 17.99587487 14.56571868 0 0 0 31.17618653 11.20615172 0 0 0 17.36139352 40.69423431 0 0 28.2524757 8.651481125 0 14.22 33.63 0 0 0 28.8549671

503 0 0 24.15013691 0 33.09054537 14.93908204 0 18.20166427 5.037111167 0 0 0 33.33333333 0 0 0 29.50190243 14.76238203 9.778887964 23.65413415 0 17.7214075 0 0 22.85989292 0 0 0 12.43064182 10.38219828 0 9.688115221 7.51835536 0 0 0 0 0 0 17.21517849 32.35918188 0 0 0 50.72496602 30.93135225 3.087595877 12.61129604 6.925438596 0 17.38285048 25.66717899 0 0 18.81823338 18.96053347 24.9026715 21.26006578 14.3258427 0 7.476666667 0 0 19.37803693 0 11.02201258 0

504 0 21.83214191 12.29227761 17.72024632 9.329981891 0 0 0 16.3106364 0 0 10.81794195 0 0 7.934665807 0 0 9.418960245 0 0 0 11.4932081 12.97396718 33.33333333 0 0 0 0 11.88999173 15.34650105 8.173195876 10.08512317 0 33.33333333 19.70272881 21.13381749 0 0 0 0 14.44917258 0 0 6.475593372 0 0 20.31471506 17.79599711 21.77357526 0 0 11.46473909 7.910613638 0 9.417221214 0 0 6.590980763 0 11.20071685 0 0 0 26.19752422 38.80510362 0 0

505 21.18488933 20.95547102 0 18.19884902 0 43.67970357 13.91336726 0 33.33333333 0 12.73632055 0 23.59023455 0 4.954549995 48.67376802 20.74036897 0 7.798296814 4.453335936 0 0 8.151922186 27.1058086 0 0 35.87557215 19.47040498 33.0052334 0 0 17.97339197 10.16152717 0 0 0 33.33333333 11.27415371 0 39.25418498 0 45.84623323 0 0 0 0 0 20.24239299 20.0405771 18.93481315 5.489662324 0 0 11.38991607 0 5.078357469 23.38190582 0 8.761469409 0 16.76333333 15.95666667 0 0 0 45.51859741 0

506 40.14146773 0 0 0 8.62859694 0 0 15.3735091 0 17.60681437 0 29.33670581 0 11.69739609 12.57587607 0 0 0 0 0 0 0 0 0 0 0 0 24.36575941 0 0 23.55731225 0 33.33333333 27.08207604 0 33.33333333 36.91132139 19.92311425 21.30584192 0 0 4.810681768 43.01893631 37.77686157 0 0 23.24005521 8.982165571 5.074561404 0 0 0 16.18832123 0 15.97193981 0 0 0 7.139513109 27.20125786 0 0 0 9.69353934 0 0 8.845719717

507 0 19.07172996 25.7123927 0 0 0 0 41.73584686 28.29622217 27.49203459 43.12010223 0 0 0 0 0 0 11.76814499 12.82479391 0 54.0907991 48.94525917 0 0 13.31712588 0 0 22.76053953 0 32.1898638 19.46833102 14.13270196 0 13.33535231 21.38399079 24.75161987 0 0 32.15454749 0 11.72294372 20.82043344 14.65741544 19.83281087 21.56385931 6.423486938 13.01861828 0 11.55975807 11.47975505 44.55781323 25.02322258 15.00694597 33.33333333 14.51509996 21.48308179 9.94886661 10.355854 0 15.32258065 0 17.32666667 27.69 0 0 22.31132075 19.03310989

508 0 0 9.18319642 10.02997889 0 0 21.26856457 0 0 33.33333333 0 8.601583113 0 66.66666667 23.67141599 0 24.13102383 22.27807472 32.8996436 14.67421359 0 0 12.49292586 0 43.80677375 35.89203999 35.07482079 0 19.03768193 0 0 12.75609566 0 0 25.57994707 0 0 22.08672087 0 0 22.58454106 0 0 0 0 35.62414429 0 0 0 16.55566698 0 13.41388859 12.15368739 0 0 8.787938901 0 14.45916115 13.59720465 0 0 0 0 21.3746019 31.65128401 0 17.27632429

509 0 6.979083929 8.108504399 17.60422356 32.84161375 18.39425129 13.54536471 0 17.02269693 12.06560645 13.39880348 0 17.62243845 0 8.952444792 13.97189132 13.85894272 0 0 16.1682749 0 7.34232187 33.33333333 12.1954221 0 10.47371649 12.01333333 13.86292835 0 11.79325324 18.45223368 10.45487514 35.90956257 0 11.58306105 12.19951585 10.54104952 9.598527774 9.767866458 16.11815484 6.189125296 14.69020653 23.01413263 0 9.715299804 10.96011716 20.65074014 11.73987172 8.561403509 0 10.46082053 7.666154348 0 19.01138721 23.91611212 4.995854063 28.79397484 0 27.92349444 24.68185221 3.57 11.27 9.23 0 7.091288304 5.489103719 9.932869958

510 0 4.996536197 0 0 0 24.76702509 0 0 25.91036415 0 0 0 0 0 0 10.4457482 6.235402748 0 0 0 0 0 7.799585386 0 6.922619553 0 0 0 17.18524353 0 0 0 0 0 0 0 0 0 0 20.9729349 3.20221886 0 0 0 20.07190871 0 0 13.39563863 23.26972373 0 0 0 5.796035904 0 0 0 6.688309223 0 3.669760247 0 12.07333333 9.426666667 0 0 0 0 0

511 16.86023544 3.003419277 11.21960842 0 16.90983913 0 0 0 16.01106331 0 0 39.51788542 26.92143341 0 6.565201729 0 0 0 11.0261294 0 10.32913117 0 9.744415542 11.25260981 18.71034381 6.873315364 20.3009973 9.509915853 5.753543993 38.33255123 0 0 0 0 37.74574917 0 0 5.09439146 0 0 4.337794186 16.35678697 0 4.78742899 11.19405887 0 14.904191 0 0 4.280655801 5.380791808 19.23681607 0 0 0 4.998175848 0 0 0 3.059654433 0 10.92 11.32666667 8.25446898 4.787731438 22.16320246 18.7983614

512 4.493597714 5.451701212 9.0071264 10.07636246 0 0 6.005972378 19.30812079 0 5.278270416 0 0 0 0 7.919087583 8.12455126 0 0 0 3.262902503 0 0 21.52528497 0 0 0 0 15.72119144 0 0 0 12.23278648 0 11.53055063 0 25.60574593 5.922214269 0 0 0 0 0 0 0 0 0 0 0 17.24595704 3.073103921 5.74978776 0 2.620856734 0 19.62277092 5.180744777 0 1.578823059 0 13.13794991 3.543333333 0 0 0 4.434411312 0 17.44319816

513 0 3.082784898 15.97596094 0 17.96577947 0 12.0145429 33.33333333 7.422969188 0 5.172792342 3.369352118 0 40.91496891 0 0 16.75084864 5.790954532 0 0.997346509 2.891210975 38.05748242 3.591524648 5.821506743 0 7.258849444 5.565551157 0 0 4.367276709 29.24273398 15.82568807 0 2.929783788 0 10.55668174 6.611504177 35.95045114 13.8936223 25.75973836 5.539308447 0 12.39474571 0 13.26142463 0 22.77875415 6.665491095 0 0 0 11.26126126 7.661423646 33.33333333 0 0 0 0 4.292343387 33.33333333 0 0 0 0 10.2433182 9.904891819 0

514 0 0 0 0 0 26.23859712 0 0 0 0 0 0 0 0 1.780139289 0 0 0 1.898896587 0 0 0 0 13.00769159 27.68186513 12.33474522 0 0 32.25553145 2.490641672 0 0 0 0 0 20.47112162 8.552511416 0 0 0 21.52775984 0 0 0 21.76189298 0 0 12.64069264 0 2.00572371 0 0 0 0 7.643921783 0 0 4.293513993 0 3.939967209 13.7 0 6.973333333 0 5.155538931 8.984016168 6.45032531

515 0 0 4.162351433 3.74934371 0 0 11.17954461 9.019607843 5.324940456 35.34705753 0 0 12.95581718 4.971047744 9.059608349 46.78922187 0 0 7.22859781 0 8.395666753 3.968926554 13.22902067 0 0 4.756665566 0 9.448462929 4.438900768 0 0 38.46321153 33.9310632 3.270554881 26.63183408 7.7275874 4.189930804 3.532320735 17.35319956 33.33333333 0 18.98986874 19.36805149 10.90738658 5.668960691 1.221403182 0 4.089800968 10.06360961 4.849605919 0 0 0 0 0 0 6.853446179 1.531891819 4.635783487 0 0 0 0 0 0 11.17013087 15.89013517

516 0 0 0 0 0 8.494334393 0 0 0 0 0 0 0 0 0 0 0 0 0 0 0 0 0 0 0 0 0 0 0 0 0 0 0 0 0 0 0 0 0 0 0 0 0 0 0 0 0 0 0 0 0 0 0 1.75826199 0 0 0 0 0 0 0 0 0 0 0 0 0

517 33.33333333 30.32991406 35.30874587 23.25697087 15.36755387 0 23.11308298 0 45.3306629 12.69438029 33.33333333 39.70173441 25.90317919 0 0 17.4545592 0 0 0 0 0 21.97395833 11.80804837 13.50635374 0 14.12527275 23.68080194 0 0 0 0 0 33.33333333 0 0 12.86221171 0 0 0 0 44.33461994 47.91926174 20.93858763 0 0 0 33.33333333 42.51573597 0 0 0 22.07207207 25.67190969 0 13.71056241 0 33.33333333 0 0 0 0 0 0 25.07886435 75.37900011 0 14.53497193

518 45.31283351 53.13564436 0 62.91732296 49.75682753 15.66104446 16.14781635 38.33893804 0 28.19790007 61.49387432 17.41102805 0 35.3413918 49.68797073 0 16.16968117 94.20904547 52.53898898 95.73975099 78.3839911 35.99963269 13.56373096 56.41183812 46.6851715 54.65115166 27.76778218 0 23.14088857 0 70.75726602 33.47831392 32.73560347 52.83840846 15.90585893 22.77665159 24.78082192 31.38285947 21.85301016 0 21.05829872 0 33.33333333 55.75928009 0 98.77859682 28.98372151 20.69264069 49.42070963 30.26022941 88.86942043 47.4298506 58.24977403 31.57507134 59.02274488 89.82107937 26.47988715 92.59577113 0 0 8.256666667 0 0 66.66666667 0 24.34931717 26.88300802

519 0 0 24.32620693 0 0 24.83899894 31.53904079 0 0 18.48239169 0 0 34.21957022 18.77259154 24.98799232 17.18591948 60.84406744 0 27.30738722 0 0 0 18.73838947 0 0 0 22.68486743 65.32042978 17.22589168 54.80953039 0 0 0 29.43070224 19.71655782 0 49.94301742 24.03997719 46.90016797 19.9339934 0 16.73408254 13.96528184 28.54590434 28.04175413 0 0 0 0 55.53068124 0 0 0 33.33333333 0 0 26.64502411 0 87.40211288 46.52909511 29.94333333 0 77.90666667 0 0 23.42844151 0

520 29.52328599 0 62.09859792 0 31.1901193 33.33333333 51.24258458 15.6022777 26.81508967 0 23.51604278 0 0 16.89418143 20.15514744 62.52369918 0 0 28.69623656 0 0 0 0 26.43062689 22.07155063 0 0 0 0 25.48776218 17.41579003 43.11714416 10.44309616 0 9.417040359 31.81271196 0 0 0 24.81654079 9.467455621 33.33333333 0 0 15.76783555 0 9.452339842 0 21.41724272 0 0 0 32.49371133 33.33333333 19.96801181 0 11.01538795 0 0 0 0 75.3 31.64666667 31.20262759 63.59838099 0 19.27064276

521 0 0 0 0 0 0 0 0 0 33.33333333 28.05404862 26.19013581 26.52671756 0 0 0 22.22876202 0 0 0 30.96118299 0 28.48269123 0 0 33.33333333 20.06096556 0 30.76697463 0 0 0 8.527683817 0 0 24.91775687 0 7.170806046 33.33333333 18.97720271 0 0 0 0 0 0 11.75018514 17.94900765 0 0 0 40.7579814 0 19.86807519 0 15.67857039 0 0 0 0 0 0 0 0 0 21.43235793 33.33333333

522 0 0 0 33.33333333 0 26.40496561 0 17.99098338 33.33333333 22.47821592 0 0 0 26.17375231 0 0 0 0 0 33.33333333 0 32.5964652 0 15.86873455 0 0 0 0 0 0 0 0 0 0 7.439869547 0 0 0 0 28.47613092 29.52196382 55.61273108 15.79061685 33.33333333 9.481915934 66.66666667 0 0 8.749726057 0 0 0 0 0 0 0 22.31794538 0 0 0 0 18.17333333 0 0 23.72781065 0 0

523 20.10727056 27.14803396 0 0 0 0 0 0 33.33333333 0 22.50839866 20.85622371 0 33.33333333 0 16.00460564 33.33333333 0 33.33333333 0 0 0 0 0 16.34970509 33.33333333 0 0 0 0 27.96090861 0 6.29711344 0 0 0 66.66666667 0 33.33333333 0 23.57574523 0 12.0281339 0 17.56549778 0 43.16867847 33.33333333 20.4537448 33.33333333 33.33333333 0 0 0 0 27.79759251 0 0 0 0 9.073333333 0 0 0 0 20.49718302 0

524 9.402931713 21.88701568 0 33.33333333 48.35118137 33.33333333 8.683662017 33.33333333 0 20.43048694 5.369875223 0 58.15630737 0 58.18795052 0 0 0 23.96962119 0 33.33333333 15.16989348 33.33333333 0 33.33333333 0 13.27236777 0 42.78721546 19.07099355 41.34947903 15.59679037 16.98771304 0 0 23.43360926 33.33333333 0 0 0 0 0 33.33333333 33.33333333 33.33333333 0 0 0 0 0 33.33333333 15.08581752 0 0 33.33333333 34.9702001 0 0 0 0 12.83 0 0 33.33333333 0 0 0

525 18.23984178 7.367876318 0 0 0 0 33.33333333 0 0 0 0 0 0 16.4391519 0 0 11.10457132 0 0 0 0 33.33333333 0 50.79793212 28.24541095 33.33333333 0 33.33333333 0 0 0 0 0 0 0 0 0 0 0 8.516792544 0 0 0 33.33333333 23.8514174 0 0 33.33333333 24.58360728 0 0 5.14683621 33.33333333 13.46525815 7.451607612 0 0 0 0 0 0 0 0 0 9.605522682 0 9.504017733

526 0 10.87767375 0 0 0 0 0 17.73105563 6.518243661 10.85511741 0 7.143197522 0 0 0 4.142967488 0 0 4.637096774 0 0 0 0 0 0 0 0 51.67368914 0 7.845571151 0 7.952732134 28.72890203 0 16.47642343 0 0 10.41229046 33.33333333 0 16.43356643 0 0 0 0 0 21.58314819 0 11.91609061 19.91532324 0 11.51772756 0 0 0 0 0 0 0 0 0 4.5 0 0 0 33.33333333 3.736227995

527 22.72666996 11.44631766 4.568068747 0 7.531621311 0 6.740420074 0 0 12.90284639 20.55163472 33.33333333 0 7.159581023 0 0 0 66.66666667 9.363712142 33.33333333 33.33333333 6.327413192 38.18397543 0 0 0 0 0 20.29790898 33.33333333 7.901397601 33.33333333 0 0 0 1.520621376 0 0 0 19.21333304 13.56895761 11.05393558 21.30519943 0 0 0 14.04564836 15.38432569 0 0 0 0 0 0 0 15.09244779 0 66.66666667 0 0 0 1.52 0 0 3.068285677 12.83615032 23.8293156

528 0 16.0359661 33.33333333 33.33333333 0 6.928367724 0 15.34234996 0 0 0 12.47710963 0 0 0 0 0 33.33333333 0 0 2.372150339 0 0 6.902706444 0 0 66.66666667 0 0 14.26233979 0 0 0 0 0 8.415576467 0 0 0 0 0 0 0 0 0 0 0 0 0 0 0 18.24751581 0.839622 0 0 0 0 0 0 0 0 0 0 35.46403908 0 0 0

529 0 5.237116541 0 0 12.92707802 0 0 0 0 0 0 0 15.31697507 0 21.65690205 17.32872769 0 0 0 0 0 12.5728948 0 0 0 0 0 14.99297753 6.147900931 0 5.372424723 0 29.01549152 33.33333333 66.66666667 9.899724073 0 49.08357016 0 0 7.432311278 0 17.54271648 0 0 0 0 0 12.87958853 13.4180101 0 9.244121506 0 0 5.913713911 6.461189208 33.33333333 0 0 0 0 0 0 0 0 11.9009754 10.32646258

530 12.2735939 0 15.59799909 31.22960351 14.57940992 14.12715517 29.39192686 24.80115688 0 0 11.87510549 0 0 0 20.5582394 8.315379673 15.49138124 5.372435978 0 0 0 19.94335007 46.15631122 10.76597761 9.482288828 8.152112035 0 0 18.5533348 11.25249264 18.65253411 21.32734815 0 7.170776256 0 19.43802241 20.06487017 0 17.37314628 10.01519097 0 9.5680054 12.67698387 27.67191578 0 6.033729759 18.28155819 9.937463871 0 10.56241427 0 0 11.39447402 28.07846202 13.94904702 0 8.350407995 23.17827758 0 0 20.16 4.593333333 0 13.82874976 11.58820547 0 0

531 9.555288462 35.57102338 0 13.62444625 11.67044215 0 0 0 23.79223273 0 18.93101661 15.50550026 22.45948811 32.32027687 0 0 17.469381 0 0 25.50382717 13.8093322 17.61481969 0 27.20412844 24.79582659 15.36892214 13.45059793 38.53458876 0 10.35021416 7.08711782 0 4.415775121 11.58025458 18.76696777 4.282683638 0 0 0 25.36435694 0 21.54412772 13.91780399 0 22.93971336 11.5700842 9.074028225 20.36066719 31.4552701 0 0 10.24545701 10.19588953 6.700698234 0 5.670956193 11.35701275 0 10.65273194 35.54063916 0 0 18.70333333 10.27357633 0 24.81942442 4.10768453

532 0 15.28750265 0 0 0 0 0 0 23.65122238 23.85291446 0 19.36601307 0 0 17.00441217 0 0 12.89800051 0 14.18104053 13.08729497 13.37643462 0 0 0 0 12.03607951 22.61347597 0 0 0 0 0 0 0 11.23505976 0 8.851479772 14.56899088 0 30.22486423 0 0 0 0 16.51083324 13.95261376 0 19.86255361 0 15.19482473 16.26917478 0 0 0 0 16.02958644 0 0 0 9.093333333 23.03666667 0 0 0 0 21.43687142

533 54.39307277 0 11.49816989 5.973412321 18.75392341 26.69530034 12.03795653 14.58365439 0 14.26671985 0 13.10079648 17.00877739 12.32583065 0 0 0 0 15.03157246 0 18.63711002 0 0 0 0 25.79862172 0 0 22.49472444 26.76684961 16.90518784 11.04021655 8.889238515 21.36255708 21.50454953 0 20.96559082 20.86145934 5.585094297 8.338758681 7.698567708 0 10.47066409 20.51660517 23.73914496 0 6.656804734 20.18163471 21.17534569 23.11688747 0 0 6.406644998 20.50180062 21.04085463 23.36717548 0 0 10.44018553 0 0 0 8.406666667 21.38583958 13.01874163 0 19.10237647

534 0 18.44835667 0 11.65807793 0 0 0 0 17.06075534 0 0 0 0 12.93215012 33.33333333 52.90992316 14.91185527 61.29423069 40.74215924 17.58883039 0 0 0 0 0 0 4.876349704 0 0 0 16.8436314 10.42341977 0 21.75307875 0 12.39061257 0 0 9.310543776 25.4797702 0 0 0 22.36377302 12.66155089 13.11563529 0 10.10037311 0 0 18.35279842 0 22.9890062 0 10.64304225 19.41770527 34.69065251 36.90879026 0 0 0 0 0 7.628729647 41.65173137 26.32285791 13.86501517

535 23.77804487 0 33.33333333 0 40.51094891 20.54396346 12.02306195 19.45246766 20.15275906 15.31762569 0 13.96732026 42.0692515 10.04287245 0 0 0 0 0 5.833683915 14.50737005 0 8.380007468 21.71253823 0 3.57588122 18.88606163 15.57107642 0 15.52559111 0 0 0 16.37779452 33.33333333 16.6356795 0 0 12.12121212 0 0 15.25804404 19.41552934 16.63097787 9.594188377 10.50520059 43.64002468 0 0 22.77091907 27.19017094 34.43213936 23.1374438 0 0 25.91572991 0 24.41944609 41.51140122 39.17761279 0 6.08 10.40666667 9.888823874 12.47657296 21.33692241 0

536 0 0 0 0 0 14.53663793 0 0 0 32.0150246 69.1938779 10.16675397 0 0 12.77509393 25.01795366 0 0 18.30176087 0 11.74589936 0 14.19176186 17.75 0 16.56731247 15.21314489 0 42.76217637 15.99395314 16.4281455 32.86596273 19.04516129 0 14.56636557 0 25.22563867 0 0 0 42.95316603 36.30886027 0 0 31.06540242 16.82250009 0 13.15169862 0 18.72126742 14.98053491 17.06415855 19.50884796 17.44291016 13.08985717 0 0 0 0 0 4.59 3.693333333 0 11.94749376 0 0 11.89646191

537 0 4.94757536 17.73533424 5.839416058 0 0 0 33.07724308 0 0 0 20.23253685 6.250946539 13.27040533 16.32892117 0 15.86395233 20.43533283 0 13.31652533 0 15.71851365 20.49855839 0 65.72188458 0 0 12.56100149 16.1897644 8.765484433 14.68079922 15.29916261 33.33333333 16.95553881 0 13.89531093 33.74390033 24.48185356 34.39788231 22.94836007 19.12340203 9.970216298 20.65634947 12.81672817 0 0 0 26.26816249 22.53839799 0 0 8.197369722 0 0 0 0 29.5723403 0 22.68060139 13.73818136 0 7.826666667 0 25.04678706 0 0 15.36063364

538 0 25.74554194 21.83516345 25.66636943 0 19.42740286 46.54705466 0 9.68211095 0 0 0 7.588637777 19.10846458 0 13.7567435 27.4282686 0 25.92450743 23.57609267 0 0 0 22.56735572 0 30.53715042 15.0063857 0 0 0 0 0 34.31649174 0 11.8287838 0 0 45.80520733 0 0 0 0 0 0 0 25.44201682 0 0 0 0 24.28167099 13.79170057 0 9.362616087 31.67676502 0 0 10.85455733 14.71507992 11.54356669 12.59333333 10.06333333 20.24 0 7.838018742 11.31021945 14.23095686

539 0 0 0 6.008674495 14.48527561 4.66954023 0 8.085477988 5.66091954 14.5477154 0 7.661079099 4.622898682 0 0 0 8.835161555 0 0 0 28.2129934 33.34688197 10.77336107 0 0 0 20.53138063 10.71985737 0 11.3454149 9.402584112 9.043890178 0 4.8 0 22.12263119 0 0 6.643130332 7.853563134 0 7.350746269 22.86266925 0 0 0 8.394970414 0 4.96843261 24.82851178 0 0 6.36769348 17.91351288 9.600433918 25.62843315 0 4.638928742 0 0 16.46333333 5.573333333 3.04 0 13.42672982 16.21057581 0

540 0 0 0 0 0 0 0 0 0 0 0 0 0 0 0 0 0 0 0 0 0 0 0 0 0 0 0 0 0 0 0 0 0 0 0 0 0 0 0 0 0 0 0 0 0 0 0 0 0 0 0 0 0 0 0 0 0 0 0 0 0 0 0 0 0 0 0

541 8.150523118 30.57721311 33.33333333 0 0 33.33333333 17.02068292 20.18450714 16.29148294 12.4235645 0 0 0 14.81951495 0 0 0 28.12939522 0 0 8.763086468 0 0 8.935191103 27.24943597 13.43157895 0 9.307069548 0 21.12549673 22.99155001 26.3626112 17.32593782 0 15.86245147 0 0 22.07415001 0 49.24588086 0 23.4772979 38.71766648 0 25.86705745 0 25.59839825 15.854535 0 0 0 12.37498134 0 25.88059569 0 0 15.37130624 0 33.33333333 33.33333333 12.68333333 0 0 0 0 4.808087933 0

542 55.80470767 16.70948711 20.10516548 20.60127112 18.77072437 20.66556785 33.33333333 33.33333333 28.36156134 0 15.36692341 50.58213482 66.69272131 18.22222222 85.04256966 42.43381108 33.33333333 46.70349125 83.11996988 0 40.01812083 53.92521915 33.33333333 47.07333333 33.33333333 19.79895889 0 0 55.09898341 27.57369615 0 0 19.03443088 61.80256581 23.25445921 52.31615518 37.04390768 36.87500173 59.80282738 33.33333333 62.95820145 59.38856016 0 12.30526523 47.92709299 0 33.33333333 43.39397805 69.24368628 66.66666667 100 58.05452292 100 8.125149926 16.87185712 89.39820399 0 91.55500705 0 0 7.293333333 58.99666667 46.13333333 45.865452 56.5163109 17.19703485 55.07293946

543 0 0 0 60.1298907 0 18.89133358 0 33.33333333 25.58447003 38.14567448 40.80572183 18.61224827 0 21.37877965 0 18.35714527 0 0 0 33.33333333 0 27.0877849 0 0 0 24.69843349 17.52214878 20.60778727 14.27115189 9.417446423 54.46337556 26.29664276 12.6040027 0 0 0 20.10056414 0 16.54253558 0 0 0 42.18950651 33.33333333 0 66.66666667 2.183758298 6.511427339 0 0 0 0 0 0 54.82683778 0 33.33333333 0 0 58.39859358 0 0 0 25.99388379 0 23.52674729 33.33333333

544 12.89233885 0 23.07204592 0 33.33333333 0 25.25920166 0 0 20.90976883 0 0 0 18.51381839 0 0 66.66666667 19.96317541 0 60.82177821 19.59561308 0 48.38223799 13.91622992 0 0 33.33333333 20.26515152 0 0 0 0 0 38.19743419 10.22370124 33.33333333 11.95743063 19.33788342 0 0 0 0 0 54.36140143 0 33.33333333 0 0 0 0 0 0 0 16.91430004 0 0 14.59785457 0 56.84195646 0 0 0 0 13.43728101 31.48590578 0 0

545 0 16.62384623 10.10069767 0 31.29708232 0 9.856649576 0 17.04185039 0 17.96640993 0 8.690351794 0 0 0 0 0 0 0 19.02115376 0 0 19.59333333 31.34870559 0 20.65832867 0 0 23.91588691 0 33.33333333 9.083832044 0 0 0 0 0 16.79079775 17.4207858 9.791484398 0 0 0 0 0 20.58135238 0 23.51786425 0 0 11.10986714 0 40.49560151 0 0 0 0 0 0 8.273333333 0 0 0 0 4.896255804 0

546 0 15.87997692 0 6.536775965 0 5.980692253 0 0 0 13.74327005 0 14.72108506 0 0 14.95743034 10.92037731 0 5.203938115 0 0 7.052932761 0 0 0 0 19.90175439 0 24.02626379 19.06218145 5.759637188 0 6.970722132 3.4405217 0 16.69003479 14.35051148 13.23276919 10.45378151 0 0 0 7.278106509 0 0 13.32715477 0 0 10.96737099 0 0 0 0 0 0 0 10.60179601 26.57342657 0 9.824710206 0 9.753333333 0 3.346666667 0 0 0 0

547 5.771153908 13.23505848 10.26128742 0 0 0 0 0 4.971771991 14.77772214 19.21132847 16.08453185 14.39684198 15.11111111 0 13.31247828 0 0 16.88003012 5.844888452 0 12.74144752 8.342908209 10.48191231 0 8.634899842 0 12.72554606 11.56768325 12.2078366 10.34178333 7.036690573 0 0 26.72217266 0 10.34587609 11.25918332 0 0 9.841269841 4.422041471 7.66831774 0 7.46627588 0 12.94124946 8.770234356 7.238449477 0 0 0 0 0 16.46147622 0 0 0 0 8.268073091 0 0 2.56 14.7033832 0 6.458614899 11.59372721

548 0 0 3.127470186 12.73206222 16.59885998 0 6.456000837 0 0 0 6.649616368 0 10.22008491 0 0 14.97618806 0 0 0 0 0 0 0 0 0 0 28.48618922 13.06818182 0 0 0 0 6.92356347 0 0 0 0 0 6.863839286 0 17.40904431 0 0 0 0 0 0 14.50245426 0 33.33333333 0 0 0 0 0 0 0 0 0 0 13.93333333 0 0 0 0 0 0

549 17.38127646 6.974418157 0 0 0 21.12907298 8.074131674 13.14882619 7.748863307 0 0 0 0 11.95455368 0 0 0 0 0 0 5.549093102 6.245548433 9.941520468 0 8.068525104 13.53437444 0 0 0 0 12.2032911 0 31.58771139 0 7.247180625 0 7.31945226 0 0 0 0 5.433993966 11.42450926 0 5.412418906 0 5.361908281 0 0 0 0 18.46062859 0 8.584352832 11.83982888 0 10.12407929 8.444992948 0 0 0 0 0 0 11.99778332 43.11325922 0

550 0 11.13924051 24.50549451 6.670896785 0 10.69602585 0 13.34745763 16.10405083 12.71658138 0 36.31688619 14.72038708 0 0 0 0 15.98776297 0 0 20.70059807 0 0 0 10.06058262 35.82744372 0 13.67168528 0 0 21.82455431 40.50089077 0 12.87112291 12.88396271 12.26188383 0 20.51625132 0 13.51645207 0 0 0 12.49150133 0 23.80066833 14.9747097 10.23855386 14.00457156 9.680045303 35.70371751 0 12.37706439 19.53360241 0 12.05553765 0 19.54069846 26.89201878 14.14721013 5.576666667 33.83 0 13.40977606 26.39616887 0 0

551 0 0 11.42256745 20.86810757 0 21.16667158 42.49590917 16.00899382 14.45568493 33.4951926 26.48176292 0 13.49527665 25.69229778 18.02220501 21.43092851 29.4555842 28.20010789 25.59374217 45.30838516 0 0 8.865905115 44.93459201 0 0 29.99103155 0 20.96445101 37.15259061 0 0 23.43072765 38.40723467 0 49.82543183 37.31199895 0 0 66.66666667 15.66289931 31.23873471 35.47284002 9.73106341 8.014973412 22.60575793 8.428571429 16.11903036 17.42999783 0 0 25.17870259 0 20.36628568 0 30.99814574 51.91432097 24.61112468 21.28928171 31.48753836 0 0 6.36 18.42338745 0 0 39.65920626

552 21.78082192 24.74820144 0 0 39.79156034 0 0 0 0 0 0 0 0 0 33.33333333 0 21.85271001 0 23.38148053 24.5691467 11.98612054 0 14.17910448 0 0 0 0 0 15.23186034 27.19830882 26.13706779 0 0 0 14.9457861 0 0 16.41037411 56.0326879 0 26.08321501 0 0 20.77473958 35.79758096 0 0 0 20.44800225 0 0 0 25.05665369 0 18.89839701 0 0 0 0 0 7.543333333 0 35.15 0 7.773109244 0 25.45260916

553 30.20860106 5.209860093 15.01369657 36.52805154 0 13.56607384 0 11.38344227 33.33333333 13.42580124 0 0 17.38461538 0 0 49.56122604 0 0 16.84293834 0 0 19.42083727 0 0 66.66666667 15.99935291 52.72456923 0 15.87261362 0 12.53237319 8.563278612 24.49212272 13.98794714 12.05911415 0 13.2847038 22.75025278 0 12.81545064 0 0 9.558573028 13.26410779 0 26.82283704 6.613095238 21.34870364 0 41.48593801 13.06107923 0 3.816416156 0 19.6402022 8.693200129 0 15.32382847 27.59450172 19.1861232 18.56 22.26 0 17.07023506 11.96736674 15.2991453 0

554 0 0 16.8936528 0 26.4536174 10.08194493 7.680334093 0 11.14716549 0 33.33333333 14.68345291 0 13.60089186 0 0 18.36061065 19.45405613 0 0 0 25.5186054 24.46742822 0 7.969193091 15.0860517 0 19.66164805 0 0 0 12.05062157 0 0 15.84255553 0 0 0 0 0 0 0 12.53206703 0 13.23385996 0 11.51243143 0 0 0 11.9991739 10.18538248 0 40.73135704 0 27.54740342 0 0 0 0 0 0 0 0 0 30.15894217 0

555 28.4949858 14.89009335 15.75091575 0 0 19.6310293 0 59.26010629 0 14.34039391 12.24158391 33.35951324 16.10404406 50.73607458 28.29707225 0 6.653985305 11.31925237 0 21.35828151 33.33333333 29.57386364 20.31594011 25.39102173 0 0 0 66.66666667 7.986763259 17.98345477 0 10.79056585 37.60941007 28.259432 18.38754723 27.53953855 12.63601871 14.13816846 33.33333333 0 35.08479849 45.36196378 11.88811189 0 0 0 42.99452409 17.21430297 19.32876178 0 8.58239649 4.718417047 2.375208759 0 43.32087903 0 15.65905404 13.79263487 12.04405163 35.1791283 17.04 7.676666667 14.34 19.92355728 0 12.0697935 0

556 0 16.98423273 0 0 0 0 7.958550983 0 0 18.83108016 0 0 0 0 15.31112832 12.72659853 0 19.01250265 0 0 7.233914159 13.91249607 19.15422886 0 0 5.388280616 0 0 0 0 20.80096014 0 0 6.474263283 0 0 0 12.81708201 0 0 0 23.39930151 0 7.57772421 17.63522575 0 0 16.78273324 15.9033355 0 0 0 41.61001297 0 0 0 0 0 5.738831615 0 0 0 0 16.26309827 12.7334267 0 0

557 0 8.585131894 16.41367292 14.28413121 26.87546423 24.85825451 33.33333333 0 24.95976542 0 6.851570415 7.108341811 27.72218344 9.970735786 0 0 11.48062332 0 28.69583277 8.764186633 0 0 0 17.50644481 0 27.69887105 7.412790698 0 0 11.53062129 7.196265543 20.87028647 0 0 8.390256476 0 11.3990143 0 10.63397876 7.001430615 10.80465809 0 23.77476031 12.55859375 25.31835992 7.939053729 0 0 0 10.21557395 11.3380861 33.33333333 0 12.31374519 13.69313113 0 22.2963488 8.722208649 6.441314554 0 0 6.416666667 2.95 0 0 39.29772787 30.62524615

558 0 18.44323999 0 9.18358714 6.87935803 0 8.531872425 0 0 0 21.09174942 8.531805849 0 0 5.03626108 0 12.19648652 6.026317995 0 0 14.11329863 0 0 12.16794144 0 0 0 0 0 0 11.50877902 7.224356735 8.841210614 0 17.4907778 0 5.319634703 10.58308055 0 0 7.250118325 0 6.773647727 23.60226992 0 18.83168296 0 18.29667592 12.88533109 18.67793604 7.584856978 26.58416454 14.76464403 0 4.447390623 0 0 18.00950486 0 0 0 0 0 14.90994588 15.79555154 0 0

559 19.51559122 0 0 12.46522577 0 0 0 0 0 7.190950714 0 0 10.57349338 0 0 16.28124692 0 0 5.486006187 0 12.63273526 11.57419763 13.01739322 0 15.30355762 0 9.871608527 0 39.94431177 6.135024513 0 0 5.626528948 0 0 10.37314579 20.04862953 2.784790758 0 0 5.114310781 0 0 0 0 0 15.47666812 0 0 19.9405067 11.7306898 0 0 7.055009692 0 20.70571306 10.13027619 0 0 0 0 18.54333333 4.383333333 0 25.3343769 3.174391167 4.262938435

560 10.08542952 0 5.596389426 0 0 14.90317784 0 5.304876341 12.06428248 0 0 0 0 0 0 11.25183309 0 8.013415893 12.82805867 13.90746189 0 9.19921978 0 0 31.31057105 39.38817011 24.22411694 0 0 0 5.647049791 0 6.882363132 28.87636603 0 0 0 10.07322101 17.79448622 14.29590972 0 0 0 16.95177424 0 0 0 6.140512631 0 0 0 0 0 0 15.15044456 0 0 0 14.46196986 0 15.68 0 21.56 0 0 0 0

561 0 12.16205313 0 7.933224147 29.57430991 0 29.61308275 8.022228555 0 6.678849584 0 14.16686475 0 0 0 0 5.284903356 0 0 0 48.76451232 0 0 0 0 0 0 23.61273885 0 25.29873506 4.161393359 0 0 0 15.44839726 13.04601729 3.990571726 21.91454828 0 26.09736629 0 38.04772141 6.47507751 0 27.13630529 11.03671465 15.33930969 0 6.951151039 9.083483793 15.48816151 10.97996489 0 0 8.805123771 0 0 1.468717474 18.0875576 27.29089837 8.43 0 0 15.74358506 0 16.7373996 0

562 17.74375583 6.204796346 14.55604076 0 0 8.959590419 0 0 13.15359477 0 13.57537726 30.26686203 0 23.82023726 32.19990407 0 21.80506337 4.991007194 0 0 0 7.320795857 8.047567283 0 0 0 0 13.69925897 0 17.55935092 0 13.09181153 0 0 0 0 0 0 11.88122742 0 4.152477205 8.992248062 0 5.422907707 0 0 12.08796714 0 0 0 12.14462605 0 9.776207303 12.52096098 0 0 27.82423314 16.90962099 0 0 0 6.253333333 0 0 0 12.28273465 9.403357607

563 0 0 0 0 12.03915757 7.798612306 14.11077887 21.19826594 12.6066307 0 50.6619594 0 25.41237242 18.72834373 0 9.421105276 0 16.78209166 20.76793249 0 0 33.33333333 29.30266425 22.86778289 15.66522539 0 5.733649329 0 27.24693215 14.27689891 18.94333333 0 4.274948025 28.87764755 0 17.7029361 0 33.0573341 0 0 4.311073542 0 0 10.48012865 0 0 0 8.817858955 13.3425961 12.31967318 0 11.9444828 13.90034609 25.35944836 0 32.07687867 0 0 9.39067146 27.43273824 0 0 0 0 53.80130244 0 11.45859395

564 14.28123038 0 0 0 0 0 0 0 0 36.70690347 0 0 0 0 23.95631382 33.33333333 0 0 9.803018675 0 0 0 0 0 0 0 6.489167319 0 0 0 18.75824277 0 0 0 0 0 0 9.641723356 0 10.30903515 0 0 0 0 0 0 0 33.33333333 17.19020026 0 0 0 0 0 8.926850635 0 8.417904594 0 12.37995093 0 5.85 10.66333333 0 0 0 5.941249483 0

565 15.58957751 0 61.07027724 47.89100861 0 0 0 0 21.26905085 0 0 14.159395 40.91354436 14.6049896 19.13553743 3.719504066 0 0 27.97009943 0 40.12727273 0 0 0 0 12.54027581 29.02915777 12.44843906 0 0 0 47.43728154 66.66666667 21.94210609 41.00889383 35.54472076 86.05067754 0 32.28496169 29.7876379 46.89296569 20.14049672 76.84633119 25.64873116 15.35558668 56.09028166 39.23938984 27.1928207 11.12109302 33.33333333 0 17.01969528 33.33333333 0 8.903020668 33.33333333 13.08250573 32.22827905 15.24577573 29.44508429 0 4.6 13.84 0 22.94948839 8.949429829 48.38106825

566 11.6911881 32.17142465 18.77729258 0 58.38653252 0 14.62516055 29.23713879 20.17973856 0 28.52823932 22.24040964 0 13.39818418 0 0 46.83834819 37.69042552 16.0654899 86.09253811 0 14.51315304 25.28576605 33.33333333 19.69087024 31.16020102 0 41.65982278 26.31907783 15.77398242 0 0 22.17602218 0 18.27309237 0 0 0 0 0 29.02225979 8.771929825 0 0 50.47418335 0 33.33333333 13.57686454 19.99073723 7.27052215 72.36721243 22.35336844 42.99011328 30.37714097 28.70984192 0 19.09453437 0 30.43407442 0 20.95666667 9.13 19.62666667 73.03153818 5.80039376 0 0

567 19.05210295 28.72478718 0 25.40010919 0 34.42028166 41.65097783 23.47981962 20.72670263 21.84957899 0 0 0 0 10.51044878 18.36199618 0 16.67698658 12.56540084 0 11.10821495 26.01253748 17.54293726 43.79888378 33.33333333 0 11.09370921 0 0 0 7.331404809 25.86073589 0 0 0 0 0 12.43335466 0 13.19886766 0 0 0 41.49645825 0 0 0 0 16.14313307 24.24984954 0 26.78182712 0 31.74244969 0 34.58978799 24.91542874 6.497923845 0 15.8312791 4.69 10.11333333 12.84666667 0 0 21.05059869 0

568 0 0 0 0 0 0 0 0 0 14.90651193 0 0 15.32284729 0 14.19779591 0 0 0 0 0 0 0 19.82106516 0 0 7.518150964 23.43019943 0 14.01909349 8.03459827 14.39 0 0 20.30388032 10.20937558 33.70632586 9.958750737 12.87981859 38.03932467 6.31118329 0 15.79281184 0 0 0 32.87300368 0 0 15.26108928 0 0 0 0 0 20.22485034 0 6.66539343 20.48880151 0 0 0 2.683333333 0 11.22487675 0 0 14.48037421

569 11.55671571 20.73693869 0 18.77565805 0 33.91833778 0 12.75767075 0 19.85815603 7.234424025 19.16646858 18.35123593 29.44824523 0 23.91222806 26.07168508 15.84607314 0 0 0 9.620960515 0 0 0 9.393202094 0 8.57974035 32.41489652 19.05643442 30.76857594 13.61017104 0 0 15.06024096 0 0 0 0 0 15.62122377 8.25479215 16.6785913 0 7.033924681 0 0 10.93860984 0 13.74313801 0 10.92066146 0 0 9.279868103 0 0 22.40665713 0 0 4.54 7.973333333 6.143333333 0 17.44881541 35.03858776 16.27660599

570 0 0 4.751020408 0 0 0 4.9766057 15.97683341 0 0 12.37876468 13.07529366 11.74867377 0 10.67718964 0 0 0 13.98946435 19.02842251 43.9186876 0 0 9.869111142 0 0 0 0 0 0 0 9.72285555 0 0 0 0 0 14.06423034 0 0 17.08390418 12.41011813 0 14.31808817 0 0 0 0 0 4.430716796 0 5.681384885 0 0 0 15.37742151 0 0 6.503121719 0 15.28333333 4.97 0 9.303070333 9.96623404 9.55165692 4.603056938

571 13.49893363 17.57804632 26.86474965 5.893082643 12.61146497 0 0 0 24.38297535 0 0 6.808343172 0 0 0 0 0 5.273948392 12.14260625 10.69574247 0 6.791447326 18.98105002 16.64973704 0 10.93474427 8.059124621 0 10.5596912 7.229669348 0 12.8432271 6.517388718 0 10.14866204 6.981981982 21.0576693 0 11.04536489 0 0 14.14308465 6.174819567 29.39246094 20.74390969 0 40.34140223 23.66281725 33.33333333 10.71819176 15.76372922 0 32.46753273 0 0 8.149273894 0 0 18.91630382 16.6159861 0 9.903333333 0 0 0 0 0

572 0 0 5.066666667 0 27.6305445 4.746723344 0 8.750270739 0 0 0 0 0 15.22461919 13.52385239 7.689537187 0 6.31212409 0 7.227903068 0 9.1966759 0 0 23.84442302 18.52796958 7.106124357 11.65995853 0 9.80952381 17.67458182 0 6.030744974 0 0 18.56135912 10.66368382 0 0 29.33952944 0 16.43192488 0 0 0 0 0 0 0 5.552597011 0 0 12.51235762 47.84663568 15.08521086 0 13.82554376 0 9.784075574 15.50574436 5.176666667 4.57 39.68666667 0 6.205288964 0 0

573 0 0 0 0 0 0 0 0 0 3.627619594 4.938744257 8.72328134 15.71027799 7.613314448 12.75815979 11.64859605 8.073424662 0 0 0 0 18.65615616 9.178990311 0 33.33333333 0 0 0 25.12003271 25.01318983 9.173017217 14.76770073 0 16.39599844 23.18467129 10.29546496 25.42226072 10.86186541 0 16.32059801 33.41029399 0 22.88425555 0 0 0 12.75694444 15.41545511 8.231406377 23.53500373 0 0 0 0 9.830339321 8.134676825 8.093423652 14.54525999 2.348196033 0 5.426666667 8.983333333 0 6.697819315 0 16.54276935 0

574 0 17.90851976 11.01566743 0 0 8.717454969 0 0 20.73335131 0 66.66666667 15.26957891 14.86835061 0 7.878622942 0 0 0 0 0 0 0 20.43518519 16.10343412 0 0 0 0 22.77364213 0 10.0748263 0 0 0 0 0 0 0 18.79063309 0 0 0 0 0 23.79350843 0 0 6.232139362 0 5.529225908 0 0 18.88787464 0 0 0 0 0 0 0 0 0 0 0 0 0 23.9315023

575 59.47677583 0 0 60.77358402 9.140628715 0 29.48518676 31.01695572 4.657061141 0 0 0 0 59.05335222 0 0 23.81614262 0 22.3580377 0 6.804492919 0 0 22.21161192 21.44005143 0 50.17637809 66.66666667 0 0 8.336257652 0 13.71356881 16.30758652 0 31.95333052 0 33.33333333 0 18.71458481 0 19.19024868 33.33333333 19.01524516 39.98049119 33.33333333 13.06079108 0 0 0 0 57.30718557 0 0 33.33333333 10.46426186 24.63848979 0 0 14.27073091 21.18333333 10.40333333 0 0 15.32284319 22.64204545 0

576 0 37.07246417 33.86429403 0 0 48.01983705 32.1537546 0 13.74253828 29.93764124 0 44.86809166 0 0 19.80948095 46.85392199 0 28.05938494 17.35126444 32.27012576 33.00898075 65.35572062 24.15434302 0 0 0 0 0 6.35551142 42.00445764 24.16031612 31.53044119 0 16.93733489 33.33333333 0 34.94531355 0 55.62130178 0 0 0 0 7.426320397 0 66.66666667 0 9.036785031 33.33333333 17.08591566 56.31954523 27.65194845 0 18.42086459 41.75111648 0 35.75108621 28.25353241 23.54925776 0 0 7.823333333 13.99333333 31.62728188 0 22.31246179 62.06360973

577 22.04791786 0 0 33.33333333 38.88958325 0 0 44.25594014 12.59998203 0 0 0 24.33936429 0 35.35269429 0 0 0 0 0 0 0 14.35228332 27.80531771 0 37.20395282 0 21.6733748 15.32187395 8.320143505 0 0 54.11853297 33.33333333 33.33333333 0 0 0 14.54270025 0 49.50580183 16.90140845 0 0 0 0 0 21.35625495 13.82344105 0 27.91672554 0 29.21758417 0 0 25.19865651 0 57.2012076 14.41702951 35.77994966 0 0 8.356666667 21.42403051 6.357497098 0 9.401831033

578 4.976372684 15.42481358 6.998207885 0 11.72777857 24.61587836 17.45356586 0 0 33.33333333 0 0 33.33333333 0 0 21.68473728 54.07199835 60.35454258 22.92008704 14.30491082 16.26783873 0 0 7.36078807 0 33.33333333 0 0 19.86924859 0 7.322493859 18.92384861 19.61976452 0 0 0 0 0 0 23.09973129 0 20.9232152 18.29457364 25.82103969 0 0 9.602111246 0 0 23.35001953 0 0 0 18.37837838 0 25.18405944 0 0 0 17.82758898 3.463333333 18.76666667 0 0 27.12804437 21.71215942 0

579 0 12.01615618 11.43939394 0 0 13.90010627 15.93088708 0 23.8840919 33.10140583 16.0158244 11.25541126 0 18.10871414 0 12.12320749 14.03843436 0 11.23854023 16.47289536 0 0 12.89814815 0 21.38219222 0 34.65837293 0 0 7.623015873 23.25850704 12.21192682 0 17.02574681 0 32.20786342 7.911072614 41.74057092 0 12.52555646 0 0 19.31301791 4.026845638 15.48209069 0 24.238751 24.2965483 11.2784859 9.798329599 0 9.359481099 6.914650844 15.35412134 0 7.491649967 17.69145658 0 24.48201558 0 8.996666667 0 5.903333333 30.94779796 35.02009234 7.23890706 0

580 9.425438596 3.931146778 6.760257374 13.0915928 0 0 0 0 0 0 12.94389258 20.33116499 0 0 0 0 0 12.55156766 0 0 0 0 7.421690007 0 0 0 0 0 14.97339438 6.486823639 8.400082152 0 0 6.97167756 8.684582744 0 19.10120465 24.92169258 0 0 8.651214357 41.85483659 0 7.25220695 0 18.06335493 0 9.183743876 17.44503619 0 0 6.385281385 3.123673712 11.17149758 0 7.013906884 0 13.59518916 0 11.34519573 0 4.45 9.92 0 0 0 0

581 7.132434483 0 0 0 0 13.29276952 0 33.33333333 0 0 0 0 0 0 0 0 0 0 0 0 12.76421023 10.57552167 19.47721105 0 20.36809097 0 0 0 0 0 0 6.651774634 15.29814272 0 12.59421347 0 0 0 14.20741066 6.165902141 27.70847012 0 14.73165389 0 0 19.20966592 12.08321039 0 0 0 0 0 0 16.84880598 0 0 0 0 0 0 0 12.78333333 5.706666667 0 0 0 0

582 0 4.884456598 0 21.94438888 0 0 19.66873706 15.30144494 0 0 5.112548984 22.88000363 13.13435331 0 0 40.6486243 57.13228996 31.63880734 27.63836253 0 0 18.26459875 9.776063866 0 0 0 0 19.30738078 0 12.99339319 13.05057702 17.46825397 0 0 0 0 15.46796348 13.04656229 0 0 0 0 0 35.74975299 0 0 0 0 32.8703523 0 33.33333333 12.8500761 14.36747582 33.33333333 0 33.33333333 14.73853517 8.884958733 41.75615192 0 20.37 0 18.30333333 0 16.1694291 8.220967911 33.33333333

583 23.48512675 37.68868979 8.051307763 31.56007291 77.60267092 0 51.38971081 0 54.89589775 32.82252971 12.67050621 0 11.41449394 60.97279173 82.09386892 33.33333333 0 12.05651815 57.22049367 63.95749251 53.21565618 18.13320627 0 74.68762091 35.84306645 54.78152828 56.77932971 17.90769713 33.76940192 51.78543583 23.31284753 0 51.43061516 44.76003277 66.92135984 56.12769956 24.18839611 33.33333333 75.71439012 74.53363914 20.24884387 16.43784787 40.11048028 10.77960294 44.73488399 0 50.77807634 57.93898098 15.88829715 79.12970833 61.32954171 0 43.18146674 22.16183575 78.83192462 52.08348964 36.74201618 19.73814417 0 58.05723786 10.37333333 5.52 6.59 18.76408579 66.66666667 53.43182165 58.57308109

584 0 0 0 0 0 24.31430131 0 0 25.99707663 0 20.9520277 0 0 0 0 0 0 0 0 0 14.274493 0 33.33333333 0 0 0 0 17.90141823 0 0 17.05689053 35.72653384 18.03519062 14.82028557 0 0 0 0 0 0 0 0 0 0 0 0 0 0 0 0 0 37.61285657 0 0 0 0 0 0 0 14.30604982 0 0 0 33.33333333 0 0 0

585 26.20089885 6.538523404 33.33333333 11.361805 0 19.47826087 0 16.23529412 0 56.27155564 9.857529087 38.83322325 35.73273737 16.40988893 0 9.520659335 10.1521715 30.15644019 0 0 0 25.74379057 9.891076382 0 0 17.3699373 19.14925152 0 17.09401709 10.16432323 10.03376749 15.86507937 0 0 0 23.78160558 22.67850698 0 10.07819923 0 0 24.81183007 0 0 16.21950161 0 21.25012295 0 23.28697422 0 0 0 0 16.48452736 0 0 18.59479816 57.78170793 10.69902189 0 0 18.01333333 4.4 19.28830462 0 25.11236542 0

586 0 14.30479707 0 11.38894446 0 0 0 0 0 0 0 0 20.19898002 0 11.20103085 5.706016126 0 13.59666667 9.446172993 15.5304888 0 0 0 0 8.554507567 14.82198856 0 0 0 0 0 0 0 0 4.534403112 0 0 20.28677104 0 0 22.25861576 0 25.0923064 30.91691368 20.6824147 15.2699784 0 8.045263231 0 0 0 5.707762557 0 0 9.093603652 7.569270141 20.3784219 0 0 8.609428809 0 3.023333333 0 0 0 0 0

587 19.37280702 0 16.39739229 0 0 19.00809297 13.66459627 18.03188839 0 0 0 0 19.51943536 4.508881129 0 0 0 0 0 5.913891145 0 0 6.244503078 0 0 13.02654586 14.18408181 30.8575513 0 10.17561692 10.02048581 0 0 14.93495634 0 0 0 0 0 0 0 16.89548547 0 0 0 7.731271661 15.88859032 11.14851326 0 0 5.337124959 0 0 0 0 0 0 0 3.632942539 0 6.646666667 0 8.626666667 8.97128317 10.49723757 0 0

588 14.3832943 21.91035333 0 10.65319596 12.32658418 10.05150287 3.966306837 17.09803922 0 0 5.419362678 0 0 18.10843821 6.705100239 0 9.534376703 0 0 0 0 15.20012706 0 25.31237909 0 0 9.887336958 0 0 8.394407194 0 9.10896004 15.2360515 18.51304776 0 0 9.144937226 0 0 0 0 0 0 15.30152345 0 0 0 0 0 11.40237305 0 17.72613352 0 0 0 0 0 0 0 0 0 5.12 9.396666667 14.04502871 0 0 8.093585582

589 0 10.74203303 35.45770924 0 10.0707449 13.85507246 11.31064902 0 19.10702561 10.90591465 33.04413276 17.95560812 0 0 0 10.79136691 23.18116183 0 5.694970808 14.59812754 19.74564059 12.08275568 13.85612228 0 35.23433501 0 0 14.02595256 34.1631866 0 18.12534948 15.17939815 0 0 7.26544083 20.09069486 9.418991554 8.411640754 0 19.30045872 21.13285589 0 20.06555943 0 18.3631997 39.72572909 0 13.68349865 10.50934014 9.467918623 0 19.71788987 39.32738373 0 12.07447173 0 9.546228589 0 43.91188366 7.682087782 3.946666667 5.883333333 7.966666667 5.597964377 6.666666667 13.23484501 0

590 0 0 17.38949911 11.20953876 0 0 0 0 0 0 0 0 12.31658515 20.13056019 14.75607654 0 8.682873578 7.017779569 0 10.34435262 0 0 17.11622536 0 0 14.2293799 6.72089535 0 13.80386508 6.483388556 0 8.136682737 17.92730941 11.82864179 0 0 0 0 0 11.45123658 21.05761492 29.47634176 0 13.07084744 0 7.96604636 0 9.683804761 11.33403731 0 40.24252554 0 15.98413019 18.92809657 0 9.491733007 13.4680917 7.277784697 0 7.836368686 3.32 0 0 0 0 19.51697425 0

591 22.84525155 7.112545574 0 9.564010206 4.11332992 6.935379213 0 0 17.44159149 0 0 0 0 10.9765387 0 12.29773463 2.855723138 25.6952189 37.99793052 0 0 0 13.6076437 27.45454185 0 0 22.54430554 17.40496387 0 11.32969449 0 0 0 0 0 0 0 7.78417573 43.88045902 9.777538572 0 0 0 0 9.217027746 0 16.16602544 8.065807673 33.33333333 0 0 8.313028522 0 0 14.50479 0 0 0 12.45736852 4.179775281 0 14.7 0 24.4519539 21.68554651 0 33.33333333

592 0 0 0 21.02796674 25.89895505 41.28889143 16.17038875 0 4.996022275 9.472979195 40.07585514 33.44592583 0 16.90301803 0 19.34105534 0 0 19.30553694 0 0 25.8989899 0 0 0 0 8.590051708 14.99676794 0 0 0 0 0 24.98385735 21.22222222 17.74027578 14.44892473 21.0203769 0 0 0 21.24900239 6.906560358 17.52972766 0 0 7.180924695 0 0 19.44501683 0 11.79430872 33.33333333 6.657890538 0 18.84492406 0 0 0 27.02233789 0 0 16.09333333 11.82873535 0 17.70575437 29.96450144

593 12.10671645 11.64185545 21.49959481 12.00660363 6.613756614 0 0 15.68113017 10.09751977 0 0 0 2.90993528 0 0 0 16.55120292 15.00291101 0 28.51147022 41.20906801 0 0 10.85785106 22.72186791 33.5488958 10.92073382 0 10.3767968 12.33448144 38.37690204 0 5.932119041 10.97215274 39.92832914 15.22424802 33.33333333 4.994668373 0 0 0 0 14.56628478 28.11038193 26.14666818 28.16556379 0 18.40913209 16.40267511 26.09361625 6.074104769 21.79588608 0 10.6109463 0 0 0 24.19613158 12.68774704 24.90966877 4.343333333 29.25333333 15.44 0 33.33333333 0 0

594 22.66709674 0 0 17.95993123 0 0 0 0 0 0 0 33.33333333 16.53834765 5.781535649 18.27711942 28.34142001 27.82503676 14.02944156 0 22.66791045 25.72516718 19.09993254 19.72568963 0 0 26.43081251 0 49.2617028 24.62363348 0 0 23.80839566 26.30033187 15.96824184 0 21.14828583 0 0 15.51462622 10.28464769 0 18.14362108 0 0 0 0 13.17648886 23.64952857 0 0 18.26097509 19.16774163 14.01269717 18.84768399 23.23262551 33.37397727 40.44738226 16.02524183 33.33333333 0 6.943333333 0 0 15.78440808 44.98112016 0 0

595 0 0 0 0 19.94714641 12.89743883 33.33333333 37.98183653 36.28275071 22.94654498 0 0 33.33333333 14.26679646 0 0 13.49234187 0 8.147622907 17.03397612 10.34424853 27.68656716 16.21710798 0 0 0 9.096205666 0 0 29.67513548 0 33.33333333 0 0 0 0 49.49596774 18.1594715 0 21.9998066 43.5689601 0 44.57167721 22.72432942 0 33.33333333 11.83900886 11.67649414 20.26482441 15.4069449 0 0 17.34920315 0 33.33333333 0 33.33333333 33.33333333 20.87596482 0 0 13.48333333 0 18.45268161 0 26.98591381 5.845719791

596 14.55194733 0 0 4.541194255 28.06481991 0 17.16294458 0 15.89174185 57.19368747 0 33.22074083 5.42463317 0 0 0 0 0 0 0 0 0 0 33.33333333 0 0 0 0 0 9.242250287 28.48149648 0 0 22.36118059 4.017094017 0 0 12.63382384 12.42955221 9.099390025 0 0 0 10.48755864 24.11630559 0 0 0 0 0 13.95045459 21.53902461 0 16.32486349 0 8.996136424 0 0 0 16.15283895 2.35 0 0 0 0 15.62757896 0

597 0 13.44211344 50.32510511 0 0 0 18.86784867 33.33333333 0 10.38678835 59.92414486 0 16.79498569 24.52289807 33.33333333 11.10763128 0 29.35066183 0 0 0 0 33.33333333 28.35427375 0 14.316327 8.988115842 18.3365654 0 0 0 10.79675209 0 8.349475985 19.68705548 27.13841204 0 0 8.397299218 0 0 19.04670382 18.91491254 0 40.51999848 11.84006211 25.01729625 14.92420124 0 12.58005639 0 17.39001044 19.32063617 20.891176 0 11.83933157 0 6.040602815 0 0 15.71 0 16.55333333 4.804299005 0 0 0

598 16.81510391 21.69147789 0 11.7627195 0 13.50051529 0 13.00369997 0 0 0 0 0 7.418652902 33.63347071 0 30.59282174 8.903987127 0 10.77686771 0 21.66774422 0 0 77.27813209 0 10.7890278 0 41.78807049 11.75660161 11.81838415 0 20.65314911 0 8.094017094 0 2.721774194 0 0 11.33352674 23.09770657 12.08433094 0 0 0 0 12.30685612 13.59103152 5.596620908 0 11.55415839 0 0 7.739343117 20.88277171 0 0 0 0 0 0 0 0 0 0 20.1637786 0

599 11.01388403 46.11200765 10.78580097 11.92803568 15.36199209 25.37777523 14.46548466 0 15.29037391 0 0 0 12.68217973 0 0 28.91215875 0 0 34.54890964 10.66542289 22.72151628 5.646766169 0 0 0 11.4745848 22.35066428 0 9.407634138 19.17844814 21.32321733 23.92483618 29.18709057 5.536449709 7.051282051 18.74877833 0 35.40748366 19.77806334 26.0538538 12.27571841 0 15.04056511 8.077154913 0 18.6949944 14.31339978 0 13.06850892 26.47436563 9.917781621 0 0 0 8.046479438 17.45389767 12.75119271 13.12690575 20.6455863 19.89901043 7.336666667 0 12.41666667 24.67792204 0 0 30.85644543

600 0 0 0 0 5.832225014 0 0 6.774069812 14.87300627 14.01199977 16.51452123 15.48436044 0 0 0 0 0 0 0 0 12.96431173 0 7.407834101 7.038512616 0 0 0 0 0 0 0 27.96413552 0 0 0 0 0 0 0 0 17.90004976 18.70412115 0 21.26116967 0 0 0 0 16.49141121 0 0 12.22327855 0 0 0 0 12.89039605 0 9.011135318 0 0 0 14.98333333 0 6.709390531 13.07133704 14.12042075

601 0 0 0 0 0 0 0 2.048789785 0 19.852357 0 0 12.72530819 0 0 6.493262771 0 0 0 7.593393194 8.094015098 0 0 6.35978195 0 3.256883585 0 14.55205901 0 0 0 0 0 0 22.30895548 7.778963595 19.705437 0 12.82051282 21.06303419 0 14.48907824 22.66609977 0 0 0 0 19.80756142 0 0 0 18.18753142 0 46.07170099 0 0 0 18.87761823 0 17.00713758 0 12.34 0 0 20.47909817 11.33614858 0

602 5.744893755 0 0 0 0 20.95321335 0 0 0 0 0 0 0 13.86513451 21.57749066 9.641796585 28.02193498 32.81556771 0 0 33.33333333 0 28.7211778 0 0 0 5.399093282 0 0 0 0 0 0 0 6.927414765 20.20750988 33.33333333 22.68829208 0 0 10.67211626 0 0 7.22334004 0 20.37506336 18.97171816 0 0 23.75539417 18.56693295 0 22.8966823 0 8.633861364 15.26345135 0 40.74763678 9.621353197 0 0 13.50666667 0 13.20628552 0 0 0

603 42.66608729 23.52559301 47.98468963 13.99101307 38.48332132 0 22.73216657 16.58227848 22.69919462 0 9.331671011 0 0 0 0 5.55493 13.99682219 18.24111878 43.60918412 0 0 54.01875902 0 0 0 10.99463061 28.69106971 0 39.76215513 0 32.38328798 0 9.650664864 0 0 16.00211448 0 17.11611183 14.54227686 19.29882586 21.19854722 0 0 9.341355288 10.74013887 0 0 14.65698365 31.48818717 44.34116757 33.33333333 0 0 17.0867448 0 24.02425161 37.99982916 0 0 0 0 12.41666667 0 0 18.48991849 8.483732351 20.25170922

604 0 61.26152495 0 0 0 12.92939326 0 49.18536131 43.42085282 9.340316748 21.65843582 9.028319044 12.47192109 33.33333333 22.98738178 0 0 0 0 42.98528701 9.423808368 0 11.51305684 33.33333333 0 33.33333333 0 0 0 19.35810322 0 0 14.42873969 0 5.229910386 0 0 0 18.6259542 19.24460432 0 20.28923661 0 4.408511323 20.61746669 0 0 9.428325485 11.19976359 0 25.78767123 18.9112426 0 16.24713959 0 0 0 0 0 26.14461195 0 6.946666667 10 0 14.37352864 6.198680526 0

605 0 0 33.33333333 66.66666667 19.85616472 14.46665173 0 0 0 8.555498953 29.65333333 45.95520468 8.098816365 19.46819882 7.713402182 10.31922791 23.907243 6.124218218 0 20.44268593 15.63545801 0 0 26.29482072 66.66666667 0 11.58126116 48.73420067 16.70393375 0 0 8.144361428 0 70.97039295 16.3424328 0 14.32175432 0 20.51282051 0 0 32.0286915 48.0272574 26.10999329 0 66.91128326 0 0 0 0 0 0 5.902192243 0 67.21096805 33.33333333 0 25.91902989 20.73859414 9.349593496 31.82666667 0 7.36 42.54429957 0 0 33.33333333

606 36.19670628 0 0 0 23.51190476 20.40394007 50.69070685 0 0 0 9.766141524 0 0 33.33333333 0 28.53524894 19.33651115 7.001736356 11.6096711 0 0 0 4.736501778 26.97355138 33.33333333 8.354563468 0 0 14.85548583 55.04517553 67.61671202 40.69403133 41.01419878 0 0 13.12582345 15.97758177 0 0 0 9.778988798 0 0 13.25733587 0 0 33.33333333 0 0 0 14.76640039 15.14580191 24.04604458 0 0 18.06988198 33.33333333 14.4557151 24.32219802 0 14.26333333 0 0 0 0 21.1521016 25.37034374

607 0 0 8.457314029 19.34232026 0 0 26.57712658 0 8.372807579 10.73107663 0 14.93244999 0 0 12.04462915 0 0 27.72688073 0 16.08798646 10.94521324 32.7861565 14.4124424 0 0 0 37.97559696 33.33333333 0 7.599633753 0 14.35749552 18.90459364 0 0 7.518083719 0 0 0 26.35902816 0 0 0 0 33.33333333 0 34.53520185 37.43077976 10.40927896 24.65353952 0 35.53214551 0 0 0 0 15.77644146 0 23.71198014 23.98373984 0 20.96666667 12.73333333 33.33333333 14.84341484 14.06331577 6.924192963

608 15.39231267 9.807740325 0 0 12.31638418 0 0 15.43251557 10.63413871 0 0 0 12.50920878 0 20.05951339 13.37230883 0 0 11.44781145 0 0 0 0 0 0 23.21461868 16.35297889 0 6.799171843 0 0 0 0 0 26.40591857 0 0 22.45234604 14.70737913 0 12.13478612 0 10.77252977 0 35.3090611 0 0 0 16.63678024 7.249898744 7.5456621 0 0 20.59441462 9.797233276 9.309081723 0 0 0 23.51491713 0 0 6.76 10.91608158 0 0 0

609 0 5.405141714 10.22466301 0 0 31.24680159 0 9.97698504 0 37.50875089 13.07589709 14.59966585 54.19474558 0 15.61758283 26.08322495 14.73748869 8.090478202 33.33333333 12.8906474 9.603860223 13.19508449 33.20898709 0 0 20.84597032 0 3.380406983 21.87925345 17.99708749 0 8.839976205 16.00180302 29.02960705 22.785368 35.36750487 16.66189358 37.74325005 18.79105647 14.03450747 28.31551185 14.4888725 18.53411306 18.39829452 0 12.71365339 13.15974666 18.67634968 13.77457883 0 0 0 47.15508088 0 14.35793731 0 0 0 12.59473919 0 7.476666667 13.05 14.82666667 0 25.10464933 25.69468414 0

610 0 6.266622946 0 0 0 0 4.899252219 0 10.17789942 0 0 0 0 38.37248429 0 0 0 21.80982099 11.69915382 0 0 0 0 1.818049331 0 0 0 0 0 0 0 0 0 0 19.03796152 0 29.75131189 0 0 10.60848679 0 21.07166338 0 16.06393783 0 0 0 4.01693366 13.1301056 5.047015928 0 0 0 0 0 0 4.14981911 24.8782848 0 0 0 0 4.973333333 20.89562715 17.89627465 0 10.6925769

611 0 0 0 9.21535077 4.98953394 0 0 12.25107017 25.92762604 0 0 10.37467259 9.070351759 0 0 0 0 8.676019997 0 0 17.67897023 40.13520765 10.49857749 4.030277409 0 14.55677868 9.68004602 19.71050269 0 4.586117501 0 0 12.69100744 0 0 17.98367057 0 0 0 7.575493105 10.13721073 7.108403148 10.02985679 16.29103749 15.60696189 20.36296607 11.06414385 0 0 11.91773652 17.49025258 12.38489257 0 8.064862543 0 10.59190031 0 22.86042714 27.16403635 31.3326065 11.21 7.09 0 14.32200835 15.05082689 11.00561328 3.44142506

612 0 0 9.35828877 13.19563215 0 12.82843895 0 16.03363568 11.15065243 8.715184187 11.77230229 0 0 0 0 9.116339477 0 0 12.82779938 21.78627092 0 0 0 0 0 0 0 4.553707248 20.24516479 0 0 0 0 0 0 0 5.380683347 20.57172027 15.61349607 0 33.33333333 0 0 0 0 0 0 0 0 0 0 0 0 6.890881913 0 0 16.79383372 0 12.62564354 0 0 0 0 14.35252672 0 0 0

613 0 0 5.132521799 0 0 0 3.401694707 25.31732084 8.512943323 6.288976724 8.034307719 45.9232459 0 0 0 0 5.977796755 0 0 38.78691864 0 11.41232612 7.573492775 0 0 15.60313759 0 0 14.57551758 12.71950393 21.53332159 9.507870094 9.397237338 13.83422376 6.948356808 0 9.62818287 0 10.41019514 40.3189218 0 0 0 0 7.049476345 0 9.998982809 16.59071519 0 7.154097102 0 0 4.493545184 0 0 0 0 0 0 0 3.446666667 19.85 3.13 0 4.317144043 0 0

614 0 0 0 0 0 0 0 0 0 0 0 0 0 0 0 0 12.13640302 0 0 0 0 0 0 0 0 0 0 0 0 0 0 0 0 0 33.33333333 0 0 0 0 0 5.925319134 0 0 0 0 0 0 0 0 0 0 0 0 0 0 0 0 0 0 0 0 0 0 0 8.741173606 0 0

615 33.33333333 0 33.33333333 0 0 69.13954403 0 29.09827567 36.82517149 33.33333333 21.56103104 0 0 0 33.33333333 0 0 56.39862871 35.38540702 0 21.39076577 38.51747916 33.33333333 0 0 37.93316874 33.33333333 21.43125405 0 0 0 0 20.6423259 0 14.29537181 73.54284948 0 12.28417029 21.20573779 25.75784023 33.33333333 33.33333333 33.33333333 33.33333333 54.86621781 0 19.7469194 0 53.53656107 33.33333333 0 0 0 0 0 33.33333333 14.93225509 18.77538962 33.33333333 0 0 0 6.69 8.331578578 0 33.33333333 0

616 51.99861368 93.73337705 46.44331703 69.79548656 95.01046606 0 73.10949042 17.29969766 0 45.34717857 25.29902561 14.01890445 23.61788618 61.62751571 66.66666667 48.0309328 81.88580022 0 0 19.01257796 33.33333333 9.934987061 48.5945964 94.15167326 100 0 56.98662065 54.30453601 65.17931764 62.08054917 78.46667841 90.49212991 51.36737826 61.40063071 10.15567087 0 51.65780045 12.76161307 52.770571 15.73925807 0 26.22493019 33.33333333 34.31169135 17.72637145 39.18686795 0 57.79231266 28.69853093 42.54781711 82.50974742 87.61510743 95.50645482 85.04425554 100 56.07476636 38.97443967 33.48589845 0 50.35748065 24.18 6.31 0 19.01132498 35.63885656 42.44297146 79.97479647

617 0 0 0 0 0 0 0 0 7.405707291 0 33.33333333 0 0 0 0 24.21699386 0 0 23.80610126 7.976980965 0 0 0 0 0 0 0 0 0 0 0 0 0 0 0 0 3.582021446 0 0 0 0 0 23.30347654 0 0 0 33.33333333 21.60003849 0 0 0 0 0 0 0 0 0 0 26.87698678 13.35957144 0 0 35.1 0 6.695885074 0 0

618 0 0 0 0 0 0 0 0 0 0 0 0 0 0 0 12.64495045 0 0 6.754306437 0 0 0 0 0 0 0 0 0 0 20.6138294 0 0 0 0 0 0 0 21.04916304 0 0 17.27080347 12.26166995 0 0 0 33.33333333 17.65297907 0 0 0 0 0 0 0 0 0 15.66536497 0 0 0 0 15.67 0 23.08693421 11.65983917 0 0

619 14.66805299 0 5.732539066 7.793530514 0 18.03201702 18.58956265 0 0 6.315327185 0 29.68317706 67.31176206 0 0 5.99078341 0 13.1155303 9.527232072 12.43725151 27.59693067 0 0 0 0 31.906915 0 0 0 0 0 0 5.902051067 24.76514553 16.22930566 8.473479948 0 33.33333333 0 0 0 0 0 0 4.750972515 7.116832645 8.203641542 0 4.6348024 0 0 0 0 0 0 0 9.484287437 0 0 4.950341403 9.283333333 0 14.38 0 0 13.21808193 5.891201572

620 14.19853339 19.79966761 0 0 0 0 10.65162907 0 21.66956204 9.25081103 0 0 0 15.95532184 30.07133635 0 39.04145302 16.65549847 0 6.460578944 7.081641858 0 0 0 11.04525615 0 23.8796199 9.633467665 25.64721473 12.92190195 12.30121116 11.11637829 14.6331324 0 0 9.299963729 0 3.038149491 9.857875857 19.0186443 9.512267721 18.43862352 0 0 16.54161782 0 0 10.45715343 0 10.33450475 28.79436665 24.81941552 33.95179265 14.63745436 26.36975113 22.37962285 0 23.90464258 13.79489849 0 0 3.706666667 12.1 0 0 0 17.93277564

621 0 11.81022195 0 0 11.11464968 0 0 20.37547937 18.51133496 14.41273228 12.45517746 15.20075036 0 23.6337088 24.81844227 0 14.56456456 24.38856146 7.712854758 0 0 0 0 5.059652149 12.19784306 16.3883618 23.19089718 12.03961652 0 27.73482727 46.06346601 20.45137273 15.11078216 10.22475795 25.77286576 0 13.89786318 0 27.53842278 0 28.91558544 0 0 37.7084006 0 16.84106335 0 41.04318328 0 0 14.33669618 0 0 8.193734902 0 0 0 17.95646699 24.61969163 10.74103168 9.236666667 0 0 29.94305221 65.32443525 18.15030856 0

622 0 16.67732719 33.66969602 56.89661319 47.3727422 8.053202729 0 19.82460375 0 15.51286298 0 0 12.67548044 0 0 0 0 0 20.38849217 0 6.871147196 34.28775977 0 23.00819062 0 0 0 0 11.96174696 0 0 0 0 0 0 12.33248005 18.22481914 19.46700271 20.57691589 21.51611261 0 21.42241041 28.69628814 0 0 0 0 0 9.532033049 0 0 18.01389639 0 16.95762997 8.892401358 19.7286507 49.72367161 0 0 14.96776442 8.016666667 0 0 0 0 24.26258251 3.57371515

623 11.21526898 0 0 0 0 16.8080294 0 21.8255525 24.64168119 24.39849599 33.33333333 0 0 16.08280996 11.35650988 56.05175292 0 8.915940734 31.22742852 32.49084088 35.44779597 16.78851522 0 0 0 38.97676496 0 0 0 0 0 33.33333333 14.37387503 11.40733057 0 24.87810436 9.693409981 21.00248012 12.17817736 11.76365688 10.57107624 3.942978465 8.112638284 19.25672465 9.431721798 33.33333333 18.20854027 5.856005923 30.33504377 5.507043893 7.715023994 0 8.369307354 0 0 15.29178124 0 10.38735058 15.5416739 0 5.52 15.98 28.84333333 0 0 0 0

624 3.651754515 0 33.33333333 0 0 0 42.32433018 7.49953934 0 0 0 33.33333333 33.33333333 0 10.17121233 17.64141898 41.40447537 0 0 0 0 0 0 0 17.00436481 0 35.83892415 22.94769982 0 22.15422012 21.03212217 0 19.61219956 0 0 0 13.17236298 0 0 0 4.91645081 0 0 4.37851464 10.79379425 22.25946617 33.33333333 0 0 36.2602288 15.66238246 0 33.33333333 18.69587898 23.46512587 0 5.496327884 11.11613876 21.3062343 33.33333333 0 0 0 61.86467489 0 0 0

625 6.042527153 16.65600614 17.92645917 0 34.34700302 13.54335991 0 0 10.73213122 17.82047035 33.33333333 0 30.63062613 0 8.514891065 0 0 7.761894126 0 29.40820159 8.95604671 16.82397548 70.42887362 33.33333333 0 0 0 0 33.33333333 20.41143139 12.26363136 0 0 9.027156073 12.21311475 17.83861671 0 0 0 0 0 14.89470981 15.50458251 7.529026648 31.62728563 0 21.7317206 17.02017398 14.39221056 0 9.676755199 6.251989812 6.008633765 25.11427884 0 5.352404084 14.96474098 6.245727649 0 18.36556891 10.21333333 37.42 3.016666667 0 10.06733187 28.60072755 40.71363166

626 22.11806435 13.53366572 15.07051147 21.65329692 0 46.85742514 33.33333333 0 0 0 0 0 0 0 0 0 0 42.27810521 0 0 31.2188707 32.09974954 0 28.27368118 54.4688236 0 0 0 21.37158638 16.77761927 0 0 32.21695759 0 0 21.00085329 6.956037841 9.347488348 11.29728012 11.01151349 0 14.3767061 28.94137988 14.07660869 0 0 0 17.84740316 2.998289566 0 14.49489356 16.90850464 0 0 0 0 0 0 0 0 12.36 0 0 0 7.296511628 0 0

627 15.48304543 21.52311138 0 21.45008989 0 0 0 0 11.6637713 0 20.87815587 18.13258298 0 35.05417954 10.35580784 15.69191435 4.989507045 0 12.94484116 0 0 0 29.57112638 10.32514272 5.283712382 33.6223398 17.09055877 26.99045548 7.686118605 0 0 0 0 0 26.55775754 0 29.90303053 38.89354151 18.551328 11.81722072 13.24998937 4.645965655 0 6.519294163 9.066041422 16.49226998 0 0 23.80130028 27.82628944 0 34.00619364 11.46492263 0 0 23.64285849 0 14.4802542 20.74074074 0 7.063333333 9.903333333 10.61333333 0 0 0 0

628 27.29080618 0 0 0 7.165605096 0 0 16.96609545 12.7815193 9.171334578 0 33.33333333 23.36056009 0 0 10.61491375 0 0 27.72638339 27.71524684 0 0 0 0 0 0 0 28.38876051 0 0 0 35.09891565 0 69.34075541 21.12021858 0 0 0 0 17.67393562 32.83463042 0 0 10.53143062 0 11.07386716 17.40626536 0 18.94112277 0 9.319881957 0 6.872010267 8.825085365 41.27272164 13.60468264 21.2378253 9.131138694 3.996760946 0 0 0 0 0 17.31172125 28.98638138 37.77987755

629 0 0 0 0 0 14.73798282 13.69070741 13.50872958 0 9.433292782 0 0 0 9.273979862 4.711800268 0 0 0 0 3.925131746 10.42449757 0 0 0 0 11.01253345 0 0 0 0 8.339569302 0 4.053053255 0 14.33604336 14.64998186 8.152476349 8.251337822 0 7.198916378 0 22.27860604 18.74511119 0 22.53953909 0 9.32014044 7.776080229 0 20.07193312 0 0 0 7.575937587 0 0 8.577434228 6.778280543 0 22.59230165 0 0 20.08333333 8.192272903 0 0 0

630 0 0 14.68312584 26.70553936 19.41490211 0 0 0 0 0 0 0 18.15803244 0 33.9418404 0 22.32816516 0 0 0 0 0 15.51271421 0 0 0 10.03301025 0 0 0 9.513770371 12.42717116 18.89944819 0 0 17.04550549 0 0 12.951585 0 0 0 0 12.98944127 22.63362198 0 0 0 16.85446009 18.54051565 10.45751634 0 12.56356384 0 18.04024785 0 0 0 0 0 0 24.08333333 0 0 0 18.41476416 0

631 29.73928777 62.18302555 50.21552423 31.17169027 40.36362965 0 72.86373074 14.81152665 19.21454628 28.46821402 60.40879956 11.16044642 25.1366765 6.456200228 0 32.78757204 10.50336901 0 17.13752619 0 36.38803959 36.78728718 35.46845347 73.19288938 66.01038059 18.8755796 33.38946432 30.97298262 12.59029928 25.16223194 11.25875567 14.92075059 13.64675622 12.05932738 6.440339491 17.21111653 20.30720132 0 26.49241691 36.88835608 6.983421321 15.64119901 42.58286747 16.69005997 23.40794451 19.25947899 19.18565684 34.96834361 14.68045431 17.97101449 30.08198841 8.114965477 32.76411513 31.78063352 31.08491114 0 15.98054275 8.147413651 15.15166076 33.07489106 23.85333333 2.503333333 6.293333333 6.495071971 0 8.986124768 29.30233072

632 11.17886179 0 0 0 0 47.26898616 0 14.01944895 5.805937438 0 0 0 13.83559507 21.16578783 0 0 11.29534588 12.48371123 0 3.351538067 0 0 0 0 0 6.067757499 0 16.71066415 22.16773276 20.53420489 11.4090375 16.92614143 0 0 0 0 0 12.92378628 0 33.33333333 0 21.76783409 0 8.568170712 0 0 0 0 12.59010897 9.710013135 0 37.35762746 0 0 0 23.58198834 18.78719184 0 0 0 7.306666667 0 0 7.017870439 0 23.16266913 0

633 0 0 6.601252022 0 0 0 0 0 0 11.96154782 0 18.17121844 0 0 13.87055463 0 7.739703816 0 15.16080837 6.935340293 0 9.853229582 10.12898846 0 0 0 0 0 13.27949753 0 0 0 8.46432314 29.70412427 38.11272101 0 3.006779486 10.65604854 10.75359865 0 0 0 0 0 0 11.00716101 0 0 0 0 0 0 6.086903566 0 0 19.53274467 0 7.020980239 0 0 0 5.62 9.96 17.28407908 0 0 11.4655486

634 30.4099954 0 0 0 14.38555743 0 0 0 0 17.65471542 7.768559088 22.17288692 0 0 4.952027236 0 0 0 0 0 0 10.35982602 0 0 0 0 0 0 0 0 0 0 14.43388514 0 0 28.04180864 0 12.15988265 0 19.24657534 59.68324535 8.939974457 0 0 0 13.48171562 66.66666667 6.770436486 0 0 6.79157589 0 0 0 0 0 0 1.669145596 17.00531898 0 3.87 0 0 0 11.41960784 0 14.71503831

635 0 0 10.29978934 21.43011969 0 0 0 9.536492435 0 0 0 0 0 0 0 39.40908423 11.00516817 0 0 0 41.07602605 16.01208459 8.395522388 0 0 0 23.30032308 24.12422528 0 0 0 0 26.7645951 0 0 0 19.18120504 8.516409005 0 0 0 40.14892253 33.33333333 0 27.90956925 0 0 15.25929007 7.923839332 34.56254672 0 5.592182778 0 4.924242424 0 0 31.89893207 0 0 0 0 7.163333333 14.10666667 13.71928518 0 24.34720857 0

636 0 20.61797779 0 0 0 33.33333333 0 33.33333333 0 19.5175821 16.43157209 28.00291608 19.49773826 27.79190891 0 15.66164154 0 33.33333333 18.17252497 0 0 0 15.03733876 0 16.70818801 17.96589236 33.27720235 16.62266918 0 0 45.7438588 30.01036189 0 0 0 16.1222168 0 0 0 0 0 0 6.048738033 46.76972955 0 0 14.1476765 16.83921702 18.65287902 15.36231884 0 10.13159362 0 22.07559716 32.53908167 0 33.33333333 19.16638389 0 52.99020629 0 15.60666667 14.65 26.83826136 88.58039216 14.91856917 0

637 16.97760417 2.892976589 4.058521488 6.627793975 11.91747958 19.39768051 27.13626926 19.31388439 60.86072923 0 15.39106926 0 23.37195772 35.30383237 12.25970632 0 37.12824797 54.18295543 49.52914047 89.71312164 22.53593436 0 9.425096739 26.80711062 6.711758585 41.72332957 0 0 40.79686986 54.30356317 22.07457767 16.40719191 0 58.23654835 55.4469395 0 39.32958256 43.85073547 49.80239944 0 33.33333333 8.752159868 18.03506117 0 3.70259779 56.25164439 0 0 0 0 28.97387525 10.96233954 48.58541746 22.90553692 18.33575934 56.88526699 0 49.82912718 67.84302026 8.495592367 0 21.06 18.33333333 16.04925425 0 10.17066421 44.51708237

638 0 0 0 0 13.91843122 0 0 0 14.11878705 13.09726616 0 15.16211489 0 0 0 0 0 0 0 0 0 0 6.031885975 0 0 0 0 0 0 0 0 9.308383028 11.22225398 0 0 0 0 11.89313805 0 10.53173524 0 0 0 0 22.34626647 0 0 14.99219912 29.29825827 3.85359116 16.0842411 0 0 10.59059988 0 0 0 0 0 0 0 0 0 0 0 0 0

639 11.69425087 14.30602007 14.14178709 14.06485671 0 0 0 8.985314246 0 9.300674476 0 5.330417256 0 9.282270659 34.97587142 12.14170219 0 0 0 0 0 26.98757263 0 0 10.56967281 15.36744098 0 11.56945877 11.16560057 0 0 0 6.56873823 0 0 21.57935254 18.17523159 0 0 0 0 4.74991004 0 14.9825985 0 0 0 11.17051369 0 0 7.610803012 27.84129112 0 7.723390108 0 0 0 14.16694944 0 5.439310278 12.54 0 0 12.59617771 0 0 0

640 0 12.70980392 17.30927188 0 0 20.0731661 0 9.504050583 0 16.26406259 0 12.98179404 27.28283349 25.81196581 0 0 16.07529878 0 0 0 17.37640177 11.8059433 38.28340402 11.56826282 0 0 0 0 0 8.924911496 0 26.30433256 21.86544343 0 0 17.22552731 0 0 0 0 31.24038746 11.65113741 20.8045977 16.33543761 24.35633325 6.692939245 0 0 0 5.541305321 13.04224714 0 0 0 4.692748469 24.95211472 13.02040913 0 8.9120556 0 0 0 8.403333333 13.83370125 26.79346618 0 4.023217688

641 0 0 0 0 24.67267583 26.65035115 29.28329254 16.9702832 0 0 0 30.03004434 0 9.262789959 8.224988474 10.05975805 9.802743567 0 23.72270545 39.60667398 4.21341609 0 33.33333333 15.62761436 0 12.04574802 5.124727736 25.0939956 0 11.99491741 4.662747833 17.97494626 29.28270585 0 12.62738854 0 0 7.111975789 14.60313592 25.33105264 0 14.71467329 13.23844506 0 0 2.041771094 30.20504389 15.89219331 5.364294128 23.20051372 0 0 14.34203849 3.829613586 15.65531341 6.029676645 6.593007196 3.924238673 10.96165522 7.874883937 18.24 0 10.06 14.25743799 0 17.9737287 0

642 15.12516801 24.75696446 0 13.39894408 0 0 21.07726569 21.24841227 16.83617256 6.893567926 16.18470304 0 0 0 0 8.313998453 0 23.40185067 0 0 18.71362633 19.43426943 0 0 24.37682817 0 13.0764982 0 8.078982597 33.33333333 34.07120272 0 0 14.37683074 9.835212489 0 0 0 0 0 0 0 12.63712154 8.092880314 0 0 17.16443529 0 14.32943795 0 13.37032633 0 12.35387782 18.06811837 5.227630464 0 12.06324917 0 8.61549628 0 0 3.586666667 11.93333333 0 0 21.45441004 10.70710552

643 24.67847397 0 18.038143 8.566054117 9.862096139 0 0 0 18.71650931 0 26.30753313 10.10588112 0 10.34318398 33.33333333 0 0 10.36576112 0 0 0 0 0 0 0 8.844470573 11.19141665 14.11102959 20.35575598 0 0 0 8.50764526 0 0 25.97112531 40.853422 10.44788975 9.331343055 0 17.83452098 0 0 0 13.85778905 0 0 0 0 10.01140048 15.63522874 17.23474682 11.63491288 0 0 0 0 66.09000059 10.64421278 0 23.75666667 6.926666667 0 0 7.891177157 15.72105506 0

644 0 0 0 0 10.5516783 15.09331891 0 0 0 9.239906345 3.1392261 0 5.172863666 0 9.714808601 16.11637477 7.133568642 22.78932997 0 15.81612469 32.70894917 46.41106768 10.63026626 25.63923486 0 0 28.2086056 0 22.61013076 32.91329265 8.256119026 0 0 0 0 16.10780602 0 16.76394293 33.33333333 13.47496409 0 0 0 0 8.478377893 0 17.54801651 8.178438662 7.77385159 15.36969697 15.29051988 0 0 31.20576683 0 5.921033822 0 0 13.89005651 0 0 0 11.06333333 19.49963208 0 15.35960463 23.62379948

645 18.20816532 33.33333333 17.34180567 33.33333333 0 14.85251892 0 0 35.76501412 13.45543698 14.78353042 22.24294122 0 33.33333333 0 8.406937764 5.49714629 0 26.9138469 0 0 0 0 0 54.35109496 0 10.98993458 5.091967162 11.9210174 0 15.37450723 0 0 6.501766784 6.815286624 0 33.33333333 0 0 23.1710228 12.97981907 38.57083736 26.88945738 0 0 0 0 19.68894988 15.59438708 0 0 24.69248016 0 0 0 33.33333333 23.96505376 0 0 19.38216841 23.80333333 16.68333333 7.396666667 14.49248469 0 0 22.62622781

646 9.682051282 0 15.29519034 44.70166847 0 13.26016724 12.87542088 23.82928275 0 0 0 0 9.475465313 0 9.017980636 0 33.33333333 9.931482659 23.22920243 0 0 13.8990639 0 26.50639641 0 9.811222157 0 3.733991668 0 0 0 0 19.21140431 0 33.33333333 0 0 31.31727887 31.4641508 18.16459123 0 0 0 0 12.59292079 14.00167084 9.005151768 19.22330097 0 12.01091161 10.31840402 33.33333333 6.772846157 0 43.81796711 0 9.03540016 8.511798533 0 22.65888047 0 0 0 0 34.02190264 7.612511969 29.31011565

647 0 0 0 0 16.1930874 0 0 16.36305014 8.990388705 41.16269773 7.025800207 0 23.23342134 7.521367521 0 17.21695856 0 10.54400336 0 20.20018984 26.98760663 0 17.75299638 0 0 21.28758532 0 0 10.72320257 12.83354511 37.63542319 55.72072118 2.960244648 6.454652532 23.49812084 23.90480521 25.81324467 9.457414613 0 0 7.264367816 35.06335194 12.52873563 33.33333333 11.94449429 14.03160517 16.16889804 9.262701363 19.00389538 8.634758251 5.000566316 16.09858651 21.69842045 0 0 8.381218616 17.6179546 0 0 0 7.52 0 0 0 0 0 9.709533849

648 22.11126961 20.62352941 32.01558913 0 25.88358289 0 28.24774704 0 14.06547999 12.98432843 30.19410723 24.63933928 17.16242661 13.72735939 39.70888895 0 10.12446591 0 26.13424522 0 0 8.449655686 0 7.694098475 0 0 31.40881724 36.62159017 0 0 0 0 0 29.8559581 13.89065817 16.79073615 0 0 0 0 15.18209232 0 13.90164269 42.23834874 28.77008472 26.64039409 9.908454496 13.64438345 37.93413387 0 0 0 12.21844869 27.03901307 17.67801992 0 0 0 33.19945865 0 0 20.79 21.06 33.33333333 31.29345402 0 0

649 10.19487179 8.576368876 0 0 12.83687943 10.07047768 8.51627385 12.08492107 5.626435315 0 2.365099871 0 17.67298958 0 0 39.8859724 18.03344348 22.96757222 0 24.37701149 0 0 0 12.96439307 21.27207687 48.01097394 0 15.34742581 26.31091069 0 0 0 18.17255651 42.81079184 0 0 0 24.90149805 11.2680369 19.85836924 15.49881235 0 0 0 0 36.59161956 0 14.11003236 0 25.23141364 27.34270758 8.640853178 20.97945551 19.85748815 12.92832062 21.38262287 17.70492598 21.4739622 13.77706496 50.08406719 6.423333333 9.333333333 16.70333333 4.583410651 0 21.87868959 0

650 0 0 0 8.989552472 0 0 0 0 0 31.0416939 0 0 0 9.896554909 11.75860325 0 16.06336769 16.33584303 0 16.39567402 6.743587236 8.711539867 0 0 0 0 11.96948021 0 0 10.54117885 0 5.61494439 16.51634858 0 16.34434304 11.27974205 10.79372999 5.96285435 0 27.67077446 26.51683022 25.48185109 0 0 0 0 8.307098182 11.9828686 7.782337741 19.19652321 0 16.08632016 0 6.665918108 0 38.8153899 13.89523955 29.55562358 0 0 21.28666667 0 0 0 46.11241455 17.66666667 5.070799847

651 6.544123904 0 2.493280723 0 0 9.884621589 0 11.82549845 2.310999642 6.723858103 2.098839536 8.876076589 0 32.24362474 5.63505269 0 6.000363108 19.32089909 0 23.1256873 4.99477534 6.373551466 10.59855128 2.665420496 0 0 0 0 0 0 15.64480031 2.620105298 0 0 11.76592547 7.741224351 14.11807847 0 25.42577482 0 1.216216216 11.84419714 0 12.88790633 12.25950208 0 7.481277701 9.353798243 0 0 7.976303819 0 2.264413228 0 0 0 13.27251808 0 19.39476413 0 0 14.23333333 7.74 48.41502561 0 0 9.347241464

652 2.252944188 0 8.025455796 4.536225838 2.685624013 0 0 40.34815299 0 0 0 15.81232662 24.97972425 0 0 14.47675307 0 0 6.031075105 0 0 15.86622286 33.33333333 3.894654614 1.731282483 33.33333333 0 0 21.00844368 0 7.575447919 0 0 17.4238965 0 0 0 19.95672649 0 0 0 0 0 0 9.190290902 0 0 3.89282646 0 31.91881808 26.44305189 0 6.847392939 14.72405895 1.904034133 0 0 0 30.20318883 17.70088647 11.52333333 19.14333333 0 9.910335064 6.73420164 0 0

653 4.360587002 4.918594478 0 9.365959499 0 0 0 0 30.92850335 0 11.25828418 16.7342382 0 7.633892886 7.559388976 0 9.179229481 0 37.74468745 8.961318583 4.435970144 0 25.95647596 0 0 5.222917287 5.948188792 9.963739135 13.27192085 4.948336335 0 0 0 10.36994806 0 6.85882039 30.3425974 8.631164511 29.54410799 0 0 0 39.06483967 10.27655563 0 0 28.6691901 14.04228176 0 0 0 5.327453862 7.750965251 0 0 0 0 0 0 0 2.463333333 0 27.52 0 0 9.194404245 0

654 9.492958144 3.074325163 6.053067993 2.996517491 0 8.543788501 2.383360728 0 9.82518821 10.60770826 0 10.39931668 39.68415096 4.858224808 3.866771262 4.300553737 6.908133624 0 0 23.31073066 0 15.80062852 0 0 0 0 0 0 0 11.21607238 2.862018149 4.060036713 24.58196542 0 0 8.700244608 0 8.705332899 0 21.93943299 39.45803079 12.97686765 0 0 0 15.79360576 9.307657038 6.578801023 16.19980206 0 0 0 0 0 5.828905829 0 22.7295139 3.530918405 0 15.44909123 0 0 7 0 13.88969288 0 0

655 0 0 8.079773747 0 4.836564926 7.333006696 0 0 0 0 0 0 0 6.522005639 0 12.76446334 0 36.34127974 0 0 0 19.90746494 7.520650654 0 0 0 4.023430819 20.65369786 33.33333333 11.69449598 6.667964567 13.20746956 11.52568247 10.27105073 0 4.804702956 14.31243124 0 6.094801223 24.70682053 6.816503113 18.86985355 0 0 18.98784198 0 0 12.39261168 4.949253051 0 0 0 1.821773486 0 3.757124208 12.91208791 0 0 0 12.23260222 0 11.85 0 0 0 17.52190238 10.08225638

656 0 0 0 0 2.566334591 0 0 0 0 0 3.159263705 16.59909513 0 0 3.926788686 0 0 0 4.199444249 0 2.470027445 0 7.376857377 2.592795472 1.674110278 28.11041605 0 0 10.96554077 0 0 5.510913093 0 0 54.9007412 0 0 9.554356311 0 14.28907168 0 0 3.995360227 9.588143781 7.875905215 0 9.939973975 7.308182978 0 22.38974746 0 0 5.203441661 0 0 0 0 0 0 0 6.49 0 0 0 9.933179131 0 7.701083856

657 0 0 0 0 0 0 3.878551191 0 0 0 0 17.52100671 8.353609084 0 0 0 0 0 0 0 0 8.821164949 0 3.187888257 0 0 0 0 0 0 0 0 0 0 0 0 0 13.37660685 0 0 5.907960199 0 0 0 0 0 16.54439859 0 0 12.35810114 0 0 0 2.979264915 0 0 6.418474688 0 0 0 0 9.29 0 10.04458432 0 0 0

658 0 0 0 0 0 0 0 32.83548934 0 0 0 14.05794007 0 7.165624617 0 0 0 14.01243424 28.92197922 0 0 24.5194274 0 0 0 33.33333333 0 0 0 0 0 0 0 38.87282211 0 13.35334668 10.08689867 0 0 0 0 0 33.97721901 23.05677771 0 0 10.94798658 17.40073407 0 0 0 0 29.24714662 0 0 20.42124542 0 0 0 0 0 6.523333333 19.73666667 8.207056742 0 15.66666667 0

659 77.34938676 92.00708036 75.34842174 74.1117447 89.91147647 74.23858321 93.73808808 14.99085923 56.9353088 51.62673973 83.48361258 0 26.9825157 31.6800724 67.25339514 68.45822986 61.8489061 13.9895439 23.10281398 28.20658944 81.35563983 0 15.2141314 87.65924116 96.59460724 0 78.05890018 69.382563 21.42076137 61.59991646 67.24976905 68.98653094 47.37600353 23.0622826 16.98899029 47.26191897 20.34626421 33.8129586 38.93531597 11.39390034 20.08445946 30.82723056 22.96258109 44.19061656 51.68645982 84.20639424 8.802417832 17.04789519 71.06860715 14.13681012 65.58064429 78.58622598 46.86486682 75.63075803 88.50993583 27.85127677 43.68425378 66.91345801 50.40204705 54.61742008 58.03666667 0 9.48 23.42299827 23.3305118 39.95036004 67.79861846

660 0 0 0 0 0 0 0 0 0 0 0 0 27.81096937 0 0 0 0 0 21.78403756 0 0 36.82626169 26.49549735 0 0 0 0 0 0 0 0 0 0 0 0 0 0 0 0 0 6.838453915 25.29218584 0 0 0 0 0 0 0 36.76910099 0 0 0 0 0 0 6.353685651 0 0 0 0 10.00333333 0 0 0 0 0

661 0 22.73975196 0 0 0 0 0 17.51141553 10.41903678 0 0 6.6224381 0 10.3423085 0 0 0 18.08022417 0 10.65267395 0 0 0 0 0 0 0 0 12.93970299 0 0 0 18.78061138 21.90133862 0 0 0 0 29.69729592 6.499197362 10.97201767 15.43543544 0 15.43499003 0 0 28.06885624 0 13.78138075 0 0 0 0 0 0 26.10231077 0 10.02362777 0 0 0 0 0 0 0 0 0

662 50.54291239 0 33.33333333 12.66622778 0 16.74867054 0 13.8690575 0 14.47114057 0 25.62071592 0 0 5.599809368 8.803885853 0 0 0 0 23.9995433 0 3.950249628 33.33333333 0 24.87889944 23.73135447 41.74334988 13.56335515 0 0 21.42602496 0 10.00516396 0 0 12.6292065 9.728322759 0 3.867327278 0 0 17.9305696 0 10.17260894 0 18.93634166 42.85271478 0 0 14.79462014 0 0 0 0 0 7.857798767 0 0 12.27676983 10.34666667 0 10.39666667 0 21.78448968 5.651765338 0

663 0 0 12.69472416 0 24.16803055 0 0 0 28.01334167 0 0 10.95446827 14.12192812 40.98810818 0 17.00976128 52.70741669 0 0 43.47046809 33.33333333 0 0 0 0 0 0 0 9.973966808 0 13.52046784 7.765667575 0 19.79279605 22.89748312 7.978723404 45.43084067 0 0 16.39076708 0 0 0 0 13.22834646 44.44444444 0 12.56108473 6.084379358 19.38128795 0 0 18.43239914 18.65828092 10.86665744 0 7.739011459 0 18.32061069 13.86474323 5.713333333 17.37666667 26.44333333 0 46.40357192 0 6.013214524

664 0 0 0 0 5.77713636 7.962181591 33.33333333 0 0 13.05294323 0 22.64620093 0 0 0 0 0 55.75554786 28.84725006 9.186313974 33.33333333 0 0 0 22.75955871 0 22.51873643 0 0 62.08205727 0 0 0 0 40.70654305 0 0 7.744616547 0 8.586055794 43.06229278 0 8.309438471 22.96876316 0 33.33333333 21.66193182 0 11.2825702 0 11.05103629 13.65412297 17.29979763 0 0 8.404669261 0 0 18.71396896 2.326839827 0 0 0 14.86707566 0 11.02110424 12.63707572

665 16.12375428 33.33333333 0 33.33333333 0 0 0 0 0 0 33.33333333 0 33.33333333 14.53767481 44.42401961 41.19177726 0 5.07783791 0 23.19619857 0 7.506928546 41.06820556 0 0 62.57400829 0 0 8.681338343 0 0 33.33333333 8.378741215 20.36563788 0 14.26324585 0 35.71175364 48.53709533 16.11615745 0 0 0 3.339404979 56.49405772 0 8.719669953 16.32099414 33.33333333 0 0 0 0 16.17154507 66.66666667 14.42300665 38.94489205 0 15.29642974 18.96919004 7.663333333 1.973333333 11.53666667 33.33333333 5.967753702 0 0

666 13.26925836 0 53.97194251 54.00043889 9.165302782 49.91799612 33.33333333 0 33.33333333 33.33333333 0 0 0 25.67855849 0 16.32357205 0 0 33.33333333 0 0 0 0 0 20.69417158 8.454433895 42.9353122 13.20604614 43.12934471 0 7.119328445 25.70245964 33.33333333 0 10.43585021 0 20.70412684 0 0 0 17.82283016 25.93904539 33.33333333 0 20.10498688 0 14.39699167 7.492957746 13.46757322 30.038658 33.33333333 66.66666667 0 40.48224139 0 0 17.57838121 46.2462135 15.01272265 0 21.29333333 7.096666667 13.28 0 0 0 0

667 0 43.9269147 0 0 0 25.37115174 0 0 0 18.86219276 33.33333333 15.75642696 5.522363959 0 33.33333333 0 9.010021542 0 1.687111919 0 0 19.75863504 0 33.33333333 33.33333333 4.092658378 10.8145969 0 0 0 0 0 39.50731407 14.39452621 11.47578609 77.75803075 10.61157208 25.58871679 13.67214748 16.94256625 17.22277282 0 19.38786025 0 0 0 0 0 0 0 0 0 14.9009342 0 22.46667589 33.33333333 0 0 14.61936438 0 0 12.58 0 33.33333333 0 55.64556242 20.69625762

668 0 0 0 0 27.55619697 0 0 33.33333333 22.91429655 0 0 10.68713241 19.21140521 8.453350031 0 0 4.949228432 21.08639006 0 0 0 22.33347644 0 0 0 0 0 33.33333333 11.712292 24.29753657 0 0 0 13.54053728 0 0 0 21.22659027 0 0 0 0 0 33.33333333 0 0 8.216208657 0 0 0 0 19.67921036 16.0335357 17.16178826 0 17.73667998 13.6290775 33.33333333 18.03690359 39.63111591 0 0 0 18.46625767 13.561607 0 60.65345214

669 20.06407498 0 0 0 0 0 33.33333333 35.28619364 5.319991661 20.28039011 0 7.712617416 0 0 16.64283769 16.67100356 33.33333333 0 14.34826713 13.49434541 9.333790033 13.57469829 28.48604747 0 23.21293638 0 0 11.71727065 0 13.62040617 46.02687038 11.7725145 0 0 14.48433753 0 10.62425391 0 8.093461265 31.59792879 4.081632653 33.33333333 21.03879835 24.92350851 0 22.22222222 0 20.77224861 22.05076313 13.81095306 40.82101024 0 33.33333333 7.526144351 0 0 7.897153352 10.3968254 0 12.93134116 18.09333333 11.28333333 12.63333333 0 12.2825777 27.681568 0

670 35.32629746 0 18.22363296 30.48009703 21.85451955 6.662894327 6.987989393 0 17.06287008 0 11.5865451 0 0 8.445051308 24.014587 0 0 0 0 3.63126374 16.93898723 0 10.89197361 11.2657389 0 0 9.639693441 9.907770257 0 0 19.25422214 0 21.06835152 0 17.01356529 7.119239512 0 16.59007203 0 17.78206089 0 11.91803279 0 0 34.01283104 0 10.78538386 0 0 6.593001842 0 4.824712314 5.351114112 0 13.48823043 0 0 5.576769381 0 0 0 0 6.816666667 27.48042642 0 7.050536384 0

671 0 0 4.406446541 4.005167959 0 0 18.38530067 0 12.71335341 0 21.93239938 11.18834924 18.06304529 23.50133892 0 7.104332449 14.55147808 4.529544987 15.44067692 0 22.10863371 5.184759717 0 0 0 17.70046272 6.155167478 0 0 29.43702419 0 16.80914688 0 14.88231255 0 12.86117947 6.26594487 0 0 9.738868624 26.65491447 12.35329873 4.380703094 0 0 14.18864908 9.596291429 0 9.118869493 0 0 9.928680781 0 13.85790995 0 0 4.672507301 0 0 14.99773154 0 0 0 0 2.562710392 23.33400851 0

672 0 36.71802217 0 0 0 19.37016482 0 19.52503761 16.31610837 12.40844464 0 0 13.8578659 0 0 13.75872841 0 20.77251953 0 13.85820165 0 10.95297315 24.73678831 35.12421194 40.45219956 32.05962568 0 0 12.4804337 0 0 7.060969255 0 0 19.36375266 22.59107933 14.8058375 11.298027 36.83904487 0 13.41884188 17.39101342 20.29337872 11.93125424 0 0 15.39102703 32.00870257 18.27463987 10.33244812 15.29268563 19.61911828 16.97456951 11.75949249 0 29.1533382 0 7.740350877 9.15078805 0 0 14.13333333 0 7.748267106 13.76176566 0 12.73728697

673 0 27.61849035 16.94116127 0 16.92624155 32.56887854 36.69084386 0 0 6.321561039 23.37610985 0 17.70100235 15.02877128 8.87043019 12.21927498 20.96301648 0 14.36541144 0 0 23.24227022 0 0 26.3973198 0 19.3884731 10.14527174 17.37160632 0 0 0 16.9849472 0 0 17.52944823 11.49052524 0 0 0 0 0 0 7.747538587 25.2552757 0 0 0 7.003378572 0 11.22972526 0 5.531172631 15.64502479 14.88368925 20.46702663 27.00400605 0 11.32566153 33.33333333 6.706666667 0 0 14.69111548 7.376841496 26.83504966 0

674 3.07957625 0 35.72765521 0 37.8760538 0 0 15.95808521 16.27046325 0 0 28.2436138 0 0 0 0 0 25.26217665 9.535701073 0 0 0 16.28141299 0 0 0 25.2379804 18.89544187 0 0 22.11726384 19.05468419 10.72551842 0 16.31976804 0 0 0 0 43.77279833 0 0 22.55431521 7.47716895 0 0 7.30287437 33.33333333 0 0 0 0 0 0 33.33333333 0 0 0 0 18.33560179 13.23333333 0 17.05 0 0 0 33.33333333

675 8.625584502 0 0 0 0 0 0 19.58429705 0 33.33333333 17.80220889 0 9.76283097 18.26441221 45.82816591 0 0 0 6.732135137 31.41085807 34.76381206 20.88071989 0 0 0 9.304541124 0 33.33333333 45.12656053 33.33333333 44.73390161 0 0 0 0 0 29.08534448 18.80625541 9.802944758 7.830959774 2.059284595 21.41530055 28.95263024 21.40207909 17.65955888 27.5301226 0 14.43315213 7.950443859 37.03388287 0 22.07821998 0 10.14553596 0 0 0 0 0 0 21.21 4.68 0 9.670377647 0 0 0

676 33.33333333 11.73118583 0 54.15126985 10.11852287 13.96316852 14.94803267 0 37.63720488 32.36147788 0 0 0 0 15.83346152 52.90793826 0 0 23.79763226 0 14.84538133 0 8.596545027 0 26.21446711 0 0 0 15.96172702 0 0 0 0 46.31201188 25.80097602 20.47215386 0 0 29.11228688 0 15.23003338 20.9800346 0 0 1.991150442 45.12082642 0 8.628812299 26.32995476 0 40.77311792 15.88066166 0 0 32.31402426 43.77573097 39.24963739 53.34954641 45.52694883 0 4.363333333 8.866666667 9.466666667 9.663029305 0 18.08385371 53.9293797

677 12.18280365 23.93230165 0 0 0 0 0 17.37524812 0 0 0 27.23470363 30.90261903 18.10940797 0 0 45.70365019 28.80378835 18.9679219 0 0 21.40330114 0 53.61004916 0 31.3309105 13.94486023 14.43789146 0 37.22964248 0 26.27236408 6.41393835 24.33226043 21.50193798 10.742254 13.91326466 0 0 0 19.91449145 0 0 25.58579475 0 0 0 0 0 17.26693618 0 13.71421505 38.80981042 0 0 0 10.05226693 33.33333333 0 33.33333333 14.21 17.63666667 3.453333333 14.82474712 27.69043689 0 0

678 7.45240481 0 17.93238994 11.36346516 0 0 22.98783341 6.125080593 0 0 15.3455133 33.33333333 0 0 5.453355381 0 18.78185525 12.5608138 11.16052128 19.47513168 0 0 39.49328006 0 6.936013534 0 6.867721708 13.28029134 0 0 0 30.8028356 22.60781492 0 0 8.684645588 0 31.27033922 0 13.15499971 0 0 10.77901813 0 6.086907193 0 41.6793482 11.59599967 24.21446384 12.80540209 22.10360808 6.43040104 33.33333333 17.68830855 0 6.603904195 0 0 24.18254528 0 0 4.876666667 8.69 15.92203692 33.33333333 0 0

679 0 0 6.768714073 0 13.22466222 27.4348938 0 21.43225142 0 15.57518311 9.95722348 0 9.712636466 16.65101831 0 14.00972591 0 8.071156685 0 31.62454486 11.34318568 18.33597587 0 0 0 9.60445997 18.76610364 0 9.059672432 0 13.89461241 0 22.19942959 14.47341513 0 0 24.43908325 22.03530633 24.24572349 7.720312667 22.72243422 15.94231992 13.03995462 25.85616438 14.99427675 13.16040191 15.24507511 0 7.108249602 15.96832891 10.60086312 7.523990889 0 30.90372826 5.980722727 0 19.02158233 0 9.814056303 0 8.073333333 6.636666667 0 0 15.27491222 24.69655174 0

680 0 0 0 0 0 0 0 0 8.419064659 0 0 34.4158927 0 0 0 0 0 0 7.394424971 0 0 13.18480085 0 0 0 5.442866802 0 0 0 0 0 0 0 0 14.25306462 14.66416354 0 0 0 0 0 0 0 0 0 0 0 32.63536848 0 0 0 0 0 0 0 0 0 0 3.069088907 19.95543557 0 14.83333333 5.143333333 6.66456096 0 0 3.78397766

681 5.466249907 0 0 0 1.495288816 0 0.403655577 6.127634758 0 2.215457467 0 0 6.099469445 0 3.663681445 8.307487816 0 55.1945435 8.856211666 0 0 0 0 0 0 0 0 8.79901308 0 0 0 9.030712877 0 10.29818373 14.19703104 16.22832651 0 27.82721708 7.778456526 6.031696382 0 10.55089925 0 0 0 0 0 0 0 7.082286432 8.915676192 3.148129542 13.08363035 0 0 33.33333333 6.621229785 0 0 0 2.986666667 4.136666667 13.45 0 0 4.203609054 0

682 0 0 0 1.192227882 0 0 0 1.816560793 0 0 0 0 0 0 1.387779257 6.243745377 0 0 7.069763822 43.38389139 7.600514044 4.683013033 9.789360609 0.549905838 1.250444347 0 4.66323818 0 9.701572361 5.352356247 3.040310636 2.309591643 6.448311157 6.15297082 0 13.1358468 9.260235742 0 0 11.34433802 0 7.656601866 11.26554153 7.339565437 11.33549247 0 0 0 5.028326443 0 0 0 0 0 0 5.953109072 0 0 10.42642467 15.81345622 0 4.243333333 0 9.467314656 34.24065005 0 11.26619236

683 0 0 12.19954649 0 2.555489201 9.52271366 0 0 16.87294303 0 4.510258285 0 0 8.806077746 0 7.1237349 11.20284577 0 0 0 1.227894189 10.59775082 7.557748374 0 1.380748546 0 0 0 9.260493005 0 1.231386025 0 18.46396477 0 0 0 0 0 0 0 0 0 4.06809102 0 0 10.48159797 13.63832809 4.142926549 25.70071143 14.13014608 0 4.300229433 7.119828049 16.08087904 0 0 11.44253332 7.898973078 15.17342856 0 0 0 0 23.08667598 0 0 0

684 0 5.918403193 0 1.735783027 1.682911971 0 0 5.169540948 26.2538371 0 0 0 0 7.779408854 0.91433644 6.958597942 0 0 10.47571357 0 0 12.52352612 12.84163987 5.607407407 0 0 6.835822493 0 0 0 0 12.47534517 0 8.136820223 0 0 33.33333333 33.33333333 13.03891713 19.30820356 44.72219066 0 0 9.345114001 20.36545807 4.062843991 47.16275322 33.64870621 0 0 10.77887939 5.473588342 0 0 4.862805831 8.284155439 6.376109766 0 0 0 11.19 3.773333333 8.643333333 0 0 0 7.639689494

685 0 0 8.160359685 0 2.762984205 33.33333333 3.984398032 22.90403139 16.4603903 7.55495536 20.49453943 27.63576107 19.48198198 0 5.062541633 4.707827525 0 1.907740422 6.74930069 23.28277527 0 0 8.126663864 5.37948871 0 0 14.03081866 0 19.90164067 4.855503186 0 8.789281483 9.170364394 0 45.76698156 18.71649668 0 17.73785838 0 10.15535398 0 0 14.79486893 0 0 0 0 0 0 7.741834171 0 4.591580428 0 8.124957647 1.312829077 0 0 12.4222252 5.31323185 4.988486661 0 11.26333333 4.7 0 0 0 8.483376602

686 21.63173037 3.61455647 26.44156307 14.72677627 0 0 5.141753989 9.071659423 0 25.28101092 5.717017208 0 0 0 4.43139778 0 6.795101553 0 0 0 31.03561502 3.274056839 7.744636316 4.672316384 4.859586391 61.22379986 0 8.604540874 0 33.33333333 14.47794147 0 0 0 19.08026872 17.10500682 10.85036852 0 13.71295758 6.266557978 21.05679702 13.46254584 13.66050563 0 21.99784086 6.23345367 0 9.363544013 8.066146073 29.63768145 0 0 0 3.817237075 1.228270747 0 0 0 8.558153477 0 0 0 8.183333333 0 17.72739213 19.93589073 0

687 0 0 0 0 0 0 0 0 0 0 0 0 0 0 0 0 0 5.270545301 0 0 0 7.625006358 20.49169346 0 0 0 0 0 24.07284033 0 0 0 0 0 0 20.15015965 0 0 0 0 0 0 0 33.33333333 0 28.67203219 19.50391344 0 0 0 0 0 0 0 0 0 0 0 0 0 0 0 0 23.86601868 21.68143604 0 0

688 61.20041676 0 0 0 0 0 0 0 11.10282789 0 22.73946155 11.37420719 0 0 0 0 0 0 0 33.33333333 30.12408791 0 0 0 0 33.33333333 0 0 37.06345364 30.86650287 30.2930227 0 26.88502218 48.23166271 0 0 0 15.60916696 19.48347248 17.14628297 0 0 0 25.9937679 20.94684587 0 19.69500525 9.132205514 0 18.50921273 0 27.85974499 0 29.51609626 0 27.38022426 0 28.28532991 0 0 0 14.74 0 0 0 0 0

689 11.70160296 90.46704034 53.19853076 82.34521282 91.50332581 57.14395301 90.4701924 54.91057268 20.89093702 64.94857625 46.53872353 26.57413905 74.41854857 83.4145134 84.54026344 66.65860644 82.00205268 37.62717078 59.45458528 0 30.01188884 48.11184598 33.4482575 83.79088166 92.50922072 0 74.47012067 82.59644605 0 25.59230437 50.95733918 67.39506883 39.0323375 27.18036251 6.702654071 0 46.55606241 5.492424242 45.98619629 29.74756711 34.22101232 68.32995305 56.21099289 23.98821933 25.35436273 50.55007217 0 11.07724924 61.20481606 22.89883914 80.30544442 54.62672726 79.7965416 42.46082998 92.59609434 25.04917789 75.56012713 51.39347182 57.45967254 59.24262155 78.85333333 38.35666667 16.43333333 36.91542973 26.35052179 75.86050021 68.82676388

690 0 0 0 0 0 22.27225588 0 0 0 0 0 9.528653781 0 0 0 0 0 14.59463292 0 41.17935914 0 16.12447547 16.12481315 0 0 35.94901653 0 0 12.09624258 0 0 0 0 0 44.07782498 56.7936601 16.67266835 0 0 0 13.12151346 0 0 0 13.57213619 15.49924683 23.11996051 8.820832569 0 12.00652816 0 0 0 0 0 23.21811562 0 0 0 0 0 0 10.55666667 0 35.21001957 0 0

691 25.32564412 0 33.33333333 0 0 24.73316331 0 12.22131116 14.96773005 17.2815534 21.70994928 0 13.56455196 14.72389223 0 0 0 0 22.96002643 8.642233857 26.9190326 0 0 0 0 0 0 0 0 14.1099111 18.48904049 0 15.52202957 0 0 0 0 14.40415964 0 0 20.48388908 27.64225955 25.22346088 21.28894319 0 0 29.59187564 43.90821029 0 0 0 0 0 0 0 0 0 0 0 0 0 9.483333333 0 18.59183921 0 0 0

692 37.08029451 0 0 24.37519284 0 0 0 0 0 0 0 0 14.592867 0 0 7.301029048 0 21.1637364 0 0 0 0 0 0 0 21.18528875 0 0 21.69579662 0 0 0 0 7.63172189 0 0 13.54246366 8.330234288 0 22.62215291 0 33.33333333 0 0 0 7.183761411 0 0 0 0 0 0 0 18.6309903 0 0 0 23.34233423 0 22.31031543 5.103333333 0 18.61333333 0 0 0 0

693 0 0 0 0 0 0 0 0 0 0 0 52.01377848 0 23.09333881 45.05815653 0 25.55809234 0 8.106554235 10.2547993 0 0 0 0 0 0 0 16.99649123 0 0 0 13.8947533 0 19.02957583 0 12.82016073 0 0 5.736975423 0 0 0 0 17.91581806 20.53676084 6.477297181 0 0 0 27.32584982 0 33.33333333 0 0 0 20.22077591 0 0 19.27227875 19.55222659 11.54666667 0 0 0 0 23.33225563 0

694 0 0 0 19.85789981 0 0 0 64.42706814 9.472357284 0 0 12.7204137 0 12.75438596 0 0 0 0 0 0 0 21.01240791 19.42980916 0 0 0 0 0 6.873947118 9.947836309 0 0 0 25.70161144 14.08069884 0 0 0 0 16.35894731 20.21181987 0 33.33333333 22.71900407 19.76119714 0 8.03597331 15.54495996 55.36159601 27.38300453 33.33333333 22.2786238 13.40081947 0 33.33333333 24.1599881 33.33333333 0 22.13363705 11.5596175 0 10.04666667 0 33.33333333 0 0 60.19814429

695 0 0 33.33333333 0 33.33333333 26.38033595 0 0 13.52923847 0 44.95671739 0 33.33333333 0 33.33333333 20.269697 0 16.42383636 33.33333333 25.48730752 20.81871345 33.33333333 33.33333333 33.33333333 0 0 33.33333333 0 17.437181 46.39448568 14.84429284 0 33.33333333 0 0 0 16.66066499 0 0 0 0 0 0 0 33.33333333 48.07506946 0 0 0 6.713422434 66.66666667 0 33.33333333 40.7729879 0 0 33.33333333 0 33.33333333 0 0 0 17.07 28.36095764 19.58162605 33.33333333 0

696 9.640715207 36.43264647 0 0 33.33333333 8.60017002 54.67653197 0 33.33333333 49.38511327 0 14.65288818 0 0 0 33.33333333 33.33333333 45.50293027 28.44071086 14.43630017 0 0 17.20852018 0 100 32.2309238 18.7230371 9.263157895 11.63753671 0 18.26451227 0 17.81130376 33.33333333 10.65952675 21.02350762 43.12148303 52.26250703 22.88342722 8.732808119 5.978443921 19.69742511 33.33333333 0 0 11.35678933 15.5985591 22.75845637 0 13.65602129 0 0 0 0 0 0 0 33.33333333 25.26075086 0 3.52 5.453333333 21.05 9.601646688 22.52386539 33.33333333 0

697 10.59640523 0 0 13.47543353 0 0 0 23.3516207 0 0 33.33333333 0 18.74046633 49.428383 21.60851014 0 0 0 0 0 6.414300736 11.69969671 0 0 0 0 0 33.33333333 0 0 0 52.77191336 33.33333333 14.3037575 26.50511574 0 0 25.00309905 33.33333333 21.62496508 0 19.326982 8.109872452 22.65871941 0 0 23.65363144 8.967540803 11.30507066 0 0 33.33333333 53.26584719 33.33333333 0 19.09789335 33.33333333 33.33333333 0 21.77371583 2.863333333 16.13666667 0 0 0 0 15.2833024

698 9.349251724 46.77819083 33.33333333 8.958140492 0 18.01407484 0 0 10.9441744 0 0 11.08426585 19.76878137 0 0 39.09594062 0 0 7.159375147 0 0 17.83008658 0 0 0 0 0 33.33333333 14.36314363 20.27218098 33.33333333 0 0 0 0 9.362671556 0 0 0 16.97438603 0 0 0 15.41751527 12.79657249 11.4078358 0 0 33.33333333 6.964844956 0 11.05470953 0 7.262688469 33.33333333 13.30322702 0 0 0 24.80412465 0 0 0 4.972375691 0 0 0

699 8.007689218 16.7891627 0 33.33333333 33.33333333 0 45.32346803 0 17.75316646 0 0 0 0 0 0 0 7.775240994 2.31486405 0 0 45.84795322 0 13.90352418 0 0 10.63477093 47.94362957 7.073684211 15.89615234 9.275585923 15.06882107 33.33333333 0 0 4.676833696 0 10.00271998 0 38.04626403 13.68674055 40.20433367 0 0 0 0 0 0 0 0 5.9503288 0 0 0 0 0 0 0 9.9909991 0 0 4.886666667 0 0 5.139847439 22.68448899 10.0010777 24.51855331

700 0 0 16.05646037 0 0 0 0 21.67258926 0 15.23824001 10.82741343 9.659850134 10.89896579 22.74011299 0 0 0 4.58364119 0 0 0 0 21.3107116 31.83572136 12.43694218 9.971246663 0 11.72957987 0 0 0 0 0 0 9.55826926 0 0 0 0 0 2.83321454 6.448959845 0 6.785622921 17.1132604 0 6.611988659 9.110827095 17.43761147 0 10.34499514 4.781542577 0 6.163194444 17.29981378 9.401709402 13.04121022 0 1.711261896 0 0 3.11 3.963333333 0 0 15.16998856 0

701 0 0 0 0 0 11.53755592 0 0 0 0 0 0 11.37203021 0 0 0 9.579870729 0 0 0 0 18.96338356 0 0 0 0 0 0 0 0 0 8.934907962 9.572291261 0 0 13.70048527 0 4.452335427 0 0 0 0 5.464259147 0 0 0 0 5.07663612 0 0 18.25976263 0 0 0 0 0 0 0 3.261533761 6.134838186 0 0 5.89 0 0 0 0

702 0 24.24611224 0 0 33.33333333 19.28007384 28.62749334 27.44752161 29.3114985 0 33.33333333 16.44774415 17.74794128 12.29177782 16.3512468 0 0 0 0 13.34075724 0 60.28827038 36.48888071 12.87958115 0 0 29.89490074 24.48318567 0 33.33333333 26.07942238 0 16.90510253 0 20.40052636 0 0 0 0 0 0 20.41282597 17.45937962 26.54771041 0 17.1868283 30.44750055 0 0 24.54351331 0 33.33333333 72.05587145 20.91957923 41.32588454 20.80101537 0 0 9.418341655 0 28.48 0 8.023333333 0 0 20.94358462 0

703 56.50366588 52.05634225 75.31377266 27.63085399 51.98969117 48.35728911 56.88080711 22.00460194 0 51.53431962 55.83925323 40.16039856 19.21614023 0 26.01032521 63.02725221 27.00976511 61.19211293 85.96827872 33.33333333 66.66666667 0 33.33333333 13.92324303 69.34266384 80.27397651 66.66666667 23.43708957 74.01389586 50.38020801 48.89964428 58.83606557 23.11931662 18.52587757 26.94809786 56.46475381 21.55890884 52.75097749 51.75494382 61.46434331 56.45355423 17.75966184 57.78899937 53.61131196 66.86245238 61.74855203 13.53062063 44.15437409 42.73367081 51.72957764 38.1949186 43.89284118 0 16.74952652 27.25591398 0 0 89.76097349 20.41933068 25.00719011 35.92 20.43 16.67666667 33.33333333 33.33333333 26.52149637 26.25996493

704 33.33333333 0 0 50.05970421 0 0 0 0 18.19489392 0 0 0 22.43436754 21.21040937 33.33333333 18.13447624 37.13940986 0 0 33.33333333 0 10.82477979 0 0 0 0 0 0 0 0 0 11.21922821 23.76104207 66.66666667 23.77506407 14.71993379 33.33333333 28.88099791 0 0 16.96958898 26.88437349 0 0 0 0 0 12.0590382 12.74148226 0 0 0 0 33.33333333 0 45.21990851 86.95878978 0 0 46.67555225 0 7.28 14.16666667 62.37026309 16.1593964 14.88775739 22.48211337

705 0 0 0 0 0 0 0 5.1452141 22.48788027 16.76927587 0 0 12.64232009 5.024008536 7.17728977 0 0 0 0 15.03340757 33.33333333 0 4.178736187 12.42582897 0 0 0 21.60375347 15.71853667 0 0 0 9.434112224 0 12.93280697 0 33.33333333 0 24.59823282 9.518842092 0 20.9217151 0 0 0 8.077551371 26.72134467 0 0 0 15.07357071 0 5.171014493 0 8.040968343 0 0 0 20.12113646 0 0 0 0 0 18.26094525 0 0

706 0 9.087221095 8.629766973 0 14.67697549 0 4.705839994 0 0 7.307525011 0 0 0 10.59322034 0 15.19885709 0 4.75354174 11.89666667 0 0 6.378396289 0 0 0 0 0 0 0 13.23505324 7.253910951 0 10.21401672 14.80745577 0 0 0 0 0 15.70922755 0 0 6.128344001 0 0 0 19.8027127 5.212752282 0 8.657874321 0 0 10.61659232 0 0 12.53231796 0 10.23902651 12.91400265 0 0 4.9 0 0 0 12.38974871 22.90775083

707 10.16300079 3.851438733 0 0 0 6.771821632 0 0 0 0 0 16.88558919 2.745162891 0 0 0 6.323568224 0 2.135054618 0 0 3.545169979 0 13.65293997 13.01901436 0 3.438432594 9.896243761 0 3.051405419 5.150600815 7.830601093 6.994118582 0 6.385235469 15.11482713 0 0 0 0 19.93806309 0 3.413408148 0 0 0 0 0 6.585879874 6.279214709 0 5.037163282 0 22.83436647 0 12.04504876 0 0 6.887722443 0 7.84 0 0 4.296403572 17.17393693 0 28.35017086

708 0 0 0 0 0 0 0 12.90386227 14.8672879 0 0 4.738023595 0 0 0 3.639414454 0 0 0 4.959168523 0 0 4.688338161 15.28268551 0 9.754776824 0 0 10.26756746 0 0 0 0 0 0 0 0 13.91568917 0 13.30758705 0 7.572463768 0 13.0553547 16.02428722 8.068953658 0 8.188713202 0 0 0 0 0 0 0 0 0 0 0 8.326143227 0 2.833333333 0 0 0 10.08742434 0

709 0 10.75888569 0 22.3094418 0 14.05325949 9.785859553 10.82621083 15.13843941 9.150639492 0 12.10839437 2.943071966 28.14047094 17.12780488 0 19.94738608 29.47070414 0 0 0 0 0 0 5.201379617 0 0 8.850147661 0 0 12.61642157 13.17919716 0 0 0 0 11.7744245 0 23.64682336 0 3.805579156 0 9.745609716 0 0 4.91811464 2.885832788 16.19765902 20.50135558 8.78982002 18.12675293 12.95511963 12.15652174 0 6.077419355 0 0 0 25.26667045 13.85627623 2.34 0 14.01333333 0 15.07238808 0 0

710 10.32366318 43.6264776 28.06831682 0 57.49619482 13.57030175 16.96869283 0 0 0 7.329083362 6.627818836 12.91769131 0 9.911485084 0 0 0 29.22039239 9.212367986 7.617337779 21.16777686 0 20.77493817 14.43141741 27.72877298 11.90281682 15.38259958 26.17848592 12.70608398 28.74914312 0 30.90447386 25.85264965 19.7450668 27.93178898 14.97890295 14.88293338 0 18.97140952 19.74255524 34.09744776 15.75598505 16.26858618 12.63437171 0 13.32075811 0 0 20.36142815 16.36000571 15.78434557 34.51542186 24.64815607 9.179288188 0 42.76122377 7.577686613 17.7859029 0 12.28666667 16.74333333 14.04 37.32947475 15.05747126 22.4262059 0

711 0 0 21.31330527 0 0 9.082756854 8.01611279 7.800135655 28.07588509 0 21.75541976 0 21.70843776 0 0 14.36930195 18.55367755 0 12.1817391 8.892579206 0 0 35.99888699 0 0 0 0 0 0 20.55429361 0 0 33.33333333 5.543791063 7.02509896 0 9.924134661 0 0 16.70996796 0 7.069374835 12.14359985 0 0 6.167355899 0 0 11.10771114 12.0354748 11.41468641 0 0 0 0 11.53251187 0 4.981081221 0 9.598487376 13.45333333 0 0 0 0 0 0

712 17.87770891 0 0 3.44491112 19.37514729 0 0 10.13234743 0 15.2371599 0 0 4.696462255 17.88640212 0 0 15.93426224 8.520575643 0 0 17.01437181 0 13.68523949 25.85282651 15.99539701 0 19.6129405 24.67864867 0 0 0 33.68149081 0 0 11.7035217 0 0 33.33333333 0 6.322052808 35.09857625 0 0 8.918057581 0 21.72217222 32.25960515 35.66473144 16.96298781 0 0 16.18530295 13.85421732 19.79018492 33.33333333 0 0 0 0 17.796342 0 11.87666667 6.966666667 9.471892538 10.62093266 13.8197335 9.177542987

713 15.06410256 0 1.878103476 5.040495905 0 16.17827689 33.33333333 0 0 0 0.827000447 5.464712961 0 18.59088218 10.23024594 16.60633686 0 0 0 0 9.739588543 30.02435964 0 0 0 0 0 0 9.021764032 0 9.627207325 0 4.726812565 33.33333333 0 0 0 0 63.59507764 0 0 17.8991278 0 9.28563542 5.846027172 0 0 0 11.56095367 16.15980025 0 11.12968047 19.82410139 3.188910512 36.77137478 43.64993565 7.993628988 10.69783918 0 23.73484596 0 4.453333333 0 6.451093951 0 15.49543418 29.29435008

714 0 0 0 16.84735643 0 4.539243317 0 36.08007423 0 0 0 9.522455941 4.710931096 33.33333333 20.79979558 15.93628594 5.259705179 0 6.286563884 12.68609238 0 0 6.772021288 12.55839516 0 0 43.70659159 0 0 13.49647757 0 16.10151687 0 6.374545257 10.41802601 8.414181339 0 0 26.31111111 0 0 0 0 0 43.28234693 33.33333333 0 10.95817541 0 9.093662124 0 0 0 0 0 0 13.87384338 0 16.92318216 0 7.74 0 0 0 9.417721519 10.90712743 0

715 0 0 12.02002806 0 9.170471842 0 12.08862034 0 0 33.33333333 3.830418884 33.33333333 34.07129046 0 0 0 0 35.48464288 29.96957043 0 0 0 0 16.71693236 17.62463064 0 0 0 4.367526158 28.40604603 16.68763509 17.65249538 6.76910299 0 21.62981163 25.44373447 27.49122969 33.33333333 0 0 0 0 17.57734829 9.611350337 0 0 20.43612856 15.6424581 6.668915879 0 0 0 12.32714342 0 0 0 5.236175402 36.03839348 23.75979514 33.33333333 0 3.806666667 10.31666667 0 0 0 0

716 5.270561343 47.31588287 24.68364568 0 0 36.91808803 0 0 33.33333333 33.33333333 0 0 0 0 0 0 33.33333333 0 0 33.33333333 39.91270631 15.8695119 19.7539754 0 0 33.33333333 0 41.98801799 0 0 13.9715898 18.98409441 0 0 13.58826654 13.29171084 0 0 0 16.62336538 13.5907781 40.93404961 0 0 0 0 0 15.35947712 0 12.97190518 0 0 0 13.54314841 0 0 0 7.917109458 0 0 8.813333333 19.93 22.23 16.50579151 0 0 0

717 18.26923077 9.057639524 12.03660069 58.18125964 13.95818604 19.71133316 0 45.98744269 16.43927533 18.09617343 25.82455378 0 6.19047619 15.44693121 35.63662514 35.69102786 17.3990711 0 13.3627756 0 0 0 12.87607255 24.0969078 0 38.93789369 24.77765109 17.95073375 35.58637188 0 0 13.58040254 24.26627725 21.41499701 0 0 6.672694394 18.45039995 0 0 21.12613216 0 0 55.91637048 28.15097967 0 20.01257523 22.37515792 15.10346379 17.17353308 16.97332763 39.75264063 19.47911601 0 0 21.80082146 0 0 16.41015117 15.53699133 9.226666667 0 12.13666667 30.24174726 42.19147388 17.83789915 41.0428383

718 33.19473323 0 0 16.4859769 0 0 29.5932407 0 0 0 40.43352377 45.05167893 15.70471093 14.74245115 23.42184825 17.3970474 9.519950606 0 8.978958596 35.8756271 25.71599555 18.66348449 10.91380428 0 51.94855495 0 0 0 24.84585202 24.83709882 7.258298655 0 0 7.480683681 15.89020836 7.889598864 40.9330383 0 10.09381124 41.37320434 10.44195826 0 54.52306682 0 10.08627451 38.77713855 13.97093296 0 38.59596772 12.20419641 55.25198025 17.14803038 0 38.82960008 20.7160037 23.01673102 30.13512846 32.78789005 25.12096863 0 3.306666667 6.16 0 0 0 19.51359983 20.48526863

719 0 0 0 0 0 0 0 0 22.15150625 0 0 0 0 0 0 0 0 22.66144814 0 0 0 14.27486712 0 0 0 0 0 0 0 0 23.70612601 0 0 0 0 17.02898551 0 0 0 0 0 0 0 0 0 0 0 0 0 0 0 0 0 0 0 0 0 0 0 0 0 17.84666667 0 0 22.71240068 0 0

720 0 0 0 0 0 0 0 0 17.50408842 0 0 0 0 0 0 0 0 25.23865415 0 0 0 0 0 0 0 0 0 0 0 0 0 0 0 0 0 0 0 41.23458785 0 0 0 0 0 0 0 0 0 0 0 0 0 0 63.67117316 0 0 0 0 0 25 11.42428653 0 0 9.49 0 0 0 0

721 76.8534405 93.32459636 83.10966927 76.99352801 77.81039184 79.5904371 81.92098931 43.36057223 21.01861825 90.08143916 86.84650895 80.17364302 77.46300981 95.30612245 85.72218646 78.35879988 79.94465132 20.34682988 67.66304713 33.03804211 80.44572643 33.06613812 76.82448992 91.71779141 88.30608761 35.27438386 80.58920684 50.86532058 39.64419188 65.93351947 86.97707578 54.3402303 67.38503744 47.9013329 55.73918763 11.57619359 84.0941278 26.2962963 74.86872226 43.15193876 20.8942107 51.78004544 68.78098249 84.79221594 51.59818374 85.68168177 71.17794408 64.14556932 71.24120031 48.36297573 66.28760478 48.42164743 31.16367677 89.40712408 95.92814371 84.48530991 51.89860503 37.4167036 56.69463381 47.43938951 44.86 27.39666667 18.31 48.93441658 23.00770376 61.73123815 75.42932932

722 0 0 0 0 0 0 0 0 0 0 0 0 0 0 0 0 0 0 0 0 0 17.18301779 0 0 0 23.8374365 0 22.66763848 27.62467192 0 0 24.22859887 0 20.71953604 0 0 0 0 0 18.06440503 45.27931673 26.63668968 0 0 0 0 0 0 0 33.33333333 0 33.33333333 0 0 0 0 0 22.75231379 0 0 20.03 0 0 23.02801804 43.26013653 0 0

723 0 0 0 0 0 0 0 0 0 0 0 10.26414588 0 0 0 0 0 8.094679186 0 33.33333333 0 0 0 0 0 0 0 0 0 0 0 0 0 0 0 31.66086121 0 12.1945223 0 0 0 0 0 0 20.28411737 0 0 0 0 0 0 0 0 0 0 0 0 0 0 29.94344358 0 0 0 0 0 33.33333333 0

724 11.04678639 0 0 11.74206403 0 0 0 20.27058549 10.46072295 0 0 0 0 0 0 0 0 0 0 0 10.07298753 0 0 0 0 8.861358455 0 0 0 11.84155078 0 0 7.407886789 0 5.622653458 0 0 0 0 0 0 0 0 7.784055242 25.24159269 0 0 0 0 0 7.222741595 10.32919198 0 0 0 0 12.32924694 11.75062378 9.972032861 0 0 6.216666667 11.96 0 10.32562957 0 0

725 7.362717167 6.675403636 12.6048238 0 0 5.112234105 9.997111156 15.37347889 0 0 0 0 3.659103393 0 3.527927403 0 0 5.269146265 6.210033904 15.73474258 5.707901686 21.72209027 0 8.282208589 8.817180697 0 0 10.8651134 4.164948454 8.747237804 0 9.104734462 13.37715505 0 19.23675126 8.804385063 0 0 12.02681388 29.8256414 0 12.66814174 18.25579295 7.423728814 0 0 3.674486195 0 19.04721576 11.27488224 0 0 0 4.934175882 4.071856287 5.135802469 13.71495118 0 0 0 0 8.006666667 6.57 7.185628743 0 4.93542852 3.483906826

726 0 0 4.285506931 0 3.909977183 0 0 14.58839659 12.31471508 9.918560836 0 0 11.21735075 0 0 3.967692489 13.85622533 0 20.43062585 10.45840101 0 6.849620705 14.81930756 0 0 13.74588838 6.706766917 10.66569485 5.708661417 2.317335697 13.02292422 0 0 2.889027952 12.82882883 19.86052037 9.939700447 7.037037037 1.513461845 0 4.18909517 0 10.1709281 0 0 0 14.23137015 29.24731045 9.711583924 7.028808698 0 0 0 5.658700038 0 0 0 5.490671368 0 5.670603001 0 0 0 17.73225009 23.40653013 0 0

727 2.539513408 0 0 0 0 0 0 0 5.368521965 0 3.762392329 5.818101851 7.660536047 4.693877551 7.535350844 12.04445869 6.199123356 7.717357188 0 0 3.773384354 5.05260909 0 0 2.876731694 3.226222738 3.272687538 0 0 0 0 0 2.683579256 0 6.572578821 0 5.966171752 7.346815504 7.751588286 0 0 0 0 0 0 0 0 6.607120233 0 0 2.654548727 1.301758759 2.995493505 0 0 5.856841764 16.85250278 12.2865805 8.333333333 0 0 0 12.57333333 0 0 0 0

728 2.197542533 0 0 0 2.958819715 15.29732879 0 6.406966799 0 0 9.391098725 0 0 0 0 0 0 33.33333333 5.696293116 0 0 0 8.356202522 0 0 0 0 4.936232682 6.219725648 0 0 0 0 12.61379729 0 28.09803977 0 0 3.839413733 0 17.4822918 0 0 0 0 0 0 0 0 0 0 0 2.169656559 0 0 0 5.204694079 5.090348171 0 0 0 0 0 0 0 0 0

729 0 0 0 11.26440796 15.32081126 0 8.081899537 0 0 0 0 3.744109243 0 0 3.21453529 5.629048941 0 0 0 7.43548096 0 16.12652404 0 0 0 15.05471007 9.431338703 0 16.63780069 11.16035625 0 12.32643636 9.146341463 15.87630582 0 0 0 5.890741014 0 8.958014818 12.1550856 8.915123143 2.792296464 0 2.876106195 14.31831823 10.91619957 0 0 0 23.8351049 6.614068499 0 0 0 4.522045855 0 5.212758783 0 5.522277369 5.523333333 7.04 0 3.119686553 0 0 21.08676386

730 15.78655852 26.90414133 33.33333333 0 0 4.009885697 19.54193819 0 7.689444173 0 0 11.9581981 34.02927767 49.14976957 13.15585297 0 0 0 8.300614115 12.18161683 0 0 9.597711643 9.404920616 35.8418003 20.71880454 0 0 20.4458702 0 0 25.38037118 33.33333333 0 4.163874369 0 13.58119331 8.425422441 2.784448256 0 0 34.13521165 23.42354425 31.19283391 6.462086301 0 19.83077469 18.16523377 13.47861178 27.70048949 0 33.33333333 17.91014351 0 6.0283314 0 7.082549865 0 2.36534745 0 0 10.52 0 0 17.3798592 9.914208975 0

731 4.312721544 23.80811225 43.14188371 0 33.33333333 0 22.87504433 20.73304158 0 17.33069307 23.91039653 0 0 0 0 0 3.522012579 44.30354666 0 0 11.46690386 22.20507545 0 18.65300702 0 0 9.759283448 17.70062272 0 0 31.30470623 10.43885656 0 0 26.4911343 0 11.88875306 0 4.306334248 10.36734694 0 0 0 0 21.63614584 9.11823184 13.88216303 0 0 0 0 0 19.9110962 23.09660909 19.39850752 31.48096122 0 0 6.177606178 13.73380014 9.496666667 0 8.473333333 0 0 20.30940817 0

732 0 16.99684827 0 48.65813895 9.162623362 10.00967269 0 0 0 13.30730953 0 0 0 0 0 0 0 0 4.540271276 0 17.36960415 0 12.25123243 22.94998175 0 33.33333333 43.16648917 28.15944636 0 4.63707855 24.35141909 0 0 33.33333333 0 50.97632781 0 20.8986622 0 0 29.04479822 14.38097745 15.86873455 0 0 15.92306167 0 0 23.71538306 0 33.33333333 14.78326708 0 33.33333333 0 0 10.20819035 33.33333333 4.188723637 32.55601906 10.67 28.66666667 19.01333333 26.10915614 27.69070538 0 0

733 4.94306066 0 0 8.514715948 0 14.77053289 33.33333333 0 34.04309716 20.0148258 11.42622277 9.159128053 16.02977407 0 0 22.08104052 42.84940692 22.36312001 30.70426445 74.84347277 20.03597769 66.66666667 0 0 0 0 0 0 0 20.57492985 0 0 41.70412817 0 0 0 23.26893145 42.45454014 37.89342189 44.57434053 0 0 26.91066439 4.042210696 13.06730904 8.548831423 13.50255865 43.7549946 10.9996541 18.01629743 33.33333333 0 0 23.67906067 0 0 0 19.1890742 16.76224085 0 0 11.97333333 9.013333333 21.70618034 0 33.33333333 37.97086798

734 0 0 0 0 14.71182085 29.32344764 9.115506269 11.48061105 18.17037626 0 0 21.5028677 49.94094826 20.52691867 86.84414703 22.170767 15.40181691 33.33333333 8.426359381 0 7.249277296 0 49.59945068 0 43.99137608 8.905977312 0 22.66606822 24.57747247 33.64740804 18.68216007 3.576739454 0 56.67482502 29.16945896 11.75390266 0 0 18.86792453 0 28.88724896 15.76508746 0 20.863711 45.03052083 0 33.33333333 0 33.33333333 7.953439153 0 18.19771138 5.100293542 0 39.81176517 0 44.61381333 0 0 8.015478165 20.69333333 16.71 7.163333333 21.97101449 28.72615999 21.33183411 0

735 2.903739061 0 0 0 6.096313166 0 10.458289 45.93362509 0 0 0 0 0 0 0 33.33333333 0 0 0 0 0 0 0 20.40690491 0 0 0 0 0 0 16.67980037 7.952962158 0 0 0 0 0 0 0 22.96598639 0 0 0 0 0 0 0 0 0 33.33333333 33.33333333 0 12.83537045 10.23672425 0 33.33333333 0 0 0 18.45999895 0 0 0 0 0 0 33.33333333

736 12.87908625 22.76567707 15.90619424 24.81861739 0 0 0 0 0 0 17.96520286 33.33333333 0 6.862541528 0 11.25229281 12.47817527 0 0 12.97491039 0 11.12825789 0 0 20.16682362 33.33333333 23.57404988 31.47386269 33.33333333 0 8.981914245 35.20566094 0 0 16.12055514 0 29.81654191 0 0 0 4.28853511 16.76636756 17.46459879 19.26906338 0 3.03030303 19.4511703 0 9.617950273 9.172486772 0 0 0 9.654272668 1.011975783 13.37619181 7.417067033 6.412771687 27.80985788 4.825258141 10.43666667 15.47333333 7.413333333 0 0 0 0

737 0 9.525221086 7.618588714 18.00852772 18.62151248 41.88646109 4.675888876 21.85272229 24.44598076 0 46.69817783 11.83046564 0 11.08881563 0 11.16256633 25.74858832 0 8.936327511 0 29.26108483 0 11.48438926 0 0 3.708551483 0 0 21.643324 20.93208074 0 0 4.912186859 9.991841642 19.91192064 37.26976953 21.44458028 5.349938607 7.209763127 0 0 0 16.33245803 12.16255869 0 24.21510149 0 38.07977163 8.855067451 3.823953824 0 15.13562195 10.32289628 0 0 0 4.910096819 0 0 4.683373257 4.08 0 8.066666667 12.78446088 26.20327544 0 8.737549224

738 0 0 0 0 0 0 0 0 0 0 0 0 0 0 0 0 0 0 0 0 0 0 0 10.38335159 0 0 0 0 0 0 0 0 0 0 0 0 0 12.43467114 0 0 0 0 0 0 13.803938 0 0 0 0 0 0 0 0 0 0 0 0 0 0 0 0 0 0 0 0 0 0

739 59.17483396 0 0 0 18.0743968 0 0 0 15.65110165 49.34717161 0 12.21600718 0 12.3719546 0 0 0 0 39.09216326 0 14.61715218 0 17.06721599 18.20183412 0 0 23.50017749 0 0 20.20850282 0 17.44540972 20.05035164 0 4.143056582 0 0 10.43676548 28.93810795 22.09232614 37.77941771 18.95235588 0 12.46962233 0 39.16447054 0 0 0 0 0 18.55006625 33.92020001 0 33.74942012 21.80951364 25.76828261 41.06482078 42.69622401 17.7260723 0 6.29 22.45333333 17.42918814 0 15.11121542 19.95824946

740 0 74.58616767 80.87309572 72.7371755 46.29539563 39.80837962 84.3867141 46.33269588 50.52761664 0 82.05335513 42.4477879 61.99323718 27.88269357 38.65360066 68.2597514 50.6981056 70.89417093 19.11535666 37.45674821 54.08487914 67.5599349 46.70326094 48.44605081 79.1693574 63.59510345 34.24524975 66.42285949 29.25326406 47.73354014 68.91808896 56.03425966 32.06488975 25.90370823 37.46896492 20.55047038 45.33349758 25.3300564 14.39969473 44.80184473 16.93964881 23.14584996 49.64426112 37.64654675 30.13605296 14.14163886 40.06096344 50.34720679 63.37688883 55.77981735 56.65655819 26.09281041 21.9665272 54.53126353 22.94885854 21.46859528 13.46008426 17.83506092 0 6.740649404 25.5 26.14666667 11.66 24.52316076 34.54015908 37.00903458 44.22231478

741 0 0 0 0 0 0 0 0 0 0 0 0 0 0 0 0 0 0 0 0 0 0 0 0 0 0 0 0 0 0 0 0 0 20.42398355 0 0 0 0 0 0 0 0 0 0 0 0 12.97771776 0 0 0 0 0 0 0 0 0 0 0 0 0 0 0 0 0 0 0 0

742 0 0 0 0 0 0 0 0 14.07487401 0 0 0 0 0 0 0 11.64616286 0 0 0 0 0 0 0 0 0 0 0 25.87906866 0 0 0 0 0 0 0 0 0 6.580946815 0 13.51142083 0 0 0 0 0 0 0 0 0 0 16.21258343 0 0 0 0 11.83861149 0 0 0 0 0 9.083333333 7.782982045 10.76957099 11.44913314 13.27224767

743 0 0 2.555246054 5.741303614 0 0 0 4.04942178 0 0 0 0 9.799834574 16.11991645 0 0 0 0 0 7.785312516 0 14.07802043 9.271523179 0 0 0 0 0 0 0 0 21.56978654 0 6.614583333 17.94926683 0 0 11.79589132 0 0 0 0 10.11922357 10.93454408 23.55584082 10.57100482 8.429692105 9.004018681 0 0 8.325347389 0 0 10.12631128 0 0 36.61733463 0 0 0 0 6.533333333 4.526666667 12.166236 0 0 0

744 21.32772078 5.262579653 0 0 3.788077158 23.95166349 0 13.68250994 0 0 4.048843188 26.94045096 13.07371818 8.293101599 13.08561495 9.2819454 0 7.996047091 0 0 37.90213571 0 4.634034065 0 0 12.35494234 2.676610731 21.59241088 0 9.152214216 14.64647154 0 16.68045216 10.99201345 5.064824912 28.23354815 13.62258935 27.19847203 28.28074992 0 0 18.35703998 7.844769493 8.17693829 0 33.33333333 0 0 0 12.69451269 0 0 9.322580645 0 0 26.03017959 0 17.47327134 14.77297896 12.47185298 21.4 0 0 0 0 23.9507204 0

745 0 0 4.036490877 6.196689255 0 8.218646341 0 0 0 0 0 8.695021574 0 0 35.86998974 4.982074422 8.180999311 9.385484968 10.77796434 0 0 2.532372598 6.737786024 0 7.090922719 0 10.20164373 0 33.33333333 0 0 0 0 0 19.56773447 20.48484848 19.65655697 0 26.75238652 0 19.8219125 16.33277164 0 4.097085365 7.505079954 0 8.855188042 12.68396109 12.19521387 10.04031888 16.88464118 24.37280829 15.65771438 10.31395217 10.38447479 10.93461086 0 9.933624295 25.89014831 26.59268393 16.84333333 20.3 0 0 35.15511253 0 15.77473802

746 0 10.97744473 0 0 0 0 0 6.354767017 9.411670471 66.66666667 0 0 0 0 0 0 15.15030593 0 0 0 0 0 24.06181015 6.474344001 0 0 0 0 0 8.785942492 0 11.7635468 23.23881584 12.90934979 0 0 0 0 0 12.40504699 33.33333333 0 0 0 9.26417704 0 0 10.11859056 0 0 0 0 0 0 33.33333333 0 0 0 0 0 0 3.723333333 0 0 19.5351574 0 9.172104216

747 33.33333333 9.173807954 2.87093779 3.188665175 49.91652721 5.287412079 7.155447392 5.433623764 25.98583888 0 0 9.409409409 4.327293755 0 0 0 7.088459285 9.792042623 9.441733292 35.39738939 2.09322983 9.633458647 0 36.36668764 5.37452986 0 19.54316246 7.13712911 0 9.780912313 9.328897142 8.808269943 0 0 2.176986146 0 0 23.95417958 0 0 0 31.20334393 14.22163551 6.909633935 9.777492512 41.95402299 20.35561558 0 16.65147372 0 12.04017741 11.78781204 21.99047251 16.31712935 0 0 16.58924778 0 23.56919875 54.19481368 0 0 7.893333333 46.71744862 0 0 17.55859531

748 45.33894589 0 9.664229559 9.722222222 0 16.1526295 8.457838505 24.14698163 0 0 13.89780169 0 5.999953834 47.70428838 12.39079465 0 0 0 22.55536899 0 5.919755322 6.196213425 8.591585643 8.712917543 8.365190016 0 33.33333333 4.847600519 11.53433395 0 7.106542359 1.824137062 0 23.15636165 13.76559887 17.88264814 10.54039593 0 23.98622201 33.33333333 16.39368452 0 18.17011031 22.39878925 12.52760845 0 9.32082308 7.010969914 7.77642359 17.91623171 6.093275835 7.076550518 31.06270527 0 33.33333333 34.90137945 0 33.33333333 18.56035437 0 0 10.94 18.28 8.81017257 0 22.26016173 0

749 0 0 0 2.413944237 0 6.581268967 0 0 0 33.33333333 0 12.50733015 4.805962478 0 0 17.47622878 7.235967013 1.932254383 38.10957671 19.36054989 0 0 0 0 0 24.04995421 0 0 0 24.54739084 0 0 28.01584225 0 4.006623854 12.84848485 10.84696017 11.72140068 0 9.459774945 0 10.96099449 0 9.836462331 7.233748271 0 0 10.83525296 0 3.569119359 0 14.45743532 0 8.711343668 0 6.665234822 21.49472184 21.42471012 17.20731961 0 9.213333333 0 0 0 0 5.330950152 0

750 0 0 6.79876161 0 1.309963942 0 6.771947691 2.860238353 5.475482346 0 4.63070379 3.58883846 0 0 0 14.41860465 0 2.632363395 0 0 0 0 0 0 0 0 0 3.75089222 0 0 0 0 4.533376715 0 6.243982623 8.191229371 0 2.800533435 0 0 0 0 3.619828816 0 0 0 0 4.088573703 0 0 0 0 3.925857379 0 0 0 6.963537898 0 3.556810162 7.028003613 0 0 2.793333333 0 6.517950505 0 2.784110535

751 4.392711859 0.863810519 0 0 0 0 0 5.096794824 0 5.093903229 0 7.539826432 2.677155045 0 2.478809032 0 1.249480295 7.389705882 0 0 3.781094527 3.732779688 29.22734152 0 0 0 5.393081761 0 0 0 2.754023298 2.27184464 0 6.309408607 0 0 0 1.559811645 0 0 4.266047703 0 0 0 0 4.911939986 1.833118754 4.873472642 0 0 1.124194718 0 0 0 0 1.492836389 0 0 0 1.554480129 0 6.31 0 3.448740401 0 0 0

752 0 3.21219987 0 0 0 4.949494949 0 0 0 5.718022975 2.69823888 6.168304458 0 1.625262 0 0 0 1.09670987 0 12.39051094 0 3.36058129 2.294603792 5.935781019 2.048054348 2.602812491 0.800475959 5.064239829 4.661889252 3.632947395 2.103863128 6.36930534 0 3.393672381 0 3.389182159 0 0 1.231837669 1.762983707 0 3.27675788 0 0 0 0 0 0 4.091487206 0 0 0 0 2.574860151 0 0 0 0 0 0 4.893333333 0 0 0 0 9.001332492 0

753 0 0 6.671345648 3.447201744 1.406926407 33.33333333 0 1.17972794 12.16068605 0 0 0 1.809214164 0 4.547226192 21.18328363 0 0 2.951858945 4.514622974 1.792991035 0 0 0 0 0 0 5.806014324 0 0 0 0 0 0 2.642756017 0 5.854054367 3.75879072 0 0 3.654682363 0 0 0 3.184763711 0 6.235056097 0 1.796215429 0 2.660177345 2.130364043 0 1.432019682 2.870438403 1.95461694 5.873255174 3.271055235 0 3.11384856 0 0 12.94666667 0.929481127 1.315895049 0 3.355386325

754 1.242827607 0 0 0 0 0 0 0 0 0 0 1.271544951 0 2.665213303 0 0 0 1.387264681 0 0 0 0 0 0 1.231209236 0 4.785679493 0 0 0 0 1.557410423 0 0 0 0 0 0 0 19.73402992 0 1.748364045 0 0 0 0 0 4.303763128 1.665728522 0 0 0 0 0 3.267071784 4.598929119 0 0 2.896238719 0 0 0 0 0 0 0 0

755 0 0 0 0 0 0 0 0 0 0 0 0 0 0 0 0 0 0 0 0 0 0 0 0 0 8.987038286 0 0 4.994922981 0 0 0 0 0 5.075357771 0 0 8.005071086 0 0 0 0 0 0 0 0 0 0 0 0 0 0 0 0 0 0 0 0 0 0 0 0 0 0 3.939907518 0 0

756 28.46461853 0 0 15.85967181 0 17.96555066 0 10.1793668 0 0 0 0 0 0 0 0 0 0 0 0 0 0 0 0 0 0 0 0 0 13.87014916 0 0 9.354156499 7.920042557 0 0 0 10.48877035 0 0 0 0 22.82705252 0 0 0 0 0 0 0 12.48560092 0 13.5208161 0 0 12.89950907 0 12.26354694 0 0 37.49 0 0 0 0 33.33333333 32.36629518

757 28.94062147 95.92398961 54.87695141 29.88613159 32.02336939 28.38383838 93.22805231 33.33333333 49.76490227 0 54.25106654 7.99293999 31.80186648 13.81502537 28.78610714 0 0 0 0 0 61.0925811 33.33333333 28.20207273 28.66033973 32.1021241 0 27.94025157 54.20595672 0 0 0 69.91035518 33.33333333 0 0 4.433478693 33.33333333 0 0 33.33333333 0 0 0 0 0 0 0 0 18.04888655 0 16.31194367 33.33333333 0 31.90131365 61.16132482 10.5068839 0 0 0 13.9712155 24.70333333 0 0 0 0 24.33200084 61.49420796

758 0 0 0 33.33333333 65.25974026 0 0 0 0 0 0 0 0 31.70807133 30.8545243 0 98.75051971 0 0 0 33.33333333 0 40.27598196 65.40387925 0 0 32.53285737 31.17289691 31.30078571 63.03371927 63.91264337 0 0 0 0 0 0 0 0 0 0 31.58496929 0 100 33.33333333 30.39406866 58.59849182 0 31.5371179 0 30.67315599 64.53630262 0 64.09180652 32.701165 0 33.33333333 0 60.21361779 54.97033436 0 0 0 0 0 33.33333333 0

759 36.95922053 0 31.65294133 17.47366152 0 15.36778268 0 47.35053875 32.59892933 89.1880738 38.41999079 73.43854571 63.71176432 50.186428 33.33333333 64.39811172 0 87.49395617 97.04814106 83.09486609 0 59.57330569 0 0 64.61861232 88.41014922 28.54765384 0 59.04240206 19.46318417 31.22947021 19.89108442 52.77913345 82.37687645 86.03790359 83.98610978 60.8126123 73.38702277 98.76816233 45.16965304 92.07926993 63.38990879 73.55311866 0 63.48190296 64.69399136 33.33333333 86.73419053 42.86056439 100 36.74492736 0 82.55332652 0 0 68.54722458 53.82987359 84.46539782 33.33333333 19.36211783 52.12333333 114.5266667 131.5066667 95.62177847 88.22624693 0 0

760 28.9348918 78.90521763 47.88306216 21.79306014 33.33333333 30.09581442 60.28176834 26.4974696 50.789914 0 0 0 24.83481134 0 17.28666209 20.37897512 0 0 0 0 39.87462218 25.15428656 42.69123191 29.24178155 33.33333333 0 19.28104575 55.65534296 0 0 0 30.65103473 24.858184 0 0 0 30.76206447 0 0 25.36140528 0 0 0 0 0 0 0 0 0 0 0 0 0 0 24.45922189 0 0 0 0 0 14.84 0 0 0 0 28.82653061 84.59550897

761 0 0 0 0 0 0 0 0 0 0 0 0 0 0 0 0 0 0 0 0 0 0 0 0 0 0 0 0 0 0 0 0 0 0 0 0 0 0 0 33.33333333 0 0 0 0 0 0 12.17194015 0 0 0 0 0 0 0 0 0 0 0 0 17.43392263 0 0 0 0 0 0 0

762 0 0 0 0 0 0 0 0 0 0 0 0 0 0 0 0 0 0 0 0 0 0 0 0 0 0 0 0 0 0 0 0 0 0 0 0 0 0 0 0 0 0 0 0 0 0 0 0 0 0 0 0 0 0 0 0 0 0 0 0 0 0 0 0 0 0 0

763 0 0 0 6.918930235 0 11.20263591 11.7488882 0 0 0 28.64480153 0 0 0 0 0 0 0 0 0 7.616221563 0 0 0 0 0 0 33.33333333 18.6683523 0 0 0 0 0 0 15.18847007 0 0 0 0 13.04807184 0 0 0 0 0 0 0 0 25.20550476 0 0 24.8694517 41.88284247 0 0 0 0 0 0 3.493333333 0 0 0 0 0 0

764 11.96741855 0 0 33.33333333 0 0 0 0 0 33.33333333 0 13.03017053 0 22.94759431 33.33333333 0 0 17.53914989 0 0 0 30 33.33333333 0 0 20.77743378 0 11.0113237 20.40505558 0 0 0 0 0 0 0 0 0 0 0 0 0 0 0 24.91558128 0 5.095658572 33.33333333 0 0 0 33.33333333 0 0 0 7.43618202 0 15.97385621 0 20.26189916 0 0 0 0 0 4.783184376 0

765 4.398441534 5.084401058 0 0 0 33.33333333 6.384898327 70.35274568 7.172326457 33.33333333 0 33.33333333 0 0 0 33.33333333 49.4100924 0 33.33333333 0 0 33.33333333 0 37.42488512 0 33.33333333 21.73447537 0 14.66498104 0 0 0 0 0 33.33333333 0 0 0 0 7.971928055 0 0 19.74687405 0 33.33333333 33.33333333 0 0 0 66.66666667 44.35934259 18.31641286 0 0 0 49.35153584 33.33333333 9.700176367 0 15.8994107 4.67 1.976666667 0 33.33333333 33.33333333 4.506802721 0

766 54.69924812 16.01038131 52.11693784 4.621342963 0 3.237518911 0 0 33.33333333 0 0 0 0 0 16.46881477 0 33.33333333 15.79418345 0 0 0 0 5.235315578 0 66.66666667 0 0 0 16.52859961 33.33333333 64.01727564 33.33333333 18.39301381 66.66666667 0 33.33333333 35.9046022 33.33333333 14.52587339 33.33333333 0 33.33333333 0 0 33.33333333 33.33333333 15.86141562 49.68297101 0 0 33.33333333 33.33333333 0 0 31.24809518 0 0 0 31.92325824 33.33333333 6.936666667 6.31 9.47 33.33333333 0 33.33333333 11.37796173

767 0 0 0 0 42.13325597 0 21.58444513 3.149784727 0 0 0 7.28756741 41.83185533 0 0 0 0 33.33333333 0 33.33333333 0 3.293925199 0 0 0 33.33333333 3.933900604 0 12.92827775 33.33333333 0 36.01563194 0 0 33.33333333 18.14486327 0 66.66666667 66.66666667 0 66.66666667 0 0 33.33333333 0 0 0 0 66.66666667 0 0 0 0 0 33.33333333 0 10.2 0 33.33333333 0 0 0 0 0 33.33333333 0 4.026529307

768 0 0 0 0 0 22.13069742 0 0 0 0 71.35519847 0 0 43.71907236 16.04667124 0 0 0 0 0 0 3.333333333 0 0 0 12.55589955 11.59885796 0 0 0 0 0 0 0 0 0 33.33333333 0 0 0 0 0 66.66666667 0 8.41775205 0 17.47191772 0 33.33333333 0 0 15.01692047 0 0 0 17.31513083 33.33333333 17.35947712 34.74340843 0 0 11.89 0 0 0 0 0

769 0 0 0 0 24.5334107 0 0 0 8.704426207 0 0 46.34892873 33.33333333 0 16.86451856 12.95435821 17.25657427 33.33333333 0 66.66666667 19.17582293 4.88512157 18.74011918 0 0 0 10.11838698 0 16.80473373 0 35.98272436 0 56.74880219 0 0 0 0 0 18.80745994 0 20.28526149 33.33333333 13.58645929 66.66666667 0 0 16.06573461 16.98369565 0 8.127828577 22.30732408 0 8.463881636 58.11715753 10.95934959 25.89715131 23.13333333 56.9664903 0 13.07143417 0 9.426666667 17.58 33.33333333 0 28.55014896 0

770 4.308246004 0 20.77066149 0 0 33.33333333 40.27401184 6.047437987 21.66433032 0 6.67386498 0 0 16.91313955 25.7606377 11.49369347 5.566357025 6.308310887 34.22616157 0 0 0 0 6.861631649 0 23.45106073 0 7.12962963 22.49951873 6.868651075 0 2.524554942 6.117164743 15.72123233 0 2.567584249 19.30010063 8.835607365 0 21.42640594 7.463694698 16.81937173 8.667850799 0 4.227792538 11.92937623 0 0 15.87753909 27.75801539 9.381851616 15.03554202 0 0 9.424385139 0 12.48552585 11.37234105 0 11.72851375 3.55 0 0 0 7.51534777 0 16.23228166

771 9.486183653 55.25047106 24.82140418 41.69106336 31.44582668 4.472730487 0 10.57497833 14.88123329 37.0090242 0 11.28605564 22.79266712 3.226134228 13.13773766 10.83271664 0 0 0 0 0 0 36.71282835 15.93754299 0 0 22.13341228 15.55911681 0 30.98019673 8.822778811 44.99268935 9.367681499 0 13.16337417 5.271483976 15.44638341 0 13.38919174 33.38802349 0 0 0 0 7.002695756 5.161389723 9.276318805 15.8400843 3.940455342 0 15.08001829 17.4123926 12.61008057 16.02102306 21.72890745 14.90160539 5.749340181 0 0 24.08100347 32.87333333 0 0 0 7.827691434 12.44747537 0

772 0 0 0 0 0 0 0 0 0 0 13.94550067 24.89481623 6.945258088 0 11.22256672 0 14.1330946 0 0 0 23.84531732 0 0 20.37436519 48.61917572 12.15023801 37.45250507 23.05411694 23.63396972 0 22.20716281 0 0 15.73836712 20.18119969 0 0 1.880284824 0 0 0 0 24.01201176 14.57085828 4.882368796 0 20.97803503 0 12.25432704 7.730349832 0 0 0 0 0 7.311704469 0 6.732528357 0 0 0 0 14.83 0 19.4409256 15.55319065 17.91918084

773 18.62690137 0 0 0 0 10.16201185 18.04255909 0 0 0 0 0 0 32.86510326 0 0 11.78080219 0 14.55024664 10.79770564 0 8.493213711 22.02927478 0 0 32.80345843 0 19.13703704 5.883204808 5.881013379 0 14.99002455 0 14.21811379 0 18.05012313 14.49477601 0 25.48167947 0 27.56137147 22.51304422 0 0 12.48197605 5.19220399 0 15.24552384 0 0 15.06473361 8.695931203 54.65335851 0 0 0 17.86117381 19.19943742 0 0 13.73 46.13666667 11.82666667 28.4420824 0 7.12268375 0

774 0 5.843621399 7.613617985 7.814561014 0 7.672589057 0 7.618222094 12.86317058 10.70274637 0 0 27.31968272 0 0 12.46044304 19.00431916 0 0 33.33333333 0 19.07549841 0 8.94771946 0 0 0 5.316951567 0 10.74887786 10.69670786 0 27.21616859 0 7.381356617 43.77184401 5.943265514 19.76352674 0 0 0 16.51396161 0 5.886214442 16.21293735 33.33333333 33.33333333 0 0 0 10.62309923 9.463061691 4.821685091 2.990349327 17.7603349 30.25060916 27.58399315 0 44.9810989 6.906555815 6.103333333 0 0 20.86593636 16.98036835 0 45.15967902

775 29.02508733 0 4.043236214 21.22646471 39.75535168 0 0 27.28589535 8.533899638 19.48076032 31.69837669 43.71053081 0 0 0 0 8.122948374 18.92934425 0 0 14.68686869 35.74694805 0 17.39579034 0 0 0 0 18.4765502 0 0 0 33.33333333 24.77618155 23.11280014 0 17.88694992 19.49821985 33.33333333 23.44103601 12.31870409 0 0 22.21866154 0 0 24.05701453 0 17.45579424 34.28541914 8.614540466 18.35965207 0 33.33333333 0 0 20.84780748 0 24.16706905 23.85352498 0 0 0 18.16928591 0 0 0

776 23.84714968 0 16.38709677 0 0 23.17132148 15.29077424 33.33333333 0 0 26.38871016 0 0 0 0 21.83963986 0 27.02502245 51.22359179 33.33333333 42.82134935 0 20.40563324 16.60988075 16.80318139 0 17.84372473 12.45726496 0 0 33.33333333 14.45546025 23.96565183 17.59496621 0 0 0 0 19.9441416 0 0 0 24.66548253 0 24.223172 44.38369672 0 68.91439185 29.39287799 0 18.26859973 0 0 33.33333333 20.76316493 0 0 39.01610166 0 0 0 0 0 0 25.5056419 17.78014268 5.274705986

777 0 13.36991312 0 29.26791091 0 0 16.93077565 0 42.05736617 22.00079318 0 8.438517107 16.55431683 18.65569273 20.19559568 43.37350699 41.39247865 14.40398909 0 0 18.64646465 14.25783492 20.85226362 13.87306962 34.57764289 10.41214751 11.19992106 0 19.80739293 29.80545656 0 7.218517066 0 11.95113899 36.16126939 15.05575443 15.89732371 38.06753256 0 0 25.86963864 44.15362244 26.34983977 46.20959394 0 0 12.35529831 0 0 30.22621563 13.32838249 0 12.01330816 0 30.32320758 26.02162886 15.47215952 23.67959151 0 21.60481959 6.44 6.62 3.676666667 0 0 47.09650754 15.4141525

778 14.70643196 25.53599442 26.36398335 0 28.79882163 21.18801379 9.461879182 15.14013291 0 10.80667594 21.2935475 11.67008022 26.38807525 28.33993023 22.11076661 0 0 33.33333333 0 22.5356277 0 15.53439541 0 0 0 21.18309532 0 17.34588306 9.699363616 15.71580439 24.94001719 15.81875384 0 0 0 15.2832102 11.0312008 11.95482866 7.851653859 21.74453456 26.7865911 0 16.30481513 11.11467179 30.96905751 0 0 0 21.07900629 0 9.638774577 31.03342042 15.90156767 14.32196094 0 21.51445212 0 0 30.85183205 11.8255824 5.18 22.31 17.24333333 32.52269533 22.73002495 0 0

779 0 0 0 0 0 0 0 0 0 0 0 0 0 0 7.572695633 0 0 0 0 0 0 6.892109501 0 0 0 0 11.37043686 0 0 0 0 0 0 0 0 0 0 0 0 0 0 0 0 0 0 0 0 0 0 0 0 0 0 0 0 0 0 0 0 0 0 0 0 0 0 0 0

780 0 0 0 0 0 0 0 0 0 16.61106834 0 0 0 0 16.05816343 0 0 0 0 0 0 0 0 0 0 0 0 0 0 0 0 13.29963585 0 0 0 0 0 0 0 0 0 11.17564238 0 0 0 0 0 0 0 0 0 0 0 0 0 0 14.29236499 8.211478505 0 0 0 0 0 0 10.67069935 0 0

781 84.63768171 81.53581775 74.73413674 75.40763324 56.08526994 48.00437484 83.17930748 52.66721251 44.46516702 24.24999133 93.85108407 25.09539843 58.95337908 50.08021849 48.27276596 64.55379916 53.56697943 66.66666667 75.37833066 62.39185334 70.92058192 36.1839488 69.17777897 69.48774472 82.23801535 68.48713079 74.56701656 64.59503411 72.29016149 81.21370929 56.95229476 47.62377011 33.85405117 50.97153855 47.82185592 38.05736122 68.41464587 56.15190487 36.44289624 67.10152778 57.76377564 41.2941921 65.758235 58.96385328 55.21863895 51.89221963 79.08040432 45.7716479 81.55228966 57.74809631 49.25792995 50.98653509 91.7450117 48.43414915 68.52998021 55.9400656 25.27983539 58.46789696 57.5318512 48.80915921 6.096666667 26.05333333 42.51333333 28.82795792 18.16944539 51.32013201 52.45242935

782 0 0 0 0 0 15.06508206 0 0 0 26.12899798 0 28.44505106 0 0 0 0 0 17.66960775 0 0 0 25.06423365 0 0 0 0 0 0 0 0 0 0 22.38304564 0 23.92392392 0 0 0 21.31561238 0 0 0 0 0 0 0 0 0 0 0 0 0 0 0 0 0 0 0 0 25.42802026 13.84 7.44 0 24.05342145 0 22.45494398 0

783 0 0 0 0 0 0 0 0 0 0 0 33.33333333 0 12.26216846 9.414677836 0 0 0 0 18.28272935 0 0 0 0 0 0 0 0 0 0 0 10.42991467 0 13.86259204 0 0 0 0 0 0 0 23.29450733 0 0 0 33.33333333 0 0 0 0 0 0 0 13.38591126 0 0 41.07149256 11.65415185 0 0 16.92 0 0 13.50149802 0 0 0

784 0 7.019332908 0 0 0 0 0 18.64358481 5.473351001 0 0 0 0 0 0 0 8.816899932 0 0 3.239545689 0 0 5.690187693 0 14.61187215 0 19.3922082 11.78511606 10.24978467 0 0 0 0 0 0 23.34275485 6.377644055 0 2.719936798 0 0 0 0 8.075390745 0 0 3.309790888 4.824497347 0 12.33622847 10.8964267 0 0 4.106311157 0 11.19934385 0 0 4.774638608 0 0 7.613333333 0 0 41.21910317 0 11.70524482

785 7.686942659 0 6.049600117 9.6316783 30.03434719 0 6.96660163 0 14.15912781 16.72226499 0 0 0 8.931585097 10.1701887 7.264652233 11.66944908 0 0 0 0 0 0 6.604747162 0 0 0 0 6.735972657 0 0 0 0 11.21699053 0 0 0 0 9.452180391 0 14.46608947 0 0 0 0 0 0 23.81062534 0 9.995613211 0 8.092190688 0 0 0 13.08493094 0 0 0 11.89181021 0 0 0 0 0 0 0

786 7.675375634 5.670470756 11.13759767 10.92188078 0 18.25219257 9.85409089 0 0 16.28767736 6.148915928 8.237934905 22.26647104 0 0 22.4515145 0 0 7.55260477 0 5.862286797 15.68312285 11.58962421 9.495622148 3.150112504 14.35871585 0 0 3.812397148 4.629539952 0 8.767998672 10.9502877 0 13.91366077 28.60930545 0 0 0 0 0 11.09474536 9.794942248 0 18.36315032 7.726969292 6.300192286 11.79643277 0 0 3.156662084 20.2606773 0 4.549568365 9.902322045 5.737506065 8.053497942 0 9.168228341 5.965697241 0 0 0 0 0 15.34653465 0

787 0 0 0 0 8.608058608 10.10638298 0 28.68920268 17.35143504 0 0 4.888282277 5.11068598 22.70672811 13.18431205 0 12.79706197 0 2.535947712 0 16.32725974 8.26909968 0 9.346526489 0 7.10968775 6.040775233 8.344900105 0 0 13.04183943 0 9.556646408 14.10001656 14.34055939 0 25.20771008 34.53754491 22.31623684 20.49751405 27.7701349 0 24.44682275 16.42992405 26.41821074 7.047477745 4.066842081 13.79679665 18.44771034 9.710976986 0 15.31372251 8.254988298 0 16.77156248 0 11.30280911 2.061707036 0 0 39.87 15.74333333 19.69 16.29643841 23.07900773 10.87838935 35.84232583

788 0 5.774378585 0 0 5.272324256 0 0 0 4.755277198 0 0 0 13.6694639 6.01929984 0 0 0 0 0 0 0 0 0 0 0 0 0 10.21393035 0 0 15.6339762 9.603782814 14.29929152 0 0 0 0 0 7.753137356 0 0 0 0 0 0 0 0 0 0 10.20908502 14.58451102 0 0 0 0 0 0 0 0 7.905313078 0 0 0 17.3206842 0 0 0

789 0 0 8.078665477 4.038807674 0 8.571967553 0 0 13.79564193 0 0 0 0 0 2.899892025 5.730034103 13.14960958 15.66372559 14.53311686 16.08587162 6.889871552 14.79959502 13.54240912 5.065359477 0 10.04446562 0 5.061019383 6.911684037 14.15675076 14.37188961 10.27489789 8.956677575 9.848862315 0 9.990578481 0 9.31055022 0 12.40095817 0 13.14091282 0 16.53083192 0 0 7.242770426 0 0 0 22.10447025 5.34687442 0 29.52406007 4.796135266 14.03815355 0 19.60476565 28.52528185 0 0 14.6 0 0 6.861744357 0 0

790 12.42474001 7.993382523 0 15.54035279 5.775454319 4.735755186 15.05783385 0 8.44765343 13.62354743 14.46119532 20.204853 5.656934307 0 6.390128353 4.23171568 4.820383335 15.9491412 11.57119853 23.97950109 0 0 22.96281013 0 8.607330684 25.57400486 10.53851998 1.696949855 19.54173803 11.50565709 0 0 0 5.259562842 8.034894399 0 0 22.86089239 0 7.501419648 9.237079573 8.740865655 4.922015832 12.77593601 4.62006079 17.78143052 32.87443597 2.218209956 0 0 0 0 13.78197572 0 0 9.158702577 20.02546989 8.455372748 10.72545694 17.4728632 0 14 21.89 0 8.316137696 7.947715258 0

791 10.46045384 0 10.55060471 0 2.382604673 7.390080139 0 0 12.46615363 0 9.949680748 14.27034934 0 1.341248901 0 0 0 9.671238403 0 0 4.089219331 3.512195122 0 0 0 0 0 0 0 0 0 1.853281853 0 0 14.44653601 0 14.93963115 0 9.188102043 0 8.945433275 0 0 0 0 0 0 0 2.548497528 25.13994436 0 0 0 0 0 0 0 0 0 5.671837807 1.36 0 0 34.78567958 0 0 6.999778663

792 0 0 0 0 0 0 0 0 0 0 0 0 0 4.144638742 0 0 0 0 3.830398243 0 0 24.16110559 0 0 2.229389464 0 1.857527631 0 0 0 0 0 8.113855717 0 19.20345003 0 0 0 0 0 0 0 0 0 0 0 0 0 0 0 0 0 0 0 0 0 0 0 0 0 2.946666667 0 0 0 19.76376643 6.047595682 0

793 9.39088279 0 0 10.6112215 7.336726039 0 0 41.55785081 0 25.80070084 6.286724856 8.633950818 0 10.32541777 10.89580146 11.67847938 0 0 0 0 0 9.224117223 0 14.8856305 0 0 0 27.15364414 0 0 10.94115355 0 0 8.00204918 0 19.91617432 17.79833595 0 9.785730064 17.06325402 29.89040864 14.85969164 8.883925072 7.159827214 24.91894398 6.048978671 0 23.29845253 0 0 18.78420665 10.88878726 0 0 10.39639901 22.68447396 7.580105213 0 0 0 0 0 6.456666667 6.871794872 17.56665704 10.70908713 23.76926114

794 19.70378301 38.52083127 39.79612002 17.10240494 15.7664719 8.504646578 30.59142559 0 0 14.52171332 5.590088376 19.062984 18.83305951 15.07625272 10.41701096 12.99366655 31.47982763 0 0 2.479252022 24.90640098 0 7.29628223 27.94624634 0 5.190351642 22.81667166 15.32165657 33.8936304 32.75265984 0 25.72473191 11.8180008 16.91589776 16.02034329 11.46295123 0 19.60183285 15.35556954 12.96992481 9.329823682 0 51.2987988 8.608285785 0 9.431919501 0 17.83447341 6.318494049 0 11.51269166 13.21593153 0 31.86084074 20.90997368 6.611243072 14.73845208 5.827758693 15.47441629 24.63138671 11.69 16.57666667 0 0 0 0 9.146746348

795 10.45164133 0 0 0 0 9.90686593 0 0 20.86717971 0 0 0 0 0 0 0 0 0 14.15408862 10.61094577 0 0 0 0 0 0 0 0 0 0 18.21019771 0 0 0 0 0 8.766006985 0 0 10.80636002 0 0 0 0 0 0 0 0 0 0 0 0 10.26166098 0 0 0 0 0 12.63957377 7.932103048 10.29 0 8.643333333 0 0 0 0

796 11.51771053 0 16.71561429 0 27.55787901 46.03398104 0 17.79996402 24.8856799 0 9.142035604 0 53.43950453 39.92374728 38.96372589 47.35824742 0 33.33333333 51.2650699 40.08815777 33.33333333 40.91680419 52.68103275 0 0 33.33333333 28.13225597 36.88506911 13.48739687 48.02458744 33.33333333 54.88394591 58.55281095 45.05464481 18.88679733 43.76808933 0 33.33333333 0 0 13.53021602 42.40859762 0 21.93664507 37.1276619 21.92754768 0 0 27.01483928 33.33333333 15.24029543 25.75968222 0 13.09960159 46.13430676 22.94613711 14.19177427 19.05020189 34.97216369 15.23434294 10.6 17.16666667 0 14.06177247 13.98578494 38.86840966 17.18680832

797 26.05078848 53.4857862 32.93766098 56.74602078 41.18086405 16.03638726 54.35074057 31.68742216 33.33333333 37.63017506 45.91874649 37.82786285 22.07050165 29.18869459 33.33333333 16.10795111 41.79543582 17.38419213 19.17924471 0 37.67104636 9.172227744 8.00523273 57.16812316 89.16327985 11.82795699 33.31147503 18.94268032 6.056353233 0 37.51531541 17.53804033 21.51533254 16.41743557 23.40797895 0 39.5709192 8.874494824 65.67059835 51.6590415 15.05807638 24.59246768 30.14300804 49.51930592 33.33333333 37.31426323 67.12556403 56.64886411 64.11816914 29.93906241 42.45678575 42.56194788 75.9563633 44.00104505 22.55932055 38.59944329 21.25071963 66.66666667 20.69375956 29.0574663 28.63666667 9.72 8.07 44.28075308 29.3362432 22.62424621 42.89740552

798 0 0 0 0 0 0 0 0 0 0 0 0 0 0 0 0 21.90435322 23.66209493 0 0 0 0 0 0 0 24.07435318 0 0 0 0 0 0 0 0 0 24.85278512 18.92510671 15.3294466 0 0 14.00896243 0 0 0 0 0 0 0 0 0 0 0 0 0 0 0 10.65202507 0 0 0 0 0 6.613333333 0 0 0 0

799 0 0 0 0 0 7.392283864 0 8.954763008 0 8.423863345 8.651528606 0 0 0 0 7.629939863 0 0 0 22.84214336 0 13.01355014 9.054642166 0 0 0 3.343549735 0 27.02088147 7.717095627 0 0 0 8.350409836 0 0 0 0 0 0 0 9.39837741 4.752252252 0 0 7.4958604 0 0 0 11.58765989 12.00602051 7.573651109 0 11.03851262 0 0 11.56145385 0 5.494629734 0 6.013333333 5.513333333 8.916666667 0 11.0314107 13.80294607 0

800 4.836451572 6.72332389 0 0 9.768064228 6.587955561 0 0 7.080283877 0 4.888963473 0 0 0 0 0 0 0 5.947105578 0 0 0 4.310964219 14.83385014 0 0 7.632048035 4.132824764 0 0 0 0 2.941600577 0 10.83510764 0 0 0 0 2.23970283 0 0 0 8.386187456 9.885365491 0 1.900654847 7.976729153 0 0 6.670174208 0 0 5.941284636 5.204736063 6.941313966 0 0 4.459142309 0 8.5 0 6.923333333 16.8895196 4.162923031 0 0

801 0 0 0 0 0 0 0 0 0 0 0 0 0 0 0 0 0 0 0 0 0 0 0 0 0 0 0 0 0 0 0 0 0 0 0 0 0 0 0 0 0 0 0 0 0 0 0 0 0 0 0 0 0 0 0 0 0 0 0 0 0 0 0 15.91869191 0 0 0

802 0 0 30.63092278 0 0 0 0 33.28759583 13.75929148 0 15.8168896 0 15.28179423 0 0 0 0 0 0 13.50606395 0 19.65841489 0 21.65566887 36.2998721 0 0 12.6330565 17.92177466 0 33.33333333 0 25.76667312 0 0 22.91438581 0 33.33333333 23.10699199 0 0 15.13090785 33.33333333 0 41.46545391 0 12.45274983 14.4046943 0 0 0 0 0 14.52424321 14.00214362 0 39.41440465 29.12558581 0 0 0 6.643333333 0 0 8.998777554 44.21185641 20.61827215

803 79.38935642 87.38603897 63.46980744 86.71606245 79.99745498 39.83783968 92.87037037 15.3893364 43.493689 100 61.77770319 85.0906833 9.769220796 51.73381439 92.10458527 61.8200701 98.9668616 83.40524686 39.14909172 26.05794717 100 51.38244393 57.329944 51.83281652 33.33333333 78.5740284 72.25103931 14.75511469 50.7332667 100 52.87612111 100 41.38877084 29.54048646 11.62763189 28.68763902 69.22490996 0 16.6711554 91.48867875 89.30090697 48.74185554 55.73002252 60.25359799 16.05875639 93.35569063 66.66666667 21.52891349 66.66666667 54.99296897 84.54343505 66.66666667 89.4583267 54.88010189 48.57278861 45.96376993 22.55852679 0 95.54085769 79.73349632 20.97 38.41333333 18.74333333 25.96373536 13.37868481 33.33333333 66.66666667

804 0 0 0 0 0 10.14225393 0 0 0 0 0 0 29.81958001 33.33333333 0 33.33333333 0 0 33.33333333 27.86873474 0 0 30.9186906 0 0 0 0 33.33333333 0 0 0 0 0 33.33333333 61.79071904 33.33333333 0 40.31461931 18.48288826 0 0 0 0 0 0 0 0 25.35660418 0 0 0 11.58279963 0 0 0 26.99208737 0 33.33333333 0 0 37.91666667 0 42.56 41.22805313 0 0 0

805 0 0 0 0 0 0 0 12.76666667 19.57404186 0 17.51644374 0 0 0 0 0 0 0 0 12.69753767 0 13.67491844 0 11.67766447 30.36679457 0 0 18.42320739 15.41155867 0 0 0 0 15.81046696 10.8705938 0 0 0 10.22634134 0 0 0 0 0 18.39622642 0 0 18.92863903 33.33333333 0 0 0 0 18.80909012 0 0 19.35119249 28.09961526 0 0 0 0 0 0 57.66788911 12.82657658 10.86116478

806 0 0 0 0 10.23448079 18.88429316 0 25.54144138 0 0 0 10.40731238 41.61565164 14.93285228 0 0 0 0 0 0 0 0 0 0 0 12.79447031 12.42503259 9.712750568 0 0 0 0 10.87270477 11.86656945 0 4.645694312 0 0 0 6.271618423 10.69909303 36.12723661 10.93664415 6.843966745 0 0 18.97992866 0 0 8.804649987 0 0 0 0 19.33118971 13.76158277 10.77480654 0 0 0 0 8.856666667 0 0 4.38540196 0 0

807 0 0 3.196859226 0 0 0 0 0 4.790419162 0 0 4.502004317 3.513753327 0 7.895414735 4.846596564 1.033138402 16.59475314 12.69606809 10.65317564 0 0 2.414642731 0 0 8.631501291 0 0 7.299558081 0 0 0 7.56666021 0 4.875947622 0 29.44058093 14.83525673 0 0 0 0 0 6.115417743 7.389211454 0 0 11.80441984 0 3.458297007 0 21.7505337 0 5.845280144 12.88914199 0 7.901069519 9.441465594 0 10.80704422 0 0 6.92 0 0 9.628233682 0

808 15.77419201 5.890637141 2.70241055 0 0 0 7.12962963 0 0 0 0 0 0 0 0 0 0 0 0 0 0 0 5.025758443 0 0 0 7.691880062 0 8.633841886 0 0 0 0 9.4491438 0 0 1.334509112 0 14.85044507 0 0 0 0 15.13961101 6.804986339 0 0 0 0 0 0 0 0 0 0 6.34124596 0 0 0 9.459459459 0 0 0 0 0 0 1.853896403

809 0 0 0 13.28393755 0 24.54765767 0 13.01495972 11.30227463 0 0 0 0 0 0 0 0 0 8.874401277 9.216540828 0 15.28422274 0 0 0 0 0 7.009712751 0 0 13.79054555 0 11.46359048 0 0 10.41894752 0 11.51679062 16.66217793 0 0 0 0 3.261219053 0 6.644309369 0 0 0 32.74408403 8.78639074 0 10.5416733 0 0 0 0 0 0 0 0 0 0 0 11.40632354 0 0

810 0 49.01296332 0 33.33333333 42.214694 0 0 15.54529189 0 33.74113616 0 22.10005036 0 19.34129089 15.69150061 0 0 0 0 0 19.33010492 19.99667 5.296557238 31.01023857 6.242318391 0 0 30.24450231 10.94599243 35.45903932 0 26.25485643 11.11111111 14.17127384 0 0 18.26666667 16.2304184 0 40.87043922 16.62469571 33.6232269 12.06607613 0 10.84924292 15.10599232 0 0 18.03605349 0 0 0 6.903035187 0 0 12.41069371 12.65603914 0 0 10.74153553 8.416666667 7.673333333 0 0 11.94080054 32.22486143 33.33333333

811 18.9378802 0 57.31011198 0 0 33.33333333 36.30894265 24.38911941 14.27813163 0 37.51379899 3.732414585 53.05941794 39.6102658 9.886938646 21.71799028 0 0 0 29.63447614 32.54408619 7.384477344 14.23913043 0 0 14.11344175 42.1138543 17.15888278 33.33333333 0 15.46018614 7.602890355 12.52774794 47.96608558 54.01431039 51.63646096 8.604269294 16.35401635 33.10656787 33.33333333 49.60161537 4.111237806 25.22942539 26.24627344 22.3677178 28.26254232 46.65425863 68.45507008 22.3731547 25.85401655 0 30.57335467 0 34.64687765 22.10763093 0 10.37655113 44.45483591 32.32644859 12.73042061 40.31333333 3.016666667 6.02 34.74943539 24.03703091 29.36383579 53.28135383

812 50.04198084 0 0 24.60095442 10.02920317 15.85437463 19.35567392 22.42208482 33.33333333 0 0 23.98458346 0 0 23.63609113 54.01041428 57.68861037 27.22363547 33.33333333 44.53932086 0 38.04139735 18.88008325 17.92460133 49.91758131 54.32485969 0 8.271287397 20.36418489 20.28444095 43.93287657 10.40622188 16.66666667 0 24.2231498 0 0 0 0 15.51960645 0 0 0 40.41018588 0 14.83834315 25.8759544 0 21.83730281 28.54220885 35.75578951 0 41.14834968 28.5845921 50.28592131 0 0 29.86205557 25.72362278 9.298998569 0 0 30.63333333 13.15604334 22.89967638 0 0

813 0 9.353143928 0 30.72534637 0 0 0 0 0 38.0085258 28.7497251 0 0 0 0 5.628407249 27.12298469 18.6259542 51.12602903 0 0 0 0 0 15.52493693 0 0 0 7.478500172 7.423057962 0 43.53651983 0 0 0 13.73289684 33.33333333 27.1155479 0 0 0 12.29637119 0 0 14.1926522 6.131419351 0 0 0 0 22.80177849 14.74931863 15.17593339 0 0 53.70654187 31.5988681 0 0 14.64956808 7.816666667 12.56666667 14.10666667 16.36062229 13.11806256 0 0

814 6.949069757 0 0 0 19.62168522 33.33333333 0 0 22.52451968 0 0 0 29.13435666 7.112068966 6.785971147 0 0 28.41011743 0 0 9.451116087 20.89726335 22.94378853 8.204144691 0 0 53.28095773 0 0 0 0 0 13.88148148 0 0 0 7.918719212 0 54.46124203 0 11.90716025 21.24667427 30.30421868 0 13.4562212 0 0 5.397110562 0 0 7.534176174 7.132784959 0 0 0 0 0 0 16.60304569 0 0 19.95666667 0 15.55660899 0 18.72405703 0

815 0 0 15.34384281 0 0 17.4789587 7.413449338 26.73225537 19.0552017 0 4.991976158 19.76026414 0 9.070641954 9.697242206 0 0 0 0 0 12.60144607 0 0 0 0 0 0 25.06204594 0 0 0 0 12.2556391 18.30119842 0 0 8.752380952 0 0 0 0 0 7.900406991 0 0 0 0 6.665800416 21.84434655 0 0 33.33333333 0 16.08582132 11.2257024 0 8.001711596 5.263157895 0 0 0 0 0 15.44252146 0 0 6.895903796

816 4.768882376 0 0 0 14.44305382 0 7.342062435 10.91124852 0 0 0 4.318608558 0 0 0 0 0 14.70737913 0 0 14.00322841 8.628599317 0 3.980899479 0 0 0 0 14.90884073 0 10.22061358 0 16.66666667 0 4.594127807 10.4955265 0 13.68233195 0 0 0 0 0 3.324653761 0 0 11.74786101 8.567258028 0 10.7724746 0 0 9.987205539 0 10.92313635 0 0 3.471277767 0 13.29279924 8.5 4.326666667 0 0 0 10.72767422 0

817 5.014976466 33.29135686 0 0 10.10732904 0 0 0 10.80881365 11.66903842 0 0 5.515374059 0 0 0 3.889519485 0 0 0 0 0 11.7173837 0 0 0 0 16.17445055 12.96914844 15.25459539 0 12.19951151 0 4.940184181 9.110183529 19.6004365 16.81034483 16.97931698 12.43219009 0 0 20.74706857 4.804589459 12.51321074 19.14068113 18.49499019 0 0 0 7.479316787 4.194040746 10.57235216 6.356730477 13.27380952 0 23.45398044 7.426619433 0 0 20.60291272 2.13 0 0 0 19.72995938 0 2.468364318

818 11.60970936 8.34253589 27.34604521 0 0 0 10.31720138 0 0 16.58129962 14.14544162 9.840654608 0 8.858381503 34.30225626 10.30145305 0 11.03291376 9.058149058 9.198296025 0 0 12.46980676 38.88011593 28.31516337 31.56169856 4.605187979 3.088831019 0 21.57886637 17.50512178 0 8.549946294 5.267765942 0 0 6.314285714 9.638368417 0 6.284378438 21.86652868 7.975421259 13.36685021 17.50567618 19.99348475 5.070791013 15.72192596 0 10.96017864 18.96274291 23.37670098 3.638856248 0 4.748741238 0 0 29.9402106 6.589147287 25.34688294 18.68376525 0 0 8.986666667 4.734768538 0 3.969497545 0

819 2.677500999 0 0 11.34036588 3.584034754 0 19.26267028 0 0 0 14.59905813 16.26342429 12.29085134 16.00735089 0 8.341735148 11.29888545 0 6.48248858 16.62790698 12.07001832 5.051592642 14.45325008 0 0 0 0 0 0 0 12.88120193 0 8.340740741 9.353492046 8.058228473 4.534679208 0 0 0 3.992242562 0 0 6.32843313 0 0 12.09592166 0 10.91476091 4.948963811 8.389240304 6.337514102 0 20.42874573 2.660158172 5.457609008 10.42878398 0 10.35952557 0 0 4.913333333 0 0 0 8.274470232 4.990073994 4.02104472

820 0 0 0 3.206714732 0 22.49970571 0 0 1.970918613 0 0 0 0 0 7.739242132 0 3.321380709 0 0 0 0 10.06790418 0 0 0 14.99389729 3.360920076 0 5.179405431 2.118323498 0 0 0 11.55528737 0 0 0 0 0 8.673986859 0 0 0 0 0 0 0 0 0 5.955961531 0 0 0 0 0 0 0 0 10.59513508 0 3.083333333 0 10.09666667 0 0 0 9.764462479

821 0 0 0 0 13.38550583 0 0 11.19963092 0 0 5.726286914 0 0 0 0 4.055059524 0 0 0 0 0 0 5.989029441 5.71330718 0 0 7.606511666 0 0 0 0 13.12803191 0 0 0 4.376378842 5.991963615 4.564155804 0 0 7.878397606 0 0 0 0 2.76383737 10.35343224 0 0 0 0 20.6581535 0 2.413737243 3.598036638 0 0 11.77328844 0 1.25432526 5.53 10.72 12.75333333 0 0 0 0

822 0 14.61597087 10.91392318 0 5.800511931 0 10.06403146 0 5.775502465 0 7.925849403 12.44377811 7.979515829 17.89261513 7.867805318 0 13.6 7.586282942 15.59847841 15.08935911 6.638176638 0 0 0 0 0 4.821851809 8.183891046 0 11.27489025 2.764350453 0 4.245922842 4.875400091 0 10.73253245 7.040967705 14.90360371 9.93697479 0 0 4.061436965 12.72763508 14.54443428 0 0 3.946254761 10.39215686 4.126984127 9.42330834 9.076993724 0 7.221220319 5.571293673 0 27.05680877 15.56787962 5.498507028 16.16138197 5.565969719 0 8.656666667 0 3.796367357 25.99766763 7.339604194 8.77688172

823 7.261516957 0 0 15.32742345 7.710271429 14.33817393 0 6.582411796 8.57538036 34.0462807 2.756266134 3.471015461 11.63146618 0 4.738966878 9.967299912 11.88125949 14.7475178 10.03543407 0 7.119205298 7.217125382 0 13.62784999 0 1.999812224 6.286877753 15.77633178 8.410024685 0 9.708118715 8.234388742 13.88940036 13.31327028 8.755945847 5.506276151 0 0 19.3890401 13.25609894 0 17.35397373 5.369400814 10.09401021 13.40372672 18.25264258 0 11.9992444 0 8.974901297 14.2257484 10.03385294 0 19.17278601 6.7052246 0 12.47265883 0 6.004230362 0 2.636666667 10.16666667 8.1 7.40948115 8.663558664 12.51933037 11.04322936

824 4.157269584 0 0 6.302234657 0 6.104507182 6.42833976 10.66230443 5.878299221 0 0 0 0 2.319015931 0 0 0 0 0 0 0 16.42235995 6.216160364 0 0 4.95451273 5.293804076 0 0 3.432835821 0 0 0 4.233921657 15.10787548 0 0 0 0 2.610574591 5.60134882 0 0 0 0 0 7.843137255 0 7.974563891 0 0 0 7.358728744 0 12.58709638 0 0 7.057710501 0 6.083925283 5.926666667 0 0 8.503138165 0 0 0

825 0 0 0 0 0 0 0 0 0 0 0 2.08484409 0 0 0 0 0 0 0 0 0 0 0 0 0 0 0 0 0 0 0 0 0 0 0 0 0 0 0 0 0 0 0 0 0 0 0 0 0 0 0 0 0 0 0 0 0 0 0 0 0 0 0 0 0 0 0

826 0 0 0 0 0 0 0 0 0 0 0 0 0 0 0 0 0 0 0 0 0 0 0 0 0 16.33962382 0 0 33.33333333 0 0 0 0 0 0 0 0 0 0 0 0 0 0 0 0 0 0 0 0 0 0 0 0 0 0 0 0 0 0 0 0 0 0 0 18.08526331 0 0

827 88.58121346 85.38402913 89.08607682 75.16362716 73.10371081 57.05761318 83.50762878 71.55565285 77.79989934 52.72044644 83.59159755 82.00036234 80.38901799 79.78836894 79.65398567 85.97764056 71.1973598 77.66619925 74.36608752 84.91064089 60.02849003 66.29261048 87.79481019 80.65884283 100 61.71215394 72.63003462 76.03977717 53.07723655 83.17395043 87.52753083 78.63757935 81.8646768 66.0221206 76.13617868 65.47290977 86.96706868 80.53224049 70.67398511 75.45933961 86.52025357 78.58458931 81.90296411 75.36155551 86.59627328 78.98352005 77.85717574 77.60859874 87.89845198 75.64582883 76.69725787 69.30799356 85.42005094 72.84218307 77.10964238 72.94319123 71.95946155 75.67049403 67.23925258 87.09577974 68.26333333 41.05333333 29.11333333 80.29101333 47.25351039 68.59623992 70.41542644

828 0 0 0 0 0 0 0 0 0 0 0 0 0 0 0 0 0 0 0 0 26.21412804 0 0 0 0 0 0 0 0 0 0 0 0 0 0 0 0 0 0 0 0 0 0 0 0 0 0 0 0 0 0 0 0 0 0 0 0 0 0 0 0 0 19.31666667 0 0 0 0

829 0 0 0 0 0 0 0 0 0 13.23327286 0 0 0 0 0 0 0 0 0 0 0 0 0 0 0 0 0 0 0 0 0 0 0 0 0 13.91190279 0 0 0 0 0 0 0 0 0 0 0 0 0 0 0 0 0 0 0 0 0 0 0 0 0 0 0 0 0 11.54482551 0

830 0 0 11.13704218 0 0 19.86408976 0 7.648921862 0 0 12.5897825 26.82186731 0 0 0 7.776437475 0 0 0 0 29.11996759 0 0 0 0 15.42107917 0 5.394270384 17.85230352 0 15.37918247 0 14.57701026 0 6.906802564 18.96941389 10.91328717 0 0 11.2369338 24.16404802 0 20.21319707 0 0 4.430615393 15.09470806 25.58599656 0 15.60022128 0 0 17.12436812 5.60263107 0 5.223854677 0 13.15746504 0 0 0 6.996666667 4.29 0 0 0 14.78063785

831 0 0 0 15.77128495 0 0 0 0 10.40211356 0 12.72708235 0 0 6.704639848 12.17329486 0 8.352567802 3.408434431 0 0 0 0 4.730868139 0 0 0 0 0 0 0 0 0 0 0 0 14.62825036 0 0 7.165109034 0 0 0 0 0 0 0 0 0 13.74212169 0 2.787538065 13.36107621 0 0 0 0 0 0 2.561019063 0 5.473333333 0 0 0 6.669987822 0 0

832 0 0 0 5.950133711 0 0 0 4.293125811 0 10.02230276 0 0 0 0 6.885327106 0 0 0 3.2668566 0 0 25.48667053 0 6.320332974 12.88965543 27.54931277 0 6.242344064 0 6.825965751 0 0 0 0 0 0 21.16534344 16.18876941 0 0 0 6.723484848 0 6.523633195 6.40270249 0 0 0 0 22.1496677 0 7.657120127 8.625349274 2.878614189 5.713082538 0 12.326237 9.342371671 6.102983362 0 0 0 0 20.41653848 21.14924394 2.269596113 0

833 0 0 0 0 0 0 0 0 11.05091159 3.860606061 0 0 28.68318796 0 0 0 0 0 0 20.79125133 0 0 0 0 0 0 0 0 0 0 8.667818962 0 34.95557659 0 9.024024024 0 0 20.37117273 0 9.851745576 0 0 0 0 2.600886401 0 0 5.647870698 0 7.289937919 1.598735067 0 0 0 0 0 18.40977872 0 0 0 7.41 4.03 3.823333333 0 0 0 0

834 0 0 0 0 0 0 0 0 0 0 0 3.746676336 0 0 0 0 0 0 0 0 0 0 0 0 0 0 0 0 0 0 0 0 0 0 0 0 0 0 0 0 0 0 0 0 0 0 0 0 0 0 0 0 0 0 0 0 0 0 0 0 0 0 0 0 0 0 0

835 33.33333333 33.33333333 61.14729745 0 0 22.74674768 0 33.33333333 33.33333333 0 13.57612544 0 0 33.33333333 0 41.74974218 33.33333333 17.89634398 30.06647673 0 37.54669908 24.98703992 41.06145251 0 0 33.98569315 0 0 0 19.8091157 0 16.07843137 0 0 33.33333333 0 17.89617486 17.14456392 47.02252576 56.81492109 21.45669291 33.33333333 24.0715763 57.20164609 0 15.05175632 0 18.80036438 0 0 0 0 0 58.18542141 0 33.33333333 34.88729988 23.71896461 0 20.13772849 26.56666667 0 0 0 0 10.53521541 33.33333333

836 33.33333333 66.66666667 27.71566036 78.27858134 100 24.05582923 100 54.72461899 45.21364151 86.11709118 53.93958431 52.95413784 50.751912 59.96202682 73.74559805 25.55689586 52.00038469 33.33333333 33.33333333 52.89419882 15.22026432 36.54242422 0 93.67966703 78.43140797 13.1014334 100 88.36338555 48.81436314 59.84070092 75.95299857 50.76260763 38.27254455 100 50.73584008 23.5717396 19.1883299 33.33333333 33.33333333 22.09639954 9.169285311 59.94318182 55.71522663 26.80970014 90.99641111 62.23605127 84.90529194 49.96576837 86.25787831 54.9601731 84.07706723 69.32187057 39.35970491 33.33333333 84.17521846 61.44281199 8.866878906 45.13760107 91.33599758 66.66666667 44.11666667 35.12 11.19 69.57020355 32.81116734 20.19894999 39.01591927

837 33.33333333 0 0 0 0 0 0 0 0 0 0 0 20.56490003 0 0 0 0 0 33.33333333 26.31454984 0 0 0 0 0 0 0 0 33.33333333 0 0 0 12.1948686 0 0 0 0 0 0 0 0 0 0 0 0 0 0 0 0 0 0 0 24.70798406 0 0 0 0 0 0 0 0 0 31.27 0 20.38771152 31.06373722 0

838 0 0 0 0 0 33.33333333 0 0 0 0 0 16.47731851 0 0 0 0 0 33.33333333 0 0 0 0 33.33333333 0 0 9.942481512 0 0 0 13.52421763 0 33.158961 0 0 0 42.83059615 30.83686462 0 12.47903187 0 45.20997375 0 0 0 0 18.28157701 0 0 0 0 0 0 0 0 0 0 25.5098055 0 0 13.19560484 0 0 0 10.01325797 0 0 0

839 0 0 0 0 0 0 0 0 0 0 7.167425392 0 0 0 7.195779979 24.91692449 6.313714176 12.02855492 0 0 18.11306902 12.98386533 20.87434601 0 8.678936605 0 0 0 0 0 0 0 0 0 0 0 0 12.9621606 0 0 0 0 0 9.465020576 0 0 0 0 0 0 11.53665964 9.659933088 10.18259363 0 10.111699 0 0 8.643597605 0 0 0 0 0 0 18.98188938 35.93250127 12.87010955

840 15.44007618 57.61807626 0 11.48728507 34.67559682 0 19.59163076 0 13.95595087 0 45.75494694 0 0 53.90018089 11.0347599 0 0 12.28310502 0 0 39.44203954 17.19755024 0 12.78622833 0 6.903844701 12.67004927 28.20450983 22.18979049 0 21.57361672 0 0 0 0 0 11.34479718 37.34961641 7.460943203 19.03765312 9.422278517 14.27176483 23.78351328 19.12845642 0 23.80331353 0 0 0 25.83295559 0 0 0 0 0 0 0 0 0 0 0 0 0 0 6.825336859 0 0

841 26.89534537 23.44176285 0 14.93997838 0 0 25.28963919 49.78643031 16.94243364 23.18064956 0 31.0785928 62.78965975 0 0 15.96064638 22.98219022 0 58.16296805 0 0 0 0 0 0 11.25631313 0 23.08575422 8.71476269 45.03863436 37.93475014 50.4225717 36.67025099 15.7133261 29.36818896 0 41.40987325 11.30937099 9.763166236 20.12333658 0 12.03544883 20.64791741 0 16.78722606 0 56.78492377 28.38389115 0 21.05824522 18.19770115 25.1951952 0 30.94069668 0 22.60339241 0 14.51718853 44.2465654 8.320910693 18.76 15.74333333 0 22.21499431 23.4608505 16.5485006 0

842 0 0 60.6216624 22.77838727 26.75145025 67.58483612 27.30960349 27.63645345 0 44.48781969 23.37248828 0 0 0 47.94952712 17.94708995 35.12439192 15.42317332 0 36.30816195 0 24.50369078 46.56957308 49.22742867 69.71038864 19.50207469 40.79288071 24.24961492 12.23776224 16.82263235 0 21.42586272 24.68233799 48.46052638 0 38.88150364 0 0 27.0477442 0 43.08827739 33.33333333 0 53.20512821 50.52195312 49.54195173 0 20.64545249 74.60204442 0 48.3380637 52.52206753 66.09081764 19.4904664 80.73430584 36.88525459 67.29988074 44.71302917 21.02025845 41.44373709 42.41333333 4.583333333 20.8 18.46402297 23.13312141 39.75624401 75.45618917

843 0 0 0 0 16.84189005 0 0 0 17.68155785 0 0 0 0 17.18259865 22.29857344 0 0 10.82916827 0 0 0 4.82257717 0 13.23192673 0 8.187929602 15.313626 0 0 0 0 0 0 0 0 18.62833184 0 0 0 25.40189201 8.359721343 12.53982938 28.28181879 0 0 5.525769212 0 0 0 11.64462704 0 0 0 0 0 0 0 0 0 0 0 0 8.15 21.63074845 0 0 0

844 7.397896796 0 0 8.573704273 0 0 0 0 0 0 3.365089977 0 0 14.01330171 0 7.875821991 6.839712012 0 0 0 2.930084131 0 8.372588605 0 0 0 0 0 3.926292211 0 5.690730838 0 0 0 23.39866119 0 16.27991256 16.64955071 7.693701037 0 0 0 0 0 0 0 4.022352259 3.995131982 0 0 0 0 0 6.839920592 0 0 0 0 0 11.96187379 0 0 0 0 17.32779247 18.34736923 0

845 0 9.891570479 0 21.84604827 0 0 0 1.366592574 7.941550191 0 0 30.4180224 0 0 0 16.98617922 0 0 16.26564935 14.78649258 0 0 21.50567784 3.577322018 7.444500083 23.79337986 5.186147186 0 0 7.919591023 4.176021554 0 0 0 0 0 0 15.52988632 25.87239013 0 0 9.868496284 0 8.890444522 8.348088399 0 0 7.32077965 5.753077897 15.85038814 16.10821779 0 0 7.65309637 0 0 0 0 2.183570514 0 0 0 0 0 0 0 3.120713306

846 12.1071963 0 5.62423957 0 9.225996081 0 8.186793991 0 0 0 0 0 12.01805759 0 5.063350102 3.44973545 0 0 0 0 13.3989402 23.65311088 0 0 0 0 0 5.128823506 0 0 0 0 8.650995341 0 0 0 13.07065599 0 0 11.06721163 0 0 0 3.824300699 7.796625144 8.371821415 0 0 0 0 0 0 0 6.873060023 0 12.6762643 0 18.81614481 0 0 0.83 0 26.68666667 0 3.785124921 0 0

847 0 0 0 10.55494606 6.581883088 15.66030073 8.043694141 8.058802692 11.43583227 8.049612403 9.960845058 0 8.023124647 11.72990522 0 0 18.28488611 17.91016002 0 0 44.22893613 29.82307093 0 0 7.755810658 0 0 0 20.69227843 15.82002903 8.721033868 28.15156558 16.83010987 27.03211906 10.7369123 14.7050015 7.555254849 19.16157557 0 11.15990991 33.23289404 0 18.01076648 0 0 8.752893519 30.47253118 28.1648207 9.387139792 12.27508811 0 8.138138138 29.0540212 0 4.778322946 27.83508869 29.96460366 21.9536375 11.88625592 13.05054885 8.046666667 0 13.71333333 11.11833903 0 0 6.670096022

848 32.3734245 9.048590407 33.75409803 9.81965068 5.923183711 10.9473151 6.023729845 13.15172097 32.04267518 10.15268377 17.54662975 38.50338481 17.16915801 0 13.65378944 37.78052701 16.76881974 43.55439338 25.57138261 48.90534546 0 0 19.26873037 21.17709426 0 30.35645802 16.1894849 10.24757912 32.23911394 14.39911323 21.90384687 0 13.1663058 0 36.49623754 27.78516303 10.33950617 0 15.87646606 13.20999675 5.896828713 17.95112735 9.275984045 14.95167015 16.54610727 4.004250592 8.720192796 11.48992402 10.25773789 13.3386959 17.35601736 14.14459914 4.855161159 28.20275994 14.48737122 0 2.735515599 0 20.66334971 14.33333333 0 37.05666667 0 26.57189525 25.46777384 25.34788615 14.7530015

849 5.786060853 0 0 0 0 5.807548054 5.554908583 0 0 14.12923457 0 0 0 3.174013538 0 0 0 0 0 0 0 0 4.28343011 0 15.08930062 0 9.847811926 9.083718413 0 0 0 0 0 8.794028458 0 0 0 0 6.285589137 0 0 0 0 0 0 0 0 0 0 0 0 0 0 0 0 0 0 0 0 10.88959625 9.74 0 0 0 0 0 0

850 0 2.095043434 0 0 0 0 0 0 0 0 0 0 0 0 0 0 0 0 0 0 0 0 0 0 0 0 0 0 0 0 0 0 0 0 0 0 0 0 0 0 0 0 0 0 0 0 0 0 0 0 0 0 0 0 0 0 0 0 0 0 0 0 0 0 0 0 0

851 0 0 0 0 0 0 0 0 33.33333333 0 0 0 0 0 0 0 0 0 0 0 0 0 0 0 0 0 0 0 0 0 0 0 0 0 0 0 0 0 0 0 0 0 0 0 0 0 0 0 0 0 0 0 0 0 0 0 0 0 0 0 0 9.72 0 0 0 0 0

852 89.59061015 97.90495657 93.83304404 89.3383038 81.11726432 35.15371667 91.70700234 77.00749567 62.50696379 81.34737978 83.2644892 92.6301656 84.23600293 78.86575661 81.70055816 80.43062468 87.75206873 90.15362753 93.53504448 74.35295293 93.91043398 86.34135472 88.62371572 78.17770847 89.14040991 75.78877205 62.34955713 79.45926197 80.68331981 91.63616003 100 89.85859568 67.8561601 74.74369181 83.94753191 76.65679964 95.17747577 100 69.43884673 88.25292937 82.85632576 79.63741145 91.52382292 85.63071909 77.47076325 100 69.95451154 91.22001021 84.9038672 76.16248935 79.44164683 86.08059899 95.72972046 81.39145453 82.51751137 91.17496629 69.82707032 86.36858077 76.96112525 100 15.95666667 26.35333333 39.05666667 85.63063139 74.92779226 88.87440697 94.34109595

853 0 0 0 0 0 33.33333333 0 0 0 0 0 0 0 0 0 0 0 0 0 0 0 0 0 0 0 0 0 0 0 0 0 0 0 0 0 0 0 0 0 0 0 0 0 0 0 0 0 0 0 0 0 0 0 0 0 0 0 0 0 0 12.76666667 0 0 0 0 0 0

854 0 0 0 0 0 20.55983701 0 0 0 0 0 0 0 0 0 0 0 0 0 0 0 0 0 0 0 0 0 0 0 0 0 0 12.68292683 0 0 0 0 0 0 0 0 0 0 0 0 0 0 0 0 0 0 0 0 0 0 0 0 0 0 0 0 0 0 0 0 0 0

855 0 0 0 0 0 0 0 0 0 0 0 0 0 7.033138402 0 0 0 0 6.464955522 10.3425429 0 5.514705882 4.761100794 0 0 0 22.93867246 0 11.37494346 0 0 0 12 0 0 4.798516822 0 0 23.42804933 0 0 4.126145596 0 0 10.83897863 0 22.52777847 0 0 0 0 0 0 0 8.082153761 0 0 0 0 0 0 6.496666667 15.99666667 0 0 0 0

856 0 0 0 0 8.804589627 0 0 10.29821628 4.159702878 4.255319149 6.514510278 0 7.305397075 11.23170928 0 0 0 8.363277672 0 15.30450417 0 0 1.583949314 0 10.85959009 24.21122795 0 13.1063799 0 4.842964511 0 0 0 0 0 0 0 0 0 6.763532764 4.25170068 0 0 0 0 0 0 0 0 10.18685955 0 8.750094969 0 0 1.928783383 8.82503371 13.98597946 5.237326879 0 0 6.94 2.156666667 1.073333333 7.701352146 0 0 0

857 10.40938985 0 6.166955962 5.25477707 10.07814605 0 5.354919054 12.69428805 0 8.634111819 5.090190264 7.369834403 8.45859999 0 18.29944184 19.56937532 6.808727729 1.483094794 0 0 6.08956602 8.143939394 0 10.65297525 0 0 3.834515366 0 3.230274823 3.520875461 0 10.14140432 7.460913071 18.13501206 3.478563353 0 4.822524227 0 0 4.983537863 6.848908037 0 0 6.057842825 0 0 0 0 5.348023182 0 6.778938393 0 4.27027954 18.60854547 0 0 16.18695022 0 7.434819176 0 7.376666667 0 0 6.668016468 9.619133303 11.12559303 5.658904047

858 0 0 0 5.406919133 0 10.95311299 2.938078607 0 0 5.763189254 5.130810262 0 0 2.869395712 0 0 5.439203545 0 0 0 0 0 0 5.540661305 0 0 0 7.434358135 4.711461903 0 0 0 0 7.121296125 0 13.15180498 0 0 7.133103938 0 6.043065524 0 8.476177082 0 0 0 7.51770999 8.779989791 0 13.6506511 0 0 0 0 7.471551485 0 0 8.394092354 4.35016835 0 0 10.08666667 0 0 0 0 0

859 0 0 0 0 0 0 0 0 0 0 0 0 0 0 0 0 0 0 0 0 0 0 5.031234172 5.628654971 0 0 10.87725504 0 0 0 0 0 0 0 12.57390474 5.392878565 0 0 0 0 0 16.23644295 0 8.31143808 11.69025812 0 0 0 9.748109618 0 13.77941477 5.169306038 0 0 0 0 0 0 11.25388723 0 12.07333333 5.76 0 0 15.45307443 0 0

860 0 11.75438596 22.346324 0 0 15.23508889 15.80716412 10.55973659 0 0 0 18.66711347 21.64430573 22.47109395 0 0 0 14.79686057 0 17.81100037 17.76875238 3.960703849 10.06922918 0 33.65793461 6.637168142 0 7.684793227 0 20.52238806 39.39418312 0 23.79037469 0 0 0 37.17795849 21.07489666 12.14141414 23.37042852 0 0 0 0 14.31284358 4.790214569 23.61171051 42.62590238 8.981121599 14.09214092 0 48.15345757 21.14639814 0 0 0 0 0 0 21.04429916 0 0 21.25333333 0 10.05686687 0 13.18661495

861 41.99225885 6.581765336 19.91595377 13.97247845 47.94133978 35.99116025 19.85920262 16.56912638 38.11039065 12.2306473 13.5021097 0 11.29102845 6.575573162 31.07409912 31.5281992 0 13.29318133 18.24247765 36.57875906 40.24438501 0 6.554559961 0 0 0 15.68701569 0 23.69112473 21.90143365 14.11691542 20.53959028 0 18.00637959 13.14536497 0 0 14.82856519 32.00542978 0 10.93259764 19.3055919 26.39786502 0 0 33.42948467 5.147630148 0 0 0 8.221024259 0 0 26.48003084 26.32459314 36.18041822 37.34268636 19.95498875 0 21.74685621 0 21.69666667 0 35.52020147 23.71352297 0 29.16877856

862 0 14.1350211 0 10.24230015 0 0 0 12.7838752 16.82350606 27.062191 8.400220334 25.17015941 0 0 33.33333333 27.70584608 29.56213651 20.41953069 16.57956758 0 0 27.65655491 9.18061583 0 0 14.34119542 17.49301397 18.41915152 19.52948273 0 0 46.00608064 7.5044719 18.85871494 14.87278273 0 0 0 0 0 14.79814219 12.38182373 11.14767348 52.39290137 19.55453852 9.307093071 0 0 6.639288159 0 22.13317791 12.20230686 12.34106698 18.70460048 25.97670401 0 4.562737643 57.67565122 50.96437155 0 6.3 9.37 11.14666667 0 0 21.60771102 0

863 0 10.37480064 0 0 0 0 0 0 18.05555556 13.48649224 0 0 9.646895915 12.3227958 0 0 23.65248718 18.53647276 28.84450959 0 0 37.53028898 21.40250674 47.20471102 0 32.68050799 0 0 0 0 0 0 8.272453803 0 7.142488517 28.65919143 15.47365466 0 0 33.32416957 14.38928054 0 25.97930525 33.33333333 33.33333333 0 15.44873351 0 18.87015121 0 7.097933513 0 0 0 0 0 0 0 0 39.34134888 6.356666667 0 0 22.31319748 18.23650235 48.3580135 0

864 0 0 0 21.35394699 8.479573881 18.09824445 8.584711861 0 0 7.908152337 30.60590706 8.440971669 0 0 0 0 0 0 0 8.096468562 0 0 0 0 0 0 0 0 0 0 0 0 0 13.46461736 0 0 0 31.27421648 7.164983165 0 0 10.61929722 0 0 19.02048976 0 0 21.08484239 0 45.49881436 0 9.966550618 33.33333333 0 5.058632735 0 0 0 0 0 12.72 0 0 0 0 0 11.01453941

865 0 10.37173015 0 0 17.23219637 0 9.627388841 0 16.50982728 0 0 10.45157989 3.792851933 10.60971409 0 10.23383372 0 8.668530867 10.04238533 21.07556193 6.358674959 0 11.6075447 0 41.88491578 0 14.06265224 14.55551937 0 30.37451759 32.48715969 0 38.70823284 15.32695375 9.16656316 23.84856098 18.98889696 0 0 0 22.40073569 0 0 0 0 0 50.98493384 25.04504505 0 25.12686675 8.54233921 0 0 3.984831531 0 30.90639155 9.985475672 0 4.854285222 0 10.91666667 12.7 17.46 0 0 11.72562231 8.700880912

866 22.45979561 0 14.35082141 13.85120723 0 0 22.55895085 7.446988974 0 19.8468411 7.245516878 0 21.63210015 0 9.799971219 11.7695354 21.31384639 0 16.75376575 0 26.42228166 0 11.93082659 0 0 0 26.87888767 7.534476959 6.947613976 0 0 0 0 5.307064253 6.543840636 0 0 0 21.97190149 21.87702265 18.53519114 41.13339586 0 8.351318945 13.77879481 7.098441058 0 0 17.81529499 0 18.01437556 0 5.485646874 13.44004546 0 10.99099099 19.33850463 0 8.213780175 0 22.42 0 0 10.32054605 10.16982161 0 17.31440187

867 15.44821584 11.20414673 29.96952126 16.51590532 0 0 0 43.32305487 0 12.85555736 24.933113 0 0 0 0 0 15.79068377 6.292960417 0 7.425864403 0 5.676778428 14.08348832 16.19003152 0 0 0 24.97136312 25.29716068 0 0 0 0 0 0 0 0 19.07036699 8 0 0 0 6.935468313 0 0 0 4.806991988 0 0 0 11.20015542 0 12.18693519 0 35.28344079 0 28.77059569 0 0 0 0 7.666666667 0 12.01306275 0 0 0

868 0 19.19831224 0 14.8243359 21.30985293 16.67706384 7.898780371 0 0 0 0 19.07052522 18.24945295 11.99908925 15.83432895 5.627487248 0 11.37162114 4.488823745 9.012345679 0 25.17567384 0 0 0 19.26745329 18.76465797 11.09302073 24.53461788 27.2016607 5.273671594 20.66058603 17.28857274 19.86871598 0 47.4922476 13.15891424 0 0 7.689863362 18.9440528 0 22.18565985 5.922446348 0 0 0 0 20.36730946 8.20646658 0 0 0 11.93584483 7.356629326 0 0 13.37834459 26.07047441 11.58647713 0 9.423333333 22.37333333 0 0 18.30865317 11.44583747

869 20.0997297 16.37983785 13.41737956 9.239825953 5.037037037 13.99844258 15.66380134 9.317217981 10.50072046 6.610118665 15.31313303 18.19965033 13.74336487 36.02173375 9.958267377 13.13509835 9.68084615 6.620842222 5.048470351 0 9.205905995 0 15.17122867 36.60525746 24.45714961 27.07367516 7.113772455 15.74167508 0 0 8.728070175 12.79374305 4.435894034 9.167554136 49.12895999 0 15.20057564 13.75195468 18.71627142 13.7385159 0 16.55989128 7.354028086 0 0 45.37476663 0 11.24421019 27.32683458 7.075711382 24.79099412 29.67768495 15.50661948 25.45464685 0 21.92219924 0 8.991015448 9.897088641 6.281018632 9.833333333 8.583333333 0 19.83299226 37.82328621 0 9.168946832

870 0 8.934351936 0 0 0 0 0 0 0 6.928625664 0 0 0 0 19.64083482 0 0 9.947131388 0 0 0 8.00744879 4.500967338 0 0 0 0 0 0 0 3.565806334 0 0 0 0 0 0 0 6.958741317 0 0 0 0 13.69014434 20.54416215 0 17.402509 0 0 9.95443038 6.601123596 12.44112579 0 0 0 7.136436472 7.465764459 11.85562324 0 12.27377007 0 0 0 0 0 0 2.234368018

871 0 0 0 0 5.60564663 5.6745378 6.861214523 0 0 0 0 0 8.350815207 0 0 8.322992377 0 0 2.438201299 12.78322836 0 5.439353696 8.755400991 0 10.84405088 0 9.560295145 0 4.732277526 0 10.56850413 9.69572567 5.076322329 0 0 0 4.832927755 0 0 0 9.374688156 0 0 0 0 0 0 6.046388102 0 0 0 0 7.316582105 13.92100193 0 0 12.84441682 0 0 6.905769659 0 14.10666667 0 0 8.73616135 11.47302041 0

872 0 0 0 0 8.572683355 26.82399594 0 0 3.033403991 1.238188335 0 19.5416972 5.036055923 0 0 5.377307761 0 0 0 0 18.73521388 0 0 0 0 0 0 1.83858899 5.576923077 2.253855279 0 0 0 10.19509603 0 2.15222411 0 7.115022549 0 2.800283175 0 0 6.3551728 10.20029674 0 4.902611548 11.72863924 0 0 0 4.76588047 0 0 4.743742551 0 2.4538223 0 11.39642016 0 0 0 0 7.473333333 5.301973908 13.64985163 0 12.00183256

873 7.198388721 7.400611621 3.798904538 4.11445739 17.21821525 0 0 4.940437039 0 0 6.489383632 0 0 6.791731805 0 12.50612122 9.35188832 7.265062213 20.41380853 0 0 10.75133964 8.996244452 21.18951766 0 6.606986133 0 0 7.696151924 3.364547396 0 5.478861087 3.532641127 0 6.069671586 7.046820626 7.515488911 5.478431998 0 0 0 8.885805324 0 0 11.93108654 0 0 7.186389979 0 0 0 0 0 0 14.60235098 0 0 0 4.07176096 12.53112592 0 11.21333333 0 0 0 0 0

874 0 0 0 6.620574062 0 7.663676856 0 0 8.575703172 7.930521092 7.812725645 0 0 0 12.42456099 0 0 0 0 8.442387488 0 2.202301903 0 13.60772028 28.22231502 7.974842767 5.043122351 11.25568828 0 0 11.53370257 7.849904863 8.633896653 0 26.97946356 0 11.83138505 0 22.81708417 7.6603804 0 3.285163776 0 16.81218581 0 0 10.43869112 0 0 0 9.142520837 16.58130184 8.786812884 2.069924513 7.436129421 0 22.75553217 0 3.525585177 0 7.42 0 15.48333333 0 2.172235087 17.65683621 0

875 8.735455168 7.613762486 5.648226351 6.51519762 15.59299337 2.910181582 5.360073656 17.55353558 0 13.22029725 12.33328686 2.11660656 17.89214869 16.74742938 18.29241803 9.195196272 29.63929731 15.29345703 2.826288703 7.946996341 3.140154225 0 2.841184529 0 0 6.156786458 9.221329185 7.169654023 11.75470771 9.67553481 6.381798002 0 0 3.835525062 0 0 7.135567941 0 0 12.67844851 22.47647405 20.61957869 11.95516812 0 0 4.041405132 0 5.491619452 21.54114481 22.08069128 8.861720404 4.587401876 4.538464424 11.31382466 0 28.00617997 0 0.806377292 19.41708957 0 17.77666667 6.18 21.89 19.76110445 0 0 3.345951887

876 0 0 0 0 0 0 9.308752764 0 7.153473187 0 0 5.419847328 0 0 0 0 0 0 0 0 3.658567939 3.364232892 0 0 0 0 0 0 0 4.294397897 0 13.46706931 0 12.24325006 0 9.345183636 0 1.984126984 0 0 0 0 14.57117388 0 0 5.365136007 7.62460443 5.122940803 0 0 0 0 0 0 0 0 2.827843381 7.327938904 0 0 0 0 0 0 0 8.652140442 3.015430871

877 0 0 0 0 0 0 0 0 0 0 0 0 0 0 0 0 0 0 0 0 0 0 0 0 0 0 0 0 0 0 0 0 0 0 0 10.00363603 0 0 0 0 0 0 0 0 0 0 3.876872284 0 0 0 0 0 0 0 0 0 0 0 0 0 0 0 0 0 0 0 0

878 0 0 0 0 16.11511809 0 0 0 0 0 0 0 19.51345184 0 0 0 0 26.06827112 0 0 0 25.69167773 0 0 0 25.35849057 0 22.07764505 0 0 0 23.63760766 30.83362198 23.1382373 0 0 0 0 0 0 0 54.49569757 0 0 0 0 0 0 0 0 22.86346612 26.12140086 26.01675123 0 0 0 23.35692151 0 0 20.80220742 0 0 0 0 0 0 0

879 84.06615611 76.05127396 90.55286911 82.74977093 36.89534331 56.92760783 78.46995906 77.50602738 81.23741965 70.68236766 73.36460386 72.92184891 49.20752833 76.46083881 49.64218616 64.59838237 61.00881437 41.42607824 74.32170147 70.82738782 74.46606395 44.54364534 74.90620269 65.20276206 60.9336341 53.90289408 76.17525332 57.65842365 70.23993976 80.41166462 67.95018896 39.8708314 51.92351791 50.58789154 66.95086486 71.45213559 68.68463034 85.42241847 70.22417451 76.86088792 68.14883779 12.71375465 67.1184852 59.29737312 67.52475131 85.69084731 48.92868392 76.15266166 78.45885519 67.96487834 47.76528857 40.26876963 53.34138936 67.95150635 77.9615196 62.40356126 30.74952166 68.61364041 72.98556429 47.48712693 53.9 39.86 62.69333333 74.93692164 75.44175193 62.21800295 79.40241666

880 0 0 0 0 0 0 0 0 0 0 0 0 0 0 0 0 0 0 0 0 0 0 0 0 0 0 0 0 0 0 0 0 0 0 0 0 0 0 0 0 0 0 0 0 0 0 0 0 0 0 0 0 0 0 0 0 0 0 0 0 0 0 0 0 0 0 0

881 0 0 0 0 17.53106429 0 0 0 0 0 23.20490922 0 0 0 0 0 16.53292181 0 0 0 0 0 0 0 0 26.10945697 0 0 0 0 0 15.40451849 0 0 22.12356641 0 0 0 0 0 0 0 0 0 0 0 0 0 0 0 0 0 0 25.29745561 0 0 0 0 0 0 0 0 0 0 0 0 0

882 27.89780521 0 46.13225284 0 0 0 0 0 0 0 33.33333333 0 22.92566984 17.90574416 0 0 0 0 0 8.920737058 0 0 0 0 0 0 0 66.66666667 0 33.33333333 24.33464614 0 0 0 18.25605723 0 24.66395749 0 0 20.80584147 46.23496206 45.9135543 33.33333333 66.66666667 0 0 0 0 33.33333333 0 0 0 0 0 0 26.43224022 0 0 0 0 0 12.24666667 3.61 0 33.33333333 11.58269441 0

883 10.63973064 96.66951324 33.33333333 33.33333333 64.11171645 16.93710692 31.51038053 66.66666667 0 54.76392423 20.47864718 38.83801928 66.66666667 42.42045231 100 49.77971492 55.6942405 22.03467677 66.66666667 16.31104789 0 52.13899075 0 100 92.00706558 33.33333333 50.14363407 26.42078793 0 66.66666667 61.21731488 57.38146552 66.66666667 81.22323042 0 34.09050051 0 33.33333333 66.66666667 0 33.33333333 23.13313777 22.31324877 26.24753313 93.12251598 33.33333333 25.0739844 55.7186375 66.66666667 42.59728816 60.56383149 75.31380443 23.17910616 33.33333333 100 66.66666667 66.66666667 53.66885485 87.46120422 17.07035755 39.72333333 4.746666667 0 33.33333333 0 33.33333333 81.83953097

884 0 0 0 0 15.80226904 33.33333333 19.91551746 0 33.33333333 33.33333333 0 33.33333333 0 0 0 0 0 0 0 7.738845475 0 0 0 0 0 12.40245262 0 0 0 0 0 12.82973621 0 0 11.20976693 0 0 33.33333333 33.33333333 33.33333333 0 0 33.33333333 0 0 33.33333333 66.66666667 0 0 33.33333333 20.94475775 0 25.04072264 0 0 0 33.33333333 0 0 66.66666667 0 0 0 14.27581428 0 33.33333333 0

885 33.33333333 0 0 0 0 16.39622642 0 0 0 0 0 0 0 15.42758917 0 0 16.80041152 33.33333333 0 0 0 0 0 0 0 0 0 0 11.14560695 0 0 0 0 0 0 0 0 0 0 0 0 0 0 0 0 33.33333333 0 0 0 0 0 0 0 0 0 0 0 0 0 0 0 3.83 0 0 0 0 0

886 0 3.330486763 0 66.66666667 0 33.33333333 0 0 33.33333333 11.90274244 22.98311027 0 0 17.62080911 0 32.1486307 0 0 0 0 33.33333333 0 0 0 7.992934423 0 9.050599896 0 33.33333333 0 8.998687194 0 0 0 0 65.90949949 42.00270918 0 0 33.33333333 0 0 0 7.085800207 0 0 0 33.33333333 0 0 0 7.122177186 0 0 0 0 0 0 0 0 0 0 11.38333333 0 0 0 8.69899923

887 22.69360269 0 10.02104156 0 0 0 48.57410202 0 33.33333333 0 0 9.496124031 0 0 0 0 10.97242617 11.29865656 0 0 0 14.52767592 0 0 0 0 7.472432699 6.912545404 0 0 5.449351791 9.285201149 0 0 0 0 33.33333333 0 0 12.52749186 3.868471954 19.34241673 11.02008457 0 0 0 8.259348937 0 0 10.88675214 18.49141076 17.56401838 10.15422718 33.33333333 0 0 0 0 12.53879578 16.26297578 2.68 6.963333333 0 0 33.33333333 0 9.461469801

888 5.435528121 0 10.51337227 0 2.554950216 0 0 33.33333333 0 0 0 0 0 6.625405246 0 18.07165437 0 33.33333333 0 24.41259628 33.33333333 0 0 0 0 28.15475708 0 0 55.52105971 0 0 5.099078632 0 18.77676958 15.0772761 0 0 33.33333333 0 0 0 0 0 0 6.877484016 0 0 10.94802917 0 13.18262637 0 0 41.62594402 0 0 6.901093112 0 12.99781182 0 0 0 0 0 52.39085239 0 0 0

889 0 0 0 0 0 0 0 0 0 0 0 18.33252335 10.4076635 0 0 0 0 0 0 42.6167733 0 0 0 0 0 0 0 0 0 0 0 0 0 0 0 0 0 0 0 0 16.56323266 11.61089121 0 0 0 0 0 0 0 0 0 0 0 8.035877723 0 0 0 0 0 0 0 0 19.78333333 0 33.33333333 21.75063892 0

890 17.75067751 0 11.25090016 23.32821301 0 22.5857264 0 39.38430192 22.54040053 34.32167062 0 0 17.96225419 13.4754573 0 17.36456988 20.61805044 21.07615139 17.87354637 0 16.20655563 16.04432943 27.81653301 0 0 0 0 17.04495417 8.790904049 35.17878301 0 20.57768924 18.99338323 0 9.578454197 8.156028369 7.236005089 0 17.48328189 32.5724911 0 0 0 18.81092956 0 30.33900541 28.77940571 7.753759398 17.52052545 33.09150513 0 49.68858086 10.83257827 0 0 0 23.57722199 10.63956964 18.299646 0 0 19.63666667 0 0 0 10.44033564 32.11192018

891 31.66223845 76.28877971 39.39839578 15.56156252 77.97974242 35.7195941 76.3820828 0 12.24393775 15.86348004 43.95160204 12.88674588 15.71218796 39.82412387 59.27458646 30.43320821 39.78430937 12.31466227 16.89991914 3.708801713 35.08924571 10.11893757 6.957287539 66.01772117 86.14823049 22.79798545 55.41489881 41.41955158 8.65058607 17.8675362 61.7297321 40.11258525 48.63576088 32.27922028 0 30.08349658 21.73048465 6.85759085 11.84195882 14.59533951 9.554799586 38.45953876 41.6457992 19.00479616 59.73645295 46.21875163 8.084118531 26.22052312 38.90122214 0 62.84577544 14.84103846 39.52365959 54.69649142 72.98506395 66.55867662 13.75619943 21.24818319 44.81701592 43.05193448 47.53 0 9.306666667 32.3367283 47.48756261 0 19.6763398

892 0 0 0 27.03511479 0 0 0 0 0 0 0 20.93934662 0 0 0 0 0 0 16.86830154 0 0 0 0 0 0 0 0 0 0 0 0 0 0 0 18.40506998 0 19.86451049 0 0 0 0 0 0 20.8723784 0 0 0 0 0 0 0 0 0 0 0 0 0 0 0 0 0 0 0 0 0 17.5740353 0

893 0 0 0 0 0 0 0 0 0 0 0 19.75014347 0 6.869324911 0 0 0 17.19416785 0 22.02214219 0 4.425326136 3.010126231 0 0 0 0 0 9.373266778 0 7.236780314 0 0 10.38047064 5.250979837 0 14.24406277 30.18953832 0 0 16.89525346 0 8.906721302 0 0 0 18.10126728 6.286549708 0 6.27775622 0 0 0 0 6.508494435 0 27.22942675 49.05746298 3.779833276 0 0 4.153333333 5.01 17.99131379 0 15.7742842 7.312440646

894 0 0 6.904731647 0 0 0 0 0 9.085948158 0 13.8512949 0 0 0 8.675702386 13.15789474 0 0 8.839050132 33.33333333 33.03148629 0 15.95898597 0 0 15.02833692 0 0 0 5.631879168 0 0 0 0 0 6.210739615 0 0 37.18286049 0 0 12.7233261 13.44796552 4.338871344 13.78430344 0 10.80841185 10.09906957 0 0 0 0 16.37974429 0 0 0 0 0 0 0 9.986666667 7.61 0 10.72182037 0 9.86856761 6.697840737

895 18.88763764 3.157396653 0 0 3.584604647 8.003481767 10.13736919 11.91155135 8.092933204 18.87188209 17.49844688 0 21.1947103 0 0 0 25.98309359 0 0 0 15.67271237 6.990784874 0 7.720423434 3.179059868 7.516255989 11.66648438 28.08102279 23.39038103 6.444205239 0 11.18934835 4.696821781 19.63229294 20.30922432 2.323544565 0 0 0 36.6059393 0 15.26887983 3.586076932 4.261206419 0 0 0 0 5.919962112 12.85229202 0 0 0 0 8.473635523 0 0 0 14.36060365 31.75025133 0 0 5.963333333 12.94657489 0 15.75929804 0

896 31.69944641 13.95512273 0 10.21890073 3.771217225 0 0 0 21.08939558 30.94296726 6.1942959 0 15.37107914 21.66454927 15.54913295 0 0 8.9375285 25.14549412 14.52167928 0 13.0153448 24.38210758 10.27762199 0 0 20.00334412 0 0 24.75880377 12.52861722 11.23380659 0 13.47408548 0 0 11.60284869 12.28132528 6.539351852 11.65761737 73.54994695 0 0 5.499776885 0 3.264094955 15.33333333 19.29302423 0 0 1.849846598 0 16.53987039 8.623068433 0 10.6969287 19.57713391 7.521418609 2.674333698 10.32741205 0 8.386666667 18.58333333 0 23.08514998 17.55904914 13.32217789

897 0 0 35.71213817 0 14.66443571 26.85594026 0 33.33333333 14.31105048 0 0 34.02977732 0 12.98855113 11.08284745 5.952161348 0 0 0 0 0 0 0 6.822877299 3.943246729 36.35242523 0 5.808414395 0 0 8.049215742 12.75564409 18.03090579 11.03434465 14.92826336 36.30192503 25.32208832 43.10530143 19.12075682 0 0 0 9.043309999 10.18353243 4.263149824 0 14.44080295 30.34707398 0 39.17629609 0 31.16823953 0 0 0 0 0 0 0 0 5.12 15.93 0 0 0 13.02443009 6.959152799

898 0 6.598700908 0 0 0 6.835257467 7.436985276 0 12.6363343 0 9.908103592 12.39398671 29.75976841 0 0 23.07556372 9.082644393 37.15783654 0 11.31119114 0 39.20622622 0 0 0 18.30499642 0 7.646057067 25.11211481 5.834230355 0 0 0 13.19958602 31.52800831 16.92426584 0 0 0 0 0 33.54825532 16.83094317 10.06733075 10.07507095 20.178148 0 0 15.81280788 8.602150538 27.27784846 4.302141158 13.68103673 30.2096896 0 11.93340625 15.86001793 0 11.25385406 0 7.89 0 9.713333333 26.00356264 14.86089239 0 0

899 0 0 6.733834244 23.85620896 0 0 6.043562736 15.3708134 0 0 8.596256684 0 0 5.177993528 5.417730755 10.0166021 4.531902206 3.319653443 14.37368869 15.10285234 0 10.19905096 21.87495967 9.161356108 6.729462911 0 12.91527269 0 24.68274726 4.284562252 10.45565463 4.130926471 9.643128322 0 0 0 0 7.566244126 7.831790123 4.568612712 0 0 6.539183883 6.961178046 12.14102283 0 4.452660344 0 21.84548241 0 8.026529507 0 3.043110735 6.470750552 12.03280609 10.81098843 0 11.53336559 4.814713399 14.87040213 4.56 2.116666667 0 0 14.56639502 0 13.92012796

900 6.042007638 0 0 0 8.511086533 0 5.625033837 6.326034063 4.583906322 0 0 0 0 0 5.913621262 0 0 0 0 0 26.67704291 0 0 16.78238872 7.12131756 9.172661871 0 8.367466355 0 0 0 6.948768752 0 9.267863428 0 0 0 0 0 4.627343393 0 0 0 6.231768762 0 6.878659847 7.531137851 0 0 0 0 0 0 0 14.18169598 0 9.969885997 0 3.439885896 13.00561653 0 0 0 0 0 4.319600499 9.295985819

901 8.397172569 6.032365265 6.927155371 4.442212978 0 0 0 0 0 3.005832212 0 13.61544438 0 0 2.953448714 0 0 7.056798623 0 0 0 0 0 0 0 0 6.292226292 0 7.219214216 0 2.71146478 0 8.709437002 0 0 3.46981263 1.449825094 0 0 0 9.797972724 0 0 0 0 0 0 0 0 0 5.679195516 0 7.539466522 0 9.416928525 0 0 0 0 0 13.38666667 0 0 0 10.13891508 0 0

902 8.757954493 8.620107962 10.15710385 6.083811955 0 0 6.190748871 8.899335387 0 5.904647915 6.395745038 7.390462784 6.07922186 0 11.32458895 3.582504545 0 3.767491927 3.392465252 7.576001287 0 0 6.368142001 11.54997935 0 0 15.56463114 0 0 0 0 0 0 0 13.0887297 12.57881244 15.60486433 0 0 0 0 0 9.036980942 15.39138083 12.4245313 6.842772927 2.799173398 14.52342918 0 7.323692198 0 26.79741369 0 12.69727403 0 7.450624487 8.044733045 8.076612438 0 0 0 0 5.576666667 0 0 19.25485934 0

903 0 0 0 0 16.30465713 28.99745327 2.838784431 4.504402373 14.74807292 16.20676105 0 5.859688554 0 8.303671395 0 0 12.41333846 7.584317754 15.7702908 12.72779499 8.134226884 0 0 8.925430883 14.81799549 12.55290334 0 28.72944475 12.77681578 8.705055204 15.76978145 16.30950952 6.650760513 2.953185498 6.755948272 5.005881584 0 7.994917522 12.98593418 19.37453619 14.46328293 19.5032074 7.993692382 0 5.778301887 4.413518887 13.83208064 0 14.67015524 11.96744598 9.210170408 8.165076548 7.156626506 25.30338572 0 7.797091439 21.41082961 17.88382269 6.525157233 0 7.653333333 12.59666667 5.27 15.01056502 8.030216467 0 5.584495603

904 1.773049645 11.59077458 0 0 0 0 0 0 0 0 0 0 7.201268057 2.845994951 0 22.79562191 0 0 0 0 0 5.122059476 9.55639276 0 0 6.822612086 0 0 0 0 0 0 9.032435458 18.58377211 0 0 0 0 0 0 0 0 0 2.70043313 7.680525164 0 8.634156639 17.64106696 0 0 0 0 0 0 0 11.50964451 0 0 6.502223341 13.81968864 0 0 0 0 0 0 0

905 0 0 0 0 0 0 0 0 0 0 0 0 0 0 0 0 0 0 0 0 0 0 0 0 0 0 0 0 0 0 0 0 0 0 0 0 0 0 0 0 0 0 7.204779756 0 0 0 0 0 0 0 0 0 0 0 0 0 0 0 0 7.857941834 0 0 0 0 6.356589147 0 0

906 0 0 0 0 0 0 0 0 0 0 0 0 0 0 0 0 0 0 0 0 0 0 0 0 53.15858656 0 0 0 0 0 0 0 0 0 0 0 0 0 0 0 0 0 0 0 0 0 0 0 0 0 0 0 0 0 0 0 0 0 0 0 0 0 0 0 0 33.33333333 0

907 75.02981565 73.75675219 82.91574078 89.47397507 75.18425634 71.00254673 85.34543286 80.27022818 80.66802076 74.88275883 63.94323801 73.13440428 86.71951008 88.85033365 79.80834107 73.62187355 87.58666154 81.5913917 80.83724395 79.69620372 65.18873021 86.76565758 84.07546524 62.74220105 24.90210039 71.45182271 78.14314257 62.90308889 80.00397 91.2949448 81.51875377 65.53783457 75.60736703 69.19517897 80.15532203 78.94549334 82.94531058 92.00508248 87.01406582 75.99812042 75.73874435 80.4967926 75.76454692 75.67641728 74.11664165 52.94523389 67.20345147 67.83550386 85.32984476 80.70886182 85.11063408 65.03750977 85.30390697 61.99934025 76.4013755 47.70639767 37.20904618 74.03956487 83.53273353 39.8413615 50.53333333 52.92 52.13333333 84.98943498 50.17116244 43.09220683 56.20624794

908 0 0 0 0 0 0 0 0 0 0 29.66101695 0 0 0 0 0 0 0 0 0 0 0 0 0 0 0 0 0 0 0 0 0 0 0 0 0 0 0 0 0 0 0 0 0 0 28.91981445 0 0 0 0 0 0 0 0 0 25.53624189 0 0 0 25.4753915 0 0 0 0 25.30311687 0 0

909 0 0 0 0 0 0 0 0 0 0 0 0 0 0 0 0 0 0 0 0 0 8.112282944 0 0 0 0 0 0 0 0 0 11.20388716 0 0 0 0 0 0 0 0 0 0 0 0 0 0 0 0 0 0 0 0 0 0 0 0 23.36550517 0 0 0 0 0 0 0 0 0 28.91327064

910 0 0 17.2081539 0 0 45.48432382 0 8.646368789 6.397094769 7.4985482 0 6.502779375 0 10.40822378 13.57310448 8.946721046 0 0 0 0 10.72591146 0 0 0 0 24.08269286 16.8813607 12.17241379 0 0 0 0 0 3.927608779 0 0 0 0 2.832530296 0 7.932001019 0 0 0 0 13.64297771 0 14.76414422 0 12.83391405 0 12.76295355 14.04978459 8.364443002 5.78623835 0 0 0 0 0 0 0 0 13.62534528 0 2.552778975 6.485974672

911 0 14.62207722 17.33433484 0 0 0 13.19456009 3.153541272 0 0 0 21.21441169 0 0 0 12.88218508 0 6.79516726 0 0 0 0 19.83837517 0 0 0 10.91245733 0 0 5.141787052 26.08799049 7.776833758 0 0 10.3194395 0 7.052414605 0 0 33.33333333 0 9.405799578 0 0 24.65055663 10.88463106 36.49469308 0 22.61519661 0 18.56215264 0 0 0 0 0 0 49.44981213 12.1997549 14.42322268 7.24 11.88333333 4.543333333 0 0 7.179487179 0

912 30.72430185 0 0 21.26961818 29.44016351 0 7.7491577 0 36.82519729 0 23.38459591 20 0 16.14647502 17.88009375 11.43157214 0 0 6.281325262 5.734593328 20.16761475 9.87962963 20.84511871 41.13442353 6.137865911 7.790990991 0 15.45190664 31.58277084 0 0 0 17.41167323 0 0 0 21.88178528 18.32535622 26.58783856 23.3008445 0 0 47.37215053 7.781967775 0 0 0 19.74545455 38.85160931 27.17302751 0 0 19.78248089 11.43162393 10.11077767 9.345687486 27.4343963 0 19.1826031 12.09425718 8.82 0 0 0 0 26.6718507 11.86701151

913 0 11.03993971 21.01949389 20.12963368 0 0 0 10.68754255 0 24.8720265 19.89708405 0 49.42058623 18.73848166 0 0 12.28049499 24.59633575 20.21707559 9.160982643 0 21.99028774 0 33.33333333 0 8.98554337 33.40826294 25.31969309 12.95079574 0 33.23370688 16.45358805 14.00262276 32.21942053 3.405684755 22.50327439 22.39022239 25.041511 13.27201051 0 12.25292126 46.65474625 16.34531874 0 33.33333333 11.08782958 17.27602568 7.896613191 0 20.65343547 5.162019593 0 5.791335102 14.49981064 33.9794297 25.26539278 4.025512338 0 0 0 0 9.81 10.47666667 23.66128268 14.58253665 10.06749964 0

914 33.33333333 18.20728291 13.29640933 25.24891499 0 0 0 0 0 2.609853529 7.002096436 0 18.49253463 0 15.47838936 24.38661229 33.33333333 0 0 0 0 56.78703704 0 0 0 27.17416899 0 0 14.73231731 0 0 26.1414008 0 0 0 35.93965844 0 17.96581909 0 0 22.79314888 0 0 8.96556809 0 11.3894081 0 0 0 20.49941928 17.26439994 32.12809474 0 0 0 0 0 0 0 0 0 0 6.846666667 12.97141891 12.66113088 0 11.08851837

915 0 0 0 0 12.93897632 21.75720426 15.39970185 38.80609833 6.934841819 5.828977933 0 23.89495879 0 0 0 0 21.62608957 9.691048323 0 0 32.43383081 0 15.54250347 1.794713566 0 0 0 21.16091954 12.16174662 6.792401257 4.526357511 0 0 0 0 0 26.28091873 0 18.07369415 0 0 9.593801629 0 0 0 3.818453594 8.829691709 22.54806892 10.71813673 0 20.73701843 0 20.06057835 10.4923433 0 0 13.45707657 0 0 19.07689785 16.04 0 3.196666667 0 0 0 0

916 0 0 0 0 0 0 0 0 33.33333333 8.461306835 0 0 5.180640763 14.63213733 0 0 0 0 46.44959107 0 0 0 0 0 25.20944402 0 0 0 20.3825376 33.33333333 0 0 63.38389722 0 13.23514212 0 0 0 0 0 33.33333333 0 0 0 14.31503177 8.602526046 37.39958954 0 0 0 0 9.072759539 0 0 0 11.54770075 0 0 21.13357843 18.91011066 0 5.176666667 0 0 0 11.13390313 12.47484909

917 19.16507024 18.71125612 15.9989985 33.35183316 0 0 0 0 0 0 26.3312369 12.11892164 0 0 30.07237801 0 21.05283834 17.41741742 0 33.33333333 36.67264298 0 0 0 27.19546742 16.36756757 13.93012803 0 0 0 15.94202899 9.997167941 0 0 56.34722717 0 0 23.65933657 0 43.36582217 10.54018445 20.01192042 21.62412959 33.33333333 0 0 0 18.56918911 0 18.84020369 0 21.77561837 19.28354875 0 0 41.40127389 29.307821 17.21685454 30.28926246 21.23907615 4.95 0 7.93 15.13796685 52.08413002 6.661482633 0

918 0 0 0 0 0 0 0 0 16.50953279 0 13.43624929 0 0 14.59485167 0 0 0 23.64228501 27.05200807 0 0 0 25.98317279 0 0 0 13.33131344 0 8.189831898 0 17.4909308 0 0 30.51963735 0 10.83005894 0 15.00797711 17.22879252 0 13.14841105 0 0 25.55136556 10.88449979 0 0 0 27.81505735 0 0 0 21.03227233 43.80241303 37.94395166 0 25.7751938 33.33333333 17.19480112 14.25643548 0 11.75666667 7.453333333 0 20.67220245 15.01994302 22.24481497

919 16.77729458 37.41944404 15.14260955 0 57.62086017 32.75847193 63.65658036 38.70644907 0 50.72928701 9.948737422 16.2689285 26.90623838 25.47983054 22.9960344 42.35290944 11.70724376 17.85774624 0 51.7710907 0 11.34304559 17.79082986 23.73752957 41.45722265 15.59903622 11.53647757 25.89506694 0 54.73247836 2.718985335 39.63100945 5.20180679 33.33333333 16.69250646 30.72700822 22.39465899 0 22.00513396 0 0 14.33373213 14.65840114 24.36776524 16.81657848 40.57417391 0 16.47653001 0 0 38.27440941 24.26057379 0 11.4093661 12.17960262 12.43994509 0 0 0 0 17.78 22.09666667 0 34.60398628 0 20.71305472 35.83883139

920 18.11602536 3.920816144 30.26634274 0 0 17.11100494 0 12.12370568 29.54471447 10.15955153 0 0 13.97063798 0 0 7.076939303 0 0 15.14200808 0 8.460773941 16.80955098 0 0 0 0 0 10.33767581 20.63202347 17.20847945 7.417757513 0 4.595507268 0 13.83128196 0 0 25.3655597 0 12.11547563 9.238478368 17.8178599 10.20654304 0 12.05609882 0 23.73917009 14.24550948 18.96235725 38.51581369 0 13.82545583 11.2575508 0 0 16.44612004 28.73043094 0 8.274421594 0 12.55 0 10.04 0 8.189537113 8.928941371 0

921 0 0 0 11.49570254 0 0 0 0 0 0 4.377372817 0 0 0 19.97438009 0 0 0 0 0 0 0 0 40.71243558 0 25.58430531 0 7.939313596 0 0 0 8.454003407 14.42514475 0 0 0 8.938808939 3.604074171 0 19.13897821 0 0 18.76991714 0 0 9.699213928 18.24501378 14.19784726 0 0 11.39875355 0 0 0 0 0 8.838767481 15.44205964 0 0 0 0 0 0 10.03064592 0 0

922 0 0 0 0 0 8.654898741 0 0 0 0 0 18.42390234 9.616677875 0 0 0 13.16089768 4.216954023 0 18.10926949 10.44361834 0 6.350076104 0 0 0 18.88690021 0 10.48151104 0 0 0 0 0 0 3.718016277 13.72363865 0 7.17050801 0 0 0 0 0 13.78488768 0 0 22.11417609 0 0 0 0 26.48480928 0 25.74755983 0 0 0 0 14.76584022 0 3.616666667 0 6.706398137 11.43065827 17.78189396 0

923 8.832630098 18.45967443 0 17.85014106 0 18.86647454 6.414114593 6.349478008 7.268363761 12.87701888 0 9.321227843 16.60988819 0 0 20.46256579 0 13.29539892 0 0 0 0 6.44209725 0 22.49976871 0 0 16.18380971 0 6.959845828 15.0911641 0 7.091222031 0 0 0 0 3.82626036 0 11.15966812 0 11.04615002 3.961254334 0 5.938655645 0 0 0 0 4.90913526 19.58192296 0 4.859967051 30.978589 0 9.526768147 6.568210938 0 36.18356845 17.21398818 0 0 2.826666667 16.28717182 0 0 10.83733974

924 18.22704592 0 0 16.36566314 0 8.189655172 0 0 0 10.34462375 31.68505779 16.42998028 0 21.27259714 0 6.687358916 0 28.41313296 0 0 9.232936355 20.7506337 5.789954338 0 0 14.96973773 0 0 13.38311143 17.24174654 14.64531262 23.16261798 11.85826849 24.92157132 0 0 15.60178971 8.406184232 10.90408639 0 33.82068529 11.98945982 18.23149528 44.00420757 5.639903929 17.50378484 18.42373343 6.633340165 16.38471641 0 0 44.39920707 0 0 5.543722636 5.835850957 0 14.28136419 0 13.91208445 2.296666667 5.246666667 3.66 0 9.700612918 0 23.92850185

925 0 0 0 0 18.16964286 0 0 9.32592745 16.1208577 11.73350582 21.13746959 0 19.36269535 0 18.39253462 0 34.69941945 0 0 27.45868604 0 15.12856917 0 36.7998573 0 20.73269912 18.53463964 9.22079835 0 0 0 37.00312323 19.93242496 0 40.13784778 33.33333333 0 7.845703247 0 12.78750592 0 16.13365356 0 0 0 0 0 0 18.47644104 14.09259542 0 20.83516806 0 23.30583859 0 13.21161049 16.30244244 33.33333333 0 0 5.64 9.513333333 0 5.397778991 14.50820529 24.40439196 0

926 0 0 53.99097027 5.828910247 0 10.66411849 33.33333333 0 0 0 0 7.116299179 0 0 46.69228658 5.526551706 0 0 0 15.22406384 0 0 21.19330289 5.126162019 0 0 0 0 8.222612184 0 0 0 0 14.01806624 5.771932783 22.78771206 16.20841621 0 59.49615866 0 33.33333333 0 0 0 0 47.3656857 9.594163247 15.74691042 19.27955353 13.60265684 33.33333333 0 57.39767287 0 33.33333333 0 0 0 24.7023214 0 0 7.796666667 20.30333333 20.94202899 13.71313795 0 47.01522436

927 30.32359539 55.71652435 15.74268699 29.40694523 33.33333333 30.95563821 0 26.98385533 26.06496957 0 15.11009871 7.028270874 0 57.18249075 14.94079872 0 0 20.03793441 67.46926086 0 48.97295637 18.20476416 33.33333333 0 66.66666667 18.09288072 0 33.33333333 0 58.58992818 41.53871081 21.20954003 0 41.74509535 0 0 17.73154362 41.10614114 0 22.17366521 0 0 19.16553596 0 19.54844565 0 0 0 12.04003948 20.33174777 35.68599015 0 0 0 6.922385376 34.85792752 14.75487221 17.8912737 22.23956301 44.53401016 20.18 8.736666667 0 0 0 15.55143938 0

928 24.50070323 0 0 0 48.49702381 0 60.25255207 45.21703353 0 54.88530002 15.49413735 14.90943099 40.4401006 21.54491211 0 26.64597442 19.1252082 34.03657968 0 39.20798063 22.88971499 0 26.89123608 13.75312166 10.83356462 18.36359561 49.76303628 6.811847819 41.06782302 0 0 10.17071535 24.89720302 0 12.71253101 29.61531706 0 9.846077148 22.42924695 22.62470692 15.61823066 33.42706792 14.56341619 55.99579243 27.39467769 15.82954849 29.99791945 27.06221659 14.85689229 0 0 12.49816527 0 45.71557241 21.4057508 20.12172285 16.61315257 19.05196914 0 0 0 18.29 19.89333333 50.66662207 32.42720254 0 9.404831483

929 0 21.90298507 0 19.05263778 0 5.558209906 0 0 21.0010945 0 12.19586375 26.77088849 0 0 0 33.60060987 33.01447467 0 17.38873106 0 0 29.10648199 0 3.608423449 0 2.256781517 12.81542387 16.17322139 6.21291886 0 21.30705496 0 17.20022949 19.31526709 27.54640646 10.54562127 27.79580287 0 0 0 7.989272355 9.585808789 15.10183805 0 15.63733059 9.601767037 0 0 0 8.548051024 0 8.442003764 0 0 7.047248027 0 8.192123408 0 8.600125549 9.574076992 5.75 0 0 0 0 0 8.814102564

930 6.816572238 0 0 4.526748971 0 0 0 0 0 0 0 0 0 0 0 0 0 0 8.172243701 3.798721757 0 0 0 8.978494624 7.839746361 0 0 0 0 0 0 21.09648726 0 0 0 0 0 16.82530526 0 0 0 0 0 10.22139458 0 0 0 0 0 17.15403409 0 0 5.469052599 0 0 0 0 4.561887859 2.27379278 0 0 0 0 3.544938918 0 24.32330197 7.519415728

931 0 0 9.7166278 0 0 10.02487961 0 5.775792543 0 0 0 0 0 9.552593511 9.671595616 0 0 7.031383982 0 0 0 0 12.84529783 0 0 3.144566579 0 0 0 0 15.19932452 10.31213192 0 0 0 0 0 6.974513565 0 0 0 0 0 6.876613899 0 0 0 0 0 0 0 0 4.399283656 0 0 0 0 0 0 0 2.293333333 11.79333333 0 0 0 0 0

932 0 0 2.88101771 0 11.44084342 0 0 0 0 0 0 13.2265822 0 0 0 0 0 0 0 15.24607532 0 0 0 0 0 10.38672742 0 6.997716895 11.08325961 4.799111908 0 0 0 13.43575226 0 0 13.86986047 0 7.2500452 20.62994148 1.875211445 20.11930381 0 0 6.298634562 0 10.52435156 5.720199454 0 0 0 0 0 0 0 0 0 0 0 3.115516893 3.076666667 0 0 0 9.364026531 0 8.153085748

933 19.59981482 14.17971298 6.255498449 12.91136522 26.78402691 14.29126814 8.690348044 0 24.49105732 7.870433556 22.02170433 9.83325355 0 14.80114801 15.85501445 9.239337758 15.57079049 8.392687855 13.15372081 10.82005581 0 15.47062302 0 0 0 0 15.91579689 8.640669085 0 5.783942833 0 8.443771299 14.69010892 0 27.95669952 14.09227486 0 6.476861364 0 9.86582872 5.639504778 0 9.387526054 14.97580135 0 0 15.2861607 0 21.55276745 10.00139295 28.5302561 11.05084746 7.873109796 31.56725327 18.93522161 33.69709368 14.9339136 18.23834417 9.821847164 0 5.81 0 0 15.48676705 14.52492212 0 9.969239374

934 6.887393768 18.56796117 0 14.51018118 7.17167773 0 0 5.149202804 8.329785724 0 0 0 0 0 12.57053505 0 14.56870748 0 0 0 6.146049297 0 5.968331303 18.38547583 0 0 13.30754906 6.805428018 0 0 27.62102731 0 8.558558559 8.731307284 0 0 0 0 26.79404334 0 0 0 7.991791264 0 0 22.30697625 16.73532669 0 0 4.960907389 0 0 0 0 8.879483562 0 6.290052253 0 14.74827246 0 0 0 4.353333333 0 0 0 0

935 0 15.81564987 0 0 0 0 0 0 0 0 0 0 5.307908205 9.232279397 0 0 0 0 0 0 0 0 0 0 0 0 0 0 0 0 0 0 0 0 0 0 0 0 0 0 0 0 0 0 0 0 0 15.15069482 0 6.024224961 0 0 0 0 0 0 0 0 0 30.47658863 7.643333333 0 0 0 0 0 0

936 21.6374269 0 0 24.09028646 0 0 0 0 19.02366864 0 0 0 0 0 0 0 0 0 0 0 0 0 0 0 33.33333333 0 0 26.33561644 0 0 25.54723966 23.02120141 33.33333333 0 0 0 0 0 0 0 0 13.66065938 0 0 66.66666667 0 0 33.33333333 0 0 19.88212862 0 28.93404968 26.30345636 33.33333333 0 33.33333333 28.77144547 0 0 11.76666667 16.20666667 0 0 0 0 14.02404922

937 45.05879227 51.43667599 81.14685604 34.7183713 54.60345194 75.68385225 91.30965196 89.07500465 48.15548831 92.12956644 77.97829567 76.94016425 94.69209179 66.41397908 61.90285488 90.76066224 69.86050203 84.57592816 78.67403549 70.13514712 93.8539507 84.52937698 81.18637087 72.63602955 58.82692031 86.468706 70.77665405 51.22056956 88.91674039 89.41694526 31.63240852 37.1264081 43.41799919 77.83294046 72.04330048 85.90772514 86.13013953 69.72331981 65.95591146 69.5042298 92.48528378 53.54324763 82.62068268 67.92619017 27.03469877 77.69302375 57.45416106 45.79577239 78.44723255 61.85944061 51.58761528 88.94915254 53.32450427 42.12929037 38.85196149 66.30290632 45.44270081 48.4283225 73.1560876 63.55114977 30.28 18.74333333 39.66666667 80.96829404 76.11105135 75.67669803 50.99416519

938 0 0 0 0 0 0 0 0 0 0 0 0 0 0 0 0 0 0 0 0 0 0 0 0 0 0 0 0 0 0 0 0 0 0 0 0 0 0 0 0 0 0 0 0 0 0 0 0 0 0 0 0 0 0 0 0 0 0 0 0 0 0 0 0 0 0 0

939 0 0 0 9.24304687 0 0 0 0 0 0 0 0 0 0 0 0 0 0 0 0 0 0 0 0 0 0 0 0 0 0 0 0 0 0 0 0 0 0 0 0 0 12.67678918 0 0 0 0 0 0 0 0 0 0 0 0 0 0 0 0 0 2.856744705 0 0 0 0 0 0 9.340044743

940 0 0 0 11.39506163 12.15856039 0 0 7.222534683 13.1758723 0 3.287868707 0 0 11.61606518 10.66430991 0 0 6.11888936 0 0 0 13.04566703 0 0 10.53020848 0 0 0 0 5.640332239 5.7243564 34.72213457 13.03098235 13.84617666 0 7.133661049 0 8.976533786 7.5339843 4.539217301 0 0 12.58440761 0 0 4.5204581 0 0 0 0 14.22103383 0 0 9.643377001 0 0 0 0 2.688304237 0 5.18 3.79 0 0 0 0 0

941 6.851327248 12.02261451 0 0 0 13.33913837 17.93446809 7.641140767 6.560339136 0 0 0 10.31851852 0 7.081843464 30.51409011 0 15.17050843 0 0 0 26.59722118 5.951093951 5.89078694 0 0 25.79600398 0 4.89539749 0 0 0 0 0 15.35720206 4.527927309 0 0 11.56973108 0 0 0 0 3.083247688 0 10.26570048 17.60112201 14.28063311 0 9.787486242 9.169124877 3.917967554 13.11610258 0 6.972230517 0 22.76081152 3.547593959 4.918032787 0 11.75666667 4.113333333 4.493333333 10.59419258 0 6.044455999 0

942 0 0 0 0 0 8.03117884 0 7.466401195 0 27.27107069 0 13.74471937 0 19.83403719 0 0 0 0 0 10.2308968 8.26873385 0 0 8.488127442 0 0 6.108312343 9.247740875 0 0 9.613534771 0 0 13.7166416 0 0 10.42347813 35.06046742 0 34.99755569 0 0 0 0 0 0 0 0 10.71346037 11.25120077 0 0 0 0 10.31077054 16.27389951 0 0 0 7.838782644 0 0 0 0 9.90990991 0 0

943 0 5.683910933 0 8.842123495 0 0 8.891491023 0 3.898216842 0 0 13.13671381 40.36551892 5.52512503 4.421487603 0 18.03343949 0 29.46366978 0 0 0 0 0 0 9.623782331 0 12.13847881 10.35013467 0 9.798257018 5.221706273 0 0 0 0 10.69434329 0 0 0 14.93039621 15.1684002 0 0 12.48749309 0 0 0 0 0 3.541202673 8.478781656 0 0 0 9.933065596 7.760885886 21.33463006 0 0 0 0 0 0 12.00040576 0 19.98334509

944 0 8.676246462 0 13.27300151 6.921675774 0 0 13.13796395 0 0 40.41916168 0 0 0 0 5.237761694 8.400584121 0 0 0 23.17886385 0 19.42640399 3.137929622 15.96634771 49.52556859 0 9.212107119 22.79635258 10.94487665 0 0 0 0 20.59820614 7.173672355 7.535864979 7.93751767 39.25904652 30.63804644 0 21.57889171 0 9.495868173 8.511254551 0 7.585751979 0 15.49620837 0 0 15.41983885 14.67901389 23.3207065 17.12055743 7.454682445 0 0 6.335546267 0 0 0 0 13.23236349 8.881278539 11.03925267 9.538479092

945 33.33333333 21.31071882 33.33333333 0 0 9.730250482 0 14.65073834 15.96783458 17.04458599 12.5936908 0 0 0 0 0 0 0 0 33.33333333 14.42023432 19.54969797 0 0 0 0 0 0 0 0 0 0 0 14.16781927 0 0 0 0 0 0 42.09115833 0 0 0 0 0 0 19.59183673 0 22.31878582 24.16420846 0 0 0 0 0 0 0 0 24.0833655 0 0 11.88666667 0 0 33.5497693 0

946 33.33333333 0 27.78959811 39.94800006 27.18339687 0 53.21258138 0 0 0 0 0 0 21.71726816 22.66902342 0 33.33333333 41.9988238 0 0 16.9066076 31.95391453 15.4969907 0 0 0 25.84317938 0 0 33.33333333 23.53507632 0 33.33333333 0 0 26.19967228 15.56202962 0 0 8.753071253 0 23.27455442 42.87198376 30.25008565 26.36624172 0 0 33.33333333 18.86775113 45.62797966 0 12.16632444 0 0 23.02256279 0 48.88623629 0 0 18.38356164 20.09333333 0 0 0 0 0 0

947 26.48200609 52.30650927 25.88712403 0 53.73636697 35.56609898 19.96145951 38.66502726 0 33.17978405 43.69927882 52.92194729 40.31842416 41.30750444 55.1633356 64.24814819 40.23264306 0 70.53633022 56.43576987 10.15446949 0 0 82.483156 73.50344381 28.99348552 42.2525043 53.84011939 61.95811526 50.08145777 51.3287755 60.05615916 53.63568431 12.07309599 60.12034185 33.33333333 48.70732356 32.74292339 41.63723809 0 0 18.16493314 33.33333333 57.17079849 42.27834065 56.40096618 74.81312601 19.05270022 47.01889481 0 48.90443016 47.32886026 72.20488353 44.12914552 42.57387872 66.33835245 20.59206631 49.23810675 61.74863388 25.49455069 37.71333333 9.64 7.6 76.17344393 69.20840579 22.29408066 70.47817582

948 0 0 0 26.54181331 0 33.33333333 0 11.21619379 48.27826558 0 0 0 8.997538409 0 0 0 0 0 0 0 18.91309902 0 0 0 0 0 0 0 0 0 0 0 0 46.19626647 0 21.63173367 0 15.28255773 0 0 42.97844546 0 0 0 0 28.81287523 0 13.7414966 0 11.01454752 0 0 0 0 0 0 0 0 24.30948283 9.249967837 0 30.30666667 0 0 0 8.466571342 0

949 0 0 12.98994453 0 0 0 0 0 12.11947157 22.50455927 0 20.19661952 0 0 0 0 0 36.71177841 0 0 8.157991879 8.853499298 59.12551136 0 0 11.85716355 0 15.56155381 0 0 0 0 0 0 3.924249959 0 7.076960427 0 0 21.07210931 0 21.81322054 11.2102753 0 10.35666998 0 0 0 7.903685323 0 0 12.68822724 0 22.90677098 0 0 0 25.87966923 0 14.94977169 0 0 5.686666667 0 0 18.60587002 0

950 0 0 11.56462585 5.223687613 18.9903999 0 9.330933093 0 0 0 11.35829764 8.405970149 0 21.54954221 6.558607234 24.97379455 7.52710306 0 0 30.19016285 0 20.44623139 0 28.55911852 28.83476492 25.06134573 34.7758099 8.733583925 15.79964404 4.095277894 0 7.582973382 7.277879407 12.32695811 14.3142333 0 21.58260938 0 22.56024425 14.14898936 11.13374835 0 38.34353554 9.414822267 0 18.04486106 44.21116139 14.39931227 0 9.796376463 11.12834432 11.026384 0 0 0 19.36962751 7.363431359 12.4749298 0 0 7.753333333 0 0 0 0 0 12.80225771

951 7.607739666 0 0 0 0 18.296955 25.79620219 0 0 12.14999108 0 0 11.7593673 0 7.423967212 22.65201982 0 0 21.76492399 4.657057466 0 0 0 0 0 12.95377129 0 0 13.26591044 23.66896012 0 11.14722383 13.44672191 14.10346442 0 0 0 5.477707006 5.805644873 0 0 14.71207006 0 0 0 0 33.33333333 33.33333333 31.21636937 0 0 0 0 7.761529809 10.91821671 0 0 0 0 0 0 9.34 0.85 0 29.48649713 0 0

952 20.43820792 18.2983683 0 10.16291699 32.97238471 47.27596439 0 0 0 11.47999106 8.704693999 0 0 20.26966544 0 0 37.3441875 0 0 9.096118035 14.15985904 27.3078264 4.016470906 0 0 0 0 0 0 14.70956958 14.49562854 9.420856126 12.09512536 0 13.95949326 14.88362801 0 17.77813702 8.824174619 14.99169213 0 32.05570461 0 14.87062672 15.06113126 0 0 0 0 0 9.003152088 0 19.35981709 0 0 0 28.4515776 0 0 15.72712418 19.25333333 0 5.24 56.75705554 0 32.53494771 0

953 0 20.41309602 10.99367013 37.76042008 0 0 0 20.84750031 0 0 0 27.85359335 0 20.60167131 0 0 0 0 12.54642176 0 17.38879261 0 18.50053937 24.83804273 0 10.8735122 9.89010989 12.16761799 9.00883472 0 35.95058308 13.47509876 0 12.32188855 9.777211967 0 0 12.97239915 0 0 49.20240277 0 0 11.2543153 17.68750661 13.90963406 8.561085973 8.753295669 0 20.25595293 9.183145922 50.64905898 20.3427065 17.53933624 8.866934992 19.29705215 9.356159971 0 74.20061575 13.75661376 0 3.226666667 0 0 8.694980695 0 8.575460201

954 0 3.94975395 3.942089656 0 0 10.4419294 3.879330442 17.71163183 32.61633087 0 4.278936944 11.69862483 19.79898974 7.799725056 33.33333333 6.621621622 0 12.04786212 25.45140524 0 6.629491945 0 0 8.001448226 0 8.01946472 8.764172336 0 0 0 0 0 6.149149586 11.50273224 0 26.55584558 8.962370692 0 0 19.87477408 0 0 13.97111677 5.692488263 16.16978272 0 0 0 0 18.52118766 0 12.41571387 0 33.36345499 0 0 17.37469094 0 0 15.19742883 0 6.226666667 5.95 0 0 22.64449723 19.0220878

955 35.2428033 33.33333333 0 0 0 14.40158259 0 0 33.33333333 46.48169706 0 0 0 0 23.19125945 24.89865586 18.81658653 33.33333333 11.56840934 5.364506488 0 0 41.33897536 9.507133593 6.51727677 0 0 6.935900865 6.70664861 0 0 14.1356256 21.61571318 0 0 0 19.82438637 0 0 19.18434398 6.417657046 33.33333333 0 0 0 0 0 8.484848485 0 0 24.33018125 0 11.16823765 0 0 0 0 22.61543135 0 0 19.36 0 6.53 0 0 0 13.58219024

956 11.747707 0 0 31.37878901 14.34293344 0 9.15745792 33.33333333 0 0 12.74910845 5.167419766 33.33333333 0 0 0 14.97186771 33.33333333 0 9.382609941 53.80959138 0 0 0 30.32137196 5.385509492 31.75131377 7.647686668 17.5336893 0 17.55435506 0 0 0 41.64758433 29.20576322 0 48.88852965 10.644949 0 0 18.62126327 36.90963691 0 35.43575268 18.63887908 0 0 27.37717295 0 38.61708119 0 9.299941418 0 48.68615294 14.03628118 16.613742 33.33333333 0 0 0 1.523333333 0 9.430503037 20.49330085 0 11.95561542

957 0 24.0054484 17.82661783 0 33.69428196 0 39.42874665 15.6217015 0 0 0 7.657711443 21.57396603 0 0 8.359538784 9.468785107 0 0 6.437827406 0 36.69117804 0 11.94786387 0 12.36009732 0 33.33333333 0 20.70738227 0 22.1861095 13.73746184 0 0 18.44970532 10.68798844 14.88322718 27.52768846 18.34164121 9.573883162 1.277628721 0 22.07901803 15.64582672 15.28847227 0 35.02921024 0 0 0 14.02814424 0 0 9.330406147 33.33333333 4.88175573 20.85840353 12.26019185 19.57671958 0 0 5.876666667 19.11199764 16.68686869 0 0

958 10.98565545 0 55.67299654 15.47418631 0 4.594448936 0 0 19.18149024 21.18334225 42.32473809 0 13.53434359 12.87171695 29.49283277 12.49436937 11.87147009 21.28547121 0 23.31176938 0 15.55476417 21.30669831 0 34.32658635 22.45982113 0 26.39743247 17.61785 36.81881013 14.1209316 0 13.96032856 40.2413137 0 0 11.02672921 0 0 13.45855926 0 0 0 36.68872942 0 19.42369928 0 0 24.54837911 19.5067535 7.738095238 0 18.61504723 25.54168187 0 0 15.95864239 10.71790198 13.5391924 0 5.893333333 11.77333333 11.60333333 0 24.63835264 29.06698256 19.75114309

959 13.97788667 0 0 0 0 4.989119683 12.4073297 12.48583302 14.86884556 8.704978549 20.58422488 39.21668046 0 16.90767904 0 0 0 0 28.66883966 11.55994843 8.012265031 0 14.83731605 17.14639307 0 2.886478109 14.8185941 4.784444754 20.0674229 0 17.87850173 22.05211281 11.71762015 9.503642987 20.30147714 10.90505787 27.91591592 0 24.6372988 0 23.67230868 0 10.77571078 0 0 14.69445426 13.89441931 0 16.85807857 31.91972945 0 11.88069892 21.21425011 15.79399709 22.19828921 13.96370583 0 0 0 35.74211365 6.863333333 14.86666667 10.04333333 14.70044379 0 15.75357251 14.31124553

960 0 2.425595238 0 0 6.305462653 8.485410867 0 0 0 0 0 8.432604144 11.63134784 0 0 0 0 0 13.9008688 0 0 7.627165859 0 0 0 0 6.174511796 0 0 0 7.232250301 0 0 0 0 0 6.059883097 0 0 0 0 4.588847192 13.65979381 0 0 0 15.41476682 33.33333333 0 0 0 10.7776305 0 0 0 0 4.969094655 0 9.974033909 0 7.913333333 0 0 0 0 0 0

961 3.250591017 2.73110285 0 7.36128759 6.080081612 3.648748319 0 5.664820826 22.56214149 8.073454959 10.05565683 0 2.684511231 5.784581186 33.33333333 32.54946203 2.746962493 0 0 0 7.481592906 8.001368659 0 0 4.531539228 5.315261444 0 0 0 7.914110429 0 0 5.044742729 0 0 0 0 0 0 0 10.53163917 10.63287343 0 4.558277654 0 0 20.41666667 0 0 0 21.6668952 5.627835103 6.929396357 4.250245476 0 21.30237064 18.20279091 7.563218391 0 0 0 5.43 0 6.378132118 0 10.41321008 0

962 6.003159558 0 6.092124814 0 6.587458746 8.745280924 0 15.83206879 0 4.841522596 0 3.582776378 0 7.839792022 0 0 3.813254097 0 0 0 0 0 0 2.86000286 0 11.76499654 0 6.203586941 0 0 4.744283139 5.749880211 0 0 6.226364127 1.871406782 0 0 22.10239263 4.164125086 0 33.33333333 0 0 11.41798183 10.85613416 0 10.75565361 19.37459703 0 0 0 0 0 5.484843838 0 0 14.65883121 0 0 0 7.36 0 0 3.398662311 0 17.80717286

963 0 0 0 0 0 0 0 0 0 0 0 0 5.776003757 15.21710953 44.74380631 0 0 33.33333333 33.33333333 33.33333333 2.341987613 0 10.15519568 0 0 0 0 16.15927642 0 0 33.33333333 0 0 0 0 0 8.751839039 0 0 0 0 0 0 8.048147274 4.156393096 23.56584094 0 33.33333333 0 0 0 0 0 0 43.7625351 0 6.407304165 0 0 7.768492727 5.433333333 0 7.87 0 0 3.545814063 0

964 0 12.94644098 19.05152627 31.47620843 4.48606466 0 39.38906463 54.43117224 10.77119184 0 0 10.42693839 10.32548858 0 0 18.91812001 30.58637084 0 19.43246453 0 0 10.27355284 33.33333333 4.066390041 0 0 12.86793954 19.04236308 49.2948098 33.33333333 0 0 9.075433557 45.22547287 0 5.503621869 6.014591883 13.95817175 0 0 37.96097335 0 15.46211865 33.33333333 0 8.882912878 17.91856651 0 2.595155709 7.712560715 33.33333333 0 33.33333333 26.83811485 0 33.33333333 21.14062913 0 0 38.19883476 11.33 6.55 12.17333333 8.503177734 6.186809768 33.33333333 0

965 20.70440719 0 11.86844444 0 19.77886021 48.96343881 0 0 20.16733333 41.61358811 25.67696587 20.28480626 21.70198549 13.12518945 0 16.2306089 16.76326304 15.096925 0 12.60720412 4.99972798 10.84349854 33.33333333 21.92388231 4.659689591 12.65234505 45.74444068 6.189575452 33.33333333 25.4192229 0 29.59891031 11.27516352 21.66208971 22.35542308 21.8744429 33.33333333 11.49261697 58.98033244 7.299418486 13.04483327 14.54528964 39.90721649 0 43.76541036 22.47719918 5.661764706 0 6.307557036 66.66666667 20.66822067 21.30423386 33.76729392 0 33.71024515 45.36429603 0 26.93886994 24.52947841 0 11.57 18.78666667 10.51666667 60.28853455 66.66666667 0 22.35437894

966 0 0 0 0 0 0 0 0 0 6.177166652 0 24.90072919 0 0 0 0 0 0 10.37088549 33.33333333 7.768547153 0 23.17813765 0 32.13240323 9.095741147 0 17.17405692 0 0 26.10108303 27.58345312 9.18344519 0 0 0 4.423164615 0 0 6.446361149 0 0 0 0 0 9.767492395 0 0 13.9587363 0 24.3315508 0 0 0 0 0 0 10.84812623 23.35929942 0 0 0 4.326666667 0 0 9.055566727 15.52616047

967 0 0 0 0 0 0 0 0 0 0 0 0 0 0 0 0 0 10.28163882 0 0 0 0 0 0 0 0 0 0 0 0 0 0 0 0 0 0 0 0 0 33.33333333 0 0 0 0 21.91535151 0 7.254901961 0 0 0 0 0 0 33.33333333 0 0 6.797209093 0 0 25.56484061 0 0 0 0 0 22.92012325 0

968 21.02310231 58.66174189 18.99230042 0 16.57425743 13.18177132 38.67197135 0 29.53940188 0 0 13.04852707 0 58.03332781 0 14.41521332 20.58591758 0 0 0 0 0 0 21.31936093 0 0 0 14.29097025 0 0 28.58905019 0 0 21.4411938 25.2627087 42.92081699 0 46.69678148 18.91727494 0 0 14.75204959 30.97087104 28.77505568 18.74486321 0 0 22.57767972 0 0 0 39.95380979 0 35.57830634 17.04237591 0 20.52603754 17.50574713 0 0 0 0 4.786666667 24.8301556 0 20.73195254 16.94012666

969 49.01873993 23.23511905 43.99560406 61.16250398 40.18781469 16.97534977 21.93896401 24.07193814 16.95993146 39.29426768 64.2673773 19.32361857 47.8806631 0 21.92286036 17.88659574 25.50423195 41.28810285 22.96244784 20.72612922 77.40814435 63.2544141 0 49.83036386 58.67636795 61.17165582 35.21310798 20.94017094 17.37185687 33.33333333 0 37.06775636 65.421215 11.67124362 46.15550409 27.82971146 41.41718803 27.8524298 0 48.75676195 38.46255421 22.14760682 0 25.28518606 0 24.45042046 33.33333333 0 57.76395392 25.62077262 0 22.33649075 25.96997639 0 0 0 21.95693451 22.4852071 42.13718825 28.46783191 36.41666667 39.42 10.08333333 0 23.74786125 0 27.37216107

970 0 0 0 0 0 0 0 49.64627591 25.69248036 0 0 24.89470746 22.10200321 0 25.24324692 16.89099458 29.99540954 28.1422884 46.04312677 51.6131958 0 0 63.26060979 0 0 0 33.33333333 23.44430139 18.28069442 66.66666667 57.76877251 28.52195595 0 23.51305729 0 0 0 0 57.36644744 25.55163589 22.39305726 0 27.59781967 23.2532379 43.03377956 56.2674855 39.95170384 0 30.11632134 51.82824616 62.91117324 0 56.73696025 20.33771323 53.71326291 46.11562968 12.68659703 0 26.72095303 26.66806988 0 0 14.16666667 57.31389406 0 50.5463254 14.81481481

971 0 33.83158932 17.28716124 7.95871417 20.23219985 15.92247029 37.45649489 0 0 18.57113697 33.33333333 35.32521165 0 44.89713276 23.76194321 26.28455921 27.91586998 0 0 0 0 0 0 0 0 0 0 14.16202845 27.49889429 0 25.22293823 0 0 12.83480923 16.57940663 43.83210483 25.89610244 38.62147206 17.92760603 0 0 26.4101113 60.78808265 14.60838956 9.544765537 0 0 64.04965068 0 0 0 36.11753171 0 49.26426206 18.19408827 12.14656665 22.69698301 49.7316957 0 33.33333333 10.72666667 0 7.753333333 0 31.6311697 0 0

972 35.06261927 0 0 21.97131919 0 62.84117424 12.55088195 0 0 0 0 0 0 0 0 22.42403074 0 0 24.56417193 0 0 9.676071055 0 0 0 36.32790206 0 0 0 0 0 0 33.33333333 0 0 0 15.16253208 0 0 0 0 26.4221773 0 0 0 0 22.06138587 0 0 0 0 0 0 0 0 0 0 0 0 0 9.246666667 10.97 0 26.3205593 0 21.27659574 15.60412872

973 0 5.393000574 13.55761988 13.98370951 0 0 0 6.214852199 7.640852974 0 0 0 0 22.457393 0 0 19.45026788 0 0 9.768829232 0 10.7815294 8.361735969 14.54253789 66.66666667 0 0 0 18.52399737 28.27832293 0 0 0 16.71039685 0 0 0 0 0 3.85280487 19.71316342 12.83893593 0 0 12.87956579 0 0 0 0 12.15191774 0 0 4.572036151 0 0 5.703019812 11.28586865 0 19.04761905 0 9.063333333 7.54 0 0 7.066036788 0 0

974 33.33333333 0 4.480335797 14.56770833 21.70142958 0 13.04839439 0 0 0 0 3.058238221 11.38600139 0 8.090086412 0 0 5.19104493 0 7.1942959 23.7070992 0 0 22.30670292 0 22.89473684 15.22449361 9.889031942 0 0 0 33.33333333 0 11.52711595 16.7539267 0 17.34018265 0 15.40572731 33.33333333 24.67536709 0 0 0 0 0 3.384513786 5.285512034 0 0 0 0 0 0 0 0 0 3.083860644 6.612380306 0 0 0 4.393333333 0 0 0 0

975 19.06982528 15.60228854 0 0 23.21484253 0 12.69777684 17.05636071 20.47186933 7.348438457 0 0 0 0 42.90472346 7.296938373 0 33.33333333 0 7.859174964 26.18790497 33.33333333 4.831980929 0 10.53715968 0 33.33333333 17.46302617 0 0 8.897894154 0 0 9.82027604 13.79066478 0 15.71856287 9.849643221 9.30021923 9.051046579 0 0 0 0 0 0 0 11.89509633 0 5.992431664 13.11815336 5.649169596 0 12.9956201 0 20.55103698 0 33.33333333 33.33333333 33.33333333 0 0 3.676666667 5.007724682 5.129493751 0 7.219475525

976 0 0 21.54511075 0 0 17.41086305 0 5.176640231 0 33.33333333 33.33333333 8.438625869 8.858496469 1.717030048 0 0 8.755387151 33.33333333 18.86301655 23.5645041 33.33333333 46.20906621 3.444953023 0 0 10.37049962 18.10883973 0 14.80933596 0 0 4.811377384 23.27136334 0 33.33333333 23.13851567 4.635687524 0 0 0 0 4.640633843 0 28.80503921 0 0 0 0 17.74503559 8.474067402 0 0 0 8.91188251 3.924268503 0 10.63635032 0 14.28571429 0 21.3 5.68 8.906666667 4.345047923 9.803480348 0 0

977 0 27.44207678 0 11.36201415 13.10113349 3.82549243 0 5.628898328 14.88915518 33.33333333 33.33333333 28.2832168 0 0 0 0 0 0 0 0 9.626234133 0 0 24.2091672 0 10.43859649 0 35.04161205 0 5.055010407 0 33.33333333 0 3.788127255 19.54266855 0 0 51.52888472 0 3.928892578 19.59824231 27.40555343 5.878584014 0 23.63288711 33.33333333 10.54427384 3.599182004 0 0 23.9706734 11.92467774 36.21596078 0 24.16838031 0 0 0 0 0 0 8.506666667 0 0 33.33333333 28.17707885 4.973229563

978 0 0 31.34154975 0 21.75039456 0 7.734056988 16.27697262 0 0 0 0 21.94733195 21.76953391 0 0 13.88306546 0 0 0 7.145428366 0 5.405033696 38.94159199 22.79617366 0 0 0 0 0 8.110395105 0 0 0 0 12.62250561 18.44538907 0 0 0 0 0 5.735513662 0 10.909002 10.39918117 11.27194746 10.27655805 0 0 0 0 0 8.4905221 0 15.48374687 33.33333333 6.446935475 0 6.665263453 0 0 0 0 0 0 0

979 12.53422212 17.73104479 11.78822258 30.15653466 0 0 16.51239494 0 31.30564216 7.41375791 0 0 35.70616698 9.158910281 0 27.1034771 0 0 10.52968475 0 0 0 14.69568659 0 0 19.96826498 0 0 20.88707796 0 0 0 10.06196999 21.80621738 0 20.40687389 2.801543365 0 0 24.28228675 13.62016991 2.282588191 0 0 0 0 12.7861752 4.894000902 18.80530973 21.55333704 0 12.97528762 2.475042826 0 0 0 9.360867651 7.404174849 0 0 0 0 2.34 7.012774038 13.03648608 0 57.38835138

980 21.9771439 7.868211223 0 20.36316472 0 23.64919032 15.17273576 11.8745289 13.69085174 6.146115191 23.27635984 0 0 0 7.585030171 0 18.24767749 0 36.31756676 38.02038803 30.18351873 14.21431892 0 9.186922665 0 0 16.44772712 0 0 17.16065075 20.20956812 19.44261729 0 11.60135462 9.409432308 7.113906359 0 15.58810475 23.32898592 0 10.3793844 0 7.369118622 47.3219412 3.420966306 34.70046359 0 12.24130762 14.63397299 0 9.214412286 9.211490264 7.521091366 19.58290776 32.66690578 30.05870793 0 0 24.0737308 13.11834378 0 4.726666667 15.44666667 21.85613682 7.786016949 25.98545773 0

981 0 0 0 0 0 10.33148242 0 8.022404516 0 0 17.85145768 20.12350345 0 10.829767 30.95669688 24.10181106 0 33.33333333 0 0 0 16.69113022 18.35056283 0 0 8.366402116 14.92324561 8.817251785 0 0 0 0 18.60979303 21.88087774 0 0 15.16896411 7.548486117 0 0 0 33.30311613 17.49763771 0 9.041191529 0 9.847131229 0 0 0 22.73561952 33.33333333 23.05127512 0 0 0 0 0 9.001722459 42.40005101 27.46666667 0 0 0 16.8016342 0 0

982 8.334740278 0 0 0 33.33333333 33.33333333 0 0 0 0 0 21.01833314 23.89304331 0 0 0 36.52993955 20.82004253 0 0 0 10.86826858 0 0 0 33.33333333 0 0 22.25405922 0 0 0 0 14.065762 25.41893802 0 21.09945644 0 0 0 0 0 20.97415507 33.33333333 4.086414546 0 33.33333333 0 0 25.08514734 0 0 17.83398242 0 0 0 0 22.4178783 0 0 0 0 0 40.78798186 0 0 0

983 40.9527357 6.970563161 56.67222534 33.50277736 0 4.534746761 36.21772605 9.369745463 15.87076757 28.55523952 21.15107914 0 0 22.50356633 29.86934063 39.77598721 7.542827921 33.33333333 12.11949329 0 36.14073885 0 0 33.04473209 76.0229949 0 25.66118656 51.31644014 15.59381238 10.60320452 12.71183124 48.70461053 33.33333333 2.356071601 16.11956232 72.74167249 0 17.030984 20.3340341 59.20226344 23.15422477 4.207920792 26.3625024 0 16.22391189 21.49361291 21.07081174 53.91348601 49.86454285 13.41249393 9.95552067 9.438982071 0 45.38875487 48.61061244 11.70243205 28.93024173 44.75434341 0 14.16343187 0 29.12666667 8.453333333 0 14.48371448 0 60.63482931

984 0 59.15880503 0 0 22.25945428 0 26.37759711 15.20283576 46.65825447 0 28.6703006 0 14.11321057 46.17891307 29.21229587 0 0 0 26.6111625 39.20169297 0 2.26251356 39.94849572 0 0 24.96693122 22.5825493 13.88827556 33.33333333 48.9444211 23.39661849 0 0 0 0 0 27.05249085 25.78484722 26.78740272 0 12.48934792 28.03578609 7.21471847 0 22.39574835 17.27228682 4.824300179 0 0 0 20.25670561 0 0 0 0 24.23286245 22.29088501 0 17.97288573 0 0 0 15.20333333 0 14.18903013 50.0673236 27.57153146

985 0 0 0 10.58365759 0 0 0 25.31092882 0 18.93864013 0 0 10.9809391 0 0 0 0 0 0 0 0 4.655063955 0 28.43945928 0 18.29960135 0 0 0 0 0 0 23.14241895 0 23.97009967 0 0 17.74522858 11.91858302 0 27.84683526 0 0 0 0 0 0 21.09202572 0 10.71932299 24.11892105 28.6977423 0 20.61007795 0 0 0 0 0 0 22.64666667 0 0 6.566515495 0 0 0

986 28.73538013 0 28.26041558 19.33149099 33.33333333 28.15124717 8.596772042 0 17.46256576 0 0 27.35691326 13.74113475 0 0 3.377333809 13.96415173 0 0 0 19.26996479 14.02522154 8.78015162 0 0 0 0 0 8.900948104 0 33.74526731 0 0 13.43009192 0 0 0 0 0 25.66700091 0 9.39657092 8.620977155 19.34472547 17.10942145 16.08467027 0 7.150127226 16.69836878 8.248185991 13.71882086 14.682861 41.31159288 0 13.85542169 23.26923077 17.4671074 21.98065246 24.33161087 0 0 0 0 0 0 0 0

987 0 0 15.06735907 0 0 0 6.955736224 0 0 46.36000515 9.050802747 12.3150002 0 20.4877536 0 8.945620589 0 12.5132908 18.22960661 5.718762241 0 0 0 0 0 0 0 24.02642177 0 0 9.936714839 19.35505633 18.63173217 30.72002692 0 0 13.40932088 0 0 0 10.17910856 0 0 0 10.27073152 0 12.26252159 0 18.80311537 0 0 0 10.28205821 14.41825942 0 3.919885551 0 10.84712582 20.26024811 11.59602742 2.75 0 25.83 0 35.38131798 0 0

988 0 18.49455895 0 13.83239832 11.07387905 0 6.679432818 30.21955654 6.317560463 0 0 8.912922906 12.0409112 0 0 9.231522271 23.71540331 0 0 0 9.520504367 28.84364001 0 21.49761764 23.9770051 15.03373198 14.64125378 0 0 23.29172362 0 12.49771585 0 0 7.914395316 0 0 16.30234934 17.63099424 6.488000606 15.95109909 5.297547246 4.990059642 0 0 0 0 0 0 22.61401034 0 0 0 0 4.867060093 0 31.31176586 0 0 18.72214591 10.75333333 11.49666667 0 11.47719651 11.35828625 12.63674389 5.761801873

989 0 7.507861635 0 2.386511025 0 0 0 0 0 0 0 10.27332705 25.23076106 0 2.376636455 14.56772506 0 0 6.722170829 17.05915675 4.885273258 8.439843217 32.92078983 7.831268325 0 0 5.744037622 1.951610747 19.91784697 0 0 0 6.282722513 5.945815191 17.16757237 20.14442115 23.26976772 0 0 8.642735043 0 19.75905882 6.970830935 0 17.45161441 10.44896641 18.66190193 5.603053435 0 19.9208394 0 4.635591038 0 0 0 6.816881259 0 0 4.359802023 0 0 1.433333333 0 19.31216931 0 11.31047479 6.031837361

990 0 0 0 0 10.13930777 19.36347543 0 18.54972552 0 0 0 6.108978245 0 0 0 7.186752054 16.60780585 0 0 17.59043348 7.962012782 0 0 0 0 0 0 0 0 0 3.992303992 0 0 0 0 0 8.949955317 17.17421482 17.01339638 0 0 0 3.513051964 47.95929711 0 0 3.753719387 0 5.047057607 0 0 0 0 0 0 8.269525268 9.460345204 0 0 0 0 0 9.213333333 0 4.047403836 0 0

991 0 0 0 0 0 0 0 0 4.425301917 0 14.00245657 0 0 0 0 0 0 26.43659538 12.00917168 14.09883049 0 0 0 0 0 12.81690874 0 19.70068541 0 0 0 24.36166276 0 0 0 22.34442699 0 3.928410626 0 0 0 0 0 0 0 0 17.93531526 0 0 0 0 0 0 0 0 0 0 0 0 0 0 0 0 0 0 16.55775875 0

992 22.29264857 29.55398244 50.52331261 33.33333333 26.73218914 0 0 0 0 14.34865714 26.57597241 0 17.91993607 0 0 0 17.00908144 25.11047333 16.86190983 0 0 0 30.72145987 0 0 26.07328798 14.55450352 23.46402587 36.38467511 11.38924631 0 0 20.85908293 23.94207167 12.29882634 24.12180367 12.43621963 0 0 17.35121148 15.59568809 32.74744986 18.45410628 0 0 33.33333333 0 8.994000694 0 19.22614756 23.53496718 0 16.05858507 38.69949128 18.76338631 59.69784635 29.56496435 0 40.59253076 10.05356407 0 9.61 0 0 70.5131815 0 11.54426559

993 25.32667499 53.76817981 0 45.96907942 42.68582621 53.81745887 56.84546294 29.10906236 19.87970877 66.66666667 22.60773277 66.14301469 17.17912756 87.39036214 42.14914233 56.60557972 24.35488162 27.66737639 12.44601492 0 52.34106331 13.66170507 25.80474135 66.02864233 84.17116422 6.189806861 36.78462682 26.29470672 0 36.86065512 74.57484996 42.53692943 43.45668012 51.68492954 23.69665425 24.11555693 14.0795353 5.643103707 43.64611136 8.808618504 31.02768408 22.25189934 35.11766939 38.91449132 51.71933102 51.37692398 53.13954376 14.7380363 65.47725389 16.22707291 36.02619277 82.33182352 38.65546799 13.11271975 33.64257571 18.78509444 35.94584658 75.72022087 0 38.05082036 48.46333333 21.44333333 5.733333333 54.11730751 0 36.64764424 37.68567766

994 0 0 0 0 0 0 33.33333333 6.961998383 49.34127185 0 0 0 0 0 33.33333333 0 0 0 0 0 0 0 0 0 0 0 0 0 0 0 0 0 0 0 0 0 42.27400807 23.29459131 0 33.33333333 0 0 0 0 0 0 0 17.36111111 0 0 0 0 0 0 0 0 0 0 4.938523198 0 0 0 11.61 0 0 0 0

995 33.33333333 0 16.92813629 0 0 0 0 0 0 0 0 0 15.41339727 0 0 0 16.72552748 0 46.23903235 0 0 28.68977463 5.064824912 0 0 0 0 0 33.33333333 7.45506297 0 0 12.4742504 0 0 10.98890635 0 7.400659379 16.31993696 0 0 19.10066842 0 8.986465219 33.33333333 0 0 0 0 38.47069058 0 0 0 33.67600446 0 0 0 0 46.65697674 0 0 0 4.583333333 0 7.32638179 0 0

996 0 0 12.99457283 7.49672836 0 0 0 19.32954853 0 3.508601419 0 0 33.33333333 0 0 0 0 0 0 20.84159166 0 0 0 15.95450711 0 11.6638912 0 6.086901842 15.29181211 0 0 8.971670573 15.42053002 0 28.66146107 0 0 0 0 18.79752852 15.23678853 0 14.87922705 0 0 0 0 37.4014201 6.557685081 0 4.202672711 0 36.31642793 14.51178451 14.62300739 6.968820315 7.574102396 0 0 20.41216879 4.306666667 2.733333333 0 15.99896614 0 13.60544218 29.29918486

997 19.0473431 3.779350896 19.55397827 6.106074815 0 10.10884354 4.763703517 26.04966521 0 0 6.757360925 11.63871489 0 12.60963786 15.94665665 0 16.3242519 6.896737956 0 0 5.036456927 17.50790912 15.88513268 0 0 30.88447047 4.899867846 0 6.489965096 9.803271441 21.43284605 24.12973724 0 14.98173713 0 9.217776405 11.95643896 23.761819 10.56203344 0 0 11.081434 15.34114012 0 0 7.315441853 0 0 0 0 20.21160558 0 0 0 0 0 3.76836898 0 0 0 2.696666667 9.78 9.196666667 0 8.626220363 16.41358025 0

998 0 12.89848685 0 0 0 11.55867531 0 0 12.9000929 0 30.05647733 16.10929217 0 0 0 29.22164028 8.978451716 0 12.44387121 33.33333333 25.60474639 24.70488715 0 6.169015539 15.82883578 0 41.22465944 9.869307464 0 20.0027401 0 0 0 0 20.92903756 0 10.30384272 8.758459136 0 15.98212185 0 0 12.69480519 4.139746351 0 7.974300831 25.1714216 15.97222222 16.15989244 0 0 11.43933818 8.969519016 0 0 6.278713629 0 12.00585652 7.811969296 31.48344677 3.866666667 8.143333333 2.7 20.61345841 0 16.77557458 15.7444668

999 0 0 0 7.094784072 20.44267688 5.151546859 5.05750021 0 13.45362456 15.47607477 0 0 16.15420578 0 8.570867687 6.986027944 0 13.88881695 0 14.13581103 9.055720587 15.43572403 22.52384119 11.84783502 0 12.37163475 2.536342381 14.5843727 8.500214343 14.48902406 0 0 7.789456531 9.391261659 14.41402078 9.211529663 0 10.03874202 12.45852187 5.727186312 38.13983931 14.81854839 0 0 14.94733564 0 0 5.533209571 6.758110986 26.07608895 16.02456176 6.228838295 0 0 32.97103058 0 13.68637249 12.27392261 0 0 2.006666667 0 0 9.270267942 9.486812513 0 5.72640509

1000 7.256804998 5.30128875 0 12.25498551 0 4.68401487 0 9.75793375 9.149703138 0 5.156926407 3.690106373 0 6.668173599 0 0 6.664156627 8.712879546 14.16002776 6.082062455 0 0 0 14.13476206 2.886917336 0 0 0 0 0 5.030702496 12.34022989 6.567557396 0 10.2757606 0 0 0 0 0 0 14.50396314 6.653859588 0 0 8.603257741 9.437885201 0 0 0 0 0 8.464714715 17.649842 0 22.97927494 0 7.868795254 13.17038955 0 0 0 3.456666667 0 0 6.307258633 0

1001 5.585211546 6.083567872 0 10.55911681 0 0 0 0 0 1.860282133 0 0 0 10.82225505 0 0 1.645055158 0 0 0 0 0 0 0 0 0 0 0 0 2.898550725 8.596356171 0 0 0 0 0 0 0 6.360500216 0 0 0 0 6.985294118 0 0 0 0 0 8.562003299 0 0 3.692281333 0 0 0 0 0 9.72270364 15.20201609 0 0 0 0 0 0 0

1002 0 0 0 0 0 0 0 10.08757526 0 0 0 0 0 0 0 0 0 0 0 0 0 0 0 0 0 0 0 0 0 0 0 0 0 0 0 0 0 0 0 0 0 0 0 0 0 0 0 0 0 0 0 0 0 0 0 0 0 0 0 0 0 0 4.67 0 0 0 0

1003 0 0 0 0 33.33333333 0 0 0 0 0 0 0 0 0 19.74893743 0 33.33333333 0 0 27.25127088 0 0 0 33.33333333 0 33.33333333 0 15.80105896 0 0 0 17.78080533 47.90988477 51.18570474 0 0 0 0 0 0 0 46.0558417 0 0 0 0 0 0 0 0 0 0 0 0 33.33333333 0 55.32057737 0 0 0 17.85 0 0 0 0 0 0

1004 81.99567507 79.19752052 80.13697205 71.82621107 47.28525122 75.40752736 100 63.46045705 84.20737511 98.13971787 94.84307359 50.53390708 74.39076988 75.66056593 44.59945437 85.53450788 50.00529625 83.03008837 75.50894837 53.76060872 100 80.77519621 81.44231685 44.87094291 63.74132031 56.71886459 100 66.66666667 95.58919271 88.16915761 66.29661871 54.32643678 16.09584745 0 70.31055337 100 100 100 71.66144959 100 91.81455191 26.23505254 68.85371702 75.69111229 100 48.99743756 90.5621148 55.25935558 100 58.10466337 72.93848432 100 75.83984523 77.66356317 66.66666667 77.02072506 33.33333333 92.13120475 77.10690681 71.8741556 28.14666667 39.96666667 23.20333333 96.00730689 94.12968485 84.98942918 77.93579937

1005 0 0 0 0 0 0 0 0 0 0 0 20.73802127 0 0 0 0 0 0 0 0 0 0 0 0 0 0 0 0 0 0 0 0 2.433731204 33.33333333 0 0 0 0 0 0 0 0 0 0 0 0 0 27.27272727 0 33.33333333 0 0 0 0 0 0 0 0 0 0 0 0 0 0 0 0 0

1006 0 0 0 0 0 0 0 0 0 0 0 0 0 0 3.162202381 0 0 0 0 0 0 0 6.916261789 0 0 0 0 0 0 0 0 0 0 0 0 0 0 0 0 0 0 0 0 0 0 0 0 11.40731108 0 0 0 0 0 0 0 0 11.34608929 0 0 0 0 0 4.596666667 0 0 0 0

1007 5.162308386 0 4.297139759 5.35968661 0 0 0 16.69403394 6.642921755 0 0 0 13.38657539 0 0 0 0 8.257032088 0 0 0 0 0 7.660961695 0 0 0 0 0 0 4.664525942 0 10.66992849 0 0 0 0 0 0 0 0 0 0 7.005355334 0 6.715395158 0 0 0 0 2.313157147 0 0 0 0 0 0 0 0 12.92382831 0 0 6.356666667 0 0 8.703312192 4.343410671

1008 0 0 0 0 0 8.414033974 0 0 0 0 0 0 0 0 0 0 0 0 0 0 0 19.22480379 0 0 0 0 0 0 4.410807292 0 0 0 0 0 0 0 0 0 4.042690815 0 0 0 0 0 0 0 0 0 0 0 0 0 12.00315873 4.686594833 0 0 0 0 0 0 0 4.673333333 0 0 0 0 0

1009 0 9.417622862 15.5658882 0 19.38141545 11.49442379 0 0 0 0 0 25.03796527 12.22265473 6.849005425 32.48940582 14.46549212 8.352158635 0 10.33102386 12.90605795 0 0 11.64142136 0 33.37176235 9.947802079 0 17.53227438 0 8.932291667 15.41179668 15.552528 16.32305069 15.48096192 19.41368604 0 0 0 17.93535938 0 8.185448092 13.20514262 24.49242339 10.31823825 0 35.68390954 0 6.060606061 0 0 24.74835853 0 0 0 0 0 0 0 0 0 0 6.366666667 6.19 3.992693111 5.870315149 0 17.72078996

1010 42.62591864 44.96478153 11.02761272 59.4535456 46.91471061 0 54.48702724 37.06539404 11.50649108 49.33531679 57.95607602 13.86247046 12.24136063 13.4991119 27.05961152 19.87968977 25.15979569 24.81926175 31.7933998 2.916325974 41.8751171 7.116770309 0 45.30406375 27.65673633 32.86758346 19.27988703 32.65943274 13.19730043 42.25850864 14.33542102 38.42080863 0 30.61744208 4.938585345 32.26805653 68.68000305 39.69284621 32.16454676 9.491712707 14.51731345 33.43574429 0 26.12643778 32.27538253 25.98654301 35.03874168 17.45970091 52.65811005 48.01017555 17.7994933 34.1812342 23.96162961 21.53888889 60.15731432 43.78973594 7.816452936 8.099088692 49.3911732 36.79690564 31.41666667 0 6.666666667 11.93815174 24.86997707 30.11138813 0

1011 21.44106758 0 0 0 0 7.235548565 0 0 8.456934686 15.73558063 0 0 0 28.01341472 0 0 0 16.3947502 0 11.45719887 0 0 38.5807894 23.28571429 0 0 23.20275822 0 0 0 0 0 16.2647926 0 11.44382544 20.4648234 0 0 0 42.66565468 6.416451287 0 18.35535977 0 14.54793986 0 0 16.81534976 0 0 0 15.79459249 0 16.9704142 0 0 22.10395828 11.54427319 0 15.00984673 0 6.383333333 0 14.44240517 0 0 0

1012 0 0 13.06842557 0 0 53.05679816 0 15.3308384 33.33333333 0 0 0 0 0 0 4.296332598 17.64954555 0 3.982415309 7.413228553 0 0 13.84474112 0 0 10.6428725 0 0 0 0 20.20833333 0 0 24.72424558 0 0 0 0 0 0 0 0 32.74842707 0 0 0 0 10.99064214 0 0 0 0 0 0 0 7.931107804 26.83677959 0 0 0 0 0 5.7 10.95167409 0 25.56257901 29.03599589

1013 0 0 9.251804996 0 0 0 6.790827315 0 0 0 0 0 25.08682565 0 0 0 0 0 0 0 0 47.51583757 0 0 0 0 0 0 20.62570876 33.33333333 0 0 0 0 41.08852181 0 0 0 0 0 0 13.1862953 0 0 0 0 13.91815731 0 5.976687788 0 3.365906623 0 0 17.97283177 9.070915138 0 0 32.74377499 0 20.13324643 14.03333333 5.936666667 1.986666667 0 0 0 0

1014 0 38.90191176 0 8.942793612 0 0 15.13782196 0 0 0 0 21.25860374 10.58220211 0 15.54879766 0 11.66916008 0 0 30.41700736 0 7.13157635 0 10.04761905 0 0 40.07235683 0 0 10.944 0 0 0 0 0 12.86850993 4.795698925 11.00309104 0 15.8121547 32.52103811 0 0 13.79248977 6.31991864 0 5.801104972 0 0 0 33.33333333 13.39247993 13.64930947 0 0 0 13.7049638 0 0 0 0 0 2.246666667 10.30461807 11.99444125 0 0

1015 11.89226576 14.25801369 25.12801555 17.64107004 11.57334385 0 7.404580153 25.4745603 8.114939965 19.9855052 0 15.58591273 0 16.78273249 39.60705515 39.50485581 15.68378778 0 0 5.690897184 43.76007288 0 30.70034473 0 72.34326367 6.238967065 0 4.571886906 30.08951245 2.440158029 8.794642857 12.13310899 66.66666667 15.75007929 21.88950789 0 14.35366682 6.528735632 20.31693472 11.36350192 18.81601988 17.08070175 0 38.30216189 0 56.47044365 0 0 16.47864446 12.87216709 17.81865966 19.09295253 33.33333333 16.36291913 2.813814347 29.93622484 0 11.8464793 32.08060099 0 0 11.62333333 0 8.609599208 0 26.09768438 12.39901651

1016 12.34157161 0 0 0 0 13.60986851 0 0 0 0 25.24275679 33.33333333 34.60791814 0 0 0 0 47.06555795 64.22418489 21.87613447 0 0 8.956500489 12.21111469 0 7.181251344 0 18.22954527 0 0 12.42668257 16.11274904 0 20.2991453 0 34.39861014 0 0 43.33744856 0 0 0 0 0 9.336763865 0 0 38.86067476 5.288273488 17.36574174 12.16793341 17.53874084 20.0708278 11.79444444 7.362058277 0 0 0 18.52822581 18.67725015 9.086666667 5.59 0 0 20.29486863 0 22.69761722

1017 11.69917642 1.875293015 22.30572061 0 13.82443025 0 9.269817138 0 0 5.727884458 16.80116719 12.0747296 0 0 0 0 29.8377109 0 0 0 0 11.95590957 0 9.151488227 0 33.33333333 0 33.33333333 0 11.024 0 33.33333333 17.06854073 0 0 0 7.362007168 30.78452253 0 0 12.59639081 20.14703803 33.67285893 0 27.81730786 0 12.39892183 15.87363242 0 18.60176503 0 0 0 15.36050157 11.42044845 0 18.30847035 8.663842896 0 0 0 0 10.87333333 18.89092816 42.84071305 7.770754319 0

1018 0 0 8.3700722 13.96259075 27.68751528 26.09778477 0 22.12920726 16.76145868 0 0 0 0 35.24399442 0 23.3729485 0 0 0 0 14.36481003 19.08498667 7.917624262 0 0 9.735992296 0 0 16.40421316 0 37.66369048 0 0 0 20.63955951 0 0 11.9908046 0 12.63751006 15.13278646 0 15.22335423 21.77891056 9.70268725 0 15.76511226 0 0 3.150150595 0 0 8.98489978 0 0 0 0 27.10254093 0 4.014140723 0 7.666666667 26.21666667 13.77206004 0 10.45759416 0

1019 0 0 10.84834835 0 0 0 6.909926194 0 21.82684225 9.215712926 0 3.884950141 17.48169347 6.460746461 17.78453568 12.94617332 0 11.72043011 0 20.2292076 0 7.194919535 0 0 0 0 17.44499792 11.20580175 19.68326519 0 6.571229745 0 0 8.609087756 0 0 4.808624031 0 4.181069959 8.02946593 0 16.15022062 0 0 0 17.54301335 17.07796194 0 19.59828422 0 15.51467367 0 0 0 9.175449473 18.34293141 11.22937505 0 0 5.368610326 3.263333333 10.57 0 11.09056352 0 0 35.86737038

1020 21.24944422 22.00729745 13.23072476 0 0 15.0275816 0 11.1599414 0 2.694002033 0 8.375116063 0 0 3.353624169 0 0 11.34325969 0 0 16.48042005 8.875484705 8.812035952 8.728384019 0 0 17.32052309 21.08246807 0 22.09287394 0 16.4265636 0 0 38.35176895 31.2981477 0 7.965941343 8.811829168 0 0 0 0 20.55968401 4.868162634 0 0 38.83721855 0 0 14.89588186 49.92423545 12.63393727 0 11.61831947 0 0 16.34366925 28.93722312 0 0 0 0 0 26.84153791 23.80427781 0

1021 0 0 0 42.34739677 0 0 0 0 33.33333333 24.33064596 8.74212636 0 0 0 14.89821098 6.500277316 30.06222918 4.600227976 13.59275462 28.0566972 7.861034458 0 5.212867077 42.44028652 37.97182735 8.639724501 0 0 0 0 0 0 0 8.109289617 0 0 34.8892064 4.325747602 0 28.90271185 34.14916516 21.62009596 17.11499353 14.11574161 0 21.8451334 0 10.63179996 0 25.1057758 0 0 0 19.07205913 0 17.60811328 0 0 28.24427481 26.97733165 11.49666667 12.66333333 0 0 0 0 0

1022 0 0 60.26228981 0 12.04481793 19.98553271 46.94062079 16.92434159 11.02559813 0 0 0 30.2058179 12.03065134 6.949611092 0 0 0 29.81858317 0 0 25.88799842 0 11.19558736 33.33333333 26.51845971 25.05938242 18.69057321 100 30.72687902 27.11446018 19.65495002 21.76844784 23.94214162 0 14.80888889 0 0 10.22733157 13.9501349 0 16.30404086 33.33333333 0 27.20394 0 0 0 2.654359616 0 26.81806911 19.1035816 0 36.52667791 0 32.8076337 16.00654664 59.9767712 0 0 6.233333333 0 20.00666667 29.92441215 36.64208367 10.468078 22.55277888

1023 40.10944332 18.0009704 0 0 58.66565867 18.30575173 0 0 33.33333333 30.0855687 66.66666667 33.33333333 12.52930895 33.33333333 0 0 18.83559024 8.045148895 0 8.896396396 27.31194233 0 26.8972346 0 0 24.77420137 0 0 0 0 42.70219151 33.33333333 28.73673619 0 33.33333333 28.37513917 33.33333333 44.6022575 33.33333333 9.120476092 7.001566784 0 0 15.99944398 11.88834566 22.25244283 29.5004843 0 26.90862686 15.46984426 4.047942108 0 17.70888536 0 43.678421 0 12.29242608 0 0 33.33333333 15.44 26.12333333 0 19.91949328 0 11.71425214 33.33333333

1024 0 0 0 0 0 10.65980518 0 22.17339193 0 0 24.59120697 40.87622565 0 0 33.56688646 48.28064337 0 51.15616349 0 0 0 33.33333333 0 0 0 0 33.33333333 26.09588338 0 0 0 0 13.81152373 0 0 0 0 0 0 0 10.71582579 0 12.07919662 0 33.33333333 7.437331676 33.33333333 0 0 16.52357283 0 8.941279197 20.0171545 8.389789913 21.41824752 0 0 0 0 0 5.66 0 15.25 0 6.491795425 0 7.694326869

1025 0 44.65936922 0 40.22803884 6.171500858 0 17.86283892 0 22.30773521 17.75132275 0 0 0 21.30268199 23.03009807 0 5.512474713 0 43.21167437 28.52483784 13.37295691 24.45784863 24.52129738 16.37803434 0 9.308214322 0 14.64276012 0 0 0 6.494495198 11.5648855 29.67080221 0 10.74669392 0 0 29.28260236 6.940180482 0 23.78823997 16.2183398 19.21759173 0 0 33.33333333 22.70153337 18.02774301 4.502106897 0 0 10.47145919 0 0 0 0 0 0 8.056250745 0 0 0 19.13017032 6.014009909 0 0

1026 0 15.33236293 0 5.071539878 0 0 0 19.28787879 0 0 0 0 19.98434136 0 0 33.33333333 20.82561078 0 2.868724665 0 0 7.445334916 28.12046626 0 13.55123618 6.814873627 16.01281025 12.25086526 0 39.54519572 8.432040673 0 0 0 14.99679958 0 0 7.096733747 0 13.31581974 20.13028514 0 0 12.77364933 6.129393329 21.46679257 0 10.85903645 20.78209383 17.86348908 8.406829489 0 15.62444798 23.06164775 11.3699262 21.45068554 50.66012002 23.67955955 42.81850207 26.47279349 5.91 13.00333333 0 0 0 0 0

1027 21.24414227 0 26.50698543 0 0 13.34780063 35.1965403 0 0 0 0 0 16.47650741 33.33333333 18.20156922 0 10.26635199 10.9102752 0 9.428746929 22.87430429 0 6.436098734 0 0 8.559131963 0 7.237449954 0 0 0 0 0 9.39119171 13.31809814 0 15.85206572 10.77262693 0 0 13.4810263 38.2876232 0 17.33388935 0 26.99829952 0 0 0 11.36466335 33.22951352 22.03090375 13.31617884 5.871484295 11.91508582 28.13356749 21.04090725 0 0 0 0 0 10.67 13.41384005 0 33.12734252 10.78055445

1028 17.39697019 0 0 0 15.11701455 22.67352815 0 30.45444629 0 25.13846055 0 17.41532495 20.80402439 0 0 0 14.4977431 13.94492475 0 11.7122763 0 0 0 21.25770777 0 15.38539451 8.273950911 0 0 0 15.54221033 24.09065785 4.596597146 14.79367867 0 8.485925926 15.92539455 25.23669288 0 0 0 0 0 0 0 0 0 16.97041166 0 9.170547793 0 0 0 7.078341014 0 0 0 0 0 5.160290788 1.926666667 0 0 17.6120842 0 20.88604952 0

1029 0 0 0 12.35302451 8.001008001 0 0 0 0 0 0 0 0 0 0 11.88574598 0 0 10.50826317 13.38104533 12.09934197 0 0 0 15.14360313 0 0 0 0 7.635051314 6.209097301 0 19.52180961 14.09289617 0 6.285204395 0 0 18.34490357 27.77067694 14.52213083 0 21.25413671 0 16.57682504 0 3.832849038 0 31.62717669 0 12.60176391 0 10.22793688 0 0 0 0 0 0 0 0 8.316666667 0 0 24.01057309 0 25.63900646

1030 13.89375365 0 13.63208143 28.87655185 11.68455822 0 0 5.951383068 4.865771812 20.26349539 0 0 15.38108988 34.25717333 33.97735297 0 11.96544276 0 0 0 13.94767644 12.64824967 0 0 0 0 0 7.966929726 3.739283132 6.750298686 0 0 12.61118798 0 26.05075286 0 0 6.284153005 0 0 0 0 12.12460064 8.977879481 37.40347753 0 0 4.573754789 0 0 11.35508415 0 0 16.13599568 25.01760331 15.212221 15.65907321 9.989782284 0 21.86576408 11.50666667 0 0 13.84424519 13.19277869 0 21.5740358

1031 36.31329953 9.357094708 0 0 0 10.72733311 4.186444302 0 20.90243228 33.33333333 21.2724901 52.53918641 15.04113588 0 0 41.17300768 0 8.594708683 13.88413242 22.30314023 0 39.82862477 12.19883613 35.13445954 13.12830688 13.89027018 0 18.56006741 9.822326517 16.59171598 31.8443255 12.84186298 12.92306179 0 0 6.945182038 0 14.23286296 33.33333333 0 0 9.582322006 6.599222519 15.55555556 0 8.175273865 0 8.36627141 0 0 15.14342895 28.9652972 0 37.87963612 0 17.56355103 10.45222018 0 12.49170904 0 9.656666667 10.11333333 18.95666667 6.483143826 0 31.93284118 0

1032 0 11.15702479 17.8106047 19.41360041 9.708910188 8.166533227 0 0 0 0 0 0 0 0 0 0 14.85233887 10.78604621 10.00771605 0 12.67808733 0 7.668446479 0 14.23487544 0 47.4455571 4.28030303 0 0 13.64834719 20.51682975 0 19.67871486 0 25.69028609 33.33333333 0 29.3437899 0 32.02853486 19.15551916 0 6.514111365 0 0 0 22.72389446 14.84832358 14.2295082 0 15.836989 66.66666667 0 8.941627712 0 19.54550087 6.847027388 19.1825565 0 0 0 0 15.28906158 0 13.51739152 0

1033 0 16.295306 11.6163846 0 0 11.0885484 0 27.38195027 11.54362416 0 0 0 18.03511261 25.94135673 0 0 22.00528202 7.406862023 33.33333333 15.54343592 15.14245178 0 33.33333333 0 0 11.85177658 0 12.76775648 29.5940502 3.745519713 0 0 20.72214535 14.57570208 17.96067608 0 0 7.172995781 0 0 14.28272251 0 0 0 0 0 26.16248231 0 0 55.03526735 14.26940639 0 0 0 0 0 0 23.34355105 0 33.33333333 0 0 5.853333333 0 11.71523851 0 4.993983153

1034 0 0 0 0 8.699048431 0 27.87830406 33.33333333 0 23.3369843 0 0 8.066364178 0 0 0 0 24.96378023 0 7.730448501 0 12.89189867 0 0 0 33.33333333 24.73397674 14.77326592 23.51100682 0 0 0 0 0 0 0 0 0 0 0 0 20.36550668 12.08805044 0 0 0 0 0 0 0 18.18990438 0 20.15579995 0 0 0 9.441375077 26.48630595 0 20.7835808 8.126666667 0 5.063333333 0 33.33499909 14.27232979 0

1035 0 12.48053659 41.41820064 41.04881847 21.64877512 18.30582083 62.48022236 0 33.33333333 0 33.33333333 13.06228924 25.60209546 6.974242137 33.33333333 35.08556942 21.36789057 0 0 24.13213886 0 26.83804189 0 40.03400718 20.20502646 12.22443229 12.74723677 29.0530303 0 16.74161736 12.44883041 11.40671541 53.74360488 65.74558306 0 26.3881513 42.88597792 0 0 0 0 25.97999864 15.92897581 39.29604957 26.07292675 33.33333333 14.29407034 39.36901741 0 19.10382514 0 0 0 18.80687563 33.33333333 37.60765093 12.67036604 26.04073076 34.99240113 24.01732179 5.69 15.89666667 0 0 0 0 22.31552163

1036 38.64271208 0 0 0 23.62442315 0 0 33.33333333 12.43090105 13.06983794 18.60252005 0 0 19.51268627 24.31052581 0 18.48099447 0 23.32561728 17.78989742 0 0 0 24.83153328 0 0 0 0 0 56.17084827 19.68498614 46.14983692 0 0 30.02080866 33.33333333 0 26.16033755 13.28835032 66.66666667 18.73484598 0 0 0 4.904632153 58.4913928 33.33333333 24.96706192 66.66666667 11.63139932 41.04217612 38.12139614 0 17.19733765 15.70178593 23.99089098 0 0 23.31123737 0 1.52 5.183333333 0 46.33927765 7.805325987 20.46149569 51.11645942

1037 11.15023474 23.97623862 0 0 24.6342849 22.60600022 0 0 16.92393736 9.996349032 0 6.280193237 9.88587928 13.31454154 8.378787879 23.7414229 0 25.70131573 19.44920091 0 40.0409029 0 25.66488685 0 52.43179122 0 0 12.59864713 33.33333333 0 0 0 0 0 25.96776241 7.643047239 0 19.10047038 9.538978851 33.33333333 0 0 17.05005325 9.96948894 13.87944709 0 10.24901144 0 18.48500976 0 0 17.07631766 13.17753339 9.98015492 0 5.625686059 27.22757055 0 0 0 19.07666667 0 19.12666667 18.04427175 20.13888889 19.81594181 0

1038 0 26.73379928 15.52272864 10.66102928 0 29.10576421 5.455029271 0 0 0 0 28.11833111 7.988322718 0 0 0 11.32805131 22.54728713 0 12.50093907 18.19088155 7.793184994 21.1344972 0 0 28.70018762 15.0732294 0 0 0 22.37351075 9.084754942 0 0 0 0 0 27.04918033 14.4955476 0 22.60485651 24.91665352 36.20909735 19.68691509 7.260406583 0 0 0 0 0 0 0 0 0 0 0 5.003894081 7.292602576 10.02209596 0 2.44 4.716666667 0 0 0 0 0

1039 0 0 0 0 0 0 0 0 0 0 26.79165652 0 0 0 0 0 0 0 0 0 0 0 0 0 0 0 0 0 0 0 0 0 0 0 0 0 23.78068875 0 0 0 12.34904014 0 0 0 10.4791099 0 15.96110258 0 0 0 0 0 0 0 17.00564972 0 0 0 0 0 0 0 0 0 13.81276884 0 0

1040 0 0 0 0 0 0 0 13.16552968 14.27243863 0 0 28.37422146 0 38.52106646 0 0 0 0 0 0 0 0 0 0 0 17.91892072 0 55.1454357 0 0 17.26937269 0 6.293865646 41.90417864 20.62375844 35.91975582 29.71579375 0 0 0 0 0 0 13.42826807 0 0 0 0 0 0 25.49237611 0 0 12.61030458 0 0 0 0 0 56.36753445 0 6.04 8.48 0 0 0 0

1041 73.75269142 68.9273613 68.76295663 76.65678933 41.40838511 50.17201257 80.6514504 48.36513156 58.11929224 76.34473105 58.37109684 18.50553506 80.38339868 28.25259516 13.65918803 62.13047685 70.65133905 75.73082808 86.92361 77.96085927 84.43850101 53.19365982 82.71521379 44.55413085 81.44155325 22.73272758 46.08311362 24.08582653 39.83049768 89.63092142 55.43850734 62.3735083 26.1981685 27.14075321 34.75774242 0 22.11186156 50.96886325 71.36603999 80.82421231 38.91571049 27.17425336 78.05390049 41.25082515 33.33333333 78.29648158 16.92266236 74.43838375 55.66059383 65.87822525 41.74955465 58.27755926 91.63029548 26.88106421 49.83801102 56.0019362 66.62817964 87.20545366 87.29276656 0 45.26333333 14.96666667 18.78666667 69.91135339 55.60121766 54.97249611 21.21700335

1042 0 0 0 0 33.33333333 0 0 0 0 0 0 0 0 0 50.0952277 0 0 0 0 0 0 0 0 0 0 0 0 0 17.18996416 0 0 0 0 0 0 0 0 0 0 0 0 33.33333333 0 0 23.5969594 0 0 0 0 0 0 0 0 0 0 0 0 0 0 0 0 20.74333333 0 0 0 0 19.57486796

1043 0 15.55229896 0 0 0 11.72644163 0 16.18834784 12.36996309 0 0 27.47824847 0 7.688132248 0 0 0 0 0 0 0 0 0 22.90156117 0 12.38303561 16.25301999 0 0 0 0 0 15.94478062 7.413980085 0 21.08007449 0 0 0 0 26.93854107 0 0 19.90506527 33.33333333 0 57.56041704 0 13.07501769 0 0 0 0 12.28294905 0 33.33333333 0 0 0 8.911866076 0 0 11.45 0 19.64253312 0 33.33333333

1044 0 0 12.75241041 0 21.23444389 14.87263763 0 13.44303999 8.547374429 5.705525562 10.2622246 0 0 0 0 9.758868367 0 11.79344751 0 4.528985507 7.335257335 15.35946137 10.53930643 15.32835356 0 15.41441261 0 9.2475068 0 0 9.335793358 0 0 0 8.529722125 25.28923541 0 21.91632452 8.49477035 6.76662822 17.81852374 33.43911673 0 10.92691505 0 7.119095143 8.730800323 0 10.29377747 14.0714547 0 0 0 8.492093139 0 0 0 0 0 10.73926423 0 0 11.51333333 0 0 0 9.311356219

1045 12.37906227 0 14.47638604 0 0 0 6.114481915 0 0 0 7.434976044 0 0 20.45746796 0 12.48807821 4.552250551 0 4.781704782 0 0 0 0 0 0 0 0 0 0 0 0 0 0 0 13.98750744 0 33.33333333 6.915180983 8.019118428 0 0 0 2.712245225 0 0 0 0 9.141295863 11.82340648 6.815776816 0 6.867179255 0 7.657581401 2.354931043 0 7.850707851 7.716962525 0 0 2.416666667 3.31 0 0 0 7.723653689 0

1046 5.133980134 0 0 0 0 0 4.595157511 0 0 0 0 21.87957406 6.930221481 5.080738178 18.26404894 0 11.74557628 0 0 13.61947998 0 3.339223415 4.480759093 6.784182257 0 0 14.44801415 2.420500759 0 0 0 37.6264917 32.89578937 0 0 0 0 0 0 0 0 0 0 5.342169326 0 0 9.106249623 0 9.147204529 5.860805861 0 0 6.582052995 8.440079704 5.363003807 0 0 0 5.215931348 6.837438424 6.33 0 0 22.38005441 4.710806697 8.206391195 0

1047 8.734266181 13.15611155 0 0 0 8.778715424 0 0 0 4.442444844 0 0 0 0 5.26251823 10.97584541 0 12.4757244 0 0 2.106324908 4.093567251 2.264720684 10.43177216 6.255991121 20.95029773 23.21585224 0 0 1.969857218 11.22815932 0 0 12.42347828 5.563372025 17.71093428 3.617539586 0 9.513415189 12.40915947 0 6.053296577 0 0 0 10.02237948 0 11.27590197 0 0 7.840957222 0 0 0 0 10.66473046 11.55714855 0 7.491302095 0 7.576666667 0 3.583333333 0 0 0 0

1048 0 0 0 0 4.023837667 0 5.709985419 8.837950937 0 0 0 0 12.68637984 0 0 0 0 0 0 3.890675241 6.119916747 10.17624938 0 0 12.30245563 10.60060575 0 5.827664399 14.82963704 0 6.728167282 0 0 11.11760979 0 0 0 0 2.606656045 0 16.3272247 0 13.37229653 0 9.736373929 0 7.679870655 5.144418423 0 0 10.07059128 34.85526149 0 11.85034705 0 0 0 0 0 3.685830186 3.606666667 0 3.93 0 13.69080022 0 0

1049 0 2.364228199 4.008246923 23.34321067 0 14.45019274 2.928924759 0 6.690931619 13.50729854 23.93170251 3.762420958 0 0 12.71901709 4.646731164 13.05083412 0 8.294685218 0 0 13.83783877 0 0 0 0 0 3.273065804 28.14990112 8.399221366 0 0 18.66739586 0 16.53789756 0 11.22147177 20.19963125 0 0 0 0 5.86155776 9.146757135 0 4.562043796 0 0 0 7.373737374 14.84652073 0 1.787651526 11.78558087 42.44405412 0 13.96396396 5.077583818 0 13.45806663 0 7.066666667 0 7.708592204 6.354642314 29.09745901 16.56343914

1050 0 0 6.826069055 0 0 10.17269143 0 18.36993655 12.16737762 23.8954113 0 0 20.02731778 6.181266591 5.945794915 9.41292227 0 21.07066451 7.316859663 0 7.943106329 0 20.80811473 16.92366225 0 11.89143816 0 11.22160072 0 0 6.341350754 0 0 5.84706066 25.08564577 21.00626151 13.14379085 11.07324245 21.96893367 11.31890184 0 7.507275205 4.780275075 9.751797098 0 12.63597514 14.38524097 9.201491073 9.376641569 0 0 0 0 0 0 0 0 10.84735346 0 0 15.16666667 0 8.616666667 4.220531157 0 0 13.71914894

1051 13.01084237 2.883447711 0 0 22.94493189 20.66115702 21.59521943 7.042317887 11.69493863 0 0 2.688286751 10.65085158 15.60943208 13.86898857 0 7.565221056 11.05006738 0 5.238095238 6.958877343 0 10.57283699 0 17.47936984 0 22.88473553 0 0 0 6.192257281 13.09888846 12.16435948 0 0 0 0 0 24.04234573 13.59485264 13.44695951 0 0 0 5.793090725 0 4.036564395 0 48.23183289 0 0 0 0 6.772795398 4.545705606 5.347091932 0 0 4.348175858 0 8.84 0 3.17 6.185707473 0 0 0

1052 51.16959064 5.130682864 0 19.03940161 0 0 17.50293825 0 0 0 9.128300843 21.93998988 11.87800963 5.104285173 0 2.858823529 9.625112918 0 0 28.40543246 0 4.38224484 0 0 0 17.84335561 8.295016488 0 37.3706673 8.878199418 11.05790646 21.02866987 13.117506 9.66885889 0 0 19.77908827 0 0 27.38014855 0 18.00177559 16.41821682 11.45964032 0 9.306637004 0 0 0 26.89810404 0 16.27662942 4.833368562 21.43130501 0 18.56370231 16.12335714 14.501495 0 18.25302123 4.55 0 7.71 4.892882247 26.08333678 10.28439153 7.772435897

1053 0 9.167191106 11.69289202 0 18.11854269 0 0 25.06186457 24.11928777 16.93987888 29.77224473 0 0 24.14539161 0 12.46050988 7.686494176 10.38472339 5.420599309 11.67195767 0 0 0 9.979397 0 7.517254244 0 4.922970996 0 25.40118469 0 0 0 13.57954545 20.25538707 18.76530364 0 6.899893077 25.15737755 31.2964842 12.03990701 13.7715713 0 0 0 0 10.30213887 8.772520626 7.701511703 10.34007353 13.98921124 0 16.09195402 22.26742609 0 0 9.116898511 25.34131002 44.5724965 0 0 9.986666667 3.833333333 29.57825489 0 8.513800425 33.33333333

1054 0 4.882201362 10.60525851 0 0 6.380934076 0 0 0 0 0 0 0 0 12.70187203 3.531212419 4.783579888 0 36.7349916 0 14.92439989 42.42591656 33.65869331 11.63372635 0 16.11150765 4.048986756 17.46351509 0 0 0 17.40183359 0 9.42540916 8.247687564 14.89444574 0 0 11.36439966 0 0 0 0 33.33333333 9.834400081 12.94695814 0 10.23602923 0 15.51992875 18.13557412 0 0 0 8.509023722 4.252657911 0 17.69608973 0 0 0 2.72 0 0 27.83450704 0 0

1055 0 12.4177469 0 20.15143052 0 10.54472764 15.83039508 0 0 0 5.741047975 0 0 17.72390125 12.96954948 0 0 0 0 10.93422706 0 0 7.864774917 0 0 0 0 0 0 4.198586614 7.423537563 0 0 0 26.39798244 0 0 11.39395589 9.290987603 0 0 10.99200648 8.808565358 0 22.12539337 11.40572391 0 0 0 24.24863388 0 35.39313597 23.20739194 0 7.252140293 1.146131805 0 0 8.531434777 22.92262005 0 13.09 0 0 0 0 0

1056 0 8.294524858 21.83636719 11.594541 0 0 8.58277122 0 15.26226268 0 0 30.64504658 22.68248175 22.04778157 4.544619567 4.803921569 8.739837398 27.07946274 5.534065707 0 35.09982589 28.95108849 0 0 0 15.48997773 22.09733322 0 15.45722714 6.290601992 8.353313513 4.276862144 10.63502527 6.098484848 0 15.74837787 16.36660224 33.33333333 0 0 4.210254423 9.275658492 11.32456242 14.56214115 9.054010424 0 27.45786318 0 0 0 0 0 14.92730149 18.61643991 0 3.27198364 14.20741172 15.65109805 0 7.082087836 0 5.02 14.42 7.343907582 10.15441423 7.72707231 5.056980057

1057 15.49707602 0 0 0 0 6.291242232 0 0 0 0 0 0 0 0 0 0 0 0 17.6012035 0 0 0 0 0 11.48917957 0 0 44.28018058 19.60633455 0 0 0 0 0 0 11.14672365 17.09095255 0 0 5.953184785 26.78484532 0 0 0 0 0 0 0 0 0 0 0 0 0 0 0 0 0 0 0 0 0 0 0 0 0 0

1058 20.32249096 0 0 0 0 45.9492476 0 0 0 0 19.0647482 0 14.77211927 0 0 0 0 0 0 0 0 18.01038062 0 0 0 31.14646661 0 0 0 0 0 15.93149974 20.21582734 41.72533796 0 18.43888759 0 0 8.175955781 0 26.43486183 0 0 0 11.20793997 0 25.42286269 0 18.43483377 22.9932598 19.3441221 0 0 0 0 0 0 7.992023316 0 10.41071329 0 0 0 14.55962916 12.34398782 33.33333333 0

1059 0 57.2242052 49.03941321 49.21462687 58.93652542 0 36.48867602 49.52588099 36.75613331 59.16470982 36.29365825 44.72667679 19.98921999 9.187941728 49.96917543 66.93261033 61.59975456 30.41508198 27.39228022 43.75028756 35.07379055 6.230369482 27.09558005 61.4632144 71.03145058 0 42.67392801 22.11173262 27.56577101 55.23142729 60.63163443 28.26224619 43.86728192 13.65530303 20.01329715 0 33.61956608 37.29957525 0 10.45642799 17.0831719 40.45171293 58.66838032 30.89308811 41.98516544 53.70470581 18.39532989 71.78995907 16.25518006 0 48.53109255 48.33023461 40.93998399 30.91203359 79.69313038 67.4184324 60.55233263 7.970630426 42.54789287 41.3315576 49.87666667 17.05 17.5 33.21908749 23.58375412 40.1414024 40.11810178

1060 24.41332485 0 0 0 0 27.21830719 18.29703458 0 0 0 0 0 0 14.64725256 0 0 0 21.58695539 0 0 0 0 8.986801242 0 0 8.242727477 0 0 13.42557007 0 0 0 0 0 12.51428228 35.02145256 0 0 46.28457815 26.98489752 0 0 0 0 0 0 0 0 17.87533875 30.94098483 0 0 0 14.08199643 0 0 0 0 23.98997808 0 0 11.15333333 0 0 0 0 0

1061 0 0 0 0 0 11.17819621 0 26.14252725 0 0 0 0 14.57917951 0 0 0 0 0 0 5.437305218 0 6.686720562 0 33.33333333 0 23.40536772 0 41.48669011 0 0 0 0 16.95075758 0 6.6923359 0 11.05559026 0 11.46448847 0 0 0 0 10.29619182 12.798737 0 10.53784163 0 0 8.198151055 0 18.05958599 18.79615579 0 0 0 12.35908142 14.6819283 0 0 0 0 0 10.12489762 20.64969676 7.631275245 29.11807591

1062 25.30405939 0 0 0 19.10955478 10.43649649 30.769747 27.74891775 15.66815381 25.72184793 0 21.46460043 35.52548013 10.39298335 0 0 0 0 33.33333333 0 0 0 0 0 33.84570341 10.36474164 37.28016886 0 0 0 0 26.72231008 0 0 25.43428528 7.772149384 3.686908517 24.61165936 0 20.50601818 10.65891473 12.36707365 26.72520025 14.95827425 0 0 0 0 13.90479827 0 0 0 0 0 0 0 0 33.33333333 0 35.89321644 2.83 5.52 12.8 0 0 0 0

1063 0 0 0 51.6988312 0 0 0 0 9.46896993 61.88984599 0 0 0 33.33333333 66.66666667 0 33.33333333 0 0 0 0 0 0 0 0 0 0 33.33333333 0 13.09394269 0 0 27.43358396 6.109886071 0 0 0 21.70513775 0 0 24.2786696 0 0 0 0 0 0 0 0 0 0 0 0 14.07059585 0 0 0 0 0 0 16.34 3.45 0 33.33333333 31.88733008 14.98936924 0

1064 20.10546393 29.20897029 38.32521645 0 0 0 4.352774702 0 10.70116793 0 53.56074207 13.90961857 0 0 0 7.923705169 15.75808018 0 0 31.61170225 29.47346339 0 21.33722547 0 6.04483309 0 11.065852 25.17997656 0 16.63814007 24.45128205 0 0 0 0 0 18.05121799 5.153445281 36.73326825 0 0 10.66648011 35.41872472 0 7.311230878 53.45772249 0 24.44128193 0 0 11.02723325 0 24.14747704 0 0 33.33333333 33.33333333 0 9.343355256 0 0 3.483333333 8.393333333 0 0 44.68166955 13.91748263

1065 8.920008487 10.15948963 0 33.33333333 8.204102051 17.90828191 0 0 17.66517952 0 0 0 0 9.85728318 0 33.33333333 17.5971538 25.20030805 26.34666667 21.31324004 0 31.70976346 0 25.6344086 0 13.84906508 0 0 32.7745267 19.06516399 0 41.53330559 5.781776094 33.33333333 16.88668867 47.49741911 0 0 0 18.2174336 20.17926357 26.00616469 6.608133087 38.94275479 33.33333333 19.49513382 45.88881103 0 4.504065041 34.60775678 14.51539855 18.3583512 15.69562147 19.2513369 33.33333333 11.60714286 19.07298383 38.84854847 20.03058104 0 20.90333333 10.01 0 49.02743653 9.588888889 0 0

1066 0 0 0 0 5.713072104 0 33.33333333 17.72072072 0 0 33.33333333 6.991492653 24.47608812 22.94034998 0 0 0 33.33333333 0 0 0 0 10.97955487 0 6.148934694 32.8965573 20.78291815 0 17.10687797 0 0 0 30.30677882 42.69592909 0 0 51.49842271 11.62819558 0 7.157946451 0 0 0 0 0 0 0 0 24.8372093 6.500607533 0 7.119365448 9.468892261 37.44572883 38.27292111 23.27433628 20.97425191 0 0 49.35283241 0 0 3.11 0 16.74104589 0 12.18245133

1067 0 0 0 14.96783546 17.17955827 6.115026142 0 4.081413458 0 7.611485403 13.1059246 11.86873291 25.41925224 0 0 25.40962816 0 0 33.33333333 0 33.33333333 33.33333333 45.3294412 0 32.82096326 0 0 0 0 20.23939064 58.00230757 17.94536472 5.899749373 0 0 0 0 36.90156203 5.517665131 0 0 20.96625968 15.21218011 23.93195689 33.33333333 13.20894417 33.33333333 42.22538474 0 0 22.30610009 33.33333333 17.63771186 0 0 21.72619048 0 0 0 6.079382184 5.373333333 0 8.203333333 0 10.57748283 25.48956902 0

1068 8.029273943 37.45769638 47.64880952 0 0 12.93832719 2.563586336 0 0 0 0 19.42371476 0 8.828797596 0 0 0 15.27847888 0 6.708037825 0 7.401598327 0 7.698924731 0 0 30.87106099 0 20.4665699 16.69519327 0 0 0 0 38.47240787 9.708978943 4.226525088 0 0 21.46433555 42.38799706 0 16.03576184 11.87082226 13.22336545 0 0 33.33333333 19.42853506 0 0 0 0 0 28.39374556 0 0 0 33.33333333 0 0 4.306666667 0 7.514332514 10.55555556 0 11.44865679

1069 13.2278694 23.1738437 14.02597403 0 49.7937128 14.20536487 10.68352406 24.30642082 46.49652881 4.776820673 0 26.34184068 0 0 33.33333333 0 33.31143269 4.600924339 6.986666667 34.92971467 37.19320327 20.86858432 13.36697723 33.33333333 21.13956555 11.24154078 0 0 16.22645537 14.26816934 17.54641038 13.79901961 13.62735417 17.86085151 0 0 11.48133544 0 0 5.669368705 2.495155039 29.99402187 0 0 0 13.83819951 10.24001402 0 19.45005357 19.75249981 18.81793478 23.12936402 14.25414158 15.15034198 0 10.05899705 14.2603495 13.1361899 13.30275229 8.674568966 14.45666667 12 0 0 0 7.208116946 33.33333333
[truncated: 405,531 more chars]
