# Supplementary material for: Distinguishing and phenotype monitoring of traumatic brain injury and post-concussion syndrome including chronic migraine in serum of Iraq and Afghanistan war veterans
Source: PLoS One. 2019 Apr 26;14(4):e0215762. doi: 10.1371/journal.pone.0215762 (PMC6485717; doi:10.1371/journal.pone.0215762)
Supplement: S8 Table — (DOCX) [file pone.0215762.s034.docx]

**S8 Table. Patient % classified LOOCV mass peak scores plotted in presented figures**

| Fig 3 A,B | 1 | 2 | 3 | 4 | 5 | 6 | 7 | 8 | 9 | 10 | 11 | 12 | 13 | 14 | 15 | 16 | 17 | 18 | 19 | 20 | 21 |
| --- | --- | --- | --- | --- | --- | --- | --- | --- | --- | --- | --- | --- | --- | --- | --- | --- | --- | --- | --- | --- | --- |
| TBI (MA), N=21 | 63.3 | 62.04 | 60.71 | 70.09 | 54.7 | 53.1 | 54.39 | 60.18 | 59.46 | 54.7 | 56.9 | 60.53 | 54.05 | 55.86 | 62.96 | 56.03 | 53.57 | 55.96 | 54.55 | 60.18 | 58.18 |
| Controls, N=20 | 36.45 | 34.23 | 42.98 | 43.48 | 46.55 | 38.6 | 38.18 | 36.28 | 40.91 | 36.79 | 44.07 | 45.28 | 45.13 | 34.55 | 36.28 | 41.51 | 40.52 | 36.36 | 40.91 | 38.32 |  |
| RND: TBI (MA), N=21 | 41.59 | 51 | 37.25 | 41.12 | 45.13 | 41.03 | 50 | 51.4 | 43.64 | 45.37 | 46.3 | 43.1 | 36.75 | 50.96 | 50.94 | 55 | 55.05 | 59.26 | 41.28 | 42.34 | 51.69 |
| RND: Controls, N=20 | 43.88 | 48.21 | 43.69 | 57.39 | 46.79 | 50 | 46.67 | 45.61 | 46.67 | 42.86 | 47.12 | 37.5 | 46.02 | 46.08 | 57.39 | 50 | 47.62 | 43.81 | 41.9 | 50.94 |  |
|  |  |  |  |  |  |  |  |  |  |  |  |  |  |  |  |  |  |  |  |  |  |
| Fig 4 A,B,C | 1 | 2 | 3 | 4 | 5 | 6 | 7 | 8 | 9 | 10 | 11 | 12 | 13 | 14 | 15 | 16 |  |  |  |  |  |
| TS TBI, (MA), N=16 | 63.77 | 60.29 | 63.08 | 55.88 | 60 | 53.73 | 57.14 | 60.29 | 50.72 | 62.32 | 65.67 | 47.95 | 65.63 | 52.94 | 61.19 | 55.22 |  |  |  |  |  |
| TS Controls, N=15 | 34.33 | 32.84 | 37.14 | 30.88 | 35.82 | 34.78 | 39.13 | 34.78 | 42.86 | 39.06 | 38.03 | 43.08 | 37.14 | 40.58 | 28.79 |  |  |  |  |  |  |
| TS RND: TBI (MA), N=16 | 40 | 50 | 52.46 | 40.58 | 56.9 | 51.56 | 42.86 | 53.97 | 60 | 46.15 | 53.13 | 52.38 | 49.25 | 48.28 | 55 | 45 |  |  |  |  |  |
| TS RND: Controls, N=15 | 61.76 | 42.86 | 55.56 | 52.63 | 51.52 | 40.98 | 45.45 | 47.95 | 52.63 | 52.24 | 50 | 42.11 | 48.33 | 54.24 | 43.64 |  |  |  |  |  |  |
| Blinds TBI (MA), N=5 | 53.16 | 49.37 | 54.43 | 51.9 | 50.63 |  |  |  |  |  |  |  |  |  |  |  |  |  |  |  |  |
| Blinds Control, N=5 | 48.1 | 45.57 | 39.24 | 45.57 | 44.3 |  |  |  |  |  |  |  |  |  |  |  |  |  |  |  |  |
|  |  |  |  |  |  |  |  |  |  |  |  |  |  |  |  |  |  |  |  |  |  |
| Fig 4D | 1 | 2 | 3 | 4 | 5 | 6 | 7 | 8 | 9 | 10 | 11 | 12 | 13 | 14 | 15 |  |  |  |  |  |  |
| TS TBI (MA), N=15 | 63.01 | 49.33 | 60 | 53.33 | 57.53 | 59.21 | 59.46 | 68.06 | 66.67 | 56.16 | 72.97 | 60 | 68.42 | 53.52 | 64.38 |  |  |  |  |  |  |
| TS TBI, N=12 | 36.49 | 33.33 | 25.68 | 18.92 | 21.43 | 22.97 | 21.92 | 28.77 | 23.29 | 28.17 | 29.73 | 24.66 |  |  |  |  |  |  |  |  |  |
| TS RND: TBI (MA), N=15 | 52.7 | 52.17 | 46.58 | 49.25 | 65.08 | 46.05 | 56.06 | 58.73 | 39.47 | 45.21 | 42.86 | 59.09 | 37.88 | 52.17 | 57.38 |  |  |  |  |  |  |
| TS RND: TBI, N=12 | 32.73 | 43.48 | 57.35 | 55.22 | 39.39 | 49.25 | 50.77 | 45.45 | 43.33 | 38.71 | 54.79 | 45.16 |  |  |  |  |  |  |  |  |  |
| Blinds TBI (MA), N=6 | 53.49 | 48.84 | 45.35 | 41.86 | 44.19 | 46.51 |  |  |  |  |  |  |  |  |  |  |  |  |  |  |  |
|  |  |  |  |  |  |  |  |  |  |  |  |  |  |  |  |  |  |  |  |  |  |
| Fig 5A | 1 | 2 | 3 | 4 | 5 | 6 | 7 | 8 | 9 | 10 | 11 | 12 | 13 | 14 | 15 | 16 | 17 | 18 | 19 | 20 |  |
| TBI, N=12 | 72.95 | 69.67 | 72.13 | 71.19 | 67.5 | 71.43 | 77.24 | 82.95 | 70.87 | 78.86 | 72.36 | 70.4 |  |  |  |  |  |  |  |  |  |
| Controls, N=20 | 48.44 | 42.97 | 40.94 | 42.52 | 43.75 | 38.76 | 45.38 | 50.39 | 35.71 | 47.66 | 38.89 | 41.41 | 43.31 | 47.69 | 44.96 | 47.69 | 51.56 | 50.39 | 48.44 | 44.44 |  |
| RND: TBI, N=12 | 50.44 | 45.95 | 49.15 | 56.41 | 56.07 | 53.21 | 52.68 | 67.31 | 58.42 | 51.46 | 51.38 | 66.34 |  |  |  |  |  |  |  |  |  |
| RND: Controls, N=20 | 54.13 | 58.33 | 57.39 | 57.14 | 66.13 | 55.75 | 52.94 | 50.88 | 57.89 | 63.85 | 54.76 | 54.95 | 52.85 | 49.11 | 52.63 | 51.79 | 51.38 | 54.92 | 51.33 | 50.43 |  |
|  |  |  |  |  |  |  |  |  |  |  |  |  |  |  |  |  |  |  |  |  |  |
| Fig 5 B,C | 1 | 2 | 3 | 4 | 5 | 6 | 7 | 8 | 9 | 10 | 11 | 12 | 13 | 14 | 15 | 16 | 17 | 18 | 19 | 20 |  |
| TBI with CM, N=11 | 79.52 | 78.31 | 71.95 | 73.49 | 73.81 | 83.13 | 73.49 | 75.9 | 64.29 | 81.93 | 78.05 |  |  |  |  |  |  |  |  |  |  |
| Control, N=20 | 39.29 | 39.29 | 45.24 | 36.9 | 38.1 | 39.29 | 40.48 | 32.14 | 36.9 | 43.37 | 42.86 | 34.94 | 35.71 | 38.1 | 38.1 | 39.29 | 46.99 | 44.05 | 60.71 | 46.43 |  |
| RND: TBI with CM, N=11 | 56.52 | 50.68 | 58.82 | 63.38 | 51.39 | 44.05 | 66.67 | 66.67 | 62.34 | 55.07 | 60.61 |  |  |  |  |  |  |  |  |  |  |
| RND: Control, N=20 | 62.67 | 53.95 | 36.92 | 57.97 | 53.95 | 48.72 | 56.41 | 50.63 | 54.67 | 54.93 | 59.72 | 41.18 | 50.68 | 38.16 | 63.16 | 53.52 | 56.96 | 51.32 | 53.57 | 58.9 |  |
|  |  |  |  |  |  |  |  |  |  |  |  |  |  |  |  |  |  |  |  |  |  |
| Fig 5D | 1 | 2 | 3 | 4 | 5 | 6 | 7 | 8 | 9 | 10 | 11 | 12 |  |  |  |  |  |  |  |  |  |
| TBI with CM, N=11 | 60 | 66.3 | 63.44 | 68.13 | 66.67 | 61.29 | 70.79 | 69.15 | 69.66 | 75 | 71.11 |  |  |  |  |  |  |  |  |  |  |
| TBI, N=12 | 33.33 | 37.08 | 52.13 | 30.77 | 36.96 | 30.59 | 35.56 | 24.72 | 43.48 | 27.59 | 35.96 | 43.75 |  |  |  |  |  |  |  |  |  |
| RND: TBI with CM, N=11 | 45.98 | 51.19 | 45.56 | 47.19 | 37.36 | 38.3 | 47.13 | 44.79 | 39.77 | 40.86 | 45.26 |  |  |  |  |  |  |  |  |  |  |
| RND: TBI, N=12 | 40.23 | 38.75 | 40 | 35.63 | 43.82 | 49.47 | 43.33 | 38.37 | 50 | 49.43 | 45.88 | 46.07 |  |  |  |  |  |  |  |  |  |
