# Supplementary material for: Distinguishing and phenotype monitoring of traumatic brain injury and post-concussion syndrome including chronic migraine in serum of Iraq and Afghanistan war veterans
Source: PLoS One. 2019 Apr 26;14(4):e0215762. doi: 10.1371/journal.pone.0215762 (PMC6485717; doi:10.1371/journal.pone.0215762)
Supplement: S9 Table — (DOCX) [file pone.0215762.s035.docx]

**S9 Table. Samples used in each figure.**

| Panel order | figure | sample | group type: true pathology; random pathology; blind sample |
| --- | --- | --- | --- |
| 1 | fig3 AB | TBI 1 | TRUE |
| 2 | fig3 AB | TBI 5 | TRUE |
| 3 | fig3 AB | TBI 6 | TRUE |
| 4 | fig3 AB | TBI 8 | TRUE |
| 5 | fig3 AB | TBI 9 | TRUE |
| 6 | fig3 AB | TBI 10 | TRUE |
| 7 | fig3 AB | TBI 12 | TRUE |
| 8 | fig3 AB | TBI 2 | TRUE |
| 9 | fig3 AB | TBI 13 | TRUE |
| 10 | fig3 AB | TBI 16 | TRUE |
| 11 | fig3 AB | TBI 17 | TRUE |
| 12 | fig3 AB | TBI 18 | TRUE |
| 13 | fig3 AB | TBI 23 | TRUE |
| 14 | fig3 AB | TBI 26 | TRUE |
| 15 | fig3 AB | TBI 27 | TRUE |
| 16 | fig3 AB | TBI 30 | TRUE |
| 17 | fig3 AB | TBI 32 | TRUE |
| 18 | fig3 AB | TBI 33 | TRUE |
| 19 | fig3 AB | TBI 34 | TRUE |
| 20 | fig3 AB | TBI 36 | TRUE |
| 21 | fig3 AB | TBI 37 | TRUE |
| 1 | fig3 AB | control 1 | TRUE |
| 2 | fig3 AB | control 2 | TRUE |
| 3 | fig3 AB | control 3 | TRUE |
| 4 | fig3 AB | control 4 | TRUE |
| 5 | fig3 AB | control 5 | TRUE |
| 6 | fig3 AB | control 6 | TRUE |
| 7 | fig3 AB | control 7 | TRUE |
| 8 | fig3 AB | control 8 | TRUE |
| 9 | fig3 AB | control 9 | TRUE |
| 10 | fig3 AB | control 10 | TRUE |
| 11 | fig3 AB | control 11 | TRUE |
| 12 | fig3 AB | control 12 | TRUE |
| 13 | fig3 AB | control 13 | TRUE |
| 14 | fig3 AB | control 14 | TRUE |
| 15 | fig3 AB | control 15 | TRUE |
| 16 | fig3 AB | control 16 | TRUE |
| 17 | fig3 AB | control 17 | TRUE |
| 18 | fig3 AB | control 18 | TRUE |
| 19 | fig3 AB | control 19 | TRUE |
| 20 | fig3 AB | control 20 | TRUE |
| 1 | fig3 AB RND | TBI 1 | rnd |
| 2 | fig3 AB RND | TBI 6 | rnd |
| 3 | fig3 AB RND | TBI 9 | rnd |
| 4 | fig3 AB RND | TBI 12 | rnd |
| 5 | fig3 AB RND | TBI 13 | rnd |
| 6 | fig3 AB RND | TBI 17 | rnd |
| 7 | fig3 AB RND | TBI 23 | rnd |
| 8 | fig3 AB RND | TBI 27 | rnd |
| 9 | fig3 AB RND | TBI 32 | rnd |
| 10 | fig3 AB RND | TBI 34 | rnd |
| 11 | fig3 AB RND | TBI 37 | rnd |
| 12 | fig3 AB RND | control 2 | rnd |
| 13 | fig3 AB RND | control 4 | rnd |
| 14 | fig3 AB RND | control 6 | rnd |
| 15 | fig3 AB RND | control 8 | rnd |
| 16 | fig3 AB RND | control 10 | rnd |
| 17 | fig3 AB RND | control 12 | rnd |
| 18 | fig3 AB RND | control 14 | rnd |
| 19 | fig3 AB RND | control 16 | rnd |
| 20 | fig3 AB RND | control 18 | rnd |
| 21 | fig3 AB RND | control 20 | rnd |
| 1 | fig3 AB RND | TBI 5 | rnd |
| 2 | fig3 AB RND | TBI 8 | rnd |
| 3 | fig3 AB RND | TBI 10 | rnd |
| 4 | fig3 AB RND | TBI 2 | rnd |
| 5 | fig3 AB RND | TBI 16 | rnd |
| 6 | fig3 AB RND | TBI 18 | rnd |
| 7 | fig3 AB RND | TBI 26 | rnd |
| 8 | fig3 AB RND | TBI 30 | rnd |
| 9 | fig3 AB RND | TBI 33 | rnd |
| 10 | fig3 AB RND | TBI 36 | rnd |
| 11 | fig3 AB RND | control 1 | rnd |
| 12 | fig3 AB RND | control 3 | rnd |
| 13 | fig3 AB RND | control 5 | rnd |
| 14 | fig3 AB RND | control 7 | rnd |
| 15 | fig3 AB RND | control 9 | rnd |
| 16 | fig3 AB RND | control 11 | rnd |
| 17 | fig3 AB RND | control 13 | rnd |
| 18 | fig3 AB RND | control 15 | rnd |
| 19 | fig3 AB RND | control 17 | rnd |
| 20 | fig3 AB RND | control 19 | rnd |
| 1 | fig4 ABC blinds | TBI 10 | blinds |
| 2 | fig4 ABC blinds | TBI 16 | blinds |
| 3 | fig4 ABC blinds | TBI 26 | blinds |
| 4 | fig4 ABC blinds | TBI 17 | blinds |
| 5 | fig4 ABC blinds | TBI 6 | blinds |
| 1 | fig4 ABC blinds | control 13 | blinds |
| 2 | fig4 ABC blinds | control 12 | blinds |
| 3 | fig4 ABC blinds | control 14 | blinds |
| 4 | fig4 ABC blinds | control 1 | blinds |
| 5 | fig4 ABC blinds | control 5 | blinds |
| 1 | fig4 ABC | TBI 12 | TRUE |
| 2 | fig4 ABC | TBI 18 | TRUE |
| 3 | fig4 ABC | TBI 8 | TRUE |
| 4 | fig4 ABC | TBI 9 | TRUE |
| 5 | fig4 ABC | TBI 23 | TRUE |
| 6 | fig4 ABC | TBI 27 | TRUE |
| 7 | fig4 ABC | TBI 13 | TRUE |
| 8 | fig4 ABC | TBI 1 | TRUE |
| 9 | fig4 ABC | TBI 2 | TRUE |
| 10 | fig4 ABC | TBI 5 | TRUE |
| 11 | fig4 ABC | TBI 30 | TRUE |
| 12 | fig4 ABC | TBI 32 | TRUE |
| 13 | fig4 ABC | TBI 33 | TRUE |
| 14 | fig4 ABC | TBI 34 | TRUE |
| 15 | fig4 ABC | TBI 36 | TRUE |
| 16 | fig4 ABC | TBI 37 | TRUE |
| 1 | fig4 ABC | control 4 | TRUE |
| 2 | fig4 ABC | control 8 | TRUE |
| 3 | fig4 ABC | control 7 | TRUE |
| 4 | fig4 ABC | control 3 | TRUE |
| 5 | fig4 ABC | control 6 | TRUE |
| 6 | fig4 ABC | control 11 | TRUE |
| 7 | fig4 ABC | control 2 | TRUE |
| 8 | fig4 ABC | control 9 | TRUE |
| 9 | fig4 ABC | control 10 | TRUE |
| 10 | fig4 ABC | control 15 | TRUE |
| 11 | fig4 ABC | control 16 | TRUE |
| 12 | fig4 ABC | control 17 | TRUE |
| 13 | fig4 ABC | control 18 | TRUE |
| 14 | fig4 ABC | control 19 | TRUE |
| 15 | fig4 ABC | control 20 | TRUE |
| 1 | fig4 ABC RND | control 4 | rnd |
| 2 | fig4 ABC RND | control 7 | rnd |
| 3 | fig4 ABC RND | control 6 | rnd |
| 4 | fig4 ABC RND | control 2 | rnd |
| 5 | fig4 ABC RND | control 10 | rnd |
| 6 | fig4 ABC RND | control 16 | rnd |
| 7 | fig4 ABC RND | control 18 | rnd |
| 8 | fig4 ABC RND | control 20 | rnd |
| 9 | fig4 ABC RND | TBI 12 | rnd |
| 10 | fig4 ABC RND | TBI 8 | rnd |
| 11 | fig4 ABC RND | TBI 23 | rnd |
| 12 | fig4 ABC RND | TBI 13 | rnd |
| 13 | fig4 ABC RND | TBI 2 | rnd |
| 14 | fig4 ABC RND | TBI 30 | rnd |
| 15 | fig4 ABC RND | TBI 33 | rnd |
| 16 | fig4 ABC RND | TBI 36 | rnd |
| 1 | fig4 ABC RND | control 8 | rnd |
| 2 | fig4 ABC RND | control 3 | rnd |
| 3 | fig4 ABC RND | control 11 | rnd |
| 4 | fig4 ABC RND | control 9 | rnd |
| 5 | fig4 ABC RND | control 15 | rnd |
| 6 | fig4 ABC RND | control 17 | rnd |
| 7 | fig4 ABC RND | control 19 | rnd |
| 8 | fig4 ABC RND | TBI 18 | rnd |
| 9 | fig4 ABC RND | TBI 9 | rnd |
| 10 | fig4 ABC RND | TBI 27 | rnd |
| 11 | fig4 ABC RND | TBI 1 | rnd |
| 12 | fig4 ABC RND | TBI 5 | rnd |
| 13 | fig4 ABC RND | TBI 32 | rnd |
| 14 | fig4 ABC RND | TBI 34 | rnd |
| 15 | fig4 ABC RND | TBI 37 | rnd |
| 1 | fig4d blinds | TBI 30 | blinds |
| 2 | fig4d blinds | TBI 32 | blinds |
| 3 | fig4d blinds | TBI 33 | blinds |
| 4 | fig4d blinds | TBI 34 | blinds |
| 5 | fig4d blinds | TBI 36 | blinds |
| 6 | fig4d blinds | TBI 37 | blinds |
| 1 | fig4d | TBI 1 | TRUE |
| 2 | fig4d | TBI 5 | TRUE |
| 3 | fig4d | TBI 6 | TRUE |
| 4 | fig4d | TBI 8 | TRUE |
| 5 | fig4d | TBI 9 | TRUE |
| 6 | fig4d | TBI 10 | TRUE |
| 7 | fig4d | TBI 12 | TRUE |
| 8 | fig4d | TBI 2 | TRUE |
| 9 | fig4d | TBI 13 | TRUE |
| 10 | fig4d | TBI 16 | TRUE |
| 11 | fig4d | TBI 17 | TRUE |
| 12 | fig4d | TBI 18 | TRUE |
| 13 | fig4d | TBI 23 | TRUE |
| 14 | fig4d | TBI 26 | TRUE |
| 15 | fig4d | TBI 27 | TRUE |
| 1 | fig4d | TBI 41 | TRUE |
| 2 | fig4d | TBI 4 | TRUE |
| 3 | fig4d | TBI 29 | TRUE |
| 4 | fig4d | TBI 44 | TRUE |
| 5 | fig4d | TBI 45 | TRUE |
| 6 | fig4d | TBI 14 | TRUE |
| 7 | fig4d | TBI 21 | TRUE |
| 8 | fig4d | TBI 22 | TRUE |
| 9 | fig4d | TBI 25 | TRUE |
| 10 | fig4d | TBI 42 | TRUE |
| 11 | fig4d | TBI 20 | TRUE |
| 12 | fig4d | TBI 38 | TRUE |
| 1 | fig4d RND | TBI 1 | rnd |
| 2 | fig4d RND | TBI 6 | rnd |
| 3 | fig4d RND | TBI 9 | rnd |
| 4 | fig4d RND | TBI 12 | rnd |
| 5 | fig4d RND | TBI 13 | rnd |
| 6 | fig4d RND | TBI 17 | rnd |
| 7 | fig4d RND | TBI 23 | rnd |
| 8 | fig4d RND | TBI 27 | rnd |
| 9 | fig4d RND | TBI 4 | rnd |
| 10 | fig4d RND | TBI 44 | rnd |
| 11 | fig4d RND | TBI 14 | rnd |
| 12 | fig4d RND | TBI 22 | rnd |
| 13 | fig4d RND | TBI 42 | rnd |
| 14 | fig4d RND | TBI 38 | rnd |
| 15 | fig4d RND | TBI 20 | rnd |
| 1 | fig4d RND | TBI 5 | rnd |
| 2 | fig4d RND | TBI 8 | rnd |
| 3 | fig4d RND | TBI 10 | rnd |
| 4 | fig4d RND | TBI 2 | rnd |
| 5 | fig4d RND | TBI 16 | rnd |
| 6 | fig4d RND | TBI 18 | rnd |
| 7 | fig4d RND | TBI 26 | rnd |
| 8 | fig4d RND | TBI 41 | rnd |
| 9 | fig4d RND | TBI 29 | rnd |
| 10 | fig4d RND | TBI 45 | rnd |
| 11 | fig4d RND | TBI 21 | rnd |
| 12 | fig4d RND | TBI 25 | rnd |
| 1 | fig5A | TBI 20 | TRUE |
| 2 | fig5A | TBI 4 | TRUE |
| 3 | fig5A | TBI 41 | TRUE |
| 4 | fig5A | TBI 29 | TRUE |
| 5 | fig5A | TBI 44 | TRUE |
| 6 | fig5A | TBI 45 | TRUE |
| 7 | fig5A | TBI 14 | TRUE |
| 8 | fig5A | TBI 21 | TRUE |
| 9 | fig5A | TBI 11 | TRUE |
| 10 | fig5A | TBI 22 | TRUE |
| 11 | fig5A | TBI 25 | TRUE |
| 12 | fig5A | TBI 42 | TRUE |
| 1 | fig5A | control 1 | TRUE |
| 2 | fig5A | control 2 | TRUE |
| 3 | fig5A | control 3 | TRUE |
| 4 | fig5A | control 4 | TRUE |
| 5 | fig5A | control 5 | TRUE |
| 6 | fig5A | control 6 | TRUE |
| 7 | fig5A | control 7 | TRUE |
| 8 | fig5A | control 8 | TRUE |
| 9 | fig5A | control 9 | TRUE |
| 10 | fig5A | control 10 | TRUE |
| 11 | fig5A | control 11 | TRUE |
| 12 | fig5A | control 12 | TRUE |
| 13 | fig5A | control 13 | TRUE |
| 14 | fig5A | control 14 | TRUE |
| 15 | fig5A | control 15 | TRUE |
| 16 | fig5A | control 16 | TRUE |
| 17 | fig5A | control 17 | TRUE |
| 18 | fig5A | control 18 | TRUE |
| 19 | fig5A | control 19 | TRUE |
| 20 | fig5A | control 20 | TRUE |
| 1 | fig5a RND | TBI 20 | rnd |
| 2 | fig5a RND | TBI 41 | rnd |
| 3 | fig5a RND | TBI 44 | rnd |
| 4 | fig5a RND | TBI 14 | rnd |
| 5 | fig5a RND | TBI 11 | rnd |
| 6 | fig5a RND | TBI 25 | rnd |
| 7 | fig5a RND | control 1 | rnd |
| 8 | fig5a RND | control 3 | rnd |
| 9 | fig5a RND | control 5 | rnd |
| 10 | fig5a RND | control 7 | rnd |
| 11 | fig5a RND | control 9 | rnd |
| 12 | fig5a RND | control 11 | rnd |
| 1 | fig5a RND | control 13 | rnd |
| 2 | fig5a RND | control 17 | rnd |
| 3 | fig5a RND | control 15 | rnd |
| 4 | fig5a RND | control 19 | rnd |
| 5 | fig5a RND | TBI 4 | rnd |
| 6 | fig5a RND | TBI 29 | rnd |
| 7 | fig5a RND | TBI 45 | rnd |
| 8 | fig5a RND | TBI 21 | rnd |
| 9 | fig5a RND | TBI 22 | rnd |
| 10 | fig5a RND | TBI 42 | rnd |
| 11 | fig5a RND | control 2 | rnd |
| 12 | fig5a RND | control 4 | rnd |
| 13 | fig5a RND | control 6 | rnd |
| 14 | fig5a RND | control 8 | rnd |
| 15 | fig5a RND | control 10 | rnd |
| 16 | fig5a RND | control 12 | rnd |
| 17 | fig5a RND | control 14 | rnd |
| 18 | fig5a RND | control 16 | rnd |
| 19 | fig5a RND | control 18 | rnd |
| 20 | fig5a RND | control 20 | rnd |
| 1 | Fig5BC | TBI 3 | TRUE |
| 2 | Fig5BC | TBI 15 | TRUE |
| 3 | Fig5BC | TBI 31 | TRUE |
| 4 | Fig5BC | TBI 35 | TRUE |
| 5 | Fig5BC | TBI 40 | TRUE |
| 6 | Fig5BC | TBI 39 | TRUE |
| 7 | Fig5BC | TBI 43 | TRUE |
| 8 | Fig5BC | TBI 24 | TRUE |
| 9 | Fig5BC | TBI 7 | TRUE |
| 10 | Fig5BC | TBI 19 | TRUE |
| 11 | Fig5BC | TBI 28 | TRUE |
| 1 | Fig5BC | control 1 | TRUE |
| 2 | Fig5BC | control 2 | TRUE |
| 3 | Fig5BC | control 3 | TRUE |
| 4 | Fig5BC | control 4 | TRUE |
| 5 | Fig5BC | control 5 | TRUE |
| 6 | Fig5BC | control 6 | TRUE |
| 7 | Fig5BC | control 7 | TRUE |
| 8 | Fig5BC | control 8 | TRUE |
| 9 | Fig5BC | control 9 | TRUE |
| 10 | Fig5BC | control 10 | TRUE |
| 11 | Fig5BC | control 11 | TRUE |
| 12 | Fig5BC | control 12 | TRUE |
| 13 | Fig5BC | control 13 | TRUE |
| 14 | Fig5BC | control 14 | TRUE |
| 15 | Fig5BC | control 15 | TRUE |
| 16 | Fig5BC | control 16 | TRUE |
| 17 | Fig5BC | control 17 | TRUE |
| 18 | Fig5BC | control 18 | TRUE |
| 19 | Fig5BC | control 19 | TRUE |
| 20 | Fig5BC | control 20 | TRUE |
| 1 | Fig5BC RND | TBI 3 | rnd |
| 2 | Fig5BC RND | TBI 31 | rnd |
| 3 | Fig5BC RND | TBI 40 | rnd |
| 4 | Fig5BC RND | TBI 43 | rnd |
| 5 | Fig5BC RND | control 2 | rnd |
| 6 | Fig5BC RND | control 4 | rnd |
| 7 | Fig5BC RND | control 6 | rnd |
| 8 | Fig5BC RND | control 8 | rnd |
| 9 | Fig5BC RND | control 10 | rnd |
| 10 | Fig5BC RND | control 12 | rnd |
| 11 | Fig5BC RND | control 14 | rnd |
| 1 | Fig5BC RND | control 16 | rnd |
| 2 | Fig5BC RND | control 18 | rnd |
| 3 | Fig5BC RND | control 20 | rnd |
| 4 | Fig5BC RND | TBI 7 | rnd |
| 5 | Fig5BC RND | TBI 28 | rnd |
| 6 | Fig5BC RND | TBI 15 | rnd |
| 7 | Fig5BC RND | TBI 35 | rnd |
| 8 | Fig5BC RND | TBI 39 | rnd |
| 9 | Fig5BC RND | TBI 24 | rnd |
| 10 | Fig5BC RND | TBI 19 | rnd |
| 11 | Fig5BC RND | control 1 | rnd |
| 12 | Fig5BC RND | control 3 | rnd |
| 13 | Fig5BC RND | control 5 | rnd |
| 14 | Fig5BC RND | control 7 | rnd |
| 15 | Fig5BC RND | control 9 | rnd |
| 16 | Fig5BC RND | control 11 | rnd |
| 17 | Fig5BC RND | control 13 | rnd |
| 18 | Fig5BC RND | control 15 | rnd |
| 19 | Fig5BC RND | control 17 | rnd |
| 20 | Fig5BC RND | control 19 | rnd |
| 1 | fig5D | TBI 3 | TRUE |
| 2 | fig5D | TBI 15 | TRUE |
| 3 | fig5D | TBI 31 | TRUE |
| 4 | fig5D | TBI 35 | TRUE |
| 5 | fig5D | TBI 40 | TRUE |
| 6 | fig5D | TBI 43 | TRUE |
| 7 | fig5D | TBI 24 | TRUE |
| 8 | fig5D | TBI 7 | TRUE |
| 9 | fig5D | TBI 39 | TRUE |
| 10 | fig5D | TBI 19 | TRUE |
| 11 | fig5D | TBI 28 | TRUE |
| 1 | fig5D | TBI 20 | TRUE |
| 2 | fig5D | TBI 4 | TRUE |
| 3 | fig5D | TBI 41 | TRUE |
| 4 | fig5D | TBI 29 | TRUE |
| 5 | fig5D | TBI 44 | TRUE |
| 6 | fig5D | TBI 45 | TRUE |
| 7 | fig5D | TBI 14 | TRUE |
| 8 | fig5D | TBI 21 | TRUE |
| 9 | fig5D | TBI 11 | TRUE |
| 10 | fig5D | TBI 22 | TRUE |
| 11 | fig5D | TBI 25 | TRUE |
| 12 | fig5D | TBI 42 | TRUE |
| 1 | fig5D RND | TBI 3 | rnd |
| 2 | fig5D RND | TBI 31 | rnd |
| 3 | fig5D RND | TBI 40 | rnd |
| 4 | fig5D RND | TBI 24 | rnd |
| 5 | fig5D RND | TBI 39 | rnd |
| 6 | fig5D RND | TBI 28 | rnd |
| 7 | fig5D RND | TBI 4 | rnd |
| 8 | fig5D RND | TBI 29 | rnd |
| 9 | fig5D RND | TBI 45 | rnd |
| 10 | fig5D RND | TBI 21 | rnd |
| 11 | fig5D RND | TBI 22 | rnd |
| 1 | fig5D RND | TBI 42 | rnd |
| 2 | fig5D RND | TBI 15 | rnd |
| 3 | fig5D RND | TBI 35 | rnd |
| 4 | fig5D RND | TBI 43 | rnd |
| 5 | fig5D RND | TBI 7 | rnd |
| 6 | fig5D RND | TBI 19 | rnd |
| 7 | fig5D RND | TBI 20 | rnd |
| 8 | fig5D RND | TBI 41 | rnd |
| 9 | fig5D RND | TBI 44 | rnd |
| 10 | fig5D RND | TBI 14 | rnd |
| 11 | fig5D RND | TBI 11 | rnd |
| 12 | fig5D RND | TBI 25 | rnd |
| 1.7 (10n10) control | Table 3 | control 3 | Control(minimal) |
| 1.7 (10n10) control | Table 3 | control 4 | Control(minimal) |
| 1.7 (10n10) control | Table 3 | control 6 | Control(minimal) |
| 1.7 (10n10) control | Table 3 | control 7 | Control(minimal) |
| 1.7 (10n10) control | Table 3 | control 8 | Control(minimal) |
| 1.7 (10n10) control | Table 3 | control 9 | Control(minimal) |
| 1.7 (10n10) control | Table 3 | control 10 | Control(minimal) |
| 1.7 (10n10) control | Table 3 | control 11 | Control(minimal) |
| 1.7 (10n10) control | Table 3 | control 12 | Control(minimal) |
| 1.7 (10n10) control | Table 3 | control 13 | Control(minimal) |
| 1.7 (10n10) TBI (most affected) | Table 3 | TBI 1 | TBI(most affected) |
| 1.7 (10n10) TBI (most affected) | Table 3 | TBI 2 | TBI(most affected) |
| 1.7 (10n10) TBI (most affected) | Table 3 | TBI 5 | TBI(most affected) |
| 1.7 (10n10) TBI (most affected) | Table 3 | TBI 6 | TBI(most affected) |
| 1.7 (10n10) TBI (most affected) | Table 3 | TBI 8 | TBI(most affected) |
| 1.7 (10n10) TBI (most affected) | Table 3 | TBI 9 | TBI(most affected) |
| 1.7 (10n10) TBI (most affected) | Table 3 | TBI 10 | TBI(most affected) |
| 1.7 (10n10) TBI (most affected) | Table 3 | TBI 46 | TBI(most affected) |
| 1.7 (10n10) TBI (most affected) | Table 3 | TBI 30 | TBI(most affected) |
| 1.7 (10n10) TBI (most affected) | Table 3 | TBI 47 | TBI(most affected) |
| 1.7 (10n10)+-cm | Table 3 | TBI 19 | TBI+CM |
| 1.7 (10n10)+-cm | Table 3 | TBI 24 | TBI+CM |
| 1.7 (10n10)+-cm | Table 3 | TBI 28 | TBI+CM |
| 1.7 (10n10)+-cm | Table 3 | TBI 3 | TBI+CM |
| 1.7 (10n10)+-cm | Table 3 | TBI 31 | TBI+CM |
| 1.7 (10n10)+-cm | Table 3 | TBI 35 | TBI+CM |
| 1.7 (10n10)+-cm | Table 3 | TBI 39 | TBI+CM |
| 1.7 (10n10)+-cm | Table 3 | TBI 40 | TBI+CM |
| 1.7 (10n10)+-cm | Table 3 | TBI 43 | TBI+CM |
| 1.7 (10n10)+-cm | Table 3 | TBI 7 | TBI+CM |
| 1.7 (10n10)+-cm | Table 3 | TBI 11 | TBI |
| 1.7 (10n10)+-cm | Table 3 | TBI 20 | TBI |
| 1.7 (10n10)+-cm | Table 3 | TBI 21 | TBI |
| 1.7 (10n10)+-cm | Table 3 | TBI 22 | TBI |
| 1.7 (10n10)+-cm | Table 3 | TBI 25 | TBI |
| 1.7 (10n10)+-cm | Table 3 | TBI 41 | TBI |
| 1.7 (10n10)+-cm | Table 3 | TBI 42 | TBI |
| 1.7 (10n10)+-cm | Table 3 | TBI 44 | TBI |
| 1.7 (10n10)+-cm | Table 3 | TBI 45 | TBI |
| 1.7 (10n10)+-cm | Table 3 | TBI 46 | TBI |
