# Supplementary material for: Distinguishing and phenotype monitoring of traumatic brain injury and post-concussion syndrome including chronic migraine in serum of Iraq and Afghanistan war veterans
Source: PLoS One. 2019 Apr 26;14(4):e0215762. doi: 10.1371/journal.pone.0215762 (PMC6485717; doi:10.1371/journal.pone.0215762)
Supplement: S10 Table — MS peaks used by figure number. (DOCX) [file pone.0215762.s036.docx]

**S10 Table. Mass peaks selected by LOOCV process. MS peaks used by figure number.**

| Fig 3 panel A and Fig 3 panel B: peaks utilized | | | | | | | | |
| --- | --- | --- | --- | --- | --- | --- | --- | --- |
| m/Z | | m/Z | | m/Z | | | m/Z | |
| 408 | | 721 | | 1193 | | | 1569 | |
| 410 | | 729 | | 1197 | | | 1571 | |
| 425 | | 772 | | 1202 | | | 1586 | |
| 427 | | 785 | | 1211 | | | 1597 | |
| 429 | | 817 | | 1226 | | | 1606 | |
| 436 | | 830 | | 1235 | | | 1626 | |
| 449 | | 832 | | 1238 | | | 1651 | |
| 460 | | 847 | | 1239 | | | 1681 | |
| 467 | | 848 | | 1240 | | | 1684 | |
| 470 | | 865 | | 1243 | | | 1691 | |
| 495 | | 866 | | 1248 | | | 1739 | |
| 528 | | 873 | | 1305 | | | 1741 | |
| 529 | | 874 | | 1309 | | | 1767 | |
| 534 | | 875 | | 1316 | | | 1770 | |
| 539 | | 876 | | 1328 | | | 1779 | |
| 559 | | 878 | | 1346 | | | 1780 | |
| 564 | | 907 | | 1350 | | | 1786 | |
| 565 | | 919 | | 1362 | | | 1788 | |
| 569 | | 921 | | 1379 | | | 1793 | |
| 573 | | 936 | | 1402 | | | 1798 | |
| 591 | | 944 | | 1416 | | | 1815 | |
| 604 | | 945 | | 1422 | | | 1864 | |
| 611 | | 950 | | 1440 | | | 1866 | |
| 618 | | 955 | | 1446 | | | 1873 | |
| 635 | | 1017 | | 1489 | | | 1880 | |
| 648 | | 1048 | | 1491 | | | 1889 | |
| 651 | | 1051 | | 1494 | | | 1903 | |
| 656 | | 1061 | | 1499 | | | 1920 | |
| 685 | | 1067 | | 1505 | | | 1942 | |
| 687 | | 1117 | | 1515 | | | 1946 | |
| 689 | | 1149 | | 1517 | | | 1962 | |
| 697 | | 1157 | | 1554 | | | 1974 | |
| 709 | | 1184 | | 1562 | | | 1975 | |
| Fig 4 panel A, Fig 4 panel B, Fig 4 panel C:Peaks Utilized | | | | | | | | |
| m/Z | | m/Z | | m/Z | | | m/Z | |
| 408 | | 1091 | | 1505 | | | 1796 | |
| 410 | | 1104 | | 1533 | | | 1798 | |
| 425 | | 1134 | | 1554 | | | 1815 | |
| 427 | | 1202 | | 1561 | | | 1819 | |
| 467 | | 1211 | | 1562 | | | 1858 | |
| 470 | | 1226 | | 1571 | | | 1864 | |
| 539 | | 1232 | | 1586 | | | 1866 | |
| 565 | | 1240 | | 1626 | | | 1880 | |
| 573 | | 1284 | | 1627 | | | 1886 | |
| 611 | | 1305 | | 1659 | | | 1889 | |
| 635 | | 1316 | | 1679 | | | 1903 | |
| 656 | | 1323 | | 1681 | | | 1904 | |
| 710 | | 1327 | | 1684 | | | 1906 | |
| 770 | | 1328 | | 1733 | | | 1917 | |
| 830 | | 1362 | | 1739 | | | 1923 | |
| 832 | | 1402 | | 1741 | | | 1927 | |
| 847 | | 1417 | | 1755 | | | 1931 | |
| 848 | | 1422 | | 1767 | | | 1942 | |
| 865 | | 1446 | | 1770 | | | 1946 | |
| 907 | | 1489 | | 1774 | | | 1962 | |
| 945 | | 1494 | | 1780 | | | 1968 | |
|  | |  | |  | | |  | |
| Fig 4 panel D:Peaks Utilized | | | | | | | | |
| m/Z | | m/Z | | m/Z | | | m/Z | |
| 438 | | 787 | | 1147 | | | 1555 | |
| 462 | | 800 | | 1155 | | | 1601 | |
| 506 | | 816 | | 1157 | | | 1613 | |
| 510 | | 840 | | 1202 | | | 1650 | |
| 520 | | 842 | | 1214 | | | 1656 | |
| 521 | | 852 | | 1229 | | | 1670 | |
| 524 | | 859 | | 1249 | | | 1673 | |
| 534 | | 860 | | 1292 | | | 1679 | |
| 542 | | 865 | | 1294 | | | 1700 | |
| 543 | | 866 | | 1296 | | | 1707 | |
| 546 | | 875 | | 1297 | | | 1721 | |
| 554 | | 897 | | 1366 | | | 1782 | |
| 591 | | 1049 | | 1374 | | | 1786 | |
| 606 | | 1053 | | 1407 | | | 1788 | |
| 610 | | 1056 | | 1411 | | | 1799 | |
| 626 | | 1064 | | 1418 | | | 1807 | |
| 653 | | 1067 | | 1420 | | | 1815 | |
| 675 | | 1084 | | 1490 | | | 1918 | |
| 722 | | 1127 | | 1502 | | | 1951 | |
| 736 | | 1136 | | 1513 | | | 1962 | |
| 740 | | 1137 | | 1535 | | |  | |
| 744 | | 1139 | | 1553 | | |  | |
|  | |  | |  | | |  | |
| Fig 5 panel A: Peaks Utilized | | | | | | | | |
| m/Z | | m/Z | | m/Z | | | m/Z | |
| 401 | | 885 | | 1284 | | | 1613 | |
| 410 | | 890 | | 1287 | | | 1626 | |
| 413 | | 902 | | 1292 | | | 1629 | |
| 425 | | 903 | | 1294 | | | 1649 | |
| 433 | | 907 | | 1296 | | | 1650 | |
| 470 | | 919 | | 1298 | | | 1670 | |
| 484 | | 933 | | 1324 | | | 1673 | |
| 501 | | 975 | | 1327 | | | 1678 | |
| 504 | | 990 | | 1334 | | | 1679 | |
| 538 | | 1029 | | 1339 | | | 1700 | |
| 554 | | 1033 | | 1340 | | | 1712 | |
| 562 | | 1041 | | 1344 | | | 1718 | |
| 568 | | 1049 | | 1351 | | | 1721 | |
| 617 | | 1056 | | 1363 | | | 1722 | |
| 618 | | 1062 | | 1364 | | | 1724 | |
| 653 | | 1065 | | 1366 | | | 1774 | |
| 673 | | 1070 | | 1367 | | | 1782 | |
| 685 | | 1108 | | 1388 | | | 1807 | |
| 721 | | 1117 | | 1411 | | | 1808 | |
| 732 | | 1119 | | 1420 | | | 1831 | |
| 737 | | 1136 | | 1460 | | | 1837 | |
| 750 | | 1138 | | 1465 | | | 1846 | |
| 753 | | 1139 | | 1466 | | | 1864 | |
| 757 | | 1154 | | 1470 | | | 1873 | |
| 760 | | 1155 | | 1484 | | | 1892 | |
| 781 | | 1164 | | 1495 | | | 1909 | |
| 787 | | 1176 | | 1500 | | | 1920 | |
| 790 | | 1193 | | 1505 | | | 1922 | |
| 803 | | 1201 | | 1513 | | | 1935 | |
| 807 | | 1228 | | 1526 | | | 1958 | |
| 812 | | 1238 | | 1530 | | | 1963 | |
| 861 | | 1275 | | 1582 | | | 1964 | |
| 863 | | 1277 | | 1601 | | | 1986 | |
| 867 | |  | |  | | | 1992 | |
| Fig 5 panel B and Fig 5 panel C: Peaks Utilized | | | | | | | | |
| m/Z | | m/Z | | m/Z | | | m/Z | |
| 437 | | 856 | | 1148 | | | 1479 | |
| 474 | | 857 | | 1149 | | | 1483 | |
| 486 | | 861 | | 1154 | | | 1486 | |
| 520 | | 874 | | 1188 | | | 1500 | |
| 562 | | 914 | | 1203 | | | 1505 | |
| 569 | | 918 | | 1212 | | | 1532 | |
| 571 | | 919 | | 1236 | | | 1557 | |
| 578 | | 943 | | 1261 | | | 1595 | |
| 585 | | 954 | | 1274 | | | 1604 | |
| 632 | | 979 | | 1279 | | | 1605 | |
| 645 | | 1020 | | 1284 | | | 1615 | |
| 671 | | 1026 | | 1320 | | | 1629 | |
| 694 | | 1028 | | 1365 | | | 1636 | |
| 740 | | 1069 | | 1376 | | | 1651 | |
| 745 | | 1088 | | 1387 | | | 1783 | |
| 752 | | 1093 | | 1404 | | | 1788 | |
| 760 | | 1096 | | 1416 | | | 1842 | |
| 770 | | 1119 | | 1426 | | | 1858 | |
| 778 | | 1125 | | 1436 | | | 1864 | |
| 800 | | 1136 | | 1437 | | | 1870 | |
| 810 | | 1138 | | 1438 | | | 1880 | |
| 815 | | 1142 | | 1478 | | | 1922 | |
| 840 | |  | |  | | |  | |
| Fig 5 panel D: Peaks Utilized | | | | | | | | |
| m/Z | | m/Z | | m/Z | | | m/Z | |
| 421 | | 807 | | 1197 | | | 1604 | |
| 445 | | 810 | | 1221 | | | 1615 | |
| 459 | | 856 | | 1236 | | | 1616 | |
| 470 | | 862 | | 1274 | | | 1626 | |
| 472 | | 874 | | 1275 | | | 1636 | |
| 474 | | 903 | | 1292 | | | 1640 | |
| 486 | | 932 | | 1294 | | | 1650 | |
| 487 | | 941 | | 1301 | | | 1670 | |
| 491 | | 943 | | 1316 | | | 1694 | |
| 509 | | 973 | | 1319 | | | 1736 | |
| 519 | | 975 | | 1320 | | | 1741 | |
| 520 | | 979 | | 1327 | | | 1772 | |
| 522 | | 980 | | 1334 | | | 1774 | |
| 535 | | 993 | | 1359 | | | 1786 | |
| 570 | | 1020 | | 1365 | | | 1788 | |
| 576 | | 1022 | | 1411 | | | 1812 | |
| 617 | | 1026 | | 1412 | | | 1842 | |
| 632 | | 1029 | | 1460 | | | 1846 | |
| 645 | | 1069 | | 1465 | | | 1856 | |
| 650 | | 1076 | | 1474 | | | 1865 | |
| 653 | | 1093 | | 1478 | | | 1870 | |
| 707 | | 1100 | | 1479 | | | 1892 | |
| 710 | | 1142 | | 1502 | | | 1903 | |
| 752 | | 1155 | | 1507 | | | 1954 | |
| 770 | | 1179 | | 1549 | | | 1964 | |
| 778 | | 1188 | | 1585 | | | 1982 | |
| 787 | |  | |  | | |  | |
| Peak selection minimum frequency and m/Z range values by Figure | | | | | | | | |
|  | Fig 3 panel A and Fig 3 panel B | Fig 5 panel A | Fig 4 panel A, Fig 4 panel B and Fig 4 panel C | | Fig 4 panel D | Fig 5 panel B and Fig 5 panel C | | Fig 5 panel D |
| Frequency minimum | 0.4 | 0.6 | 0.36 | | 0.8 | 0.89 | | 0.4 |
| Range m/Z | 400-2000 | 400-2000mz | 400-2000 select peaks as indicated by peak list | | 438-897, 1049-1297, 1366-1420, 1490-1721, 1782-1815, 1918-2000 | 437-1656, 1783-1953 | | 400-2000 |
