# Supplementary material for: Distinguishing and phenotype monitoring of traumatic brain injury and post-concussion syndrome including chronic migraine in serum of Iraq and Afghanistan war veterans
Source: PLoS One. 2019 Apr 26;14(4):e0215762. doi: 10.1371/journal.pone.0215762 (PMC6485717; doi:10.1371/journal.pone.0215762)
Supplement: S11 Table — Mass peaks selected for MS/MS analysis in Figs 6 and 7. (DOCX) [file pone.0215762.s037.docx]

**S11 Table. LOOCV MS/MS peaks utilized. Mass peaks selected for MS/MS analysis in Fig 6 and Fig 7.**

| **Fig 6: Mass Peaks selected for MS/MS analysis** | | | |
| --- | --- | --- | --- |
| 539 | 618 | 785 | 875 |
| 559 | 635 | 817 | 876 |
| 564 | 648 | 830 | 878 |
| 565 | 651 | 832 | 907 |
| 569 | 687 | 847 | 919 |
| 573 | 689 | 848 | 921 |
| 591 | 709 | 865 | 936 |
| 604 | 721 | 866 | 945 |
| 611 | 729 | 874 | 955 |
| **Fig 7: Mass Peaks selected for MS/MS analysis** | | | |
| 630 | 770 | 875 | 980 |
| 645 | 777 | 902 | 983 |
| 654 | 778 | 903 | 1003 |
| 655 | 820 | 932 | 1020 |
| 675 | 824 | 933 | 1029 |
| 702 | 830 | 943 | 1049 |
| 747 | 847 | 957 | 1076 |
| 759 | 862 | 973 | 1097 |
| 769 | 874 | 979 | 1100 |
