# Supplementary material for: Distinguishing and phenotype monitoring of traumatic brain injury and post-concussion syndrome including chronic migraine in serum of Iraq and Afghanistan war veterans
Source: PLoS One. 2019 Apr 26;14(4):e0215762. doi: 10.1371/journal.pone.0215762 (PMC6485717; doi:10.1371/journal.pone.0215762)
Supplement: S12 Table — (DOCX) [file pone.0215762.s038.docx]

**S12 Table. Peptides identified using MS/MS by patient: TBI most affected vs control (least affected).**

| Protein Gene symbol | IMLOG2 ratio: ((#Hits Worst+1)/ (#Hits least+1)) | # unique peptides identified for protein [ TBI (most affected) Total hits : control (least affected) Total hits ] | # sera TBI (most affected) vs control (Least) | control 3 | control 4 | control 6 | control 7 | control 8 | control 9 | control 10 | control 11 | control 12 | control 13 | TBI 1 | TBI 2 | TBI 5 | TBI 6 | TBI 8 | TBI 9 | TBI 10 | TBI 46 | TBI 30 | TBI 47 |
| --- | --- | --- | --- | --- | --- | --- | --- | --- | --- | --- | --- | --- | --- | --- | --- | --- | --- | --- | --- | --- | --- | --- | --- |
| IGH | 1.96347412397489 | 13 [ 38 : 9 ] | 8 : 3 | 0 | 0 | 0 | 0 | 0 | 2 | 2 | 5 | 0 | 0 | 9 | 9 | 3 | 3 | 0 | 4 | 3 | 9 | 4 | 3 |
| TTN | 0.0766212816029124 | 13 [ 57 : 54 ] | 4 : 5 | 0 | 0 | 0 | 0 | 6 | 15 | 0 | 4 | 13 | 16 | 54 | 0 | 0 | 11 | 0 | 0 | 4 | 0 | 0 | 22 |
| SSPO | 0.282399730700725 | 15 [ 44 : 36 ] | 3 : 4 | 0 | 4 | 0 | 10 | 7 | 0 | 15 | 0 | 0 | 0 | 36 | 0 | 0 | 0 | 0 | 0 | 0 | 31 | 11 | 0 |
| NOTCH4 | -0.0759488532332986 | 7 [ 36 : 38 ] | 4 : 2 | 13 | 25 | 0 | 0 | 0 | 0 | 0 | 0 | 0 | 0 | 38 | 7 | 0 | 0 | 0 | 7 | 13 | 0 | 0 | 9 |
| IGL | -0.567040592723894 | 6 [ 26 : 39 ] | 4 : 2 | 0 | 0 | 0 | 36 | 0 | 0 | 3 | 0 | 0 | 0 | 39 | 0 | 3 | 0 | 5 | 0 | 0 | 15 | 0 | 0 |
| PCLO | 2.92599941855622 | 6 [ 37 : 4 ] | 4 : 1 | 0 | 0 | 4 | 0 | 0 | 0 | 0 | 0 | 0 | 0 | 4 | 0 | 8 | 0 | 0 | 0 | 5 | 15 | 0 | 9 |
| TRB | -0.415037499278844 | 7 [ 11 : 15 ] | 2 : 4 | 0 | 3 | 0 | 3 | 6 | 0 | 0 | 0 | 3 | 0 | 15 | 0 | 0 | 0 | 0 | 0 | 6 | 0 | 0 | 0 |
| MUC19 | 4.16992500144231 | 4 [ 17 : 0 ] | 4 : 0 | 0 | 0 | 0 | 0 | 0 | 0 | 0 | 0 | 0 | 0 | 0 | 0 | 0 | 0 | 3 | 3 | 5 | 0 | 0 | 6 |
| SON | -6.58496250072116 | 3 [ 0 : 95 ] | 0 : 3 | 0 | 0 | 0 | 0 | 3 | 0 | 0 | 69 | 0 | 23 | 95 | 0 | 0 | 0 | 0 | 0 | 0 | 0 | 0 | 0 |
| LAMA5 | 0.405992359675837 | 10 [ 52 : 39 ] | 3 : 3 | 0 | 5 | 0 | 0 | 0 | 0 | 18 | 0 | 16 | 0 | 39 | 0 | 0 | 0 | 0 | 9 | 0 | 0 | 0 | 17 |
| MUC5AC | 1 | 7 [ 49 : 24 ] | 1 : 3 | 0 | 12 | 0 | 3 | 0 | 0 | 0 | 9 | 0 | 0 | 24 | 0 | 0 | 0 | 0 | 0 | 0 | 0 | 0 | 0 |
| ZNF268 | 0.271302021817394 | 8 [ 34 : 28 ] | 3 : 2 | 0 | 0 | 0 | 13 | 0 | 0 | 0 | 0 | 15 | 0 | 28 | 0 | 2 | 12 | 0 | 0 | 20 | 0 | 0 | 0 |
| SYNE1 | -5.85798099512757 | 3 [ 0 : 57 ] | 0 : 3 | 0 | 0 | 0 | 31 | 0 | 0 | 0 | 0 | 17 | 9 | 57 | 0 | 0 | 0 | 0 | 0 | 0 | 0 | 0 | 0 |
| FBN2 | 0.874469117916141 | 5 [ 32 : 17 ] | 2 : 3 | 0 | 0 | 0 | 0 | 0 | 7 | 0 | 7 | 0 | 3 | 17 | 0 | 0 | 26 | 6 | 0 | 0 | 0 | 0 | 0 |
| IGK | 0.347923303420307 | 9 [ 27 : 21 ] | 3 : 3 | 0 | 0 | 0 | 0 | 12 | 2 | 0 | 7 | 0 | 0 | 21 | 0 | 0 | 0 | 0 | 10 | 14 | 0 | 0 | 0 |
| FAT4 | 1.28010791919274 | 4 [ 33 : 13 ] | 3 : 1 | 0 | 0 | 13 | 0 | 0 | 0 | 0 | 0 | 0 | 0 | 13 | 0 | 7 | 0 | 0 | 0 | 17 | 9 | 0 | 0 |
| GRM4 | 5.39231742277876 | 2 [ 41 : 0 ] | 3 : 0 | 0 | 0 | 0 | 0 | 0 | 0 | 0 | 0 | 0 | 0 | 0 | 11 | 0 | 0 | 0 | 11 | 0 | 0 | 0 | 19 |
| LRP1 | 2.08746284125034 | 7 [ 33 : 7 ] | 3 : 1 | 0 | 0 | 0 | 0 | 0 | 7 | 0 | 0 | 0 | 0 | 7 | 0 | 0 | 0 | 21 | 0 | 4 | 0 | 0 | 8 |
| MT-ND4 | -0.497499659470817 | 5 [ 16 : 23 ] | 1 : 3 | 0 | 0 | 0 | 3 | 0 | 12 | 0 | 8 | 0 | 0 | 23 | 0 | 0 | 16 | 0 | 0 | 0 | 0 | 0 | 0 |
| HECTD4 | -0.584962500721156 | 6 [ 15 : 23 ] | 3 : 3 | 0 | 0 | 0 | 0 | 0 | 3 | 11 | 0 | 9 | 0 | 23 | 4 | 0 | 0 | 0 | 4 | 0 | 0 | 0 | 7 |
| ZNRF3 | -5.20945336562895 | 1 [ 0 : 36 ] | 0 : 3 | 0 | 0 | 0 | 0 | 0 | 12 | 0 | 12 | 0 | 12 | 36 | 0 | 0 | 0 | 0 | 0 | 0 | 0 | 0 | 0 |
| NSD1 | 5.16992500144231 | 3 [ 35 : 0 ] | 3 : 0 | 0 | 0 | 0 | 0 | 0 | 0 | 0 | 0 | 0 | 0 | 0 | 11 | 0 | 0 | 0 | 11 | 0 | 13 | 0 | 0 |
| USH2A | -0.234465253637023 | 7 [ 16 : 19 ] | 2 : 3 | 0 | 0 | 13 | 0 | 0 | 3 | 0 | 3 | 0 | 0 | 19 | 0 | 0 | 0 | 11 | 0 | 0 | 0 | 0 | 0 |
| FCGBP | -0.823122237915921 | 6 [ 12 : 22 ] | 2 : 3 | 0 | 10 | 0 | 0 | 0 | 0 | 3 | 0 | 9 | 0 | 22 | 5 | 0 | 0 | 0 | 0 | 0 | 0 | 0 | 7 |
| TENM2 | -1 | 3 [ 11 : 23 ] | 1 : 3 | 0 | 0 | 0 | 11 | 0 | 6 | 0 | 6 | 0 | 0 | 23 | 0 | 11 | 0 | 0 | 0 | 0 | 0 | 0 | 0 |
| ATRN | -4.8073549220576 | 2 [ 0 : 27 ] | 0 : 3 | 0 | 0 | 0 | 0 | 0 | 11 | 5 | 11 | 0 | 0 | 27 | 0 | 0 | 0 | 0 | 0 | 0 | 0 | 0 | 0 |
| PRUNE2 | -4.8073549220576 | 2 [ 0 : 27 ] | 0 : 3 | 0 | 0 | 0 | 0 | 23 | 2 | 0 | 2 | 0 | 0 | 27 | 0 | 0 | 0 | 0 | 0 | 0 | 0 | 0 | 0 |
| FBN3 | -1.07800251200127 | 7 [ 8 : 18 ] | 2 : 3 | 0 | 4 | 0 | 0 | 0 | 4 | 0 | 10 | 0 | 0 | 18 | 4 | 0 | 0 | 0 | 0 | 0 | 0 | 4 | 0 |
| ITGA8 | 4.52356195605701 | 2 [ 22 : 0 ] | 3 : 0 | 0 | 0 | 0 | 0 | 0 | 0 | 0 | 0 | 0 | 0 | 0 | 7 | 0 | 0 | 0 | 7 | 0 | 8 | 0 | 0 |
| MALRD1 | 4.32192809488736 | 2 [ 19 : 0 ] | 3 : 0 | 0 | 0 | 0 | 0 | 0 | 0 | 0 | 0 | 0 | 0 | 0 | 7 | 0 | 0 | 0 | 7 | 0 | 0 | 0 | 0 |
| CSMD1 | 4.16992500144231 | 2 [ 17 : 0 ] | 3 : 0 | 0 | 0 | 0 | 0 | 0 | 0 | 0 | 0 | 0 | 0 | 0 | 5 | 0 | 0 | 0 | 5 | 0 | 0 | 7 | 0 |
| PKD1 | 4.08746284125034 | 3 [ 16 : 0 ] | 3 : 0 | 0 | 0 | 0 | 0 | 0 | 0 | 0 | 0 | 0 | 0 | 0 | 0 | 0 | 0 | 5 | 0 | 0 | 0 | 6 | 0 |
| C18orf15 | -4 | 3 [ 0 : 15 ] | 0 : 3 | 0 | 5 | 0 | 0 | 3 | 0 | 7 | 0 | 0 | 0 | 15 | 0 | 0 | 0 | 0 | 0 | 0 | 0 | 0 | 0 |
| CNNM1 | 4 | 2 [ 15 : 0 ] | 3 : 0 | 0 | 0 | 0 | 0 | 0 | 0 | 0 | 0 | 0 | 0 | 0 | 5 | 0 | 0 | 5 | 5 | 0 | 0 | 0 | 0 |
| PTPRM | 4 | 2 [ 15 : 0 ] | 3 : 0 | 0 | 0 | 0 | 0 | 0 | 0 | 0 | 0 | 0 | 0 | 0 | 6 | 0 | 3 | 0 | 6 | 0 | 0 | 0 | 0 |
| SERINC2 | 4 | 1 [ 15 : 0 ] | 3 : 0 | 0 | 0 | 0 | 0 | 0 | 0 | 0 | 0 | 0 | 0 | 0 | 4 | 0 | 7 | 0 | 4 | 0 | 0 | 0 | 0 |
| SIMC1 | 4 | 2 [ 15 : 0 ] | 3 : 0 | 0 | 0 | 0 | 0 | 0 | 0 | 0 | 0 | 0 | 0 | 0 | 5 | 0 | 0 | 0 | 5 | 5 | 0 | 0 | 0 |
| TNRC18 | -4 | 2 [ 0 : 15 ] | 0 : 3 | 0 | 0 | 0 | 0 | 0 | 4 | 7 | 4 | 0 | 0 | 15 | 0 | 0 | 0 | 0 | 0 | 0 | 0 | 0 | 0 |
| TCF20 | -3.80735492205761 | 2 [ 0 : 13 ] | 0 : 3 | 0 | 0 | 0 | 0 | 0 | 5 | 3 | 5 | 0 | 0 | 13 | 0 | 0 | 0 | 0 | 0 | 0 | 0 | 0 | 0 |
| POLA1 | -3.16992500144231 | 1 [ 0 : 8 ] | 0 : 3 | 0 | 0 | 0 | 0 | 0 | 0 | 2 | 0 | 3 | 3 | 8 | 0 | 0 | 0 | 0 | 0 | 0 | 0 | 0 | 0 |
| DNAJC5 | -3.03747470541866 | 3 [ 18 : 155 ] | 2 : 2 | 0 | 0 | 0 | 117 | 0 | 0 | 0 | 0 | 0 | 38 | 155 | 0 | 0 | 9 | 0 | 0 | 0 | 9 | 0 | 0 |
| ADGRL2 | -7.4262647547021 | 2 [ 0 : 171 ] | 0 : 2 | 0 | 0 | 0 | 0 | 0 | 0 | 0 | 0 | 128 | 43 | 171 | 0 | 0 | 0 | 0 | 0 | 0 | 0 | 0 | 0 |
| DNAJC5B | 4.44057259138598 | 7 [ 151 : 6 ] | 2 : 2 | 0 | 0 | 0 | 0 | 0 | 3 | 0 | 3 | 0 | 0 | 6 | 0 | 0 | 0 | 0 | 0 | 0 | 125 | 0 | 26 |
| OTOGL | 1.22239242133645 | 3 [ 90 : 38 ] | 2 : 1 | 0 | 0 | 0 | 0 | 0 | 0 | 0 | 38 | 0 | 0 | 38 | 10 | 0 | 0 | 0 | 0 | 0 | 0 | 80 | 0 |
| EBF4 | -0.691877704637668 | 7 [ 38 : 62 ] | 2 : 2 | 0 | 0 | 0 | 0 | 0 | 0 | 0 | 0 | 37 | 25 | 62 | 0 | 0 | 13 | 0 | 0 | 25 | 0 | 0 | 0 |
| MUC2 | 5.97727992349992 | 2 [ 62 : 0 ] | 2 : 0 | 0 | 0 | 0 | 0 | 0 | 0 | 0 | 0 | 0 | 0 | 0 | 0 | 3 | 0 | 0 | 0 | 0 | 0 | 0 | 0 |
| TENM4 | -0.289506617194985 | 2 [ 26 : 32 ] | 2 : 1 | 0 | 0 | 0 | 0 | 0 | 0 | 0 | 0 | 0 | 32 | 32 | 13 | 0 | 0 | 0 | 13 | 0 | 0 | 0 | 0 |
| VWF | 1.71620703399941 | 6 [ 45 : 13 ] | 2 : 1 | 0 | 0 | 0 | 0 | 0 | 0 | 0 | 0 | 0 | 13 | 13 | 0 | 32 | 0 | 0 | 0 | 0 | 13 | 0 | 0 |
| SEC16A | -5.75488750216347 | 2 [ 0 : 53 ] | 0 : 2 | 0 | 0 | 36 | 0 | 0 | 0 | 0 | 0 | 17 | 0 | 53 | 0 | 0 | 0 | 0 | 0 | 0 | 0 | 0 | 0 |
| PAMR1 | 5.70043971814109 | 2 [ 51 : 0 ] | 2 : 0 | 0 | 0 | 0 | 0 | 0 | 0 | 0 | 0 | 0 | 0 | 0 | 0 | 0 | 0 | 0 | 36 | 15 | 0 | 0 | 0 |
| EFL1 | 5.6724253419715 | 2 [ 50 : 0 ] | 2 : 0 | 0 | 0 | 0 | 0 | 0 | 0 | 0 | 0 | 0 | 0 | 0 | 0 | 0 | 17 | 0 | 0 | 33 | 0 | 0 | 0 |
| SPHKAP | -2.39231742277876 | 3 [ 7 : 41 ] | 1 : 2 | 0 | 0 | 0 | 0 | 0 | 23 | 0 | 18 | 0 | 0 | 41 | 0 | 0 | 0 | 0 | 0 | 7 | 0 | 0 | 0 |
| PTPN5 | 5.55458885167764 | 1 [ 46 : 0 ] | 2 : 0 | 0 | 0 | 0 | 0 | 0 | 0 | 0 | 0 | 0 | 0 | 0 | 23 | 0 | 0 | 0 | 23 | 0 | 0 | 0 | 0 |
| FBN1 | -5.49185309632968 | 2 [ 0 : 44 ] | 0 : 2 | 0 | 0 | 0 | 6 | 0 | 0 | 0 | 0 | 0 | 38 | 44 | 0 | 0 | 0 | 0 | 0 | 0 | 0 | 0 | 0 |
| ANO2 | 1.95935801550265 | 2 [ 34 : 8 ] | 2 : 1 | 0 | 8 | 0 | 0 | 0 | 0 | 0 | 0 | 0 | 0 | 8 | 17 | 0 | 0 | 0 | 17 | 0 | 0 | 0 | 0 |
| KCNMB3 | -5.4262647547021 | 2 [ 0 : 42 ] | 0 : 2 | 0 | 0 | 0 | 0 | 0 | 21 | 0 | 21 | 0 | 0 | 42 | 0 | 0 | 0 | 0 | 0 | 0 | 0 | 0 | 0 |
| SPATA7 | 5.4262647547021 | 3 [ 42 : 0 ] | 2 : 0 | 0 | 0 | 0 | 0 | 0 | 0 | 0 | 0 | 0 | 0 | 0 | 0 | 0 | 0 | 34 | 0 | 0 | 0 | 0 | 8 |
| WFDC3 | 1.76553474636298 | 3 [ 33 : 9 ] | 2 : 1 | 0 | 0 | 0 | 0 | 0 | 9 | 0 | 0 | 0 | 0 | 9 | 0 | 0 | 7 | 0 | 26 | 0 | 0 | 0 | 0 |
| REXO1 | -5.39231742277876 | 2 [ 0 : 41 ] | 0 : 2 | 0 | 0 | 0 | 0 | 0 | 9 | 0 | 32 | 0 | 0 | 41 | 0 | 0 | 0 | 0 | 0 | 0 | 0 | 0 | 0 |
| LOC100996750 | 0.556393348524385 | 3 [ 24 : 16 ] | 2 : 1 | 0 | 0 | 0 | 0 | 0 | 0 | 0 | 16 | 0 | 0 | 16 | 0 | 0 | 0 | 0 | 23 | 0 | 1 | 0 | 0 |
| TRPC1 | -3.70043971814109 | 2 [ 2 : 38 ] | 1 : 2 | 0 | 0 | 0 | 0 | 0 | 19 | 0 | 19 | 0 | 0 | 38 | 0 | 0 | 0 | 0 | 0 | 0 | 2 | 0 | 0 |
| CPAMD8 | -1.10691520391651 | 2 [ 12 : 27 ] | 1 : 2 | 12 | 0 | 0 | 0 | 0 | 0 | 0 | 0 | 15 | 0 | 27 | 0 | 0 | 0 | 12 | 0 | 0 | 0 | 0 | 0 |
| LIMK1 | 1.16992500144231 | 2 [ 26 : 11 ] | 2 : 1 | 0 | 0 | 0 | 11 | 0 | 0 | 0 | 0 | 0 | 0 | 11 | 13 | 0 | 0 | 0 | 13 | 0 | 0 | 0 | 0 |
| OTOG | -0.943416471633633 | 4 [ 12 : 24 ] | 2 : 2 | 0 | 0 | 12 | 12 | 0 | 0 | 0 | 0 | 0 | 0 | 24 | 6 | 0 | 0 | 0 | 6 | 0 | 0 | 0 | 0 |
| NFX1 | 3.04439411935845 | 5 [ 32 : 3 ] | 2 : 1 | 0 | 0 | 0 | 0 | 0 | 0 | 0 | 0 | 0 | 3 | 3 | 0 | 0 | 0 | 0 | 0 | 0 | 17 | 0 | 15 |
| SV2C | 2.63226821549951 | 3 [ 30 : 4 ] | 2 : 1 | 0 | 0 | 0 | 0 | 0 | 0 | 0 | 0 | 0 | 4 | 4 | 0 | 0 | 0 | 0 | 0 | 0 | 0 | 24 | 6 |
| TENM3 | 0.485426827170242 | 3 [ 20 : 14 ] | 2 : 1 | 14 | 0 | 0 | 0 | 0 | 0 | 0 | 0 | 0 | 0 | 14 | 0 | 0 | 0 | 0 | 0 | 0 | 0 | 13 | 0 |
| PFN2 | -5.04439411935845 | 1 [ 0 : 32 ] | 0 : 2 | 0 | 0 | 0 | 0 | 0 | 16 | 0 | 16 | 0 | 0 | 32 | 0 | 0 | 0 | 0 | 0 | 0 | 0 | 0 | 0 |
| KLHL8 | 4.95419631038688 | 1 [ 30 : 0 ] | 2 : 0 | 0 | 0 | 0 | 0 | 0 | 0 | 0 | 0 | 0 | 0 | 0 | 15 | 0 | 0 | 0 | 15 | 0 | 0 | 0 | 0 |
| LAMB4 | -4.95419631038688 | 2 [ 0 : 30 ] | 0 : 2 | 0 | 0 | 21 | 0 | 9 | 0 | 0 | 0 | 0 | 0 | 30 | 0 | 0 | 0 | 0 | 0 | 0 | 0 | 0 | 0 |
| LVRN | -3.27301849440642 | 3 [ 2 : 28 ] | 1 : 2 | 0 | 0 | 0 | 0 | 0 | 15 | 13 | 0 | 0 | 0 | 28 | 0 | 0 | 2 | 0 | 0 | 0 | 0 | 0 | 0 |
| TFR2 | -4.95419631038688 | 1 [ 0 : 30 ] | 0 : 2 | 0 | 0 | 0 | 15 | 15 | 0 | 0 | 0 | 0 | 0 | 30 | 0 | 0 | 0 | 0 | 0 | 0 | 0 | 0 | 0 |
| ASXL1 | -4.90689059560852 | 3 [ 0 : 29 ] | 0 : 2 | 0 | 0 | 0 | 0 | 14 | 15 | 0 | 0 | 0 | 0 | 29 | 0 | 0 | 0 | 0 | 0 | 0 | 0 | 0 | 0 |
| CRACR2B | 4.90689059560852 | 2 [ 29 : 0 ] | 2 : 0 | 0 | 0 | 0 | 0 | 0 | 0 | 0 | 0 | 0 | 0 | 0 | 0 | 0 | 22 | 0 | 0 | 0 | 0 | 0 | 0 |
| PTPN23 | -4.90689059560852 | 2 [ 0 : 29 ] | 0 : 2 | 0 | 0 | 0 | 0 | 0 | 0 | 3 | 0 | 0 | 26 | 29 | 0 | 0 | 0 | 0 | 0 | 0 | 0 | 0 | 0 |
| RUBCN | -4.90689059560852 | 2 [ 0 : 29 ] | 0 : 2 | 0 | 0 | 0 | 0 | 0 | 8 | 0 | 0 | 21 | 0 | 29 | 0 | 0 | 0 | 0 | 0 | 0 | 0 | 0 | 0 |
| TMEM55B | 4.85798099512757 | 1 [ 28 : 0 ] | 2 : 0 | 0 | 0 | 0 | 0 | 0 | 0 | 0 | 0 | 0 | 0 | 0 | 14 | 0 | 0 | 0 | 14 | 0 | 0 | 0 | 0 |
| CUBN | -4.8073549220576 | 2 [ 0 : 27 ] | 0 : 2 | 0 | 0 | 0 | 0 | 0 | 0 | 0 | 20 | 7 | 0 | 27 | 0 | 0 | 0 | 0 | 0 | 0 | 0 | 0 | 0 |
| HLA-DQB1 | 0.299560281858908 | 3 [ 15 : 12 ] | 1 : 2 | 0 | 0 | 0 | 0 | 0 | 3 | 0 | 0 | 0 | 9 | 12 | 0 | 0 | 0 | 0 | 0 | 0 | 15 | 0 | 0 |
| SHANK1 | 4.8073549220576 | 2 [ 27 : 0 ] | 2 : 0 | 0 | 0 | 0 | 0 | 0 | 0 | 0 | 0 | 0 | 0 | 0 | 0 | 0 | 0 | 0 | 20 | 0 | 0 | 7 | 0 |
| MT-CO1 | -1.32192809488736 | 5 [ 7 : 19 ] | 1 : 2 | 0 | 0 | 0 | 0 | 0 | 3 | 0 | 0 | 0 | 16 | 19 | 0 | 0 | 7 | 0 | 0 | 0 | 0 | 0 | 0 |
| MTMR3 | 4.75488750216347 | 2 [ 26 : 0 ] | 2 : 0 | 0 | 0 | 0 | 0 | 0 | 0 | 0 | 0 | 0 | 0 | 0 | 0 | 0 | 2 | 0 | 0 | 0 | 0 | 24 | 0 |
| RBM15 | 4.75488750216347 | 2 [ 26 : 0 ] | 2 : 0 | 0 | 0 | 0 | 0 | 0 | 0 | 0 | 0 | 0 | 0 | 0 | 0 | 18 | 0 | 0 | 0 | 8 | 0 | 0 | 0 |
| LAMA1 | 1.51457317282976 | 5 [ 19 : 6 ] | 2 : 1 | 6 | 0 | 0 | 0 | 0 | 0 | 0 | 0 | 0 | 0 | 6 | 0 | 0 | 0 | 0 | 0 | 11 | 0 | 0 | 8 |
| MDFIC | -1.8073549220576 | 3 [ 5 : 20 ] | 1 : 2 | 0 | 0 | 0 | 0 | 0 | 15 | 0 | 0 | 5 | 0 | 20 | 0 | 0 | 0 | 0 | 0 | 0 | 0 | 0 | 5 |
| NBAS | -4.70043971814109 | 3 [ 0 : 25 ] | 0 : 2 | 0 | 0 | 0 | 0 | 13 | 0 | 0 | 0 | 12 | 0 | 25 | 0 | 0 | 0 | 0 | 0 | 0 | 0 | 0 | 0 |
| LY96 | 4.64385618977473 | 1 [ 24 : 0 ] | 2 : 0 | 0 | 0 | 0 | 0 | 0 | 0 | 0 | 0 | 0 | 0 | 0 | 12 | 0 | 0 | 0 | 12 | 0 | 0 | 0 | 0 |
| MTERF4 | 4.64385618977473 | 1 [ 24 : 0 ] | 2 : 0 | 0 | 0 | 0 | 0 | 0 | 0 | 0 | 0 | 0 | 0 | 0 | 0 | 9 | 0 | 0 | 0 | 0 | 0 | 0 | 15 |
| RTTN | -4.64385618977473 | 2 [ 0 : 24 ] | 0 : 2 | 0 | 0 | 0 | 0 | 0 | 7 | 0 | 0 | 17 | 0 | 24 | 0 | 0 | 0 | 0 | 0 | 0 | 0 | 0 | 0 |
| ABCA13 | -4.58496250072116 | 2 [ 0 : 23 ] | 0 : 2 | 9 | 0 | 0 | 0 | 0 | 0 | 0 | 0 | 14 | 0 | 23 | 0 | 0 | 0 | 0 | 0 | 0 | 0 | 0 | 0 |
| AKAP2 | 4.52356195605701 | 2 [ 22 : 0 ] | 2 : 0 | 0 | 0 | 0 | 0 | 0 | 0 | 0 | 0 | 0 | 0 | 0 | 7 | 0 | 15 | 0 | 0 | 0 | 0 | 0 | 0 |
| FAM208B | 4.52356195605701 | 2 [ 22 : 0 ] | 2 : 0 | 0 | 0 | 0 | 0 | 0 | 0 | 0 | 0 | 0 | 0 | 0 | 0 | 0 | 7 | 0 | 0 | 0 | 0 | 0 | 15 |
| FANCA | -3.4594316186373 | 3 [ 1 : 21 ] | 1 : 2 | 0 | 0 | 0 | 0 | 0 | 0 | 8 | 0 | 0 | 13 | 21 | 0 | 0 | 1 | 0 | 0 | 0 | 0 | 0 | 0 |
| GPRC5C | -4.52356195605701 | 2 [ 0 : 22 ] | 0 : 2 | 0 | 0 | 0 | 0 | 0 | 0 | 9 | 0 | 0 | 13 | 22 | 0 | 0 | 0 | 0 | 0 | 0 | 0 | 0 | 0 |
| SLC22A18 | -4.52356195605701 | 1 [ 0 : 22 ] | 0 : 2 | 0 | 0 | 0 | 0 | 0 | 11 | 0 | 11 | 0 | 0 | 22 | 0 | 0 | 0 | 0 | 0 | 0 | 0 | 0 | 0 |
| ABCA1 | -4.4594316186373 | 2 [ 0 : 21 ] | 0 : 2 | 0 | 0 | 0 | 0 | 0 | 0 | 0 | 0 | 16 | 5 | 21 | 0 | 0 | 0 | 0 | 0 | 0 | 0 | 0 | 0 |
| MT-CYB | -1.67807190511264 | 3 [ 4 : 15 ] | 2 : 2 | 0 | 0 | 0 | 0 | 0 | 0 | 0 | 3 | 12 | 0 | 15 | 2 | 0 | 0 | 0 | 2 | 0 | 0 | 0 | 0 |
| TMEM241 | 4.32192809488736 | 2 [ 19 : 0 ] | 2 : 0 | 0 | 0 | 0 | 0 | 0 | 0 | 0 | 0 | 0 | 0 | 0 | 7 | 0 | 0 | 0 | 12 | 0 | 0 | 0 | 0 |
| ACOX3 | 4.24792751344359 | 2 [ 18 : 0 ] | 2 : 0 | 0 | 0 | 0 | 0 | 0 | 0 | 0 | 0 | 0 | 0 | 0 | 0 | 9 | 0 | 0 | 9 | 0 | 0 | 0 | 0 |
| LCE5A | 4.24792751344359 | 1 [ 18 : 0 ] | 2 : 0 | 0 | 0 | 0 | 0 | 0 | 0 | 0 | 0 | 0 | 0 | 0 | 9 | 0 | 0 | 0 | 9 | 0 | 0 | 0 | 0 |
| BIRC6 | 4.16992500144231 | 2 [ 17 : 0 ] | 2 : 0 | 0 | 0 | 0 | 0 | 0 | 0 | 0 | 0 | 0 | 0 | 0 | 0 | 0 | 0 | 0 | 0 | 9 | 0 | 0 | 8 |
| DCAF1 | 0.777607578663552 | 3 [ 11 : 6 ] | 1 : 2 | 0 | 0 | 0 | 0 | 3 | 0 | 3 | 0 | 0 | 0 | 6 | 0 | 0 | 0 | 0 | 0 | 11 | 0 | 0 | 0 |
| BAZ2B | 4.08746284125034 | 2 [ 16 : 0 ] | 2 : 0 | 0 | 0 | 0 | 0 | 0 | 0 | 0 | 0 | 0 | 0 | 0 | 0 | 0 | 0 | 0 | 0 | 0 | 13 | 3 | 0 |
| DVL3 | 4.08746284125034 | 1 [ 16 : 0 ] | 2 : 0 | 0 | 0 | 0 | 0 | 0 | 0 | 0 | 0 | 0 | 0 | 0 | 8 | 0 | 0 | 0 | 8 | 0 | 0 | 0 | 0 |
| HMCN1 | -4.08746284125034 | 2 [ 0 : 16 ] | 0 : 2 | 0 | 0 | 0 | 0 | 0 | 7 | 0 | 0 | 0 | 9 | 16 | 0 | 0 | 0 | 0 | 0 | 0 | 0 | 0 | 0 |
| PPP1R21 | 4.08746284125034 | 1 [ 16 : 0 ] | 2 : 0 | 0 | 0 | 0 | 0 | 0 | 0 | 0 | 0 | 0 | 0 | 0 | 8 | 0 | 0 | 0 | 8 | 0 | 0 | 0 | 0 |
| TMC4 | 4.08746284125034 | 2 [ 16 : 0 ] | 2 : 0 | 0 | 0 | 0 | 0 | 0 | 0 | 0 | 0 | 0 | 0 | 0 | 0 | 0 | 0 | 0 | 0 | 11 | 0 | 0 | 5 |
| NTN3 | -0.874469117916141 | 3 [ 5 : 10 ] | 1 : 2 | 0 | 0 | 0 | 0 | 0 | 0 | 9 | 0 | 1 | 0 | 10 | 0 | 0 | 5 | 0 | 0 | 0 | 0 | 0 | 0 |
