# Supplementary material for: Distinguishing and phenotype monitoring of traumatic brain injury and post-concussion syndrome including chronic migraine in serum of Iraq and Afghanistan war veterans
Source: PLoS One. 2019 Apr 26;14(4):e0215762. doi: 10.1371/journal.pone.0215762 (PMC6485717; doi:10.1371/journal.pone.0215762)
Supplement: S13 Table — (DOCX) [file pone.0215762.s039.docx]

**S13 Table. Peptides identified using MS/MS by patient TBI + CM vs TBI.**

| Symbol | IMLOG2 ratio: ((#Hits TBI with CM +1)/ (#Hits TBI only +1)) | #Unique Peptides ( TBI with Total Hits : TBI Total Hits ) | (#Sera TBI+CM : #sera TBI ) | TBI 19 | TBI 24 | TBI 28 | TBI 3 | TBI 31 | TBI 35 | TBI 39 | TBI 40 | TBI 43 | TBI 7 | TBI 11 | TBI 20 | TBI 21 | TBI 22 | TBI 25 | TBI 41 | TBI 42 | TBI 44 | TBI 45 | TBI 46 |
| --- | --- | --- | --- | --- | --- | --- | --- | --- | --- | --- | --- | --- | --- | --- | --- | --- | --- | --- | --- | --- | --- | --- | --- |
| IGH | -2.41503749927884 | 8 ( 5 : 31 ) | ( 2 : 5 ) | 0 | 0 | 0 | 0 | 0 | 3 | 2 | 0 | 0 | 0 | 0 | 0 | 0 | 0 | 14 | 4 | 3 | 7 | 0 | 3 |
| MUC19 | -0.106915203916512 | 6 ( 12 : 13 ) | ( 2 : 4 ) | 0 | 0 | 0 | 3 | 0 | 0 | 0 | 0 | 0 | 9 | 0 | 4 | 4 | 0 | 0 | 0 | 0 | 3 | 2 | 0 |
| EBF4 | 3.43295940727611 | 4 ( 107 : 9 ) | ( 2 : 3 ) | 32 | 0 | 75 | 0 | 0 | 0 | 0 | 0 | 0 | 0 | 3 | 3 | 3 | 0 | 0 | 0 | 0 | 0 | 0 | 0 |
| FBN2 | -2.56634682255381 | 4 ( 12 : 76 ) | ( 1 : 3 ) | 0 | 12 | 0 | 0 | 0 | 0 | 0 | 0 | 0 | 0 | 0 | 32 | 32 | 0 | 0 | 0 | 0 | 0 | 12 | 0 |
| MT-ND5 | 2.54748779530249 | 4 ( 75 : 12 ) | ( 1 : 3 ) | 0 | 0 | 0 | 75 | 0 | 0 | 0 | 0 | 0 | 0 | 0 | 2 | 2 | 8 | 0 | 0 | 0 | 0 | 0 | 0 |
| SYNE2 | 1.92599941855622 | 5 ( 75 : 19 ) | ( 3 : 2 ) | 0 | 0 | 13 | 39 | 0 | 23 | 0 | 0 | 0 | 0 | 0 | 0 | 0 | 0 | 12 | 0 | 0 | 0 | 0 | 7 |
| IGL | 0 | 4 ( 8 : 8 ) | ( 2 : 2 ) | 0 | 0 | 0 | 0 | 0 | 3 | 0 | 0 | 5 | 0 | 0 | 0 | 0 | 5 | 0 | 0 | 0 | 0 | 0 | 3 |
| TTN | 1.58496250072116 | 6 ( 41 : 13 ) | ( 3 : 1 ) | 0 | 0 | 0 | 5 | 0 | 24 | 0 | 12 | 0 | 0 | 0 | 0 | 0 | 0 | 0 | 13 | 0 | 0 | 0 | 0 |
| TNRC6B | -1.22239242133645 | 4 ( 14 : 34 ) | ( 2 : 3 ) | 0 | 3 | 0 | 0 | 0 | 0 | 0 | 11 | 0 | 0 | 0 | 0 | 0 | 6 | 0 | 25 | 0 | 0 | 3 | 0 |
| HUWE1 | -5.08746284125034 | 2 ( : 33 ) | ( 0 : 3 ) | 0 | 0 | 0 | 0 | 0 | 0 | 0 | 0 | 0 | 0 | 0 | 12 | 12 | 0 | 0 | 9 | 0 | 0 | 0 | 0 |
| CCDC148 | -0.906890595608519 | 3 ( 15 : 29 ) | ( 1 : 3 ) | 0 | 0 | 0 | 0 | 0 | 0 | 15 | 0 | 0 | 0 | 0 | 11 | 11 | 0 | 7 | 0 | 0 | 0 | 0 | 0 |
| RXFP1 | 4.4594316186373 | 3 ( 21 : ) | ( 3 : 0 ) | 0 | 11 | 0 | 0 | 0 | 0 | 0 | 8 | 2 | 0 | 0 | 0 | 0 | 0 | 0 | 0 | 0 | 0 | 0 | 0 |
| MUC5B | 4.08746284125034 | 3 ( 16 : ) | ( 3 : 0 ) | 0 | 5 | 0 | 0 | 0 | 0 | 0 | 0 | 7 | 4 | 0 | 0 | 0 | 0 | 0 | 0 | 0 | 0 | 0 | 0 |
| PCDH8 | -4.08746284125034 | 2 ( : 16 ) | ( 0 : 3 ) | 0 | 0 | 0 | 0 | 0 | 0 | 0 | 0 | 0 | 0 | 0 | 5 | 5 | 0 | 0 | 6 | 0 | 0 | 0 | 0 |
| TRB | -1.09953567355091 | 4 ( 6 : 14 ) | ( 2 : 3 ) | 0 | 0 | 0 | 0 | 3 | 0 | 0 | 0 | 3 | 0 | 0 | 4 | 4 | 0 | 6 | 0 | 0 | 0 | 0 | 0 |
| RUNX2 | -2.8073549220576 | 1 ( : 6 ) | ( 0 : 2 ) | 0 | 0 | 0 | 0 | 0 | 0 | 0 | 0 | 0 | 0 | 0 | 3 | 3 | 0 | 0 | 0 | 0 | 0 | 0 | 0 |
| MUC6 | -3.4594316186373 | 2 ( : 10 ) | ( 0 : 3 ) | 0 | 0 | 0 | 0 | 0 | 0 | 0 | 0 | 0 | 0 | 0 | 3 | 3 | 0 | 0 | 0 | 0 | 0 | 4 | 0 |
| LSM12 | -3.32192809488736 | 2 ( : 9 ) | ( 0 : 3 ) | 0 | 0 | 0 | 0 | 0 | 0 | 0 | 0 | 0 | 0 | 0 | 3 | 3 | 0 | 0 | 3 | 0 | 0 | 0 | 0 |
| AFF3 | -7.65105169117893 | 1 ( : 200 ) | ( 0 : 2 ) | 0 | 0 | 0 | 0 | 0 | 0 | 0 | 0 | 0 | 0 | 0 | 100 | 100 | 0 | 0 | 0 | 0 | 0 | 0 | 0 |
| TF | -6.04439411935845 | 1 ( : 65 ) | ( 0 : 1 ) | 0 | 0 | 0 | 0 | 0 | 0 | 0 | 0 | 0 | 0 | 0 | 0 | 0 | 0 | 65 | 0 | 0 | 0 | 0 | 0 |
| MT-ND1 | 2.03394733192334 | 6 ( 85 : 20 ) | ( 2 : 2 ) | 0 | 0 | 0 | 0 | 82 | 3 | 0 | 0 | 0 | 0 | 0 | 0 | 0 | 0 | 0 | 0 | 0 | 15 | 0 | 5 |
| PXN | 5.52356195605701 | 2 ( 45 : ) | ( 2 : 0 ) | 0 | 0 | 9 | 0 | 0 | 0 | 0 | 0 | 36 | 0 | 0 | 0 | 0 | 0 | 0 | 0 | 0 | 0 | 0 | 0 |
| MAPRE2 | -5.4262647547021 | 1 ( : 42 ) | ( 0 : 2 ) | 0 | 0 | 0 | 0 | 0 | 0 | 0 | 0 | 0 | 0 | 0 | 21 | 21 | 0 | 0 | 0 | 0 | 0 | 0 | 0 |
| LAMA1 | 2.8073549220576 | 1 ( 6 : ) | ( 1 : 0 ) | 0 | 0 | 0 | 0 | 0 | 0 | 0 | 0 | 0 | 6 | 0 | 0 | 0 | 0 | 0 | 0 | 0 | 0 | 0 | 0 |
| MSI2 | 5.39231742277876 | 2 ( 41 : ) | ( 2 : 0 ) | 0 | 0 | 0 | 0 | 0 | 0 | 33 | 0 | 8 | 0 | 0 | 0 | 0 | 0 | 0 | 0 | 0 | 0 | 0 | 0 |
| ING2 | -5.35755200461808 | 1 ( : 40 ) | ( 0 : 2 ) | 0 | 0 | 0 | 0 | 0 | 0 | 0 | 0 | 0 | 0 | 0 | 20 | 20 | 0 | 0 | 0 | 0 | 0 | 0 | 0 |
| PCLO | 5.28540221886225 | 3 ( 38 : ) | ( 2 : 0 ) | 0 | 0 | 0 | 0 | 0 | 18 | 20 | 0 | 0 | 0 | 0 | 0 | 0 | 0 | 0 | 0 | 0 | 0 | 0 | 0 |
| MEGF10 | 2.92599941855622 | 4 ( 37 : 4 ) | ( 2 : 1 ) | 0 | 32 | 0 | 0 | 0 | 0 | 0 | 0 | 0 | 5 | 0 | 0 | 0 | 4 | 0 | 0 | 0 | 0 | 0 | 0 |
| MAN1B1 | -5.20945336562895 | 1 ( : 36 ) | ( 0 : 2 ) | 0 | 0 | 0 | 0 | 0 | 0 | 0 | 0 | 0 | 0 | 0 | 18 | 18 | 0 | 0 | 0 | 0 | 0 | 0 | 0 |
| PRRC2A | 5.08746284125034 | 3 ( 33 : ) | ( 2 : 0 ) | 0 | 0 | 0 | 16 | 0 | 0 | 0 | 0 | 17 | 0 | 0 | 0 | 0 | 0 | 0 | 0 | 0 | 0 | 0 | 0 |
| DKC1 | -5.04439411935845 | 1 ( : 32 ) | ( 0 : 2 ) | 0 | 0 | 0 | 0 | 0 | 0 | 0 | 0 | 0 | 0 | 0 | 16 | 16 | 0 | 0 | 0 | 0 | 0 | 0 | 0 |
| KIAA2026 | 5.04439411935845 | 2 ( 32 : ) | ( 2 : 0 ) | 0 | 0 | 0 | 0 | 5 | 0 | 0 | 0 | 27 | 0 | 0 | 0 | 0 | 0 | 0 | 0 | 0 | 0 | 0 | 0 |
| ARID1B | -4.85798099512757 | 1 ( : 28 ) | ( 0 : 2 ) | 0 | 0 | 0 | 0 | 0 | 0 | 0 | 0 | 0 | 0 | 0 | 14 | 14 | 0 | 0 | 0 | 0 | 0 | 0 | 0 |
| RNF219 | 4.85798099512757 | 2 ( 28 : ) | ( 2 : 0 ) | 0 | 0 | 8 | 0 | 0 | 0 | 0 | 0 | 20 | 0 | 0 | 0 | 0 | 0 | 0 | 0 | 0 | 0 | 0 | 0 |
| FREM2 | -4.75488750216347 | 2 ( : 26 ) | ( 0 : 2 ) | 0 | 0 | 0 | 0 | 0 | 0 | 0 | 0 | 0 | 0 | 0 | 13 | 13 | 0 | 0 | 0 | 0 | 0 | 0 | 0 |
| CCDC18 | -4.64385618977473 | 1 ( : 24 ) | ( 0 : 2 ) | 0 | 0 | 0 | 0 | 0 | 0 | 0 | 0 | 0 | 0 | 0 | 12 | 12 | 0 | 0 | 0 | 0 | 0 | 0 | 0 |
| PADI3 | -4.64385618977473 | 1 ( : 24 ) | ( 0 : 2 ) | 0 | 0 | 0 | 0 | 0 | 0 | 0 | 0 | 0 | 0 | 0 | 12 | 12 | 0 | 0 | 0 | 0 | 0 | 0 | 0 |
| FRAS1 | 1.58496250072116 | 4 ( 23 : 7 ) | ( 2 : 1 ) | 0 | 0 | 0 | 0 | 0 | 11 | 0 | 12 | 0 | 0 | 0 | 0 | 0 | 0 | 0 | 0 | 0 | 0 | 7 | 0 |
| MYO15A | 4.58496250072116 | 2 ( 23 : ) | ( 2 : 0 ) | 21 | 0 | 0 | 0 | 0 | 0 | 2 | 0 | 0 | 0 | 0 | 0 | 0 | 0 | 0 | 0 | 0 | 0 | 0 | 0 |
| SLC39A14 | 4.58496250072116 | 2 ( 23 : ) | ( 2 : 0 ) | 9 | 0 | 0 | 0 | 0 | 0 | 0 | 14 | 0 | 0 | 0 | 0 | 0 | 0 | 0 | 0 | 0 | 0 | 0 | 0 |
| ZKSCAN7 | 4.58496250072116 | 2 ( 23 : ) | ( 2 : 0 ) | 0 | 0 | 0 | 0 | 6 | 17 | 0 | 0 | 0 | 0 | 0 | 0 | 0 | 0 | 0 | 0 | 0 | 0 | 0 | 0 |
| ZNF571 | 4.58496250072116 | 5 ( 23 : ) | ( 2 : 0 ) | 0 | 0 | 8 | 0 | 0 | 0 | 0 | 15 | 0 | 0 | 0 | 0 | 0 | 0 | 0 | 0 | 0 | 0 | 0 | 0 |
| ERBB3 | 4.52356195605701 | 2 ( 22 : ) | ( 2 : 0 ) | 15 | 0 | 0 | 0 | 0 | 0 | 0 | 0 | 0 | 7 | 0 | 0 | 0 | 0 | 0 | 0 | 0 | 0 | 0 | 0 |
| PSD3 | -4.52356195605701 | 1 ( : 22 ) | ( 0 : 2 ) | 0 | 0 | 0 | 0 | 0 | 0 | 0 | 0 | 0 | 0 | 0 | 11 | 11 | 0 | 0 | 0 | 0 | 0 | 0 | 0 |
| RP1 | -4.32192809488736 | 2 ( : 19 ) | ( 0 : 1 ) | 0 | 0 | 0 | 0 | 0 | 0 | 0 | 0 | 0 | 0 | 0 | 0 | 0 | 0 | 19 | 0 | 0 | 0 | 0 | 0 |
| SMYD3 | -1.13750352374994 | 2 ( 9 : 21 ) | ( 1 : 2 ) | 0 | 9 | 0 | 0 | 0 | 0 | 0 | 0 | 0 | 0 | 12 | 0 | 0 | 0 | 0 | 0 | 0 | 0 | 9 | 0 |
| DNAH9 | -4.39231742277876 | 2 ( : 20 ) | ( 0 : 2 ) | 0 | 0 | 0 | 0 | 0 | 0 | 0 | 0 | 0 | 0 | 12 | 0 | 0 | 0 | 0 | 0 | 0 | 0 | 0 | 8 |
| IGFN1 | 1.58496250072116 | 3 ( 20 : 6 ) | ( 1 : 2 ) | 0 | 0 | 0 | 0 | 20 | 0 | 0 | 0 | 0 | 0 | 2 | 0 | 0 | 0 | 4 | 0 | 0 | 0 | 0 | 0 |
| ITGB3 | -4.32192809488736 | 2 ( : 19 ) | ( 0 : 2 ) | 0 | 0 | 0 | 0 | 0 | 0 | 0 | 0 | 0 | 0 | 0 | 0 | 0 | 0 | 0 | 0 | 11 | 0 | 0 | 8 |
| RELN | 3.70043971814109 | 1 ( 12 : ) | ( 1 : 0 ) | 0 | 0 | 0 | 0 | 0 | 0 | 0 | 0 | 0 | 12 | 0 | 0 | 0 | 0 | 0 | 0 | 0 | 0 | 0 | 0 |
| SBF1 | -4.24792751344359 | 1 ( : 18 ) | ( 0 : 2 ) | 0 | 0 | 0 | 0 | 0 | 0 | 0 | 0 | 0 | 0 | 0 | 9 | 9 | 0 | 0 | 0 | 0 | 0 | 0 | 0 |
| ZNF91 | 4.24792751344359 | 3 ( 18 : ) | ( 2 : 0 ) | 8 | 0 | 0 | 0 | 0 | 0 | 0 | 10 | 0 | 0 | 0 | 0 | 0 | 0 | 0 | 0 | 0 | 0 | 0 | 0 |
| NOTCH4 | -0.502500340529183 | 4 ( 11 : 16 ) | ( 2 : 2 ) | 0 | 0 | 0 | 6 | 5 | 0 | 0 | 0 | 0 | 0 | 0 | 0 | 0 | 0 | 5 | 0 | 0 | 11 | 0 | 0 |
| PLOD1 | 4.08746284125034 | 2 ( 16 : ) | ( 2 : 0 ) | 0 | 11 | 0 | 0 | 0 | 0 | 5 | 0 | 0 | 0 | 0 | 0 | 0 | 0 | 0 | 0 | 0 | 0 | 0 | 0 |
| SLC12A1 | -4.08746284125034 | 1 ( : 16 ) | ( 0 : 2 ) | 0 | 0 | 0 | 0 | 0 | 0 | 0 | 0 | 0 | 0 | 0 | 8 | 8 | 0 | 0 | 0 | 0 | 0 | 0 | 0 |
| DUOX1 | 4 | 2 ( 15 : ) | ( 2 : 0 ) | 0 | 0 | 0 | 0 | 0 | 0 | 5 | 10 | 0 | 0 | 0 | 0 | 0 | 0 | 0 | 0 | 0 | 0 | 0 | 0 |
| POU4F3 | 0.540568381362703 | 2 ( 15 : 10 ) | ( 2 : 1 ) | 0 | 10 | 0 | 0 | 0 | 5 | 0 | 0 | 0 | 0 | 0 | 0 | 0 | 0 | 0 | 0 | 0 | 0 | 10 | 0 |
| TANC1 | -4 | 2 ( : 15 ) | ( 0 : 2 ) | 0 | 0 | 0 | 0 | 0 | 0 | 0 | 0 | 0 | 0 | 0 | 0 | 0 | 0 | 9 | 6 | 0 | 0 | 0 | 0 |
| EGF | 3.90689059560852 | 2 ( 14 : ) | ( 2 : 0 ) | 9 | 0 | 0 | 0 | 0 | 0 | 0 | 0 | 0 | 5 | 0 | 0 | 0 | 0 | 0 | 0 | 0 | 0 | 0 | 0 |
| LRP1 | -1.58496250072116 | 4 ( 4 : 14 ) | ( 1 : 2 ) | 0 | 0 | 4 | 0 | 0 | 0 | 0 | 0 | 0 | 0 | 0 | 0 | 0 | 0 | 8 | 0 | 0 | 0 | 0 | 6 |
| PLP1 | 3.90689059560852 | 2 ( 14 : ) | ( 2 : 0 ) | 0 | 0 | 0 | 0 | 0 | 0 | 0 | 0 | 7 | 7 | 0 | 0 | 0 | 0 | 0 | 0 | 0 | 0 | 0 | 0 |
| STAT1 | -3.90689059560852 | 1 ( : 14 ) | ( 0 : 2 ) | 0 | 0 | 0 | 0 | 0 | 0 | 0 | 0 | 0 | 0 | 0 | 7 | 7 | 0 | 0 | 0 | 0 | 0 | 0 | 0 |
| TENM1 | -1.90689059560852 | 2 ( 3 : 14 ) | ( 1 : 2 ) | 0 | 0 | 0 | 0 | 0 | 0 | 3 | 0 | 0 | 0 | 0 | 7 | 7 | 0 | 0 | 0 | 0 | 0 | 0 | 0 |
| ZBED4 | -3.90689059560852 | 2 ( : 14 ) | ( 0 : 2 ) | 0 | 0 | 0 | 0 | 0 | 0 | 0 | 0 | 0 | 0 | 7 | 0 | 0 | 0 | 7 | 0 | 0 | 0 | 0 | 0 |
| ABCG5 | -3.70043971814109 | 1 ( : 12 ) | ( 0 : 2 ) | 0 | 0 | 0 | 0 | 0 | 0 | 0 | 0 | 0 | 0 | 0 | 6 | 6 | 0 | 0 | 0 | 0 | 0 | 0 | 0 |
| CACHD1 | -3.70043971814109 | 2 ( : 12 ) | ( 0 : 2 ) | 0 | 0 | 0 | 0 | 0 | 0 | 0 | 0 | 0 | 0 | 0 | 0 | 0 | 0 | 3 | 0 | 0 | 0 | 9 | 0 |
| CACNA2D1 | -3.70043971814109 | 2 ( : 12 ) | ( 0 : 2 ) | 0 | 0 | 0 | 0 | 0 | 0 | 0 | 0 | 0 | 0 | 0 | 6 | 6 | 0 | 0 | 0 | 0 | 0 | 0 | 0 |
| MCRS1 | -3.70043971814109 | 1 ( : 12 ) | ( 0 : 2 ) | 0 | 0 | 0 | 0 | 0 | 0 | 0 | 0 | 0 | 0 | 0 | 6 | 6 | 0 | 0 | 0 | 0 | 0 | 0 | 0 |
| MIPOL1 | -3.70043971814109 | 1 ( : 12 ) | ( 0 : 2 ) | 0 | 0 | 0 | 0 | 0 | 0 | 0 | 0 | 0 | 0 | 0 | 6 | 6 | 0 | 0 | 0 | 0 | 0 | 0 | 0 |
| MT1M | 3.70043971814109 | 2 ( 12 : ) | ( 2 : 0 ) | 0 | 0 | 0 | 0 | 5 | 7 | 0 | 0 | 0 | 0 | 0 | 0 | 0 | 0 | 0 | 0 | 0 | 0 | 0 | 0 |
| PRAG1 | 3.70043971814109 | 2 ( 12 : ) | ( 2 : 0 ) | 0 | 0 | 0 | 0 | 0 | 0 | 9 | 3 | 0 | 0 | 0 | 0 | 0 | 0 | 0 | 0 | 0 | 0 | 0 | 0 |
| REEP6 | -3.70043971814109 | 1 ( : 12 ) | ( 0 : 2 ) | 0 | 0 | 0 | 0 | 0 | 0 | 0 | 0 | 0 | 0 | 0 | 6 | 6 | 0 | 0 | 0 | 0 | 0 | 0 | 0 |
| SLC25A40 | -3.70043971814109 | 1 ( : 12 ) | ( 0 : 2 ) | 0 | 0 | 0 | 0 | 0 | 0 | 0 | 0 | 0 | 0 | 0 | 6 | 6 | 0 | 0 | 0 | 0 | 0 | 0 | 0 |
| VLDLR | -3.70043971814109 | 2 ( : 12 ) | ( 0 : 2 ) | 0 | 0 | 0 | 0 | 0 | 0 | 0 | 0 | 0 | 0 | 0 | 6 | 6 | 0 | 0 | 0 | 0 | 0 | 0 | 0 |
| CRCT1 | 3.58496250072116 | 2 ( 11 : ) | ( 2 : 0 ) | 0 | 0 | 0 | 0 | 9 | 2 | 0 | 0 | 0 | 0 | 0 | 0 | 0 | 0 | 0 | 0 | 0 | 0 | 0 | 0 |
| FBN1 | -2 | 4 ( 2 : 11 ) | ( 1 : 2 ) | 0 | 0 | 0 | 2 | 0 | 0 | 0 | 0 | 0 | 0 | 5 | 0 | 0 | 0 | 0 | 0 | 0 | 0 | 0 | 6 |
| SVEP1 | -0.415037499278844 | 2 ( 8 : 11 ) | ( 1 : 2 ) | 0 | 8 | 0 | 0 | 0 | 0 | 0 | 0 | 0 | 0 | 0 | 0 | 0 | 0 | 0 | 0 | 3 | 0 | 8 | 0 |
| WDHD1 | 1 | 2 ( 11 : 5 ) | ( 1 : 1 ) | 0 | 0 | 0 | 0 | 0 | 0 | 0 | 0 | 11 | 0 | 0 | 0 | 0 | 0 | 0 | 5 | 0 | 0 | 0 | 0 |
| C7 | -0.459431618637297 | 2 ( 7 : 10 ) | ( 1 : 2 ) | 0 | 0 | 0 | 7 | 0 | 0 | 0 | 0 | 0 | 0 | 0 | 5 | 5 | 0 | 0 | 0 | 0 | 0 | 0 | 0 |
| CA3 | -3.4594316186373 | 1 ( : 10 ) | ( 0 : 2 ) | 0 | 0 | 0 | 0 | 0 | 0 | 0 | 0 | 0 | 0 | 0 | 5 | 5 | 0 | 0 | 0 | 0 | 0 | 0 | 0 |
| CTSW | 0.459431618637297 | 2 ( 10 : 7 ) | ( 2 : 1 ) | 0 | 7 | 0 | 0 | 3 | 0 | 0 | 0 | 0 | 0 | 0 | 0 | 0 | 0 | 0 | 0 | 0 | 0 | 7 | 0 |
| DNAH14 | -3.4594316186373 | 1 ( : 10 ) | ( 0 : 2 ) | 0 | 0 | 0 | 0 | 0 | 0 | 0 | 0 | 0 | 0 | 0 | 5 | 5 | 0 | 0 | 0 | 0 | 0 | 0 | 0 |
| EPS8 | -3.4594316186373 | 1 ( : 10 ) | ( 0 : 2 ) | 0 | 0 | 0 | 0 | 0 | 0 | 0 | 0 | 0 | 0 | 0 | 5 | 5 | 0 | 0 | 0 | 0 | 0 | 0 | 0 |
| SH2D7 | -3.4594316186373 | 2 ( : 10 ) | ( 0 : 2 ) | 0 | 0 | 0 | 0 | 0 | 0 | 0 | 0 | 0 | 0 | 2 | 0 | 0 | 8 | 0 | 0 | 0 | 0 | 0 | 0 |
| SLAMF7 | -3.4594316186373 | 1 ( : 10 ) | ( 0 : 2 ) | 0 | 0 | 0 | 0 | 0 | 0 | 0 | 0 | 0 | 0 | 0 | 5 | 5 | 0 | 0 | 0 | 0 | 0 | 0 | 0 |
| SLC23A1 | -3.4594316186373 | 1 ( : 10 ) | ( 0 : 2 ) | 0 | 0 | 0 | 0 | 0 | 0 | 0 | 0 | 0 | 0 | 0 | 5 | 5 | 0 | 0 | 0 | 0 | 0 | 0 | 0 |
| TECPR1 | -3.4594316186373 | 1 ( : 10 ) | ( 0 : 2 ) | 0 | 0 | 0 | 0 | 0 | 0 | 0 | 0 | 0 | 0 | 0 | 5 | 5 | 0 | 0 | 0 | 0 | 0 | 0 | 0 |
| UBR7 | -3.4594316186373 | 1 ( : 10 ) | ( 0 : 2 ) | 0 | 0 | 0 | 0 | 0 | 0 | 0 | 0 | 0 | 0 | 0 | 5 | 5 | 0 | 0 | 0 | 0 | 0 | 0 | 0 |
| PAPPA | 3.32192809488736 | 2 ( 9 : ) | ( 2 : 0 ) | 0 | 0 | 0 | 2 | 0 | 0 | 0 | 0 | 7 | 0 | 0 | 0 | 0 | 0 | 0 | 0 | 0 | 0 | 0 | 0 |
| VWDE | 3.32192809488736 | 2 ( 9 : ) | ( 2 : 0 ) | 0 | 0 | 4 | 0 | 0 | 0 | 0 | 5 | 0 | 0 | 0 | 0 | 0 | 0 | 0 | 0 | 0 | 0 | 0 | 0 |
| ZFHX3 | -3.32192809488736 | 2 ( : 9 ) | ( 0 : 2 ) | 0 | 0 | 0 | 0 | 0 | 0 | 0 | 0 | 0 | 0 | 0 | 0 | 0 | 4 | 0 | 5 | 0 | 0 | 0 | 0 |
| FBXO34 | -3.16992500144231 | 1 ( : 8 ) | ( 0 : 2 ) | 0 | 0 | 0 | 0 | 0 | 0 | 0 | 0 | 0 | 0 | 0 | 4 | 4 | 0 | 0 | 0 | 0 | 0 | 0 | 0 |
| GLRB | -3.16992500144231 | 1 ( : 8 ) | ( 0 : 2 ) | 0 | 0 | 0 | 0 | 0 | 0 | 0 | 0 | 0 | 0 | 0 | 4 | 4 | 0 | 0 | 0 | 0 | 0 | 0 | 0 |
| LRP2 | 3.16992500144231 | 2 ( 8 : ) | ( 2 : 0 ) | 0 | 0 | 0 | 0 | 0 | 3 | 0 | 0 | 5 | 0 | 0 | 0 | 0 | 0 | 0 | 0 | 0 | 0 | 0 | 0 |
| NFASC | -3.16992500144231 | 1 ( : 8 ) | ( 0 : 2 ) | 0 | 0 | 0 | 0 | 0 | 0 | 0 | 0 | 0 | 0 | 0 | 4 | 4 | 0 | 0 | 0 | 0 | 0 | 0 | 0 |
| NSD3 | -3.16992500144231 | 1 ( : 8 ) | ( 0 : 2 ) | 0 | 0 | 0 | 0 | 0 | 0 | 0 | 0 | 0 | 0 | 0 | 4 | 4 | 0 | 0 | 0 | 0 | 0 | 0 | 0 |
| PKD1 | -3.16992500144231 | 1 ( : 8 ) | ( 0 : 2 ) | 0 | 0 | 0 | 0 | 0 | 0 | 0 | 0 | 0 | 0 | 0 | 4 | 4 | 0 | 0 | 0 | 0 | 0 | 0 | 0 |
| ZNF646 | -3.16992500144231 | 2 ( : 8 ) | ( 0 : 2 ) | 0 | 0 | 0 | 0 | 0 | 0 | 0 | 0 | 0 | 0 | 0 | 0 | 0 | 3 | 0 | 0 | 5 | 0 | 0 | 0 |
| MUC17 | 0.192645077942396 | 2 ( 7 : 6 ) | ( 1 : 2 ) | 0 | 0 | 7 | 0 | 0 | 0 | 0 | 0 | 0 | 0 | 0 | 3 | 3 | 0 | 0 | 0 | 0 | 0 | 0 | 0 |
| ZNF879 | 3 | 2 ( 7 : ) | ( 2 : 0 ) | 0 | 0 | 0 | 0 | 0 | 4 | 0 | 0 | 0 | 3 | 0 | 0 | 0 | 0 | 0 | 0 | 0 | 0 | 0 | 0 |
| ATP9B | 2.8073549220576 | 1 ( 6 : ) | ( 2 : 0 ) | 3 | 0 | 0 | 0 | 0 | 0 | 0 | 0 | 3 | 0 | 0 | 0 | 0 | 0 | 0 | 0 | 0 | 0 | 0 | 0 |
| CCER1 | -2.8073549220576 | 1 ( : 6 ) | ( 0 : 2 ) | 0 | 0 | 0 | 0 | 0 | 0 | 0 | 0 | 0 | 0 | 0 | 3 | 3 | 0 | 0 | 0 | 0 | 0 | 0 | 0 |
| IQCN | 2.8073549220576 | 2 ( 6 : ) | ( 2 : 0 ) | 0 | 0 | 0 | 0 | 0 | 0 | 0 | 3 | 3 | 0 | 0 | 0 | 0 | 0 | 0 | 0 | 0 | 0 | 0 | 0 |
| LOC646383 | -2.8073549220576 | 1 ( : 6 ) | ( 0 : 2 ) | 0 | 0 | 0 | 0 | 0 | 0 | 0 | 0 | 0 | 0 | 0 | 3 | 3 | 0 | 0 | 0 | 0 | 0 | 0 | 0 |
| MDFIC | -2.8073549220576 | 1 ( : 6 ) | ( 0 : 2 ) | 0 | 0 | 0 | 0 | 0 | 0 | 0 | 0 | 0 | 0 | 0 | 3 | 3 | 0 | 0 | 0 | 0 | 0 | 0 | 0 |
| MTO1 | 2.8073549220576 | 2 ( 6 : ) | ( 2 : 0 ) | 0 | 0 | 3 | 0 | 3 | 0 | 0 | 0 | 0 | 0 | 0 | 0 | 0 | 0 | 0 | 0 | 0 | 0 | 0 | 0 |
| MUC4 | -2.8073549220576 | 1 ( : 6 ) | ( 0 : 2 ) | 0 | 0 | 0 | 0 | 0 | 0 | 0 | 0 | 0 | 0 | 0 | 3 | 3 | 0 | 0 | 0 | 0 | 0 | 0 | 0 |
| NFKB2 | -2.8073549220576 | 1 ( : 6 ) | ( 0 : 2 ) | 0 | 0 | 0 | 0 | 0 | 0 | 0 | 0 | 0 | 0 | 0 | 3 | 3 | 0 | 0 | 0 | 0 | 0 | 0 | 0 |
| TBX22 | -2.8073549220576 | 1 ( : 6 ) | ( 0 : 2 ) | 0 | 0 | 0 | 0 | 0 | 0 | 0 | 0 | 0 | 0 | 0 | 3 | 3 | 0 | 0 | 0 | 0 | 0 | 0 | 0 |
| TNFRSF14 | -1.22239242133645 | 2 ( 2 : 6 ) | ( 1 : 2 ) | 0 | 0 | 0 | 0 | 0 | 0 | 2 | 0 | 0 | 0 | 0 | 3 | 3 | 0 | 0 | 0 | 0 | 0 | 0 | 0 |
| WDR46 | -0.222392421336448 | 3 ( 5 : 6 ) | ( 1 : 2 ) | 0 | 0 | 0 | 5 | 0 | 0 | 0 | 0 | 0 | 0 | 0 | 0 | 0 | 4 | 0 | 0 | 0 | 2 | 0 | 0 |
| KCNK18 | -2.58496250072116 | 3 ( : 5 ) | ( 0 : 2 ) | 0 | 0 | 0 | 0 | 0 | 0 | 0 | 0 | 0 | 0 | 0 | 0 | 0 | 2 | 0 | 0 | 0 | 3 | 0 | 0 |
