# Supplementary material for: Distinguishing and phenotype monitoring of traumatic brain injury and post-concussion syndrome including chronic migraine in serum of Iraq and Afghanistan war veterans
Source: PLoS One. 2019 Apr 26;14(4):e0215762. doi: 10.1371/journal.pone.0215762 (PMC6485717; doi:10.1371/journal.pone.0215762)
Supplement: S14 Table — (DOCX) [file pone.0215762.s040.docx]

**S14 Table. Proteins identified MSMS analysis TBI (most affected, N=10) vs control (least affected, N=10) MS/MS results.**

| Symbol | Sequence_mod | Xcorr_mod | pathology | deid_number |
| --- | --- | --- | --- | --- |
| ABCA1 | ALDVGLPSsKLKSKTSQ | 2.221044 | Control(minimal) | control 12 |
| ABCA1 | ALDVGLPSsKLKSKTSQ | 2.221044 | Control(minimal) | control 12 |
| ABCA1 | ALDVGLPSsKLKSKTSQ | 2.221044 | Control(minimal) | control 12 |
| ABCA1 | ALDVGLPsSKLKSKTSQ | 2.221044 | Control(minimal) | control 12 |
| ABCA1 | ALDVGLPsSKLKSKTSQ | 2.221044 | Control(minimal) | control 12 |
| ABCA1 | ALDVGLPSsKLKSKTSQ | 2.221044 | Control(minimal) | control 12 |
| ABCA1 | ALDVGLPSsKLKSKTSQ | 2.221044 | Control(minimal) | control 12 |
| ABCA1 | ALDVGLPsSKLKSKTSQ | 2.221044 | Control(minimal) | control 12 |
| ABCA1 | ALDVGLPSsKLKSKTSQ | 2.221044 | Control(minimal) | control 12 |
| ABCA1 | ALDVGLPSsKLKSKTSQ | 2.221044 | Control(minimal) | control 12 |
| ABCA1 | ALDVGLPsSKLKSKTSQ | 2.221044 | Control(minimal) | control 12 |
| ABCA1 | ALDVGLPsSKLKSKTSQ | 1.863953 | Control(minimal) | control 12 |
| ABCA1 | ALDVGLPsSKLKSKTSQ | 1.863953 | Control(minimal) | control 12 |
| ABCA1 | ALDVGLPsSKLKSKTSQ | 1.863953 | Control(minimal) | control 12 |
| ABCA1 | ALDVGLPsSKLKSKTSQ | 1.863953 | Control(minimal) | control 12 |
| ABCA1 | ALDVGLPsSKLKSKTSQ | 1.863953 | Control(minimal) | control 12 |
| ABCA1 | ADILQDLtGRNISDYL | 1.834556 | Control(minimal) | control 13 |
| ABCA1 | ADILQDLtGRNISDYL | 1.834556 | Control(minimal) | control 13 |
| ABCA1 | ADILQDLtGRNISDYL | 1.834556 | Control(minimal) | control 13 |
| ABCA1 | ADILQDLtGRNISDYL | 1.834556 | Control(minimal) | control 13 |
| ABCA1 | ADILQDLtGRNISDYL | 1.834556 | Control(minimal) | control 13 |
| ABCA13 | VAENPsWTKDILCAT | 1.700052 | Control(minimal) | control 3 |
| ABCA13 | VAENPsWTKDILCAT | 1.700052 | Control(minimal) | control 3 |
| ABCA13 | VAENPsWTKDILCAT | 1.700052 | Control(minimal) | control 3 |
| ABCA13 | VAENPsWTKDILCAT | 1.700052 | Control(minimal) | control 3 |
| ABCA13 | VAENPsWTKDILCAT | 1.700052 | Control(minimal) | control 3 |
| ABCA13 | VAENPsWTKDILCAT | 1.700052 | Control(minimal) | control 3 |
| ABCA13 | VAENPsWTKDILCAT | 1.700052 | Control(minimal) | control 3 |
| ABCA13 | VAENPsWTKDILCAT | 1.700052 | Control(minimal) | control 3 |
| ABCA13 | VAENPsWTKDILCAT | 1.700052 | Control(minimal) | control 3 |
| ABCA13 | StEGQELEVIHTTLTGL | 2.284242 | Control(minimal) | control 12 |
| ABCA13 | StEGQELEVIHTTLTGL | 2.284242 | Control(minimal) | control 12 |
| ABCA13 | StEGQELEVIHTTLTGL | 2.284242 | Control(minimal) | control 12 |
| ABCA13 | StEGQELEVIHTTLTGL | 2.284242 | Control(minimal) | control 12 |
| ABCA13 | StEGQELEVIHTTLTGL | 2.284242 | Control(minimal) | control 12 |
| ABCA13 | sTEGQELEVIHTTLTGL | 2.284242 | Control(minimal) | control 12 |
| ABCA13 | sTEGQELEVIHTTLTGL | 2.284242 | Control(minimal) | control 12 |
| ABCA13 | sTEGQELEVIHTTLTGL | 2.284242 | Control(minimal) | control 12 |
| ABCA13 | sTEGQELEVIHTTLTGL | 2.284242 | Control(minimal) | control 12 |
| ABCA13 | StEGQELEVIHTTLTGL | 2.284242 | Control(minimal) | control 12 |
| ABCA13 | sTEGQELEVIHTTLTGL | 2.284242 | Control(minimal) | control 12 |
| ABCA13 | sTEGQELEVIHTTLTGL | 2.078189 | Control(minimal) | control 12 |
| ABCA13 | sTEGQELEVIHTTLTGL | 2.078189 | Control(minimal) | control 12 |
| ABCA13 | sTEGQELEVIHTTLTGL | 2.078189 | Control(minimal) | control 12 |
| ACOX3 | EARNKcQVsHGRPL | 1.883704 | TBI(extreme) | TBI 2 |
| ACOX3 | EARNKcQVsHGRPL | 1.883704 | TBI(extreme) | TBI 2 |
| ACOX3 | EARNKcQVsHGRPL | 1.883704 | TBI(extreme) | TBI 2 |
| ACOX3 | EARNKcQVsHGRPL | 1.883704 | TBI(extreme) | TBI 2 |
| ACOX3 | EARNKcQVsHGRPL | 1.883704 | TBI(extreme) | TBI 2 |
| ACOX3 | EARNKcQVsHGRPL | 1.883704 | TBI(extreme) | TBI 2 |
| ACOX3 | EARNKcQVsHGRPL | 1.883704 | TBI(extreme) | TBI 2 |
| ACOX3 | EARNKcQVsHGRPL | 1.883704 | TBI(extreme) | TBI 2 |
| ACOX3 | EARNKcQVsHGRPL | 1.883704 | TBI(extreme) | TBI 2 |
| ACOX3 | NCCmFCAGSYHAMAs | 1.869164 | TBI(extreme) | TBI 8 |
| ACOX3 | NCCmFCAGSYHAMAs | 1.869164 | TBI(extreme) | TBI 8 |
| ACOX3 | NCCmFCAGSYHAMAs | 1.869164 | TBI(extreme) | TBI 8 |
| ACOX3 | NCCmFCAGSyHAMAS | 1.869164 | TBI(extreme) | TBI 8 |
| ACOX3 | NCCmFCAGSYHAMAs | 1.869164 | TBI(extreme) | TBI 8 |
| ACOX3 | NCCmFCAGSyHAMAS | 1.869164 | TBI(extreme) | TBI 8 |
| ACOX3 | NCCmFCAGSYHAMAs | 1.869164 | TBI(extreme) | TBI 8 |
| ACOX3 | NCCmFCAGSyHAMAS | 1.869164 | TBI(extreme) | TBI 8 |
| ACOX3 | NCCmFCAGSYHAMAs | 1.869164 | TBI(extreme) | TBI 8 |
| ADGRL2 | RHSYccGGLPTES | 1.86337 | Control(minimal) | control 12 |
| ADGRL2 | RHSYccGGLPTES | 1.86337 | Control(minimal) | control 12 |
| ADGRL2 | RHSYccGGLPTES | 1.86337 | Control(minimal) | control 12 |
| ADGRL2 | RHSYccGGLPTES | 1.86337 | Control(minimal) | control 12 |
| ADGRL2 | RHSYccGGLPTES | 1.86337 | Control(minimal) | control 12 |
| ADGRL2 | RHSYccGGLPTES | 1.86337 | Control(minimal) | control 12 |
| ADGRL2 | RHSYccGGLPTES | 1.86337 | Control(minimal) | control 12 |
| ADGRL2 | RHSYccGGLPTES | 1.86337 | Control(minimal) | control 12 |
| ADGRL2 | RHSYccGGLPTES | 1.86337 | Control(minimal) | control 12 |
| ADGRL2 | RHSYccGGLPTES | 1.86337 | Control(minimal) | control 12 |
| ADGRL2 | RHSYccGGLPTES | 1.86337 | Control(minimal) | control 12 |
| ADGRL2 | RHSYccGGLPTES | 1.86337 | Control(minimal) | control 12 |
| ADGRL2 | RHSYccGGLPTES | 1.86337 | Control(minimal) | control 12 |
| ADGRL2 | RHSYccGGLPTES | 1.86337 | Control(minimal) | control 12 |
| ADGRL2 | RHSYccGGLPTES | 1.86337 | Control(minimal) | control 12 |
| ADGRL2 | RHSYccGGLPTES | 1.86337 | Control(minimal) | control 12 |
| ADGRL2 | RHSYccGGLPTES | 1.86337 | Control(minimal) | control 12 |
| ADGRL2 | RHSYccGGLPTES | 1.86337 | Control(minimal) | control 12 |
| ADGRL2 | RHSYccGGLPTES | 1.86337 | Control(minimal) | control 12 |
| ADGRL2 | RHSYccGGLPTES | 1.86337 | Control(minimal) | control 12 |
| ADGRL2 | RHSYccGGLPTES | 1.86337 | Control(minimal) | control 12 |
| ADGRL2 | RHSYccGGLPTES | 1.86337 | Control(minimal) | control 12 |
| ADGRL2 | RHSYccGGLPTES | 1.86337 | Control(minimal) | control 12 |
| ADGRL2 | RHSYccGGLPTES | 1.86337 | Control(minimal) | control 12 |
| ADGRL2 | RHSYccGGLPTES | 1.86337 | Control(minimal) | control 12 |
| ADGRL2 | RHSYccGGLPTES | 1.86337 | Control(minimal) | control 12 |
| ADGRL2 | RHSYccGGLPTES | 1.86337 | Control(minimal) | control 12 |
| ADGRL2 | RHSYccGGLPTES | 1.86337 | Control(minimal) | control 12 |
| ADGRL2 | RHSYccGGLPTES | 1.86337 | Control(minimal) | control 12 |
| ADGRL2 | RHSYccGGLPTES | 1.86337 | Control(minimal) | control 12 |
| ADGRL2 | RHSYccGGLPTES | 1.86337 | Control(minimal) | control 12 |
| ADGRL2 | RHSYccGGLPTES | 1.86337 | Control(minimal) | control 12 |
| ADGRL2 | RHSYccGGLPTES | 1.86337 | Control(minimal) | control 12 |
| ADGRL2 | RHSYccGGLPTES | 1.86337 | Control(minimal) | control 12 |
| ADGRL2 | RHSYccGGLPTES | 1.86337 | Control(minimal) | control 12 |
| ADGRL2 | RHSYccGGLPTES | 1.86337 | Control(minimal) | control 12 |
| ADGRL2 | RHSYccGGLPTES | 1.86337 | Control(minimal) | control 12 |
| ADGRL2 | RHSYccGGLPTES | 1.86337 | Control(minimal) | control 12 |
| ADGRL2 | RHSYccGGLPTES | 1.86337 | Control(minimal) | control 12 |
| ADGRL2 | RHSYccGGLPTES | 1.86337 | Control(minimal) | control 12 |
| ADGRL2 | RHSYccGGLPTES | 1.86337 | Control(minimal) | control 12 |
| ADGRL2 | RHSYccGGLPTES | 1.86337 | Control(minimal) | control 12 |
| ADGRL2 | RHSYccGGLPTES | 1.86337 | Control(minimal) | control 12 |
| ADGRL2 | RHSYccGGLPTES | 1.86337 | Control(minimal) | control 12 |
| ADGRL2 | RHSYccGGLPTES | 1.86337 | Control(minimal) | control 12 |
| ADGRL2 | RHSYccGGLPTES | 1.86337 | Control(minimal) | control 12 |
| ADGRL2 | RHSYccGGLPTES | 1.86337 | Control(minimal) | control 12 |
| ADGRL2 | RHSYccGGLPTES | 1.86337 | Control(minimal) | control 12 |
| ADGRL2 | RHSYccGGLPTES | 1.86337 | Control(minimal) | control 12 |
| ADGRL2 | RHSYccGGLPTES | 1.86337 | Control(minimal) | control 12 |
| ADGRL2 | RHSYccGGLPTES | 1.86337 | Control(minimal) | control 12 |
| ADGRL2 | RHSYccGGLPTES | 1.86337 | Control(minimal) | control 12 |
| ADGRL2 | RHSYccGGLPTES | 1.86337 | Control(minimal) | control 12 |
| ADGRL2 | RHSYccGGLPTES | 1.86337 | Control(minimal) | control 12 |
| ADGRL2 | RHSYccGGLPTES | 1.86337 | Control(minimal) | control 12 |
| ADGRL2 | RHSYccGGLPTES | 1.86337 | Control(minimal) | control 12 |
| ADGRL2 | RHSYccGGLPTES | 1.86337 | Control(minimal) | control 12 |
| ADGRL2 | RHSYccGGLPTES | 1.86337 | Control(minimal) | control 12 |
| ADGRL2 | RHSYccGGLPTES | 1.86337 | Control(minimal) | control 12 |
| ADGRL2 | RHSYccGGLPTES | 1.86337 | Control(minimal) | control 12 |
| ADGRL2 | RHSYccGGLPTES | 1.86337 | Control(minimal) | control 12 |
| ADGRL2 | RHSYccGGLPTES | 1.86337 | Control(minimal) | control 12 |
| ADGRL2 | RHSYccGGLPTES | 1.86337 | Control(minimal) | control 12 |
| ADGRL2 | RHSYccGGLPTES | 1.86337 | Control(minimal) | control 12 |
| ADGRL2 | RHSYccGGLPTES | 1.86337 | Control(minimal) | control 12 |
| ADGRL2 | RHSYccGGLPTES | 1.86337 | Control(minimal) | control 12 |
| ADGRL2 | RHSYccGGLPTES | 1.86337 | Control(minimal) | control 12 |
| ADGRL2 | RHSYccGGLPTES | 1.86337 | Control(minimal) | control 12 |
| ADGRL2 | RHSYccGGLPTES | 1.86337 | Control(minimal) | control 12 |
| ADGRL2 | RHSYccGGLPTES | 1.86337 | Control(minimal) | control 12 |
| ADGRL2 | RHSYccGGLPTES | 1.86337 | Control(minimal) | control 12 |
| ADGRL2 | RHSYccGGLPTES | 1.86337 | Control(minimal) | control 12 |
| ADGRL2 | RHSYccGGLPTES | 1.86337 | Control(minimal) | control 12 |
| ADGRL2 | RHSYccGGLPTES | 1.86337 | Control(minimal) | control 12 |
| ADGRL2 | RHSYccGGLPTES | 1.86337 | Control(minimal) | control 12 |
| ADGRL2 | RHSYccGGLPTES | 1.86337 | Control(minimal) | control 12 |
| ADGRL2 | RHSYccGGLPTES | 1.86337 | Control(minimal) | control 12 |
| ADGRL2 | RHSYccGGLPTES | 1.86337 | Control(minimal) | control 12 |
| ADGRL2 | RHSYccGGLPTES | 1.86337 | Control(minimal) | control 12 |
| ADGRL2 | RHSYccGGLPTES | 1.86337 | Control(minimal) | control 12 |
| ADGRL2 | RHSYccGGLPTES | 1.86337 | Control(minimal) | control 12 |
| ADGRL2 | RHSYccGGLPTES | 1.86337 | Control(minimal) | control 12 |
| ADGRL2 | RHSYccGGLPTES | 1.86337 | Control(minimal) | control 12 |
| ADGRL2 | RHSYccGGLPTES | 1.86337 | Control(minimal) | control 12 |
| ADGRL2 | RHSYccGGLPTES | 1.86337 | Control(minimal) | control 12 |
| ADGRL2 | RHSYccGGLPTES | 1.752844 | Control(minimal) | control 12 |
| ADGRL2 | RHSYccGGLPTES | 1.752844 | Control(minimal) | control 12 |
| ADGRL2 | RHSYccGGLPTES | 1.752844 | Control(minimal) | control 12 |
| ADGRL2 | RHSYccGGLPTES | 1.752844 | Control(minimal) | control 12 |
| ADGRL2 | RHSYccGGLPTES | 1.752844 | Control(minimal) | control 12 |
| ADGRL2 | RHSYccGGLPTES | 1.752844 | Control(minimal) | control 12 |
| ADGRL2 | RHSYccGGLPTES | 1.752844 | Control(minimal) | control 12 |
| ADGRL2 | RHSYccGGLPTES | 1.752844 | Control(minimal) | control 12 |
| ADGRL2 | RHSYccGGLPTES | 1.752844 | Control(minimal) | control 12 |
| ADGRL2 | RHSYccGGLPTES | 1.752844 | Control(minimal) | control 12 |
| ADGRL2 | RHSYccGGLPTES | 1.752844 | Control(minimal) | control 12 |
| ADGRL2 | RHSYccGGLPTES | 1.752844 | Control(minimal) | control 12 |
| ADGRL2 | RHSYccGGLPTES | 1.752844 | Control(minimal) | control 12 |
| ADGRL2 | RHSYccGGLPTES | 1.752844 | Control(minimal) | control 12 |
| ADGRL2 | RHSYccGGLPTES | 1.752844 | Control(minimal) | control 12 |
| ADGRL2 | RHSYccGGLPTES | 1.752844 | Control(minimal) | control 12 |
| ADGRL2 | RHSYccGGLPTES | 1.752844 | Control(minimal) | control 12 |
| ADGRL2 | RHSYccGGLPTES | 1.752844 | Control(minimal) | control 12 |
| ADGRL2 | RHSYccGGLPTES | 1.752844 | Control(minimal) | control 12 |
| ADGRL2 | RHSYccGGLPTES | 1.752844 | Control(minimal) | control 12 |
| ADGRL2 | RHSYccGGLPTES | 1.752844 | Control(minimal) | control 12 |
| ADGRL2 | RHSYccGGLPTES | 1.752844 | Control(minimal) | control 12 |
| ADGRL2 | RHSYccGGLPTES | 1.752844 | Control(minimal) | control 12 |
| ADGRL2 | RHSYccGGLPTES | 1.752844 | Control(minimal) | control 12 |
| ADGRL2 | RHSYccGGLPTES | 1.752844 | Control(minimal) | control 12 |
| ADGRL2 | RHSYccGGLPTES | 1.752844 | Control(minimal) | control 12 |
| ADGRL2 | RHSYccGGLPTES | 1.752844 | Control(minimal) | control 12 |
| ADGRL2 | RHSYccGGLPTES | 1.752844 | Control(minimal) | control 12 |
| ADGRL2 | RHSYccGGLPTES | 1.752844 | Control(minimal) | control 12 |
| ADGRL2 | RHSYccGGLPTES | 1.752844 | Control(minimal) | control 12 |
| ADGRL2 | RHSYccGGLPTES | 1.752844 | Control(minimal) | control 12 |
| ADGRL2 | RHSYccGGLPTES | 1.752844 | Control(minimal) | control 12 |
| ADGRL2 | RHSYccGGLPTES | 1.752844 | Control(minimal) | control 12 |
| ADGRL2 | RHSYccGGLPTES | 1.752844 | Control(minimal) | control 12 |
| ADGRL2 | RHSYccGGLPTES | 1.752844 | Control(minimal) | control 12 |
| ADGRL2 | RHSYccGGLPTES | 1.752844 | Control(minimal) | control 12 |
| ADGRL2 | RHSYccGGLPTES | 1.752844 | Control(minimal) | control 12 |
| ADGRL2 | RHSYccGGLPTES | 1.752844 | Control(minimal) | control 12 |
| ADGRL2 | RHSYccGGLPTES | 1.752844 | Control(minimal) | control 12 |
| ADGRL2 | RHSYccGGLPTES | 1.752844 | Control(minimal) | control 12 |
| ADGRL2 | RHSYccGGLPTES | 1.752844 | Control(minimal) | control 12 |
| ADGRL2 | RHSYccGGLPTES | 1.752844 | Control(minimal) | control 12 |
| ADGRL2 | RHSYccGGLPTES | 1.752844 | Control(minimal) | control 12 |
| ADGRL2 | sAAIDYKSYGTEKAc | 1.917539 | Control(minimal) | control 13 |
| ADGRL2 | sAAIDYKSYGTEKAc | 1.917539 | Control(minimal) | control 13 |
| ADGRL2 | sAAIDYKSYGTEKAc | 1.917539 | Control(minimal) | control 13 |
| ADGRL2 | sAAIDYKSYGTEKAc | 1.917539 | Control(minimal) | control 13 |
| ADGRL2 | sAAIDYKSYGTEKAc | 1.917539 | Control(minimal) | control 13 |
| ADGRL2 | sAAIDYKSYGTEKAc | 1.917539 | Control(minimal) | control 13 |
| ADGRL2 | sAAIDYKSYGTEKAc | 1.917539 | Control(minimal) | control 13 |
| ADGRL2 | sAAIDYKSYGTEKAc | 1.917539 | Control(minimal) | control 13 |
| ADGRL2 | sAAIDYKSYGTEKAc | 1.917539 | Control(minimal) | control 13 |
| ADGRL2 | sAAIDYKSYGTEKAc | 1.917539 | Control(minimal) | control 13 |
| ADGRL2 | sAAIDYKSYGTEKAc | 1.917539 | Control(minimal) | control 13 |
| ADGRL2 | sAAIDYKSYGTEKAc | 1.917539 | Control(minimal) | control 13 |
| ADGRL2 | sAAIDYKSYGTEKAc | 1.917539 | Control(minimal) | control 13 |
| ADGRL2 | sAAIDYKSYGTEKAc | 1.917539 | Control(minimal) | control 13 |
| ADGRL2 | sAAIDYKSYGTEKAc | 1.917539 | Control(minimal) | control 13 |
| ADGRL2 | sAAIDYKSYGTEKAc | 1.917539 | Control(minimal) | control 13 |
| ADGRL2 | sAAIDYKSYGTEKAc | 1.917539 | Control(minimal) | control 13 |
| ADGRL2 | sAAIDYKSYGTEKAc | 1.917539 | Control(minimal) | control 13 |
| ADGRL2 | sAAIDYKSYGTEKAc | 1.917539 | Control(minimal) | control 13 |
| ADGRL2 | sAAIDYKSYGTEKAc | 1.917539 | Control(minimal) | control 13 |
| ADGRL2 | sAAIDYKSYGTEKAc | 1.917539 | Control(minimal) | control 13 |
| ADGRL2 | sAAIDYKSYGTEKAc | 1.917539 | Control(minimal) | control 13 |
| ADGRL2 | sAAIDYKSYGTEKAc | 1.917539 | Control(minimal) | control 13 |
| ADGRL2 | sAAIDYKSYGTEKAc | 1.917539 | Control(minimal) | control 13 |
| ADGRL2 | sAAIDYKSYGTEKAc | 1.917539 | Control(minimal) | control 13 |
| ADGRL2 | sAAIDYKSYGTEKAc | 1.917539 | Control(minimal) | control 13 |
| ADGRL2 | sAAIDYKSYGTEKAc | 1.917539 | Control(minimal) | control 13 |
| ADGRL2 | sAAIDYKSYGTEKAc | 1.917539 | Control(minimal) | control 13 |
| ADGRL2 | sAAIDYKSYGTEKAc | 1.917539 | Control(minimal) | control 13 |
| ADGRL2 | sAAIDYKSYGTEKAc | 1.917539 | Control(minimal) | control 13 |
| ADGRL2 | sAAIDYKSYGTEKAc | 1.917539 | Control(minimal) | control 13 |
| ADGRL2 | sAAIDYKSYGTEKAc | 1.917539 | Control(minimal) | control 13 |
| ADGRL2 | sAAIDYKSYGTEKAc | 1.917539 | Control(minimal) | control 13 |
| ADGRL2 | sAAIDYKSYGTEKAc | 1.917539 | Control(minimal) | control 13 |
| ADGRL2 | sAAIDYKSYGTEKAc | 1.917539 | Control(minimal) | control 13 |
| ADGRL2 | sAAIDYKSYGTEKAc | 1.917539 | Control(minimal) | control 13 |
| ADGRL2 | sAAIDYKSYGTEKAc | 1.917539 | Control(minimal) | control 13 |
| ADGRL2 | sAAIDYKSYGTEKAc | 1.917539 | Control(minimal) | control 13 |
| ADGRL2 | sAAIDYKSYGTEKAc | 1.917539 | Control(minimal) | control 13 |
| ADGRL2 | sAAIDYKSYGTEKAc | 1.917539 | Control(minimal) | control 13 |
| ADGRL2 | sAAIDYKSYGTEKAc | 1.917539 | Control(minimal) | control 13 |
| ADGRL2 | sAAIDYKSYGTEKAc | 1.917539 | Control(minimal) | control 13 |
| ADGRL2 | sAAIDYKSYGTEKAc | 1.917539 | Control(minimal) | control 13 |
| AKAP2 | LSVRsQDTTVLETLS | 2.029934 | TBI(extreme) | TBI 1 |
| AKAP2 | LSVRsQDTTVLETLS | 2.029934 | TBI(extreme) | TBI 1 |
| AKAP2 | LSVRsQDTTVLETLS | 2.029934 | TBI(extreme) | TBI 1 |
| AKAP2 | LSVRsQDTTVLETLS | 2.029934 | TBI(extreme) | TBI 1 |
| AKAP2 | LSVRsQDTTVLETLS | 2.029934 | TBI(extreme) | TBI 1 |
| AKAP2 | LSVRsQDTTVLETLS | 2.029934 | TBI(extreme) | TBI 1 |
| AKAP2 | LSVRsQDTTVLETLS | 2.029934 | TBI(extreme) | TBI 1 |
| AKAP2 | LEANCCDSAVDGTyNGT | 1.705357 | TBI(extreme) | TBI 5 |
| AKAP2 | LEANCCDSAVDGTyNGT | 1.705357 | TBI(extreme) | TBI 5 |
| AKAP2 | LEANCCDSAVDGTyNGT | 1.705357 | TBI(extreme) | TBI 5 |
| AKAP2 | LEANCCDSAVDGTyNGT | 1.705357 | TBI(extreme) | TBI 5 |
| AKAP2 | LEANCCDSAVDGTyNGT | 1.705357 | TBI(extreme) | TBI 5 |
| AKAP2 | LEANCCDSAVDGTyNGT | 1.705357 | TBI(extreme) | TBI 5 |
| AKAP2 | LEANCCDSAVDGTyNGT | 1.705357 | TBI(extreme) | TBI 5 |
| AKAP2 | LEANCCDSAVDGTyNGT | 1.705357 | TBI(extreme) | TBI 5 |
| AKAP2 | LEANCCDSAVDGTyNGT | 1.705357 | TBI(extreme) | TBI 5 |
| AKAP2 | LEANCCDSAVDGTyNGT | 1.705357 | TBI(extreme) | TBI 5 |
| AKAP2 | LEANCCDSAVDGTyNGT | 1.705357 | TBI(extreme) | TBI 5 |
| AKAP2 | LEANCCDSAVDGTyNGT | 1.705357 | TBI(extreme) | TBI 5 |
| AKAP2 | LEANCCDSAVDGTyNGT | 1.705357 | TBI(extreme) | TBI 5 |
| AKAP2 | LEANCCDSAVDGTyNGT | 1.705357 | TBI(extreme) | TBI 5 |
| AKAP2 | LEANCCDSAVDGTyNGT | 1.705357 | TBI(extreme) | TBI 5 |
| ANO2 | PGGcLmELcIQLSIImLG | 1.809303 | TBI(extreme) | TBI 1 |
| ANO2 | PGGcLmELcIQLSIImLG | 1.809303 | TBI(extreme) | TBI 1 |
| ANO2 | PGGcLmELcIQLSIImLG | 1.809303 | TBI(extreme) | TBI 1 |
| ANO2 | PGGcLmELcIQLSIImLG | 1.809303 | TBI(extreme) | TBI 1 |
| ANO2 | PGGcLmELcIQLSIImLG | 1.809303 | TBI(extreme) | TBI 1 |
| ANO2 | PGGcLmELcIQLSIImLG | 1.809303 | TBI(extreme) | TBI 1 |
| ANO2 | PGGcLmELcIQLSIImLG | 1.809303 | TBI(extreme) | TBI 1 |
| ANO2 | PGGcLmELcIQLSIImLG | 1.809303 | TBI(extreme) | TBI 1 |
| ANO2 | PGGcLmELcIQLSIImLG | 1.809303 | TBI(extreme) | TBI 1 |
| ANO2 | PGGcLmELcIQLSIImLG | 1.809303 | TBI(extreme) | TBI 1 |
| ANO2 | PGGcLmELcIQLSIImLG | 1.809303 | TBI(extreme) | TBI 1 |
| ANO2 | PGGcLmELcIQLSIImLG | 1.809303 | TBI(extreme) | TBI 1 |
| ANO2 | PGGcLmELcIQLSIImLG | 1.809303 | TBI(extreme) | TBI 1 |
| ANO2 | PGGcLmELcIQLSIImLG | 1.809303 | TBI(extreme) | TBI 1 |
| ANO2 | PGGcLmELcIQLSIImLG | 1.809303 | TBI(extreme) | TBI 1 |
| ANO2 | PGGcLmELcIQLSIImLG | 1.809303 | TBI(extreme) | TBI 1 |
| ANO2 | PGGcLmELcIQLSIImLG | 1.809303 | TBI(extreme) | TBI 1 |
| ANO2 | PGGcLmELcIQLSIImLG | 1.809303 | TBI(extreme) | TBI 8 |
| ANO2 | PGGcLmELcIQLSIImLG | 1.809303 | TBI(extreme) | TBI 8 |
| ANO2 | PGGcLmELcIQLSIImLG | 1.809303 | TBI(extreme) | TBI 8 |
| ANO2 | PGGcLmELcIQLSIImLG | 1.809303 | TBI(extreme) | TBI 8 |
| ANO2 | PGGcLmELcIQLSIImLG | 1.809303 | TBI(extreme) | TBI 8 |
| ANO2 | PGGcLmELcIQLSIImLG | 1.809303 | TBI(extreme) | TBI 8 |
| ANO2 | PGGcLmELcIQLSIImLG | 1.809303 | TBI(extreme) | TBI 8 |
| ANO2 | PGGcLmELcIQLSIImLG | 1.809303 | TBI(extreme) | TBI 8 |
| ANO2 | PGGcLmELcIQLSIImLG | 1.809303 | TBI(extreme) | TBI 8 |
| ANO2 | PGGcLmELcIQLSIImLG | 1.809303 | TBI(extreme) | TBI 8 |
| ANO2 | PGGcLmELcIQLSIImLG | 1.809303 | TBI(extreme) | TBI 8 |
| ANO2 | PGGcLmELcIQLSIImLG | 1.809303 | TBI(extreme) | TBI 8 |
| ANO2 | PGGcLmELcIQLSIImLG | 1.809303 | TBI(extreme) | TBI 8 |
| ANO2 | PGGcLmELcIQLSIImLG | 1.809303 | TBI(extreme) | TBI 8 |
| ANO2 | PGGcLmELcIQLSIImLG | 1.809303 | TBI(extreme) | TBI 8 |
| ANO2 | PGGcLmELcIQLSIImLG | 1.809303 | TBI(extreme) | TBI 8 |
| ANO2 | PGGcLmELcIQLSIImLG | 1.809303 | TBI(extreme) | TBI 8 |
| ANO2 | MDFAAVAFTVALTAsEAL | 1.76963 | Control(minimal) | control 4 |
| ANO2 | MDFAAVAFTVALTAsEAL | 1.76963 | Control(minimal) | control 4 |
| ANO2 | MDFAAVAFTVALTAsEAL | 1.76963 | Control(minimal) | control 4 |
| ANO2 | MDFAAVAFTVALtASEAL | 1.76963 | Control(minimal) | control 4 |
| ANO2 | MDFAAVAFTVALTAsEAL | 1.76963 | Control(minimal) | control 4 |
| ANO2 | MDFAAVAFTVALtASEAL | 1.733693 | Control(minimal) | control 4 |
| ANO2 | MDFAAVAFTVALtASEAL | 1.733693 | Control(minimal) | control 4 |
| ANO2 | MDFAAVAFTVALtASEAL | 1.733693 | Control(minimal) | control 4 |
| ASXL1 | DFETPGSILVNT | 2.350786 | Control(minimal) | control 8 |
| ASXL1 | DFETPGSILVNT | 2.350786 | Control(minimal) | control 8 |
| ASXL1 | DFETPGSILVNT | 2.350786 | Control(minimal) | control 8 |
| ASXL1 | DFETPGSILVNT | 2.350786 | Control(minimal) | control 8 |
| ASXL1 | DFETPGSILVNT | 2.350786 | Control(minimal) | control 8 |
| ASXL1 | DFETPGSILVNT | 2.350786 | Control(minimal) | control 8 |
| ASXL1 | DFETPGSILVNT | 2.350786 | Control(minimal) | control 8 |
| ASXL1 | DFETPGSILVNT | 2.350786 | Control(minimal) | control 8 |
| ASXL1 | DFETPGSILVNT | 2.350786 | Control(minimal) | control 8 |
| ASXL1 | DFETPGSILVNT | 2.350786 | Control(minimal) | control 8 |
| ASXL1 | DFETPGSILVNT | 2.350786 | Control(minimal) | control 8 |
| ASXL1 | DFETPGSILVNT | 2.350786 | Control(minimal) | control 8 |
| ASXL1 | DFETPGSILVNT | 2.350786 | Control(minimal) | control 8 |
| ASXL1 | IMCQGcGAFCHD | 2.350786 | Control(minimal) | control 8 |
| ASXL1 | QICPRIIPTtESSCRG | 1.817055 | Control(minimal) | control 9 |
| ASXL1 | QICPRIIPTtESSCRG | 1.817055 | Control(minimal) | control 9 |
| ASXL1 | QICPRIIPTtESSCRG | 1.817055 | Control(minimal) | control 9 |
| ASXL1 | QICPRIIPTtESSCRG | 1.817055 | Control(minimal) | control 9 |
| ASXL1 | QICPRIIPTtESSCRG | 1.817055 | Control(minimal) | control 9 |
| ASXL1 | QICPRIIPTtESSCRG | 1.817055 | Control(minimal) | control 9 |
| ASXL1 | QICPRIIPTtESSCRG | 1.817055 | Control(minimal) | control 9 |
| ASXL1 | QICPRIIPTtESSCRG | 1.817055 | Control(minimal) | control 9 |
| ASXL1 | QICPRIIPTtESSCRG | 1.817055 | Control(minimal) | control 9 |
| ASXL1 | QICPRIIPTtESSCRG | 1.817055 | Control(minimal) | control 9 |
| ASXL1 | QICPRIIPTtESSCRG | 1.817055 | Control(minimal) | control 9 |
| ASXL1 | QICPRIIPTtESSCRG | 1.817055 | Control(minimal) | control 9 |
| ASXL1 | QICPRIIPTtESSCRG | 1.817055 | Control(minimal) | control 9 |
| ASXL1 | QICPRIIPTtESSCRG | 1.817055 | Control(minimal) | control 9 |
| ASXL1 | QICPRIIPTtESSCRG | 1.817055 | Control(minimal) | control 9 |
| ATRN | THNDTSMSHGAKCFs | 1.717648 | Control(minimal) | control 9 |
| ATRN | THNDTSMSHGAKCFs | 1.717648 | Control(minimal) | control 9 |
| ATRN | THNDTSMSHGAKCFs | 1.717648 | Control(minimal) | control 9 |
| ATRN | THNDTSMSHGAKCFs | 1.717648 | Control(minimal) | control 9 |
| ATRN | THNDTSMSHGAKCFs | 1.717648 | Control(minimal) | control 9 |
| ATRN | THNDTSMSHGAKCFs | 1.717648 | Control(minimal) | control 9 |
| ATRN | THNDTSMSHGAKCFs | 1.717648 | Control(minimal) | control 9 |
| ATRN | THNDTSMSHGAKCFs | 1.717648 | Control(minimal) | control 9 |
| ATRN | THNDTSMSHGAKCFs | 1.717648 | Control(minimal) | control 9 |
| ATRN | THNDTSMSHGAKCFs | 1.717648 | Control(minimal) | control 9 |
| ATRN | THNDTSMSHGAKCFs | 1.717648 | Control(minimal) | control 9 |
| ATRN | RNRKQQPPAQPGTCI | 1.740042 | Control(minimal) | control 10 |
| ATRN | RNRKQQPPAQPGTCI | 1.740042 | Control(minimal) | control 10 |
| ATRN | RNRKQQPPAQPGTCI | 1.740042 | Control(minimal) | control 10 |
| ATRN | RNRKQQPPAQPGTCI | 1.740042 | Control(minimal) | control 10 |
| ATRN | RNRKQQPPAQPGTCI | 1.740042 | Control(minimal) | control 10 |
| ATRN | THNDTSMSHGAKCFs | 1.717648 | Control(minimal) | control 11 |
| ATRN | THNDTSMSHGAKCFs | 1.717648 | Control(minimal) | control 11 |
| ATRN | THNDTSMSHGAKCFs | 1.717648 | Control(minimal) | control 11 |
| ATRN | THNDTSMSHGAKCFs | 1.717648 | Control(minimal) | control 11 |
| ATRN | THNDTSMSHGAKCFs | 1.717648 | Control(minimal) | control 11 |
| ATRN | THNDTSMSHGAKCFs | 1.717648 | Control(minimal) | control 11 |
| ATRN | THNDTSMSHGAKCFs | 1.717648 | Control(minimal) | control 11 |
| ATRN | THNDTSMSHGAKCFs | 1.717648 | Control(minimal) | control 11 |
| ATRN | THNDTSMSHGAKCFs | 1.717648 | Control(minimal) | control 11 |
| ATRN | THNDTSMSHGAKCFs | 1.717648 | Control(minimal) | control 11 |
| ATRN | THNDTSMSHGAKCFs | 1.717648 | Control(minimal) | control 11 |
| BAZ2B | YRRKLFDAsHSLR | 1.856307 | TBI(extreme) | TBI 10 |
| BAZ2B | YRRKLFDAsHSLR | 1.856307 | TBI(extreme) | TBI 10 |
| BAZ2B | YRRKLFDAsHSLR | 1.856307 | TBI(extreme) | TBI 10 |
| BAZ2B | YRRKLFDAsHSLR | 1.856307 | TBI(extreme) | TBI 10 |
| BAZ2B | YRRKLFDAsHSLR | 1.856307 | TBI(extreme) | TBI 10 |
| BAZ2B | YRRKLFDAsHSLR | 1.856307 | TBI(extreme) | TBI 10 |
| BAZ2B | YRRKLFDAsHSLR | 1.856307 | TBI(extreme) | TBI 10 |
| BAZ2B | YRRKLFDAsHSLR | 1.856307 | TBI(extreme) | TBI 10 |
| BAZ2B | YRRKLFDAsHSLR | 1.856307 | TBI(extreme) | TBI 10 |
| BAZ2B | YRRKLFDAsHSLR | 1.856307 | TBI(extreme) | TBI 10 |
| BAZ2B | YRRKLFDAsHSLR | 1.856307 | TBI(extreme) | TBI 10 |
| BAZ2B | YRRKLFDAsHSLR | 1.856307 | TBI(extreme) | TBI 10 |
| BAZ2B | YRRKLFDAsHSLR | 1.856307 | TBI(extreme) | TBI 10 |
| BAZ2B | MLFTSSVPsCtLsN | 1.756865 | TBI(extreme) | TBI 46 |
| BAZ2B | MLFTSSVPsCtLsN | 1.756865 | TBI(extreme) | TBI 46 |
| BAZ2B | MLFTSSVPsCtLsN | 1.756865 | TBI(extreme) | TBI 46 |
| BIRC6 | LcNSSAMAMIIGASG | 1.96741 | TBI(extreme) | TBI 9 |
| BIRC6 | LcNSSAMAMIIGASG | 1.96741 | TBI(extreme) | TBI 9 |
| BIRC6 | LcNSSAMAMIIGASG | 1.96741 | TBI(extreme) | TBI 9 |
| BIRC6 | LcNSSAMAMIIGASG | 1.96741 | TBI(extreme) | TBI 9 |
| BIRC6 | LcNSSAMAMIIGASG | 1.96741 | TBI(extreme) | TBI 9 |
| BIRC6 | LcNSSAMAMIIGASG | 1.96741 | TBI(extreme) | TBI 9 |
| BIRC6 | LcNSSAMAMIIGASG | 1.96741 | TBI(extreme) | TBI 9 |
| BIRC6 | LcNSSAMAMIIGASG | 1.96741 | TBI(extreme) | TBI 9 |
| BIRC6 | LcNSSAMAMIIGASG | 1.96741 | TBI(extreme) | TBI 9 |
| BIRC6 | DGTSGATLQASALsAKPGG | 2.003645 | TBI(extreme) | TBI 30 |
| BIRC6 | DGTSGATLQASALsAKPGG | 2.003645 | TBI(extreme) | TBI 30 |
| BIRC6 | DGTSGATLQASALsAKPGG | 2.003645 | TBI(extreme) | TBI 30 |
| BIRC6 | DGTSGATLQASALsAKPGG | 2.003645 | TBI(extreme) | TBI 30 |
| BIRC6 | DGTSGATLQASALsAKPGG | 2.003645 | TBI(extreme) | TBI 30 |
| BIRC6 | DGTSGATLQASALsAKPGG | 2.003645 | TBI(extreme) | TBI 30 |
| BIRC6 | DGTSGATLQASALsAKPGG | 2.003645 | TBI(extreme) | TBI 30 |
| BIRC6 | DGTSGATLQASALsAKPGG | 2.003645 | TBI(extreme) | TBI 30 |
| C18orf15 | GPCcPVHPQsLTVVCMC | 1.758958 | Control(minimal) | control 4 |
| C18orf15 | GPCcPVHPQsLTVVCMC | 1.758958 | Control(minimal) | control 4 |
| C18orf15 | GPCcPVHPQsLTVVCMC | 1.758958 | Control(minimal) | control 4 |
| C18orf15 | GPCcPVHPQSLtVVCMC | 1.758958 | Control(minimal) | control 4 |
| C18orf15 | GPCcPVHPQsLTVVCMC | 1.758958 | Control(minimal) | control 4 |
| C18orf15 | VCMcACAcMRAHRy | 1.873156 | Control(minimal) | control 8 |
| C18orf15 | VCMcACAcMRAHRy | 1.873156 | Control(minimal) | control 8 |
| C18orf15 | VCMcACAcMRAHRy | 1.873156 | Control(minimal) | control 8 |
| C18orf15 | cMCVLVcMCACAcmR | 1.780471 | Control(minimal) | control 10 |
| C18orf15 | cMCVLVcMCACAcmR | 1.780471 | Control(minimal) | control 10 |
| C18orf15 | cMCVLVcMCACAcmR | 1.780471 | Control(minimal) | control 10 |
| C18orf15 | CmCVLVcMCACAcmR | 1.780471 | Control(minimal) | control 10 |
| C18orf15 | cMCVLVcMCACAcmR | 1.780471 | Control(minimal) | control 10 |
| C18orf15 | cMCVLVcMCAcACmR | 1.780471 | Control(minimal) | control 10 |
| C18orf15 | cMCVLVcMCACAcmR | 1.780471 | Control(minimal) | control 10 |
| CNNM1 | RINRNNNGcLPCsRCS | 1.739692 | TBI(extreme) | TBI 1 |
| CNNM1 | RINRNNNGcLPCsRCS | 1.739692 | TBI(extreme) | TBI 1 |
| CNNM1 | RINRNNNGcLPCsRCS | 1.739692 | TBI(extreme) | TBI 1 |
| CNNM1 | RINRNNNGcLPCsRCS | 1.739692 | TBI(extreme) | TBI 1 |
| CNNM1 | RINRNNNGcLPCsRCS | 1.739692 | TBI(extreme) | TBI 1 |
| CNNM1 | LAGSSVFLPVSVsRTF | 1.794883 | TBI(extreme) | TBI 6 |
| CNNM1 | LAGSSVFLPVSVsRTF | 1.794883 | TBI(extreme) | TBI 6 |
| CNNM1 | LAGSSVFLPVSVsRTF | 1.794883 | TBI(extreme) | TBI 6 |
| CNNM1 | LAGSSVFLPVSVSRtF | 1.794883 | TBI(extreme) | TBI 6 |
| CNNM1 | LAGSSVFLPVSVsRTF | 1.794883 | TBI(extreme) | TBI 6 |
| CNNM1 | RINRNNNGcLPCsRCS | 1.739692 | TBI(extreme) | TBI 8 |
| CNNM1 | RINRNNNGcLPCsRCS | 1.739692 | TBI(extreme) | TBI 8 |
| CNNM1 | RINRNNNGcLPCsRCS | 1.739692 | TBI(extreme) | TBI 8 |
| CNNM1 | RINRNNNGcLPCsRCS | 1.739692 | TBI(extreme) | TBI 8 |
| CNNM1 | RINRNNNGcLPCsRCS | 1.739692 | TBI(extreme) | TBI 8 |
| CPAMD8 | IARcGcDHDcGA | 1.882194 | TBI(extreme) | TBI 6 |
| CPAMD8 | VSAKGDGcCLm | 1.882194 | TBI(extreme) | TBI 6 |
| CPAMD8 | VSAKGDGcCLm | 1.882194 | TBI(extreme) | TBI 6 |
| CPAMD8 | VSAKGDGcCLm | 1.882194 | TBI(extreme) | TBI 6 |
| CPAMD8 | VSAKGDGccLM | 1.882194 | TBI(extreme) | TBI 6 |
| CPAMD8 | VSAKGDGcCLm | 1.882194 | TBI(extreme) | TBI 6 |
| CPAMD8 | VSAKGDGcCLm | 1.882194 | TBI(extreme) | TBI 6 |
| CPAMD8 | VSAKGDGcCLm | 1.882194 | TBI(extreme) | TBI 6 |
| CPAMD8 | VSAKGDGcCLm | 1.882194 | TBI(extreme) | TBI 6 |
| CPAMD8 | VSAKGDGcCLm | 1.882194 | TBI(extreme) | TBI 6 |
| CPAMD8 | VSAKGDGcCLm | 1.882194 | TBI(extreme) | TBI 6 |
| CPAMD8 | VSAKGDGcCLm | 1.882194 | TBI(extreme) | TBI 6 |
| CPAMD8 | IARcGcDHDcGA | 2.209423 | Control(minimal) | control 3 |
| CPAMD8 | IARcGcDHDcGA | 2.209423 | Control(minimal) | control 3 |
| CPAMD8 | IARcGcDHDcGA | 2.209423 | Control(minimal) | control 3 |
| CPAMD8 | IARcGcDHDcGA | 2.209423 | Control(minimal) | control 3 |
| CPAMD8 | IARcGcDHDcGA | 2.209423 | Control(minimal) | control 3 |
| CPAMD8 | IARcGcDHDcGA | 2.209423 | Control(minimal) | control 3 |
| CPAMD8 | IARcGcDHDcGA | 2.209423 | Control(minimal) | control 3 |
| CPAMD8 | IARcGcDHDcGA | 2.209423 | Control(minimal) | control 3 |
| CPAMD8 | IARcGcDHDcGA | 2.209423 | Control(minimal) | control 3 |
| CPAMD8 | IARcGcDHDcGA | 2.209423 | Control(minimal) | control 3 |
| CPAMD8 | IARcGcDHDcGA | 2.209423 | Control(minimal) | control 3 |
| CPAMD8 | VSAKGDGccLM | 2.209423 | Control(minimal) | control 3 |
| CPAMD8 | IARcGcDHDcGA | 2.362285 | Control(minimal) | control 12 |
| CPAMD8 | IARCGcDHDCGA | 2.362285 | Control(minimal) | control 12 |
| CPAMD8 | IARCGcDHDCGA | 2.362285 | Control(minimal) | control 12 |
| CPAMD8 | IARCGcDHDCGA | 2.362285 | Control(minimal) | control 12 |
| CPAMD8 | IARCGcDHDCGA | 2.362285 | Control(minimal) | control 12 |
| CPAMD8 | IARcGcDHDcGA | 2.362285 | Control(minimal) | control 12 |
| CPAMD8 | IARCGcDHDCGA | 2.362285 | Control(minimal) | control 12 |
| CPAMD8 | IARCGcDHDCGA | 2.362285 | Control(minimal) | control 12 |
| CPAMD8 | IARCGcDHDCGA | 2.362285 | Control(minimal) | control 12 |
| CPAMD8 | IARCGcDHDCGA | 2.362285 | Control(minimal) | control 12 |
| CPAMD8 | IARCGcDHDCGA | 2.362285 | Control(minimal) | control 12 |
| CPAMD8 | IARCGcDHDCGA | 2.362285 | Control(minimal) | control 12 |
| CPAMD8 | IARCGcDHDCGA | 2.362285 | Control(minimal) | control 12 |
| CPAMD8 | IARCGcDHDCGA | 2.362285 | Control(minimal) | control 12 |
| CPAMD8 | IARCGcDHDCGA | 2.362285 | Control(minimal) | control 12 |
| CRACR2B | CCccccWARPPRRG | 1.830102 | TBI(extreme) | TBI 5 |
| CRACR2B | CCccccWARPPRRG | 1.830102 | TBI(extreme) | TBI 5 |
| CRACR2B | CCccccWARPPRRG | 1.830102 | TBI(extreme) | TBI 5 |
| CRACR2B | CCccccWARPPRRG | 1.830102 | TBI(extreme) | TBI 5 |
| CRACR2B | CCccccWARPPRRG | 1.830102 | TBI(extreme) | TBI 5 |
| CRACR2B | CCccccWARPPRRG | 1.830102 | TBI(extreme) | TBI 5 |
| CRACR2B | cCCcccWARPPRRG | 1.830102 | TBI(extreme) | TBI 5 |
| CRACR2B | CCccccWARPPRRG | 1.830102 | TBI(extreme) | TBI 5 |
| CRACR2B | cCCcccWARPPRRG | 1.830102 | TBI(extreme) | TBI 5 |
| CRACR2B | CCccccWARPPRRG | 1.830102 | TBI(extreme) | TBI 5 |
| CRACR2B | CcCcccWARPPRRG | 1.830102 | TBI(extreme) | TBI 5 |
| CRACR2B | CCccccWARPPRRG | 1.830102 | TBI(extreme) | TBI 5 |
| CRACR2B | cCCcccWARPPRRG | 1.830102 | TBI(extreme) | TBI 5 |
| CRACR2B | CCccccWARPPRRG | 1.830102 | TBI(extreme) | TBI 5 |
| CRACR2B | cCCcccWARPPRRG | 1.830102 | TBI(extreme) | TBI 5 |
| CRACR2B | CCccccWARPPRRG | 1.830102 | TBI(extreme) | TBI 5 |
| CRACR2B | cCCcccWARPPRRG | 1.830102 | TBI(extreme) | TBI 5 |
| CRACR2B | CCccccWARPPRRG | 1.830102 | TBI(extreme) | TBI 5 |
| CRACR2B | cCCcccWARPPRRG | 1.830102 | TBI(extreme) | TBI 5 |
| CRACR2B | CCccccWARPPRRG | 1.830102 | TBI(extreme) | TBI 5 |
| CRACR2B | cCCcccWARPPRRG | 1.830102 | TBI(extreme) | TBI 5 |
| CRACR2B | CCccccWARPPRRG | 1.830102 | TBI(extreme) | TBI 5 |
| CRACR2B | TcCCcCcWARPPRRGSG | 1.714516 | TBI(extreme) | TBI 47 |
| CRACR2B | TcCCcCcWARPPRRGSG | 1.714516 | TBI(extreme) | TBI 47 |
| CRACR2B | TcCCcCcWARPPRRGSG | 1.714516 | TBI(extreme) | TBI 47 |
| CRACR2B | TcCCcCcWARPPRRGSG | 1.714516 | TBI(extreme) | TBI 47 |
| CRACR2B | TcCCcCcWARPPRRGSG | 1.714516 | TBI(extreme) | TBI 47 |
| CRACR2B | TcCCcCcWARPPRRGSG | 1.714516 | TBI(extreme) | TBI 47 |
| CRACR2B | TcCCcCcWARPPRRGSG | 1.714516 | TBI(extreme) | TBI 47 |
| CSMD1 | PSsIVSTGSILTLWF | 1.959275 | TBI(extreme) | TBI 1 |
| CSMD1 | PSsIVSTGSILTLWF | 1.959275 | TBI(extreme) | TBI 1 |
| CSMD1 | PSsIVSTGSILTLWF | 1.959275 | TBI(extreme) | TBI 1 |
| CSMD1 | PSsIVSTGSILTLWF | 1.959275 | TBI(extreme) | TBI 1 |
| CSMD1 | PSsIVSTGSILTLWF | 1.959275 | TBI(extreme) | TBI 1 |
| CSMD1 | PSsIVSTGSILTLWF | 1.959275 | TBI(extreme) | TBI 8 |
| CSMD1 | PSsIVSTGSILTLWF | 1.959275 | TBI(extreme) | TBI 8 |
| CSMD1 | PSsIVSTGSILTLWF | 1.959275 | TBI(extreme) | TBI 8 |
| CSMD1 | PSsIVSTGSILTLWF | 1.959275 | TBI(extreme) | TBI 8 |
| CSMD1 | PSsIVSTGSILTLWF | 1.959275 | TBI(extreme) | TBI 8 |
| CSMD1 | ERTcLLNGSWsGLQ | 1.789065 | TBI(extreme) | TBI 46 |
| CSMD1 | ERTcLLNGSWsGLQ | 1.789065 | TBI(extreme) | TBI 46 |
| CSMD1 | ERTcLLNGSWsGLQ | 1.789065 | TBI(extreme) | TBI 46 |
| CSMD1 | ERTcLLNGSWsGLQ | 1.789065 | TBI(extreme) | TBI 46 |
| CSMD1 | ERTcLLNGSWsGLQ | 1.789065 | TBI(extreme) | TBI 46 |
| CSMD1 | ERTcLLNGSWsGLQ | 1.789065 | TBI(extreme) | TBI 46 |
| CSMD1 | ERTcLLNGSWsGLQ | 1.789065 | TBI(extreme) | TBI 46 |
| CUBN | GcGGELSGATGSFsSPGFP | 1.846899 | Control(minimal) | control 11 |
| CUBN | GcGGELSGATGSFsSPGFP | 1.846899 | Control(minimal) | control 11 |
| CUBN | GcGGELSGATGSFsSPGFP | 1.846899 | Control(minimal) | control 11 |
| CUBN | GcGGELSGATGsFSSPGFP | 1.846899 | Control(minimal) | control 11 |
| CUBN | GcGGELSGATGSFsSPGFP | 1.846899 | Control(minimal) | control 11 |
| CUBN | GcGGELSGATGsFSSPGFP | 1.846899 | Control(minimal) | control 11 |
| CUBN | GcGGELSGATGSFsSPGFP | 1.846899 | Control(minimal) | control 11 |
| CUBN | GcGGELSGATGsFSSPGFP | 1.846899 | Control(minimal) | control 11 |
| CUBN | GcGGELSGATGSFsSPGFP | 1.846899 | Control(minimal) | control 11 |
| CUBN | GcGGELSGATGsFSSPGFP | 1.846899 | Control(minimal) | control 11 |
| CUBN | GcGGELSGATGSFsSPGFP | 1.846899 | Control(minimal) | control 11 |
| CUBN | GcGGELSGATGsFSSPGFP | 1.846899 | Control(minimal) | control 11 |
| CUBN | GcGGELSGATGSFsSPGFP | 1.846899 | Control(minimal) | control 11 |
| CUBN | GcGGELSGATGsFSSPGFP | 1.788015 | Control(minimal) | control 11 |
| CUBN | GcGGELSGATGsFSSPGFP | 1.788015 | Control(minimal) | control 11 |
| CUBN | GcGGELSGATGsFSSPGFP | 1.788015 | Control(minimal) | control 11 |
| CUBN | GcGGELSGATGsFSSPGFP | 1.788015 | Control(minimal) | control 11 |
| CUBN | GcGGELSGATGsFSSPGFP | 1.788015 | Control(minimal) | control 11 |
| CUBN | GcGGELSGATGsFSSPGFP | 1.788015 | Control(minimal) | control 11 |
| CUBN | GcGGELSGATGsFSSPGFP | 1.788015 | Control(minimal) | control 11 |
| CUBN | PDIyPPNVEcVWNI | 1.734801 | Control(minimal) | control 12 |
| CUBN | PDIyPPNVEcVWNI | 1.734801 | Control(minimal) | control 12 |
| CUBN | PDIyPPNVEcVWNI | 1.734801 | Control(minimal) | control 12 |
| CUBN | PDIyPPNVEcVWNI | 1.734801 | Control(minimal) | control 12 |
| CUBN | PDIyPPNVEcVWNI | 1.734801 | Control(minimal) | control 12 |
| CUBN | PDIyPPNVEcVWNI | 1.734801 | Control(minimal) | control 12 |
| CUBN | PDIyPPNVEcVWNI | 1.734801 | Control(minimal) | control 12 |
| DCAF1 | LPLFSscQIQQLmK | 1.783993 | TBI(extreme) | TBI 9 |
| DCAF1 | LPLFSscQIQQLmK | 1.783993 | TBI(extreme) | TBI 9 |
| DCAF1 | LPLFSscQIQQLmK | 1.783993 | TBI(extreme) | TBI 9 |
| DCAF1 | LPLFSscQIQQLmK | 1.783993 | TBI(extreme) | TBI 9 |
| DCAF1 | LPLFSscQIQQLmK | 1.783993 | TBI(extreme) | TBI 9 |
| DCAF1 | LPLFSscQIQQLmK | 1.783993 | TBI(extreme) | TBI 9 |
| DCAF1 | LPLFSscQIQQLmK | 1.783993 | TBI(extreme) | TBI 9 |
| DCAF1 | LPLFSscQIQQLmK | 1.783993 | TBI(extreme) | TBI 9 |
| DCAF1 | LPLFSscQIQQLmK | 1.783993 | TBI(extreme) | TBI 9 |
| DCAF1 | LPLFSscQIQQLmK | 1.783993 | TBI(extreme) | TBI 9 |
| DCAF1 | LPLFSscQIQQLmK | 1.783993 | TBI(extreme) | TBI 9 |
| DCAF1 | ILQVEQYLMAsDMAG | 2.415767 | Control(minimal) | control 8 |
| DCAF1 | ILQVEQYLMAsDMAG | 2.415767 | Control(minimal) | control 8 |
| DCAF1 | ILQVEQYLMAsDMAG | 2.415767 | Control(minimal) | control 8 |
| DCAF1 | HVsWAVcGPGAcCHSTm | 1.932994 | Control(minimal) | control 10 |
| DCAF1 | HVsWAVcGPGAcCHSTm | 1.932994 | Control(minimal) | control 10 |
| DCAF1 | HVsWAVcGPGAcCHSTm | 1.932994 | Control(minimal) | control 10 |
| DNAJC5 | CCccLcCCFNCCcGK | 1.716994 | TBI(extreme) | TBI 5 |
| DNAJC5 | CCccLcCCFNCCcGK | 1.716994 | TBI(extreme) | TBI 5 |
| DNAJC5 | CCccLcCCFNCCcGK | 1.716994 | TBI(extreme) | TBI 5 |
| DNAJC5 | CCccLcCCFNCCcGK | 1.716994 | TBI(extreme) | TBI 5 |
| DNAJC5 | CCccLcCCFNCCcGK | 1.716994 | TBI(extreme) | TBI 5 |
| DNAJC5 | CCccLcCCFNCCcGK | 1.716994 | TBI(extreme) | TBI 5 |
| DNAJC5 | CCccLcCCFNCCcGK | 1.716994 | TBI(extreme) | TBI 5 |
| DNAJC5 | CCccLcCCFNCCcGK | 1.716994 | TBI(extreme) | TBI 5 |
| DNAJC5 | CCccLcCCFNCCcGK | 1.716994 | TBI(extreme) | TBI 5 |
| DNAJC5 | CCCCLCCcFNcccGK | 1.861474 | TBI(extreme) | TBI 10 |
| DNAJC5 | CCCCLCCcFNcccGK | 1.861474 | TBI(extreme) | TBI 10 |
| DNAJC5 | CCCCLCCcFNcccGK | 1.861474 | TBI(extreme) | TBI 10 |
| DNAJC5 | CCCCLCCcFNcccGK | 1.861474 | TBI(extreme) | TBI 10 |
| DNAJC5 | CCCCLCCcFNcccGK | 1.861474 | TBI(extreme) | TBI 10 |
| DNAJC5 | CCCCLCCcFNcccGK | 1.861474 | TBI(extreme) | TBI 10 |
| DNAJC5 | CCCCLCCcFNcccGK | 1.861474 | TBI(extreme) | TBI 10 |
| DNAJC5 | KSKcACHQLTIQATACt | 1.861474 | TBI(extreme) | TBI 10 |
| DNAJC5 | CCCCLCCcFNcccGK | 1.859996 | TBI(extreme) | TBI 10 |
| DNAJC5 | TCCYCCccLCCcFNcC | 2.080314 | Control(minimal) | control 7 |
| DNAJC5 | TCCYCCccLCCcFNcC | 2.080314 | Control(minimal) | control 7 |
| DNAJC5 | TCCYCCccLCCcFNcC | 2.080314 | Control(minimal) | control 7 |
| DNAJC5 | TCCYCCccLCCcFNcC | 2.080314 | Control(minimal) | control 7 |
| DNAJC5 | TCCYCCccLCCcFNcC | 2.080314 | Control(minimal) | control 7 |
| DNAJC5 | TCCYCCccLCCcFNcC | 2.080314 | Control(minimal) | control 7 |
| DNAJC5 | TCCYCCccLCCcFNcC | 2.080314 | Control(minimal) | control 7 |
| DNAJC5 | TCCYCCccLCCcFNcC | 2.080314 | Control(minimal) | control 7 |
| DNAJC5 | TCCYCCccLCCcFNcC | 2.080314 | Control(minimal) | control 7 |
| DNAJC5 | TCCYcCCcLCCcFNcC | 2.080314 | Control(minimal) | control 7 |
| DNAJC5 | TCCYcCCcLCCcFNcC | 2.080314 | Control(minimal) | control 7 |
| DNAJC5 | TCCYcCCcLCCcFNcC | 2.080314 | Control(minimal) | control 7 |
| DNAJC5 | TCCYcCCcLCCcFNcC | 2.080314 | Control(minimal) | control 7 |
| DNAJC5 | TCCYCcCcLCCcFNcC | 2.080314 | Control(minimal) | control 7 |
| DNAJC5 | TCCYCcCcLCCcFNcC | 2.080314 | Control(minimal) | control 7 |
| DNAJC5 | TCCYCcCcLCCcFNcC | 2.080314 | Control(minimal) | control 7 |
| DNAJC5 | TCCYCcCcLCCcFNcC | 2.080314 | Control(minimal) | control 7 |
| DNAJC5 | TCCYCCccLCCcFNCc | 2.080314 | Control(minimal) | control 7 |
| DNAJC5 | TCCYCCccLCCcFNCc | 2.080314 | Control(minimal) | control 7 |
| DNAJC5 | TCCYCCccLCCcFNCc | 2.080314 | Control(minimal) | control 7 |
| DNAJC5 | TCCYCCccLCCcFNCc | 2.080314 | Control(minimal) | control 7 |
| DNAJC5 | TCcYCCCcLCCcFNcC | 2.080314 | Control(minimal) | control 7 |
| DNAJC5 | TCcYCCCcLCCcFNcC | 2.080314 | Control(minimal) | control 7 |
| DNAJC5 | TCcYCCCcLCCcFNcC | 2.080314 | Control(minimal) | control 7 |
| DNAJC5 | TCcYCCCcLCCcFNcC | 2.080314 | Control(minimal) | control 7 |
| DNAJC5 | TCCYCCccLCCcFNcC | 2.080314 | Control(minimal) | control 7 |
| DNAJC5 | TCCYCCccLCCcFNcC | 2.080314 | Control(minimal) | control 7 |
| DNAJC5 | TCCYCCccLCCcFNcC | 2.080314 | Control(minimal) | control 7 |
| DNAJC5 | TCCYCCccLCCcFNcC | 2.080314 | Control(minimal) | control 7 |
| DNAJC5 | TCCYccCCLCCcFNcC | 2.080314 | Control(minimal) | control 7 |
| DNAJC5 | TCCYCCccLCCcFNcC | 2.080314 | Control(minimal) | control 7 |
| DNAJC5 | TCCYccCCLCCcFNcC | 2.080314 | Control(minimal) | control 7 |
| DNAJC5 | TCCYCCccLCCcFNcC | 2.080314 | Control(minimal) | control 7 |
| DNAJC5 | TCCYccCCLCCcFNcC | 2.080314 | Control(minimal) | control 7 |
| DNAJC5 | TCCYCCccLCCcFNcC | 2.080314 | Control(minimal) | control 7 |
| DNAJC5 | TCCYccCCLCCcFNcC | 2.080314 | Control(minimal) | control 7 |
| DNAJC5 | TCCYCCccLCCcFNcC | 2.080314 | Control(minimal) | control 7 |
| DNAJC5 | TCCYccCCLCCcFNcC | 2.080314 | Control(minimal) | control 7 |
| DNAJC5 | TCCYCCccLCCcFNcC | 2.080314 | Control(minimal) | control 7 |
| DNAJC5 | TCCYccCCLCCcFNcC | 2.080314 | Control(minimal) | control 7 |
| DNAJC5 | TCCYCCccLCCcFNcC | 2.080314 | Control(minimal) | control 7 |
| DNAJC5 | TCCYccCCLCCcFNcC | 2.080314 | Control(minimal) | control 7 |
| DNAJC5 | TCCYCCccLCCcFNcC | 2.080314 | Control(minimal) | control 7 |
| DNAJC5 | TCCYccCCLCCcFNcC | 2.080314 | Control(minimal) | control 7 |
| DNAJC5 | TCCYCCccLCCcFNcC | 2.080314 | Control(minimal) | control 7 |
| DNAJC5 | TCCYCCccLCCcFNcC | 2.054693 | Control(minimal) | control 7 |
| DNAJC5 | TCCYCCccLCCcFNcC | 2.054693 | Control(minimal) | control 7 |
| DNAJC5 | TCCYCCccLCCcFNcC | 2.054693 | Control(minimal) | control 7 |
| DNAJC5 | TCCYCCccLCCcFNcC | 2.054693 | Control(minimal) | control 7 |
| DNAJC5 | TCCYCCccLCCcFNcC | 2.054693 | Control(minimal) | control 7 |
| DNAJC5 | TCCYCCccLCCcFNcC | 2.054693 | Control(minimal) | control 7 |
| DNAJC5 | TCCYCCccLCCcFNcC | 2.054693 | Control(minimal) | control 7 |
| DNAJC5 | TCCYCCccLCCcFNcC | 2.054693 | Control(minimal) | control 7 |
| DNAJC5 | TCCYCCccLCCcFNcC | 2.054693 | Control(minimal) | control 7 |
| DNAJC5 | TCCYcCCcLCCcFNcC | 1.88794 | Control(minimal) | control 7 |
| DNAJC5 | TCCYcCCcLCCcFNcC | 1.88794 | Control(minimal) | control 7 |
| DNAJC5 | TCCYcCCcLCCcFNcC | 1.88794 | Control(minimal) | control 7 |
| DNAJC5 | TCCYcCCcLCCcFNcC | 1.88794 | Control(minimal) | control 7 |
| DNAJC5 | TCCYcCCcLCCcFNcC | 1.88794 | Control(minimal) | control 7 |
| DNAJC5 | TCCYcCCcLCCcFNcC | 1.88794 | Control(minimal) | control 7 |
| DNAJC5 | TCCYcCCcLCCcFNcC | 1.88794 | Control(minimal) | control 7 |
| DNAJC5 | TCCYcCCcLCCcFNcC | 1.88794 | Control(minimal) | control 7 |
| DNAJC5 | TCCYcCCcLCCcFNcC | 1.88794 | Control(minimal) | control 7 |
| DNAJC5 | TCCYCcCcLCCcFNcC | 1.878683 | Control(minimal) | control 7 |
| DNAJC5 | TCCYCcCcLCCcFNcC | 1.878683 | Control(minimal) | control 7 |
| DNAJC5 | TCCYCcCcLCCcFNcC | 1.878683 | Control(minimal) | control 7 |
| DNAJC5 | TCCYCcCcLCCcFNcC | 1.878683 | Control(minimal) | control 7 |
| DNAJC5 | TCCYCcCcLCCcFNcC | 1.878683 | Control(minimal) | control 7 |
| DNAJC5 | TCCYCcCcLCCcFNcC | 1.878683 | Control(minimal) | control 7 |
| DNAJC5 | TCCYCcCcLCCcFNcC | 1.878683 | Control(minimal) | control 7 |
| DNAJC5 | TCCYCcCcLCCcFNcC | 1.878683 | Control(minimal) | control 7 |
| DNAJC5 | TCCYCcCcLCCcFNcC | 1.878683 | Control(minimal) | control 7 |
| DNAJC5 | TCCYCCccLCCcFNCc | 1.849697 | Control(minimal) | control 7 |
| DNAJC5 | TCCYCCccLCCcFNCc | 1.849697 | Control(minimal) | control 7 |
| DNAJC5 | TCCYCCccLCCcFNCc | 1.849697 | Control(minimal) | control 7 |
| DNAJC5 | TCCYCCccLCCcFNCc | 1.849697 | Control(minimal) | control 7 |
| DNAJC5 | TCCYCCccLCCcFNCc | 1.849697 | Control(minimal) | control 7 |
| DNAJC5 | TCCYCCccLCCcFNCc | 1.849697 | Control(minimal) | control 7 |
| DNAJC5 | TCCYCCccLCCcFNCc | 1.849697 | Control(minimal) | control 7 |
| DNAJC5 | TCCYCCccLCCcFNCc | 1.849697 | Control(minimal) | control 7 |
| DNAJC5 | TCCYCCccLCCcFNCc | 1.849697 | Control(minimal) | control 7 |
| DNAJC5 | TCcYCCCcLCCcFNcC | 1.848457 | Control(minimal) | control 7 |
| DNAJC5 | TCcYCCCcLCCcFNcC | 1.848457 | Control(minimal) | control 7 |
| DNAJC5 | TCcYCCCcLCCcFNcC | 1.848457 | Control(minimal) | control 7 |
| DNAJC5 | TCcYCCCcLCCcFNcC | 1.848457 | Control(minimal) | control 7 |
| DNAJC5 | TCcYCCCcLCCcFNcC | 1.848457 | Control(minimal) | control 7 |
| DNAJC5 | TCcYCCCcLCCcFNcC | 1.848457 | Control(minimal) | control 7 |
| DNAJC5 | TCcYCCCcLCCcFNcC | 1.848457 | Control(minimal) | control 7 |
| DNAJC5 | TCcYCCCcLCCcFNcC | 1.848457 | Control(minimal) | control 7 |
| DNAJC5 | TCcYCCCcLCCcFNcC | 1.848457 | Control(minimal) | control 7 |
| DNAJC5 | TCCYCCccLCCcFNcC | 1.790059 | Control(minimal) | control 7 |
| DNAJC5 | TCCYCCccLCCcFNcC | 1.790059 | Control(minimal) | control 7 |
| DNAJC5 | TCCYCCccLCCcFNcC | 1.790059 | Control(minimal) | control 7 |
| DNAJC5 | TCCYCCccLCCcFNcC | 1.790059 | Control(minimal) | control 7 |
| DNAJC5 | TCCYCCccLCCcFNcC | 1.790059 | Control(minimal) | control 7 |
| DNAJC5 | TCCYCCccLCCcFNcC | 1.790059 | Control(minimal) | control 7 |
| DNAJC5 | TCCYCCccLCCcFNcC | 1.790059 | Control(minimal) | control 7 |
| DNAJC5 | TCCYCCccLCCcFNcC | 1.790059 | Control(minimal) | control 7 |
| DNAJC5 | TCCYCCccLCCcFNcC | 1.790059 | Control(minimal) | control 7 |
| DNAJC5 | TCCYccCCLCCcFNcC | 1.788763 | Control(minimal) | control 7 |
| DNAJC5 | TCCYccCCLCCcFNcC | 1.788763 | Control(minimal) | control 7 |
| DNAJC5 | TCCYccCCLCCcFNcC | 1.788763 | Control(minimal) | control 7 |
| DNAJC5 | TCCYccCCLCCcFNcC | 1.788763 | Control(minimal) | control 7 |
| DNAJC5 | TCCYccCCLCCcFNcC | 1.788763 | Control(minimal) | control 7 |
| DNAJC5 | TCCYccCCLCCcFNcC | 1.788763 | Control(minimal) | control 7 |
| DNAJC5 | TCCYccCCLCCcFNcC | 1.788763 | Control(minimal) | control 7 |
| DNAJC5 | TCCYccCCLCCcFNcC | 1.788763 | Control(minimal) | control 7 |
| DNAJC5 | TCCYccCCLCCcFNcC | 1.788763 | Control(minimal) | control 7 |
| DNAJC5 | TCCYccCCLCCcFNcC | 1.780395 | Control(minimal) | control 7 |
| DNAJC5 | TCCYccCCLCCcFNcC | 1.780395 | Control(minimal) | control 7 |
| DNAJC5 | TCCYccCCLCCcFNcC | 1.780395 | Control(minimal) | control 7 |
| DNAJC5 | TCCYccCCLCCcFNcC | 1.780395 | Control(minimal) | control 7 |
| DNAJC5 | TCCYccCCLCCcFNcC | 1.780395 | Control(minimal) | control 7 |
| DNAJC5 | TCCYccCCLCCcFNcC | 1.780395 | Control(minimal) | control 7 |
| DNAJC5 | TCCYccCCLCCcFNcC | 1.780395 | Control(minimal) | control 7 |
| DNAJC5 | TCCYccCCLCCcFNcC | 1.780395 | Control(minimal) | control 7 |
| DNAJC5 | TCCYccCCLCCcFNcC | 1.780395 | Control(minimal) | control 7 |
| DNAJC5 | CcCcLcCCFNCCcGK | 1.854487 | Control(minimal) | control 13 |
| DNAJC5 | CcCcLcCCFNCCcGK | 1.854487 | Control(minimal) | control 13 |
| DNAJC5 | CcCcLcCCFNCCcGK | 1.854487 | Control(minimal) | control 13 |
| DNAJC5 | cCcCLcCCFNCCcGK | 1.854487 | Control(minimal) | control 13 |
| DNAJC5 | CcCcLcCCFNCCcGK | 1.854487 | Control(minimal) | control 13 |
| DNAJC5 | cCcCLcCCFNCCcGK | 1.854487 | Control(minimal) | control 13 |
| DNAJC5 | CcCcLcCCFNCCcGK | 1.854487 | Control(minimal) | control 13 |
| DNAJC5 | cCcCLcCCFNCCcGK | 1.854487 | Control(minimal) | control 13 |
| DNAJC5 | CcCcLcCCFNCCcGK | 1.854487 | Control(minimal) | control 13 |
| DNAJC5 | cCCcLcCCFNCCcGK | 1.854487 | Control(minimal) | control 13 |
| DNAJC5 | CcCcLcCCFNCCcGK | 1.854487 | Control(minimal) | control 13 |
| DNAJC5 | cCcCLcCCFNCCcGK | 1.854487 | Control(minimal) | control 13 |
| DNAJC5 | CcCcLcCCFNCCcGK | 1.854487 | Control(minimal) | control 13 |
| DNAJC5 | cCcCLcCCFNCCcGK | 1.854487 | Control(minimal) | control 13 |
| DNAJC5 | CcCcLcCCFNCCcGK | 1.854487 | Control(minimal) | control 13 |
| DNAJC5 | cCcCLcCCFNCCcGK | 1.854487 | Control(minimal) | control 13 |
| DNAJC5 | CcCcLcCCFNCCcGK | 1.854487 | Control(minimal) | control 13 |
| DNAJC5 | cCcCLcCCFNCCcGK | 1.854487 | Control(minimal) | control 13 |
| DNAJC5 | CcCcLcCCFNCCcGK | 1.854487 | Control(minimal) | control 13 |
| DNAJC5 | cCcCLcCCFNCCcGK | 1.854487 | Control(minimal) | control 13 |
| DNAJC5 | CcCcLcCCFNCCcGK | 1.854487 | Control(minimal) | control 13 |
| DNAJC5 | cCcCLcCCFNCCcGK | 1.854487 | Control(minimal) | control 13 |
| DNAJC5 | CcCcLcCCFNCCcGK | 1.854487 | Control(minimal) | control 13 |
| DNAJC5 | cCcCLcCCFNCCcGK | 1.854487 | Control(minimal) | control 13 |
| DNAJC5 | CcCcLcCCFNCCcGK | 1.854487 | Control(minimal) | control 13 |
| DNAJC5 | cCCcLcCCFNCCcGK | 1.836408 | Control(minimal) | control 13 |
| DNAJC5 | cCCcLcCCFNCCcGK | 1.836408 | Control(minimal) | control 13 |
| DNAJC5 | cCCcLcCCFNCCcGK | 1.836408 | Control(minimal) | control 13 |
| DNAJC5 | cCCcLcCCFNCCcGK | 1.836408 | Control(minimal) | control 13 |
| DNAJC5 | cCCcLcCCFNCCcGK | 1.836408 | Control(minimal) | control 13 |
| DNAJC5 | cCCcLcCCFNCCcGK | 1.836408 | Control(minimal) | control 13 |
| DNAJC5 | cCCcLcCCFNCCcGK | 1.836408 | Control(minimal) | control 13 |
| DNAJC5 | cCCcLcCCFNCCcGK | 1.836408 | Control(minimal) | control 13 |
| DNAJC5 | cCCcLcCCFNCCcGK | 1.836408 | Control(minimal) | control 13 |
| DNAJC5 | cCCcLcCCFNCCcGK | 1.836408 | Control(minimal) | control 13 |
| DNAJC5 | cCCcLcCCFNCCcGK | 1.836408 | Control(minimal) | control 13 |
| DNAJC5 | cCCcLcCCFNCCcGK | 1.836408 | Control(minimal) | control 13 |
| DNAJC5 | cCCcLcCCFNCCcGK | 1.836408 | Control(minimal) | control 13 |
| DNAJC5B | ALFLLGGQTFMcDKLYL | 2.049965 | TBI(extreme) | TBI 10 |
| DNAJC5B | GLLTGCYFCCcLcCcc | 2.049965 | TBI(extreme) | TBI 10 |
| DNAJC5B | GLLTGCYFCCcLcCcc | 2.049965 | TBI(extreme) | TBI 10 |
| DNAJC5B | GLLTGCYFCCcLcCcc | 2.049965 | TBI(extreme) | TBI 10 |
| DNAJC5B | GLLTGCYFCCcLcCcc | 2.049965 | TBI(extreme) | TBI 10 |
| DNAJC5B | GLLTGCYFCCcLcCcc | 2.049965 | TBI(extreme) | TBI 10 |
| DNAJC5B | GLLTGCYFCCcLcCcc | 2.049965 | TBI(extreme) | TBI 10 |
| DNAJC5B | GLLTGCYFCCcLcCcc | 2.049965 | TBI(extreme) | TBI 10 |
| DNAJC5B | GLLTGCYFCCcLcCcc | 2.049965 | TBI(extreme) | TBI 10 |
| DNAJC5B | GLLTGCYFCCcLcCcc | 2.049965 | TBI(extreme) | TBI 10 |
| DNAJC5B | GLLTGCYFCCcLcCcc | 2.049965 | TBI(extreme) | TBI 10 |
| DNAJC5B | GLLTGCYFCCcLcCcc | 2.049965 | TBI(extreme) | TBI 10 |
| DNAJC5B | GLLTGCYFCCcLcCcc | 2.049965 | TBI(extreme) | TBI 10 |
| DNAJC5B | GLLTGCYFCCcLcCcc | 2.049965 | TBI(extreme) | TBI 10 |
| DNAJC5B | GLLTGCYFCCcLcCcc | 2.049965 | TBI(extreme) | TBI 10 |
| DNAJC5B | GLLTGCYFCCcLcCcc | 2.049965 | TBI(extreme) | TBI 10 |
| DNAJC5B | GLLTGCYFCCcLcCcc | 2.049965 | TBI(extreme) | TBI 10 |
| DNAJC5B | GLLTGCYFCCcLcCcc | 2.049965 | TBI(extreme) | TBI 10 |
| DNAJC5B | LILVPEFSLPLEy | 2.049965 | TBI(extreme) | TBI 10 |
| DNAJC5B | TLTSHtSNLSWI | 2.049965 | TBI(extreme) | TBI 10 |
| DNAJC5B | TLTSHtSNLSWI | 2.049965 | TBI(extreme) | TBI 10 |
| DNAJC5B | TLTSHtSNLSWI | 2.049965 | TBI(extreme) | TBI 10 |
| DNAJC5B | TLTSHtSNLSWI | 2.049965 | TBI(extreme) | TBI 10 |
| DNAJC5B | TLTSHtSNLSWI | 2.049965 | TBI(extreme) | TBI 10 |
| DNAJC5B | TLTSHtSNLSWI | 2.049965 | TBI(extreme) | TBI 10 |
| DNAJC5B | TLTSHtSNLSWI | 2.049965 | TBI(extreme) | TBI 10 |
| DNAJC5B | TLTSHtSNLSWI | 2.049965 | TBI(extreme) | TBI 10 |
| DNAJC5B | TLTSHtSNLSWI | 2.049965 | TBI(extreme) | TBI 10 |
| DNAJC5B | TLTSHtSNLSWI | 2.049965 | TBI(extreme) | TBI 10 |
| DNAJC5B | yTcPNGTcIGFQLVCDG | 2.049965 | TBI(extreme) | TBI 10 |
| DNAJC5B | GLLTGCYFCCcLcccC | 1.956163 | TBI(extreme) | TBI 10 |
| DNAJC5B | GLLTGCYFCCcLcccC | 1.956163 | TBI(extreme) | TBI 10 |
| DNAJC5B | GLLTGCYFCCcLcccC | 1.956163 | TBI(extreme) | TBI 10 |
| DNAJC5B | GLLTGCYFCCcLcccC | 1.956163 | TBI(extreme) | TBI 10 |
| DNAJC5B | GLLTGCYFCCcLcccC | 1.956163 | TBI(extreme) | TBI 10 |
| DNAJC5B | GLLTGCYFCCcLcccC | 1.956163 | TBI(extreme) | TBI 10 |
| DNAJC5B | GLLTGCYFCCcLcccC | 1.956163 | TBI(extreme) | TBI 10 |
| DNAJC5B | GLLTGCYFCCcLcccC | 1.956163 | TBI(extreme) | TBI 10 |
| DNAJC5B | GLLTGCYFCCcLcccC | 1.956163 | TBI(extreme) | TBI 10 |
| DNAJC5B | GLLTGCYFCCcLcccC | 1.956163 | TBI(extreme) | TBI 10 |
| DNAJC5B | GLLTGCYFCCcLcccC | 1.954545 | TBI(extreme) | TBI 10 |
| DNAJC5B | GLLTGCYFCCcLcccC | 1.954545 | TBI(extreme) | TBI 10 |
| DNAJC5B | GLLTGCYFCCcLcccC | 1.954545 | TBI(extreme) | TBI 10 |
| DNAJC5B | GLLTGCYFCCcLcccC | 1.954545 | TBI(extreme) | TBI 10 |
| DNAJC5B | GLLTGCYFCCcLcccC | 1.954545 | TBI(extreme) | TBI 10 |
| DNAJC5B | GLLTGCYFCCcLcccC | 1.954545 | TBI(extreme) | TBI 10 |
| DNAJC5B | GLLTGCYFCCcLcccC | 1.954545 | TBI(extreme) | TBI 10 |
| DNAJC5B | GLLTGCYFCCcLcccC | 1.954545 | TBI(extreme) | TBI 10 |
| DNAJC5B | GLLTGCYFCCcLcccC | 1.954545 | TBI(extreme) | TBI 10 |
| DNAJC5B | GLLTGCYFCCcLcccC | 1.954545 | TBI(extreme) | TBI 10 |
| DNAJC5B | GLLTGCYFCCcLccCc | 1.950311 | TBI(extreme) | TBI 10 |
| DNAJC5B | GLLTGCYFCCcLccCc | 1.950311 | TBI(extreme) | TBI 10 |
| DNAJC5B | GLLTGCYFCCcLccCc | 1.950311 | TBI(extreme) | TBI 10 |
| DNAJC5B | GLLTGCYFCCcLccCc | 1.950311 | TBI(extreme) | TBI 10 |
| DNAJC5B | GLLTGCYFCCcLccCc | 1.950311 | TBI(extreme) | TBI 10 |
| DNAJC5B | GLLTGCYFCCcLccCc | 1.950311 | TBI(extreme) | TBI 10 |
| DNAJC5B | GLLTGCYFCCcLccCc | 1.950311 | TBI(extreme) | TBI 10 |
| DNAJC5B | GLLTGCYFCCcLccCc | 1.950311 | TBI(extreme) | TBI 10 |
| DNAJC5B | GLLTGCYFCCcLccCc | 1.950311 | TBI(extreme) | TBI 10 |
| DNAJC5B | GLLTGCYFCCcLccCc | 1.950311 | TBI(extreme) | TBI 10 |
| DNAJC5B | GLLTGCYFCCcLccCc | 1.948694 | TBI(extreme) | TBI 10 |
| DNAJC5B | GLLTGCYFCCcLccCc | 1.948694 | TBI(extreme) | TBI 10 |
| DNAJC5B | GLLTGCYFCCcLccCc | 1.948694 | TBI(extreme) | TBI 10 |
| DNAJC5B | GLLTGCYFCCcLccCc | 1.948694 | TBI(extreme) | TBI 10 |
| DNAJC5B | GLLTGCYFCCcLccCc | 1.948694 | TBI(extreme) | TBI 10 |
| DNAJC5B | GLLTGCYFCCcLccCc | 1.948694 | TBI(extreme) | TBI 10 |
| DNAJC5B | GLLTGCYFCCcLccCc | 1.948694 | TBI(extreme) | TBI 10 |
| DNAJC5B | GLLTGCYFCCcLccCc | 1.948694 | TBI(extreme) | TBI 10 |
| DNAJC5B | GLLTGCYFCCcLccCc | 1.948694 | TBI(extreme) | TBI 10 |
| DNAJC5B | GLLTGCYFCCcLccCc | 1.948694 | TBI(extreme) | TBI 10 |
| DNAJC5B | GLLTGCYFCCcLcccC | 1.917411 | TBI(extreme) | TBI 10 |
| DNAJC5B | GLLTGCYFCCcLcccC | 1.917411 | TBI(extreme) | TBI 10 |
| DNAJC5B | GLLTGCYFCCcLcccC | 1.917411 | TBI(extreme) | TBI 10 |
| DNAJC5B | GLLTGCYFCCcLcccC | 1.917411 | TBI(extreme) | TBI 10 |
| DNAJC5B | GLLTGCYFCCcLcccC | 1.917411 | TBI(extreme) | TBI 10 |
| DNAJC5B | GLLTGCYFCCcLcccC | 1.917411 | TBI(extreme) | TBI 10 |
| DNAJC5B | GLLTGCYFCCcLcccC | 1.917411 | TBI(extreme) | TBI 10 |
| DNAJC5B | GLLTGCYFCCcLcccC | 1.917411 | TBI(extreme) | TBI 10 |
| DNAJC5B | GLLTGCYFCCcLcccC | 1.917411 | TBI(extreme) | TBI 10 |
| DNAJC5B | GLLTGCYFCCcLcccC | 1.917411 | TBI(extreme) | TBI 10 |
| DNAJC5B | GLLTGCYFCCcLcccC | 1.913489 | TBI(extreme) | TBI 10 |
| DNAJC5B | GLLTGCYFCCcLcccC | 1.913489 | TBI(extreme) | TBI 10 |
| DNAJC5B | GLLTGCYFCCcLcCcc | 1.913489 | TBI(extreme) | TBI 10 |
| DNAJC5B | GLLTGCYFCCcLcccC | 1.913489 | TBI(extreme) | TBI 10 |
| DNAJC5B | GLLTGCYFCCcLcccC | 1.913489 | TBI(extreme) | TBI 10 |
| DNAJC5B | GLLTGCYFCCcLcccC | 1.913489 | TBI(extreme) | TBI 10 |
| DNAJC5B | GLLTGCYFCCcLcccC | 1.913489 | TBI(extreme) | TBI 10 |
| DNAJC5B | GLLTGCYFCCcLcccC | 1.913489 | TBI(extreme) | TBI 10 |
| DNAJC5B | GLLTGCYFCCcLcccC | 1.913489 | TBI(extreme) | TBI 10 |
| DNAJC5B | GLLTGCYFCCcLcccC | 1.913489 | TBI(extreme) | TBI 10 |
| DNAJC5B | GLLTGCYFCCcLcccC | 1.913489 | TBI(extreme) | TBI 10 |
| DNAJC5B | GLLTGCYFCCcLcCcc | 1.911284 | TBI(extreme) | TBI 10 |
| DNAJC5B | GLLTGCYFCCcLccCc | 1.908741 | TBI(extreme) | TBI 10 |
| DNAJC5B | GLLTGCYFCCcLccCc | 1.908741 | TBI(extreme) | TBI 10 |
| DNAJC5B | GLLTGCYFCCcLccCc | 1.908741 | TBI(extreme) | TBI 10 |
| DNAJC5B | GLLTGCYFCCcLccCc | 1.908741 | TBI(extreme) | TBI 10 |
| DNAJC5B | GLLTGCYFCCcLccCc | 1.908741 | TBI(extreme) | TBI 10 |
| DNAJC5B | GLLTGCYFCCcLccCc | 1.908741 | TBI(extreme) | TBI 10 |
| DNAJC5B | GLLTGCYFCCcLccCc | 1.908741 | TBI(extreme) | TBI 10 |
| DNAJC5B | GLLTGCYFCCcLccCc | 1.908741 | TBI(extreme) | TBI 10 |
| DNAJC5B | GLLTGCYFCCcLccCc | 1.908741 | TBI(extreme) | TBI 10 |
| DNAJC5B | GLLTGCYFCCcLccCc | 1.908741 | TBI(extreme) | TBI 10 |
| DNAJC5B | GLLTGCYFCCcLccCc | 1.907638 | TBI(extreme) | TBI 10 |
| DNAJC5B | GLLTGCYFCCcLccCc | 1.907638 | TBI(extreme) | TBI 10 |
| DNAJC5B | GLLTGCYFCCcLccCc | 1.907638 | TBI(extreme) | TBI 10 |
| DNAJC5B | GLLTGCYFCCcLccCc | 1.907638 | TBI(extreme) | TBI 10 |
| DNAJC5B | GLLTGCYFCCcLccCc | 1.907638 | TBI(extreme) | TBI 10 |
| DNAJC5B | GLLTGCYFCCcLccCc | 1.907638 | TBI(extreme) | TBI 10 |
| DNAJC5B | GLLTGCYFCCcLcCcc | 1.907638 | TBI(extreme) | TBI 10 |
| DNAJC5B | GLLTGCYFCCcLccCc | 1.907638 | TBI(extreme) | TBI 10 |
| DNAJC5B | GLLTGCYFCCcLccCc | 1.907638 | TBI(extreme) | TBI 10 |
| DNAJC5B | GLLTGCYFCCcLccCc | 1.907638 | TBI(extreme) | TBI 10 |
| DNAJC5B | GLLTGCYFCCcLccCc | 1.907638 | TBI(extreme) | TBI 10 |
| DNAJC5B | GLLTGCYFCCcLcCcc | 1.903928 | TBI(extreme) | TBI 10 |
| DNAJC5B | GLLTGCYFCCcLcCcc | 1.903646 | TBI(extreme) | TBI 10 |
| DNAJC5B | GLLTGCYFCCcLcCcc | 1.903646 | TBI(extreme) | TBI 10 |
| DNAJC5B | GLLTGCYFCCcLcCcc | 1.903646 | TBI(extreme) | TBI 10 |
| DNAJC5B | GLLTGCYFCCcLcCcc | 1.903646 | TBI(extreme) | TBI 10 |
| DNAJC5B | GLLTGCYFCCcLcCcc | 1.903646 | TBI(extreme) | TBI 10 |
| DNAJC5B | GLLTGCYFCCcLcCcc | 1.903646 | TBI(extreme) | TBI 10 |
| DNAJC5B | GLLTGCYFCCcLcCcc | 1.903646 | TBI(extreme) | TBI 10 |
| DNAJC5B | GLLTGCYFCCcLcCcc | 1.903646 | TBI(extreme) | TBI 10 |
| DNAJC5B | GLLTGCYFCCcLcCcc | 1.903646 | TBI(extreme) | TBI 10 |
| DNAJC5B | GLLTGCYFCCcLcCcc | 1.903646 | TBI(extreme) | TBI 10 |
| DNAJC5B | GLLTGCYFCCcLcCcc | 1.894403 | TBI(extreme) | TBI 10 |
| DNAJC5B | FCCCLccCCNcc | 1.767117 | TBI(extreme) | TBI 30 |
| DNAJC5B | FCCCLccCCNcc | 1.767117 | TBI(extreme) | TBI 30 |
| DNAJC5B | FCCCLccCCNcc | 1.767117 | TBI(extreme) | TBI 30 |
| DNAJC5B | FCCcLCCcCNcc | 1.767117 | TBI(extreme) | TBI 30 |
| DNAJC5B | FCCCLccCCNcc | 1.767117 | TBI(extreme) | TBI 30 |
| DNAJC5B | FCCCLcCcCNcc | 1.767117 | TBI(extreme) | TBI 30 |
| DNAJC5B | FCCCLccCCNcc | 1.767117 | TBI(extreme) | TBI 30 |
| DNAJC5B | FCCcLCCcCNcc | 1.767117 | TBI(extreme) | TBI 30 |
| DNAJC5B | FCCCLccCCNcc | 1.767117 | TBI(extreme) | TBI 30 |
| DNAJC5B | FCCcLCcCCNcc | 1.767117 | TBI(extreme) | TBI 30 |
| DNAJC5B | FCCCLccCCNcc | 1.767117 | TBI(extreme) | TBI 30 |
| DNAJC5B | FCCcLCCcCNcc | 1.767117 | TBI(extreme) | TBI 30 |
| DNAJC5B | FCCCLccCCNcc | 1.767117 | TBI(extreme) | TBI 30 |
| DNAJC5B | FCCcLCCcCNcc | 1.767117 | TBI(extreme) | TBI 30 |
| DNAJC5B | FCCCLccCCNcc | 1.767117 | TBI(extreme) | TBI 30 |
| DNAJC5B | FCCcLCCcCNcc | 1.767117 | TBI(extreme) | TBI 30 |
| DNAJC5B | FCCCLccCCNcc | 1.767117 | TBI(extreme) | TBI 30 |
| DNAJC5B | FCCCLcCcCNcc | 1.721613 | TBI(extreme) | TBI 30 |
| DNAJC5B | FCCCLcCcCNcc | 1.721613 | TBI(extreme) | TBI 30 |
| DNAJC5B | FCCCLcCcCNcc | 1.721613 | TBI(extreme) | TBI 30 |
| DNAJC5B | FCCCLcCcCNcc | 1.721613 | TBI(extreme) | TBI 30 |
| DNAJC5B | FCCCLcCcCNcc | 1.721613 | TBI(extreme) | TBI 30 |
| DNAJC5B | FCCCLcCcCNcc | 1.721613 | TBI(extreme) | TBI 30 |
| DNAJC5B | FCCCLcCcCNcc | 1.721613 | TBI(extreme) | TBI 30 |
| DNAJC5B | FCCCLcCcCNcc | 1.721613 | TBI(extreme) | TBI 30 |
| DNAJC5B | FCCCLcCcCNcc | 1.721613 | TBI(extreme) | TBI 30 |
| DNAJC5B | GcYFcccLCCCCNCCC | 1.831838 | Control(minimal) | control 9 |
| DNAJC5B | GcYFcccLCCCCNCCC | 1.831838 | Control(minimal) | control 9 |
| DNAJC5B | GcYFcccLCCCCNCCC | 1.831838 | Control(minimal) | control 9 |
| DNAJC5B | GcYFcccLCCCCNCCC | 1.831838 | Control(minimal) | control 11 |
| DNAJC5B | GcYFcccLCCCCNCCC | 1.831838 | Control(minimal) | control 11 |
| DNAJC5B | GcYFcccLCCCCNCCC | 1.831838 | Control(minimal) | control 11 |
| DVL3 | tSFFDSDEDDSTSR | 1.945699 | TBI(extreme) | TBI 1 |
| DVL3 | tSFFDSDEDDSTSR | 1.945699 | TBI(extreme) | TBI 1 |
| DVL3 | tSFFDSDEDDSTSR | 1.945699 | TBI(extreme) | TBI 1 |
| DVL3 | tSFFDSDEDDSTSR | 1.945699 | TBI(extreme) | TBI 1 |
| DVL3 | TsFFDSDEDDSTSR | 1.945699 | TBI(extreme) | TBI 1 |
| DVL3 | TsFFDSDEDDSTSR | 1.945699 | TBI(extreme) | TBI 1 |
| DVL3 | TsFFDSDEDDSTSR | 1.945699 | TBI(extreme) | TBI 1 |
| DVL3 | TsFFDSDEDDSTSR | 1.919376 | TBI(extreme) | TBI 1 |
| DVL3 | tSFFDSDEDDSTSR | 1.945699 | TBI(extreme) | TBI 8 |
| DVL3 | tSFFDSDEDDSTSR | 1.945699 | TBI(extreme) | TBI 8 |
| DVL3 | tSFFDSDEDDSTSR | 1.945699 | TBI(extreme) | TBI 8 |
| DVL3 | tSFFDSDEDDSTSR | 1.945699 | TBI(extreme) | TBI 8 |
| DVL3 | TsFFDSDEDDSTSR | 1.945699 | TBI(extreme) | TBI 8 |
| DVL3 | TsFFDSDEDDSTSR | 1.945699 | TBI(extreme) | TBI 8 |
| DVL3 | TsFFDSDEDDSTSR | 1.945699 | TBI(extreme) | TBI 8 |
| DVL3 | TsFFDSDEDDSTSR | 1.919376 | TBI(extreme) | TBI 8 |
| EBF4 | EPETTPSmATSHGTEA | 1.733862 | TBI(extreme) | TBI 5 |
| EBF4 | EPETTPSmATSHGTEA | 1.733862 | TBI(extreme) | TBI 5 |
| EBF4 | EPETTPSmATSHGTEA | 1.733862 | TBI(extreme) | TBI 5 |
| EBF4 | EPETTPSmATSHGTEA | 1.733862 | TBI(extreme) | TBI 5 |
| EBF4 | EPETTPSmATSHGTEA | 1.733862 | TBI(extreme) | TBI 5 |
| EBF4 | EPETTPSmATSHGTEA | 1.733862 | TBI(extreme) | TBI 5 |
| EBF4 | EPETTPSmATSHGTEA | 1.733862 | TBI(extreme) | TBI 5 |
| EBF4 | EPETTPSmATSHGTEA | 1.733862 | TBI(extreme) | TBI 5 |
| EBF4 | EPETTPSmATSHGTEA | 1.733862 | TBI(extreme) | TBI 5 |
| EBF4 | EPETTPSmATSHGTEA | 1.733862 | TBI(extreme) | TBI 5 |
| EBF4 | EPETTPSmATSHGTEA | 1.733862 | TBI(extreme) | TBI 5 |
| EBF4 | EPETTPSmATSHGTEA | 1.733862 | TBI(extreme) | TBI 5 |
| EBF4 | EPETTPSmATSHGTEA | 1.733862 | TBI(extreme) | TBI 5 |
| EBF4 | EGtHSPVTQRFPHsE | 3.27635 | TBI(extreme) | TBI 9 |
| EBF4 | sTKGIWLEETSADTL | 3.27635 | TBI(extreme) | TBI 9 |
| EBF4 | sTKGIWLEETSADTL | 3.27635 | TBI(extreme) | TBI 9 |
| EBF4 | sTKGIWLEETSADTL | 3.27635 | TBI(extreme) | TBI 9 |
| EBF4 | sTKGIWLEETSADTL | 3.27635 | TBI(extreme) | TBI 9 |
| EBF4 | sTKGIWLEETSADTL | 3.27635 | TBI(extreme) | TBI 9 |
| EBF4 | sTKGIWLEETSADTL | 3.27635 | TBI(extreme) | TBI 9 |
| EBF4 | sTKGIWLEETSADTL | 3.27635 | TBI(extreme) | TBI 9 |
| EBF4 | sTKGIWLEETSADTL | 3.27635 | TBI(extreme) | TBI 9 |
| EBF4 | sTKGIWLEETSADTL | 3.27635 | TBI(extreme) | TBI 9 |
| EBF4 | sTKGIWLEETSADTL | 3.27635 | TBI(extreme) | TBI 9 |
| EBF4 | sTKGIWLEETSADTL | 3.27635 | TBI(extreme) | TBI 9 |
| EBF4 | EGtHSPVTQRFPHsE | 1.796872 | TBI(extreme) | TBI 9 |
| EBF4 | EGtHSPVTQRFPHsE | 1.796872 | TBI(extreme) | TBI 9 |
| EBF4 | EGtHSPVTQRFPHsE | 1.796872 | TBI(extreme) | TBI 9 |
| EBF4 | EGtHSPVTQRFPHsE | 1.796872 | TBI(extreme) | TBI 9 |
| EBF4 | EGtHSPVTQRFPHsE | 1.796872 | TBI(extreme) | TBI 9 |
| EBF4 | EGtHSPVTQRFPHsE | 1.796872 | TBI(extreme) | TBI 9 |
| EBF4 | EGtHSPVTQRFPHsE | 1.796872 | TBI(extreme) | TBI 9 |
| EBF4 | EGtHSPVTQRFPHsE | 1.796872 | TBI(extreme) | TBI 9 |
| EBF4 | EGtHSPVTQRFPHsE | 1.796872 | TBI(extreme) | TBI 9 |
| EBF4 | EGtHSPVTQRFPHsE | 1.796872 | TBI(extreme) | TBI 9 |
| EBF4 | EGtHSPVTQRFPHsE | 1.796872 | TBI(extreme) | TBI 9 |
| EBF4 | EGtHSPVTQRFPHsE | 1.796872 | TBI(extreme) | TBI 9 |
| EBF4 | EGtHSPVTQRFPHsE | 1.796872 | TBI(extreme) | TBI 9 |
| EBF4 | GHItSAVTPAAMARSSG | 3.516287 | Control(minimal) | control 12 |
| EBF4 | GHItSAVTPAAMARSSG | 3.516287 | Control(minimal) | control 12 |
| EBF4 | GHItSAVTPAAMARSSG | 3.516287 | Control(minimal) | control 12 |
| EBF4 | GHItSAVTPAAMARSSG | 3.516287 | Control(minimal) | control 12 |
| EBF4 | GHItSAVTPAAMARSSG | 3.516287 | Control(minimal) | control 12 |
| EBF4 | GHItSAVTPAAMARSSG | 3.516287 | Control(minimal) | control 12 |
| EBF4 | GHItSAVTPAAMARSSG | 3.516287 | Control(minimal) | control 12 |
| EBF4 | GHItSAVTPAAMARSSG | 3.516287 | Control(minimal) | control 12 |
| EBF4 | GHItSAVTPAAMARSSG | 3.516287 | Control(minimal) | control 12 |
| EBF4 | GHItSAVTPAAMARSSG | 3.516287 | Control(minimal) | control 12 |
| EBF4 | GHItSAVTPAAMARSSG | 3.516287 | Control(minimal) | control 12 |
| EBF4 | GHItSAVTPAAMARSSG | 3.516287 | Control(minimal) | control 12 |
| EBF4 | GHItSAVTPAAMARSSG | 3.516287 | Control(minimal) | control 12 |
| EBF4 | GHItSAVTPAAMARSSG | 3.516287 | Control(minimal) | control 12 |
| EBF4 | GHItSAVTPAAMARSSG | 3.516287 | Control(minimal) | control 12 |
| EBF4 | GHItSAVTPAAMARSSG | 3.516287 | Control(minimal) | control 12 |
| EBF4 | tPSPGEPETTASSATHP | 3.516287 | Control(minimal) | control 12 |
| EBF4 | TPsPGEPETTASSATHP | 3.516287 | Control(minimal) | control 12 |
| EBF4 | tPSPGEPETTASSATHP | 1.893695 | Control(minimal) | control 12 |
| EBF4 | tPSPGEPETTASSATHP | 1.893695 | Control(minimal) | control 12 |
| EBF4 | tPSPGEPETTASSATHP | 1.893695 | Control(minimal) | control 12 |
| EBF4 | tPSPGEPETTASSATHP | 1.893695 | Control(minimal) | control 12 |
| EBF4 | tPSPGEPETTASSATHP | 1.893695 | Control(minimal) | control 12 |
| EBF4 | tPSPGEPETTASSATHP | 1.893695 | Control(minimal) | control 12 |
| EBF4 | tPSPGEPETTASSATHP | 1.893695 | Control(minimal) | control 12 |
| EBF4 | tPSPGEPETTASSATHP | 1.893695 | Control(minimal) | control 12 |
| EBF4 | tPSPGEPETTASSATHP | 1.893695 | Control(minimal) | control 12 |
| EBF4 | tPSPGEPETTASSATHP | 1.893695 | Control(minimal) | control 12 |
| EBF4 | tPSPGEPETTASSATHP | 1.893695 | Control(minimal) | control 12 |
| EBF4 | tPSPGEPETTASSATHP | 1.893695 | Control(minimal) | control 12 |
| EBF4 | tPSPGEPETTASSATHP | 1.893695 | Control(minimal) | control 12 |
| EBF4 | tPSPGEPETTASSATHP | 1.893695 | Control(minimal) | control 12 |
| EBF4 | tPSPGEPETTASSATHP | 1.893695 | Control(minimal) | control 12 |
| EBF4 | tPSPGEPETTASSATHP | 1.893695 | Control(minimal) | control 12 |
| EBF4 | tPSPGEPETTASSATHP | 1.893695 | Control(minimal) | control 12 |
| EBF4 | tPSPGEPETTASSATHP | 1.893695 | Control(minimal) | control 12 |
| EBF4 | tPSPGEPETTASSATHP | 1.893695 | Control(minimal) | control 12 |
| EBF4 | IsLVTHPAESS | 3.217055 | Control(minimal) | control 13 |
| EBF4 | IsLVTHPAESS | 3.217055 | Control(minimal) | control 13 |
| EBF4 | IsLVTHPAESS | 3.217055 | Control(minimal) | control 13 |
| EBF4 | IsLVTHPAESS | 3.217055 | Control(minimal) | control 13 |
| EBF4 | IsLVTHPAESS | 3.217055 | Control(minimal) | control 13 |
| EBF4 | IsLVTHPAESS | 3.217055 | Control(minimal) | control 13 |
| EBF4 | IsLVTHPAESS | 3.217055 | Control(minimal) | control 13 |
| EBF4 | IsLVTHPAESS | 3.217055 | Control(minimal) | control 13 |
| EBF4 | IsLVTHPAESS | 3.217055 | Control(minimal) | control 13 |
| EBF4 | IsLVTHPAESS | 3.217055 | Control(minimal) | control 13 |
| EBF4 | IsLVTHPAESS | 3.217055 | Control(minimal) | control 13 |
| EBF4 | KtRPSFSLMSSPATT | 3.217055 | Control(minimal) | control 13 |
| EBF4 | KtRPSFSLMSSPATT | 1.861735 | Control(minimal) | control 13 |
| EBF4 | KtRPSFSLMSSPATT | 1.861735 | Control(minimal) | control 13 |
| EBF4 | KtRPSFSLMSSPATT | 1.861735 | Control(minimal) | control 13 |
| EBF4 | KtRPSFSLMSSPATT | 1.861735 | Control(minimal) | control 13 |
| EBF4 | KtRPSFSLMSSPATT | 1.861735 | Control(minimal) | control 13 |
| EBF4 | KtRPSFSLMSSPATT | 1.861735 | Control(minimal) | control 13 |
| EBF4 | KtRPSFSLMSSPATT | 1.861735 | Control(minimal) | control 13 |
| EBF4 | KtRPSFSLMSSPATT | 1.861735 | Control(minimal) | control 13 |
| EBF4 | KtRPSFSLMSSPATT | 1.861735 | Control(minimal) | control 13 |
| EBF4 | KtRPSFSLMSSPATT | 1.861735 | Control(minimal) | control 13 |
| EBF4 | KtRPSFSLMSSPATT | 1.861735 | Control(minimal) | control 13 |
| EBF4 | KtRPSFSLMSSPATT | 1.861735 | Control(minimal) | control 13 |
| EBF4 | KtRPSFSLMSSPATT | 1.861735 | Control(minimal) | control 13 |
| EFL1 | GVCFVLEKWDLsKF | 1.879684 | TBI(extreme) | TBI 5 |
| EFL1 | GVCFVLEKWDLsKF | 1.879684 | TBI(extreme) | TBI 5 |
| EFL1 | GVCFVLEKWDLsKF | 1.879684 | TBI(extreme) | TBI 5 |
| EFL1 | GVCFVLEKWDLsKF | 1.879684 | TBI(extreme) | TBI 5 |
| EFL1 | GVCFVLEKWDLsKF | 1.879684 | TBI(extreme) | TBI 5 |
| EFL1 | GVCFVLEKWDLsKF | 1.879684 | TBI(extreme) | TBI 5 |
| EFL1 | GVCFVLEKWDLsKF | 1.879684 | TBI(extreme) | TBI 5 |
| EFL1 | GVCFVLEKWDLsKF | 1.879684 | TBI(extreme) | TBI 5 |
| EFL1 | GVCFVLEKWDLsKF | 1.879684 | TBI(extreme) | TBI 5 |
| EFL1 | GVCFVLEKWDLsKF | 1.879684 | TBI(extreme) | TBI 5 |
| EFL1 | GVCFVLEKWDLsKF | 1.879684 | TBI(extreme) | TBI 5 |
| EFL1 | GVCFVLEKWDLsKF | 1.879684 | TBI(extreme) | TBI 5 |
| EFL1 | GVCFVLEKWDLsKF | 1.879684 | TBI(extreme) | TBI 5 |
| EFL1 | GVCFVLEKWDLsKF | 1.879684 | TBI(extreme) | TBI 5 |
| EFL1 | GVCFVLEKWDLsKF | 1.879684 | TBI(extreme) | TBI 5 |
| EFL1 | GVCFVLEKWDLsKF | 1.879684 | TBI(extreme) | TBI 5 |
| EFL1 | GVCFVLEKWDLsKF | 1.879684 | TBI(extreme) | TBI 5 |
| EFL1 | PLtDCYGPFSGQLIA | 1.766082 | TBI(extreme) | TBI 9 |
| EFL1 | PLtDCYGPFSGQLIA | 1.766082 | TBI(extreme) | TBI 9 |
| EFL1 | PLtDCYGPFSGQLIA | 1.766082 | TBI(extreme) | TBI 9 |
| EFL1 | PLTDCyGPFSGQLIA | 1.766082 | TBI(extreme) | TBI 9 |
| EFL1 | PLtDCYGPFSGQLIA | 1.766082 | TBI(extreme) | TBI 9 |
| EFL1 | PLTDCyGPFSGQLIA | 1.766082 | TBI(extreme) | TBI 9 |
| EFL1 | PLtDCYGPFSGQLIA | 1.766082 | TBI(extreme) | TBI 9 |
| EFL1 | PLTDCyGPFSGQLIA | 1.766082 | TBI(extreme) | TBI 9 |
| EFL1 | PLtDCYGPFSGQLIA | 1.766082 | TBI(extreme) | TBI 9 |
| EFL1 | PLTDCyGPFSGQLIA | 1.766082 | TBI(extreme) | TBI 9 |
| EFL1 | PLtDCYGPFSGQLIA | 1.766082 | TBI(extreme) | TBI 9 |
| EFL1 | PLTDCyGPFSGQLIA | 1.766082 | TBI(extreme) | TBI 9 |
| EFL1 | PLtDCYGPFSGQLIA | 1.766082 | TBI(extreme) | TBI 9 |
| EFL1 | PLTDCyGPFSGQLIA | 1.766082 | TBI(extreme) | TBI 9 |
| EFL1 | PLtDCYGPFSGQLIA | 1.766082 | TBI(extreme) | TBI 9 |
| EFL1 | PLTDCyGPFSGQLIA | 1.766082 | TBI(extreme) | TBI 9 |
| EFL1 | PLtDCYGPFSGQLIA | 1.766082 | TBI(extreme) | TBI 9 |
| EFL1 | PLTDCyGPFSGQLIA | 1.766082 | TBI(extreme) | TBI 9 |
| EFL1 | PLtDCYGPFSGQLIA | 1.766082 | TBI(extreme) | TBI 9 |
| EFL1 | PLTDCyGPFSGQLIA | 1.766082 | TBI(extreme) | TBI 9 |
| EFL1 | PLtDCYGPFSGQLIA | 1.766082 | TBI(extreme) | TBI 9 |
| EFL1 | PLTDCyGPFSGQLIA | 1.766082 | TBI(extreme) | TBI 9 |
| EFL1 | PLtDCYGPFSGQLIA | 1.766082 | TBI(extreme) | TBI 9 |
| EFL1 | PLTDCyGPFSGQLIA | 1.766082 | TBI(extreme) | TBI 9 |
| EFL1 | PLtDCYGPFSGQLIA | 1.766082 | TBI(extreme) | TBI 9 |
| EFL1 | PLTDCyGPFSGQLIA | 1.766082 | TBI(extreme) | TBI 9 |
| EFL1 | PLtDCYGPFSGQLIA | 1.766082 | TBI(extreme) | TBI 9 |
| EFL1 | PLTDCyGPFSGQLIA | 1.766082 | TBI(extreme) | TBI 9 |
| EFL1 | PLtDCYGPFSGQLIA | 1.766082 | TBI(extreme) | TBI 9 |
| EFL1 | PLTDCyGPFSGQLIA | 1.766082 | TBI(extreme) | TBI 9 |
| EFL1 | PLtDCYGPFSGQLIA | 1.766082 | TBI(extreme) | TBI 9 |
| EFL1 | PLTDCyGPFSGQLIA | 1.766082 | TBI(extreme) | TBI 9 |
| EFL1 | PLtDCYGPFSGQLIA | 1.766082 | TBI(extreme) | TBI 9 |
| FAM208B | DGIcDAGFSLVMTP | 1.768044 | TBI(extreme) | TBI 5 |
| FAM208B | DGIcDAGFSLVMTP | 1.768044 | TBI(extreme) | TBI 5 |
| FAM208B | DGIcDAGFSLVMTP | 1.768044 | TBI(extreme) | TBI 5 |
| FAM208B | DGIcDAGFSLVMTP | 1.768044 | TBI(extreme) | TBI 5 |
| FAM208B | DGIcDAGFSLVMTP | 1.768044 | TBI(extreme) | TBI 5 |
| FAM208B | DGIcDAGFSLVMTP | 1.768044 | TBI(extreme) | TBI 5 |
| FAM208B | DGIcDAGFSLVMTP | 1.768044 | TBI(extreme) | TBI 5 |
| FAM208B | PRGSStKIPVPRPRSP | 1.959335 | TBI(extreme) | TBI 30 |
| FAM208B | PRGSStKIPVPRPRSP | 1.959335 | TBI(extreme) | TBI 30 |
| FAM208B | PRGSStKIPVPRPRSP | 1.959335 | TBI(extreme) | TBI 30 |
| FAM208B | PRGsSTKIPVPRPRSP | 1.959335 | TBI(extreme) | TBI 30 |
| FAM208B | PRGSStKIPVPRPRSP | 1.959335 | TBI(extreme) | TBI 30 |
| FAM208B | PRGSsTKIPVPRPRSP | 1.959335 | TBI(extreme) | TBI 30 |
| FAM208B | PRGSStKIPVPRPRSP | 1.959335 | TBI(extreme) | TBI 30 |
| FAM208B | PRGsSTKIPVPRPRSP | 1.810269 | TBI(extreme) | TBI 30 |
| FAM208B | PRGsSTKIPVPRPRSP | 1.810269 | TBI(extreme) | TBI 30 |
| FAM208B | PRGsSTKIPVPRPRSP | 1.810269 | TBI(extreme) | TBI 30 |
| FAM208B | PRGsSTKIPVPRPRSP | 1.810269 | TBI(extreme) | TBI 30 |
| FAM208B | PRGSsTKIPVPRPRSP | 1.725686 | TBI(extreme) | TBI 30 |
| FAM208B | PRGSsTKIPVPRPRSP | 1.725686 | TBI(extreme) | TBI 30 |
| FAM208B | PRGSsTKIPVPRPRSP | 1.725686 | TBI(extreme) | TBI 30 |
| FAM208B | PRGSsTKIPVPRPRSP | 1.725686 | TBI(extreme) | TBI 30 |
| FANCA | FEKLISSERNCL | 1.700739 | TBI(extreme) | TBI 5 |
| FANCA | RtRDSLFFcLKFCT | 2.003752 | Control(minimal) | control 10 |
| FANCA | RtRDSLFFcLKFCT | 2.003752 | Control(minimal) | control 10 |
| FANCA | RtRDSLFFcLKFCT | 2.003752 | Control(minimal) | control 10 |
| FANCA | RtRDSLFFcLKFCT | 2.003752 | Control(minimal) | control 10 |
| FANCA | RtRDSLFFcLKFCT | 2.003752 | Control(minimal) | control 10 |
| FANCA | RtRDSLFFcLKFCT | 2.003752 | Control(minimal) | control 10 |
| FANCA | RtRDSLFFcLKFCT | 2.003752 | Control(minimal) | control 10 |
| FANCA | RtRDSLFFcLKFCT | 2.003752 | Control(minimal) | control 10 |
| FANCA | QAFEScQLDSMVTAF | 1.782909 | Control(minimal) | control 13 |
| FANCA | QAFEScQLDSMVTAF | 1.782909 | Control(minimal) | control 13 |
| FANCA | QAFEScQLDSMVTAF | 1.782909 | Control(minimal) | control 13 |
| FANCA | QAFEScQLDSMVTAF | 1.782909 | Control(minimal) | control 13 |
| FANCA | QAFEScQLDSMVTAF | 1.782909 | Control(minimal) | control 13 |
| FANCA | QAFEScQLDSMVTAF | 1.782909 | Control(minimal) | control 13 |
| FANCA | QAFEScQLDSMVTAF | 1.782909 | Control(minimal) | control 13 |
| FANCA | QAFEScQLDSMVTAF | 1.782909 | Control(minimal) | control 13 |
| FANCA | QAFEScQLDSMVTAF | 1.782909 | Control(minimal) | control 13 |
| FANCA | QAFEScQLDSMVTAF | 1.782909 | Control(minimal) | control 13 |
| FANCA | QAFEScQLDSMVTAF | 1.782909 | Control(minimal) | control 13 |
| FANCA | QAFEScQLDSMVTAF | 1.782909 | Control(minimal) | control 13 |
| FANCA | QAFEScQLDSMVTAF | 1.782909 | Control(minimal) | control 13 |
| FAT4 | GVLIHIQESSNYTTVKI | 1.870384 | TBI(extreme) | TBI 2 |
| FAT4 | GVLIHIQESSNYTTVKI | 1.870384 | TBI(extreme) | TBI 2 |
| FAT4 | GVLIHIQESSNYTTVKI | 1.870384 | TBI(extreme) | TBI 2 |
| FAT4 | GVLIHIQESSNYTTVKI | 1.870384 | TBI(extreme) | TBI 2 |
| FAT4 | GVLIHIQESSNYTTVKI | 1.870384 | TBI(extreme) | TBI 2 |
| FAT4 | GVLIHIQESSNYTTVKI | 1.870384 | TBI(extreme) | TBI 2 |
| FAT4 | GVLIHIQESSNYTTVKI | 1.870384 | TBI(extreme) | TBI 2 |
| FAT4 | LLGPLDVHAGSyQIE | 1.789777 | TBI(extreme) | TBI 9 |
| FAT4 | LLGPLDVHAGSyQIE | 1.789777 | TBI(extreme) | TBI 9 |
| FAT4 | LLGPLDVHAGSyQIE | 1.789777 | TBI(extreme) | TBI 9 |
| FAT4 | LLGPLDVHAGsYQIE | 1.789777 | TBI(extreme) | TBI 9 |
| FAT4 | LLGPLDVHAGSyQIE | 1.789777 | TBI(extreme) | TBI 9 |
| FAT4 | LLGPLDVHAGsYQIE | 1.789777 | TBI(extreme) | TBI 9 |
| FAT4 | LLGPLDVHAGSyQIE | 1.789777 | TBI(extreme) | TBI 9 |
| FAT4 | LLGPLDVHAGsYQIE | 1.789777 | TBI(extreme) | TBI 9 |
| FAT4 | LLGPLDVHAGSyQIE | 1.789777 | TBI(extreme) | TBI 9 |
| FAT4 | LLGPLDVHAGsYQIE | 1.789777 | TBI(extreme) | TBI 9 |
| FAT4 | LLGPLDVHAGSyQIE | 1.789777 | TBI(extreme) | TBI 9 |
| FAT4 | LLGPLDVHAGsYQIE | 1.789777 | TBI(extreme) | TBI 9 |
| FAT4 | LLGPLDVHAGSyQIE | 1.789777 | TBI(extreme) | TBI 9 |
| FAT4 | LLGPLDVHAGsYQIE | 1.789777 | TBI(extreme) | TBI 9 |
| FAT4 | LLGPLDVHAGSyQIE | 1.789777 | TBI(extreme) | TBI 9 |
| FAT4 | LLGPLDVHAGsYQIE | 1.789777 | TBI(extreme) | TBI 9 |
| FAT4 | LLGPLDVHAGSyQIE | 1.789777 | TBI(extreme) | TBI 9 |
| FAT4 | VLQVAAADADEGtNADIR | 1.949267 | TBI(extreme) | TBI 10 |
| FAT4 | VLQVAAADADEGtNADIR | 1.949267 | TBI(extreme) | TBI 10 |
| FAT4 | VLQVAAADADEGtNADIR | 1.949267 | TBI(extreme) | TBI 10 |
| FAT4 | VLQVAAADADEGtNADIR | 1.949267 | TBI(extreme) | TBI 10 |
| FAT4 | VLQVAAADADEGtNADIR | 1.949267 | TBI(extreme) | TBI 10 |
| FAT4 | VLQVAAADADEGtNADIR | 1.949267 | TBI(extreme) | TBI 10 |
| FAT4 | VLQVAAADADEGtNADIR | 1.949267 | TBI(extreme) | TBI 10 |
| FAT4 | VLQVAAADADEGtNADIR | 1.949267 | TBI(extreme) | TBI 10 |
| FAT4 | VLQVAAADADEGtNADIR | 1.949267 | TBI(extreme) | TBI 10 |
| FAT4 | QcPRLEGACTRsPCQ | 1.850502 | Control(minimal) | control 6 |
| FAT4 | QcPRLEGACTRsPCQ | 1.850502 | Control(minimal) | control 6 |
| FAT4 | QcPRLEGACTRsPCQ | 1.850502 | Control(minimal) | control 6 |
| FAT4 | QcPRLEGACTRsPCQ | 1.850502 | Control(minimal) | control 6 |
| FAT4 | QcPRLEGACTRsPCQ | 1.850502 | Control(minimal) | control 6 |
| FAT4 | QcPRLEGACTRsPCQ | 1.850502 | Control(minimal) | control 6 |
| FAT4 | QcPRLEGACTRsPCQ | 1.850502 | Control(minimal) | control 6 |
| FAT4 | QcPRLEGACTRsPCQ | 1.850502 | Control(minimal) | control 6 |
| FAT4 | QcPRLEGACTRsPCQ | 1.850502 | Control(minimal) | control 6 |
| FAT4 | QcPRLEGACTRsPCQ | 1.850502 | Control(minimal) | control 6 |
| FAT4 | QcPRLEGACTRsPCQ | 1.850502 | Control(minimal) | control 6 |
| FAT4 | QcPRLEGACTRsPCQ | 1.850502 | Control(minimal) | control 6 |
| FAT4 | QcPRLEGACTRsPCQ | 1.850502 | Control(minimal) | control 6 |
| FBN1 | CPNGICENLRGTYKCIC | 2.036953 | Control(minimal) | control 7 |
| FBN1 | CPNGICENLRGTYKCIC | 2.036953 | Control(minimal) | control 7 |
| FBN1 | CPNGICENLRGTYKCIC | 2.036953 | Control(minimal) | control 7 |
| FBN1 | CPNGICENLRGTYKCIC | 2.036953 | Control(minimal) | control 7 |
| FBN1 | CPNGICENLRGTYKCIC | 2.036953 | Control(minimal) | control 7 |
| FBN1 | CPNGICENLRGTYKCIC | 2.036953 | Control(minimal) | control 7 |
| FBN1 | HCLcQKGyIGTH | 1.982241 | Control(minimal) | control 13 |
| FBN1 | HCLcQKGyIGTH | 1.982241 | Control(minimal) | control 13 |
| FBN1 | HCLcQKGyIGTH | 1.982241 | Control(minimal) | control 13 |
| FBN1 | HCLcQKGYIGtH | 1.982241 | Control(minimal) | control 13 |
| FBN1 | HCLcQKGyIGTH | 1.982241 | Control(minimal) | control 13 |
| FBN1 | HCLcQKGYIGtH | 1.982241 | Control(minimal) | control 13 |
| FBN1 | HCLcQKGyIGTH | 1.982241 | Control(minimal) | control 13 |
| FBN1 | HCLcQKGYIGtH | 1.982241 | Control(minimal) | control 13 |
| FBN1 | HCLcQKGyIGTH | 1.982241 | Control(minimal) | control 13 |
| FBN1 | HCLcQKGYIGtH | 1.982241 | Control(minimal) | control 13 |
| FBN1 | HCLcQKGyIGTH | 1.982241 | Control(minimal) | control 13 |
| FBN1 | HCLcQKGYIGtH | 1.982241 | Control(minimal) | control 13 |
| FBN1 | HCLcQKGyIGTH | 1.982241 | Control(minimal) | control 13 |
| FBN1 | HCLcQKGYIGtH | 1.982241 | Control(minimal) | control 13 |
| FBN1 | HCLcQKGyIGTH | 1.982241 | Control(minimal) | control 13 |
| FBN1 | HCLcQKGYIGtH | 1.982241 | Control(minimal) | control 13 |
| FBN1 | HCLcQKGyIGTH | 1.982241 | Control(minimal) | control 13 |
| FBN1 | HCLcQKGYIGtH | 1.982241 | Control(minimal) | control 13 |
| FBN1 | HCLcQKGyIGTH | 1.982241 | Control(minimal) | control 13 |
| FBN1 | HCLcQKGYIGtH | 1.982241 | Control(minimal) | control 13 |
| FBN1 | HCLcQKGyIGTH | 1.982241 | Control(minimal) | control 13 |
| FBN1 | HCLcQKGYIGtH | 1.982241 | Control(minimal) | control 13 |
| FBN1 | HCLcQKGyIGTH | 1.982241 | Control(minimal) | control 13 |
| FBN1 | HCLcQKGYIGtH | 1.982241 | Control(minimal) | control 13 |
| FBN1 | HCLcQKGyIGTH | 1.982241 | Control(minimal) | control 13 |
| FBN1 | HCLcQKGYIGtH | 1.89066 | Control(minimal) | control 13 |
| FBN1 | HCLcQKGYIGtH | 1.89066 | Control(minimal) | control 13 |
| FBN1 | HCLcQKGYIGtH | 1.89066 | Control(minimal) | control 13 |
| FBN1 | HCLcQKGYIGtH | 1.89066 | Control(minimal) | control 13 |
| FBN1 | HCLcQKGYIGtH | 1.89066 | Control(minimal) | control 13 |
| FBN1 | HCLcQKGYIGtH | 1.89066 | Control(minimal) | control 13 |
| FBN1 | HCLcQKGYIGtH | 1.89066 | Control(minimal) | control 13 |
| FBN1 | HCLcQKGYIGtH | 1.89066 | Control(minimal) | control 13 |
| FBN1 | HCLcQKGYIGtH | 1.89066 | Control(minimal) | control 13 |
| FBN1 | HCLcQKGYIGtH | 1.89066 | Control(minimal) | control 13 |
| FBN1 | HCLcQKGYIGtH | 1.89066 | Control(minimal) | control 13 |
| FBN1 | HCLcQKGYIGtH | 1.89066 | Control(minimal) | control 13 |
| FBN1 | HCLcQKGYIGtH | 1.89066 | Control(minimal) | control 13 |
| FBN2 | cVcPRGYVtSTDGSRC | 1.910144 | TBI(extreme) | TBI 5 |
| FBN2 | cVcPRGYVtSTDGSRC | 1.910144 | TBI(extreme) | TBI 5 |
| FBN2 | cVcPRGYVtSTDGSRC | 1.910144 | TBI(extreme) | TBI 5 |
| FBN2 | cVcPRGYVtSTDGSRC | 1.910144 | TBI(extreme) | TBI 5 |
| FBN2 | cVcPRGYVtSTDGSRC | 1.910144 | TBI(extreme) | TBI 5 |
| FBN2 | cVcPRGYVtSTDGSRC | 1.910144 | TBI(extreme) | TBI 5 |
| FBN2 | cVcPRGYVtSTDGSRC | 1.910144 | TBI(extreme) | TBI 5 |
| FBN2 | cVcPRGYVTsTDGSRC | 1.910144 | TBI(extreme) | TBI 5 |
| FBN2 | cVcPRGYVtSTDGSRC | 1.910144 | TBI(extreme) | TBI 5 |
| FBN2 | cVcPRGYVTsTDGSRC | 1.910144 | TBI(extreme) | TBI 5 |
| FBN2 | cVcPRGYVtSTDGSRC | 1.910144 | TBI(extreme) | TBI 5 |
| FBN2 | cVcPRGYVTsTDGSRC | 1.910144 | TBI(extreme) | TBI 5 |
| FBN2 | cVcPRGYVtSTDGSRC | 1.910144 | TBI(extreme) | TBI 5 |
| FBN2 | cVcPRGYVTsTDGSRC | 1.910144 | TBI(extreme) | TBI 5 |
| FBN2 | cVcPRGYVtSTDGSRC | 1.910144 | TBI(extreme) | TBI 5 |
| FBN2 | cVcPRGYVTsTDGSRC | 1.910144 | TBI(extreme) | TBI 5 |
| FBN2 | cVcPRGYVtSTDGSRC | 1.910144 | TBI(extreme) | TBI 5 |
| FBN2 | cVcPRGYVTsTDGSRC | 1.910144 | TBI(extreme) | TBI 5 |
| FBN2 | cVcPRGYVtSTDGSRC | 1.910144 | TBI(extreme) | TBI 5 |
| FBN2 | cVcPRGYVTsTDGSRC | 1.85095 | TBI(extreme) | TBI 5 |
| FBN2 | cVcPRGYVTsTDGSRC | 1.85095 | TBI(extreme) | TBI 5 |
| FBN2 | cVcPRGYVTsTDGSRC | 1.85095 | TBI(extreme) | TBI 5 |
| FBN2 | cVcPRGYVTsTDGSRC | 1.85095 | TBI(extreme) | TBI 5 |
| FBN2 | cVcPRGYVTsTDGSRC | 1.85095 | TBI(extreme) | TBI 5 |
| FBN2 | cVcPRGYVTsTDGSRC | 1.85095 | TBI(extreme) | TBI 5 |
| FBN2 | cVcPRGYVTsTDGSRC | 1.85095 | TBI(extreme) | TBI 5 |
| FBN2 | cEcQRGFSLDATG | 2.470417 | TBI(extreme) | TBI 6 |
| FBN2 | cEcQRGFSLDATG | 2.470417 | TBI(extreme) | TBI 6 |
| FBN2 | cEcQRGFSLDATG | 2.470417 | TBI(extreme) | TBI 6 |
| FBN2 | cEcQRGFSLDATG | 2.470417 | TBI(extreme) | TBI 6 |
| FBN2 | cEcQRGFSLDATG | 2.470417 | TBI(extreme) | TBI 6 |
| FBN2 | CQNILGGYRc | 2.470417 | TBI(extreme) | TBI 6 |
| FBN2 | CcATLGAAWGSPcERCEL | 2.11315 | Control(minimal) | control 9 |
| FBN2 | CcATLGAAWGSPcERCEL | 2.11315 | Control(minimal) | control 9 |
| FBN2 | CcATLGAAWGSPcERCEL | 2.11315 | Control(minimal) | control 9 |
| FBN2 | CcATLGAAWGSPcERCEL | 2.11315 | Control(minimal) | control 9 |
| FBN2 | CcATLGAAWGSPcERCEL | 2.11315 | Control(minimal) | control 9 |
| FBN2 | CcATLGAAWGSPcERCEL | 2.11315 | Control(minimal) | control 9 |
| FBN2 | CcATLGAAWGSPcERCEL | 2.11315 | Control(minimal) | control 9 |
| FBN2 | CcATLGAAWGSPcERCEL | 2.11315 | Control(minimal) | control 11 |
| FBN2 | CcATLGAAWGSPcERCEL | 2.11315 | Control(minimal) | control 11 |
| FBN2 | CcATLGAAWGSPcERCEL | 2.11315 | Control(minimal) | control 11 |
| FBN2 | CcATLGAAWGSPcERCEL | 2.11315 | Control(minimal) | control 11 |
| FBN2 | CcATLGAAWGSPcERCEL | 2.11315 | Control(minimal) | control 11 |
| FBN2 | CcATLGAAWGSPcERCEL | 2.11315 | Control(minimal) | control 11 |
| FBN2 | CcATLGAAWGSPcERCEL | 2.11315 | Control(minimal) | control 11 |
| FBN2 | GFGAcANtPRKRSSS | 1.788898 | Control(minimal) | control 13 |
| FBN2 | GFGAcANtPRKRSSS | 1.788898 | Control(minimal) | control 13 |
| FBN2 | GFGAcANtPRKRSSS | 1.788898 | Control(minimal) | control 13 |
| FBN3 | KSCQGcVRGVTASTRLA | 1.91715 | TBI(extreme) | TBI 1 |
| FBN3 | KSCQGcVRGVTASTRLA | 1.91715 | TBI(extreme) | TBI 1 |
| FBN3 | KScQGCVRGVTASTRLA | 1.91715 | TBI(extreme) | TBI 1 |
| FBN3 | KScQGCVRGVTASTRLA | 1.911337 | TBI(extreme) | TBI 1 |
| FBN3 | VcSHGDcMDtEGSYMC | 2.853752 | TBI(extreme) | TBI 46 |
| FBN3 | YRcEcNVGY | 2.853752 | TBI(extreme) | TBI 46 |
| FBN3 | YRcEcNVGY | 2.853752 | TBI(extreme) | TBI 46 |
| FBN3 | YRcEcNVGY | 2.853752 | TBI(extreme) | TBI 46 |
| FBN3 | RsEccATLGAAW | 2.449282 | Control(minimal) | control 4 |
| FBN3 | VLsDNGHRcFDTRQS | 2.449282 | Control(minimal) | control 4 |
| FBN3 | VLsDNGHRcFDTRQS | 2.449282 | Control(minimal) | control 4 |
| FBN3 | VLsDNGHRcFDTRQS | 2.449282 | Control(minimal) | control 4 |
| FBN3 | CcATLGAAWGSPcERCEI | 2.11315 | Control(minimal) | control 9 |
| FBN3 | CcATLGAAWGSPcERCEI | 2.11315 | Control(minimal) | control 9 |
| FBN3 | CcATLGAAWGSPcERCEI | 2.11315 | Control(minimal) | control 9 |
| FBN3 | CcATLGAAWGSPcERCEI | 2.11315 | Control(minimal) | control 9 |
| FBN3 | CcATLGAAWGSPcERCEI | 2.11315 | Control(minimal) | control 11 |
| FBN3 | CcATLGAAWGSPcERCEI | 2.11315 | Control(minimal) | control 11 |
| FBN3 | CcATLGAAWGSPcERCEI | 2.11315 | Control(minimal) | control 11 |
| FBN3 | CcATLGAAWGSPcERCEI | 2.11315 | Control(minimal) | control 11 |
| FBN3 | GAmCRDVDECADGQQD | 2.062801 | Control(minimal) | control 11 |
| FBN3 | GAmCRDVDECADGQQD | 2.062801 | Control(minimal) | control 11 |
| FBN3 | GAmCRDVDECADGQQD | 2.062801 | Control(minimal) | control 11 |
| FBN3 | GAmCRDVDECADGQQD | 2.062801 | Control(minimal) | control 11 |
| FBN3 | GAmCRDVDECADGQQD | 2.062801 | Control(minimal) | control 11 |
| FBN3 | GAmCRDVDECADGQQD | 2.062801 | Control(minimal) | control 11 |
| FCGBP | CsLGcSALSAPPQCQDGC | 1.736187 | TBI(extreme) | TBI 1 |
| FCGBP | CsLGcSALSAPPQCQDGC | 1.736187 | TBI(extreme) | TBI 1 |
| FCGBP | CsLGcSALSAPPQCQDGC | 1.736187 | TBI(extreme) | TBI 1 |
| FCGBP | CsLGcSALSAPPQCQDGC | 1.736187 | TBI(extreme) | TBI 1 |
| FCGBP | CsLGcSALSAPPQCQDGC | 1.736187 | TBI(extreme) | TBI 1 |
| FCGBP | QLRPPFRGRQcGLAGP | 2.134471 | TBI(extreme) | TBI 30 |
| FCGBP | QLRPPFRGRQcGLAGP | 2.134471 | TBI(extreme) | TBI 30 |
| FCGBP | sEGRLRVYQSGPRAV | 1.791541 | TBI(extreme) | TBI 30 |
| FCGBP | sEGRLRVYQSGPRAV | 1.791541 | TBI(extreme) | TBI 30 |
| FCGBP | sEGRLRVYQSGPRAV | 1.791541 | TBI(extreme) | TBI 30 |
| FCGBP | sEGRLRVYQSGPRAV | 1.791541 | TBI(extreme) | TBI 30 |
| FCGBP | sEGRLRVYQSGPRAV | 1.791541 | TBI(extreme) | TBI 30 |
| FCGBP | WCRCGPGGGSLVctPAS | 2.399524 | Control(minimal) | control 4 |
| FCGBP | WCRCGPGGGSLVctPAS | 2.399524 | Control(minimal) | control 4 |
| FCGBP | WCRCGPGGGSLVctPAS | 2.399524 | Control(minimal) | control 4 |
| FCGBP | WCRCGPGGGsLVcTPAS | 2.399524 | Control(minimal) | control 4 |
| FCGBP | WCRCGPGGGsLVcTPAS | 2.399524 | Control(minimal) | control 4 |
| FCGBP | WCRCGPGGGSLVctPAS | 2.399524 | Control(minimal) | control 4 |
| FCGBP | WCRCGPGGGsLVcTPAS | 2.399524 | Control(minimal) | control 4 |
| FCGBP | WCRCGPGGGsLVcTPAS | 2.232509 | Control(minimal) | control 4 |
| FCGBP | WCRCGPGGGsLVcTPAS | 2.232509 | Control(minimal) | control 4 |
| FCGBP | WCRCGPGGGsLVcTPAS | 2.232509 | Control(minimal) | control 4 |
| FCGBP | APLQcPDGcAEGcQcD | 1.877767 | Control(minimal) | control 10 |
| FCGBP | APLQcPDGcAEGcQcD | 1.877767 | Control(minimal) | control 10 |
| FCGBP | APLQcPDGcAEGcQcD | 1.877767 | Control(minimal) | control 10 |
| FCGBP | VcGLcGNYNGDPADDF | 1.868837 | Control(minimal) | control 12 |
| FCGBP | VcGLcGNYNGDPADDF | 1.868837 | Control(minimal) | control 12 |
| FCGBP | VcGLcGNYNGDPADDF | 1.868837 | Control(minimal) | control 12 |
| FCGBP | VcGLcGNYNGDPADDF | 1.868837 | Control(minimal) | control 12 |
| FCGBP | VcGLcGNYNGDPADDF | 1.868837 | Control(minimal) | control 12 |
| FCGBP | VcGLcGNYNGDPADDF | 1.868837 | Control(minimal) | control 12 |
| FCGBP | VcGLcGNYNGDPADDF | 1.868837 | Control(minimal) | control 12 |
| FCGBP | VcGLcGNYNGDPADDF | 1.868837 | Control(minimal) | control 12 |
| FCGBP | VcGLcGNYNGDPADDF | 1.868837 | Control(minimal) | control 12 |
| GPRC5C | FLFGVLFAIcFscL | 1.825425 | Control(minimal) | control 10 |
| GPRC5C | FLFGVLFAIcFscL | 1.825425 | Control(minimal) | control 10 |
| GPRC5C | FLFGVLFAIcFscL | 1.825425 | Control(minimal) | control 10 |
| GPRC5C | FLFGVLFAIcFscL | 1.825425 | Control(minimal) | control 10 |
| GPRC5C | FLFGVLFAIcFscL | 1.825425 | Control(minimal) | control 10 |
| GPRC5C | FLFGVLFAIcFscL | 1.825425 | Control(minimal) | control 10 |
| GPRC5C | FLFGVLFAIcFscL | 1.825425 | Control(minimal) | control 10 |
| GPRC5C | FLFGVLFAIcFscL | 1.825425 | Control(minimal) | control 10 |
| GPRC5C | FLFGVLFAIcFscL | 1.825425 | Control(minimal) | control 10 |
| GPRC5C | AIHKALVMcLGLPLFLF | 1.992994 | Control(minimal) | control 13 |
| GPRC5C | AIHKALVMcLGLPLFLF | 1.992994 | Control(minimal) | control 13 |
| GPRC5C | AIHKALVMcLGLPLFLF | 1.992994 | Control(minimal) | control 13 |
| GPRC5C | AIHKALVMcLGLPLFLF | 1.992994 | Control(minimal) | control 13 |
| GPRC5C | AIHKALVMcLGLPLFLF | 1.992994 | Control(minimal) | control 13 |
| GPRC5C | AIHKALVMcLGLPLFLF | 1.992994 | Control(minimal) | control 13 |
| GPRC5C | AIHKALVMcLGLPLFLF | 1.992994 | Control(minimal) | control 13 |
| GPRC5C | AIHKALVMcLGLPLFLF | 1.992994 | Control(minimal) | control 13 |
| GPRC5C | AIHKALVMcLGLPLFLF | 1.992994 | Control(minimal) | control 13 |
| GPRC5C | AIHKALVMcLGLPLFLF | 1.992994 | Control(minimal) | control 13 |
| GPRC5C | AIHKALVMcLGLPLFLF | 1.992994 | Control(minimal) | control 13 |
| GPRC5C | AIHKALVMcLGLPLFLF | 1.992994 | Control(minimal) | control 13 |
| GPRC5C | AIHKALVMcLGLPLFLF | 1.992994 | Control(minimal) | control 13 |
| GRM4 | LGYSmLLMVTCTVy | 1.954956 | TBI(extreme) | TBI 1 |
| GRM4 | LGYSmLLMVTCTVy | 1.954956 | TBI(extreme) | TBI 1 |
| GRM4 | LGYSmLLMVTCTVy | 1.954956 | TBI(extreme) | TBI 1 |
| GRM4 | LGYSmLLMVTCTVy | 1.954956 | TBI(extreme) | TBI 1 |
| GRM4 | LGYSmLLMVTCTVy | 1.954956 | TBI(extreme) | TBI 1 |
| GRM4 | LGYSmLLMVTCTVy | 1.954956 | TBI(extreme) | TBI 1 |
| GRM4 | LGYSmLLMVTCTVy | 1.954956 | TBI(extreme) | TBI 1 |
| GRM4 | LGYSmLLMVTCTVy | 1.954956 | TBI(extreme) | TBI 1 |
| GRM4 | LGYSmLLMVTCTVy | 1.954956 | TBI(extreme) | TBI 1 |
| GRM4 | LGYSmLLMVTCTVy | 1.954956 | TBI(extreme) | TBI 1 |
| GRM4 | LGYSmLLMVTCTVy | 1.954956 | TBI(extreme) | TBI 1 |
| GRM4 | LGYSmLLMVTCTVy | 1.954956 | TBI(extreme) | TBI 8 |
| GRM4 | LGYSmLLMVTCTVy | 1.954956 | TBI(extreme) | TBI 8 |
| GRM4 | LGYSmLLMVTCTVy | 1.954956 | TBI(extreme) | TBI 8 |
| GRM4 | LGYSmLLMVTCTVy | 1.954956 | TBI(extreme) | TBI 8 |
| GRM4 | LGYSmLLMVTCTVy | 1.954956 | TBI(extreme) | TBI 8 |
| GRM4 | LGYSmLLMVTCTVy | 1.954956 | TBI(extreme) | TBI 8 |
| GRM4 | LGYSmLLMVTCTVy | 1.954956 | TBI(extreme) | TBI 8 |
| GRM4 | LGYSmLLMVTCTVy | 1.954956 | TBI(extreme) | TBI 8 |
| GRM4 | LGYSmLLMVTCTVy | 1.954956 | TBI(extreme) | TBI 8 |
| GRM4 | LGYSmLLMVTCTVy | 1.954956 | TBI(extreme) | TBI 8 |
| GRM4 | LGYSmLLMVTCTVy | 1.954956 | TBI(extreme) | TBI 8 |
| GRM4 | DPVDGtQLLKYIRNV | 1.788902 | TBI(extreme) | TBI 30 |
| GRM4 | DPVDGtQLLKYIRNV | 1.788902 | TBI(extreme) | TBI 30 |
| GRM4 | DPVDGtQLLKYIRNV | 1.788902 | TBI(extreme) | TBI 30 |
| GRM4 | DPVDGtQLLKYIRNV | 1.788902 | TBI(extreme) | TBI 30 |
| GRM4 | DPVDGtQLLKYIRNV | 1.788902 | TBI(extreme) | TBI 30 |
| GRM4 | DPVDGtQLLKYIRNV | 1.788902 | TBI(extreme) | TBI 30 |
| GRM4 | DPVDGtQLLKYIRNV | 1.788902 | TBI(extreme) | TBI 30 |
| GRM4 | DPVDGtQLLKYIRNV | 1.788902 | TBI(extreme) | TBI 30 |
| GRM4 | DPVDGtQLLKYIRNV | 1.788902 | TBI(extreme) | TBI 30 |
| GRM4 | DPVDGtQLLKYIRNV | 1.788902 | TBI(extreme) | TBI 30 |
| GRM4 | DPVDGtQLLKYIRNV | 1.788902 | TBI(extreme) | TBI 30 |
| GRM4 | DPVDGtQLLKYIRNV | 1.788902 | TBI(extreme) | TBI 30 |
| GRM4 | DPVDGtQLLKYIRNV | 1.788902 | TBI(extreme) | TBI 30 |
| GRM4 | DPVDGtQLLKYIRNV | 1.788902 | TBI(extreme) | TBI 30 |
| GRM4 | DPVDGtQLLKYIRNV | 1.788902 | TBI(extreme) | TBI 30 |
| GRM4 | DPVDGtQLLKYIRNV | 1.788902 | TBI(extreme) | TBI 30 |
| GRM4 | DPVDGtQLLKYIRNV | 1.788902 | TBI(extreme) | TBI 30 |
| GRM4 | DPVDGtQLLKYIRNV | 1.788902 | TBI(extreme) | TBI 30 |
| GRM4 | DPVDGtQLLKYIRNV | 1.788902 | TBI(extreme) | TBI 30 |
| HECTD4 | LPAtGDGSAPVMA | 2.780243 | TBI(extreme) | TBI 1 |
| HECTD4 | LPAtGDGSAPVMA | 2.780243 | TBI(extreme) | TBI 1 |
| HECTD4 | LPAtGDGSAPVMA | 2.780243 | TBI(extreme) | TBI 1 |
| HECTD4 | PQsPSLLSKRKKVKmK | 2.780243 | TBI(extreme) | TBI 1 |
| HECTD4 | LPAtGDGSAPVMA | 2.780243 | TBI(extreme) | TBI 8 |
| HECTD4 | LPAtGDGSAPVMA | 2.780243 | TBI(extreme) | TBI 8 |
| HECTD4 | LPAtGDGSAPVMA | 2.780243 | TBI(extreme) | TBI 8 |
| HECTD4 | PQsPSLLSKRKKVKmK | 2.780243 | TBI(extreme) | TBI 8 |
| HECTD4 | ELLtAAVRVGGVTHLVGP | 1.973143 | TBI(extreme) | TBI 30 |
| HECTD4 | ELLtAAVRVGGVTHLVGP | 1.973143 | TBI(extreme) | TBI 30 |
| HECTD4 | ELLtAAVRVGGVTHLVGP | 1.973143 | TBI(extreme) | TBI 30 |
| HECTD4 | ELLtAAVRVGGVTHLVGP | 1.973143 | TBI(extreme) | TBI 30 |
| HECTD4 | ELLtAAVRVGGVTHLVGP | 1.973143 | TBI(extreme) | TBI 30 |
| HECTD4 | ELLtAAVRVGGVTHLVGP | 1.973143 | TBI(extreme) | TBI 30 |
| HECTD4 | ELLtAAVRVGGVTHLVGP | 1.973143 | TBI(extreme) | TBI 30 |
| HECTD4 | WIWSPASLNEKTPK | 1.745807 | Control(minimal) | control 9 |
| HECTD4 | WIWSPASLNEKTPK | 1.745807 | Control(minimal) | control 9 |
| HECTD4 | WIWSPASLNEKTPK | 1.745807 | Control(minimal) | control 9 |
| HECTD4 | VFKFFFsPQTE | 1.76327 | Control(minimal) | control 10 |
| HECTD4 | VFKFFFsPQTE | 1.76327 | Control(minimal) | control 10 |
| HECTD4 | VFKFFFsPQTE | 1.76327 | Control(minimal) | control 10 |
| HECTD4 | VFKFFFsPQTE | 1.76327 | Control(minimal) | control 10 |
| HECTD4 | VFKFFFsPQTE | 1.76327 | Control(minimal) | control 10 |
| HECTD4 | VFKFFFsPQTE | 1.76327 | Control(minimal) | control 10 |
| HECTD4 | VFKFFFsPQTE | 1.76327 | Control(minimal) | control 10 |
| HECTD4 | VFKFFFsPQTE | 1.76327 | Control(minimal) | control 10 |
| HECTD4 | VFKFFFsPQTE | 1.76327 | Control(minimal) | control 10 |
| HECTD4 | VFKFFFsPQTE | 1.76327 | Control(minimal) | control 10 |
| HECTD4 | VFKFFFsPQTE | 1.76327 | Control(minimal) | control 10 |
| HECTD4 | HVGAmCIHQLNLLAtN | 1.910931 | Control(minimal) | control 12 |
| HECTD4 | HVGAmCIHQLNLLAtN | 1.910931 | Control(minimal) | control 12 |
| HECTD4 | HVGAmCIHQLNLLAtN | 1.910931 | Control(minimal) | control 12 |
| HECTD4 | HVGAmCIHQLNLLAtN | 1.910931 | Control(minimal) | control 12 |
| HECTD4 | HVGAmCIHQLNLLAtN | 1.910931 | Control(minimal) | control 12 |
| HECTD4 | HVGAmCIHQLNLLAtN | 1.910931 | Control(minimal) | control 12 |
| HECTD4 | HVGAmCIHQLNLLAtN | 1.910931 | Control(minimal) | control 12 |
| HECTD4 | HVGAmCIHQLNLLAtN | 1.910931 | Control(minimal) | control 12 |
| HECTD4 | HVGAmCIHQLNLLAtN | 1.910931 | Control(minimal) | control 12 |
| HLA-DQB1 | FRNDQEETtGVVsTP | 1.812876 | TBI(extreme) | TBI 10 |
| HLA-DQB1 | FRNDQEETtGVVsTP | 1.812876 | TBI(extreme) | TBI 10 |
| HLA-DQB1 | FRNDQEETtGVVsTP | 1.812876 | TBI(extreme) | TBI 10 |
| HLA-DQB1 | FRNDQEETtGVVsTP | 1.812876 | TBI(extreme) | TBI 10 |
| HLA-DQB1 | FRNDQEETtGVVsTP | 1.812876 | TBI(extreme) | TBI 10 |
| HLA-DQB1 | FRNDQEETtGVVsTP | 1.812876 | TBI(extreme) | TBI 10 |
| HLA-DQB1 | FRNDQEETtGVVsTP | 1.812876 | TBI(extreme) | TBI 10 |
| HLA-DQB1 | FRNDQEETtGVVsTP | 1.812876 | TBI(extreme) | TBI 10 |
| HLA-DQB1 | FRNDQEETtGVVsTP | 1.812876 | TBI(extreme) | TBI 10 |
| HLA-DQB1 | FRNDQEETtGVVsTP | 1.812876 | TBI(extreme) | TBI 10 |
| HLA-DQB1 | FRNDQEETtGVVsTP | 1.812876 | TBI(extreme) | TBI 10 |
| HLA-DQB1 | FRNDQEETtGVVsTP | 1.812876 | TBI(extreme) | TBI 10 |
| HLA-DQB1 | FRNDQEETtGVVsTP | 1.812876 | TBI(extreme) | TBI 10 |
| HLA-DQB1 | FRNDQEETtGVVsTP | 1.812876 | TBI(extreme) | TBI 10 |
| HLA-DQB1 | FRNDQEETtGVVsTP | 1.812876 | TBI(extreme) | TBI 10 |
| HLA-DQB1 | FEPKSCDKtHtcPPC | 1.856292 | Control(minimal) | control 9 |
| HLA-DQB1 | FEPKSCDKtHtcPPC | 1.856292 | Control(minimal) | control 9 |
| HLA-DQB1 | FEPKSCDKtHtcPPC | 1.856292 | Control(minimal) | control 9 |
| HLA-DQB1 | RAVTLLGLPAAEyWNS | 2.14155 | Control(minimal) | control 13 |
| HLA-DQB1 | RAVTLLGLPAAEyWNS | 2.14155 | Control(minimal) | control 13 |
| HLA-DQB1 | RAVTLLGLPAAEyWNS | 2.14155 | Control(minimal) | control 13 |
| HLA-DQB1 | RAVTLLGLPAAEyWNS | 2.14155 | Control(minimal) | control 13 |
| HLA-DQB1 | RAVTLLGLPAAEyWNS | 2.14155 | Control(minimal) | control 13 |
| HLA-DQB1 | RAVTLLGLPAAEyWNS | 2.14155 | Control(minimal) | control 13 |
| HLA-DQB1 | RAVTLLGLPAAEyWNS | 2.14155 | Control(minimal) | control 13 |
| HLA-DQB1 | RAVTLLGLPAAEyWNS | 2.14155 | Control(minimal) | control 13 |
| HLA-DQB1 | RAVTLLGLPAAEyWNS | 2.14155 | Control(minimal) | control 13 |
| HMCN1 | GGRPCPGDtTQVTRCNV | 2.288169 | Control(minimal) | control 9 |
| HMCN1 | GGRPCPGDtTQVTRCNV | 2.288169 | Control(minimal) | control 9 |
| HMCN1 | GGRPCPGDtTQVTRCNV | 2.288169 | Control(minimal) | control 9 |
| HMCN1 | GGRPCPGDtTQVTRCNV | 2.288169 | Control(minimal) | control 9 |
| HMCN1 | GGRPCPGDtTQVTRCNV | 2.288169 | Control(minimal) | control 9 |
| HMCN1 | GGRPCPGDtTQVTRCNV | 2.288169 | Control(minimal) | control 9 |
| HMCN1 | GGRPCPGDtTQVTRCNV | 2.288169 | Control(minimal) | control 9 |
| HMCN1 | SILENGFLHIQsAHV | 1.787602 | Control(minimal) | control 13 |
| HMCN1 | SILENGFLHIQsAHV | 1.787602 | Control(minimal) | control 13 |
| HMCN1 | SILENGFLHIQsAHV | 1.787602 | Control(minimal) | control 13 |
| HMCN1 | SILENGFLHIQsAHV | 1.787602 | Control(minimal) | control 13 |
| HMCN1 | SILENGFLHIQsAHV | 1.787602 | Control(minimal) | control 13 |
| HMCN1 | SILENGFLHIQsAHV | 1.787602 | Control(minimal) | control 13 |
| HMCN1 | SILENGFLHIQsAHV | 1.787602 | Control(minimal) | control 13 |
| HMCN1 | SILENGFLHIQsAHV | 1.787602 | Control(minimal) | control 13 |
| HMCN1 | SILENGFLHIQsAHV | 1.787602 | Control(minimal) | control 13 |
| IGH | AVYYcARNGYcGGDcYS | 1.930753 | TBI(extreme) | TBI 1 |
| IGH | AVYYcARNGYcGGDcYS | 1.930753 | TBI(extreme) | TBI 1 |
| IGH | SEsGSGSLLPPIcPQMI | 1.902237 | TBI(extreme) | TBI 1 |
| IGH | SEsGSGSLLPPIcPQMI | 1.902237 | TBI(extreme) | TBI 1 |
| IGH | SESGsGSLLPPIcPQMI | 1.902237 | TBI(extreme) | TBI 1 |
| IGH | SESGsGSLLPPIcPQMI | 1.859094 | TBI(extreme) | TBI 1 |
| IGH | AVYYcARILTATWYSI | 1.85842 | TBI(extreme) | TBI 1 |
| IGH | AVYYcARILTATWYSI | 1.85842 | TBI(extreme) | TBI 1 |
| IGH | AVYYcARILTATWYSI | 1.85842 | TBI(extreme) | TBI 1 |
| IGH | YycARDPIADGGGDY | 1.73781 | TBI(extreme) | TBI 2 |
| IGH | YycARDPIADGGGDY | 1.73781 | TBI(extreme) | TBI 2 |
| IGH | YycARDPIADGGGDY | 1.73781 | TBI(extreme) | TBI 2 |
| IGH | YYVDsVKGRYS | 1.721747 | TBI(extreme) | TBI 5 |
| IGH | YYVDsVKGRYS | 1.721747 | TBI(extreme) | TBI 5 |
| IGH | YYVDsVKGRYS | 1.721747 | TBI(extreme) | TBI 5 |
| IGH | SEsGSGSLLPPIcPQMI | 1.902237 | TBI(extreme) | TBI 8 |
| IGH | SEsGSGSLLPPIcPQMI | 1.902237 | TBI(extreme) | TBI 8 |
| IGH | SESGsGSLLPPIcPQMI | 1.902237 | TBI(extreme) | TBI 8 |
| IGH | SESGsGSLLPPIcPQMI | 1.859094 | TBI(extreme) | TBI 8 |
| IGH | DTDcGGDCYSIWGQGT | 1.850751 | TBI(extreme) | TBI 9 |
| IGH | DTDcGGDCYSIWGQGT | 1.850751 | TBI(extreme) | TBI 9 |
| IGH | DTDcGGDCYSIWGQGT | 1.850751 | TBI(extreme) | TBI 9 |
| IGH | ASVKcVRDPLPtALGGAGV | 2.054145 | TBI(extreme) | TBI 10 |
| IGH | ASVKcVRDPLPtALGGAGV | 2.054145 | TBI(extreme) | TBI 10 |
| IGH | ASVKcVRDPLPtALGGAGV | 2.054145 | TBI(extreme) | TBI 10 |
| IGH | ASVKcVRDPLPtALGGAGV | 2.054145 | TBI(extreme) | TBI 10 |
| IGH | KPSGTVSLTcAVSGGsIS | 1.795928 | TBI(extreme) | TBI 10 |
| IGH | KPSGTVSLTcAVSGGsIS | 1.795928 | TBI(extreme) | TBI 10 |
| IGH | KPSGTVSLTcAVSGGsIS | 1.795928 | TBI(extreme) | TBI 10 |
| IGH | KPSGTVSLTcAVSGGsIS | 1.795928 | TBI(extreme) | TBI 10 |
| IGH | KPSGTVSLTcAVSGGsIS | 1.795928 | TBI(extreme) | TBI 10 |
| IGH | YYCARASYCGyDc | 1.958568 | TBI(extreme) | TBI 46 |
| IGH | YYCARASYCGyDc | 1.958568 | TBI(extreme) | TBI 46 |
| IGH | YYCARASyCGYDc | 1.958568 | TBI(extreme) | TBI 46 |
| IGH | YYCARASYCGyDc | 1.958568 | TBI(extreme) | TBI 46 |
| IGH | FCAKDPsGGRTtGGRAF | 2.006882 | TBI(extreme) | TBI 30 |
| IGH | FCAKDPsGGRTtGGRAF | 2.006882 | TBI(extreme) | TBI 30 |
| IGH | FCAKDPsGGRTtGGRAF | 2.006882 | TBI(extreme) | TBI 30 |
| IGH | tPPSLPVNPGEPASISCR | 1.916138 | Control(minimal) | control 9 |
| IGH | tPPSLPVNPGEPASISCR | 1.916138 | Control(minimal) | control 9 |
| IGH | ALYYCAREWGSGAyYP | 2.052197 | Control(minimal) | control 10 |
| IGH | ALYYCAREWGSGAyYP | 2.052197 | Control(minimal) | control 10 |
| IGH | tPPSLPVNPGEPASISCR | 1.916138 | Control(minimal) | control 11 |
| IGH | tPPSLPVNPGEPASISCR | 1.916138 | Control(minimal) | control 11 |
| IGH | TIsRDNVKNSLYLQ | 1.91387 | Control(minimal) | control 11 |
| IGH | TIsRDNVKNSLYLQ | 1.91387 | Control(minimal) | control 11 |
| IGH | TIsRDNVKNSLYLQ | 1.91387 | Control(minimal) | control 11 |
| IGK | TGFPSSSAQsISAHL | 1.869335 | TBI(extreme) | TBI 8 |
| IGK | TGFPSSSAQsISAHL | 1.869335 | TBI(extreme) | TBI 8 |
| IGK | TGFPSSSAQsISAHL | 1.869335 | TBI(extreme) | TBI 8 |
| IGK | TGFPSSSAQsISAHL | 1.869335 | TBI(extreme) | TBI 8 |
| IGK | TGFPSSSAQsISAHL | 1.869335 | TBI(extreme) | TBI 8 |
| IGK | VtKVAALIGNAQKLPm | 1.772421 | TBI(extreme) | TBI 8 |
| IGK | VtKVAALIGNAQKLPm | 1.772421 | TBI(extreme) | TBI 8 |
| IGK | VtKVAALIGNAQKLPm | 1.772421 | TBI(extreme) | TBI 8 |
| IGK | VtKVAALIGNAQKLPm | 1.772421 | TBI(extreme) | TBI 8 |
| IGK | VtKVAALIGNAQKLPm | 1.772421 | TBI(extreme) | TBI 8 |
| IGK | KEKVTITcRASQsIG | 1.752042 | TBI(extreme) | TBI 9 |
| IGK | KEKVTITcRASQsIG | 1.752042 | TBI(extreme) | TBI 9 |
| IGK | KEKVTITcRASQsIG | 1.752042 | TBI(extreme) | TBI 9 |
| IGK | KEKVTITcRASQsIG | 1.752042 | TBI(extreme) | TBI 9 |
| IGK | KEKVTITcRASQsIG | 1.752042 | TBI(extreme) | TBI 9 |
| IGK | KEKVTITcRASQsIG | 1.752042 | TBI(extreme) | TBI 9 |
| IGK | KEKVTITcRASQsIG | 1.752042 | TBI(extreme) | TBI 9 |
| IGK | KEKVTITcRASQsIG | 1.752042 | TBI(extreme) | TBI 9 |
| IGK | KEKVTITcRASQsIG | 1.752042 | TBI(extreme) | TBI 9 |
| IGK | StLSASVGDRVTIt | 1.741847 | TBI(extreme) | TBI 9 |
| IGK | StLSASVGDRVTIt | 1.741847 | TBI(extreme) | TBI 9 |
| IGK | StLSASVGDRVTIt | 1.741847 | TBI(extreme) | TBI 9 |
| IGK | StLSASVGDRVTIt | 1.741847 | TBI(extreme) | TBI 9 |
| IGK | StLSASVGDRVTIt | 1.741847 | TBI(extreme) | TBI 9 |
| IGK | QSPGTLSASPGERATLS | 2.027269 | TBI(extreme) | TBI 47 |
| IGK | QSPGTLSASPGERATLS | 2.027269 | TBI(extreme) | TBI 47 |
| IGK | QSPGTLSASPGERATLS | 2.027269 | TBI(extreme) | TBI 47 |
| IGK | YLASsRASGVPDRFSG | 2.087673 | Control(minimal) | control 8 |
| IGK | YLASsRASGVPDRFSG | 2.087673 | Control(minimal) | control 8 |
| IGK | YLASsRASGVPDRFSG | 2.087673 | Control(minimal) | control 8 |
| IGK | TcRASQsISTYLNWY | 1.88979 | Control(minimal) | control 8 |
| IGK | TcRASQsISTYLNWY | 1.88979 | Control(minimal) | control 8 |
| IGK | TcRASQsISTYLNWY | 1.88979 | Control(minimal) | control 8 |
| IGK | TcRASQsISTYLNWY | 1.88979 | Control(minimal) | control 8 |
| IGK | TcRASQsISTYLNWY | 1.88979 | Control(minimal) | control 8 |
| IGK | TcRASQsISTYLNWY | 1.88979 | Control(minimal) | control 8 |
| IGK | TcRASQsISTYLNWY | 1.88979 | Control(minimal) | control 8 |
| IGK | TcRASQsISTYLNWY | 1.88979 | Control(minimal) | control 8 |
| IGK | TcRASQsISTYLNWY | 1.88979 | Control(minimal) | control 8 |
| IGK | GARCDIRLTQSPPSLsA | 1.847596 | Control(minimal) | control 9 |
| IGK | GARCDIRLTQSPPSLsA | 1.847596 | Control(minimal) | control 9 |
| IGK | GARCDIRLTQSPPSLsA | 1.847596 | Control(minimal) | control 11 |
| IGK | GARCDIRLTQSPPSLsA | 1.847596 | Control(minimal) | control 11 |
| IGK | TDFTLTINRLEPEDF | 1.747523 | Control(minimal) | control 11 |
| IGK | TDFTLTINRLEPEDF | 1.747523 | Control(minimal) | control 11 |
| IGK | TDFTLTINRLEPEDF | 1.747523 | Control(minimal) | control 11 |
| IGK | TDFTLTINRLEPEDF | 1.747523 | Control(minimal) | control 11 |
| IGK | TDFTLTINRLEPEDF | 1.747523 | Control(minimal) | control 11 |
| IGL | SSLsAVVFGGGSKVTVLG | 1.74487 | TBI(extreme) | TBI 2 |
| IGL | SSLsAVVFGGGSKVTVLG | 1.74487 | TBI(extreme) | TBI 2 |
| IGL | SSLsAVVFGGGSKVTVLG | 1.74487 | TBI(extreme) | TBI 2 |
| IGL | GLQSEDEADYYCtS | 1.711334 | TBI(extreme) | TBI 6 |
| IGL | GLQSEDEADYYCtS | 1.711334 | TBI(extreme) | TBI 6 |
| IGL | GLQSEDEADYYCtS | 1.711334 | TBI(extreme) | TBI 6 |
| IGL | GLQSEDEADYYCTs | 1.711334 | TBI(extreme) | TBI 6 |
| IGL | GLQSEDEADYYCtS | 1.711334 | TBI(extreme) | TBI 6 |
| IGL | IATLScRASQSVSssY | 1.90429 | TBI(extreme) | TBI 10 |
| IGL | IATLScRASQSVSssY | 1.90429 | TBI(extreme) | TBI 10 |
| IGL | IATLScRASQSVSssY | 1.90429 | TBI(extreme) | TBI 10 |
| IGL | IATLScRASQsVSSsY | 1.90429 | TBI(extreme) | TBI 10 |
| IGL | IATLScRASQSVSssY | 1.90429 | TBI(extreme) | TBI 10 |
| IGL | IATLScRASQSVSsSy | 1.90429 | TBI(extreme) | TBI 10 |
| IGL | IATLScRASQSVSssY | 1.90429 | TBI(extreme) | TBI 10 |
| IGL | IATLScRASQsVSSsY | 1.804754 | TBI(extreme) | TBI 10 |
| IGL | IATLScRASQsVSSsY | 1.804754 | TBI(extreme) | TBI 10 |
| IGL | IATLScRASQsVSSsY | 1.804754 | TBI(extreme) | TBI 10 |
| IGL | IATLScRASQsVSSsY | 1.804754 | TBI(extreme) | TBI 10 |
| IGL | IATLScRASQSVSsSy | 1.792078 | TBI(extreme) | TBI 10 |
| IGL | IATLScRASQSVSsSy | 1.792078 | TBI(extreme) | TBI 10 |
| IGL | IATLScRASQSVSsSy | 1.792078 | TBI(extreme) | TBI 10 |
| IGL | IATLScRASQSVSsSy | 1.792078 | TBI(extreme) | TBI 10 |
| IGL | FLTLITHCAGSWAQs | 1.766473 | TBI(extreme) | TBI 47 |
| IGL | FLTLITHCAGSWAQs | 1.766473 | TBI(extreme) | TBI 47 |
| IGL | FLTLITHCAGSWAQs | 1.766473 | TBI(extreme) | TBI 47 |
| IGL | QFVmtQSPAtLSsSP | 1.95425 | Control(minimal) | control 7 |
| IGL | QFVmtQSPAtLSsSP | 1.95425 | Control(minimal) | control 7 |
| IGL | QFVmtQSPATLssSP | 1.95425 | Control(minimal) | control 7 |
| IGL | QFVmtQSPATLSssP | 1.95425 | Control(minimal) | control 7 |
| IGL | QFVmtQSPAtLSsSP | 1.95425 | Control(minimal) | control 7 |
| IGL | QFVmtQSPAtLSSsP | 1.95425 | Control(minimal) | control 7 |
| IGL | QFVmtQSPAtLSsSP | 1.95425 | Control(minimal) | control 7 |
| IGL | QFVmtQSPATLsSsP | 1.95425 | Control(minimal) | control 7 |
| IGL | QFVmtQSPAtLSsSP | 1.95425 | Control(minimal) | control 7 |
| IGL | QFVmtQSPAtLsSSP | 1.95425 | Control(minimal) | control 7 |
| IGL | QFVmtQSPAtLSsSP | 1.95425 | Control(minimal) | control 7 |
| IGL | QFVmtQSPATLssSP | 1.952227 | Control(minimal) | control 7 |
| IGL | QFVmtQSPATLssSP | 1.952227 | Control(minimal) | control 7 |
| IGL | QFVmtQSPATLssSP | 1.952227 | Control(minimal) | control 7 |
| IGL | QFVmtQSPATLssSP | 1.952227 | Control(minimal) | control 7 |
| IGL | QFVmtQSPATLssSP | 1.952227 | Control(minimal) | control 7 |
| IGL | QFVmtQSPATLSssP | 1.912452 | Control(minimal) | control 7 |
| IGL | QFVmtQSPATLSssP | 1.912452 | Control(minimal) | control 7 |
| IGL | QFVmtQSPATLSssP | 1.912452 | Control(minimal) | control 7 |
| IGL | QFVmtQSPATLSssP | 1.912452 | Control(minimal) | control 7 |
| IGL | QFVmtQSPATLSssP | 1.912452 | Control(minimal) | control 7 |
| IGL | QFVmtQSPAtLSSsP | 1.844964 | Control(minimal) | control 7 |
| IGL | QFVmtQSPAtLSSsP | 1.844964 | Control(minimal) | control 7 |
| IGL | QFVmtQSPAtLSSsP | 1.844964 | Control(minimal) | control 7 |
| IGL | QFVmtQSPAtLSSsP | 1.844964 | Control(minimal) | control 7 |
| IGL | QFVmtQSPAtLSSsP | 1.844964 | Control(minimal) | control 7 |
| IGL | QFVmtQSPATLsSsP | 1.842941 | Control(minimal) | control 7 |
| IGL | QFVmtQSPATLsSsP | 1.842941 | Control(minimal) | control 7 |
| IGL | QFVmtQSPATLsSsP | 1.842941 | Control(minimal) | control 7 |
| IGL | QFVmtQSPATLsSsP | 1.842941 | Control(minimal) | control 7 |
| IGL | QFVmtQSPATLsSsP | 1.842941 | Control(minimal) | control 7 |
| IGL | QFVmtQSPAtLsSSP | 1.826692 | Control(minimal) | control 7 |
| IGL | QFVmtQSPAtLsSSP | 1.826692 | Control(minimal) | control 7 |
| IGL | QFVmtQSPAtLsSSP | 1.826692 | Control(minimal) | control 7 |
| IGL | QFVmtQSPAtLsSSP | 1.826692 | Control(minimal) | control 7 |
| IGL | QFVmtQSPAtLsSSP | 1.826692 | Control(minimal) | control 7 |
| IGL | GVyYcMQGTHWPR | 1.883699 | Control(minimal) | control 10 |
| IGL | GVyYcMQGTHWPR | 1.883699 | Control(minimal) | control 10 |
| IGL | GVyYcMQGTHWPR | 1.883699 | Control(minimal) | control 10 |
| ITGA8 | NSDGLDDVLVGAPLFmE | 1.80216 | TBI(extreme) | TBI 1 |
| ITGA8 | NSDGLDDVLVGAPLFmE | 1.80216 | TBI(extreme) | TBI 1 |
| ITGA8 | NSDGLDDVLVGAPLFmE | 1.80216 | TBI(extreme) | TBI 1 |
| ITGA8 | NSDGLDDVLVGAPLFmE | 1.80216 | TBI(extreme) | TBI 1 |
| ITGA8 | NSDGLDDVLVGAPLFmE | 1.80216 | TBI(extreme) | TBI 1 |
| ITGA8 | NSDGLDDVLVGAPLFmE | 1.80216 | TBI(extreme) | TBI 1 |
| ITGA8 | NSDGLDDVLVGAPLFmE | 1.80216 | TBI(extreme) | TBI 1 |
| ITGA8 | NSDGLDDVLVGAPLFmE | 1.80216 | TBI(extreme) | TBI 8 |
| ITGA8 | NSDGLDDVLVGAPLFmE | 1.80216 | TBI(extreme) | TBI 8 |
| ITGA8 | NSDGLDDVLVGAPLFmE | 1.80216 | TBI(extreme) | TBI 8 |
| ITGA8 | NSDGLDDVLVGAPLFmE | 1.80216 | TBI(extreme) | TBI 8 |
| ITGA8 | NSDGLDDVLVGAPLFmE | 1.80216 | TBI(extreme) | TBI 8 |
| ITGA8 | NSDGLDDVLVGAPLFmE | 1.80216 | TBI(extreme) | TBI 8 |
| ITGA8 | NSDGLDDVLVGAPLFmE | 1.80216 | TBI(extreme) | TBI 8 |
| ITGA8 | NLPsPLLSPLCcCPAA | 1.780936 | TBI(extreme) | TBI 10 |
| ITGA8 | NLPsPLLSPLCcCPAA | 1.780936 | TBI(extreme) | TBI 10 |
| ITGA8 | NLPsPLLSPLCcCPAA | 1.780936 | TBI(extreme) | TBI 10 |
| ITGA8 | NLPsPLLSPLCCcPAA | 1.780936 | TBI(extreme) | TBI 10 |
| ITGA8 | NLPsPLLSPLCcCPAA | 1.780936 | TBI(extreme) | TBI 10 |
| ITGA8 | NLPsPLLSPLCCcPAA | 1.753223 | TBI(extreme) | TBI 10 |
| ITGA8 | NLPsPLLSPLCCcPAA | 1.753223 | TBI(extreme) | TBI 10 |
| ITGA8 | NLPsPLLSPLCCcPAA | 1.753223 | TBI(extreme) | TBI 10 |
| KCNMB3 | GtPFScFYSPASQSE | 3.190473 | Control(minimal) | control 9 |
| KCNMB3 | LDCAFTcGVHcHGQGKy | 3.190473 | Control(minimal) | control 9 |
| KCNMB3 | LDCAFTcGVHcHGQGKy | 3.190473 | Control(minimal) | control 9 |
| KCNMB3 | LDCAFTcGVHcHGQGKy | 3.190473 | Control(minimal) | control 9 |
| KCNMB3 | LDCAFTcGVHcHGQGKy | 3.190473 | Control(minimal) | control 9 |
| KCNMB3 | LDCAFTcGVHcHGQGKy | 3.190473 | Control(minimal) | control 9 |
| KCNMB3 | LDCAFTcGVHcHGQGKy | 3.190473 | Control(minimal) | control 9 |
| KCNMB3 | LDCAFTcGVHcHGQGKy | 3.190473 | Control(minimal) | control 9 |
| KCNMB3 | GtPFScFYSPASQSE | 1.771665 | Control(minimal) | control 9 |
| KCNMB3 | GtPFScFYSPASQSE | 1.771665 | Control(minimal) | control 9 |
| KCNMB3 | GtPFScFYSPASQSE | 1.771665 | Control(minimal) | control 9 |
| KCNMB3 | GtPFScFYSPASQSE | 1.771665 | Control(minimal) | control 9 |
| KCNMB3 | GtPFScFYSPASQSE | 1.771665 | Control(minimal) | control 9 |
| KCNMB3 | GtPFScFYSPASQSE | 1.771665 | Control(minimal) | control 9 |
| KCNMB3 | GtPFScFYSPASQSE | 1.771665 | Control(minimal) | control 9 |
| KCNMB3 | GtPFScFYSPASQSE | 1.771665 | Control(minimal) | control 9 |
| KCNMB3 | GtPFScFYSPASQSE | 1.771665 | Control(minimal) | control 9 |
| KCNMB3 | GtPFScFYSPASQSE | 1.771665 | Control(minimal) | control 9 |
| KCNMB3 | GtPFScFYSPASQSE | 1.771665 | Control(minimal) | control 9 |
| KCNMB3 | GtPFScFYSPASQSE | 1.771665 | Control(minimal) | control 9 |
| KCNMB3 | LDCAFTcGVHcHGQGKy | 1.771665 | Control(minimal) | control 9 |
| KCNMB3 | GtPFScFYSPASQSE | 3.190473 | Control(minimal) | control 11 |
| KCNMB3 | LDCAFTcGVHcHGQGKy | 3.190473 | Control(minimal) | control 11 |
| KCNMB3 | LDCAFTcGVHcHGQGKy | 3.190473 | Control(minimal) | control 11 |
| KCNMB3 | LDCAFTcGVHcHGQGKy | 3.190473 | Control(minimal) | control 11 |
| KCNMB3 | LDCAFTcGVHcHGQGKy | 3.190473 | Control(minimal) | control 11 |
| KCNMB3 | LDCAFTcGVHcHGQGKy | 3.190473 | Control(minimal) | control 11 |
| KCNMB3 | LDCAFTcGVHcHGQGKy | 3.190473 | Control(minimal) | control 11 |
| KCNMB3 | LDCAFTcGVHcHGQGKy | 3.190473 | Control(minimal) | control 11 |
| KCNMB3 | GtPFScFYSPASQSE | 1.771665 | Control(minimal) | control 11 |
| KCNMB3 | GtPFScFYSPASQSE | 1.771665 | Control(minimal) | control 11 |
| KCNMB3 | GtPFScFYSPASQSE | 1.771665 | Control(minimal) | control 11 |
| KCNMB3 | GtPFScFYSPASQSE | 1.771665 | Control(minimal) | control 11 |
| KCNMB3 | GtPFScFYSPASQSE | 1.771665 | Control(minimal) | control 11 |
| KCNMB3 | GtPFScFYSPASQSE | 1.771665 | Control(minimal) | control 11 |
| KCNMB3 | GtPFScFYSPASQSE | 1.771665 | Control(minimal) | control 11 |
| KCNMB3 | GtPFScFYSPASQSE | 1.771665 | Control(minimal) | control 11 |
| KCNMB3 | GtPFScFYSPASQSE | 1.771665 | Control(minimal) | control 11 |
| KCNMB3 | GtPFScFYSPASQSE | 1.771665 | Control(minimal) | control 11 |
| KCNMB3 | GtPFScFYSPASQSE | 1.771665 | Control(minimal) | control 11 |
| KCNMB3 | GtPFScFYSPASQSE | 1.771665 | Control(minimal) | control 11 |
| KCNMB3 | LDCAFTcGVHcHGQGKy | 1.771665 | Control(minimal) | control 11 |
| KLHL8 | STVAPmNtPRGGVGSVAL | 1.834733 | TBI(extreme) | TBI 1 |
| KLHL8 | STVAPmNtPRGGVGSVAL | 1.834733 | TBI(extreme) | TBI 1 |
| KLHL8 | STVAPmNtPRGGVGSVAL | 1.834733 | TBI(extreme) | TBI 1 |
| KLHL8 | STVAPmNtPRGGVGSVAL | 1.834733 | TBI(extreme) | TBI 1 |
| KLHL8 | STVAPmNtPRGGVGSVAL | 1.834733 | TBI(extreme) | TBI 1 |
| KLHL8 | STVAPmNtPRGGVGSVAL | 1.834733 | TBI(extreme) | TBI 1 |
| KLHL8 | STVAPmNtPRGGVGSVAL | 1.834733 | TBI(extreme) | TBI 1 |
| KLHL8 | STVAPmNtPRGGVGSVAL | 1.834733 | TBI(extreme) | TBI 1 |
| KLHL8 | STVAPmNtPRGGVGSVAL | 1.834733 | TBI(extreme) | TBI 1 |
| KLHL8 | STVAPmNtPRGGVGSVAL | 1.834733 | TBI(extreme) | TBI 1 |
| KLHL8 | STVAPmNtPRGGVGSVAL | 1.834733 | TBI(extreme) | TBI 1 |
| KLHL8 | STVAPmNtPRGGVGSVAL | 1.834733 | TBI(extreme) | TBI 1 |
| KLHL8 | STVAPmNtPRGGVGSVAL | 1.834733 | TBI(extreme) | TBI 1 |
| KLHL8 | STVAPmNtPRGGVGSVAL | 1.834733 | TBI(extreme) | TBI 1 |
| KLHL8 | STVAPmNtPRGGVGSVAL | 1.834733 | TBI(extreme) | TBI 1 |
| KLHL8 | STVAPmNtPRGGVGSVAL | 1.834733 | TBI(extreme) | TBI 8 |
| KLHL8 | STVAPmNtPRGGVGSVAL | 1.834733 | TBI(extreme) | TBI 8 |
| KLHL8 | STVAPmNtPRGGVGSVAL | 1.834733 | TBI(extreme) | TBI 8 |
| KLHL8 | STVAPmNtPRGGVGSVAL | 1.834733 | TBI(extreme) | TBI 8 |
| KLHL8 | STVAPmNtPRGGVGSVAL | 1.834733 | TBI(extreme) | TBI 8 |
| KLHL8 | STVAPmNtPRGGVGSVAL | 1.834733 | TBI(extreme) | TBI 8 |
| KLHL8 | STVAPmNtPRGGVGSVAL | 1.834733 | TBI(extreme) | TBI 8 |
| KLHL8 | STVAPmNtPRGGVGSVAL | 1.834733 | TBI(extreme) | TBI 8 |
| KLHL8 | STVAPmNtPRGGVGSVAL | 1.834733 | TBI(extreme) | TBI 8 |
| KLHL8 | STVAPmNtPRGGVGSVAL | 1.834733 | TBI(extreme) | TBI 8 |
| KLHL8 | STVAPmNtPRGGVGSVAL | 1.834733 | TBI(extreme) | TBI 8 |
| KLHL8 | STVAPmNtPRGGVGSVAL | 1.834733 | TBI(extreme) | TBI 8 |
| KLHL8 | STVAPmNtPRGGVGSVAL | 1.834733 | TBI(extreme) | TBI 8 |
| KLHL8 | STVAPmNtPRGGVGSVAL | 1.834733 | TBI(extreme) | TBI 8 |
| KLHL8 | STVAPmNtPRGGVGSVAL | 1.834733 | TBI(extreme) | TBI 8 |
| LAMA1 | DIsVGGmcICYGHASSc | 1.799906 | TBI(extreme) | TBI 9 |
| LAMA1 | DIsVGGmcICYGHASSc | 1.799906 | TBI(extreme) | TBI 9 |
| LAMA1 | DIsVGGmcICYGHASSc | 1.799906 | TBI(extreme) | TBI 9 |
| LAMA1 | DIsVGGmcICYGHASSc | 1.799906 | TBI(extreme) | TBI 9 |
| LAMA1 | DIsVGGmcICYGHASSc | 1.799906 | TBI(extreme) | TBI 9 |
| LAMA1 | DIsVGGmcICYGHASSc | 1.799906 | TBI(extreme) | TBI 9 |
| LAMA1 | DIsVGGmcICYGHASSc | 1.799906 | TBI(extreme) | TBI 9 |
| LAMA1 | DIsVGGmcICYGHASSc | 1.799906 | TBI(extreme) | TBI 9 |
| LAMA1 | DIsVGGmcICYGHASSc | 1.799906 | TBI(extreme) | TBI 9 |
| LAMA1 | DIsVGGmcICYGHASSc | 1.799906 | TBI(extreme) | TBI 9 |
| LAMA1 | DIsVGGmcICYGHASSc | 1.799906 | TBI(extreme) | TBI 9 |
| LAMA1 | ITAAYEPKtATVLcDG | 1.888005 | TBI(extreme) | TBI 30 |
| LAMA1 | GINcETcIDGYYRPHK | 1.737844 | TBI(extreme) | TBI 30 |
| LAMA1 | GINcETcIDGYYRPHK | 1.737844 | TBI(extreme) | TBI 30 |
| LAMA1 | GINcETcIDGYYRPHK | 1.737844 | TBI(extreme) | TBI 30 |
| LAMA1 | GINcETcIDGYYRPHK | 1.737844 | TBI(extreme) | TBI 30 |
| LAMA1 | GINcETcIDGYYRPHK | 1.737844 | TBI(extreme) | TBI 30 |
| LAMA1 | GINcETcIDGYYRPHK | 1.737844 | TBI(extreme) | TBI 30 |
| LAMA1 | GINcETcIDGYYRPHK | 1.737844 | TBI(extreme) | TBI 30 |
| LAMA1 | CRPcNcDPVGS | 2.414484 | Control(minimal) | control 3 |
| LAMA1 | CRPcNcDPVGS | 2.414484 | Control(minimal) | control 3 |
| LAMA1 | CRPcNcDPVGS | 2.414484 | Control(minimal) | control 3 |
| LAMA1 | CRPcNcDPVGS | 2.414484 | Control(minimal) | control 3 |
| LAMA1 | CRPcNcDPVGS | 2.414484 | Control(minimal) | control 3 |
| LAMA1 | GKcLNcGDNTAGD | 2.414484 | Control(minimal) | control 3 |
| LAMA5 | CNcSGPGIQELtDPTc | 1.802958 | TBI(extreme) | TBI 8 |
| LAMA5 | CNcSGPGIQELtDPTc | 1.802958 | TBI(extreme) | TBI 8 |
| LAMA5 | CNcSGPGIQELtDPTc | 1.802958 | TBI(extreme) | TBI 8 |
| LAMA5 | CNcSGPGIQELtDPTc | 1.802958 | TBI(extreme) | TBI 8 |
| LAMA5 | CNcSGPGIQELtDPTc | 1.802958 | TBI(extreme) | TBI 8 |
| LAMA5 | CNcSGPGIQELtDPTc | 1.802958 | TBI(extreme) | TBI 8 |
| LAMA5 | CNcSGPGIQELtDPTc | 1.802958 | TBI(extreme) | TBI 8 |
| LAMA5 | CNcSGPGIQELtDPTc | 1.802958 | TBI(extreme) | TBI 8 |
| LAMA5 | CNcSGPGIQELtDPTc | 1.802958 | TBI(extreme) | TBI 8 |
| LAMA5 | CQPcDcSGNGD | 2.945776 | TBI(extreme) | TBI 30 |
| LAMA5 | CQPcDcSGNGD | 2.945776 | TBI(extreme) | TBI 30 |
| LAMA5 | CQPcDcSGNGD | 2.945776 | TBI(extreme) | TBI 30 |
| LAMA5 | CQPcDcSGNGD | 2.945776 | TBI(extreme) | TBI 30 |
| LAMA5 | CQPcDcSGNGD | 2.945776 | TBI(extreme) | TBI 30 |
| LAMA5 | CQPcDcSGNGD | 2.945776 | TBI(extreme) | TBI 30 |
| LAMA5 | CQPcDcSGNGD | 2.945776 | TBI(extreme) | TBI 30 |
| LAMA5 | TEACDPHSGHcLCKAGVTG | 2.945776 | TBI(extreme) | TBI 30 |
| LAMA5 | TEACDPHSGHcLCKAGVTG | 1.760853 | TBI(extreme) | TBI 30 |
| LAMA5 | TEACDPHSGHcLCKAGVTG | 1.760853 | TBI(extreme) | TBI 30 |
| LAMA5 | TEACDPHSGHcLCKAGVTG | 1.760853 | TBI(extreme) | TBI 30 |
| LAMA5 | TEACDPHSGHcLCKAGVTG | 1.760853 | TBI(extreme) | TBI 30 |
| LAMA5 | TEACDPHSGHcLCKAGVTG | 1.760853 | TBI(extreme) | TBI 30 |
| LAMA5 | TEACDPHSGHcLCKAGVTG | 1.760853 | TBI(extreme) | TBI 30 |
| LAMA5 | TEACDPHSGHcLCKAGVTG | 1.760853 | TBI(extreme) | TBI 30 |
| LAMA5 | TEACDPHSGHcLCKAGVTG | 1.760853 | TBI(extreme) | TBI 30 |
| LAMA5 | TEACDPHSGHcLCKAGVTG | 1.760853 | TBI(extreme) | TBI 30 |
| LAMA5 | LLANsTALEEAmLQE | 1.731907 | TBI(extreme) | TBI 47 |
| LAMA5 | LLANsTALEEAmLQE | 1.731907 | TBI(extreme) | TBI 47 |
| LAMA5 | LLANsTALEEAmLQE | 1.731907 | TBI(extreme) | TBI 47 |
| LAMA5 | LLANStALEEAmLQE | 1.731907 | TBI(extreme) | TBI 47 |
| LAMA5 | LLANsTALEEAmLQE | 1.731907 | TBI(extreme) | TBI 47 |
| LAMA5 | LLANStALEEAmLQE | 1.731907 | TBI(extreme) | TBI 47 |
| LAMA5 | LLANsTALEEAmLQE | 1.731907 | TBI(extreme) | TBI 47 |
| LAMA5 | LLANStALEEAmLQE | 1.731907 | TBI(extreme) | TBI 47 |
| LAMA5 | LLANsTALEEAmLQE | 1.731907 | TBI(extreme) | TBI 47 |
| LAMA5 | LLANStALEEAmLQE | 1.731907 | TBI(extreme) | TBI 47 |
| LAMA5 | LLANsTALEEAmLQE | 1.731907 | TBI(extreme) | TBI 47 |
| LAMA5 | LLANStALEEAmLQE | 1.731907 | TBI(extreme) | TBI 47 |
| LAMA5 | LLANsTALEEAmLQE | 1.731907 | TBI(extreme) | TBI 47 |
| LAMA5 | LLANStALEEAmLQE | 1.731907 | TBI(extreme) | TBI 47 |
| LAMA5 | LLANsTALEEAmLQE | 1.731907 | TBI(extreme) | TBI 47 |
| LAMA5 | LLANStALEEAmLQE | 1.731907 | TBI(extreme) | TBI 47 |
| LAMA5 | LLANsTALEEAmLQE | 1.731907 | TBI(extreme) | TBI 47 |
| LAMA5 | LLANStALEEAmLQE | 1.701687 | TBI(extreme) | TBI 47 |
| LAMA5 | LLANStALEEAmLQE | 1.701687 | TBI(extreme) | TBI 47 |
| LAMA5 | LLANStALEEAmLQE | 1.701687 | TBI(extreme) | TBI 47 |
| LAMA5 | LLANStALEEAmLQE | 1.701687 | TBI(extreme) | TBI 47 |
| LAMA5 | LLANStALEEAmLQE | 1.701687 | TBI(extreme) | TBI 47 |
| LAMA5 | LLANStALEEAmLQE | 1.701687 | TBI(extreme) | TBI 47 |
| LAMA5 | LLANStALEEAmLQE | 1.701687 | TBI(extreme) | TBI 47 |
| LAMA5 | LLANStALEEAmLQE | 1.701687 | TBI(extreme) | TBI 47 |
| LAMA5 | LLANStALEEAmLQE | 1.701687 | TBI(extreme) | TBI 47 |
| LAMA5 | VAMtRSVEVHGAVGASGCP | 1.81767 | Control(minimal) | control 4 |
| LAMA5 | VAMtRSVEVHGAVGASGCP | 1.81767 | Control(minimal) | control 4 |
| LAMA5 | VAMtRSVEVHGAVGASGCP | 1.81767 | Control(minimal) | control 4 |
| LAMA5 | VAMtRSVEVHGAVGASGCP | 1.81767 | Control(minimal) | control 4 |
| LAMA5 | VAMtRSVEVHGAVGASGCP | 1.81767 | Control(minimal) | control 4 |
| LAMA5 | cSPGFHGFPscVPCHcS | 1.763615 | Control(minimal) | control 10 |
| LAMA5 | cSPGFHGFPscVPCHcS | 1.763615 | Control(minimal) | control 10 |
| LAMA5 | cSPGFHGFPscVPCHcS | 1.763615 | Control(minimal) | control 10 |
| LAMA5 | cSPGFHGFPscVPCHcS | 1.763615 | Control(minimal) | control 10 |
| LAMA5 | cSPGFHGFPscVPCHcS | 1.763615 | Control(minimal) | control 10 |
| LAMA5 | cSPGFHGFPscVPCHcS | 1.763615 | Control(minimal) | control 10 |
| LAMA5 | cSPGFHGFPscVPCHcS | 1.763615 | Control(minimal) | control 10 |
| LAMA5 | cSPGFHGFPscVPCHcS | 1.763615 | Control(minimal) | control 10 |
| LAMA5 | cSPGFHGFPscVPCHcS | 1.763615 | Control(minimal) | control 10 |
| LAMA5 | PLTGAcRGcLRHTTGPRc | 1.744826 | Control(minimal) | control 10 |
| LAMA5 | PLTGAcRGcLRHTTGPRc | 1.744826 | Control(minimal) | control 10 |
| LAMA5 | PLTGAcRGcLRHTTGPRc | 1.744826 | Control(minimal) | control 10 |
| LAMA5 | PLTGAcRGcLRHTTGPRc | 1.744826 | Control(minimal) | control 10 |
| LAMA5 | PLTGAcRGcLRHTTGPRc | 1.744826 | Control(minimal) | control 10 |
| LAMA5 | PLTGAcRGcLRHTTGPRc | 1.744826 | Control(minimal) | control 10 |
| LAMA5 | PLTGAcRGcLRHTTGPRc | 1.744826 | Control(minimal) | control 10 |
| LAMA5 | PLTGAcRGcLRHTTGPRc | 1.744826 | Control(minimal) | control 10 |
| LAMA5 | PLTGAcRGcLRHTTGPRc | 1.744826 | Control(minimal) | control 10 |
| LAMA5 | cDTcVPGAyNF | 2.278038 | Control(minimal) | control 12 |
| LAMA5 | LcQLcGCSPAGTL | 2.278038 | Control(minimal) | control 12 |
| LAMA5 | LcQLcGCSPAGTL | 2.278038 | Control(minimal) | control 12 |
| LAMA5 | LcQLcGCSPAGTL | 2.278038 | Control(minimal) | control 12 |
| LAMA5 | LcQLcGCSPAGTL | 2.278038 | Control(minimal) | control 12 |
| LAMA5 | LcQLcGCSPAGTL | 2.278038 | Control(minimal) | control 12 |
| LAMA5 | LcQLcGCSPAGTL | 2.278038 | Control(minimal) | control 12 |
| LAMA5 | LcQLcGCSPAGTL | 2.278038 | Control(minimal) | control 12 |
| LAMA5 | SAtCANcTAQSQPVAF | 1.786623 | Control(minimal) | control 12 |
| LAMA5 | SAtCANcTAQSQPVAF | 1.786623 | Control(minimal) | control 12 |
| LAMA5 | SAtCANcTAQSQPVAF | 1.786623 | Control(minimal) | control 12 |
| LAMA5 | sATCANcTAQSQPVAF | 1.786623 | Control(minimal) | control 12 |
| LAMA5 | SAtCANcTAQSQPVAF | 1.786623 | Control(minimal) | control 12 |
| LAMA5 | sATCANcTAQSQPVAF | 1.744527 | Control(minimal) | control 12 |
| LAMA5 | sATCANcTAQSQPVAF | 1.744527 | Control(minimal) | control 12 |
| LAMA5 | sATCANcTAQSQPVAF | 1.744527 | Control(minimal) | control 12 |
| LAMB4 | cLCLsYVTGAHcEEc | 1.743726 | Control(minimal) | control 6 |
| LAMB4 | cLCLsYVTGAHcEEc | 1.743726 | Control(minimal) | control 6 |
| LAMB4 | cLCLsYVTGAHcEEc | 1.743726 | Control(minimal) | control 6 |
| LAMB4 | CLcLsYVTGAHcEEc | 1.743726 | Control(minimal) | control 6 |
| LAMB4 | cLCLsYVTGAHcEEc | 1.743726 | Control(minimal) | control 6 |
| LAMB4 | CLcLsYVTGAHcEEc | 1.743726 | Control(minimal) | control 6 |
| LAMB4 | cLCLsYVTGAHcEEc | 1.743726 | Control(minimal) | control 6 |
| LAMB4 | CLcLsYVTGAHcEEc | 1.743726 | Control(minimal) | control 6 |
| LAMB4 | cLCLsYVTGAHcEEc | 1.743726 | Control(minimal) | control 6 |
| LAMB4 | CLcLsYVTGAHcEEc | 1.743726 | Control(minimal) | control 6 |
| LAMB4 | cLCLsYVTGAHcEEc | 1.743726 | Control(minimal) | control 6 |
| LAMB4 | CLcLsYVTGAHcEEc | 1.743726 | Control(minimal) | control 6 |
| LAMB4 | cLCLsYVTGAHcEEc | 1.743726 | Control(minimal) | control 6 |
| LAMB4 | CLcLsYVTGAHcEEc | 1.743726 | Control(minimal) | control 6 |
| LAMB4 | cLCLsYVTGAHcEEc | 1.743726 | Control(minimal) | control 6 |
| LAMB4 | CLcLsYVTGAHcEEc | 1.743726 | Control(minimal) | control 6 |
| LAMB4 | cLCLsYVTGAHcEEc | 1.743726 | Control(minimal) | control 6 |
| LAMB4 | CLcLsYVTGAHcEEc | 1.743726 | Control(minimal) | control 6 |
| LAMB4 | cLCLsYVTGAHcEEc | 1.743726 | Control(minimal) | control 6 |
| LAMB4 | CLcLsYVTGAHcEEc | 1.743726 | Control(minimal) | control 6 |
| LAMB4 | cLCLsYVTGAHcEEc | 1.743726 | Control(minimal) | control 6 |
| LAMB4 | SDPyAcIPCEcDPDG | 1.80522 | Control(minimal) | control 8 |
| LAMB4 | SDPyAcIPCEcDPDG | 1.80522 | Control(minimal) | control 8 |
| LAMB4 | SDPyAcIPCEcDPDG | 1.80522 | Control(minimal) | control 8 |
| LAMB4 | SDPyAcIPCEcDPDG | 1.80522 | Control(minimal) | control 8 |
| LAMB4 | SDPyAcIPCEcDPDG | 1.80522 | Control(minimal) | control 8 |
| LAMB4 | SDPyAcIPCEcDPDG | 1.80522 | Control(minimal) | control 8 |
| LAMB4 | SDPyAcIPCEcDPDG | 1.80522 | Control(minimal) | control 8 |
| LAMB4 | SDPyAcIPCEcDPDG | 1.80522 | Control(minimal) | control 8 |
| LAMB4 | SDPyAcIPCEcDPDG | 1.80522 | Control(minimal) | control 8 |
| LCE5A | PPVSScCGSSSGGcc | 3.135827 | TBI(extreme) | TBI 1 |
| LCE5A | PPVSSCcGSSSGGcc | 3.135827 | TBI(extreme) | TBI 1 |
| LCE5A | PPVSSCcGSSSGGcc | 3.135827 | TBI(extreme) | TBI 1 |
| LCE5A | PPVSSCcGSSSGGcc | 3.135827 | TBI(extreme) | TBI 1 |
| LCE5A | PPVSScCGSSSGGcc | 1.749494 | TBI(extreme) | TBI 1 |
| LCE5A | PPVSScCGSSSGGcc | 1.749494 | TBI(extreme) | TBI 1 |
| LCE5A | PPVSScCGSSSGGcc | 1.749494 | TBI(extreme) | TBI 1 |
| LCE5A | PPVSScCGSSSGGcc | 1.749494 | TBI(extreme) | TBI 1 |
| LCE5A | PPVSScCGSSSGGcc | 1.749494 | TBI(extreme) | TBI 1 |
| LCE5A | PPVSScCGSSSGGcc | 3.135827 | TBI(extreme) | TBI 8 |
| LCE5A | PPVSSCcGSSSGGcc | 3.135827 | TBI(extreme) | TBI 8 |
| LCE5A | PPVSSCcGSSSGGcc | 3.135827 | TBI(extreme) | TBI 8 |
| LCE5A | PPVSSCcGSSSGGcc | 3.135827 | TBI(extreme) | TBI 8 |
| LCE5A | PPVSScCGSSSGGcc | 1.749494 | TBI(extreme) | TBI 8 |
| LCE5A | PPVSScCGSSSGGcc | 1.749494 | TBI(extreme) | TBI 8 |
| LCE5A | PPVSScCGSSSGGcc | 1.749494 | TBI(extreme) | TBI 8 |
| LCE5A | PPVSScCGSSSGGcc | 1.749494 | TBI(extreme) | TBI 8 |
| LCE5A | PPVSScCGSSSGGcc | 1.749494 | TBI(extreme) | TBI 8 |
| LIMK1 | PItVRccDLDPEKRPS | 1.840199 | TBI(extreme) | TBI 1 |
| LIMK1 | PItVRccDLDPEKRPS | 1.840199 | TBI(extreme) | TBI 1 |
| LIMK1 | PItVRccDLDPEKRPS | 1.840199 | TBI(extreme) | TBI 1 |
| LIMK1 | PItVRccDLDPEKRPS | 1.840199 | TBI(extreme) | TBI 1 |
| LIMK1 | PItVRccDLDPEKRPS | 1.840199 | TBI(extreme) | TBI 1 |
| LIMK1 | PItVRccDLDPEKRPS | 1.840199 | TBI(extreme) | TBI 1 |
| LIMK1 | PItVRccDLDPEKRPS | 1.840199 | TBI(extreme) | TBI 1 |
| LIMK1 | PItVRccDLDPEKRPS | 1.840199 | TBI(extreme) | TBI 1 |
| LIMK1 | PItVRccDLDPEKRPS | 1.840199 | TBI(extreme) | TBI 1 |
| LIMK1 | PItVRccDLDPEKRPS | 1.840199 | TBI(extreme) | TBI 1 |
| LIMK1 | PItVRccDLDPEKRPS | 1.840199 | TBI(extreme) | TBI 1 |
| LIMK1 | PItVRccDLDPEKRPS | 1.840199 | TBI(extreme) | TBI 1 |
| LIMK1 | PItVRccDLDPEKRPS | 1.840199 | TBI(extreme) | TBI 1 |
| LIMK1 | PItVRccDLDPEKRPS | 1.840199 | TBI(extreme) | TBI 8 |
| LIMK1 | PItVRccDLDPEKRPS | 1.840199 | TBI(extreme) | TBI 8 |
| LIMK1 | PItVRccDLDPEKRPS | 1.840199 | TBI(extreme) | TBI 8 |
| LIMK1 | PItVRccDLDPEKRPS | 1.840199 | TBI(extreme) | TBI 8 |
| LIMK1 | PItVRccDLDPEKRPS | 1.840199 | TBI(extreme) | TBI 8 |
| LIMK1 | PItVRccDLDPEKRPS | 1.840199 | TBI(extreme) | TBI 8 |
| LIMK1 | PItVRccDLDPEKRPS | 1.840199 | TBI(extreme) | TBI 8 |
| LIMK1 | PItVRccDLDPEKRPS | 1.840199 | TBI(extreme) | TBI 8 |
| LIMK1 | PItVRccDLDPEKRPS | 1.840199 | TBI(extreme) | TBI 8 |
| LIMK1 | PItVRccDLDPEKRPS | 1.840199 | TBI(extreme) | TBI 8 |
| LIMK1 | PItVRccDLDPEKRPS | 1.840199 | TBI(extreme) | TBI 8 |
| LIMK1 | PItVRccDLDPEKRPS | 1.840199 | TBI(extreme) | TBI 8 |
| LIMK1 | PItVRccDLDPEKRPS | 1.840199 | TBI(extreme) | TBI 8 |
| LIMK1 | PVcAScGQRIyDGQY | 1.75359 | Control(minimal) | control 7 |
| LIMK1 | PVcAScGQRIyDGQY | 1.75359 | Control(minimal) | control 7 |
| LIMK1 | PVcAScGQRIyDGQY | 1.75359 | Control(minimal) | control 7 |
| LIMK1 | PVcAScGQRIyDGQY | 1.75359 | Control(minimal) | control 7 |
| LIMK1 | PVcAScGQRIyDGQY | 1.75359 | Control(minimal) | control 7 |
| LIMK1 | PVcAScGQRIyDGQY | 1.75359 | Control(minimal) | control 7 |
| LIMK1 | PVcAScGQRIyDGQY | 1.75359 | Control(minimal) | control 7 |
| LIMK1 | PVcAScGQRIyDGQY | 1.75359 | Control(minimal) | control 7 |
| LIMK1 | PVcAScGQRIyDGQY | 1.75359 | Control(minimal) | control 7 |
| LIMK1 | PVcAScGQRIyDGQY | 1.75359 | Control(minimal) | control 7 |
| LIMK1 | PVcAScGQRIyDGQY | 1.75359 | Control(minimal) | control 7 |
| LOC100996750 | PScCEtTcCHPRcCIS | 1.874555 | TBI(extreme) | TBI 8 |
| LOC100996750 | PScCEtTcCHPRcCIS | 1.874555 | TBI(extreme) | TBI 8 |
| LOC100996750 | PScCEtTcCHPRcCIS | 1.874555 | TBI(extreme) | TBI 8 |
| LOC100996750 | PScCETtcCHPRcCIS | 1.874555 | TBI(extreme) | TBI 8 |
| LOC100996750 | PScCEtTcCHPRcCIS | 1.874555 | TBI(extreme) | TBI 8 |
| LOC100996750 | PScCEtTcCHPRcCIS | 1.874555 | TBI(extreme) | TBI 8 |
| LOC100996750 | PScCEtTcCHPRcCIS | 1.874555 | TBI(extreme) | TBI 8 |
| LOC100996750 | PScCETtcCHPRcCIS | 1.874555 | TBI(extreme) | TBI 8 |
| LOC100996750 | PScCEtTcCHPRcCIS | 1.874555 | TBI(extreme) | TBI 8 |
| LOC100996750 | PScCEtTcCHPRcCIS | 1.874555 | TBI(extreme) | TBI 8 |
| LOC100996750 | PScCEtTcCHPRcCIS | 1.874555 | TBI(extreme) | TBI 8 |
| LOC100996750 | PScCETtcCHPRcCIS | 1.874555 | TBI(extreme) | TBI 8 |
| LOC100996750 | PScCEtTcCHPRcCIS | 1.874555 | TBI(extreme) | TBI 8 |
| LOC100996750 | PScCEtTccHPRCCIS | 1.731456 | TBI(extreme) | TBI 8 |
| LOC100996750 | PScCEtTccHPRCCIS | 1.731456 | TBI(extreme) | TBI 8 |
| LOC100996750 | PScCEtTccHPRCCIS | 1.731456 | TBI(extreme) | TBI 8 |
| LOC100996750 | PScCEtTccHPRCCIS | 1.731456 | TBI(extreme) | TBI 8 |
| LOC100996750 | PScCEtTccHPRCCIS | 1.731456 | TBI(extreme) | TBI 8 |
| LOC100996750 | PScCEtTccHPRCCIS | 1.731456 | TBI(extreme) | TBI 8 |
| LOC100996750 | PScCEtTccHPRCCIS | 1.731456 | TBI(extreme) | TBI 8 |
| LOC100996750 | PScCEtTccHPRCCIS | 1.731456 | TBI(extreme) | TBI 8 |
| LOC100996750 | PScCEtTccHPRCCIS | 1.731456 | TBI(extreme) | TBI 8 |
| LOC100996750 | PScCEtTccHPRCCIS | 1.731456 | TBI(extreme) | TBI 8 |
| LOC100996750 | TtCCRTTCYRPScC | 1.847985 | TBI(extreme) | TBI 10 |
| LOC100996750 | MVsScCGSVCsDQGcGQ | 1.734045 | Control(minimal) | control 11 |
| LOC100996750 | MVsScCGSVCsDQGcGQ | 1.734045 | Control(minimal) | control 11 |
| LOC100996750 | MVsScCGSVCsDQGcGQ | 1.734045 | Control(minimal) | control 11 |
| LOC100996750 | MVSscCGSVCsDQGcGQ | 1.734045 | Control(minimal) | control 11 |
| LOC100996750 | MVsScCGSVCsDQGcGQ | 1.734045 | Control(minimal) | control 11 |
| LOC100996750 | MVsScCGSVCsDQGcGQ | 1.734045 | Control(minimal) | control 11 |
| LOC100996750 | MVsScCGSVCsDQGcGQ | 1.734045 | Control(minimal) | control 11 |
| LOC100996750 | MVSscCGSVCsDQGcGQ | 1.734045 | Control(minimal) | control 11 |
| LOC100996750 | MVsScCGSVCsDQGcGQ | 1.734045 | Control(minimal) | control 11 |
| LOC100996750 | MVSscCGSVCsDQGcGQ | 1.728146 | Control(minimal) | control 11 |
| LOC100996750 | MVSscCGSVCsDQGcGQ | 1.728146 | Control(minimal) | control 11 |
| LOC100996750 | MVSscCGSVCsDQGcGQ | 1.728146 | Control(minimal) | control 11 |
| LOC100996750 | MVSscCGSVCsDQGcGQ | 1.728146 | Control(minimal) | control 11 |
| LOC100996750 | MVSscCGSVCsDQGcGQ | 1.728146 | Control(minimal) | control 11 |
| LOC100996750 | MVSscCGSVCsDQGcGQ | 1.728146 | Control(minimal) | control 11 |
| LOC100996750 | MVSscCGSVCsDQGcGQ | 1.728146 | Control(minimal) | control 11 |
| LRP1 | GQGPcSHLCLINYNR | 3.423573 | TBI(extreme) | TBI 6 |
| LRP1 | GRVAPScLTcV | 3.423573 | TBI(extreme) | TBI 6 |
| LRP1 | GRVAPScLTcV | 3.423573 | TBI(extreme) | TBI 6 |
| LRP1 | GRVAPScLTcV | 3.423573 | TBI(extreme) | TBI 6 |
| LRP1 | GRVAPScLTcV | 3.423573 | TBI(extreme) | TBI 6 |
| LRP1 | GRVAPScLTcV | 3.423573 | TBI(extreme) | TBI 6 |
| LRP1 | GRVAPScLTcV | 3.423573 | TBI(extreme) | TBI 6 |
| LRP1 | GRVAPScLTcV | 3.423573 | TBI(extreme) | TBI 6 |
| LRP1 | GRVAPScLTcV | 3.423573 | TBI(extreme) | TBI 6 |
| LRP1 | GRVAPScLTcV | 3.423573 | TBI(extreme) | TBI 6 |
| LRP1 | GRVAPScLTcV | 3.423573 | TBI(extreme) | TBI 6 |
| LRP1 | GRVAPScLTcV | 3.423573 | TBI(extreme) | TBI 6 |
| LRP1 | GRVAPScLTcV | 3.423573 | TBI(extreme) | TBI 6 |
| LRP1 | GRVAPScLTcV | 3.423573 | TBI(extreme) | TBI 6 |
| LRP1 | GRVAPScLTcV | 3.423573 | TBI(extreme) | TBI 6 |
| LRP1 | GRVAPScLTcV | 3.423573 | TBI(extreme) | TBI 6 |
| LRP1 | GRVAPScLTcV | 3.423573 | TBI(extreme) | TBI 6 |
| LRP1 | GRVAPScLTcV | 3.423573 | TBI(extreme) | TBI 6 |
| LRP1 | GRVAPScLTcVGHcSNG | 3.423573 | TBI(extreme) | TBI 6 |
| LRP1 | GRVAPScLTcVGHcSNG | 3.423573 | TBI(extreme) | TBI 6 |
| LRP1 | GRVAPScLTcVGHcSNG | 3.423573 | TBI(extreme) | TBI 6 |
| LRP1 | cRcPTGFTGPKcTQQVCA | 1.882247 | TBI(extreme) | TBI 9 |
| LRP1 | cRcPTGFTGPKcTQQVCA | 1.882247 | TBI(extreme) | TBI 9 |
| LRP1 | cRcPTGFTGPKcTQQVCA | 1.882247 | TBI(extreme) | TBI 9 |
| LRP1 | cRcPTGFTGPKcTQQVCA | 1.882247 | TBI(extreme) | TBI 9 |
| LRP1 | SHScSStQFK | 2.900184 | TBI(extreme) | TBI 30 |
| LRP1 | SHScSStQFK | 2.900184 | TBI(extreme) | TBI 30 |
| LRP1 | SHScSStQFK | 2.900184 | TBI(extreme) | TBI 30 |
| LRP1 | SHScSStQFK | 2.900184 | TBI(extreme) | TBI 30 |
| LRP1 | SHScSStQFK | 2.900184 | TBI(extreme) | TBI 30 |
| LRP1 | SHScSStQFK | 2.900184 | TBI(extreme) | TBI 30 |
| LRP1 | SHScSStQFK | 2.900184 | TBI(extreme) | TBI 30 |
| LRP1 | tRPGAFERETVITM | 2.900184 | TBI(extreme) | TBI 30 |
| LRP1 | tCGPSSFScPGTHVcV | 1.932005 | Control(minimal) | control 9 |
| LRP1 | tCGPSSFScPGTHVcV | 1.932005 | Control(minimal) | control 9 |
| LRP1 | tCGPSSFScPGTHVcV | 1.932005 | Control(minimal) | control 9 |
| LRP1 | tCGPSSFScPGTHVcV | 1.932005 | Control(minimal) | control 9 |
| LRP1 | tCGPSSFScPGTHVcV | 1.932005 | Control(minimal) | control 9 |
| LRP1 | tCGPSSFScPGTHVcV | 1.932005 | Control(minimal) | control 9 |
| LRP1 | tCGPSSFScPGTHVcV | 1.932005 | Control(minimal) | control 9 |
| LVRN | VTtQLQIMEWSALE | 1.932189 | TBI(extreme) | TBI 5 |
| LVRN | VTtQLQIMEWSALE | 1.932189 | TBI(extreme) | TBI 5 |
| LVRN | MIHHPsYVALSNmP | 1.872771 | Control(minimal) | control 9 |
| LVRN | MIHHPsYVALSNmP | 1.872771 | Control(minimal) | control 9 |
| LVRN | MIHHPsYVALSNmP | 1.872771 | Control(minimal) | control 9 |
| LVRN | MIHHPsYVALSNmP | 1.872771 | Control(minimal) | control 9 |
| LVRN | MIHHPsYVALSNmP | 1.872771 | Control(minimal) | control 9 |
| LVRN | MIHHPsYVALSNmP | 1.872771 | Control(minimal) | control 9 |
| LVRN | MIHHPsYVALSNmP | 1.872771 | Control(minimal) | control 9 |
| LVRN | MIHHPsYVALSNmP | 1.872771 | Control(minimal) | control 9 |
| LVRN | MIHHPsYVALSNmP | 1.872771 | Control(minimal) | control 9 |
| LVRN | MIHHPsYVALSNmP | 1.872771 | Control(minimal) | control 9 |
| LVRN | MIHHPsYVALSNmP | 1.872771 | Control(minimal) | control 9 |
| LVRN | MIHHPsYVALSNmP | 1.872771 | Control(minimal) | control 9 |
| LVRN | MIHHPsYVALSNmP | 1.872771 | Control(minimal) | control 9 |
| LVRN | MIHHPsYVALSNmP | 1.872771 | Control(minimal) | control 9 |
| LVRN | MIHHPsYVALSNmP | 1.872771 | Control(minimal) | control 9 |
| LVRN | KIQLAYAMscSKDPW | 1.782093 | Control(minimal) | control 10 |
| LVRN | KIQLAYAMscSKDPW | 1.782093 | Control(minimal) | control 10 |
| LVRN | KIQLAYAMscSKDPW | 1.782093 | Control(minimal) | control 10 |
| LVRN | KIQLAYAMscSKDPW | 1.782093 | Control(minimal) | control 10 |
| LVRN | KIQLAYAMscSKDPW | 1.782093 | Control(minimal) | control 10 |
| LVRN | KIQLAYAMscSKDPW | 1.782093 | Control(minimal) | control 10 |
| LVRN | KIQLAYAMscSKDPW | 1.782093 | Control(minimal) | control 10 |
| LVRN | KIQLAYAMscSKDPW | 1.782093 | Control(minimal) | control 10 |
| LVRN | KIQLAYAMscSKDPW | 1.782093 | Control(minimal) | control 10 |
| LVRN | KIQLAYAMscSKDPW | 1.782093 | Control(minimal) | control 10 |
| LVRN | KIQLAYAMscSKDPW | 1.782093 | Control(minimal) | control 10 |
| LVRN | KIQLAYAMscSKDPW | 1.782093 | Control(minimal) | control 10 |
| LVRN | KIQLAYAMscSKDPW | 1.782093 | Control(minimal) | control 10 |
| LY96 | YWVcNSSDASISYt | 1.945299 | TBI(extreme) | TBI 1 |
| LY96 | YWVcNSSDASISYt | 1.945299 | TBI(extreme) | TBI 1 |
| LY96 | YWVcNSSDASISYt | 1.945299 | TBI(extreme) | TBI 1 |
| LY96 | YWVcNSSDASIsYT | 1.945299 | TBI(extreme) | TBI 1 |
| LY96 | YWVcNSSDASIsYT | 1.945299 | TBI(extreme) | TBI 1 |
| LY96 | YWVcNSSDASISYt | 1.945299 | TBI(extreme) | TBI 1 |
| LY96 | YWVcNSSDASISYt | 1.945299 | TBI(extreme) | TBI 1 |
| LY96 | YWVcNSSDASISYt | 1.945299 | TBI(extreme) | TBI 1 |
| LY96 | YWVcNSSDASIsYT | 1.945299 | TBI(extreme) | TBI 1 |
| LY96 | YWVcNSSDASIsYT | 1.945299 | TBI(extreme) | TBI 1 |
| LY96 | YWVcNSSDASIsYT | 1.945299 | TBI(extreme) | TBI 1 |
| LY96 | YWVcNSSDASIsYT | 1.913186 | TBI(extreme) | TBI 1 |
| LY96 | YWVcNSSDASISYt | 1.945299 | TBI(extreme) | TBI 8 |
| LY96 | YWVcNSSDASISYt | 1.945299 | TBI(extreme) | TBI 8 |
| LY96 | YWVcNSSDASISYt | 1.945299 | TBI(extreme) | TBI 8 |
| LY96 | YWVcNSSDASIsYT | 1.945299 | TBI(extreme) | TBI 8 |
| LY96 | YWVcNSSDASIsYT | 1.945299 | TBI(extreme) | TBI 8 |
| LY96 | YWVcNSSDASISYt | 1.945299 | TBI(extreme) | TBI 8 |
| LY96 | YWVcNSSDASISYt | 1.945299 | TBI(extreme) | TBI 8 |
| LY96 | YWVcNSSDASISYt | 1.945299 | TBI(extreme) | TBI 8 |
| LY96 | YWVcNSSDASIsYT | 1.945299 | TBI(extreme) | TBI 8 |
| LY96 | YWVcNSSDASIsYT | 1.945299 | TBI(extreme) | TBI 8 |
| LY96 | YWVcNSSDASIsYT | 1.945299 | TBI(extreme) | TBI 8 |
| LY96 | YWVcNSSDASIsYT | 1.913186 | TBI(extreme) | TBI 8 |
| MALRD1 | VAVLcFLANRKVPIRKT | 1.717122 | TBI(extreme) | TBI 1 |
| MALRD1 | VAVLcFLANRKVPIRKT | 1.717122 | TBI(extreme) | TBI 1 |
| MALRD1 | VAVLcFLANRKVPIRKT | 1.717122 | TBI(extreme) | TBI 1 |
| MALRD1 | VAVLcFLANRKVPIRKT | 1.717122 | TBI(extreme) | TBI 1 |
| MALRD1 | VAVLcFLANRKVPIRKT | 1.717122 | TBI(extreme) | TBI 1 |
| MALRD1 | VAVLcFLANRKVPIRKT | 1.717122 | TBI(extreme) | TBI 1 |
| MALRD1 | VAVLcFLANRKVPIRKT | 1.717122 | TBI(extreme) | TBI 1 |
| MALRD1 | VAVLcFLANRKVPIRKT | 1.717122 | TBI(extreme) | TBI 8 |
| MALRD1 | VAVLcFLANRKVPIRKT | 1.717122 | TBI(extreme) | TBI 8 |
| MALRD1 | VAVLcFLANRKVPIRKT | 1.717122 | TBI(extreme) | TBI 8 |
| MALRD1 | VAVLcFLANRKVPIRKT | 1.717122 | TBI(extreme) | TBI 8 |
| MALRD1 | VAVLcFLANRKVPIRKT | 1.717122 | TBI(extreme) | TBI 8 |
| MALRD1 | VAVLcFLANRKVPIRKT | 1.717122 | TBI(extreme) | TBI 8 |
| MALRD1 | VAVLcFLANRKVPIRKT | 1.717122 | TBI(extreme) | TBI 8 |
| MALRD1 | SYIGDVAVDDIsFQDc | 1.828851 | TBI(extreme) | TBI 47 |
| MALRD1 | SYIGDVAVDDIsFQDc | 1.828851 | TBI(extreme) | TBI 47 |
| MALRD1 | SYIGDVAVDDIsFQDc | 1.828851 | TBI(extreme) | TBI 47 |
| MALRD1 | SYIGDVAVDDIsFQDc | 1.828851 | TBI(extreme) | TBI 47 |
| MALRD1 | SYIGDVAVDDIsFQDc | 1.828851 | TBI(extreme) | TBI 47 |
| MDFIC | CGICTSEACcccCGDEMG | 1.910582 | TBI(extreme) | TBI 30 |
| MDFIC | CGICTSEACcccCGDEMG | 1.910582 | TBI(extreme) | TBI 30 |
| MDFIC | CGICTSEACcccCGDEMG | 1.910582 | TBI(extreme) | TBI 30 |
| MDFIC | CGICTSEACcccCGDEMG | 1.910582 | TBI(extreme) | TBI 30 |
| MDFIC | CGICTSEACcccCGDEMG | 1.910582 | TBI(extreme) | TBI 30 |
| MDFIC | LGQAScGICTSEAccCC | 1.949495 | Control(minimal) | control 9 |
| MDFIC | LGQAScGICTSEAccCC | 1.949495 | Control(minimal) | control 9 |
| MDFIC | LGQAScGICTSEAccCC | 1.949495 | Control(minimal) | control 9 |
| MDFIC | LGQAScGICTSEAcCCc | 1.949495 | Control(minimal) | control 9 |
| MDFIC | LGQAScGICTSEAcCCc | 1.949495 | Control(minimal) | control 9 |
| MDFIC | LGQAScGICTSEAcCcC | 1.949495 | Control(minimal) | control 9 |
| MDFIC | LGQAScGICTSEAccCC | 1.949495 | Control(minimal) | control 9 |
| MDFIC | LGQAScGICTSEAcCcC | 1.949495 | Control(minimal) | control 9 |
| MDFIC | LGQAScGICTSEAccCC | 1.949495 | Control(minimal) | control 9 |
| MDFIC | LGQAScGICTSEAcCCc | 1.946613 | Control(minimal) | control 9 |
| MDFIC | LGQAScGICTSEAcCCc | 1.946613 | Control(minimal) | control 9 |
| MDFIC | LGQAScGICTSEAcCCc | 1.946613 | Control(minimal) | control 9 |
| MDFIC | LGQAScGICTSEAcCcC | 1.934065 | Control(minimal) | control 9 |
| MDFIC | LGQAScGICTSEAcCcC | 1.934065 | Control(minimal) | control 9 |
| MDFIC | LGQAScGICTSEAcCcC | 1.934065 | Control(minimal) | control 9 |
| MDFIC | GDEmGDDCNCPCDMDCGI | 1.748624 | Control(minimal) | control 12 |
| MDFIC | GDEmGDDCNCPCDMDCGI | 1.748624 | Control(minimal) | control 12 |
| MDFIC | GDEmGDDCNCPCDMDCGI | 1.748624 | Control(minimal) | control 12 |
| MDFIC | GDEmGDDCNCPCDMDCGI | 1.748624 | Control(minimal) | control 12 |
| MDFIC | GDEmGDDCNCPCDMDCGI | 1.748624 | Control(minimal) | control 12 |
| MT-CO1 | mITHIcLSISmCPDAF | 1.954738 | TBI(extreme) | TBI 5 |
| MT-CO1 | mITHIcLSISmCPDAF | 1.954738 | TBI(extreme) | TBI 5 |
| MT-CO1 | ISHMCLSMSmcPDAF | 1.725228 | TBI(extreme) | TBI 5 |
| MT-CO1 | ISHMCLSMSmcPDAF | 1.725228 | TBI(extreme) | TBI 5 |
| MT-CO1 | ISHMCLSMSmcPDAF | 1.725228 | TBI(extreme) | TBI 5 |
| MT-CO1 | ISHMCLSMSmcPDAF | 1.725228 | TBI(extreme) | TBI 5 |
| MT-CO1 | ISHMCLSMSmcPDAF | 1.725228 | TBI(extreme) | TBI 5 |
| MT-CO1 | VGIsVWCPHMFTVGLD | 1.784849 | Control(minimal) | control 9 |
| MT-CO1 | VGIsVWCPHMFTVGLD | 1.784849 | Control(minimal) | control 9 |
| MT-CO1 | VGIsVWCPHMFTVGLD | 1.784849 | Control(minimal) | control 9 |
| MT-CO1 | IISHICLNISmNcDS | 1.795444 | Control(minimal) | control 13 |
| MT-CO1 | IISHICLNISmNcDS | 1.795444 | Control(minimal) | control 13 |
| MT-CO1 | IISHICLNISmNcDS | 1.795444 | Control(minimal) | control 13 |
| MT-CO1 | IISHICLNISmNcDS | 1.795444 | Control(minimal) | control 13 |
| MT-CO1 | IISHICLNISmNcDS | 1.795444 | Control(minimal) | control 13 |
| MT-CO1 | IISHICLNISmNcDS | 1.795444 | Control(minimal) | control 13 |
| MT-CO1 | IISHICLNISmNcDS | 1.795444 | Control(minimal) | control 13 |
| MT-CO1 | IISHICLNISmNcDS | 1.795444 | Control(minimal) | control 13 |
| MT-CO1 | IISHICLNISmNcDS | 1.795444 | Control(minimal) | control 13 |
| MT-CO1 | IISHICLNISmNcDS | 1.795444 | Control(minimal) | control 13 |
| MT-CO1 | IISHICLNISmNcDS | 1.795444 | Control(minimal) | control 13 |
| MT-CO1 | VcLGsSVSGHHMFTV | 1.769004 | Control(minimal) | control 13 |
| MT-CO1 | VcLGsSVSGHHMFTV | 1.769004 | Control(minimal) | control 13 |
| MT-CO1 | VcLGsSVSGHHMFTV | 1.769004 | Control(minimal) | control 13 |
| MT-CO1 | VcLGSsVSGHHMFTV | 1.769004 | Control(minimal) | control 13 |
| MT-CO1 | VcLGsSVSGHHMFTV | 1.769004 | Control(minimal) | control 13 |
| MT-CYB | FWSmLSLFVSLTy | 1.920104 | TBI(extreme) | TBI 1 |
| MT-CYB | FWSmLSLFVSLTy | 1.920104 | TBI(extreme) | TBI 1 |
| MT-CYB | FWSmLSLFVSLTy | 1.920104 | TBI(extreme) | TBI 8 |
| MT-CYB | FWSmLSLFVSLTy | 1.920104 | TBI(extreme) | TBI 8 |
| MT-CYB | GSFLYsEtRNIGII | 1.710437 | Control(minimal) | control 11 |
| MT-CYB | GSFLYsEtRNIGII | 1.710437 | Control(minimal) | control 11 |
| MT-CYB | GSFLYsEtRNIGII | 1.710437 | Control(minimal) | control 11 |
| MT-CYB | IFGATIYKYIVGGFSVs | 2.015608 | Control(minimal) | control 12 |
| MT-CYB | IFGATIYKYIVGGFSVs | 2.015608 | Control(minimal) | control 12 |
| MT-CYB | IFGATIYKYIVGGFSVs | 2.015608 | Control(minimal) | control 12 |
| MT-CYB | IFGATIYKYIVGGFSVs | 2.015608 | Control(minimal) | control 12 |
| MT-CYB | IFGATIYKYIVGGFSVs | 2.015608 | Control(minimal) | control 12 |
| MT-CYB | IFGATIYKYIVGGFSVs | 2.015608 | Control(minimal) | control 12 |
| MT-CYB | IFGATIYKYIVGGFSVs | 2.015608 | Control(minimal) | control 12 |
| MT-CYB | IFGATIYKYIVGGFSVs | 2.015608 | Control(minimal) | control 12 |
| MT-CYB | IFGATIYKYIVGGFSVs | 2.015608 | Control(minimal) | control 12 |
| MT-CYB | IFGATIYKYIVGGFSVs | 2.015608 | Control(minimal) | control 12 |
| MT-CYB | IFGATIYKYIVGGFSVs | 2.015608 | Control(minimal) | control 12 |
| MT-CYB | IFGATIYKYIVGGFSVs | 2.015608 | Control(minimal) | control 12 |
| MTERF4 | EKcLFtVQQVTKI | 2.031805 | TBI(extreme) | TBI 2 |
| MTERF4 | EKcLFtVQQVTKI | 2.031805 | TBI(extreme) | TBI 2 |
| MTERF4 | EKcLFtVQQVTKI | 2.031805 | TBI(extreme) | TBI 2 |
| MTERF4 | EKcLFtVQQVTKI | 2.031805 | TBI(extreme) | TBI 2 |
| MTERF4 | EKcLFtVQQVTKI | 2.031805 | TBI(extreme) | TBI 2 |
| MTERF4 | EKcLFtVQQVTKI | 2.031805 | TBI(extreme) | TBI 2 |
| MTERF4 | EKcLFtVQQVTKI | 2.031805 | TBI(extreme) | TBI 2 |
| MTERF4 | EKcLFtVQQVTKI | 2.031805 | TBI(extreme) | TBI 2 |
| MTERF4 | EKcLFtVQQVTKI | 2.031805 | TBI(extreme) | TBI 2 |
| MTERF4 | EKcLFtVQQVTKI | 1.719354 | TBI(extreme) | TBI 30 |
| MTERF4 | EKcLFtVQQVTKI | 1.719354 | TBI(extreme) | TBI 30 |
| MTERF4 | EKcLFtVQQVTKI | 1.719354 | TBI(extreme) | TBI 30 |
| MTERF4 | EKcLFtVQQVTKI | 1.719354 | TBI(extreme) | TBI 30 |
| MTERF4 | EKcLFtVQQVTKI | 1.719354 | TBI(extreme) | TBI 30 |
| MTERF4 | EKcLFtVQQVTKI | 1.719354 | TBI(extreme) | TBI 30 |
| MTERF4 | EKcLFtVQQVTKI | 1.719354 | TBI(extreme) | TBI 30 |
| MTERF4 | EKcLFtVQQVTKI | 1.719354 | TBI(extreme) | TBI 30 |
| MTERF4 | EKcLFtVQQVTKI | 1.719354 | TBI(extreme) | TBI 30 |
| MTERF4 | EKcLFtVQQVTKI | 1.719354 | TBI(extreme) | TBI 30 |
| MTERF4 | EKcLFtVQQVTKI | 1.719354 | TBI(extreme) | TBI 30 |
| MTERF4 | EKcLFtVQQVTKI | 1.719354 | TBI(extreme) | TBI 30 |
| MTERF4 | EKcLFtVQQVTKI | 1.719354 | TBI(extreme) | TBI 30 |
| MTERF4 | EKcLFtVQQVTKI | 1.719354 | TBI(extreme) | TBI 30 |
| MTERF4 | EKcLFtVQQVTKI | 1.719354 | TBI(extreme) | TBI 30 |
| MTMR3 | HGSVGEICYAV | 1.879809 | TBI(extreme) | TBI 5 |
| MTMR3 | HGSVGEICYAV | 1.879809 | TBI(extreme) | TBI 5 |
| MTMR3 | FcSSccNQKVPVPSQ | 1.999866 | TBI(extreme) | TBI 46 |
| MTMR3 | FcSSccNQKVPVPSQ | 1.999866 | TBI(extreme) | TBI 46 |
| MTMR3 | FcSSccNQKVPVPSQ | 1.999866 | TBI(extreme) | TBI 46 |
| MTMR3 | FcSSccNQKVPVPSQ | 1.999866 | TBI(extreme) | TBI 46 |
| MTMR3 | FcSSccNQKVPVPSQ | 1.999866 | TBI(extreme) | TBI 46 |
| MTMR3 | FcSSccNQKVPVPSQ | 1.999866 | TBI(extreme) | TBI 46 |
| MTMR3 | FcSSccNQKVPVPSQ | 1.999866 | TBI(extreme) | TBI 46 |
| MTMR3 | FcSSccNQKVPVPSQ | 1.999866 | TBI(extreme) | TBI 46 |
| MTMR3 | FcSSccNQKVPVPSQ | 1.999866 | TBI(extreme) | TBI 46 |
| MTMR3 | FcSSccNQKVPVPSQ | 1.999866 | TBI(extreme) | TBI 46 |
| MTMR3 | FcSSccNQKVPVPSQ | 1.999866 | TBI(extreme) | TBI 46 |
| MTMR3 | FcSSccNQKVPVPSQ | 1.999866 | TBI(extreme) | TBI 46 |
| MTMR3 | FcSSccNQKVPVPSQ | 1.999866 | TBI(extreme) | TBI 46 |
| MTMR3 | FcSSccNQKVPVPSQ | 1.999866 | TBI(extreme) | TBI 46 |
| MTMR3 | FcSSccNQKVPVPSQ | 1.999866 | TBI(extreme) | TBI 46 |
| MTMR3 | FcSSccNQKVPVPSQ | 1.999866 | TBI(extreme) | TBI 46 |
| MTMR3 | FcSSccNQKVPVPSQ | 1.999866 | TBI(extreme) | TBI 46 |
| MTMR3 | FcSSccNQKVPVPSQ | 1.999866 | TBI(extreme) | TBI 46 |
| MTMR3 | FcSSccNQKVPVPSQ | 1.999866 | TBI(extreme) | TBI 46 |
| MTMR3 | FcSSccNQKVPVPSQ | 1.999866 | TBI(extreme) | TBI 46 |
| MTMR3 | FcSSccNQKVPVPSQ | 1.999866 | TBI(extreme) | TBI 46 |
| MTMR3 | FcSSccNQKVPVPSQ | 1.999866 | TBI(extreme) | TBI 46 |
| MTMR3 | FcSSccNQKVPVPSQ | 1.74608 | TBI(extreme) | TBI 46 |
| MTMR3 | FcSSccNQKVPVPSQ | 1.719179 | TBI(extreme) | TBI 46 |
| MT-ND4 | ICCVVSIFFLITScs | 2.131987 | TBI(extreme) | TBI 5 |
| MT-ND4 | ICCVVSIFFLITScs | 2.131987 | TBI(extreme) | TBI 5 |
| MT-ND4 | ICCVVSIFFLITScs | 2.131987 | TBI(extreme) | TBI 5 |
| MT-ND4 | ICCVVSIFFLITscS | 2.131987 | TBI(extreme) | TBI 5 |
| MT-ND4 | ICCVVSIFFLITScs | 2.131987 | TBI(extreme) | TBI 5 |
| MT-ND4 | ICCVVSIFFLItScS | 2.131987 | TBI(extreme) | TBI 5 |
| MT-ND4 | ICCVVSIFFLITScs | 2.131987 | TBI(extreme) | TBI 5 |
| MT-ND4 | ICCVVSIFFLITscS | 2.080178 | TBI(extreme) | TBI 5 |
| MT-ND4 | ICCVVSIFFLITscS | 2.080178 | TBI(extreme) | TBI 5 |
| MT-ND4 | ICCVVSIFFLITscS | 2.080178 | TBI(extreme) | TBI 5 |
| MT-ND4 | ICCVVSIFFLITscS | 2.080178 | TBI(extreme) | TBI 5 |
| MT-ND4 | LSCIVSIFFLITScs | 1.839507 | TBI(extreme) | TBI 5 |
| MT-ND4 | LSCIVSIFFLITScs | 1.839507 | TBI(extreme) | TBI 5 |
| MT-ND4 | LSCIVSIFFLITScs | 1.839507 | TBI(extreme) | TBI 5 |
| MT-ND4 | LSCIVSIFFLITscS | 1.839507 | TBI(extreme) | TBI 5 |
| MT-ND4 | LSCIVSIFFLITScs | 1.839507 | TBI(extreme) | TBI 5 |
| MT-ND4 | VSLFLTFCsVISVSF | 1.7553 | Control(minimal) | control 7 |
| MT-ND4 | VSLFLTFCsVISVSF | 1.7553 | Control(minimal) | control 7 |
| MT-ND4 | VSLFLTFCsVISVSF | 1.7553 | Control(minimal) | control 7 |
| MT-ND4 | WILLKSSINGVsLm | 2.061841 | Control(minimal) | control 9 |
| MT-ND4 | WILLKSSINGVsLm | 2.061841 | Control(minimal) | control 9 |
| MT-ND4 | WILLKSSINGVsLm | 2.061841 | Control(minimal) | control 9 |
| MT-ND4 | WILLKSSINGVsLm | 2.061841 | Control(minimal) | control 9 |
| MT-ND4 | FLWYLFLcCALSVSF | 1.766033 | Control(minimal) | control 9 |
| MT-ND4 | FLWYLFLcCALSVSF | 1.766033 | Control(minimal) | control 9 |
| MT-ND4 | FLWYLFLcCALSVSF | 1.766033 | Control(minimal) | control 9 |
| MT-ND4 | FLWYLFLCcALSVSF | 1.766033 | Control(minimal) | control 9 |
| MT-ND4 | FLWYLFLcCALSVSF | 1.766033 | Control(minimal) | control 9 |
| MT-ND4 | FLWYLFLCcALSVSF | 1.745833 | Control(minimal) | control 9 |
| MT-ND4 | FLWYLFLCcALSVSF | 1.745833 | Control(minimal) | control 9 |
| MT-ND4 | FLWYLFLCcALSVSF | 1.745833 | Control(minimal) | control 9 |
| MT-ND4 | FLWYLFLcCALSVSF | 1.766033 | Control(minimal) | control 11 |
| MT-ND4 | FLWYLFLcCALSVSF | 1.766033 | Control(minimal) | control 11 |
| MT-ND4 | FLWYLFLcCALSVSF | 1.766033 | Control(minimal) | control 11 |
| MT-ND4 | FLWYLFLCcALSVSF | 1.766033 | Control(minimal) | control 11 |
| MT-ND4 | FLWYLFLcCALSVSF | 1.766033 | Control(minimal) | control 11 |
| MT-ND4 | FLWYLFLCcALSVSF | 1.745833 | Control(minimal) | control 11 |
| MT-ND4 | FLWYLFLCcALSVSF | 1.745833 | Control(minimal) | control 11 |
| MT-ND4 | FLWYLFLCcALSVSF | 1.745833 | Control(minimal) | control 11 |
| MUC19 | CVsGtsWVAPGtTVSP | 2.441126 | TBI(extreme) | TBI 6 |
| MUC19 | CVsGtsWVAPGtTVSP | 2.441126 | TBI(extreme) | TBI 6 |
| MUC19 | CVsGtsWVAPGtTVSP | 2.441126 | TBI(extreme) | TBI 6 |
| MUC19 | RHLPSATTIAPsQDVG | 1.874159 | TBI(extreme) | TBI 8 |
| MUC19 | RHLPSATTIAPsQDVG | 1.874159 | TBI(extreme) | TBI 8 |
| MUC19 | RHLPSATTIAPsQDVG | 1.874159 | TBI(extreme) | TBI 8 |
| MUC19 | TGQSVGVTGTtGPSAG | 1.785431 | TBI(extreme) | TBI 9 |
| MUC19 | TGQSVGVTGTtGPSAG | 1.785431 | TBI(extreme) | TBI 9 |
| MUC19 | TGQSVGVTGTtGPSAG | 1.785431 | TBI(extreme) | TBI 9 |
| MUC19 | TGQSVGVTGTtGPSAG | 1.785431 | TBI(extreme) | TBI 9 |
| MUC19 | TGQSVGVTGTtGPSAG | 1.785431 | TBI(extreme) | TBI 9 |
| MUC19 | SKGICVFPNDcPCsFG | 2.318377 | TBI(extreme) | TBI 30 |
| MUC19 | SKGICVFPNDcPCsFG | 2.318377 | TBI(extreme) | TBI 30 |
| MUC19 | SKGICVFPNDcPCsFG | 2.318377 | TBI(extreme) | TBI 30 |
| MUC19 | SKGICVFPNDcPCsFG | 2.318377 | TBI(extreme) | TBI 30 |
| MUC19 | SKGICVFPNDcPCsFG | 2.318377 | TBI(extreme) | TBI 30 |
| MUC19 | SKGICVFPNDcPCsFG | 2.318377 | TBI(extreme) | TBI 30 |
| MUC2 | GCFcPEGTVYDDIGDSGCV | 1.954579 | TBI(extreme) | TBI 2 |
| MUC2 | GCFcPEGTVYDDIGDSGCV | 1.954579 | TBI(extreme) | TBI 2 |
| MUC2 | GCFcPEGTVYDDIGDSGCV | 1.954579 | TBI(extreme) | TBI 2 |
| MUC2 | cDcycTGWGDPHYV | 1.970244 | TBI(extreme) | TBI 47 |
| MUC2 | cDcycTGWGDPHYV | 1.970244 | TBI(extreme) | TBI 47 |
| MUC2 | cDcycTGWGDPHYV | 1.970244 | TBI(extreme) | TBI 47 |
| MUC2 | cDcycTGWGDPHYV | 1.970244 | TBI(extreme) | TBI 47 |
| MUC2 | cDcycTGWGDPHYV | 1.970244 | TBI(extreme) | TBI 47 |
| MUC2 | cDcycTGWGDPHYV | 1.970244 | TBI(extreme) | TBI 47 |
| MUC2 | cDcycTGWGDPHYV | 1.970244 | TBI(extreme) | TBI 47 |
| MUC2 | cDcycTGWGDPHYV | 1.970244 | TBI(extreme) | TBI 47 |
| MUC2 | cDcycTGWGDPHYV | 1.970244 | TBI(extreme) | TBI 47 |
| MUC2 | cDcYctGWGDPHYV | 1.970244 | TBI(extreme) | TBI 47 |
| MUC2 | cDcycTGWGDPHYV | 1.970244 | TBI(extreme) | TBI 47 |
| MUC2 | cDcYctGWGDPHYV | 1.970244 | TBI(extreme) | TBI 47 |
| MUC2 | cDcycTGWGDPHYV | 1.970244 | TBI(extreme) | TBI 47 |
| MUC2 | cDcYctGWGDPHYV | 1.970244 | TBI(extreme) | TBI 47 |
| MUC2 | cDcycTGWGDPHYV | 1.970244 | TBI(extreme) | TBI 47 |
| MUC2 | cDcYctGWGDPHYV | 1.970244 | TBI(extreme) | TBI 47 |
| MUC2 | cDcycTGWGDPHYV | 1.970244 | TBI(extreme) | TBI 47 |
| MUC2 | cDcycTGWGDPHYV | 1.970244 | TBI(extreme) | TBI 47 |
| MUC2 | cDcycTGWGDPHYV | 1.970244 | TBI(extreme) | TBI 47 |
| MUC2 | cDcYctGWGDPHYV | 1.970244 | TBI(extreme) | TBI 47 |
| MUC2 | cDcycTGWGDPHYV | 1.970244 | TBI(extreme) | TBI 47 |
| MUC2 | cDcYctGWGDPHYV | 1.970244 | TBI(extreme) | TBI 47 |
| MUC2 | cDcycTGWGDPHYV | 1.970244 | TBI(extreme) | TBI 47 |
| MUC2 | cDcYctGWGDPHYV | 1.970244 | TBI(extreme) | TBI 47 |
| MUC2 | cDcycTGWGDPHYV | 1.970244 | TBI(extreme) | TBI 47 |
| MUC2 | cDcYctGWGDPHYV | 1.970244 | TBI(extreme) | TBI 47 |
| MUC2 | cDcycTGWGDPHYV | 1.970244 | TBI(extreme) | TBI 47 |
| MUC2 | cDcYctGWGDPHYV | 1.970244 | TBI(extreme) | TBI 47 |
| MUC2 | cDcycTGWGDPHYV | 1.970244 | TBI(extreme) | TBI 47 |
| MUC2 | cDcYctGWGDPHYV | 1.970244 | TBI(extreme) | TBI 47 |
| MUC2 | cDcycTGWGDPHYV | 1.970244 | TBI(extreme) | TBI 47 |
| MUC2 | cDcYctGWGDPHYV | 1.970244 | TBI(extreme) | TBI 47 |
| MUC2 | cDcycTGWGDPHYV | 1.970244 | TBI(extreme) | TBI 47 |
| MUC2 | cDcycTGWGDPHYV | 1.872327 | TBI(extreme) | TBI 47 |
| MUC2 | cDcycTGWGDPHYV | 1.872327 | TBI(extreme) | TBI 47 |
| MUC2 | cDcycTGWGDPHYV | 1.872327 | TBI(extreme) | TBI 47 |
| MUC2 | cDcycTGWGDPHYV | 1.872327 | TBI(extreme) | TBI 47 |
| MUC2 | cDcycTGWGDPHYV | 1.872327 | TBI(extreme) | TBI 47 |
| MUC2 | cDcycTGWGDPHYV | 1.872327 | TBI(extreme) | TBI 47 |
| MUC2 | cDcycTGWGDPHYV | 1.872327 | TBI(extreme) | TBI 47 |
| MUC2 | cDcycTGWGDPHYV | 1.872327 | TBI(extreme) | TBI 47 |
| MUC2 | cDcycTGWGDPHYV | 1.872327 | TBI(extreme) | TBI 47 |
| MUC2 | cDcycTGWGDPHYV | 1.872327 | TBI(extreme) | TBI 47 |
| MUC2 | cDcycTGWGDPHYV | 1.872327 | TBI(extreme) | TBI 47 |
| MUC2 | cDcycTGWGDPHYV | 1.872327 | TBI(extreme) | TBI 47 |
| MUC2 | cDcycTGWGDPHYV | 1.872327 | TBI(extreme) | TBI 47 |
| MUC2 | cDcYctGWGDPHYV | 1.729519 | TBI(extreme) | TBI 47 |
| MUC2 | cDcYctGWGDPHYV | 1.729519 | TBI(extreme) | TBI 47 |
| MUC2 | cDcYctGWGDPHYV | 1.729519 | TBI(extreme) | TBI 47 |
| MUC2 | cDcYctGWGDPHYV | 1.729519 | TBI(extreme) | TBI 47 |
| MUC2 | cDcYctGWGDPHYV | 1.729519 | TBI(extreme) | TBI 47 |
| MUC2 | cDcYctGWGDPHYV | 1.729519 | TBI(extreme) | TBI 47 |
| MUC2 | cDcYctGWGDPHYV | 1.729519 | TBI(extreme) | TBI 47 |
| MUC2 | cDcYctGWGDPHYV | 1.729519 | TBI(extreme) | TBI 47 |
| MUC2 | cDcYctGWGDPHYV | 1.729519 | TBI(extreme) | TBI 47 |
| MUC2 | cDcYctGWGDPHYV | 1.729519 | TBI(extreme) | TBI 47 |
| MUC2 | cDcYctGWGDPHYV | 1.729519 | TBI(extreme) | TBI 47 |
| MUC2 | cDcYctGWGDPHYV | 1.729519 | TBI(extreme) | TBI 47 |
| MUC2 | cDcYctGWGDPHYV | 1.729519 | TBI(extreme) | TBI 47 |
| MUC5AC | TSTTSAPTTSTTsASTTST | 3.86754 | TBI(extreme) | TBI 47 |
| MUC5AC | TSTTSAPTTSTTsASTTST | 3.86754 | TBI(extreme) | TBI 47 |
| MUC5AC | TSTTSAPTTSTTsASTTST | 3.86754 | TBI(extreme) | TBI 47 |
| MUC5AC | TSTTSAPTTSTTsASTTST | 3.86754 | TBI(extreme) | TBI 47 |
| MUC5AC | TSTTSAPTTSTTsASTTST | 3.86754 | TBI(extreme) | TBI 47 |
| MUC5AC | TSTTSAPTTSTTsASTTST | 3.86754 | TBI(extreme) | TBI 47 |
| MUC5AC | TTSTTSAPTSStTSATTTS | 3.86754 | TBI(extreme) | TBI 47 |
| MUC5AC | TTSTTSAPTSStTSATTTS | 3.86754 | TBI(extreme) | TBI 47 |
| MUC5AC | TTSTTSAPTSStTSATTTS | 3.86754 | TBI(extreme) | TBI 47 |
| MUC5AC | TTSTTSAPTSStTSATTTS | 3.86754 | TBI(extreme) | TBI 47 |
| MUC5AC | TTSTTSAPTSStTSATTTS | 3.86754 | TBI(extreme) | TBI 47 |
| MUC5AC | TTSTTSAPTSStTSATTTS | 3.86754 | TBI(extreme) | TBI 47 |
| MUC5AC | TTSTTSAPTSsTTSATTTS | 3.86754 | TBI(extreme) | TBI 47 |
| MUC5AC | TTSTTSAPTSsTTSATTTS | 3.86754 | TBI(extreme) | TBI 47 |
| MUC5AC | TTSTTSAPTSsTTSATTTS | 3.86754 | TBI(extreme) | TBI 47 |
| MUC5AC | TTSTTSAPTSsTTSATTTS | 3.86754 | TBI(extreme) | TBI 47 |
| MUC5AC | TTSTTSAPTSsTTSATTTS | 3.86754 | TBI(extreme) | TBI 47 |
| MUC5AC | TTSTTSAPTSsTTSATTTS | 3.86754 | TBI(extreme) | TBI 47 |
| MUC5AC | TTSTTSAPTSSTtSATTTS | 3.86754 | TBI(extreme) | TBI 47 |
| MUC5AC | TTSTTSAPTSSTtSATTTS | 3.86754 | TBI(extreme) | TBI 47 |
| MUC5AC | TTSTTSAPTSSTtSATTTS | 3.86754 | TBI(extreme) | TBI 47 |
| MUC5AC | TTSTTSAPTSSTtSATTTS | 3.86754 | TBI(extreme) | TBI 47 |
| MUC5AC | TTSTTSAPTSSTtSATTTS | 3.86754 | TBI(extreme) | TBI 47 |
| MUC5AC | TTSTTSAPTSSTtSATTTS | 3.86754 | TBI(extreme) | TBI 47 |
| MUC5AC | TTSTTSAPTSStTSATTTS | 2.005257 | TBI(extreme) | TBI 47 |
| MUC5AC | TTSTTSAPTSStTSATTTS | 2.005257 | TBI(extreme) | TBI 47 |
| MUC5AC | TTSTTSAPTSStTSATTTS | 2.005257 | TBI(extreme) | TBI 47 |
| MUC5AC | TTSTTSAPTSStTSATTTS | 2.005257 | TBI(extreme) | TBI 47 |
| MUC5AC | TTSTTSAPTSStTSATTTS | 2.005257 | TBI(extreme) | TBI 47 |
| MUC5AC | TTSTTSAPTSsTTSATTTS | 1.968579 | TBI(extreme) | TBI 47 |
| MUC5AC | TTSTTSAPTSsTTSATTTS | 1.968579 | TBI(extreme) | TBI 47 |
| MUC5AC | TTSTTSAPTSsTTSATTTS | 1.968579 | TBI(extreme) | TBI 47 |
| MUC5AC | TTSTTSAPTSsTTSATTTS | 1.968579 | TBI(extreme) | TBI 47 |
| MUC5AC | TTSTTSAPTSsTTSATTTS | 1.968579 | TBI(extreme) | TBI 47 |
| MUC5AC | TTSTTSAPTSSTtSATTTS | 1.909621 | TBI(extreme) | TBI 47 |
| MUC5AC | TTSTTSAPTSSTtSATTTS | 1.909621 | TBI(extreme) | TBI 47 |
| MUC5AC | TTSTTSAPTSSTtSATTTS | 1.909621 | TBI(extreme) | TBI 47 |
| MUC5AC | TTSTTSAPTSSTtSATTTS | 1.909621 | TBI(extreme) | TBI 47 |
| MUC5AC | TTSTTSAPTSSTtSATTTS | 1.909621 | TBI(extreme) | TBI 47 |
| MUC5AC | TSTTSAPTTSTTsASTTST | 1.862283 | TBI(extreme) | TBI 47 |
| MUC5AC | TSTTSAPTTSTTsASTTST | 1.862283 | TBI(extreme) | TBI 47 |
| MUC5AC | TSTTSAPTTSTTsASTTST | 1.862283 | TBI(extreme) | TBI 47 |
| MUC5AC | TSTTSAPTTSTTsASTTST | 1.862283 | TBI(extreme) | TBI 47 |
| MUC5AC | TSTTSAPTTSTTsASTTST | 1.862283 | TBI(extreme) | TBI 47 |
| MUC5AC | CQPLKcSPPPVPVtSP | 1.705391 | TBI(extreme) | TBI 47 |
| MUC5AC | CQPLKcSPPPVPVtSP | 1.705391 | TBI(extreme) | TBI 47 |
| MUC5AC | CQPLKcSPPPVPVtSP | 1.705391 | TBI(extreme) | TBI 47 |
| MUC5AC | CQPLKcSPPPVPVTsP | 1.705391 | TBI(extreme) | TBI 47 |
| MUC5AC | CQPLKcSPPPVPVtSP | 1.705391 | TBI(extreme) | TBI 47 |
| MUC5AC | cTcTHGKLScIG | 1.936055 | Control(minimal) | control 4 |
| MUC5AC | cTcTHGKLScIG | 1.936055 | Control(minimal) | control 4 |
| MUC5AC | cTcTHGKLScIG | 1.936055 | Control(minimal) | control 4 |
| MUC5AC | cTcTHGKLScIG | 1.936055 | Control(minimal) | control 4 |
| MUC5AC | cTcTHGKLScIG | 1.936055 | Control(minimal) | control 4 |
| MUC5AC | cTcTHGKLScIG | 1.936055 | Control(minimal) | control 4 |
| MUC5AC | cTcTHGKLScIG | 1.936055 | Control(minimal) | control 4 |
| MUC5AC | cTcTHGKLScIG | 1.936055 | Control(minimal) | control 4 |
| MUC5AC | cTcTHGKLScIG | 1.936055 | Control(minimal) | control 4 |
| MUC5AC | cTcTHGKLScIG | 1.936055 | Control(minimal) | control 4 |
| MUC5AC | cTcTHGKLScIG | 1.936055 | Control(minimal) | control 4 |
| MUC5AC | GcVPVSKcACVYN | 1.936055 | Control(minimal) | control 4 |
| MUC5AC | VTSLRNVTcLcSsHKA | 1.764963 | Control(minimal) | control 7 |
| MUC5AC | VTSLRNVTcLcSsHKA | 1.764963 | Control(minimal) | control 7 |
| MUC5AC | VTSLRNVTcLcSsHKA | 1.764963 | Control(minimal) | control 7 |
| MUC5AC | SNQEHsRAcEDHCVAG | 1.748829 | Control(minimal) | control 11 |
| MUC5AC | SNQEHsRAcEDHCVAG | 1.748829 | Control(minimal) | control 11 |
| MUC5AC | SNQEHsRAcEDHCVAG | 1.748829 | Control(minimal) | control 11 |
| MUC5AC | SNQEHsRAcEDHCVAG | 1.748829 | Control(minimal) | control 11 |
| MUC5AC | SNQEHsRAcEDHCVAG | 1.748829 | Control(minimal) | control 11 |
| MUC5AC | SNQEHsRAcEDHCVAG | 1.748829 | Control(minimal) | control 11 |
| MUC5AC | SNQEHsRAcEDHCVAG | 1.748829 | Control(minimal) | control 11 |
| MUC5AC | SNQEHsRAcEDHCVAG | 1.748829 | Control(minimal) | control 11 |
| MUC5AC | SNQEHsRAcEDHCVAG | 1.748829 | Control(minimal) | control 11 |
| NBAS | LsVEARKEmTRKAI | 1.83825 | Control(minimal) | control 8 |
| NBAS | LsVEARKEmTRKAI | 1.83825 | Control(minimal) | control 8 |
| NBAS | LsVEARKEmTRKAI | 1.83825 | Control(minimal) | control 8 |
| NBAS | LsVEARKEmTRKAI | 1.83825 | Control(minimal) | control 8 |
| NBAS | LsVEARKEmTRKAI | 1.83825 | Control(minimal) | control 8 |
| NBAS | LsVEARKEmTRKAI | 1.83825 | Control(minimal) | control 8 |
| NBAS | LsVEARKEmTRKAI | 1.83825 | Control(minimal) | control 8 |
| NBAS | LsVEARKEmTRKAI | 1.83825 | Control(minimal) | control 8 |
| NBAS | LsVEARKEmTRKAI | 1.83825 | Control(minimal) | control 8 |
| NBAS | LsVEARKEmTRKAI | 1.83825 | Control(minimal) | control 8 |
| NBAS | LsVEARKEmTRKAI | 1.83825 | Control(minimal) | control 8 |
| NBAS | LsVEARKEmTRKAI | 1.83825 | Control(minimal) | control 8 |
| NBAS | LsVEARKEmTRKAI | 1.83825 | Control(minimal) | control 8 |
| NBAS | LTHCPPSSIELLLAASSSL | 2.8256 | Control(minimal) | control 12 |
| NBAS | LTHCPPSSIELLLAASSSL | 2.8256 | Control(minimal) | control 12 |
| NBAS | LTHCPPSSIELLLAASSSL | 2.8256 | Control(minimal) | control 12 |
| NBAS | LTHCPPSSIELLLAASSSL | 2.8256 | Control(minimal) | control 12 |
| NBAS | LTHCPPSSIELLLAASSSL | 2.8256 | Control(minimal) | control 12 |
| NBAS | LTHCPPSSIELLLAASSSL | 2.8256 | Control(minimal) | control 12 |
| NBAS | LTHCPPSSIELLLAASSSL | 2.8256 | Control(minimal) | control 12 |
| NBAS | LTHCPPSSIELLLAASSSL | 2.8256 | Control(minimal) | control 12 |
| NBAS | LTHCPPSSIELLLAASSSL | 2.8256 | Control(minimal) | control 12 |
| NBAS | LTHCPPSSIELLLAASSSL | 2.8256 | Control(minimal) | control 12 |
| NBAS | LTHCPPSSIELLLAASSSL | 2.8256 | Control(minimal) | control 12 |
| NBAS | NDQLcLcyDLLECL | 2.8256 | Control(minimal) | control 12 |
| NFX1 | CLTVSHTsVcRSISC | 2.260651 | TBI(extreme) | TBI 10 |
| NFX1 | CLTVSHTsVcRSISC | 2.221329 | TBI(extreme) | TBI 10 |
| NFX1 | CLTVSHTsVcRSISC | 2.221329 | TBI(extreme) | TBI 10 |
| NFX1 | CLTVSHTsVcRSISC | 2.221329 | TBI(extreme) | TBI 10 |
| NFX1 | CLTVSHTsVcRSISC | 2.221329 | TBI(extreme) | TBI 10 |
| NFX1 | CLTVSHTsVcRSISC | 2.221329 | TBI(extreme) | TBI 10 |
| NFX1 | CLTVSHTsVcRSISC | 2.221329 | TBI(extreme) | TBI 10 |
| NFX1 | CLTVSHTsVcRSISC | 2.221329 | TBI(extreme) | TBI 10 |
| NFX1 | CLTVSHTsVcRSISC | 2.221329 | TBI(extreme) | TBI 10 |
| NFX1 | CLTVSHTsVcRSISC | 2.221329 | TBI(extreme) | TBI 10 |
| NFX1 | CLTVSHTsVcRSISC | 2.221329 | TBI(extreme) | TBI 10 |
| NFX1 | CLTVSHTsVcRSISC | 2.221329 | TBI(extreme) | TBI 10 |
| NFX1 | CLTVSHTsVcRSISC | 2.21638 | TBI(extreme) | TBI 10 |
| NFX1 | CLTVSHTsVcRSISC | 2.14022 | TBI(extreme) | TBI 10 |
| NFX1 | CLTVSHTsVcRSISC | 2.138872 | TBI(extreme) | TBI 10 |
| NFX1 | CLTVSHTsVcRSISC | 2.136519 | TBI(extreme) | TBI 10 |
| NFX1 | CLTVSHTsVcRSISC | 2.105746 | TBI(extreme) | TBI 10 |
| NFX1 | GKsDGFGDFSCLKTCGK | 4.130253 | TBI(extreme) | TBI 30 |
| NFX1 | KPLPCGSLDF | 4.130253 | TBI(extreme) | TBI 30 |
| NFX1 | VDIScGLPCSAT | 4.130253 | TBI(extreme) | TBI 30 |
| NFX1 | KPLPCGSLDF | 2.246494 | TBI(extreme) | TBI 30 |
| NFX1 | KPLPCGSLDF | 2.246494 | TBI(extreme) | TBI 30 |
| NFX1 | KPLPCGSLDF | 2.246494 | TBI(extreme) | TBI 30 |
| NFX1 | KPLPCGSLDF | 2.246494 | TBI(extreme) | TBI 30 |
| NFX1 | KPLPCGSLDF | 2.246494 | TBI(extreme) | TBI 30 |
| NFX1 | KPLPCGSLDF | 2.246494 | TBI(extreme) | TBI 30 |
| NFX1 | KPLPCGSLDF | 2.246494 | TBI(extreme) | TBI 30 |
| NFX1 | KPLPCGSLDF | 2.246494 | TBI(extreme) | TBI 30 |
| NFX1 | GKsDGFGDFSCLKTCGK | 1.883759 | TBI(extreme) | TBI 30 |
| NFX1 | GKsDGFGDFSCLKTCGK | 1.883759 | TBI(extreme) | TBI 30 |
| NFX1 | GKsDGFGDFSCLKTCGK | 1.883759 | TBI(extreme) | TBI 30 |
| NFX1 | GKsDGFGDFSCLKTCGK | 1.883759 | TBI(extreme) | TBI 30 |
| NFX1 | GFGDFsCLKTcGKDLK | 1.95044 | Control(minimal) | control 13 |
| NFX1 | GFGDFsCLKTcGKDLK | 1.95044 | Control(minimal) | control 13 |
| NFX1 | GFGDFsCLKTcGKDLK | 1.95044 | Control(minimal) | control 13 |
| NOTCH4 | SGYGGPDcLTPPAPKGcGPP | 1.704055 | TBI(extreme) | TBI 1 |
| NOTCH4 | SGYGGPDcLTPPAPKGcGPP | 1.704055 | TBI(extreme) | TBI 1 |
| NOTCH4 | SGYGGPDcLTPPAPKGcGPP | 1.704055 | TBI(extreme) | TBI 1 |
| NOTCH4 | SGYGGPDcLTPPAPKGcGPP | 1.704055 | TBI(extreme) | TBI 1 |
| NOTCH4 | SGYGGPDcLTPPAPKGcGPP | 1.704055 | TBI(extreme) | TBI 1 |
| NOTCH4 | SGYGGPDcLTPPAPKGcGPP | 1.704055 | TBI(extreme) | TBI 1 |
| NOTCH4 | SGYGGPDcLTPPAPKGcGPP | 1.704055 | TBI(extreme) | TBI 1 |
| NOTCH4 | SGYGGPDcLTPPAPKGcGPP | 1.704055 | TBI(extreme) | TBI 8 |
| NOTCH4 | SGYGGPDcLTPPAPKGcGPP | 1.704055 | TBI(extreme) | TBI 8 |
| NOTCH4 | SGYGGPDcLTPPAPKGcGPP | 1.704055 | TBI(extreme) | TBI 8 |
| NOTCH4 | SGYGGPDcLTPPAPKGcGPP | 1.704055 | TBI(extreme) | TBI 8 |
| NOTCH4 | SGYGGPDcLTPPAPKGcGPP | 1.704055 | TBI(extreme) | TBI 8 |
| NOTCH4 | SGYGGPDcLTPPAPKGcGPP | 1.704055 | TBI(extreme) | TBI 8 |
| NOTCH4 | SGYGGPDcLTPPAPKGcGPP | 1.704055 | TBI(extreme) | TBI 8 |
| NOTCH4 | DFcSANPcVNGGVcLA | 1.787546 | TBI(extreme) | TBI 9 |
| NOTCH4 | DFcSANPcVNGGVcLA | 1.787546 | TBI(extreme) | TBI 9 |
| NOTCH4 | DFcSANPcVNGGVcLA | 1.787546 | TBI(extreme) | TBI 9 |
| NOTCH4 | DFcSANPcVNGGVcLA | 1.787546 | TBI(extreme) | TBI 9 |
| NOTCH4 | DFcSANPcVNGGVcLA | 1.787546 | TBI(extreme) | TBI 9 |
| NOTCH4 | DFcSANPcVNGGVcLA | 1.787546 | TBI(extreme) | TBI 9 |
| NOTCH4 | DFcSANPcVNGGVcLA | 1.787546 | TBI(extreme) | TBI 9 |
| NOTCH4 | DFcSANPcVNGGVcLA | 1.787546 | TBI(extreme) | TBI 9 |
| NOTCH4 | DFcSANPcVNGGVcLA | 1.787546 | TBI(extreme) | TBI 9 |
| NOTCH4 | DFcSANPcVNGGVcLA | 1.787546 | TBI(extreme) | TBI 9 |
| NOTCH4 | DFcSANPcVNGGVcLA | 1.787546 | TBI(extreme) | TBI 9 |
| NOTCH4 | DFcSANPcVNGGVcLA | 1.787546 | TBI(extreme) | TBI 9 |
| NOTCH4 | DFcSANPcVNGGVcLA | 1.787546 | TBI(extreme) | TBI 9 |
| NOTCH4 | YPQPsGYNCTcPTGY | 1.760509 | TBI(extreme) | TBI 30 |
| NOTCH4 | YPQPsGYNCTcPTGY | 1.760509 | TBI(extreme) | TBI 30 |
| NOTCH4 | YPQPsGYNCTcPTGY | 1.760509 | TBI(extreme) | TBI 30 |
| NOTCH4 | YPQPsGYNCTcPTGY | 1.760509 | TBI(extreme) | TBI 30 |
| NOTCH4 | YPQPsGYNCTcPTGY | 1.760509 | TBI(extreme) | TBI 30 |
| NOTCH4 | YPQPsGYNCTcPTGY | 1.760509 | TBI(extreme) | TBI 30 |
| NOTCH4 | YPQPsGYNCTcPTGY | 1.760509 | TBI(extreme) | TBI 30 |
| NOTCH4 | YPQPsGYNCTcPTGY | 1.760509 | TBI(extreme) | TBI 30 |
| NOTCH4 | YPQPsGYNCTcPTGY | 1.760509 | TBI(extreme) | TBI 30 |
| NOTCH4 | AcHSGPCLNGGScNPSPGGY | 3.618993 | Control(minimal) | control 3 |
| NOTCH4 | AcHSGPCLNGGScNPSPGGY | 3.618993 | Control(minimal) | control 3 |
| NOTCH4 | AcHSGPCLNGGScNPSPGGY | 3.618993 | Control(minimal) | control 3 |
| NOTCH4 | AcHSGPCLNGGScNPSPGGY | 3.618993 | Control(minimal) | control 3 |
| NOTCH4 | SYFCHCPPGFQGsLcQ | 3.618993 | Control(minimal) | control 3 |
| NOTCH4 | SYFCHCPPGFQGsLcQ | 3.618993 | Control(minimal) | control 3 |
| NOTCH4 | SYFCHCPPGFQGsLcQ | 3.618993 | Control(minimal) | control 3 |
| NOTCH4 | SYFCHCPPGFQGsLcQ | 3.618993 | Control(minimal) | control 3 |
| NOTCH4 | AcHSGPCLNGGScNPSPGGY | 1.955903 | Control(minimal) | control 3 |
| NOTCH4 | AcHSGPCLNGGScNPSPGGY | 1.955903 | Control(minimal) | control 3 |
| NOTCH4 | AcHSGPCLNGGScNPSPGGY | 1.955903 | Control(minimal) | control 3 |
| NOTCH4 | AcHSGPCLNGGScNPSPGGY | 1.955903 | Control(minimal) | control 3 |
| NOTCH4 | AcHSGPCLNGGScNPSPGGY | 1.955903 | Control(minimal) | control 3 |
| NOTCH4 | cEKGcNT | 3.111808 | Control(minimal) | control 4 |
| NOTCH4 | cEKGcNT | 3.111808 | Control(minimal) | control 4 |
| NOTCH4 | cEKGcNT | 3.111808 | Control(minimal) | control 4 |
| NOTCH4 | cELRAGPCPPRGcSNGGt | 3.111808 | Control(minimal) | control 4 |
| NOTCH4 | cELRAGPCPPRGcSNGGt | 3.111808 | Control(minimal) | control 4 |
| NOTCH4 | cELRAGPCPPRGcSNGGt | 3.111808 | Control(minimal) | control 4 |
| NOTCH4 | cELRAGPCPPRGcsNGGT | 3.111808 | Control(minimal) | control 4 |
| NOTCH4 | cELRAGPCPPRGcsNGGT | 3.111808 | Control(minimal) | control 4 |
| NOTCH4 | cELRAGPCPPRGcsNGGT | 3.111808 | Control(minimal) | control 4 |
| NOTCH4 | cEKGcNT | 2.113183 | Control(minimal) | control 4 |
| NOTCH4 | cEKGcNT | 2.113183 | Control(minimal) | control 4 |
| NOTCH4 | cEKGcNT | 2.113183 | Control(minimal) | control 4 |
| NOTCH4 | cEKGcNT | 2.113183 | Control(minimal) | control 4 |
| NOTCH4 | cELRAGPCPPRGcSNGGt | 2.113183 | Control(minimal) | control 4 |
| NOTCH4 | cELRAGPCPPRGcSNGGt | 2.113183 | Control(minimal) | control 4 |
| NOTCH4 | cELRAGPCPPRGcSNGGt | 2.113183 | Control(minimal) | control 4 |
| NOTCH4 | cELRAGPCPPRGcSNGGt | 2.113183 | Control(minimal) | control 4 |
| NOTCH4 | cELRAGPCPPRGcSNGGt | 2.113183 | Control(minimal) | control 4 |
| NOTCH4 | cELRAGPCPPRGcSNGGt | 2.113183 | Control(minimal) | control 4 |
| NOTCH4 | cELRAGPCPPRGcsNGGT | 1.872087 | Control(minimal) | control 4 |
| NOTCH4 | cELRAGPCPPRGcsNGGT | 1.872087 | Control(minimal) | control 4 |
| NOTCH4 | cELRAGPCPPRGcsNGGT | 1.872087 | Control(minimal) | control 4 |
| NOTCH4 | cELRAGPCPPRGcsNGGT | 1.872087 | Control(minimal) | control 4 |
| NOTCH4 | cELRAGPCPPRGcsNGGT | 1.872087 | Control(minimal) | control 4 |
| NOTCH4 | cELRAGPCPPRGcsNGGT | 1.872087 | Control(minimal) | control 4 |
| NSD1 | cDICGKEAAsFcEMcP | 1.802605 | TBI(extreme) | TBI 1 |
| NSD1 | cDICGKEAAsFcEMcP | 1.802605 | TBI(extreme) | TBI 1 |
| NSD1 | cDICGKEAAsFcEMcP | 1.802605 | TBI(extreme) | TBI 1 |
| NSD1 | cDICGKEAAsFcEMcP | 1.802605 | TBI(extreme) | TBI 1 |
| NSD1 | cDICGKEAAsFcEMcP | 1.802605 | TBI(extreme) | TBI 1 |
| NSD1 | cDICGKEAAsFcEMcP | 1.802605 | TBI(extreme) | TBI 1 |
| NSD1 | cDICGKEAAsFcEMcP | 1.802605 | TBI(extreme) | TBI 1 |
| NSD1 | cDICGKEAAsFcEMcP | 1.802605 | TBI(extreme) | TBI 1 |
| NSD1 | cDICGKEAAsFcEMcP | 1.802605 | TBI(extreme) | TBI 1 |
| NSD1 | cDICGKEAAsFcEMcP | 1.802605 | TBI(extreme) | TBI 1 |
| NSD1 | cDICGKEAAsFcEMcP | 1.802605 | TBI(extreme) | TBI 1 |
| NSD1 | cDICGKEAAsFcEMcP | 1.802605 | TBI(extreme) | TBI 8 |
| NSD1 | cDICGKEAAsFcEMcP | 1.802605 | TBI(extreme) | TBI 8 |
| NSD1 | cDICGKEAAsFcEMcP | 1.802605 | TBI(extreme) | TBI 8 |
| NSD1 | cDICGKEAAsFcEMcP | 1.802605 | TBI(extreme) | TBI 8 |
| NSD1 | cDICGKEAAsFcEMcP | 1.802605 | TBI(extreme) | TBI 8 |
| NSD1 | cDICGKEAAsFcEMcP | 1.802605 | TBI(extreme) | TBI 8 |
| NSD1 | cDICGKEAAsFcEMcP | 1.802605 | TBI(extreme) | TBI 8 |
| NSD1 | cDICGKEAAsFcEMcP | 1.802605 | TBI(extreme) | TBI 8 |
| NSD1 | cDICGKEAAsFcEMcP | 1.802605 | TBI(extreme) | TBI 8 |
| NSD1 | cDICGKEAAsFcEMcP | 1.802605 | TBI(extreme) | TBI 8 |
| NSD1 | cDICGKEAAsFcEMcP | 1.802605 | TBI(extreme) | TBI 8 |
| NSD1 | PPIDLLGEIcGGGLTLLKK | 1.832707 | TBI(extreme) | TBI 10 |
| NSD1 | LAQScWSAGSTQTLAQtC | 1.832615 | TBI(extreme) | TBI 10 |
| NSD1 | LAQScWSAGSTQTLAQtC | 1.832615 | TBI(extreme) | TBI 10 |
| NSD1 | LAQScWSAGSTQTLAQtC | 1.832615 | TBI(extreme) | TBI 10 |
| NSD1 | LAQScWSAGSTQTLAQtC | 1.832615 | TBI(extreme) | TBI 10 |
| NSD1 | LAQScWSAGSTQTLAQtC | 1.832615 | TBI(extreme) | TBI 10 |
| NSD1 | LAQScWSAGSTQTLAQtC | 1.832615 | TBI(extreme) | TBI 10 |
| NSD1 | LAQScWSAGSTQTLAQtC | 1.832615 | TBI(extreme) | TBI 10 |
| NSD1 | LAQScWSAGSTQTLAQtC | 1.832615 | TBI(extreme) | TBI 10 |
| NSD1 | LAQScWSAGSTQTLAQtC | 1.832615 | TBI(extreme) | TBI 10 |
| NSD1 | PPIDLLGEIcGGGLTLLKK | 1.832615 | TBI(extreme) | TBI 10 |
| NSD1 | PPIDLLGEIcGGGLTLLKK | 1.832615 | TBI(extreme) | TBI 10 |
| NSD1 | LAQScWSAGSTQTLAQtC | 1.831703 | TBI(extreme) | TBI 10 |
| NTN3 | ACRAcDcHPVGAAGKtcN | 1.794729 | TBI(extreme) | TBI 5 |
| NTN3 | ACRAcDcHPVGAAGKtcN | 1.794729 | TBI(extreme) | TBI 5 |
| NTN3 | ACRAcDcHPVGAAGKtcN | 1.794729 | TBI(extreme) | TBI 5 |
| NTN3 | AcRACDcHPVGAAGKtcN | 1.794729 | TBI(extreme) | TBI 5 |
| NTN3 | ACRAcDcHPVGAAGKtcN | 1.794729 | TBI(extreme) | TBI 5 |
| NTN3 | AcRAcDcHPVGAAGKTCNQ | 2.229082 | Control(minimal) | control 10 |
| NTN3 | AcRAcDcHPVGAAGKTCNQ | 2.229082 | Control(minimal) | control 10 |
| NTN3 | AcRAcDcHPVGAAGKTCNQ | 2.229082 | Control(minimal) | control 10 |
| NTN3 | AcRAcDcHPVGAAGKTCNQ | 2.229082 | Control(minimal) | control 10 |
| NTN3 | AcRAcDcHPVGAAGKTCNQ | 2.229082 | Control(minimal) | control 10 |
| NTN3 | AcRAcDcHPVGAAGKTCNQ | 2.127669 | Control(minimal) | control 10 |
| NTN3 | AcRAcDcHPVGAAGKTCNQ | 2.127669 | Control(minimal) | control 10 |
| NTN3 | AcRAcDcHPVGAAGKTCNQ | 1.776126 | Control(minimal) | control 10 |
| NTN3 | AcRAcDcHPVGAAGKTCNQ | 1.776126 | Control(minimal) | control 10 |
| NTN3 | cHPVGAAGKTCNQtTGQC | 1.987233 | Control(minimal) | control 12 |
| OTOG | RLcPYDCDFF | 2.336092 | TBI(extreme) | TBI 1 |
| OTOG | TVcVcNQTLCEG | 2.336092 | TBI(extreme) | TBI 1 |
| OTOG | TVcVcNQTLCEG | 2.336092 | TBI(extreme) | TBI 1 |
| OTOG | TVcVcNQTLCEG | 2.336092 | TBI(extreme) | TBI 1 |
| OTOG | TVcVcNQTLCEG | 2.336092 | TBI(extreme) | TBI 1 |
| OTOG | TVcVcNQTLCEG | 2.336092 | TBI(extreme) | TBI 1 |
| OTOG | RLcPYDCDFF | 2.336092 | TBI(extreme) | TBI 8 |
| OTOG | TVcVcNQTLCEG | 2.336092 | TBI(extreme) | TBI 8 |
| OTOG | TVcVcNQTLCEG | 2.336092 | TBI(extreme) | TBI 8 |
| OTOG | TVcVcNQTLCEG | 2.336092 | TBI(extreme) | TBI 8 |
| OTOG | TVcVcNQTLCEG | 2.336092 | TBI(extreme) | TBI 8 |
| OTOG | TVcVcNQTLCEG | 2.336092 | TBI(extreme) | TBI 8 |
| OTOG | tLPPSQGLPTPSDEEP | 2.245657 | Control(minimal) | control 6 |
| OTOG | tLPPSQGLPTPSDEEP | 2.245657 | Control(minimal) | control 6 |
| OTOG | tLPPSQGLPTPSDEEP | 2.245657 | Control(minimal) | control 6 |
| OTOG | tLPPSQGLPTPSDEEP | 2.245657 | Control(minimal) | control 6 |
| OTOG | tLPPSQGLPTPSDEEP | 2.245657 | Control(minimal) | control 6 |
| OTOG | TLPPsQGLPTPSDEEP | 2.245657 | Control(minimal) | control 6 |
| OTOG | TLPPsQGLPTPSDEEP | 2.245657 | Control(minimal) | control 6 |
| OTOG | TLPPsQGLPTPSDEEP | 2.245657 | Control(minimal) | control 6 |
| OTOG | tLPPSQGLPTPSDEEP | 2.245657 | Control(minimal) | control 6 |
| OTOG | TLPPsQGLPTPSDEEP | 2.029679 | Control(minimal) | control 6 |
| OTOG | TLPPsQGLPTPSDEEP | 2.029679 | Control(minimal) | control 6 |
| OTOG | TLPPsQGLPTPSDEEP | 2.029679 | Control(minimal) | control 6 |
| OTOG | HCSSTPLGAVLVRsPISC | 2.192627 | Control(minimal) | control 7 |
| OTOG | HCSSTPLGAVLVRsPISC | 2.192627 | Control(minimal) | control 7 |
| OTOG | HCSSTPLGAVLVRsPISC | 2.192627 | Control(minimal) | control 7 |
| OTOG | HCSSTPLGAVLVRsPISC | 2.192627 | Control(minimal) | control 7 |
| OTOG | HCSSTPLGAVLVRsPISC | 2.192627 | Control(minimal) | control 7 |
| OTOG | HCSSTPLGAVLVRSPIsC | 2.192627 | Control(minimal) | control 7 |
| OTOG | HCSSTPLGAVLVRSPIsC | 2.192627 | Control(minimal) | control 7 |
| OTOG | HCSSTPLGAVLVRSPIsC | 2.192627 | Control(minimal) | control 7 |
| OTOG | HCSSTPLGAVLVRsPISC | 2.192627 | Control(minimal) | control 7 |
| OTOG | HCSSTPLGAVLVRSPIsC | 2.097605 | Control(minimal) | control 7 |
| OTOG | HCSSTPLGAVLVRSPIsC | 2.097605 | Control(minimal) | control 7 |
| OTOG | HCSSTPLGAVLVRSPIsC | 2.097605 | Control(minimal) | control 7 |
| OTOGL | AmNFtCTPSSPCISGc | 2.073928 | TBI(extreme) | TBI 1 |
| OTOGL | AmNFtCTPSSPCISGc | 2.073928 | TBI(extreme) | TBI 1 |
| OTOGL | AmNFtCTPSSPCISGc | 2.073928 | TBI(extreme) | TBI 1 |
| OTOGL | AmNFTCtPSSPCISGc | 2.073928 | TBI(extreme) | TBI 1 |
| OTOGL | AmNFTCtPSSPCISGc | 2.073928 | TBI(extreme) | TBI 1 |
| OTOGL | AmNFtCTPSSPCISGc | 2.073928 | TBI(extreme) | TBI 1 |
| OTOGL | AmNFTCtPSSPCISGc | 2.073928 | TBI(extreme) | TBI 1 |
| OTOGL | AmNFTCtPSSPCISGc | 1.924619 | TBI(extreme) | TBI 1 |
| OTOGL | AmNFTCtPSSPCISGc | 1.924619 | TBI(extreme) | TBI 1 |
| OTOGL | AmNFTCtPSSPCISGc | 1.924619 | TBI(extreme) | TBI 1 |
| OTOGL | tCcSKEVCGcDTTLc | 1.964468 | TBI(extreme) | TBI 46 |
| OTOGL | tCcSKEVCGcDTTLc | 1.964468 | TBI(extreme) | TBI 46 |
| OTOGL | tCcSKEVCGcDTTLc | 1.964468 | TBI(extreme) | TBI 46 |
| OTOGL | tCcSKEVCGcDTTLc | 1.964468 | TBI(extreme) | TBI 46 |
| OTOGL | TcCsKEVCGcDTTLc | 1.964468 | TBI(extreme) | TBI 46 |
| OTOGL | tCcSKEVCGcDTTLc | 1.964468 | TBI(extreme) | TBI 46 |
| OTOGL | tCcSKEVcGCDTTLc | 1.964468 | TBI(extreme) | TBI 46 |
| OTOGL | tCcSKEVCGcDTTLc | 1.964468 | TBI(extreme) | TBI 46 |
| OTOGL | tCcSKEVcGCDTTLc | 1.964468 | TBI(extreme) | TBI 46 |
| OTOGL | tCcSKEVCGcDTTLc | 1.964468 | TBI(extreme) | TBI 46 |
| OTOGL | tcCSKEVCGcDTTLc | 1.964468 | TBI(extreme) | TBI 46 |
| OTOGL | tCcSKEVCGcDTTLc | 1.964468 | TBI(extreme) | TBI 46 |
| OTOGL | tCcSKEVcGCDTTLc | 1.964468 | TBI(extreme) | TBI 46 |
| OTOGL | tCcSKEVCGcDTTLc | 1.964468 | TBI(extreme) | TBI 46 |
| OTOGL | tCcSKEVcGCDTTLc | 1.964468 | TBI(extreme) | TBI 46 |
| OTOGL | tCcSKEVCGcDTTLc | 1.964468 | TBI(extreme) | TBI 46 |
| OTOGL | TCcsKEVCGcDTTLc | 1.964468 | TBI(extreme) | TBI 46 |
| OTOGL | tCcSKEVCGcDTTLc | 1.964468 | TBI(extreme) | TBI 46 |
| OTOGL | tCcSKEVcGCDTTLc | 1.964468 | TBI(extreme) | TBI 46 |
| OTOGL | tCcSKEVCGcDTTLc | 1.964468 | TBI(extreme) | TBI 46 |
| OTOGL | tCcSKEVcGCDTTLc | 1.964468 | TBI(extreme) | TBI 46 |
| OTOGL | tCcSKEVCGcDTTLc | 1.964468 | TBI(extreme) | TBI 46 |
| OTOGL | tCcSKEVcGCDTTLc | 1.964468 | TBI(extreme) | TBI 46 |
| OTOGL | tCcSKEVCGcDTTLc | 1.964468 | TBI(extreme) | TBI 46 |
| OTOGL | tCcSKEVcGCDTTLc | 1.964468 | TBI(extreme) | TBI 46 |
| OTOGL | tCcSKEVCGcDTTLc | 1.964468 | TBI(extreme) | TBI 46 |
| OTOGL | tCcSKEVcGCDTTLc | 1.964468 | TBI(extreme) | TBI 46 |
| OTOGL | tCcSKEVCGcDTTLc | 1.964468 | TBI(extreme) | TBI 46 |
| OTOGL | TcCsKEVCGcDTTLc | 1.837791 | TBI(extreme) | TBI 46 |
| OTOGL | TcCsKEVCGcDTTLc | 1.837791 | TBI(extreme) | TBI 46 |
| OTOGL | TcCsKEVCGcDTTLc | 1.837791 | TBI(extreme) | TBI 46 |
| OTOGL | TcCsKEVCGcDTTLc | 1.837791 | TBI(extreme) | TBI 46 |
| OTOGL | TcCsKEVCGcDTTLc | 1.837791 | TBI(extreme) | TBI 46 |
| OTOGL | TcCsKEVCGcDTTLc | 1.837791 | TBI(extreme) | TBI 46 |
| OTOGL | TcCsKEVCGcDTTLc | 1.837791 | TBI(extreme) | TBI 46 |
| OTOGL | TcCsKEVCGcDTTLc | 1.837791 | TBI(extreme) | TBI 46 |
| OTOGL | TcCsKEVCGcDTTLc | 1.837791 | TBI(extreme) | TBI 46 |
| OTOGL | TcCsKEVCGcDTTLc | 1.837791 | TBI(extreme) | TBI 46 |
| OTOGL | TcCsKEVCGcDTTLc | 1.837791 | TBI(extreme) | TBI 46 |
| OTOGL | TcCsKEVCGcDTTLc | 1.837791 | TBI(extreme) | TBI 46 |
| OTOGL | TcCsKEVCGcDTTLc | 1.837791 | TBI(extreme) | TBI 46 |
| OTOGL | tcCSKEVCGcDTTLc | 1.812211 | TBI(extreme) | TBI 46 |
| OTOGL | tcCSKEVCGcDTTLc | 1.812211 | TBI(extreme) | TBI 46 |
| OTOGL | tcCSKEVCGcDTTLc | 1.812211 | TBI(extreme) | TBI 46 |
| OTOGL | tcCSKEVCGcDTTLc | 1.812211 | TBI(extreme) | TBI 46 |
| OTOGL | tcCSKEVCGcDTTLc | 1.812211 | TBI(extreme) | TBI 46 |
| OTOGL | tcCSKEVCGcDTTLc | 1.812211 | TBI(extreme) | TBI 46 |
| OTOGL | tcCSKEVCGcDTTLc | 1.812211 | TBI(extreme) | TBI 46 |
| OTOGL | tcCSKEVCGcDTTLc | 1.812211 | TBI(extreme) | TBI 46 |
| OTOGL | tcCSKEVCGcDTTLc | 1.812211 | TBI(extreme) | TBI 46 |
| OTOGL | tcCSKEVCGcDTTLc | 1.812211 | TBI(extreme) | TBI 46 |
| OTOGL | tcCSKEVCGcDTTLc | 1.812211 | TBI(extreme) | TBI 46 |
| OTOGL | tcCSKEVCGcDTTLc | 1.812211 | TBI(extreme) | TBI 46 |
| OTOGL | tcCSKEVCGcDTTLc | 1.812211 | TBI(extreme) | TBI 46 |
| OTOGL | TCcsKEVCGcDTTLc | 1.782788 | TBI(extreme) | TBI 46 |
| OTOGL | TCcsKEVCGcDTTLc | 1.782788 | TBI(extreme) | TBI 46 |
| OTOGL | TCcsKEVCGcDTTLc | 1.782788 | TBI(extreme) | TBI 46 |
| OTOGL | TCcsKEVCGcDTTLc | 1.782788 | TBI(extreme) | TBI 46 |
| OTOGL | TCcsKEVCGcDTTLc | 1.782788 | TBI(extreme) | TBI 46 |
| OTOGL | TCcsKEVCGcDTTLc | 1.782788 | TBI(extreme) | TBI 46 |
| OTOGL | TCcsKEVCGcDTTLc | 1.782788 | TBI(extreme) | TBI 46 |
| OTOGL | TCcsKEVCGcDTTLc | 1.782788 | TBI(extreme) | TBI 46 |
| OTOGL | TCcsKEVCGcDTTLc | 1.782788 | TBI(extreme) | TBI 46 |
| OTOGL | TCcsKEVCGcDTTLc | 1.782788 | TBI(extreme) | TBI 46 |
| OTOGL | TCcsKEVCGcDTTLc | 1.782788 | TBI(extreme) | TBI 46 |
| OTOGL | TCcsKEVCGcDTTLc | 1.782788 | TBI(extreme) | TBI 46 |
| OTOGL | TCcsKEVCGcDTTLc | 1.782788 | TBI(extreme) | TBI 46 |
| OTOGL | tCcSKEVcGCDTTLc | 1.746905 | TBI(extreme) | TBI 46 |
| OTOGL | tCcSKEVcGCDTTLc | 1.746905 | TBI(extreme) | TBI 46 |
| OTOGL | tCcSKEVcGCDTTLc | 1.746905 | TBI(extreme) | TBI 46 |
| OTOGL | tCcSKEVcGCDTTLc | 1.746905 | TBI(extreme) | TBI 46 |
| OTOGL | tCcSKEVcGCDTTLc | 1.746905 | TBI(extreme) | TBI 46 |
| OTOGL | tCcSKEVcGCDTTLc | 1.746905 | TBI(extreme) | TBI 46 |
| OTOGL | tCcSKEVcGCDTTLc | 1.746905 | TBI(extreme) | TBI 46 |
| OTOGL | tCcSKEVcGCDTTLc | 1.746905 | TBI(extreme) | TBI 46 |
| OTOGL | tCcSKEVcGCDTTLc | 1.746905 | TBI(extreme) | TBI 46 |
| OTOGL | tCcSKEVcGCDTTLc | 1.746905 | TBI(extreme) | TBI 46 |
| OTOGL | tCcSKEVcGCDTTLc | 1.746905 | TBI(extreme) | TBI 46 |
| OTOGL | tCcSKEVcGCDTTLc | 1.746905 | TBI(extreme) | TBI 46 |
| OTOGL | tCcSKEVcGCDTTLc | 1.746905 | TBI(extreme) | TBI 46 |
| OTOGL | QSDcGcIQyLcEKD | 1.852396 | Control(minimal) | control 11 |
| OTOGL | QSDcGcIQyLcEKD | 1.852396 | Control(minimal) | control 11 |
| OTOGL | QSDcGcIQyLcEKD | 1.852396 | Control(minimal) | control 11 |
| OTOGL | QSDcGcIQyLcEKD | 1.852396 | Control(minimal) | control 11 |
| OTOGL | QSDcGcIQyLcEKD | 1.852396 | Control(minimal) | control 11 |
| OTOGL | QSDcGcIQyLcEKD | 1.852396 | Control(minimal) | control 11 |
| OTOGL | QSDcGcIQyLcEKD | 1.852396 | Control(minimal) | control 11 |
| OTOGL | QSDcGcIQyLcEKD | 1.852396 | Control(minimal) | control 11 |
| OTOGL | QSDcGcIQyLcEKD | 1.852396 | Control(minimal) | control 11 |
| OTOGL | QSDcGcIQyLcEKD | 1.852396 | Control(minimal) | control 11 |
| OTOGL | QSDcGcIQyLcEKD | 1.852396 | Control(minimal) | control 11 |
| OTOGL | QSDcGcIQyLcEKD | 1.852396 | Control(minimal) | control 11 |
| OTOGL | QSDcGcIQyLcEKD | 1.852396 | Control(minimal) | control 11 |
| OTOGL | QSDcGcIQyLcEKD | 1.852396 | Control(minimal) | control 11 |
| OTOGL | QSDcGcIQyLcEKD | 1.852396 | Control(minimal) | control 11 |
| OTOGL | QSDcGcIQyLcEKD | 1.852396 | Control(minimal) | control 11 |
| OTOGL | QSDcGcIQyLcEKD | 1.852396 | Control(minimal) | control 11 |
| OTOGL | QSDcGcIQyLcEKD | 1.852396 | Control(minimal) | control 11 |
| OTOGL | QSDcGcIQyLcEKD | 1.852396 | Control(minimal) | control 11 |
| OTOGL | QSDcGcIQyLcEKD | 1.852396 | Control(minimal) | control 11 |
| OTOGL | QSDcGcIQyLcEKD | 1.852396 | Control(minimal) | control 11 |
| OTOGL | QSDcGcIQyLcEKD | 1.852396 | Control(minimal) | control 11 |
| OTOGL | QSDcGcIQyLcEKD | 1.852396 | Control(minimal) | control 11 |
| OTOGL | QSDcGcIQyLcEKD | 1.852396 | Control(minimal) | control 11 |
| OTOGL | QSDcGcIQyLcEKD | 1.852396 | Control(minimal) | control 11 |
| OTOGL | QSDcGcIQyLcEKD | 1.810058 | Control(minimal) | control 11 |
| OTOGL | QSDcGcIQyLcEKD | 1.810058 | Control(minimal) | control 11 |
| OTOGL | QSDcGcIQyLcEKD | 1.810058 | Control(minimal) | control 11 |
| OTOGL | QSDcGcIQyLcEKD | 1.810058 | Control(minimal) | control 11 |
| OTOGL | QSDcGcIQyLcEKD | 1.810058 | Control(minimal) | control 11 |
| OTOGL | QSDcGcIQyLcEKD | 1.810058 | Control(minimal) | control 11 |
| OTOGL | QSDcGcIQyLcEKD | 1.810058 | Control(minimal) | control 11 |
| OTOGL | QSDcGcIQyLcEKD | 1.810058 | Control(minimal) | control 11 |
| OTOGL | QSDcGcIQyLcEKD | 1.810058 | Control(minimal) | control 11 |
| OTOGL | QSDcGcIQyLcEKD | 1.810058 | Control(minimal) | control 11 |
| OTOGL | QSDcGcIQyLcEKD | 1.810058 | Control(minimal) | control 11 |
| OTOGL | QSDcGcIQyLcEKD | 1.810058 | Control(minimal) | control 11 |
| OTOGL | QSDcGcIQyLcEKD | 1.810058 | Control(minimal) | control 11 |
| PAMR1 | GGDcMPcsSSPCFHDGT | 1.997824 | TBI(extreme) | TBI 8 |
| PAMR1 | GGDcMPcsSSPCFHDGT | 1.997824 | TBI(extreme) | TBI 8 |
| PAMR1 | GGDcMPcsSSPCFHDGT | 1.997824 | TBI(extreme) | TBI 8 |
| PAMR1 | GGDcmPCsSSPCFHDGT | 1.997824 | TBI(extreme) | TBI 8 |
| PAMR1 | GGDcMPcsSSPCFHDGT | 1.997824 | TBI(extreme) | TBI 8 |
| PAMR1 | GGDcmPCSsSPCFHDGT | 1.997824 | TBI(extreme) | TBI 8 |
| PAMR1 | GGDcMPcsSSPCFHDGT | 1.997824 | TBI(extreme) | TBI 8 |
| PAMR1 | GGDcMPcsSSPCFHDGT | 1.997824 | TBI(extreme) | TBI 8 |
| PAMR1 | GGDcMPcsSSPCFHDGT | 1.997824 | TBI(extreme) | TBI 8 |
| PAMR1 | GGDcmPCSsSPCFHDGT | 1.997824 | TBI(extreme) | TBI 8 |
| PAMR1 | GGDcMPcsSSPCFHDGT | 1.997824 | TBI(extreme) | TBI 8 |
| PAMR1 | GGDcmPCSsSPCFHDGT | 1.997824 | TBI(extreme) | TBI 8 |
| PAMR1 | GGDcMPcsSSPCFHDGT | 1.997824 | TBI(extreme) | TBI 8 |
| PAMR1 | GGDcmPCSsSPCFHDGT | 1.997824 | TBI(extreme) | TBI 8 |
| PAMR1 | GGDcMPcsSSPCFHDGT | 1.997824 | TBI(extreme) | TBI 8 |
| PAMR1 | GGDcmPCsSSPCFHDGT | 1.904841 | TBI(extreme) | TBI 8 |
| PAMR1 | GGDcmPCsSSPCFHDGT | 1.904841 | TBI(extreme) | TBI 8 |
| PAMR1 | GGDcmPCsSSPCFHDGT | 1.904841 | TBI(extreme) | TBI 8 |
| PAMR1 | GGDcmPCsSSPCFHDGT | 1.904841 | TBI(extreme) | TBI 8 |
| PAMR1 | GGDcmPCsSSPCFHDGT | 1.904841 | TBI(extreme) | TBI 8 |
| PAMR1 | GGDcmPCsSSPCFHDGT | 1.904841 | TBI(extreme) | TBI 8 |
| PAMR1 | GGDcmPCsSSPCFHDGT | 1.904841 | TBI(extreme) | TBI 8 |
| PAMR1 | GGDcMPcsSSPCFHDGT | 1.870118 | TBI(extreme) | TBI 8 |
| PAMR1 | GGDcMPcsSSPCFHDGT | 1.870118 | TBI(extreme) | TBI 8 |
| PAMR1 | GGDcMPcsSSPCFHDGT | 1.870118 | TBI(extreme) | TBI 8 |
| PAMR1 | GGDcMPcsSSPCFHDGT | 1.870118 | TBI(extreme) | TBI 8 |
| PAMR1 | GGDcMPcsSSPCFHDGT | 1.870118 | TBI(extreme) | TBI 8 |
| PAMR1 | GGDcMPcsSSPCFHDGT | 1.870118 | TBI(extreme) | TBI 8 |
| PAMR1 | GGDcMPcsSSPCFHDGT | 1.870118 | TBI(extreme) | TBI 8 |
| PAMR1 | GGDcmPCSsSPCFHDGT | 1.83934 | TBI(extreme) | TBI 8 |
| PAMR1 | GGDcmPCSsSPCFHDGT | 1.83934 | TBI(extreme) | TBI 8 |
| PAMR1 | GGDcmPCSsSPCFHDGT | 1.83934 | TBI(extreme) | TBI 8 |
| PAMR1 | GGDcmPCSsSPCFHDGT | 1.83934 | TBI(extreme) | TBI 8 |
| PAMR1 | GGDcmPCSsSPCFHDGT | 1.83934 | TBI(extreme) | TBI 8 |
| PAMR1 | GGDcmPCSsSPCFHDGT | 1.83934 | TBI(extreme) | TBI 8 |
| PAMR1 | GGDcmPCSsSPCFHDGT | 1.83934 | TBI(extreme) | TBI 8 |
| PAMR1 | EYtVINEAcPGAEW | 1.776379 | TBI(extreme) | TBI 9 |
| PAMR1 | EYtVINEAcPGAEW | 1.776379 | TBI(extreme) | TBI 9 |
| PAMR1 | EYtVINEAcPGAEW | 1.776379 | TBI(extreme) | TBI 9 |
| PAMR1 | EYtVINEAcPGAEW | 1.776379 | TBI(extreme) | TBI 9 |
| PAMR1 | EYtVINEAcPGAEW | 1.776379 | TBI(extreme) | TBI 9 |
| PAMR1 | EYtVINEAcPGAEW | 1.776379 | TBI(extreme) | TBI 9 |
| PAMR1 | EYtVINEAcPGAEW | 1.776379 | TBI(extreme) | TBI 9 |
| PAMR1 | EYtVINEAcPGAEW | 1.776379 | TBI(extreme) | TBI 9 |
| PAMR1 | EYtVINEAcPGAEW | 1.776379 | TBI(extreme) | TBI 9 |
| PAMR1 | EYtVINEAcPGAEW | 1.776379 | TBI(extreme) | TBI 9 |
| PAMR1 | EYtVINEAcPGAEW | 1.776379 | TBI(extreme) | TBI 9 |
| PAMR1 | EYtVINEAcPGAEW | 1.776379 | TBI(extreme) | TBI 9 |
| PAMR1 | EYtVINEAcPGAEW | 1.776379 | TBI(extreme) | TBI 9 |
| PAMR1 | EYtVINEAcPGAEW | 1.776379 | TBI(extreme) | TBI 9 |
| PAMR1 | EYtVINEAcPGAEW | 1.776379 | TBI(extreme) | TBI 9 |
| PCLO | FNTCTEcQTTVcSLCGF | 1.964455 | TBI(extreme) | TBI 2 |
| PCLO | FNTCTEcQTTVcSLCGF | 1.964455 | TBI(extreme) | TBI 2 |
| PCLO | FNTCTEcQTTVcSLCGF | 1.964455 | TBI(extreme) | TBI 2 |
| PCLO | FNTCTEcQTTVcSLCGF | 1.964455 | TBI(extreme) | TBI 2 |
| PCLO | FNTCTEcQTTVcSLCGF | 1.964455 | TBI(extreme) | TBI 2 |
| PCLO | FNTCTEcQTTVcSLCGF | 1.964455 | TBI(extreme) | TBI 2 |
| PCLO | FNTCTEcQTTVcSLCGF | 1.964455 | TBI(extreme) | TBI 2 |
| PCLO | FNTCTEcQTTVcSLCGF | 1.964455 | TBI(extreme) | TBI 2 |
| PCLO | FLPFSNLcQcLIKTN | 1.832901 | TBI(extreme) | TBI 9 |
| PCLO | FLPFSNLcQcLIKTN | 1.832901 | TBI(extreme) | TBI 9 |
| PCLO | FLPFSNLcQcLIKTN | 1.832901 | TBI(extreme) | TBI 9 |
| PCLO | FLPFSNLcQcLIKTN | 1.832901 | TBI(extreme) | TBI 9 |
| PCLO | FLPFSNLcQcLIKTN | 1.832901 | TBI(extreme) | TBI 9 |
| PCLO | TCtECQTTVcSLCGF | 1.782583 | TBI(extreme) | TBI 10 |
| PCLO | TCtECQTTVcSLCGF | 1.782583 | TBI(extreme) | TBI 10 |
| PCLO | TCtECQTTVcSLCGF | 1.782583 | TBI(extreme) | TBI 10 |
| PCLO | TCtECQTTVcSLCGF | 1.782583 | TBI(extreme) | TBI 10 |
| PCLO | TCtECQTTVcSLCGF | 1.782583 | TBI(extreme) | TBI 10 |
| PCLO | TCtECQTTVcSLCGF | 1.782583 | TBI(extreme) | TBI 10 |
| PCLO | TCtECQTTVcSLCGF | 1.782583 | TBI(extreme) | TBI 10 |
| PCLO | TCtECQTTVcSLCGF | 1.782583 | TBI(extreme) | TBI 10 |
| PCLO | TCtECQTTVcSLCGF | 1.782583 | TBI(extreme) | TBI 10 |
| PCLO | TCtECQTTVcSLCGF | 1.782583 | TBI(extreme) | TBI 10 |
| PCLO | TCtECQTTVcSLCGF | 1.782583 | TBI(extreme) | TBI 10 |
| PCLO | TCtECQTTVcSLCGF | 1.782583 | TBI(extreme) | TBI 10 |
| PCLO | TCtECQTTVcSLCGF | 1.782583 | TBI(extreme) | TBI 10 |
| PCLO | TCtECQTTVcSLCGF | 1.782583 | TBI(extreme) | TBI 10 |
| PCLO | TCtECQTTVcSLCGF | 1.782583 | TBI(extreme) | TBI 10 |
| PCLO | LPNPPPEEISTGTQsT | 3.599046 | TBI(extreme) | TBI 30 |
| PCLO | LPNPPPEEISTGtQST | 3.599046 | TBI(extreme) | TBI 30 |
| PCLO | LPNPPPEEIStGTQST | 3.599046 | TBI(extreme) | TBI 30 |
| PCLO | PPTGEKVsPFDSKAIP | 3.599046 | TBI(extreme) | TBI 30 |
| PCLO | PPTGEKVsPFDSKAIP | 2.001242 | TBI(extreme) | TBI 30 |
| PCLO | PPTGEKVsPFDSKAIP | 2.001242 | TBI(extreme) | TBI 30 |
| PCLO | PPTGEKVsPFDSKAIP | 2.001242 | TBI(extreme) | TBI 30 |
| PCLO | PPTGEKVsPFDSKAIP | 2.001242 | TBI(extreme) | TBI 30 |
| PCLO | PPTGEKVsPFDSKAIP | 2.001242 | TBI(extreme) | TBI 30 |
| PCLO | KQDRHGEAGILNPIME | 2.039625 | Control(minimal) | control 6 |
| PCLO | KQDRHGEAGILNPIME | 2.039625 | Control(minimal) | control 6 |
| PCLO | KQDRHGEAGILNPIME | 2.039625 | Control(minimal) | control 6 |
| PCLO | KQDRHGEAGILNPIME | 2.039625 | Control(minimal) | control 6 |
| PFN2 | NLMcDGCcQEAAIVGyC | 1.878575 | Control(minimal) | control 9 |
| PFN2 | NLMcDGCcQEAAIVGyC | 1.878575 | Control(minimal) | control 9 |
| PFN2 | NLMcDGCcQEAAIVGyC | 1.878575 | Control(minimal) | control 9 |
| PFN2 | NLMcDGCcQEAAIVGyC | 1.878575 | Control(minimal) | control 9 |
| PFN2 | NLMcDGCcQEAAIVGyC | 1.878575 | Control(minimal) | control 9 |
| PFN2 | NLMcDGCcQEAAIVGyC | 1.878575 | Control(minimal) | control 9 |
| PFN2 | NLMcDGCcQEAAIVGyC | 1.878575 | Control(minimal) | control 9 |
| PFN2 | NLMcDGCcQEAAIVGyC | 1.878575 | Control(minimal) | control 9 |
| PFN2 | NLmCDGCcQEAAIVGyC | 1.878575 | Control(minimal) | control 9 |
| PFN2 | NLmCDGCcQEAAIVGyC | 1.878575 | Control(minimal) | control 9 |
| PFN2 | NLmCDGCcQEAAIVGyC | 1.878575 | Control(minimal) | control 9 |
| PFN2 | NLmCDGCcQEAAIVGyC | 1.878575 | Control(minimal) | control 9 |
| PFN2 | NLmCDGCcQEAAIVGyC | 1.878575 | Control(minimal) | control 9 |
| PFN2 | NLmCDGCcQEAAIVGyC | 1.878575 | Control(minimal) | control 9 |
| PFN2 | NLmCDGCcQEAAIVGyC | 1.878575 | Control(minimal) | control 9 |
| PFN2 | NLmCDGCcQEAAIVGyC | 1.836107 | Control(minimal) | control 9 |
| PFN2 | NLMcDGCcQEAAIVGyC | 1.878575 | Control(minimal) | control 11 |
| PFN2 | NLMcDGCcQEAAIVGyC | 1.878575 | Control(minimal) | control 11 |
| PFN2 | NLMcDGCcQEAAIVGyC | 1.878575 | Control(minimal) | control 11 |
| PFN2 | NLMcDGCcQEAAIVGyC | 1.878575 | Control(minimal) | control 11 |
| PFN2 | NLMcDGCcQEAAIVGyC | 1.878575 | Control(minimal) | control 11 |
| PFN2 | NLMcDGCcQEAAIVGyC | 1.878575 | Control(minimal) | control 11 |
| PFN2 | NLMcDGCcQEAAIVGyC | 1.878575 | Control(minimal) | control 11 |
| PFN2 | NLMcDGCcQEAAIVGyC | 1.878575 | Control(minimal) | control 11 |
| PFN2 | NLmCDGCcQEAAIVGyC | 1.878575 | Control(minimal) | control 11 |
| PFN2 | NLmCDGCcQEAAIVGyC | 1.878575 | Control(minimal) | control 11 |
| PFN2 | NLmCDGCcQEAAIVGyC | 1.878575 | Control(minimal) | control 11 |
| PFN2 | NLmCDGCcQEAAIVGyC | 1.878575 | Control(minimal) | control 11 |
| PFN2 | NLmCDGCcQEAAIVGyC | 1.878575 | Control(minimal) | control 11 |
| PFN2 | NLmCDGCcQEAAIVGyC | 1.878575 | Control(minimal) | control 11 |
| PFN2 | NLmCDGCcQEAAIVGyC | 1.878575 | Control(minimal) | control 11 |
| PFN2 | NLmCDGCcQEAAIVGyC | 1.836107 | Control(minimal) | control 11 |
| PKD1 | PSSASFACLSLcSGPPPPPA | 1.807033 | TBI(extreme) | TBI 6 |
| PKD1 | PSSASFACLSLcSGPPPPPA | 1.807033 | TBI(extreme) | TBI 6 |
| PKD1 | PSSASFACLSLcSGPPPPPA | 1.807033 | TBI(extreme) | TBI 6 |
| PKD1 | PSSASFACLSLcSGPPPPPA | 1.807033 | TBI(extreme) | TBI 6 |
| PKD1 | PSSASFACLSLcSGPPPPPA | 1.807033 | TBI(extreme) | TBI 6 |
| PKD1 | VtVERmNRMQGLQ | 2.059971 | TBI(extreme) | TBI 46 |
| PKD1 | VtVERmNRMQGLQ | 2.059971 | TBI(extreme) | TBI 46 |
| PKD1 | VtVERmNRMQGLQ | 2.059971 | TBI(extreme) | TBI 46 |
| PKD1 | VtVERmNRMQGLQ | 2.059971 | TBI(extreme) | TBI 46 |
| PKD1 | VtVERmNRMQGLQ | 2.059971 | TBI(extreme) | TBI 46 |
| PKD1 | VtVERmNRMQGLQ | 2.059971 | TBI(extreme) | TBI 46 |
| PKD1 | KLsRTGLVGLGLScLAGQ | 2.010669 | TBI(extreme) | TBI 47 |
| PKD1 | KLsRTGLVGLGLScLAGQ | 2.010669 | TBI(extreme) | TBI 47 |
| PKD1 | KLsRTGLVGLGLScLAGQ | 2.010669 | TBI(extreme) | TBI 47 |
| PKD1 | KLsRTGLVGLGLScLAGQ | 2.010669 | TBI(extreme) | TBI 47 |
| PKD1 | KLsRTGLVGLGLScLAGQ | 2.010669 | TBI(extreme) | TBI 47 |
| POLA1 | DMEPSLGGKASFVc | 1.963651 | Control(minimal) | control 10 |
| POLA1 | DMEPSLGGKASFVc | 1.963651 | Control(minimal) | control 10 |
| POLA1 | DMEPSLGGKASFVc | 2.198664 | Control(minimal) | control 12 |
| POLA1 | DMEPSLGGKASFVc | 2.198664 | Control(minimal) | control 12 |
| POLA1 | DMEPSLGGKASFVc | 2.198664 | Control(minimal) | control 12 |
| POLA1 | DMEPSLGGKASFVc | 2.125626 | Control(minimal) | control 13 |
| POLA1 | DMEPSLGGKASFVc | 2.125626 | Control(minimal) | control 13 |
| POLA1 | DMEPSLGGKASFVc | 2.125626 | Control(minimal) | control 13 |
| PPP1R21 | YEDQLSmmSDHLcSm | 1.892587 | TBI(extreme) | TBI 1 |
| PPP1R21 | YEDQLSmmSDHLcSm | 1.892587 | TBI(extreme) | TBI 1 |
| PPP1R21 | YEDQLSmmSDHLcSm | 1.892587 | TBI(extreme) | TBI 1 |
| PPP1R21 | YEDQLSmmSDHLcSm | 1.892587 | TBI(extreme) | TBI 1 |
| PPP1R21 | YEDQLSmmSDHLcSm | 1.892587 | TBI(extreme) | TBI 1 |
| PPP1R21 | YEDQLSmmSDHLcSm | 1.892587 | TBI(extreme) | TBI 1 |
| PPP1R21 | YEDQLSmmSDHLcSm | 1.892587 | TBI(extreme) | TBI 1 |
| PPP1R21 | YEDQLSmmSDHLcSm | 1.892587 | TBI(extreme) | TBI 1 |
| PPP1R21 | YEDQLSmmSDHLcSm | 1.892587 | TBI(extreme) | TBI 8 |
| PPP1R21 | YEDQLSmmSDHLcSm | 1.892587 | TBI(extreme) | TBI 8 |
| PPP1R21 | YEDQLSmmSDHLcSm | 1.892587 | TBI(extreme) | TBI 8 |
| PPP1R21 | YEDQLSmmSDHLcSm | 1.892587 | TBI(extreme) | TBI 8 |
| PPP1R21 | YEDQLSmmSDHLcSm | 1.892587 | TBI(extreme) | TBI 8 |
| PPP1R21 | YEDQLSmmSDHLcSm | 1.892587 | TBI(extreme) | TBI 8 |
| PPP1R21 | YEDQLSmmSDHLcSm | 1.892587 | TBI(extreme) | TBI 8 |
| PPP1R21 | YEDQLSmmSDHLcSm | 1.892587 | TBI(extreme) | TBI 8 |
| PRUNE2 | YHYVMENLFLyVIS | 1.709178 | Control(minimal) | control 8 |
| PRUNE2 | YHYVMENLFLyVIS | 1.709178 | Control(minimal) | control 8 |
| PRUNE2 | YHYVMENLFLyVIS | 1.709178 | Control(minimal) | control 8 |
| PRUNE2 | YHYVMENLFLyVIS | 1.709178 | Control(minimal) | control 8 |
| PRUNE2 | YHYVMENLFLyVIS | 1.709178 | Control(minimal) | control 8 |
| PRUNE2 | YHYVMENLFLyVIS | 1.709178 | Control(minimal) | control 8 |
| PRUNE2 | YHYVMENLFLyVIS | 1.709178 | Control(minimal) | control 8 |
| PRUNE2 | YHYVMENLFLyVIS | 1.709178 | Control(minimal) | control 8 |
| PRUNE2 | YHYVMENLFLyVIS | 1.709178 | Control(minimal) | control 8 |
| PRUNE2 | YHYVMENLFLyVIS | 1.709178 | Control(minimal) | control 8 |
| PRUNE2 | YHYVMENLFLyVIS | 1.709178 | Control(minimal) | control 8 |
| PRUNE2 | YHYVMENLFLyVIS | 1.709178 | Control(minimal) | control 8 |
| PRUNE2 | YHYVMENLFLyVIS | 1.709178 | Control(minimal) | control 8 |
| PRUNE2 | YHYVMENLFLyVIS | 1.709178 | Control(minimal) | control 8 |
| PRUNE2 | YHYVMENLFLyVIS | 1.709178 | Control(minimal) | control 8 |
| PRUNE2 | YHYVMENLFLyVIS | 1.709178 | Control(minimal) | control 8 |
| PRUNE2 | YHYVMENLFLyVIS | 1.709178 | Control(minimal) | control 8 |
| PRUNE2 | YHYVMENLFLyVIS | 1.709178 | Control(minimal) | control 8 |
| PRUNE2 | YHYVMENLFLyVIS | 1.709178 | Control(minimal) | control 8 |
| PRUNE2 | YHYVMENLFLyVIS | 1.709178 | Control(minimal) | control 8 |
| PRUNE2 | YHYVMENLFLyVIS | 1.709178 | Control(minimal) | control 8 |
| PRUNE2 | YHYVMENLFLyVIS | 1.709178 | Control(minimal) | control 8 |
| PRUNE2 | YHYVMENLFLyVIS | 1.709178 | Control(minimal) | control 8 |
| PRUNE2 | PEGITDGGtKMSAL | 1.825924 | Control(minimal) | control 9 |
| PRUNE2 | PEGITDGGtKMSAL | 1.825924 | Control(minimal) | control 9 |
| PRUNE2 | PEGITDGGtKMSAL | 1.825924 | Control(minimal) | control 11 |
| PRUNE2 | PEGITDGGtKMSAL | 1.825924 | Control(minimal) | control 11 |
| PTPN23 | SMLGAMDKRVSEEcAAG | 1.818727 | Control(minimal) | control 10 |
| PTPN23 | SMLGAMDKRVSEEcAAG | 1.818727 | Control(minimal) | control 10 |
| PTPN23 | SMLGAMDKRVSEEcAAG | 1.818727 | Control(minimal) | control 10 |
| PTPN23 | APSSGPPSsSLELLASL | 1.780833 | Control(minimal) | control 13 |
| PTPN23 | APSSGPPSsSLELLASL | 1.780833 | Control(minimal) | control 13 |
| PTPN23 | APSSGPPSsSLELLASL | 1.780833 | Control(minimal) | control 13 |
| PTPN23 | APSSGPPsSSLELLASL | 1.780833 | Control(minimal) | control 13 |
| PTPN23 | APSSGPPSsSLELLASL | 1.780833 | Control(minimal) | control 13 |
| PTPN23 | APSSGPPsSSLELLASL | 1.780833 | Control(minimal) | control 13 |
| PTPN23 | APSSGPPSsSLELLASL | 1.780833 | Control(minimal) | control 13 |
| PTPN23 | APSSGPPsSSLELLASL | 1.780833 | Control(minimal) | control 13 |
| PTPN23 | APSSGPPSsSLELLASL | 1.780833 | Control(minimal) | control 13 |
| PTPN23 | APSSGPPsSSLELLASL | 1.780833 | Control(minimal) | control 13 |
| PTPN23 | APSSGPPSsSLELLASL | 1.780833 | Control(minimal) | control 13 |
| PTPN23 | APSSGPPsSSLELLASL | 1.780833 | Control(minimal) | control 13 |
| PTPN23 | APSSGPPSsSLELLASL | 1.780833 | Control(minimal) | control 13 |
| PTPN23 | APSSGPPsSSLELLASL | 1.780833 | Control(minimal) | control 13 |
| PTPN23 | APSSGPPSsSLELLASL | 1.780833 | Control(minimal) | control 13 |
| PTPN23 | APSSGPPsSSLELLASL | 1.780833 | Control(minimal) | control 13 |
| PTPN23 | APSSGPPSsSLELLASL | 1.780833 | Control(minimal) | control 13 |
| PTPN23 | APSSGPPsSSLELLASL | 1.737942 | Control(minimal) | control 13 |
| PTPN23 | APSSGPPsSSLELLASL | 1.737942 | Control(minimal) | control 13 |
| PTPN23 | APSSGPPsSSLELLASL | 1.737942 | Control(minimal) | control 13 |
| PTPN23 | APSSGPPsSSLELLASL | 1.737942 | Control(minimal) | control 13 |
| PTPN23 | APSSGPPsSSLELLASL | 1.737942 | Control(minimal) | control 13 |
| PTPN23 | APSSGPPsSSLELLASL | 1.737942 | Control(minimal) | control 13 |
| PTPN23 | APSSGPPsSSLELLASL | 1.737942 | Control(minimal) | control 13 |
| PTPN23 | APSSGPPsSSLELLASL | 1.737942 | Control(minimal) | control 13 |
| PTPN23 | APSSGPPsSSLELLASL | 1.737942 | Control(minimal) | control 13 |
| PTPN5 | GSNVSLtLDmcTPGCNE | 1.814706 | TBI(extreme) | TBI 1 |
| PTPN5 | GSNVSLtLDmcTPGCNE | 1.814706 | TBI(extreme) | TBI 1 |
| PTPN5 | GSNVSLtLDmcTPGCNE | 1.814706 | TBI(extreme) | TBI 1 |
| PTPN5 | GSNVSLtLDmcTPGCNE | 1.814706 | TBI(extreme) | TBI 1 |
| PTPN5 | GSNVSLtLDmcTPGCNE | 1.814706 | TBI(extreme) | TBI 1 |
| PTPN5 | GSNVSLtLDmcTPGCNE | 1.814706 | TBI(extreme) | TBI 1 |
| PTPN5 | GSNVSLtLDmcTPGCNE | 1.814706 | TBI(extreme) | TBI 1 |
| PTPN5 | GSNVSLtLDmcTPGCNE | 1.814706 | TBI(extreme) | TBI 1 |
| PTPN5 | GSNVSLtLDmcTPGCNE | 1.814706 | TBI(extreme) | TBI 1 |
| PTPN5 | GSNVSLtLDmcTPGCNE | 1.814706 | TBI(extreme) | TBI 1 |
| PTPN5 | GSNVSLtLDmcTPGCNE | 1.814706 | TBI(extreme) | TBI 1 |
| PTPN5 | GSNVSLtLDmcTPGCNE | 1.814706 | TBI(extreme) | TBI 1 |
| PTPN5 | GSNVSLtLDmcTPGCNE | 1.814706 | TBI(extreme) | TBI 1 |
| PTPN5 | GSNVSLtLDmcTPGCNE | 1.814706 | TBI(extreme) | TBI 1 |
| PTPN5 | GSNVSLtLDmcTPGCNE | 1.814706 | TBI(extreme) | TBI 1 |
| PTPN5 | GSNVSLtLDmcTPGCNE | 1.814706 | TBI(extreme) | TBI 1 |
| PTPN5 | GSNVSLtLDmcTPGCNE | 1.814706 | TBI(extreme) | TBI 1 |
| PTPN5 | GSNVSLtLDmcTPGCNE | 1.814706 | TBI(extreme) | TBI 1 |
| PTPN5 | GSNVSLtLDmcTPGCNE | 1.814706 | TBI(extreme) | TBI 1 |
| PTPN5 | GSNVSLtLDmcTPGCNE | 1.814706 | TBI(extreme) | TBI 1 |
| PTPN5 | GSNVSLtLDmcTPGCNE | 1.814706 | TBI(extreme) | TBI 1 |
| PTPN5 | GSNVSLtLDmcTPGCNE | 1.814706 | TBI(extreme) | TBI 1 |
| PTPN5 | GSNVSLtLDmcTPGCNE | 1.814706 | TBI(extreme) | TBI 1 |
| PTPN5 | GSNVSLtLDmcTPGCNE | 1.814706 | TBI(extreme) | TBI 8 |
| PTPN5 | GSNVSLtLDmcTPGCNE | 1.814706 | TBI(extreme) | TBI 8 |
| PTPN5 | GSNVSLtLDmcTPGCNE | 1.814706 | TBI(extreme) | TBI 8 |
| PTPN5 | GSNVSLtLDmcTPGCNE | 1.814706 | TBI(extreme) | TBI 8 |
| PTPN5 | GSNVSLtLDmcTPGCNE | 1.814706 | TBI(extreme) | TBI 8 |
| PTPN5 | GSNVSLtLDmcTPGCNE | 1.814706 | TBI(extreme) | TBI 8 |
| PTPN5 | GSNVSLtLDmcTPGCNE | 1.814706 | TBI(extreme) | TBI 8 |
| PTPN5 | GSNVSLtLDmcTPGCNE | 1.814706 | TBI(extreme) | TBI 8 |
| PTPN5 | GSNVSLtLDmcTPGCNE | 1.814706 | TBI(extreme) | TBI 8 |
| PTPN5 | GSNVSLtLDmcTPGCNE | 1.814706 | TBI(extreme) | TBI 8 |
| PTPN5 | GSNVSLtLDmcTPGCNE | 1.814706 | TBI(extreme) | TBI 8 |
| PTPN5 | GSNVSLtLDmcTPGCNE | 1.814706 | TBI(extreme) | TBI 8 |
| PTPN5 | GSNVSLtLDmcTPGCNE | 1.814706 | TBI(extreme) | TBI 8 |
| PTPN5 | GSNVSLtLDmcTPGCNE | 1.814706 | TBI(extreme) | TBI 8 |
| PTPN5 | GSNVSLtLDmcTPGCNE | 1.814706 | TBI(extreme) | TBI 8 |
| PTPN5 | GSNVSLtLDmcTPGCNE | 1.814706 | TBI(extreme) | TBI 8 |
| PTPN5 | GSNVSLtLDmcTPGCNE | 1.814706 | TBI(extreme) | TBI 8 |
| PTPN5 | GSNVSLtLDmcTPGCNE | 1.814706 | TBI(extreme) | TBI 8 |
| PTPN5 | GSNVSLtLDmcTPGCNE | 1.814706 | TBI(extreme) | TBI 8 |
| PTPN5 | GSNVSLtLDmcTPGCNE | 1.814706 | TBI(extreme) | TBI 8 |
| PTPN5 | GSNVSLtLDmcTPGCNE | 1.814706 | TBI(extreme) | TBI 8 |
| PTPN5 | GSNVSLtLDmcTPGCNE | 1.814706 | TBI(extreme) | TBI 8 |
| PTPN5 | GSNVSLtLDmcTPGCNE | 1.814706 | TBI(extreme) | TBI 8 |
| PTPRM | tVVHcLNGGGRSGTFcAI | 1.851375 | TBI(extreme) | TBI 1 |
| PTPRM | tVVHcLNGGGRSGTFcAI | 1.851375 | TBI(extreme) | TBI 1 |
| PTPRM | tVVHcLNGGGRSGTFcAI | 1.851375 | TBI(extreme) | TBI 1 |
| PTPRM | tVVHcLNGGGRSGTFcAI | 1.851375 | TBI(extreme) | TBI 1 |
| PTPRM | tVVHcLNGGGRSGTFcAI | 1.851375 | TBI(extreme) | TBI 1 |
| PTPRM | tVVHcLNGGGRSGTFcAI | 1.851375 | TBI(extreme) | TBI 1 |
| PTPRM | KSPPsAGPLVVHCGAGAGRT | 1.736899 | TBI(extreme) | TBI 5 |
| PTPRM | KSPPsAGPLVVHCGAGAGRT | 1.736899 | TBI(extreme) | TBI 5 |
| PTPRM | KSPPsAGPLVVHCGAGAGRT | 1.736899 | TBI(extreme) | TBI 5 |
| PTPRM | tVVHcLNGGGRSGTFcAI | 1.851375 | TBI(extreme) | TBI 8 |
| PTPRM | tVVHcLNGGGRSGTFcAI | 1.851375 | TBI(extreme) | TBI 8 |
| PTPRM | tVVHcLNGGGRSGTFcAI | 1.851375 | TBI(extreme) | TBI 8 |
| PTPRM | tVVHcLNGGGRSGTFcAI | 1.851375 | TBI(extreme) | TBI 8 |
| PTPRM | tVVHcLNGGGRSGTFcAI | 1.851375 | TBI(extreme) | TBI 8 |
| PTPRM | tVVHcLNGGGRSGTFcAI | 1.851375 | TBI(extreme) | TBI 8 |
| RBM15 | PPSAsVVGASVGGHRHP | 2.442563 | TBI(extreme) | TBI 2 |
| RBM15 | PPSAsVVGASVGGHRHP | 2.442563 | TBI(extreme) | TBI 2 |
| RBM15 | PPSAsVVGASVGGHRHP | 2.442563 | TBI(extreme) | TBI 2 |
| RBM15 | PPSAsVVGASVGGHRHP | 2.442563 | TBI(extreme) | TBI 2 |
| RBM15 | PPSAsVVGASVGGHRHP | 2.442563 | TBI(extreme) | TBI 2 |
| RBM15 | PPSAsVVGASVGGHRHP | 2.442563 | TBI(extreme) | TBI 2 |
| RBM15 | PPSAsVVGASVGGHRHP | 2.442563 | TBI(extreme) | TBI 2 |
| RBM15 | PPsASVVGASVGGHRHP | 2.442563 | TBI(extreme) | TBI 2 |
| RBM15 | PPsASVVGASVGGHRHP | 2.442563 | TBI(extreme) | TBI 2 |
| RBM15 | PPsASVVGASVGGHRHP | 2.442563 | TBI(extreme) | TBI 2 |
| RBM15 | PPsASVVGASVGGHRHP | 2.442563 | TBI(extreme) | TBI 2 |
| RBM15 | PPsASVVGASVGGHRHP | 2.442563 | TBI(extreme) | TBI 2 |
| RBM15 | PPsASVVGASVGGHRHP | 2.442563 | TBI(extreme) | TBI 2 |
| RBM15 | PPSAsVVGASVGGHRHP | 2.442563 | TBI(extreme) | TBI 2 |
| RBM15 | PPsASVVGASVGGHRHP | 2.442563 | TBI(extreme) | TBI 2 |
| RBM15 | PPsASVVGASVGGHRHP | 2.167511 | TBI(extreme) | TBI 2 |
| RBM15 | PPsASVVGASVGGHRHP | 2.167511 | TBI(extreme) | TBI 2 |
| RBM15 | PPsASVVGASVGGHRHP | 2.167511 | TBI(extreme) | TBI 2 |
| RBM15 | PPLLyRDRDRDLYP | 1.820309 | TBI(extreme) | TBI 9 |
| RBM15 | PPLLyRDRDRDLYP | 1.820309 | TBI(extreme) | TBI 9 |
| RBM15 | PPLLyRDRDRDLYP | 1.820309 | TBI(extreme) | TBI 9 |
| RBM15 | PPLLyRDRDRDLYP | 1.820309 | TBI(extreme) | TBI 9 |
| RBM15 | PPLLyRDRDRDLYP | 1.820309 | TBI(extreme) | TBI 9 |
| RBM15 | PPLLyRDRDRDLYP | 1.820309 | TBI(extreme) | TBI 9 |
| RBM15 | PPLLyRDRDRDLYP | 1.820309 | TBI(extreme) | TBI 9 |
| RBM15 | PPLLyRDRDRDLYP | 1.820309 | TBI(extreme) | TBI 9 |
| REXO1 | QPTPLAAPAEPGsKYS | 1.712955 | Control(minimal) | control 9 |
| REXO1 | QPTPLAAPAEPGsKYS | 1.712955 | Control(minimal) | control 9 |
| REXO1 | QPTPLAAPAEPGsKYS | 1.712955 | Control(minimal) | control 9 |
| REXO1 | QPTPLAAPAEPGsKYS | 1.712955 | Control(minimal) | control 9 |
| REXO1 | QPTPLAAPAEPGsKYS | 1.712955 | Control(minimal) | control 9 |
| REXO1 | QPTPLAAPAEPGsKYS | 1.712955 | Control(minimal) | control 9 |
| REXO1 | QPTPLAAPAEPGsKYS | 1.712955 | Control(minimal) | control 9 |
| REXO1 | QPTPLAAPAEPGsKYS | 1.712955 | Control(minimal) | control 9 |
| REXO1 | QPTPLAAPAEPGsKYS | 1.712955 | Control(minimal) | control 9 |
| REXO1 | NVDGHsSSEDAGAcmH | 1.828038 | Control(minimal) | control 11 |
| REXO1 | NVDGHsSSEDAGAcmH | 1.828038 | Control(minimal) | control 11 |
| REXO1 | NVDGHsSSEDAGAcmH | 1.828038 | Control(minimal) | control 11 |
| REXO1 | NVDGHSsSEDAGAcmH | 1.828038 | Control(minimal) | control 11 |
| REXO1 | NVDGHsSSEDAGAcmH | 1.828038 | Control(minimal) | control 11 |
| REXO1 | NVDGHSsSEDAGAcmH | 1.828038 | Control(minimal) | control 11 |
| REXO1 | NVDGHsSSEDAGAcmH | 1.828038 | Control(minimal) | control 11 |
| REXO1 | NVDGHSsSEDAGAcmH | 1.828038 | Control(minimal) | control 11 |
| REXO1 | NVDGHsSSEDAGAcmH | 1.828038 | Control(minimal) | control 11 |
| REXO1 | NVDGHSsSEDAGAcmH | 1.828038 | Control(minimal) | control 11 |
| REXO1 | NVDGHsSSEDAGAcmH | 1.828038 | Control(minimal) | control 11 |
| REXO1 | NVDGHSsSEDAGAcmH | 1.828038 | Control(minimal) | control 11 |
| REXO1 | NVDGHsSSEDAGAcmH | 1.828038 | Control(minimal) | control 11 |
| REXO1 | NVDGHSsSEDAGAcmH | 1.828038 | Control(minimal) | control 11 |
| REXO1 | NVDGHsSSEDAGAcmH | 1.828038 | Control(minimal) | control 11 |
| REXO1 | NVDGHSsSEDAGAcmH | 1.828038 | Control(minimal) | control 11 |
| REXO1 | NVDGHsSSEDAGAcmH | 1.828038 | Control(minimal) | control 11 |
| REXO1 | NVDGHSsSEDAGAcmH | 1.828038 | Control(minimal) | control 11 |
| REXO1 | NVDGHsSSEDAGAcmH | 1.828038 | Control(minimal) | control 11 |
| REXO1 | NVDGHSsSEDAGAcmH | 1.828038 | Control(minimal) | control 11 |
| REXO1 | NVDGHsSSEDAGAcmH | 1.828038 | Control(minimal) | control 11 |
| REXO1 | NVDGHSsSEDAGAcmH | 1.765848 | Control(minimal) | control 11 |
| REXO1 | NVDGHSsSEDAGAcmH | 1.765848 | Control(minimal) | control 11 |
| REXO1 | NVDGHSsSEDAGAcmH | 1.765848 | Control(minimal) | control 11 |
| REXO1 | NVDGHSsSEDAGAcmH | 1.765848 | Control(minimal) | control 11 |
| REXO1 | NVDGHSsSEDAGAcmH | 1.765848 | Control(minimal) | control 11 |
| REXO1 | NVDGHSsSEDAGAcmH | 1.765848 | Control(minimal) | control 11 |
| REXO1 | NVDGHSsSEDAGAcmH | 1.765848 | Control(minimal) | control 11 |
| REXO1 | NVDGHSsSEDAGAcmH | 1.765848 | Control(minimal) | control 11 |
| REXO1 | NVDGHSsSEDAGAcmH | 1.765848 | Control(minimal) | control 11 |
| REXO1 | NVDGHSsSEDAGAcmH | 1.765848 | Control(minimal) | control 11 |
| REXO1 | NVDGHSsSEDAGAcmH | 1.765848 | Control(minimal) | control 11 |
| RTTN | AKHWtAAIDMFcTC | 1.985583 | Control(minimal) | control 9 |
| RTTN | AKHWtAAIDMFcTC | 1.985583 | Control(minimal) | control 9 |
| RTTN | AKHWtAAIDMFcTC | 1.985583 | Control(minimal) | control 9 |
| RTTN | AKHWtAAIDMFcTC | 1.985583 | Control(minimal) | control 9 |
| RTTN | AKHWtAAIDMFcTC | 1.985583 | Control(minimal) | control 9 |
| RTTN | AKHWtAAIDMFcTC | 1.985583 | Control(minimal) | control 9 |
| RTTN | AKHWtAAIDMFcTC | 1.985583 | Control(minimal) | control 9 |
| RTTN | ISLQLLcVYtANFPNGC | 1.915761 | Control(minimal) | control 12 |
| RTTN | ISLQLLcVYtANFPNGC | 1.915761 | Control(minimal) | control 12 |
| RTTN | ISLQLLcVYtANFPNGC | 1.915761 | Control(minimal) | control 12 |
| RTTN | ISLQLLcVYtANFPNGC | 1.915761 | Control(minimal) | control 12 |
| RTTN | ISLQLLcVYtANFPNGC | 1.915761 | Control(minimal) | control 12 |
| RTTN | ISLQLLcVYtANFPNGC | 1.915761 | Control(minimal) | control 12 |
| RTTN | ISLQLLcVYtANFPNGC | 1.915761 | Control(minimal) | control 12 |
| RTTN | ISLQLLcVYtANFPNGC | 1.915761 | Control(minimal) | control 12 |
| RTTN | ISLQLLcVYtANFPNGC | 1.915761 | Control(minimal) | control 12 |
| RTTN | ISLQLLcVYtANFPNGC | 1.915761 | Control(minimal) | control 12 |
| RTTN | ISLQLLcVYtANFPNGC | 1.915761 | Control(minimal) | control 12 |
| RTTN | ISLQLLcVYtANFPNGC | 1.915761 | Control(minimal) | control 12 |
| RTTN | ISLQLLcVYtANFPNGC | 1.915761 | Control(minimal) | control 12 |
| RTTN | ISLQLLcVYtANFPNGC | 1.915761 | Control(minimal) | control 12 |
| RTTN | ISLQLLcVYtANFPNGC | 1.915761 | Control(minimal) | control 12 |
| RTTN | ISLQLLcVYtANFPNGC | 1.915761 | Control(minimal) | control 12 |
| RTTN | ISLQLLcVYtANFPNGC | 1.915761 | Control(minimal) | control 12 |
| RUBCN | TASSsKSFVSSQSFSH | 1.977545 | Control(minimal) | control 9 |
| RUBCN | TASSsKSFVSSQSFSH | 1.977545 | Control(minimal) | control 9 |
| RUBCN | TASSsKSFVSSQSFSH | 1.977545 | Control(minimal) | control 9 |
| RUBCN | TASSsKSFVSSQSFSH | 1.977545 | Control(minimal) | control 9 |
| RUBCN | TASSSKsFVSSQSFSH | 1.977545 | Control(minimal) | control 9 |
| RUBCN | TASSSKsFVSSQSFSH | 1.977545 | Control(minimal) | control 9 |
| RUBCN | TASSSKsFVSSQSFSH | 1.977545 | Control(minimal) | control 9 |
| RUBCN | TASSSKsFVSSQSFSH | 1.943992 | Control(minimal) | control 9 |
| RUBCN | IAAIELMKcNMmSQCL | 1.994085 | Control(minimal) | control 12 |
| RUBCN | IAAIELMKcNMmSQCL | 1.994085 | Control(minimal) | control 12 |
| RUBCN | IAAIELMKcNMmSQCL | 1.994085 | Control(minimal) | control 12 |
| RUBCN | IAAIELMKcNMmSQCL | 1.994085 | Control(minimal) | control 12 |
| RUBCN | IAAIELMKcNmMSQCL | 1.994085 | Control(minimal) | control 12 |
| RUBCN | IAAIELMKcNmMSQCL | 1.994085 | Control(minimal) | control 12 |
| RUBCN | IAAIELMKcNmMSQCL | 1.994085 | Control(minimal) | control 12 |
| RUBCN | IAAIELmKCNMmSQCL | 1.994085 | Control(minimal) | control 12 |
| RUBCN | IAAIELMKcNMmSQCL | 1.994085 | Control(minimal) | control 12 |
| RUBCN | IAAIELmKCNMmSQCL | 1.994085 | Control(minimal) | control 12 |
| RUBCN | IAAIELMKcNMmSQCL | 1.994085 | Control(minimal) | control 12 |
| RUBCN | IAAIELmKCNMmSQCL | 1.994085 | Control(minimal) | control 12 |
| RUBCN | IAAIELMKcNMmSQCL | 1.994085 | Control(minimal) | control 12 |
| RUBCN | IAAIELMKcNmMSQCL | 1.932351 | Control(minimal) | control 12 |
| RUBCN | IAAIELMKcNmMSQCL | 1.932351 | Control(minimal) | control 12 |
| RUBCN | IAAIELMKcNmMSQCL | 1.932351 | Control(minimal) | control 12 |
| RUBCN | IAAIELMKcNmMSQCL | 1.932351 | Control(minimal) | control 12 |
| RUBCN | IAAIELmKCNMmSQCL | 1.926881 | Control(minimal) | control 12 |
| RUBCN | IAAIELmKCNMmSQCL | 1.926881 | Control(minimal) | control 12 |
| RUBCN | IAAIELmKCNMmSQCL | 1.926881 | Control(minimal) | control 12 |
| RUBCN | IAAIELmKCNMmSQCL | 1.926881 | Control(minimal) | control 12 |
| SEC16A | QHtSEQEEmRAFPGP | 2.038182 | Control(minimal) | control 6 |
| SEC16A | QHtSEQEEmRAFPGP | 2.038182 | Control(minimal) | control 6 |
| SEC16A | QHTsEQEEmRAFPGP | 2.038182 | Control(minimal) | control 6 |
| SEC16A | QHTsEQEEmRAFPGP | 2.038182 | Control(minimal) | control 6 |
| SEC16A | QHTsEQEEmRAFPGP | 2.038182 | Control(minimal) | control 6 |
| SEC16A | QHtSEQEEmRAFPGP | 2.038182 | Control(minimal) | control 6 |
| SEC16A | QHtSEQEEmRAFPGP | 2.038182 | Control(minimal) | control 6 |
| SEC16A | QHtSEQEEmRAFPGP | 2.038182 | Control(minimal) | control 6 |
| SEC16A | QHtSEQEEmRAFPGP | 2.038182 | Control(minimal) | control 6 |
| SEC16A | QHtSEQEEmRAFPGP | 2.038182 | Control(minimal) | control 6 |
| SEC16A | QHTsEQEEmRAFPGP | 2.038182 | Control(minimal) | control 6 |
| SEC16A | QHtSEQEEmRAFPGP | 2.038182 | Control(minimal) | control 6 |
| SEC16A | QHTsEQEEmRAFPGP | 2.038182 | Control(minimal) | control 6 |
| SEC16A | QHtSEQEEmRAFPGP | 2.038182 | Control(minimal) | control 6 |
| SEC16A | QHTsEQEEmRAFPGP | 2.038182 | Control(minimal) | control 6 |
| SEC16A | QHtSEQEEmRAFPGP | 2.038182 | Control(minimal) | control 6 |
| SEC16A | QHtSEQEEmRAFPGP | 2.038182 | Control(minimal) | control 6 |
| SEC16A | QHtSEQEEmRAFPGP | 2.038182 | Control(minimal) | control 6 |
| SEC16A | QHtSEQEEmRAFPGP | 2.038182 | Control(minimal) | control 6 |
| SEC16A | QHtSEQEEmRAFPGP | 2.038182 | Control(minimal) | control 6 |
| SEC16A | QHTsEQEEmRAFPGP | 2.038182 | Control(minimal) | control 6 |
| SEC16A | QHTsEQEEmRAFPGP | 2.038182 | Control(minimal) | control 6 |
| SEC16A | QHTsEQEEmRAFPGP | 2.038182 | Control(minimal) | control 6 |
| SEC16A | QHTsEQEEmRAFPGP | 2.038182 | Control(minimal) | control 6 |
| SEC16A | QHTsEQEEmRAFPGP | 2.038182 | Control(minimal) | control 6 |
| SEC16A | QHTsEQEEmRAFPGP | 2.038182 | Control(minimal) | control 6 |
| SEC16A | QHTsEQEEmRAFPGP | 1.898736 | Control(minimal) | control 6 |
| SEC16A | QHTsEQEEmRAFPGP | 1.898736 | Control(minimal) | control 6 |
| SEC16A | QHTsEQEEmRAFPGP | 1.898736 | Control(minimal) | control 6 |
| SEC16A | QHTsEQEEmRAFPGP | 1.898736 | Control(minimal) | control 6 |
| SEC16A | QHTsEQEEmRAFPGP | 1.898736 | Control(minimal) | control 6 |
| SEC16A | QHTsEQEEmRAFPGP | 1.898736 | Control(minimal) | control 6 |
| SEC16A | QHTsEQEEmRAFPGP | 1.898736 | Control(minimal) | control 6 |
| SEC16A | QHTsEQEEmRAFPGP | 1.898736 | Control(minimal) | control 6 |
| SEC16A | QHTsEQEEmRAFPGP | 1.898736 | Control(minimal) | control 6 |
| SEC16A | QHTsEQEEmRAFPGP | 1.898736 | Control(minimal) | control 6 |
| SEC16A | PEGYYSsKSGWSSQS | 1.726032 | Control(minimal) | control 12 |
| SEC16A | PEGYYSsKSGWSSQS | 1.726032 | Control(minimal) | control 12 |
| SEC16A | PEGYYSsKSGWSSQS | 1.726032 | Control(minimal) | control 12 |
| SEC16A | PEGYYSsKSGWSSQS | 1.726032 | Control(minimal) | control 12 |
| SEC16A | PEGYYSsKSGWSSQS | 1.726032 | Control(minimal) | control 12 |
| SEC16A | PEGYYSsKSGWSSQS | 1.726032 | Control(minimal) | control 12 |
| SEC16A | PEGYYSsKSGWSSQS | 1.726032 | Control(minimal) | control 12 |
| SEC16A | PEGYYSsKSGWSSQS | 1.726032 | Control(minimal) | control 12 |
| SEC16A | PEGYYSsKSGWSSQS | 1.726032 | Control(minimal) | control 12 |
| SEC16A | PEGYYSsKSGWSSQS | 1.726032 | Control(minimal) | control 12 |
| SEC16A | PEGYYSsKSGWSSQS | 1.726032 | Control(minimal) | control 12 |
| SEC16A | PEGYYSsKSGWSSQS | 1.726032 | Control(minimal) | control 12 |
| SEC16A | PEGYYSsKSGWSSQS | 1.726032 | Control(minimal) | control 12 |
| SEC16A | PEGYYSsKSGWSSQS | 1.726032 | Control(minimal) | control 12 |
| SEC16A | PEGYYSsKSGWSSQS | 1.726032 | Control(minimal) | control 12 |
| SEC16A | PEGYYSsKSGWSSQS | 1.726032 | Control(minimal) | control 12 |
| SEC16A | PEGYYSsKSGWSSQS | 1.726032 | Control(minimal) | control 12 |
| SERINC2 | cSLLSCAScLcGSAPcI | 1.892989 | TBI(extreme) | TBI 1 |
| SERINC2 | cSLLSCAScLcGSAPcI | 1.892989 | TBI(extreme) | TBI 1 |
| SERINC2 | cSLLSCAScLcGSAPcI | 1.892989 | TBI(extreme) | TBI 1 |
| SERINC2 | cSLLSCAScLcGSAPcI | 1.892989 | TBI(extreme) | TBI 1 |
| SERINC2 | cSLLScASCLcGSAPcI | 1.742675 | TBI(extreme) | TBI 5 |
| SERINC2 | cSLLScASCLcGSAPcI | 1.742675 | TBI(extreme) | TBI 5 |
| SERINC2 | cSLLScASCLcGSAPcI | 1.742675 | TBI(extreme) | TBI 5 |
| SERINC2 | cSLLScASCLcGSAPcI | 1.742675 | TBI(extreme) | TBI 5 |
| SERINC2 | cSLLScASCLcGSAPcI | 1.742675 | TBI(extreme) | TBI 5 |
| SERINC2 | cSLLScASCLcGSAPcI | 1.742675 | TBI(extreme) | TBI 5 |
| SERINC2 | cSLLScASCLcGSAPcI | 1.742675 | TBI(extreme) | TBI 5 |
| SERINC2 | cSLLSCAScLcGSAPcI | 1.892989 | TBI(extreme) | TBI 8 |
| SERINC2 | cSLLSCAScLcGSAPcI | 1.892989 | TBI(extreme) | TBI 8 |
| SERINC2 | cSLLSCAScLcGSAPcI | 1.892989 | TBI(extreme) | TBI 8 |
| SERINC2 | cSLLSCAScLcGSAPcI | 1.892989 | TBI(extreme) | TBI 8 |
| SHANK1 | AVPGRSFMAVKsYQA | 1.879208 | TBI(extreme) | TBI 8 |
| SHANK1 | AVPGRSFMAVKsYQA | 1.879208 | TBI(extreme) | TBI 8 |
| SHANK1 | AVPGRSFMAVKsYQA | 1.879208 | TBI(extreme) | TBI 8 |
| SHANK1 | AVPGRSFMAVKSyQA | 1.879208 | TBI(extreme) | TBI 8 |
| SHANK1 | AVPGRSFMAVKsYQA | 1.879208 | TBI(extreme) | TBI 8 |
| SHANK1 | AVPGRSFMAVKSyQA | 1.879208 | TBI(extreme) | TBI 8 |
| SHANK1 | AVPGRSFMAVKsYQA | 1.879208 | TBI(extreme) | TBI 8 |
| SHANK1 | AVPGRSFMAVKSyQA | 1.879208 | TBI(extreme) | TBI 8 |
| SHANK1 | AVPGRSFMAVKsYQA | 1.879208 | TBI(extreme) | TBI 8 |
| SHANK1 | AVPGRSFMAVKSyQA | 1.879208 | TBI(extreme) | TBI 8 |
| SHANK1 | AVPGRSFMAVKsYQA | 1.879208 | TBI(extreme) | TBI 8 |
| SHANK1 | AVPGRSFMAVKSyQA | 1.879208 | TBI(extreme) | TBI 8 |
| SHANK1 | AVPGRSFMAVKsYQA | 1.879208 | TBI(extreme) | TBI 8 |
| SHANK1 | AVPGRSFMAVKSyQA | 1.866868 | TBI(extreme) | TBI 8 |
| SHANK1 | AVPGRSFMAVKSyQA | 1.866868 | TBI(extreme) | TBI 8 |
| SHANK1 | AVPGRSFMAVKSyQA | 1.866868 | TBI(extreme) | TBI 8 |
| SHANK1 | AVPGRSFMAVKSyQA | 1.866868 | TBI(extreme) | TBI 8 |
| SHANK1 | AVPGRSFMAVKSyQA | 1.866868 | TBI(extreme) | TBI 8 |
| SHANK1 | AVPGRSFMAVKSyQA | 1.866868 | TBI(extreme) | TBI 8 |
| SHANK1 | AVPGRSFMAVKSyQA | 1.866868 | TBI(extreme) | TBI 8 |
| SHANK1 | LsPAFAmSPVPGSACTA | 1.894438 | TBI(extreme) | TBI 46 |
| SHANK1 | LsPAFAmSPVPGSACTA | 1.894438 | TBI(extreme) | TBI 46 |
| SHANK1 | LsPAFAmSPVPGSACTA | 1.894438 | TBI(extreme) | TBI 46 |
| SHANK1 | LsPAFAmSPVPGSACTA | 1.894438 | TBI(extreme) | TBI 46 |
| SHANK1 | LsPAFAmSPVPGSACTA | 1.894438 | TBI(extreme) | TBI 46 |
| SHANK1 | LsPAFAmSPVPGSACTA | 1.894438 | TBI(extreme) | TBI 46 |
| SHANK1 | LsPAFAmSPVPGSACTA | 1.894438 | TBI(extreme) | TBI 46 |
| SIMC1 | DVSRPPQALPSLTLStTS | 1.704516 | TBI(extreme) | TBI 1 |
| SIMC1 | DVSRPPQALPSLTLStTS | 1.704516 | TBI(extreme) | TBI 1 |
| SIMC1 | DVSRPPQALPSLTLStTS | 1.704516 | TBI(extreme) | TBI 1 |
| SIMC1 | DVSRPPQALPSLTLsTTS | 1.704516 | TBI(extreme) | TBI 1 |
| SIMC1 | DVSRPPQALPSLTLStTS | 1.704516 | TBI(extreme) | TBI 1 |
| SIMC1 | DVSRPPQALPSLTLStTS | 1.704516 | TBI(extreme) | TBI 8 |
| SIMC1 | DVSRPPQALPSLTLStTS | 1.704516 | TBI(extreme) | TBI 8 |
| SIMC1 | DVSRPPQALPSLTLStTS | 1.704516 | TBI(extreme) | TBI 8 |
| SIMC1 | DVSRPPQALPSLTLsTTS | 1.704516 | TBI(extreme) | TBI 8 |
| SIMC1 | DVSRPPQALPSLTLStTS | 1.704516 | TBI(extreme) | TBI 8 |
| SIMC1 | PTCSSNKIAEMMFGF | 1.720693 | TBI(extreme) | TBI 9 |
| SIMC1 | PTCSSNKIAEMMFGF | 1.720693 | TBI(extreme) | TBI 9 |
| SIMC1 | PTCSSNKIAEMMFGF | 1.720693 | TBI(extreme) | TBI 9 |
| SIMC1 | PTCSSNKIAEMMFGF | 1.720693 | TBI(extreme) | TBI 9 |
| SIMC1 | PTCSSNKIAEMMFGF | 1.720693 | TBI(extreme) | TBI 9 |
| SLC22A18 | VASNCPTGLFMVMFSII | 1.792684 | Control(minimal) | control 9 |
| SLC22A18 | VASNCPTGLFMVMFSII | 1.792684 | Control(minimal) | control 9 |
| SLC22A18 | VASNCPTGLFMVMFSII | 1.792684 | Control(minimal) | control 9 |
| SLC22A18 | VASNCPTGLFMVMFSII | 1.792684 | Control(minimal) | control 9 |
| SLC22A18 | VASNCPTGLFMVMFSII | 1.792684 | Control(minimal) | control 9 |
| SLC22A18 | VASNCPTGLFMVMFSII | 1.792684 | Control(minimal) | control 9 |
| SLC22A18 | VASNCPTGLFMVMFSII | 1.792684 | Control(minimal) | control 9 |
| SLC22A18 | VASNCPTGLFMVMFSII | 1.792684 | Control(minimal) | control 9 |
| SLC22A18 | VASNCPTGLFMVMFSII | 1.792684 | Control(minimal) | control 9 |
| SLC22A18 | VASNCPTGLFMVMFSII | 1.792684 | Control(minimal) | control 9 |
| SLC22A18 | VASNCPTGLFMVMFSII | 1.792684 | Control(minimal) | control 9 |
| SLC22A18 | VASNCPTGLFMVMFSII | 1.792684 | Control(minimal) | control 11 |
| SLC22A18 | VASNCPTGLFMVMFSII | 1.792684 | Control(minimal) | control 11 |
| SLC22A18 | VASNCPTGLFMVMFSII | 1.792684 | Control(minimal) | control 11 |
| SLC22A18 | VASNCPTGLFMVMFSII | 1.792684 | Control(minimal) | control 11 |
| SLC22A18 | VASNCPTGLFMVMFSII | 1.792684 | Control(minimal) | control 11 |
| SLC22A18 | VASNCPTGLFMVMFSII | 1.792684 | Control(minimal) | control 11 |
| SLC22A18 | VASNCPTGLFMVMFSII | 1.792684 | Control(minimal) | control 11 |
| SLC22A18 | VASNCPTGLFMVMFSII | 1.792684 | Control(minimal) | control 11 |
| SLC22A18 | VASNCPTGLFMVMFSII | 1.792684 | Control(minimal) | control 11 |
| SLC22A18 | VASNCPTGLFMVMFSII | 1.792684 | Control(minimal) | control 11 |
| SLC22A18 | VASNCPTGLFMVMFSII | 1.792684 | Control(minimal) | control 11 |
| SON | mVyNNcDLKEGKRK | 1.737534 | Control(minimal) | control 8 |
| SON | mVyNNcDLKEGKRK | 1.737534 | Control(minimal) | control 8 |
| SON | mVyNNcDLKEGKRK | 1.737534 | Control(minimal) | control 8 |
| SON | LATSTMDSQMLAtSSMD | 1.936866 | Control(minimal) | control 11 |
| SON | LATSTMDSQMLAtSSMD | 1.936866 | Control(minimal) | control 11 |
| SON | LATSTMDSQMLAtSSMD | 1.936866 | Control(minimal) | control 11 |
| SON | LATSTMDSQMLAtSSMD | 1.936866 | Control(minimal) | control 11 |
| SON | LATSTMDSQMLAtSSMD | 1.936866 | Control(minimal) | control 11 |
| SON | LATSTMDSQMLAtSSMD | 1.936866 | Control(minimal) | control 11 |
| SON | LATSTMDSQMLAtSSMD | 1.936866 | Control(minimal) | control 11 |
| SON | LATSTMDSQMLAtSSMD | 1.936866 | Control(minimal) | control 11 |
| SON | LATSTMDSQMLAtSSMD | 1.936866 | Control(minimal) | control 11 |
| SON | LATSTMDSQMLAtSSMD | 1.936866 | Control(minimal) | control 11 |
| SON | LATSTMDSQMLAtSSMD | 1.936866 | Control(minimal) | control 11 |
| SON | LATSTMDSQMLAtSSMD | 1.936866 | Control(minimal) | control 11 |
| SON | LATSTMDSQMLATsSMD | 1.936866 | Control(minimal) | control 11 |
| SON | LATSTMDSQMLATsSMD | 1.936866 | Control(minimal) | control 11 |
| SON | LATSTMDSQMLATsSMD | 1.936866 | Control(minimal) | control 11 |
| SON | LATSTMDSQMLATsSMD | 1.936866 | Control(minimal) | control 11 |
| SON | LATSTMDSQMLATsSMD | 1.936866 | Control(minimal) | control 11 |
| SON | LATSTMDSQMLATsSMD | 1.936866 | Control(minimal) | control 11 |
| SON | LATSTMDSQMLATsSMD | 1.936866 | Control(minimal) | control 11 |
| SON | LATSTMDSQMLATsSMD | 1.936866 | Control(minimal) | control 11 |
| SON | LATSTMDSQMLATsSMD | 1.936866 | Control(minimal) | control 11 |
| SON | LATSTMDSQMLATsSMD | 1.936866 | Control(minimal) | control 11 |
| SON | LATSTMDSQMLATsSMD | 1.936866 | Control(minimal) | control 11 |
| SON | LATSTMDSQMLATSsMD | 1.936866 | Control(minimal) | control 11 |
| SON | LATSTMDSQMLAtSSMD | 1.936866 | Control(minimal) | control 11 |
| SON | LATSTMDSQMLATSsMD | 1.936866 | Control(minimal) | control 11 |
| SON | LATSTMDSQMLAtSSMD | 1.936866 | Control(minimal) | control 11 |
| SON | LATSTMDSQMLATSsMD | 1.936866 | Control(minimal) | control 11 |
| SON | LATSTMDSQMLAtSSMD | 1.936866 | Control(minimal) | control 11 |
| SON | LATSTMDSQMLATSsMD | 1.936866 | Control(minimal) | control 11 |
| SON | LATSTMDSQMLAtSSMD | 1.936866 | Control(minimal) | control 11 |
| SON | LATSTMDSQMLATSsMD | 1.936866 | Control(minimal) | control 11 |
| SON | LATSTMDSQMLAtSSMD | 1.936866 | Control(minimal) | control 11 |
| SON | LATSTMDSQMLATSsMD | 1.936866 | Control(minimal) | control 11 |
| SON | LATSTMDSQMLAtSSMD | 1.936866 | Control(minimal) | control 11 |
| SON | LATSTMDSQMLATSsMD | 1.936866 | Control(minimal) | control 11 |
| SON | LATSTMDSQMLAtSSMD | 1.936866 | Control(minimal) | control 11 |
| SON | LATSTMDSQMLATSsMD | 1.936866 | Control(minimal) | control 11 |
| SON | LATSTMDSQMLAtSSMD | 1.936866 | Control(minimal) | control 11 |
| SON | LATSTMDSQMLATSsMD | 1.936866 | Control(minimal) | control 11 |
| SON | LATSTMDSQMLAtSSMD | 1.936866 | Control(minimal) | control 11 |
| SON | LATSTMDSQMLATSsMD | 1.936866 | Control(minimal) | control 11 |
| SON | LATSTMDSQMLAtSSMD | 1.936866 | Control(minimal) | control 11 |
| SON | LATSTMDSQMLATSsMD | 1.936866 | Control(minimal) | control 11 |
| SON | LATSTMDSQMLAtSSMD | 1.936866 | Control(minimal) | control 11 |
| SON | LATSTMDSQMLATsSMD | 1.929158 | Control(minimal) | control 11 |
| SON | LATSTMDSQMLATsSMD | 1.929158 | Control(minimal) | control 11 |
| SON | LATSTMDSQMLATsSMD | 1.929158 | Control(minimal) | control 11 |
| SON | LATSTMDSQMLATsSMD | 1.929158 | Control(minimal) | control 11 |
| SON | LATSTMDSQMLATsSMD | 1.929158 | Control(minimal) | control 11 |
| SON | LATSTMDSQMLATsSMD | 1.929158 | Control(minimal) | control 11 |
| SON | LATSTMDSQMLATsSMD | 1.929158 | Control(minimal) | control 11 |
| SON | LATSTMDSQMLATsSMD | 1.929158 | Control(minimal) | control 11 |
| SON | LATSTMDSQMLATsSMD | 1.929158 | Control(minimal) | control 11 |
| SON | LATSTMDSQMLATsSMD | 1.929158 | Control(minimal) | control 11 |
| SON | LATSTMDSQMLATsSMD | 1.929158 | Control(minimal) | control 11 |
| SON | LATSTMDSQMLATsSMD | 1.929158 | Control(minimal) | control 11 |
| SON | LATSTMDSQMLATSsMD | 1.795986 | Control(minimal) | control 11 |
| SON | LATSTMDSQMLATSsMD | 1.795986 | Control(minimal) | control 11 |
| SON | LATSTMDSQMLATSsMD | 1.795986 | Control(minimal) | control 11 |
| SON | LATSTMDSQMLATSsMD | 1.795986 | Control(minimal) | control 11 |
| SON | LATSTMDSQMLATSsMD | 1.795986 | Control(minimal) | control 11 |
| SON | LATSTMDSQMLATSsMD | 1.795986 | Control(minimal) | control 11 |
| SON | LATSTMDSQMLATSsMD | 1.795986 | Control(minimal) | control 11 |
| SON | LATSTMDSQMLATSsMD | 1.795986 | Control(minimal) | control 11 |
| SON | LATSTMDSQMLATSsMD | 1.795986 | Control(minimal) | control 11 |
| SON | LATSTMDSQMLATSsMD | 1.795986 | Control(minimal) | control 11 |
| SON | LATSTMDSQMLATSsMD | 1.795986 | Control(minimal) | control 11 |
| SON | LATSTMDSQMLATSsMD | 1.795986 | Control(minimal) | control 11 |
| SON | MLASNTMDsQMLASN | 1.737128 | Control(minimal) | control 13 |
| SON | MLASNTMDsQMLASN | 1.737128 | Control(minimal) | control 13 |
| SON | MLASNTMDsQMLASN | 1.737128 | Control(minimal) | control 13 |
| SON | MLASNTMDsQMLASN | 1.737128 | Control(minimal) | control 13 |
| SON | MLASNTMDsQMLASN | 1.737128 | Control(minimal) | control 13 |
| SON | MLASNTMDsQMLASN | 1.737128 | Control(minimal) | control 13 |
| SON | MLASNTMDsQMLASN | 1.737128 | Control(minimal) | control 13 |
| SON | MLASNTMDsQMLASN | 1.737128 | Control(minimal) | control 13 |
| SON | MLASNTMDsQMLASN | 1.737128 | Control(minimal) | control 13 |
| SON | MLASNTMDsQMLASN | 1.737128 | Control(minimal) | control 13 |
| SON | MLASNTMDsQMLASN | 1.737128 | Control(minimal) | control 13 |
| SON | MLASNTMDsQMLASN | 1.737128 | Control(minimal) | control 13 |
| SON | MLASNTMDsQMLASN | 1.737128 | Control(minimal) | control 13 |
| SON | MLASNTMDsQMLASN | 1.737128 | Control(minimal) | control 13 |
| SON | MLASNTMDsQMLASN | 1.737128 | Control(minimal) | control 13 |
| SON | MLASNTMDsQMLASN | 1.737128 | Control(minimal) | control 13 |
| SON | MLASNTMDsQMLASN | 1.737128 | Control(minimal) | control 13 |
| SON | MLASNTMDsQMLASN | 1.737128 | Control(minimal) | control 13 |
| SON | MLASNTMDsQMLASN | 1.737128 | Control(minimal) | control 13 |
| SON | MLASNTMDsQMLASN | 1.737128 | Control(minimal) | control 13 |
| SON | MLASNTMDsQMLASN | 1.737128 | Control(minimal) | control 13 |
| SON | MLASNTMDsQMLASN | 1.737128 | Control(minimal) | control 13 |
| SON | MLASNTMDsQMLASN | 1.737128 | Control(minimal) | control 13 |
| SPATA7 | PRStFP | 2.88266 | TBI(extreme) | TBI 6 |
| SPATA7 | PRStFP | 2.88266 | TBI(extreme) | TBI 6 |
| SPATA7 | PRStFP | 2.88266 | TBI(extreme) | TBI 6 |
| SPATA7 | PRStFP | 2.88266 | TBI(extreme) | TBI 6 |
| SPATA7 | PRStFP | 2.88266 | TBI(extreme) | TBI 6 |
| SPATA7 | PRStFP | 2.88266 | TBI(extreme) | TBI 6 |
| SPATA7 | PRStFP | 2.88266 | TBI(extreme) | TBI 6 |
| SPATA7 | PRStFP | 2.88266 | TBI(extreme) | TBI 6 |
| SPATA7 | PRStFP | 2.88266 | TBI(extreme) | TBI 6 |
| SPATA7 | PRStFP | 2.88266 | TBI(extreme) | TBI 6 |
| SPATA7 | PRStFP | 2.88266 | TBI(extreme) | TBI 6 |
| SPATA7 | TNGPEKNSSSsPSSVDYA | 2.88266 | TBI(extreme) | TBI 6 |
| SPATA7 | TNGPEKNSSSsPSSVDYA | 2.88266 | TBI(extreme) | TBI 6 |
| SPATA7 | TNGPEKNSSSsPSSVDYA | 2.88266 | TBI(extreme) | TBI 6 |
| SPATA7 | TNGPEKNSSSsPSSVDYA | 2.88266 | TBI(extreme) | TBI 6 |
| SPATA7 | TNGPEKNSSSsPSSVDYA | 2.88266 | TBI(extreme) | TBI 6 |
| SPATA7 | TNGPEKNSSSsPSSVDYA | 2.88266 | TBI(extreme) | TBI 6 |
| SPATA7 | TNGPEKNSSSsPSSVDYA | 2.88266 | TBI(extreme) | TBI 6 |
| SPATA7 | TNGPEKNSSSsPSSVDYA | 2.88266 | TBI(extreme) | TBI 6 |
| SPATA7 | TNGPEKNSSSsPSSVDYA | 2.88266 | TBI(extreme) | TBI 6 |
| SPATA7 | TNGPEKNSSSsPSSVDYA | 2.88266 | TBI(extreme) | TBI 6 |
| SPATA7 | TNGPEKNSSSsPSSVDYA | 2.88266 | TBI(extreme) | TBI 6 |
| SPATA7 | TNGPEKNSSSsPSSVDYA | 1.869967 | TBI(extreme) | TBI 6 |
| SPATA7 | TNGPEKNSSSsPSSVDYA | 1.869967 | TBI(extreme) | TBI 6 |
| SPATA7 | TNGPEKNSSSsPSSVDYA | 1.869967 | TBI(extreme) | TBI 6 |
| SPATA7 | TNGPEKNSSSsPSSVDYA | 1.869967 | TBI(extreme) | TBI 6 |
| SPATA7 | TNGPEKNSSSsPSSVDYA | 1.869967 | TBI(extreme) | TBI 6 |
| SPATA7 | TNGPEKNSSSsPSSVDYA | 1.869967 | TBI(extreme) | TBI 6 |
| SPATA7 | TNGPEKNSSSsPSSVDYA | 1.869967 | TBI(extreme) | TBI 6 |
| SPATA7 | TNGPEKNSSSsPSSVDYA | 1.869967 | TBI(extreme) | TBI 6 |
| SPATA7 | TNGPEKNSSSsPSSVDYA | 1.869967 | TBI(extreme) | TBI 6 |
| SPATA7 | TNGPEKNSSSsPSSVDYA | 1.869967 | TBI(extreme) | TBI 6 |
| SPATA7 | TNGPEKNSSSsPSSVDYA | 1.869967 | TBI(extreme) | TBI 6 |
| SPATA7 | TNGPEKNSSSsPSSVDYA | 1.869967 | TBI(extreme) | TBI 6 |
| SPATA7 | SSASEVYLtSGPRRLH | 2.299008 | TBI(extreme) | TBI 30 |
| SPATA7 | SSASEVYLtSGPRRLH | 2.299008 | TBI(extreme) | TBI 30 |
| SPATA7 | SSASEVYLtSGPRRLH | 2.299008 | TBI(extreme) | TBI 30 |
| SPATA7 | SSASEVyLTSGPRRLH | 2.299008 | TBI(extreme) | TBI 30 |
| SPATA7 | SSASEVYLtSGPRRLH | 2.299008 | TBI(extreme) | TBI 30 |
| SPATA7 | SSASEVyLTSGPRRLH | 2.256676 | TBI(extreme) | TBI 30 |
| SPATA7 | SSASEVyLTSGPRRLH | 2.256676 | TBI(extreme) | TBI 30 |
| SPATA7 | SSASEVyLTSGPRRLH | 2.256676 | TBI(extreme) | TBI 30 |
| SPHKAP | SSASSSGLcKSDSCLy | 1.819541 | TBI(extreme) | TBI 9 |
| SPHKAP | SSASSSGLcKSDSCLy | 1.819541 | TBI(extreme) | TBI 9 |
| SPHKAP | SSASSSGLcKSDSCLy | 1.819541 | TBI(extreme) | TBI 9 |
| SPHKAP | SSASSSGLcKSDSCLy | 1.819541 | TBI(extreme) | TBI 9 |
| SPHKAP | SSASSSGLcKSDSCLy | 1.819541 | TBI(extreme) | TBI 9 |
| SPHKAP | SSASSSGLcKSDSCLy | 1.819541 | TBI(extreme) | TBI 9 |
| SPHKAP | SSASSSGLcKSDSCLy | 1.819541 | TBI(extreme) | TBI 9 |
| SPHKAP | VASAVAVcGLGEREEVtC | 1.901729 | Control(minimal) | control 9 |
| SPHKAP | VASAVAVcGLGEREEVtC | 1.901729 | Control(minimal) | control 9 |
| SPHKAP | VASAVAVcGLGEREEVtC | 1.901729 | Control(minimal) | control 9 |
| SPHKAP | VASAVAVcGLGEREEVtC | 1.901729 | Control(minimal) | control 9 |
| SPHKAP | VASAVAVcGLGEREEVtC | 1.901729 | Control(minimal) | control 9 |
| SPHKAP | KGQSESTEAPASGPPTGtAS | 1.824338 | Control(minimal) | control 9 |
| SPHKAP | KGQSESTEAPASGPPTGtAS | 1.824338 | Control(minimal) | control 9 |
| SPHKAP | KGQSESTEAPASGPPTGtAS | 1.824338 | Control(minimal) | control 9 |
| SPHKAP | KGQSESTEAPASGPPTGtAS | 1.824338 | Control(minimal) | control 9 |
| SPHKAP | KGQSESTEAPASGPPtGTAS | 1.824338 | Control(minimal) | control 9 |
| SPHKAP | KGQSESTEAPASGPPTGtAS | 1.824338 | Control(minimal) | control 9 |
| SPHKAP | KGQSESTEAPASGPPtGTAS | 1.824338 | Control(minimal) | control 9 |
| SPHKAP | KGQSESTEAPASGPPTGtAS | 1.824338 | Control(minimal) | control 9 |
| SPHKAP | KGQSESTEAPASGPPTGtAS | 1.824338 | Control(minimal) | control 9 |
| SPHKAP | KGQSESTEAPASGPPTGtAS | 1.824338 | Control(minimal) | control 9 |
| SPHKAP | KGQSESTEAPASGPPtGTAS | 1.824338 | Control(minimal) | control 9 |
| SPHKAP | KGQSESTEAPASGPPTGtAS | 1.824338 | Control(minimal) | control 9 |
| SPHKAP | KGQSESTEAPASGPPtGTAS | 1.824338 | Control(minimal) | control 9 |
| SPHKAP | KGQSESTEAPASGPPtGTAS | 1.716159 | Control(minimal) | control 9 |
| SPHKAP | KGQSESTEAPASGPPtGTAS | 1.716159 | Control(minimal) | control 9 |
| SPHKAP | KGQSESTEAPASGPPtGTAS | 1.716159 | Control(minimal) | control 9 |
| SPHKAP | KGQSESTEAPASGPPtGTAS | 1.716159 | Control(minimal) | control 9 |
| SPHKAP | KGQSESTEAPASGPPtGTAS | 1.716159 | Control(minimal) | control 9 |
| SPHKAP | KGQSESTEAPASGPPTGtAS | 1.824338 | Control(minimal) | control 11 |
| SPHKAP | KGQSESTEAPASGPPTGtAS | 1.824338 | Control(minimal) | control 11 |
| SPHKAP | KGQSESTEAPASGPPTGtAS | 1.824338 | Control(minimal) | control 11 |
| SPHKAP | KGQSESTEAPASGPPTGtAS | 1.824338 | Control(minimal) | control 11 |
| SPHKAP | KGQSESTEAPASGPPtGTAS | 1.824338 | Control(minimal) | control 11 |
| SPHKAP | KGQSESTEAPASGPPTGtAS | 1.824338 | Control(minimal) | control 11 |
| SPHKAP | KGQSESTEAPASGPPtGTAS | 1.824338 | Control(minimal) | control 11 |
| SPHKAP | KGQSESTEAPASGPPTGtAS | 1.824338 | Control(minimal) | control 11 |
| SPHKAP | KGQSESTEAPASGPPTGtAS | 1.824338 | Control(minimal) | control 11 |
| SPHKAP | KGQSESTEAPASGPPTGtAS | 1.824338 | Control(minimal) | control 11 |
| SPHKAP | KGQSESTEAPASGPPtGTAS | 1.824338 | Control(minimal) | control 11 |
| SPHKAP | KGQSESTEAPASGPPTGtAS | 1.824338 | Control(minimal) | control 11 |
| SPHKAP | KGQSESTEAPASGPPtGTAS | 1.824338 | Control(minimal) | control 11 |
| SPHKAP | KGQSESTEAPASGPPtGTAS | 1.716159 | Control(minimal) | control 11 |
| SPHKAP | KGQSESTEAPASGPPtGTAS | 1.716159 | Control(minimal) | control 11 |
| SPHKAP | KGQSESTEAPASGPPtGTAS | 1.716159 | Control(minimal) | control 11 |
| SPHKAP | KGQSESTEAPASGPPtGTAS | 1.716159 | Control(minimal) | control 11 |
| SPHKAP | KGQSESTEAPASGPPtGTAS | 1.716159 | Control(minimal) | control 11 |
| SSPO | NcCRPScSISSccRP | 1.925606 | TBI(extreme) | TBI 10 |
| SSPO | PAMScIPGESSDNcTA | 1.925606 | TBI(extreme) | TBI 10 |
| SSPO | SLPPAcTRAERR | 1.925606 | TBI(extreme) | TBI 10 |
| SSPO | YtcPNGTcIGFQLVCDG | 1.925606 | TBI(extreme) | TBI 10 |
| SSPO | yTcPNGTcIGFQLVCDG | 1.925606 | TBI(extreme) | TBI 10 |
| SSPO | YtcPNGTcIGFQLVCDG | 1.925606 | TBI(extreme) | TBI 10 |
| SSPO | yTcPNGTcIGFQLVCDG | 1.925606 | TBI(extreme) | TBI 10 |
| SSPO | YtcPNGTcIGFQLVCDG | 1.925606 | TBI(extreme) | TBI 10 |
| SSPO | YtcPNGTcIGFQLVCDG | 1.925606 | TBI(extreme) | TBI 10 |
| SSPO | yTcPNGTcIGFQLVCDG | 1.925606 | TBI(extreme) | TBI 10 |
| SSPO | YtcPNGTcIGFQLVCDG | 1.925606 | TBI(extreme) | TBI 10 |
| SSPO | yTcPNGTcIGFQLVCDG | 1.925606 | TBI(extreme) | TBI 10 |
| SSPO | YtcPNGTcIGFQLVCDG | 1.925606 | TBI(extreme) | TBI 10 |
| SSPO | YtcPNGTcIGFQLVCDG | 1.925606 | TBI(extreme) | TBI 10 |
| SSPO | yTcPNGTcIGFQLVCDG | 1.925606 | TBI(extreme) | TBI 10 |
| SSPO | YtcPNGTcIGFQLVCDG | 1.925606 | TBI(extreme) | TBI 10 |
| SSPO | yTcPNGTcIGFQLVCDG | 1.925606 | TBI(extreme) | TBI 10 |
| SSPO | YtcPNGTcIGFQLVCDG | 1.925606 | TBI(extreme) | TBI 10 |
| SSPO | yTcPNGTcIGFQLVCDG | 1.917411 | TBI(extreme) | TBI 10 |
| SSPO | PAMScIPGESSDNcTA | 1.825744 | TBI(extreme) | TBI 10 |
| SSPO | PAMScIPGESSDNcTA | 1.820825 | TBI(extreme) | TBI 10 |
| SSPO | yTcPNGTcIGFQLVCDG | 1.820825 | TBI(extreme) | TBI 10 |
| SSPO | yTcPNGTcIGFQLVCDG | 1.820825 | TBI(extreme) | TBI 10 |
| SSPO | yTcPNGTcIGFQLVCDG | 1.820825 | TBI(extreme) | TBI 10 |
| SSPO | yTcPNGTcIGFQLVCDG | 1.820825 | TBI(extreme) | TBI 10 |
| SSPO | yTcPNGTcIGFQLVCDG | 1.820825 | TBI(extreme) | TBI 10 |
| SSPO | yTcPNGTcIGFQLVCDG | 1.820825 | TBI(extreme) | TBI 10 |
| SSPO | yTcPNGTcIGFQLVCDG | 1.820825 | TBI(extreme) | TBI 10 |
| SSPO | yTcPNGTcIGFQLVCDG | 1.820825 | TBI(extreme) | TBI 10 |
| SSPO | yTcPNGTcIGFQLVCDG | 1.820825 | TBI(extreme) | TBI 10 |
| SSPO | yTcPNGTcIGFQLVCDG | 1.819364 | TBI(extreme) | TBI 10 |
| SSPO | cGcDSGGDcEcL | 3.720609 | TBI(extreme) | TBI 46 |
| SSPO | cGcDSGGDcEcL | 3.720609 | TBI(extreme) | TBI 46 |
| SSPO | cGcDSGGDcEcL | 3.720609 | TBI(extreme) | TBI 46 |
| SSPO | cGcDSGGDcEcL | 3.720609 | TBI(extreme) | TBI 46 |
| SSPO | cGcDSGGDcEcL | 3.720609 | TBI(extreme) | TBI 46 |
| SSPO | cGcDSGGDcEcL | 3.720609 | TBI(extreme) | TBI 46 |
| SSPO | cPAECAVGGDGHy | 3.720609 | TBI(extreme) | TBI 46 |
| SSPO | RLCPSPGDSsCPGDATQ | 3.720609 | TBI(extreme) | TBI 46 |
| SSPO | cGcDSGGDcEcL | 2.191106 | TBI(extreme) | TBI 46 |
| SSPO | cGcDSGGDcEcL | 2.191106 | TBI(extreme) | TBI 46 |
| SSPO | cGcDSGGDcEcL | 2.191106 | TBI(extreme) | TBI 46 |
| SSPO | LQCEGGQVYEAcGPTCP | 2.831683 | TBI(extreme) | TBI 47 |
| SSPO | QCSScQGGDFSKR | 2.831683 | TBI(extreme) | TBI 47 |
| SSPO | TcVAGILQcQEVPDcPD | 1.845547 | Control(minimal) | control 4 |
| SSPO | TcVAGILQcQEVPDcPD | 1.845547 | Control(minimal) | control 4 |
| SSPO | TcVAGILQcQEVPDcPD | 1.845547 | Control(minimal) | control 4 |
| SSPO | TcVAGILQcQEVPDcPD | 1.845547 | Control(minimal) | control 4 |
| SSPO | LRTScGNCSCAH | 3.116137 | Control(minimal) | control 7 |
| SSPO | LRTScGNCSCAH | 3.116137 | Control(minimal) | control 7 |
| SSPO | LRTScGNCSCAH | 3.116137 | Control(minimal) | control 7 |
| SSPO | PVsPGPASGVPHHGESVQM | 3.116137 | Control(minimal) | control 7 |
| SSPO | PVsPGPASGVPHHGESVQM | 3.116137 | Control(minimal) | control 7 |
| SSPO | PVsPGPASGVPHHGESVQM | 3.116137 | Control(minimal) | control 7 |
| SSPO | PVsPGPASGVPHHGESVQM | 1.888753 | Control(minimal) | control 7 |
| SSPO | PVsPGPASGVPHHGESVQM | 1.888753 | Control(minimal) | control 7 |
| SSPO | PVsPGPASGVPHHGESVQM | 1.888753 | Control(minimal) | control 7 |
| SSPO | PVsPGPASGVPHHGESVQM | 1.888753 | Control(minimal) | control 7 |
| SSPO | AAPGcGEGQMTCSSGHcLP | 1.830968 | Control(minimal) | control 8 |
| SSPO | AAPGcGEGQMTCSSGHcLP | 1.830968 | Control(minimal) | control 8 |
| SSPO | AAPGcGEGQMTCSSGHcLP | 1.830968 | Control(minimal) | control 8 |
| SSPO | AAPGcGEGQMTCSSGHcLP | 1.830968 | Control(minimal) | control 8 |
| SSPO | AAPGcGEGQMTCSSGHcLP | 1.830968 | Control(minimal) | control 8 |
| SSPO | AAPGcGEGQMTCSSGHcLP | 1.830968 | Control(minimal) | control 8 |
| SSPO | AAPGcGEGQMTCSSGHcLP | 1.830968 | Control(minimal) | control 8 |
| SSPO | LEPASCPcEWGRNsFP | 4.11781 | Control(minimal) | control 10 |
| SSPO | LEPAScPCEWGRNsFP | 4.11781 | Control(minimal) | control 10 |
| SSPO | PCSGGTDcELGRVYVSA | 4.11781 | Control(minimal) | control 10 |
| SSPO | LEPASCPcEWGRNsFP | 2.24396 | Control(minimal) | control 10 |
| SSPO | LEPASCPcEWGRNsFP | 2.24396 | Control(minimal) | control 10 |
| SSPO | LEPASCPcEWGRNsFP | 2.24396 | Control(minimal) | control 10 |
| SSPO | LEPASCPcEWGRNsFP | 2.24396 | Control(minimal) | control 10 |
| SSPO | LEPAScPCEWGRNsFP | 2.053366 | Control(minimal) | control 10 |
| SSPO | LEPAScPCEWGRNsFP | 2.053366 | Control(minimal) | control 10 |
| SSPO | LEPAScPCEWGRNsFP | 2.053366 | Control(minimal) | control 10 |
| SSPO | LEPAScPCEWGRNsFP | 2.053366 | Control(minimal) | control 10 |
| SSPO | PCSGGTDcELGRVYVSA | 1.873849 | Control(minimal) | control 10 |
| SSPO | PCSGGTDcELGRVYVSA | 1.873849 | Control(minimal) | control 10 |
| SSPO | PCSGGTDcELGRVYVSA | 1.873849 | Control(minimal) | control 10 |
| SSPO | PCSGGTDcELGRVYVSA | 1.873849 | Control(minimal) | control 10 |
| SV2C | yQGIPSmNQAKDSIV | 2.071128 | TBI(extreme) | TBI 46 |
| SV2C | yQGIPSmNQAKDSIV | 2.071128 | TBI(extreme) | TBI 46 |
| SV2C | yQGIPSmNQAKDSIV | 2.071128 | TBI(extreme) | TBI 46 |
| SV2C | yQGIPSmNQAKDSIV | 2.071128 | TBI(extreme) | TBI 46 |
| SV2C | yQGIPSmNQAKDSIV | 2.071128 | TBI(extreme) | TBI 46 |
| SV2C | yQGIPSmNQAKDSIV | 2.071128 | TBI(extreme) | TBI 46 |
| SV2C | YQGIPsmNQAKDSIV | 2.071128 | TBI(extreme) | TBI 46 |
| SV2C | YQGIPsmNQAKDSIV | 2.071128 | TBI(extreme) | TBI 46 |
| SV2C | yQGIPSmNQAKDSIV | 2.071128 | TBI(extreme) | TBI 46 |
| SV2C | YQGIPsmNQAKDSIV | 2.071128 | TBI(extreme) | TBI 46 |
| SV2C | yQGIPSmNQAKDSIV | 2.071128 | TBI(extreme) | TBI 46 |
| SV2C | YQGIPsmNQAKDSIV | 2.071128 | TBI(extreme) | TBI 46 |
| SV2C | yQGIPSmNQAKDSIV | 2.071128 | TBI(extreme) | TBI 46 |
| SV2C | YQGIPsmNQAKDSIV | 2.071128 | TBI(extreme) | TBI 46 |
| SV2C | yQGIPSmNQAKDSIV | 2.071128 | TBI(extreme) | TBI 46 |
| SV2C | YQGIPsmNQAKDSIV | 2.071128 | TBI(extreme) | TBI 46 |
| SV2C | yQGIPSmNQAKDSIV | 2.071128 | TBI(extreme) | TBI 46 |
| SV2C | YQGIPsmNQAKDSIV | 1.753647 | TBI(extreme) | TBI 46 |
| SV2C | YQGIPsmNQAKDSIV | 1.753647 | TBI(extreme) | TBI 46 |
| SV2C | YQGIPsmNQAKDSIV | 1.753647 | TBI(extreme) | TBI 46 |
| SV2C | YQGIPsmNQAKDSIV | 1.753647 | TBI(extreme) | TBI 46 |
| SV2C | YQGIPsmNQAKDSIV | 1.753647 | TBI(extreme) | TBI 46 |
| SV2C | YQGIPsmNQAKDSIV | 1.753647 | TBI(extreme) | TBI 46 |
| SV2C | YQGIPsmNQAKDSIV | 1.753647 | TBI(extreme) | TBI 46 |
| SV2C | mDRIGRLtmLGGSMVLS | 2.557801 | TBI(extreme) | TBI 30 |
| SV2C | mDRIGRLtmLGGSMVLS | 2.557801 | TBI(extreme) | TBI 30 |
| SV2C | mDRIGRLtmLGGSMVLS | 2.557801 | TBI(extreme) | TBI 30 |
| SV2C | mDRIGRLtmLGGSMVLS | 2.557801 | TBI(extreme) | TBI 30 |
| SV2C | mDRIGRLtmLGGSMVLS | 2.557801 | TBI(extreme) | TBI 30 |
| SV2C | mDRIGRLtmLGGSMVLS | 2.557801 | TBI(extreme) | TBI 30 |
| SV2C | VELYPtDRRATGFGF | 2.146806 | Control(minimal) | control 13 |
| SV2C | VELYPtDRRATGFGF | 2.146806 | Control(minimal) | control 13 |
| SV2C | VELYPtDRRATGFGF | 2.146806 | Control(minimal) | control 13 |
| SV2C | VELYPtDRRATGFGF | 2.146806 | Control(minimal) | control 13 |
| SYNE1 | sEPLDAAIIEEELDE | 1.894575 | Control(minimal) | control 7 |
| SYNE1 | sEPLDAAIIEEELDE | 1.894575 | Control(minimal) | control 7 |
| SYNE1 | sEPLDAAIIEEELDE | 1.894575 | Control(minimal) | control 7 |
| SYNE1 | sEPLDAAIIEEELDE | 1.894575 | Control(minimal) | control 7 |
| SYNE1 | sEPLDAAIIEEELDE | 1.894575 | Control(minimal) | control 7 |
| SYNE1 | sEPLDAAIIEEELDE | 1.894575 | Control(minimal) | control 7 |
| SYNE1 | sEPLDAAIIEEELDE | 1.894575 | Control(minimal) | control 7 |
| SYNE1 | sEPLDAAIIEEELDE | 1.894575 | Control(minimal) | control 7 |
| SYNE1 | sEPLDAAIIEEELDE | 1.894575 | Control(minimal) | control 7 |
| SYNE1 | sEPLDAAIIEEELDE | 1.894575 | Control(minimal) | control 7 |
| SYNE1 | sEPLDAAIIEEELDE | 1.894575 | Control(minimal) | control 7 |
| SYNE1 | sEPLDAAIIEEELDE | 1.894575 | Control(minimal) | control 7 |
| SYNE1 | sEPLDAAIIEEELDE | 1.894575 | Control(minimal) | control 7 |
| SYNE1 | sEPLDAAIIEEELDE | 1.894575 | Control(minimal) | control 7 |
| SYNE1 | sEPLDAAIIEEELDE | 1.894575 | Control(minimal) | control 7 |
| SYNE1 | sEPLDAAIIEEELDE | 1.894575 | Control(minimal) | control 7 |
| SYNE1 | sEPLDAAIIEEELDE | 1.894575 | Control(minimal) | control 7 |
| SYNE1 | sEPLDAAIIEEELDE | 1.894575 | Control(minimal) | control 7 |
| SYNE1 | sEPLDAAIIEEELDE | 1.894575 | Control(minimal) | control 7 |
| SYNE1 | sEPLDAAIIEEELDE | 1.894575 | Control(minimal) | control 7 |
| SYNE1 | sEPLDAAIIEEELDE | 1.894575 | Control(minimal) | control 7 |
| SYNE1 | sEPLDAAIIEEELDE | 1.894575 | Control(minimal) | control 7 |
| SYNE1 | sEPLDAAIIEEELDE | 1.894575 | Control(minimal) | control 7 |
| SYNE1 | sEPLDAAIIEEELDE | 1.894575 | Control(minimal) | control 7 |
| SYNE1 | sEPLDAAIIEEELDE | 1.894575 | Control(minimal) | control 7 |
| SYNE1 | sEPLDAAIIEEELDE | 1.894575 | Control(minimal) | control 7 |
| SYNE1 | sEPLDAAIIEEELDE | 1.894575 | Control(minimal) | control 7 |
| SYNE1 | sEPLDAAIIEEELDE | 1.894575 | Control(minimal) | control 7 |
| SYNE1 | sEPLDAAIIEEELDE | 1.894575 | Control(minimal) | control 7 |
| SYNE1 | sEPLDAAIIEEELDE | 1.894575 | Control(minimal) | control 7 |
| SYNE1 | sEPLDAAIIEEELDE | 1.894575 | Control(minimal) | control 7 |
| SYNE1 | cQALESLSSAITAFsA | 1.75192 | Control(minimal) | control 12 |
| SYNE1 | cQALESLSSAITAFsA | 1.75192 | Control(minimal) | control 12 |
| SYNE1 | cQALESLSSAITAFsA | 1.75192 | Control(minimal) | control 12 |
| SYNE1 | cQALESLSSAITAFsA | 1.75192 | Control(minimal) | control 12 |
| SYNE1 | cQALESLSSAITAFsA | 1.75192 | Control(minimal) | control 12 |
| SYNE1 | cQALESLSSAITAFsA | 1.75192 | Control(minimal) | control 12 |
| SYNE1 | cQALESLSSAITAFsA | 1.75192 | Control(minimal) | control 12 |
| SYNE1 | cQALESLSSAITAFsA | 1.75192 | Control(minimal) | control 12 |
| SYNE1 | cQALESLSSAITAFsA | 1.75192 | Control(minimal) | control 12 |
| SYNE1 | cQALESLSSAITAFsA | 1.75192 | Control(minimal) | control 12 |
| SYNE1 | cQALESLSSAITAFsA | 1.75192 | Control(minimal) | control 12 |
| SYNE1 | cQALESLSSAITAFsA | 1.75192 | Control(minimal) | control 12 |
| SYNE1 | cQALESLSSAITAFsA | 1.75192 | Control(minimal) | control 12 |
| SYNE1 | cQALESLSSAITAFsA | 1.75192 | Control(minimal) | control 12 |
| SYNE1 | cQALESLSSAITAFsA | 1.75192 | Control(minimal) | control 12 |
| SYNE1 | cQALESLSSAITAFsA | 1.75192 | Control(minimal) | control 12 |
| SYNE1 | cQALESLSSAITAFsA | 1.75192 | Control(minimal) | control 12 |
| SYNE1 | KGLHLAKEFsDKCKA | 2.067942 | Control(minimal) | control 13 |
| SYNE1 | KGLHLAKEFsDKCKA | 2.067942 | Control(minimal) | control 13 |
| SYNE1 | KGLHLAKEFsDKCKA | 2.067942 | Control(minimal) | control 13 |
| SYNE1 | KGLHLAKEFsDKCKA | 2.067942 | Control(minimal) | control 13 |
| SYNE1 | KGLHLAKEFsDKCKA | 2.067942 | Control(minimal) | control 13 |
| SYNE1 | KGLHLAKEFsDKCKA | 2.067942 | Control(minimal) | control 13 |
| SYNE1 | KGLHLAKEFsDKCKA | 2.067942 | Control(minimal) | control 13 |
| SYNE1 | KGLHLAKEFsDKCKA | 2.067942 | Control(minimal) | control 13 |
| SYNE1 | KGLHLAKEFsDKCKA | 2.067942 | Control(minimal) | control 13 |
| TCF20 | EGHNVGsNAQAYGTQ | 2.007459 | Control(minimal) | control 9 |
| TCF20 | EGHNVGsNAQAYGTQ | 2.007459 | Control(minimal) | control 9 |
| TCF20 | EGHNVGsNAQAYGTQ | 2.007459 | Control(minimal) | control 9 |
| TCF20 | EGHNVGsNAQAYGTQ | 2.007459 | Control(minimal) | control 9 |
| TCF20 | EGHNVGsNAQAYGTQ | 2.007459 | Control(minimal) | control 9 |
| TCF20 | DcGGGPRScPGGsLVKK | 1.790444 | Control(minimal) | control 10 |
| TCF20 | DcGGGPRScPGGsLVKK | 1.790444 | Control(minimal) | control 10 |
| TCF20 | DcGGGPRScPGGsLVKK | 1.790444 | Control(minimal) | control 10 |
| TCF20 | EGHNVGsNAQAYGTQ | 2.007459 | Control(minimal) | control 11 |
| TCF20 | EGHNVGsNAQAYGTQ | 2.007459 | Control(minimal) | control 11 |
| TCF20 | EGHNVGsNAQAYGTQ | 2.007459 | Control(minimal) | control 11 |
| TCF20 | EGHNVGsNAQAYGTQ | 2.007459 | Control(minimal) | control 11 |
| TCF20 | EGHNVGsNAQAYGTQ | 2.007459 | Control(minimal) | control 11 |
| TENM2 | GVCIGGAcRCEEGWTGAAc | 1.742998 | TBI(extreme) | TBI 2 |
| TENM2 | GVCIGGAcRCEEGWTGAAc | 1.742998 | TBI(extreme) | TBI 2 |
| TENM2 | GVCIGGAcRCEEGWTGAAc | 1.742998 | TBI(extreme) | TBI 2 |
| TENM2 | GVCIGGAcRCEEGWTGAAc | 1.742998 | TBI(extreme) | TBI 2 |
| TENM2 | GVCIGGAcRCEEGWTGAAc | 1.742998 | TBI(extreme) | TBI 2 |
| TENM2 | GVCIGGAcRCEEGWTGAAc | 1.742998 | TBI(extreme) | TBI 2 |
| TENM2 | GVCIGGAcRCEEGWTGAAc | 1.742998 | TBI(extreme) | TBI 2 |
| TENM2 | GVCIGGAcRCEEGWTGAAc | 1.742998 | TBI(extreme) | TBI 2 |
| TENM2 | GVCIGGAcRCEEGWTGAAc | 1.742998 | TBI(extreme) | TBI 2 |
| TENM2 | GVCIGGAcRCEEGWTGAAc | 1.742998 | TBI(extreme) | TBI 2 |
| TENM2 | GVCIGGAcRCEEGWTGAAc | 1.742998 | TBI(extreme) | TBI 2 |
| TENM2 | PDTGLcScDPNWMGPDC | 1.789589 | Control(minimal) | control 7 |
| TENM2 | PDTGLcScDPNWMGPDC | 1.789589 | Control(minimal) | control 7 |
| TENM2 | PDTGLcScDPNWMGPDC | 1.789589 | Control(minimal) | control 7 |
| TENM2 | PDTGLcScDPNWMGPDC | 1.789589 | Control(minimal) | control 7 |
| TENM2 | PDTGLcScDPNWMGPDC | 1.789589 | Control(minimal) | control 7 |
| TENM2 | PDTGLcScDPNWMGPDC | 1.789589 | Control(minimal) | control 7 |
| TENM2 | PDTGLcScDPNWMGPDC | 1.789589 | Control(minimal) | control 7 |
| TENM2 | PDTGLcScDPNWMGPDC | 1.789589 | Control(minimal) | control 7 |
| TENM2 | PDTGLcScDPNWMGPDC | 1.789589 | Control(minimal) | control 7 |
| TENM2 | PDTGLcScDPNWMGPDC | 1.789589 | Control(minimal) | control 7 |
| TENM2 | PDTGLcScDPNWMGPDC | 1.789589 | Control(minimal) | control 7 |
| TENM2 | GDGLVDcLDPDccLQsA | 2.03132 | Control(minimal) | control 9 |
| TENM2 | GDGLVDcLDPDccLQsA | 2.03132 | Control(minimal) | control 9 |
| TENM2 | GDGLVDcLDPDccLQsA | 2.03132 | Control(minimal) | control 9 |
| TENM2 | GDGLVDcLDPDccLQsA | 2.03132 | Control(minimal) | control 9 |
| TENM2 | GDGLVDcLDPDccLQsA | 2.03132 | Control(minimal) | control 9 |
| TENM2 | GDGLVDcLDPDccLQsA | 2.03132 | Control(minimal) | control 9 |
| TENM2 | GDGLVDcLDPDccLQsA | 2.03132 | Control(minimal) | control 11 |
| TENM2 | GDGLVDcLDPDccLQsA | 2.03132 | Control(minimal) | control 11 |
| TENM2 | GDGLVDcLDPDccLQsA | 2.03132 | Control(minimal) | control 11 |
| TENM2 | GDGLVDcLDPDccLQsA | 2.03132 | Control(minimal) | control 11 |
| TENM2 | GDGLVDcLDPDccLQsA | 2.03132 | Control(minimal) | control 11 |
| TENM2 | GDGLVDcLDPDccLQsA | 2.03132 | Control(minimal) | control 11 |
| TENM3 | DcSRAACPVLCsGNGQY | 1.884361 | TBI(extreme) | TBI 46 |
| TENM3 | DcSRAACPVLCsGNGQY | 1.884361 | TBI(extreme) | TBI 46 |
| TENM3 | DcSRAACPVLCsGNGQY | 1.884361 | TBI(extreme) | TBI 46 |
| TENM3 | DcSRAACPVLCsGNGQY | 1.884361 | TBI(extreme) | TBI 46 |
| TENM3 | DcSRAACPVLCsGNGQY | 1.884361 | TBI(extreme) | TBI 46 |
| TENM3 | DcSRAACPVLCsGNGQY | 1.884361 | TBI(extreme) | TBI 46 |
| TENM3 | DcSRAACPVLCsGNGQY | 1.884361 | TBI(extreme) | TBI 46 |
| TENM3 | DcSRAACPVLCsGNGQY | 1.884361 | TBI(extreme) | TBI 46 |
| TENM3 | DcSRAACPVLCsGNGQY | 1.884361 | TBI(extreme) | TBI 46 |
| TENM3 | DcSRAACPVLCsGNGQY | 1.884361 | TBI(extreme) | TBI 46 |
| TENM3 | DcSRAACPVLCsGNGQY | 1.884361 | TBI(extreme) | TBI 46 |
| TENM3 | DcSRAACPVLCsGNGQY | 1.884361 | TBI(extreme) | TBI 46 |
| TENM3 | DcSRAACPVLCsGNGQY | 1.884361 | TBI(extreme) | TBI 46 |
| TENM3 | GEHctIEGCPGLCNSN | 1.776788 | TBI(extreme) | TBI 47 |
| TENM3 | GEHctIEGCPGLCNSN | 1.776788 | TBI(extreme) | TBI 47 |
| TENM3 | GEHctIEGCPGLCNSN | 1.776788 | TBI(extreme) | TBI 47 |
| TENM3 | GEHctIEGCPGLCNSN | 1.776788 | TBI(extreme) | TBI 47 |
| TENM3 | GEHctIEGCPGLCNSN | 1.776788 | TBI(extreme) | TBI 47 |
| TENM3 | GEHctIEGCPGLCNSN | 1.776788 | TBI(extreme) | TBI 47 |
| TENM3 | GEHctIEGCPGLCNSN | 1.776788 | TBI(extreme) | TBI 47 |
| TENM3 | NDANcDcYQsGDGYAKD | 1.966862 | Control(minimal) | control 3 |
| TENM3 | NDANcDcYQsGDGYAKD | 1.966862 | Control(minimal) | control 3 |
| TENM3 | NDANcDcYQsGDGYAKD | 1.966862 | Control(minimal) | control 3 |
| TENM3 | NDANcDcYQsGDGYAKD | 1.966862 | Control(minimal) | control 3 |
| TENM3 | NDANcDcYQsGDGYAKD | 1.966862 | Control(minimal) | control 3 |
| TENM3 | NDANcDcYQsGDGYAKD | 1.966862 | Control(minimal) | control 3 |
| TENM3 | NDANcDcYQsGDGYAKD | 1.966862 | Control(minimal) | control 3 |
| TENM3 | NDANcDcYQSGDGyAKD | 1.966862 | Control(minimal) | control 3 |
| TENM3 | NDANcDcYQSGDGyAKD | 1.966862 | Control(minimal) | control 3 |
| TENM3 | NDANcDcYQSGDGyAKD | 1.966862 | Control(minimal) | control 3 |
| TENM3 | NDANcDcYQSGDGyAKD | 1.966862 | Control(minimal) | control 3 |
| TENM3 | NDANcDcYQSGDGyAKD | 1.966862 | Control(minimal) | control 3 |
| TENM3 | NDANcDcYQSGDGyAKD | 1.966862 | Control(minimal) | control 3 |
| TENM3 | NDANcDcYQSGDGyAKD | 1.845551 | Control(minimal) | control 3 |
| TENM4 | MTHTKHFDAYGRMKE | 1.832009 | TBI(extreme) | TBI 1 |
| TENM4 | MTHTKHFDAYGRMKE | 1.832009 | TBI(extreme) | TBI 1 |
| TENM4 | MTHTKHFDAYGRMKE | 1.832009 | TBI(extreme) | TBI 1 |
| TENM4 | MTHTKHFDAYGRMKE | 1.832009 | TBI(extreme) | TBI 1 |
| TENM4 | MTHTKHFDAYGRMKE | 1.832009 | TBI(extreme) | TBI 1 |
| TENM4 | MTHTKHFDAYGRMKE | 1.832009 | TBI(extreme) | TBI 1 |
| TENM4 | MTHTKHFDAYGRMKE | 1.832009 | TBI(extreme) | TBI 1 |
| TENM4 | MTHTKHFDAYGRMKE | 1.832009 | TBI(extreme) | TBI 1 |
| TENM4 | MTHTKHFDAYGRMKE | 1.832009 | TBI(extreme) | TBI 1 |
| TENM4 | MTHTKHFDAYGRMKE | 1.832009 | TBI(extreme) | TBI 1 |
| TENM4 | MTHTKHFDAYGRMKE | 1.832009 | TBI(extreme) | TBI 1 |
| TENM4 | MTHTKHFDAYGRMKE | 1.832009 | TBI(extreme) | TBI 1 |
| TENM4 | MTHTKHFDAYGRMKE | 1.832009 | TBI(extreme) | TBI 1 |
| TENM4 | MTHTKHFDAYGRMKE | 1.832009 | TBI(extreme) | TBI 8 |
| TENM4 | MTHTKHFDAYGRMKE | 1.832009 | TBI(extreme) | TBI 8 |
| TENM4 | MTHTKHFDAYGRMKE | 1.832009 | TBI(extreme) | TBI 8 |
| TENM4 | MTHTKHFDAYGRMKE | 1.832009 | TBI(extreme) | TBI 8 |
| TENM4 | MTHTKHFDAYGRMKE | 1.832009 | TBI(extreme) | TBI 8 |
| TENM4 | MTHTKHFDAYGRMKE | 1.832009 | TBI(extreme) | TBI 8 |
| TENM4 | MTHTKHFDAYGRMKE | 1.832009 | TBI(extreme) | TBI 8 |
| TENM4 | MTHTKHFDAYGRMKE | 1.832009 | TBI(extreme) | TBI 8 |
| TENM4 | MTHTKHFDAYGRMKE | 1.832009 | TBI(extreme) | TBI 8 |
| TENM4 | MTHTKHFDAYGRMKE | 1.832009 | TBI(extreme) | TBI 8 |
| TENM4 | MTHTKHFDAYGRMKE | 1.832009 | TBI(extreme) | TBI 8 |
| TENM4 | MTHTKHFDAYGRMKE | 1.832009 | TBI(extreme) | TBI 8 |
| TENM4 | MTHTKHFDAYGRMKE | 1.832009 | TBI(extreme) | TBI 8 |
| TENM4 | NVARQTLETIRsV | 1.732403 | Control(minimal) | control 13 |
| TENM4 | NVARQTLETIRsV | 1.732403 | Control(minimal) | control 13 |
| TENM4 | NVARQTLETIRsV | 1.732403 | Control(minimal) | control 13 |
| TENM4 | NVARQTLEtIRSV | 1.732403 | Control(minimal) | control 13 |
| TENM4 | NVARQTLETIRsV | 1.732403 | Control(minimal) | control 13 |
| TENM4 | NVARQTLEtIRSV | 1.732403 | Control(minimal) | control 13 |
| TENM4 | NVARQTLETIRsV | 1.732403 | Control(minimal) | control 13 |
| TENM4 | NVARQTLEtIRSV | 1.732403 | Control(minimal) | control 13 |
| TENM4 | NVARQTLETIRsV | 1.732403 | Control(minimal) | control 13 |
| TENM4 | NVARQTLEtIRSV | 1.732403 | Control(minimal) | control 13 |
| TENM4 | NVARQTLETIRsV | 1.732403 | Control(minimal) | control 13 |
| TENM4 | NVARQTLEtIRSV | 1.732403 | Control(minimal) | control 13 |
| TENM4 | NVARQTLETIRsV | 1.732403 | Control(minimal) | control 13 |
| TENM4 | NVARQTLEtIRSV | 1.732403 | Control(minimal) | control 13 |
| TENM4 | NVARQTLETIRsV | 1.732403 | Control(minimal) | control 13 |
| TENM4 | NVARQTLEtIRSV | 1.732403 | Control(minimal) | control 13 |
| TENM4 | NVARQTLETIRsV | 1.732403 | Control(minimal) | control 13 |
| TENM4 | NVARQTLEtIRSV | 1.732403 | Control(minimal) | control 13 |
| TENM4 | NVARQTLETIRsV | 1.732403 | Control(minimal) | control 13 |
| TENM4 | NVARQTLEtIRSV | 1.732403 | Control(minimal) | control 13 |
| TENM4 | NVARQTLETIRsV | 1.732403 | Control(minimal) | control 13 |
| TENM4 | NVARQTLEtIRSV | 1.721735 | Control(minimal) | control 13 |
| TENM4 | NVARQTLEtIRSV | 1.721735 | Control(minimal) | control 13 |
| TENM4 | NVARQTLEtIRSV | 1.721735 | Control(minimal) | control 13 |
| TENM4 | NVARQTLEtIRSV | 1.721735 | Control(minimal) | control 13 |
| TENM4 | NVARQTLEtIRSV | 1.721735 | Control(minimal) | control 13 |
| TENM4 | NVARQTLEtIRSV | 1.721735 | Control(minimal) | control 13 |
| TENM4 | NVARQTLEtIRSV | 1.721735 | Control(minimal) | control 13 |
| TENM4 | NVARQTLEtIRSV | 1.721735 | Control(minimal) | control 13 |
| TENM4 | NVARQTLEtIRSV | 1.721735 | Control(minimal) | control 13 |
| TENM4 | NVARQTLEtIRSV | 1.721735 | Control(minimal) | control 13 |
| TENM4 | NVARQTLEtIRSV | 1.721735 | Control(minimal) | control 13 |
| TFR2 | LGYVAFRGScQACGDs | 1.76736 | Control(minimal) | control 7 |
| TFR2 | LGYVAFRGScQACGDs | 1.76736 | Control(minimal) | control 7 |
| TFR2 | LGYVAFRGScQACGDs | 1.76736 | Control(minimal) | control 7 |
| TFR2 | LGYVAFRGScQACGDs | 1.76736 | Control(minimal) | control 7 |
| TFR2 | LGYVAFRGScQACGDs | 1.76736 | Control(minimal) | control 7 |
| TFR2 | LGYVAFRGScQACGDs | 1.76736 | Control(minimal) | control 7 |
| TFR2 | LGYVAFRGScQACGDs | 1.76736 | Control(minimal) | control 7 |
| TFR2 | LGYVAFRGScQACGDs | 1.76736 | Control(minimal) | control 7 |
| TFR2 | LGYVAFRGScQACGDs | 1.76736 | Control(minimal) | control 7 |
| TFR2 | LGYVAFRGScQACGDs | 1.76736 | Control(minimal) | control 7 |
| TFR2 | LGYVAFRGScQACGDs | 1.76736 | Control(minimal) | control 7 |
| TFR2 | LGYVAFRGScQACGDs | 1.76736 | Control(minimal) | control 7 |
| TFR2 | LGYVAFRGScQACGDs | 1.76736 | Control(minimal) | control 7 |
| TFR2 | LGYVAFRGScQACGDs | 1.76736 | Control(minimal) | control 7 |
| TFR2 | LGYVAFRGScQACGDs | 1.76736 | Control(minimal) | control 7 |
| TFR2 | LGYVAFRGscQACGDS | 1.869042 | Control(minimal) | control 8 |
| TFR2 | LGYVAFRGscQACGDS | 1.869042 | Control(minimal) | control 8 |
| TFR2 | LGYVAFRGscQACGDS | 1.869042 | Control(minimal) | control 8 |
| TFR2 | LGYVAFRGscQACGDS | 1.869042 | Control(minimal) | control 8 |
| TFR2 | LGYVAFRGscQACGDS | 1.869042 | Control(minimal) | control 8 |
| TFR2 | LGYVAFRGscQACGDS | 1.869042 | Control(minimal) | control 8 |
| TFR2 | LGYVAFRGscQACGDS | 1.869042 | Control(minimal) | control 8 |
| TFR2 | LGYVAFRGscQACGDS | 1.869042 | Control(minimal) | control 8 |
| TFR2 | LGYVAFRGscQACGDS | 1.869042 | Control(minimal) | control 8 |
| TFR2 | LGYVAFRGscQACGDS | 1.869042 | Control(minimal) | control 8 |
| TFR2 | LGYVAFRGscQACGDS | 1.869042 | Control(minimal) | control 8 |
| TFR2 | LGYVAFRGscQACGDS | 1.869042 | Control(minimal) | control 8 |
| TFR2 | LGYVAFRGscQACGDS | 1.869042 | Control(minimal) | control 8 |
| TFR2 | LGYVAFRGscQACGDS | 1.869042 | Control(minimal) | control 8 |
| TFR2 | LGYVAFRGscQACGDS | 1.869042 | Control(minimal) | control 8 |
| TMC4 | cGLcPGALGRLAGTQEFQV | 1.809143 | TBI(extreme) | TBI 9 |
| TMC4 | cGLcPGALGRLAGTQEFQV | 1.809143 | TBI(extreme) | TBI 9 |
| TMC4 | cGLcPGALGRLAGTQEFQV | 1.809143 | TBI(extreme) | TBI 9 |
| TMC4 | cGLcPGALGRLAGTQEFQV | 1.809143 | TBI(extreme) | TBI 9 |
| TMC4 | cGLcPGALGRLAGTQEFQV | 1.809143 | TBI(extreme) | TBI 9 |
| TMC4 | cGLcPGALGRLAGTQEFQV | 1.809143 | TBI(extreme) | TBI 9 |
| TMC4 | cGLcPGALGRLAGTQEFQV | 1.809143 | TBI(extreme) | TBI 9 |
| TMC4 | cGLcPGALGRLAGTQEFQV | 1.809143 | TBI(extreme) | TBI 9 |
| TMC4 | cGLcPGALGRLAGTQEFQV | 1.809143 | TBI(extreme) | TBI 9 |
| TMC4 | cGLcPGALGRLAGTQEFQV | 1.809143 | TBI(extreme) | TBI 9 |
| TMC4 | cGLcPGALGRLAGTQEFQV | 1.809143 | TBI(extreme) | TBI 9 |
| TMC4 | MAcMTLLPtWLGGAPP | 2.090915 | TBI(extreme) | TBI 30 |
| TMC4 | MAcMTLLPtWLGGAPP | 2.090915 | TBI(extreme) | TBI 30 |
| TMC4 | MAcMTLLPtWLGGAPP | 2.090915 | TBI(extreme) | TBI 30 |
| TMC4 | MAcMTLLPtWLGGAPP | 2.090915 | TBI(extreme) | TBI 30 |
| TMC4 | MAcMTLLPtWLGGAPP | 2.090915 | TBI(extreme) | TBI 30 |
| TMEM241 | TLHNVAEVIIcGyQKc | 1.72685 | TBI(extreme) | TBI 1 |
| TMEM241 | TLHNVAEVIIcGyQKc | 1.72685 | TBI(extreme) | TBI 1 |
| TMEM241 | TLHNVAEVIIcGyQKc | 1.72685 | TBI(extreme) | TBI 1 |
| TMEM241 | TLHNVAEVIIcGyQKc | 1.72685 | TBI(extreme) | TBI 1 |
| TMEM241 | TLHNVAEVIIcGyQKc | 1.72685 | TBI(extreme) | TBI 1 |
| TMEM241 | TLHNVAEVIIcGyQKc | 1.72685 | TBI(extreme) | TBI 1 |
| TMEM241 | TLHNVAEVIIcGyQKc | 1.72685 | TBI(extreme) | TBI 1 |
| TMEM241 | FSVVLLAFAsHPTGDL | 1.964289 | TBI(extreme) | TBI 8 |
| TMEM241 | FSVVLLAFAsHPTGDL | 1.964289 | TBI(extreme) | TBI 8 |
| TMEM241 | FSVVLLAFAsHPTGDL | 1.964289 | TBI(extreme) | TBI 8 |
| TMEM241 | FSVVLLAFAsHPTGDL | 1.964289 | TBI(extreme) | TBI 8 |
| TMEM241 | FSVVLLAFAsHPTGDL | 1.964289 | TBI(extreme) | TBI 8 |
| TMEM241 | TLHNVAEVIIcGyQKc | 1.72685 | TBI(extreme) | TBI 8 |
| TMEM241 | TLHNVAEVIIcGyQKc | 1.72685 | TBI(extreme) | TBI 8 |
| TMEM241 | TLHNVAEVIIcGyQKc | 1.72685 | TBI(extreme) | TBI 8 |
| TMEM241 | TLHNVAEVIIcGyQKc | 1.72685 | TBI(extreme) | TBI 8 |
| TMEM241 | TLHNVAEVIIcGyQKc | 1.72685 | TBI(extreme) | TBI 8 |
| TMEM241 | TLHNVAEVIIcGyQKc | 1.72685 | TBI(extreme) | TBI 8 |
| TMEM241 | TLHNVAEVIIcGyQKc | 1.72685 | TBI(extreme) | TBI 8 |
| TMEM55B | YVRCPcNcLLIcKVt | 2.316236 | TBI(extreme) | TBI 1 |
| TMEM55B | YVRCPcNcLLIcKVt | 2.316236 | TBI(extreme) | TBI 1 |
| TMEM55B | YVRCPcNcLLIcKVt | 2.316236 | TBI(extreme) | TBI 1 |
| TMEM55B | YVRCPcNcLLIcKVt | 2.316236 | TBI(extreme) | TBI 1 |
| TMEM55B | YVRCPcNcLLIcKVt | 2.316236 | TBI(extreme) | TBI 1 |
| TMEM55B | YVRCPcNcLLIcKVt | 2.316236 | TBI(extreme) | TBI 1 |
| TMEM55B | YVRCPcNcLLIcKVt | 2.316236 | TBI(extreme) | TBI 1 |
| TMEM55B | YVRCPcNcLLIcKVt | 2.316236 | TBI(extreme) | TBI 1 |
| TMEM55B | YVRCPcNcLLIcKVt | 2.316236 | TBI(extreme) | TBI 1 |
| TMEM55B | YVRCPcNcLLIcKVt | 2.316236 | TBI(extreme) | TBI 1 |
| TMEM55B | YVRCPcNcLLIcKVt | 2.316236 | TBI(extreme) | TBI 1 |
| TMEM55B | YVRCPcNcLLIcKVt | 2.203108 | TBI(extreme) | TBI 1 |
| TMEM55B | YVRCPcNcLLIcKVt | 2.203108 | TBI(extreme) | TBI 1 |
| TMEM55B | YVRCPcNcLLIcKVt | 2.203108 | TBI(extreme) | TBI 1 |
| TMEM55B | YVRCPcNcLLIcKVt | 2.316236 | TBI(extreme) | TBI 8 |
| TMEM55B | YVRCPcNcLLIcKVt | 2.316236 | TBI(extreme) | TBI 8 |
| TMEM55B | YVRCPcNcLLIcKVt | 2.316236 | TBI(extreme) | TBI 8 |
| TMEM55B | YVRCPcNcLLIcKVt | 2.316236 | TBI(extreme) | TBI 8 |
| TMEM55B | YVRCPcNcLLIcKVt | 2.316236 | TBI(extreme) | TBI 8 |
| TMEM55B | YVRCPcNcLLIcKVt | 2.316236 | TBI(extreme) | TBI 8 |
| TMEM55B | YVRCPcNcLLIcKVt | 2.316236 | TBI(extreme) | TBI 8 |
| TMEM55B | YVRCPcNcLLIcKVt | 2.316236 | TBI(extreme) | TBI 8 |
| TMEM55B | YVRCPcNcLLIcKVt | 2.316236 | TBI(extreme) | TBI 8 |
| TMEM55B | YVRCPcNcLLIcKVt | 2.316236 | TBI(extreme) | TBI 8 |
| TMEM55B | YVRCPcNcLLIcKVt | 2.316236 | TBI(extreme) | TBI 8 |
| TMEM55B | YVRCPcNcLLIcKVt | 2.203108 | TBI(extreme) | TBI 8 |
| TMEM55B | YVRCPcNcLLIcKVt | 2.203108 | TBI(extreme) | TBI 8 |
| TMEM55B | YVRCPcNcLLIcKVt | 2.203108 | TBI(extreme) | TBI 8 |
| TNRC18 | LtPYDSLLGKNR | 1.811041 | Control(minimal) | control 9 |
| TNRC18 | LtPYDSLLGKNR | 1.811041 | Control(minimal) | control 9 |
| TNRC18 | LtPYDSLLGKNR | 1.811041 | Control(minimal) | control 9 |
| TNRC18 | LtPYDSLLGKNR | 1.811041 | Control(minimal) | control 9 |
| TNRC18 | PLESPLPLPAAEAmAtPS | 1.858385 | Control(minimal) | control 10 |
| TNRC18 | PLESPLPLPAAEAmAtPS | 1.858385 | Control(minimal) | control 10 |
| TNRC18 | PLESPLPLPAAEAmAtPS | 1.858385 | Control(minimal) | control 10 |
| TNRC18 | PLESPLPLPAAEAmAtPS | 1.858385 | Control(minimal) | control 10 |
| TNRC18 | PLESPLPLPAAEAmAtPS | 1.858385 | Control(minimal) | control 10 |
| TNRC18 | PLESPLPLPAAEAmAtPS | 1.858385 | Control(minimal) | control 10 |
| TNRC18 | PLESPLPLPAAEAmAtPS | 1.858385 | Control(minimal) | control 10 |
| TNRC18 | LtPYDSLLGKNR | 1.811041 | Control(minimal) | control 11 |
| TNRC18 | LtPYDSLLGKNR | 1.811041 | Control(minimal) | control 11 |
| TNRC18 | LtPYDSLLGKNR | 1.811041 | Control(minimal) | control 11 |
| TNRC18 | LtPYDSLLGKNR | 1.811041 | Control(minimal) | control 11 |
| TRB | PLETCcSSELKGGGSGTs | 1.924923 | TBI(extreme) | TBI 9 |
| TRB | PLETCcSSELKGGGSGtS | 1.924923 | TBI(extreme) | TBI 9 |
| TRB | PLETcCSSELKGGGsGTS | 1.924923 | TBI(extreme) | TBI 9 |
| TRB | PLETcCSSELKGGGsGTS | 1.924923 | TBI(extreme) | TBI 9 |
| TRB | PLETcCSSELKGGGsGTS | 1.924923 | TBI(extreme) | TBI 9 |
| TRB | PLETcCSSELKGGGsGTS | 1.924923 | TBI(extreme) | TBI 9 |
| TRB | LLLLLGPGSGLSAVVsQH | 1.749386 | TBI(extreme) | TBI 47 |
| TRB | LLLLLGPGSGLSAVVsQH | 1.749386 | TBI(extreme) | TBI 47 |
| TRB | LLLLLGPGSGLSAVVsQH | 1.749386 | TBI(extreme) | TBI 47 |
| TRB | LLLLLGPGSGLSAVVsQH | 1.749386 | TBI(extreme) | TBI 47 |
| TRB | LLLLLGPGSGLSAVVsQH | 1.749386 | TBI(extreme) | TBI 47 |
| TRB | mAGNQCALAPYSRARK | 1.76153 | Control(minimal) | control 4 |
| TRB | mAGNQCALAPYSRARK | 1.76153 | Control(minimal) | control 4 |
| TRB | mAGNQCALAPYSRARK | 1.76153 | Control(minimal) | control 4 |
| TRB | sFYIcsAGSGGtGGGSNQ | 1.73882 | Control(minimal) | control 7 |
| TRB | sFYIcsAGSGGtGGGSNQ | 1.73882 | Control(minimal) | control 7 |
| TRB | sFYIcsAGSGGtGGGSNQ | 1.73882 | Control(minimal) | control 7 |
| TRB | cASsPLLtRTLDsGNT | 2.174682 | Control(minimal) | control 8 |
| TRB | cASsPLLtRTLDsGNT | 2.174682 | Control(minimal) | control 8 |
| TRB | cASsPLLtRTLDsGNT | 2.174682 | Control(minimal) | control 8 |
| TRB | FcAAGETSGVSYNEQFF | 1.723861 | Control(minimal) | control 8 |
| TRB | FcAAGETSGVSYNEQFF | 1.723861 | Control(minimal) | control 8 |
| TRB | FcAAGETSGVSYNEQFF | 1.723861 | Control(minimal) | control 8 |
| TRB | RtQQGDSAVYLcASSLGV | 2.45875 | Control(minimal) | control 12 |
| TRB | RtQQGDSAVYLcASSLGV | 2.45875 | Control(minimal) | control 12 |
| TRB | RtQQGDSAVYLcASSLGV | 2.45875 | Control(minimal) | control 12 |
| TRPC1 | NCQQtLNTVLtEISQG | 2.242104 | TBI(extreme) | TBI 10 |
| TRPC1 | NCQQtLNTVLtEISQG | 2.242104 | TBI(extreme) | TBI 10 |
| TRPC1 | sLPKPHAVGcECTLCSA | 1.776552 | Control(minimal) | control 9 |
| TRPC1 | sLPKPHAVGcECTLCSA | 1.776552 | Control(minimal) | control 9 |
| TRPC1 | sLPKPHAVGcECTLCSA | 1.776552 | Control(minimal) | control 9 |
| TRPC1 | sLPKPHAVGcECTLCSA | 1.776552 | Control(minimal) | control 9 |
| TRPC1 | sLPKPHAVGcECTLCSA | 1.776552 | Control(minimal) | control 9 |
| TRPC1 | sLPKPHAVGCEcTLCSA | 1.776552 | Control(minimal) | control 9 |
| TRPC1 | sLPKPHAVGcECTLCSA | 1.776552 | Control(minimal) | control 9 |
| TRPC1 | sLPKPHAVGcECTLCSA | 1.776552 | Control(minimal) | control 9 |
| TRPC1 | sLPKPHAVGcECTLCSA | 1.776552 | Control(minimal) | control 9 |
| TRPC1 | sLPKPHAVGCECTLcSA | 1.776552 | Control(minimal) | control 9 |
| TRPC1 | sLPKPHAVGcECTLCSA | 1.776552 | Control(minimal) | control 9 |
| TRPC1 | sLPKPHAVGcECTLCSA | 1.776552 | Control(minimal) | control 9 |
| TRPC1 | sLPKPHAVGcECTLCSA | 1.776552 | Control(minimal) | control 9 |
| TRPC1 | sLPKPHAVGCECTLcSA | 1.776552 | Control(minimal) | control 9 |
| TRPC1 | sLPKPHAVGCECTLcSA | 1.776552 | Control(minimal) | control 9 |
| TRPC1 | sLPKPHAVGCECTLcSA | 1.776552 | Control(minimal) | control 9 |
| TRPC1 | sLPKPHAVGCECTLcSA | 1.776552 | Control(minimal) | control 9 |
| TRPC1 | sLPKPHAVGCECTLcSA | 1.776552 | Control(minimal) | control 9 |
| TRPC1 | sLPKPHAVGCECTLcSA | 1.776552 | Control(minimal) | control 9 |
| TRPC1 | sLPKPHAVGcECTLCSA | 1.776552 | Control(minimal) | control 11 |
| TRPC1 | sLPKPHAVGcECTLCSA | 1.776552 | Control(minimal) | control 11 |
| TRPC1 | sLPKPHAVGcECTLCSA | 1.776552 | Control(minimal) | control 11 |
| TRPC1 | sLPKPHAVGcECTLCSA | 1.776552 | Control(minimal) | control 11 |
| TRPC1 | sLPKPHAVGcECTLCSA | 1.776552 | Control(minimal) | control 11 |
| TRPC1 | sLPKPHAVGCEcTLCSA | 1.776552 | Control(minimal) | control 11 |
| TRPC1 | sLPKPHAVGcECTLCSA | 1.776552 | Control(minimal) | control 11 |
| TRPC1 | sLPKPHAVGcECTLCSA | 1.776552 | Control(minimal) | control 11 |
| TRPC1 | sLPKPHAVGcECTLCSA | 1.776552 | Control(minimal) | control 11 |
| TRPC1 | sLPKPHAVGCECTLcSA | 1.776552 | Control(minimal) | control 11 |
| TRPC1 | sLPKPHAVGcECTLCSA | 1.776552 | Control(minimal) | control 11 |
| TRPC1 | sLPKPHAVGcECTLCSA | 1.776552 | Control(minimal) | control 11 |
| TRPC1 | sLPKPHAVGcECTLCSA | 1.776552 | Control(minimal) | control 11 |
| TRPC1 | sLPKPHAVGCECTLcSA | 1.776552 | Control(minimal) | control 11 |
| TRPC1 | sLPKPHAVGCECTLcSA | 1.776552 | Control(minimal) | control 11 |
| TRPC1 | sLPKPHAVGCECTLcSA | 1.776552 | Control(minimal) | control 11 |
| TRPC1 | sLPKPHAVGCECTLcSA | 1.776552 | Control(minimal) | control 11 |
| TRPC1 | sLPKPHAVGCECTLcSA | 1.776552 | Control(minimal) | control 11 |
| TRPC1 | sLPKPHAVGCECTLcSA | 1.776552 | Control(minimal) | control 11 |
| TTN | PGKPQNPRVTDTtRT | 1.786878 | TBI(extreme) | TBI 5 |
| TTN | PGKPQNPRVTDTtRT | 1.786878 | TBI(extreme) | TBI 5 |
| TTN | PGKPQNPRVTDTtRT | 1.786878 | TBI(extreme) | TBI 5 |
| TTN | PGKPQNPRVTDTtRT | 1.786878 | TBI(extreme) | TBI 5 |
| TTN | PGKPQNPRVTDTtRT | 1.786878 | TBI(extreme) | TBI 5 |
| TTN | PGKPQNPRVTDTtRT | 1.786878 | TBI(extreme) | TBI 5 |
| TTN | PGKPQNPRVTDTtRT | 1.786878 | TBI(extreme) | TBI 5 |
| TTN | PGKPQNPRVTDTtRT | 1.786878 | TBI(extreme) | TBI 5 |
| TTN | PGKPQNPRVTDTtRT | 1.786878 | TBI(extreme) | TBI 5 |
| TTN | PGKPQNPRVTDTtRT | 1.786878 | TBI(extreme) | TBI 5 |
| TTN | PGKPQNPRVTDTtRT | 1.786878 | TBI(extreme) | TBI 5 |
| TTN | LECRVSGSAPISVG | 3.085285 | TBI(extreme) | TBI 9 |
| TTN | LECRVSGSAPISVG | 3.085285 | TBI(extreme) | TBI 9 |
| TTN | LECRVSGSAPISVG | 3.085285 | TBI(extreme) | TBI 9 |
| TTN | SFTKKLTKMDKVLGSS | 3.085285 | TBI(extreme) | TBI 9 |
| TTN | ATAINKAGRDTtR | 3.454229 | TBI(extreme) | TBI 30 |
| TTN | YEEREyERY | 3.454229 | TBI(extreme) | TBI 30 |
| TTN | YEEREyERY | 3.454229 | TBI(extreme) | TBI 30 |
| TTN | YEEREyERY | 3.454229 | TBI(extreme) | TBI 30 |
| TTN | ATAINKAGRDTtR | 1.734893 | TBI(extreme) | TBI 30 |
| TTN | ATAINKAGRDTtR | 1.734893 | TBI(extreme) | TBI 30 |
| TTN | ATAINKAGRDTtR | 1.734893 | TBI(extreme) | TBI 30 |
| TTN | ATAINKAGRDTtR | 1.734893 | TBI(extreme) | TBI 30 |
| TTN | ATAINKAGRDTtR | 1.734893 | TBI(extreme) | TBI 30 |
| TTN | ATAINKAGRDTtR | 1.734893 | TBI(extreme) | TBI 30 |
| TTN | ATAINKAGRDTtR | 1.734893 | TBI(extreme) | TBI 30 |
| TTN | ATAINKAGRDTtR | 1.734893 | TBI(extreme) | TBI 30 |
| TTN | ATAINKAGRDTtR | 1.734893 | TBI(extreme) | TBI 30 |
| TTN | ATAINKAGRDTtR | 1.734893 | TBI(extreme) | TBI 30 |
| TTN | ATAINKAGRDTtR | 1.734893 | TBI(extreme) | TBI 30 |
| TTN | ATAINKAGRDTtR | 1.734893 | TBI(extreme) | TBI 30 |
| TTN | YEEREyERY | 1.734893 | TBI(extreme) | TBI 30 |
| TTN | YEEREyERY | 1.719335 | TBI(extreme) | TBI 30 |
| TTN | YEEREyERY | 1.719335 | TBI(extreme) | TBI 30 |
| TTN | YEEREyERY | 1.719335 | TBI(extreme) | TBI 30 |
| TTN | YEEREyERY | 1.719335 | TBI(extreme) | TBI 30 |
| TTN | YEEREyERY | 1.719335 | TBI(extreme) | TBI 30 |
| TTN | ATAINKAGRDTtR | 2.059848 | TBI(extreme) | TBI 47 |
| TTN | ATAINKAGRDTtR | 2.059848 | TBI(extreme) | TBI 47 |
| TTN | ATAINKAGRDTtR | 2.059848 | TBI(extreme) | TBI 47 |
| TTN | ATAINKAGRDTtR | 2.059848 | TBI(extreme) | TBI 47 |
| TTN | ATAINKAGRDTtR | 2.059848 | TBI(extreme) | TBI 47 |
| TTN | ATAINKAGRDTtR | 2.059848 | TBI(extreme) | TBI 47 |
| TTN | ATAINKAGRDTtR | 2.059848 | TBI(extreme) | TBI 47 |
| TTN | ATAINKAGRDTtR | 2.059848 | TBI(extreme) | TBI 47 |
| TTN | ATAINKAGRDtTR | 2.059848 | TBI(extreme) | TBI 47 |
| TTN | ATAINKAGRDTtR | 2.059848 | TBI(extreme) | TBI 47 |
| TTN | ATAINKAGRDtTR | 2.059848 | TBI(extreme) | TBI 47 |
| TTN | ATAINKAGRDTtR | 2.059848 | TBI(extreme) | TBI 47 |
| TTN | ATAINKAGRDtTR | 2.059848 | TBI(extreme) | TBI 47 |
| TTN | ATAINKAGRDTtR | 2.059848 | TBI(extreme) | TBI 47 |
| TTN | ATAINKAGRDtTR | 2.059848 | TBI(extreme) | TBI 47 |
| TTN | ATAINKAGRDTtR | 2.059848 | TBI(extreme) | TBI 47 |
| TTN | ATAINKAGRDtTR | 2.059848 | TBI(extreme) | TBI 47 |
| TTN | ATAINKAGRDTtR | 2.059848 | TBI(extreme) | TBI 47 |
| TTN | ATAINKAGRDtTR | 2.059848 | TBI(extreme) | TBI 47 |
| TTN | ATAINKAGRDTtR | 2.059848 | TBI(extreme) | TBI 47 |
| TTN | LLEGLTYVFRVcAENAAG | 1.717102 | Control(minimal) | control 8 |
| TTN | LLEGLTYVFRVcAENAAG | 1.717102 | Control(minimal) | control 8 |
| TTN | LLEGLTYVFRVcAENAAG | 1.717102 | Control(minimal) | control 8 |
| TTN | LLEGLTYVFRVcAENAAG | 1.717102 | Control(minimal) | control 8 |
| TTN | LLEGLTYVFRVcAENAAG | 1.717102 | Control(minimal) | control 8 |
| TTN | LLEGLTYVFRVcAENAAG | 1.717102 | Control(minimal) | control 8 |
| TTN | HCKATNEVGSDtCsC | 2.721325 | Control(minimal) | control 9 |
| TTN | KPVYDLRVKS | 2.721325 | Control(minimal) | control 9 |
| TTN | KPVYDLRVKS | 2.721325 | Control(minimal) | control 9 |
| TTN | KPVYDLRVKS | 2.721325 | Control(minimal) | control 9 |
| TTN | GPPCVsKPLVAKDPFG | 1.774954 | Control(minimal) | control 9 |
| TTN | GPPCVsKPLVAKDPFG | 1.774954 | Control(minimal) | control 9 |
| TTN | GPPCVsKPLVAKDPFG | 1.774954 | Control(minimal) | control 9 |
| TTN | GPPCVsKPLVAKDPFG | 1.774954 | Control(minimal) | control 9 |
| TTN | GPPCVsKPLVAKDPFG | 1.774954 | Control(minimal) | control 9 |
| TTN | GPPCVsKPLVAKDPFG | 1.774954 | Control(minimal) | control 9 |
| TTN | GPPCVsKPLVAKDPFG | 1.774954 | Control(minimal) | control 9 |
| TTN | GPPCVsKPLVAKDPFG | 1.774954 | Control(minimal) | control 9 |
| TTN | GPPCVsKPLVAKDPFG | 1.774954 | Control(minimal) | control 9 |
| TTN | GPPCVsKPLVAKDPFG | 1.774954 | Control(minimal) | control 9 |
| TTN | GPPCVsKPLVAKDPFG | 1.774954 | Control(minimal) | control 9 |
| TTN | HCKATNEVGSDtCsC | 2.721325 | Control(minimal) | control 11 |
| TTN | KPVYDLRVKS | 2.721325 | Control(minimal) | control 11 |
| TTN | KPVYDLRVKS | 2.721325 | Control(minimal) | control 11 |
| TTN | KPVYDLRVKS | 2.721325 | Control(minimal) | control 11 |
| TTN | cGCMATHLLKEPPTF | 3.270761 | Control(minimal) | control 12 |
| TTN | CGcMATHLLKEPPTF | 3.270761 | Control(minimal) | control 12 |
| TTN | CGcMATHLLKEPPTF | 3.270761 | Control(minimal) | control 12 |
| TTN | CGcMATHLLKEPPTF | 3.270761 | Control(minimal) | control 12 |
| TTN | CGcMATHLLKEPPTF | 3.270761 | Control(minimal) | control 12 |
| TTN | KVTSLMEGcDYQFR | 3.270761 | Control(minimal) | control 12 |
| TTN | cGCMATHLLKEPPTF | 1.719926 | Control(minimal) | control 12 |
| TTN | cGCMATHLLKEPPTF | 1.719926 | Control(minimal) | control 12 |
| TTN | cGCMATHLLKEPPTF | 1.719926 | Control(minimal) | control 12 |
| TTN | cGCMATHLLKEPPTF | 1.719926 | Control(minimal) | control 12 |
| TTN | cGCMATHLLKEPPTF | 1.719926 | Control(minimal) | control 12 |
| TTN | cGCMATHLLKEPPTF | 1.719926 | Control(minimal) | control 12 |
| TTN | cGCMATHLLKEPPTF | 1.719926 | Control(minimal) | control 12 |
| TTN | CGPGEPAYVDEPVNMs | 1.894938 | Control(minimal) | control 13 |
| TTN | CGPGEPAYVDEPVNMs | 1.894938 | Control(minimal) | control 13 |
| TTN | CGPGEPAYVDEPVNMs | 1.894938 | Control(minimal) | control 13 |
| TTN | CGPGEPAYVDEPVNMs | 1.894938 | Control(minimal) | control 13 |
| TTN | CGPGEPAYVDEPVNMs | 1.894938 | Control(minimal) | control 13 |
| TTN | CGPGEPAYVDEPVNMs | 1.894938 | Control(minimal) | control 13 |
| TTN | CGPGEPAYVDEPVNMs | 1.894938 | Control(minimal) | control 13 |
| TTN | CGPGEPAYVDEPVNMs | 1.894938 | Control(minimal) | control 13 |
| TTN | CGPGEPAYVDEPVNMs | 1.894938 | Control(minimal) | control 13 |
| TTN | CGPGEPAYVDEPVNMs | 1.894938 | Control(minimal) | control 13 |
| TTN | CGPGEPAYVDEPVNMs | 1.894938 | Control(minimal) | control 13 |
| TTN | CGPGEPAYVDEPVNMs | 1.894938 | Control(minimal) | control 13 |
| TTN | CGPGEPAYVDEPVNMs | 1.894938 | Control(minimal) | control 13 |
| TTN | EmKELFsEGE | 1.754479 | Control(minimal) | control 13 |
| TTN | EmKELFsEGE | 1.754479 | Control(minimal) | control 13 |
| TTN | EmKELFsEGE | 1.754479 | Control(minimal) | control 13 |
| USH2A | HVGCTNSsWVLLYTA | 3.320705 | TBI(extreme) | TBI 6 |
| USH2A | HVGCtNSSWVLLYTA | 3.320705 | TBI(extreme) | TBI 6 |
| USH2A | QFLLSAcTHVGctNS | 3.320705 | TBI(extreme) | TBI 6 |
| USH2A | HVGCTNSsWVLLYTA | 1.852045 | TBI(extreme) | TBI 6 |
| USH2A | HVGCTNSsWVLLYTA | 1.852045 | TBI(extreme) | TBI 6 |
| USH2A | HVGCTNSsWVLLYTA | 1.852045 | TBI(extreme) | TBI 6 |
| USH2A | HVGCTNSsWVLLYTA | 1.852045 | TBI(extreme) | TBI 6 |
| USH2A | HVGCtNSSWVLLYTA | 1.843996 | TBI(extreme) | TBI 6 |
| USH2A | HVGCtNSSWVLLYTA | 1.843996 | TBI(extreme) | TBI 6 |
| USH2A | HVGCtNSSWVLLYTA | 1.843996 | TBI(extreme) | TBI 6 |
| USH2A | HVGCtNSSWVLLYTA | 1.843996 | TBI(extreme) | TBI 6 |
| USH2A | SAIDVcKPcDcDTVGTRN | 1.749508 | TBI(extreme) | TBI 47 |
| USH2A | SAIDVcKPcDcDTVGTRN | 1.749508 | TBI(extreme) | TBI 47 |
| USH2A | SAIDVcKPcDcDTVGTRN | 1.749508 | TBI(extreme) | TBI 47 |
| USH2A | SAIDVcKPcDcDTVGTRN | 1.749508 | TBI(extreme) | TBI 47 |
| USH2A | SAIDVcKPcDcDTVGTRN | 1.749508 | TBI(extreme) | TBI 47 |
| USH2A | PyTEYMFRLVASNG | 5.264782 | Control(minimal) | control 6 |
| USH2A | RNcELcKDYFFRQ | 5.264782 | Control(minimal) | control 6 |
| USH2A | TSLAFtQVDLLLGLS | 5.264782 | Control(minimal) | control 6 |
| USH2A | PyTEYMFRLVASNG | 1.858105 | Control(minimal) | control 6 |
| USH2A | PyTEYMFRLVASNG | 1.858105 | Control(minimal) | control 6 |
| USH2A | PyTEYMFRLVASNG | 1.858105 | Control(minimal) | control 6 |
| USH2A | PyTEYMFRLVASNG | 1.858105 | Control(minimal) | control 6 |
| USH2A | RNcELcKDYFFRQ | 1.826988 | Control(minimal) | control 6 |
| USH2A | RNcELcKDYFFRQ | 1.826988 | Control(minimal) | control 6 |
| USH2A | RNcELcKDYFFRQ | 1.826988 | Control(minimal) | control 6 |
| USH2A | RNcELcKDYFFRQ | 1.826988 | Control(minimal) | control 6 |
| USH2A | RNcELcKDYFFRQ | 1.826988 | Control(minimal) | control 6 |
| USH2A | TSLAFtQVDLLLGLS | 1.826988 | Control(minimal) | control 6 |
| USH2A | TIVACSGGNGYLGGcTES | 1.776259 | Control(minimal) | control 9 |
| USH2A | TIVACSGGNGYLGGcTES | 1.776259 | Control(minimal) | control 9 |
| USH2A | TIVACSGGNGYLGGcTES | 1.776259 | Control(minimal) | control 9 |
| USH2A | TIVACSGGNGYLGGcTES | 1.776259 | Control(minimal) | control 11 |
| USH2A | TIVACSGGNGYLGGcTES | 1.776259 | Control(minimal) | control 11 |
| USH2A | TIVACSGGNGYLGGcTES | 1.776259 | Control(minimal) | control 11 |
| VWF | MSmGCVSGCLCPPGMVR | 1.911606 | TBI(extreme) | TBI 2 |
| VWF | MSmGCVSGCLCPPGMVR | 1.911606 | TBI(extreme) | TBI 2 |
| VWF | MSmGCVSGCLCPPGMVR | 1.911606 | TBI(extreme) | TBI 2 |
| VWF | MSMGcVSGCLCPPGMVR | 1.911606 | TBI(extreme) | TBI 2 |
| VWF | MSmGCVSGCLCPPGMVR | 1.911606 | TBI(extreme) | TBI 2 |
| VWF | MSMGcVSGCLCPPGMVR | 1.911606 | TBI(extreme) | TBI 2 |
| VWF | MSmGCVSGCLCPPGMVR | 1.911606 | TBI(extreme) | TBI 2 |
| VWF | MSMGcVSGCLCPPGMVR | 1.911606 | TBI(extreme) | TBI 2 |
| VWF | MSmGCVSGCLCPPGMVR | 1.911606 | TBI(extreme) | TBI 2 |
| VWF | MSMGcVSGCLCPPGMVR | 1.911606 | TBI(extreme) | TBI 2 |
| VWF | MSmGCVSGCLCPPGMVR | 1.911606 | TBI(extreme) | TBI 2 |
| VWF | MSMGcVSGCLCPPGMVR | 1.911606 | TBI(extreme) | TBI 2 |
| VWF | MSmGCVSGCLCPPGMVR | 1.911606 | TBI(extreme) | TBI 2 |
| VWF | MSMGcVSGCLCPPGMVR | 1.911606 | TBI(extreme) | TBI 2 |
| VWF | MSmGCVSGCLCPPGMVR | 1.911606 | TBI(extreme) | TBI 2 |
| VWF | MSMGcVSGCLCPPGMVR | 1.911606 | TBI(extreme) | TBI 2 |
| VWF | MSmGCVSGCLCPPGMVR | 1.911606 | TBI(extreme) | TBI 2 |
| VWF | MSMGcVSGCLCPPGMVR | 1.911606 | TBI(extreme) | TBI 2 |
| VWF | MSmGCVSGCLCPPGMVR | 1.911606 | TBI(extreme) | TBI 2 |
| VWF | MSMGcVSGCLCPPGMVR | 1.911606 | TBI(extreme) | TBI 2 |
| VWF | MSmGCVSGCLCPPGMVR | 1.911606 | TBI(extreme) | TBI 2 |
| VWF | MSMGcVSGCLCPPGMVR | 1.817442 | TBI(extreme) | TBI 2 |
| VWF | MSMGcVSGCLCPPGMVR | 1.817442 | TBI(extreme) | TBI 2 |
| VWF | MSMGcVSGCLCPPGMVR | 1.817442 | TBI(extreme) | TBI 2 |
| VWF | MSMGcVSGCLCPPGMVR | 1.817442 | TBI(extreme) | TBI 2 |
| VWF | MSMGcVSGCLCPPGMVR | 1.817442 | TBI(extreme) | TBI 2 |
| VWF | MSMGcVSGCLCPPGMVR | 1.817442 | TBI(extreme) | TBI 2 |
| VWF | MSMGcVSGCLCPPGMVR | 1.817442 | TBI(extreme) | TBI 2 |
| VWF | MSMGcVSGCLCPPGMVR | 1.817442 | TBI(extreme) | TBI 2 |
| VWF | MSMGcVSGCLCPPGMVR | 1.817442 | TBI(extreme) | TBI 2 |
| VWF | MSMGcVSGCLCPPGMVR | 1.817442 | TBI(extreme) | TBI 2 |
| VWF | MSMGcVSGCLCPPGMVR | 1.817442 | TBI(extreme) | TBI 2 |
| VWF | NsQVGTRKAS | 3.016473 | TBI(extreme) | TBI 10 |
| VWF | PCEDScRsGFT | 3.016473 | TBI(extreme) | TBI 10 |
| VWF | PCEDScRsGFT | 3.016473 | TBI(extreme) | TBI 10 |
| VWF | PCEDScRsGFT | 3.016473 | TBI(extreme) | TBI 10 |
| VWF | PCEDScRsGFT | 3.016473 | TBI(extreme) | TBI 10 |
| VWF | PCEDScRsGFT | 3.016473 | TBI(extreme) | TBI 10 |
| VWF | PCEDScRsGFT | 3.016473 | TBI(extreme) | TBI 10 |
| VWF | PCEDScRsGFT | 3.016473 | TBI(extreme) | TBI 10 |
| VWF | PCEDScRsGFT | 3.016473 | TBI(extreme) | TBI 10 |
| VWF | VQLLESGGGVVPPGm | 3.016473 | TBI(extreme) | TBI 10 |
| VWF | CLCLsPSVSGCLCVC | 1.749922 | TBI(extreme) | TBI 10 |
| VWF | CLCLsPSVSGCLCVC | 1.749922 | TBI(extreme) | TBI 10 |
| VWF | CLCLsPSVSGCLCVC | 1.749922 | TBI(extreme) | TBI 10 |
| VWF | DTCscESIGDCACFCDT | 1.730736 | Control(minimal) | control 13 |
| VWF | DTCscESIGDCACFCDT | 1.730736 | Control(minimal) | control 13 |
| VWF | DTCscESIGDCACFCDT | 1.730736 | Control(minimal) | control 13 |
| VWF | DTCscESIGDCACFCDT | 1.730736 | Control(minimal) | control 13 |
| VWF | DTCscESIGDCACFCDT | 1.730736 | Control(minimal) | control 13 |
| VWF | DTCscESIGDCACFCDT | 1.730736 | Control(minimal) | control 13 |
| VWF | DTCscESIGDCACFCDT | 1.730736 | Control(minimal) | control 13 |
| VWF | DTCscESIGDCACFCDT | 1.730736 | Control(minimal) | control 13 |
| VWF | DTCscESIGDCACFCDT | 1.730736 | Control(minimal) | control 13 |
| VWF | DTCscESIGDCACFCDT | 1.730736 | Control(minimal) | control 13 |
| VWF | DTCscESIGDCACFCDT | 1.730736 | Control(minimal) | control 13 |
| VWF | DTCscESIGDCACFCDT | 1.730736 | Control(minimal) | control 13 |
| VWF | DTCscESIGDCACFCDT | 1.730736 | Control(minimal) | control 13 |
| WFDC3 | GDELCPAEQKcCTTGCGR | 1.882151 | TBI(extreme) | TBI 5 |
| WFDC3 | GDELCPAEQKcCTTGCGR | 1.882151 | TBI(extreme) | TBI 5 |
| WFDC3 | GDELCPAEQKcCTTGCGR | 1.882151 | TBI(extreme) | TBI 5 |
| WFDC3 | GDELCPAEQKcCTTGCGR | 1.882151 | TBI(extreme) | TBI 5 |
| WFDC3 | GDELCPAEQKcCTTGCGR | 1.882151 | TBI(extreme) | TBI 5 |
| WFDC3 | GDELCPAEQKcCTTGCGR | 1.882151 | TBI(extreme) | TBI 5 |
| WFDC3 | GDELCPAEQKcCTTGCGR | 1.882151 | TBI(extreme) | TBI 5 |
| WFDC3 | ccsTGCGRTcLGDIEGG | 1.941015 | TBI(extreme) | TBI 8 |
| WFDC3 | ccsTGCGRTcLGDIEGG | 1.941015 | TBI(extreme) | TBI 8 |
| WFDC3 | ccsTGCGRTcLGDIEGG | 1.941015 | TBI(extreme) | TBI 8 |
| WFDC3 | ccStGCGRTcLGDIEGG | 1.941015 | TBI(extreme) | TBI 8 |
| WFDC3 | ccsTGCGRTcLGDIEGG | 1.941015 | TBI(extreme) | TBI 8 |
| WFDC3 | ccStGCGRTcLGDIEGG | 1.941015 | TBI(extreme) | TBI 8 |
| WFDC3 | ccsTGCGRTcLGDIEGG | 1.941015 | TBI(extreme) | TBI 8 |
| WFDC3 | ccStGCGRTcLGDIEGG | 1.941015 | TBI(extreme) | TBI 8 |
| WFDC3 | ccsTGCGRTcLGDIEGG | 1.941015 | TBI(extreme) | TBI 8 |
| WFDC3 | ccStGCGRTcLGDIEGG | 1.941015 | TBI(extreme) | TBI 8 |
| WFDC3 | ccsTGCGRTcLGDIEGG | 1.941015 | TBI(extreme) | TBI 8 |
| WFDC3 | ccStGCGRTcLGDIEGG | 1.941015 | TBI(extreme) | TBI 8 |
| WFDC3 | ccsTGCGRTcLGDIEGG | 1.941015 | TBI(extreme) | TBI 8 |
| WFDC3 | ccStGCGRTcLGDIEGG | 1.941015 | TBI(extreme) | TBI 8 |
| WFDC3 | ccsTGCGRTcLGDIEGG | 1.941015 | TBI(extreme) | TBI 8 |
| WFDC3 | ccStGCGRTcLGDIEGG | 1.941015 | TBI(extreme) | TBI 8 |
| WFDC3 | ccsTGCGRTcLGDIEGG | 1.941015 | TBI(extreme) | TBI 8 |
| WFDC3 | ccStGCGRTcLGDIEGG | 1.851512 | TBI(extreme) | TBI 8 |
| WFDC3 | ccStGCGRTcLGDIEGG | 1.851512 | TBI(extreme) | TBI 8 |
| WFDC3 | ccStGCGRTcLGDIEGG | 1.851512 | TBI(extreme) | TBI 8 |
| WFDC3 | ccStGCGRTcLGDIEGG | 1.851512 | TBI(extreme) | TBI 8 |
| WFDC3 | ccStGCGRTcLGDIEGG | 1.851512 | TBI(extreme) | TBI 8 |
| WFDC3 | ccStGCGRTcLGDIEGG | 1.851512 | TBI(extreme) | TBI 8 |
| WFDC3 | ccStGCGRTcLGDIEGG | 1.851512 | TBI(extreme) | TBI 8 |
| WFDC3 | ccStGCGRTcLGDIEGG | 1.851512 | TBI(extreme) | TBI 8 |
| WFDC3 | ccStGCGRTcLGDIEGG | 1.851512 | TBI(extreme) | TBI 8 |
| WFDC3 | LcDGDAscPQGHKcCS | 1.879985 | Control(minimal) | control 9 |
| WFDC3 | LcDGDAscPQGHKcCS | 1.879985 | Control(minimal) | control 9 |
| WFDC3 | LcDGDAscPQGHKcCS | 1.879985 | Control(minimal) | control 9 |
| WFDC3 | LcDGDAscPQGHKcCS | 1.879985 | Control(minimal) | control 9 |
| WFDC3 | LcDGDAscPQGHKcCS | 1.879985 | Control(minimal) | control 9 |
| WFDC3 | LcDGDAscPQGHKcCS | 1.879985 | Control(minimal) | control 9 |
| WFDC3 | LcDGDAscPQGHKcCS | 1.879985 | Control(minimal) | control 9 |
| WFDC3 | LcDGDAscPQGHKcCS | 1.879985 | Control(minimal) | control 9 |
| WFDC3 | LcDGDAscPQGHKcCS | 1.879985 | Control(minimal) | control 9 |
| ZNF268 | KPYGcNEcGK | 2.772598 | TBI(extreme) | TBI 2 |
| ZNF268 | YEcSECGKAFcW | 2.772598 | TBI(extreme) | TBI 2 |
| ZNF268 | AKSFEcTtF | 2.72099 | TBI(extreme) | TBI 5 |
| ZNF268 | AKSFEcTtF | 2.72099 | TBI(extreme) | TBI 5 |
| ZNF268 | AKSFEcTtF | 2.72099 | TBI(extreme) | TBI 5 |
| ZNF268 | AKSFEcTtF | 2.72099 | TBI(extreme) | TBI 5 |
| ZNF268 | AKSFEcTtF | 2.72099 | TBI(extreme) | TBI 5 |
| ZNF268 | AKSFEcTtF | 2.72099 | TBI(extreme) | TBI 5 |
| ZNF268 | AKSFEcTtF | 2.72099 | TBI(extreme) | TBI 5 |
| ZNF268 | AKSFEcTtF | 2.72099 | TBI(extreme) | TBI 5 |
| ZNF268 | AKSFEcTtF | 2.72099 | TBI(extreme) | TBI 5 |
| ZNF268 | AKSFEcTtF | 2.72099 | TBI(extreme) | TBI 5 |
| ZNF268 | AKSFEcTtF | 2.72099 | TBI(extreme) | TBI 5 |
| ZNF268 | FEcSEcQKAF | 2.72099 | TBI(extreme) | TBI 5 |
| ZNF268 | KPYGcIQcGK | 2.210106 | TBI(extreme) | TBI 9 |
| ZNF268 | KPYGcIQcGK | 2.210106 | TBI(extreme) | TBI 9 |
| ZNF268 | KPYGcNEcGK | 2.210106 | TBI(extreme) | TBI 9 |
| ZNF268 | KPYGcNEcGK | 2.210106 | TBI(extreme) | TBI 9 |
| ZNF268 | KPYGcNEcGK | 2.210106 | TBI(extreme) | TBI 9 |
| ZNF268 | KPYGcNEcGK | 2.210106 | TBI(extreme) | TBI 9 |
| ZNF268 | KPYGcNEcGK | 2.210106 | TBI(extreme) | TBI 9 |
| ZNF268 | KPYGcNEcGK | 2.210106 | TBI(extreme) | TBI 9 |
| ZNF268 | KPYGcNEcGK | 2.210106 | TBI(extreme) | TBI 9 |
| ZNF268 | KPYGcNEcGK | 2.210106 | TBI(extreme) | TBI 9 |
| ZNF268 | KPYGcNEcGK | 2.210106 | TBI(extreme) | TBI 9 |
| ZNF268 | KPYGcNEcGK | 2.210106 | TBI(extreme) | TBI 9 |
| ZNF268 | KPYGcNEcGK | 2.210106 | TBI(extreme) | TBI 9 |
| ZNF268 | KPYGcNEcGK | 2.210106 | TBI(extreme) | TBI 9 |
| ZNF268 | KPYGcNEcGK | 2.210106 | TBI(extreme) | TBI 9 |
| ZNF268 | KPYGcNEcGK | 2.210106 | TBI(extreme) | TBI 9 |
| ZNF268 | KPYGcNEcGK | 2.210106 | TBI(extreme) | TBI 9 |
| ZNF268 | KPYGcNEcGK | 2.210106 | TBI(extreme) | TBI 9 |
| ZNF268 | KPYGcDEcGK | 2.069417 | TBI(extreme) | TBI 9 |
| ZNF268 | KPYGcNEcGK | 2.069417 | TBI(extreme) | TBI 9 |
| ZNF268 | IVHERtHAGVNPYKcS | 1.802043 | Control(minimal) | control 7 |
| ZNF268 | IVHERtHAGVNPYKcS | 1.802043 | Control(minimal) | control 7 |
| ZNF268 | IVHERtHAGVNPYKcS | 1.802043 | Control(minimal) | control 7 |
| ZNF268 | IVHERtHAGVNPYKcS | 1.802043 | Control(minimal) | control 7 |
| ZNF268 | IVHERtHAGVNPYKcS | 1.802043 | Control(minimal) | control 7 |
| ZNF268 | IVHERtHAGVNPYKcS | 1.802043 | Control(minimal) | control 7 |
| ZNF268 | IVHERtHAGVNPYKcS | 1.802043 | Control(minimal) | control 7 |
| ZNF268 | IVHERtHAGVNPYKcS | 1.802043 | Control(minimal) | control 7 |
| ZNF268 | IVHERtHAGVNPYKcS | 1.802043 | Control(minimal) | control 7 |
| ZNF268 | IVHERtHAGVNPYKcS | 1.802043 | Control(minimal) | control 7 |
| ZNF268 | IVHERtHAGVNPYKcS | 1.802043 | Control(minimal) | control 7 |
| ZNF268 | IVHERtHAGVNPYKcS | 1.802043 | Control(minimal) | control 7 |
| ZNF268 | IVHERtHAGVNPYKcS | 1.802043 | Control(minimal) | control 7 |
| ZNF268 | KPYGcNEcGKDFSSK | 1.707233 | Control(minimal) | control 12 |
| ZNF268 | KPYGcNEcGKDFSSK | 1.707233 | Control(minimal) | control 12 |
| ZNF268 | KPYGcNEcGKDFSSK | 1.707233 | Control(minimal) | control 12 |
| ZNF268 | KPYGcNEcGKDFSSK | 1.707233 | Control(minimal) | control 12 |
| ZNF268 | KPYGcNEcGKDFSSK | 1.707233 | Control(minimal) | control 12 |
| ZNF268 | KPYGcNEcGKDFSSK | 1.707233 | Control(minimal) | control 12 |
| ZNF268 | KPYGcNEcGKDFSSK | 1.707233 | Control(minimal) | control 12 |
| ZNF268 | KPYGcNEcGKDFSSK | 1.707233 | Control(minimal) | control 12 |
| ZNF268 | KPYGcNEcGKDFSSK | 1.707233 | Control(minimal) | control 12 |
| ZNF268 | KPYGcNEcGKDFSSK | 1.707233 | Control(minimal) | control 12 |
| ZNF268 | KPYGcNEcGKDFSSK | 1.707233 | Control(minimal) | control 12 |
| ZNF268 | KPYGcNEcGKDFSSK | 1.707233 | Control(minimal) | control 12 |
| ZNF268 | KPYGcNEcGKDFSSK | 1.707233 | Control(minimal) | control 12 |
| ZNF268 | KPYGcNEcGKDFSSK | 1.707233 | Control(minimal) | control 12 |
| ZNF268 | KPYGcNEcGKDFSSK | 1.707233 | Control(minimal) | control 12 |
| ZNRF3 | SSsDSVVDcTEVSNQGV | 1.954196 | Control(minimal) | control 9 |
| ZNRF3 | SSsDSVVDcTEVSNQGV | 1.954196 | Control(minimal) | control 9 |
| ZNRF3 | SSsDSVVDcTEVSNQGV | 1.954196 | Control(minimal) | control 9 |
| ZNRF3 | SSsDSVVDcTEVSNQGV | 1.954196 | Control(minimal) | control 9 |
| ZNRF3 | SSsDSVVDcTEVSNQGV | 1.954196 | Control(minimal) | control 9 |
| ZNRF3 | SSsDSVVDcTEVSNQGV | 1.954196 | Control(minimal) | control 9 |
| ZNRF3 | SsSDSVVDcTEVSNQGV | 1.954196 | Control(minimal) | control 9 |
| ZNRF3 | SsSDSVVDcTEVSNQGV | 1.954196 | Control(minimal) | control 9 |
| ZNRF3 | SsSDSVVDcTEVSNQGV | 1.954196 | Control(minimal) | control 9 |
| ZNRF3 | SsSDSVVDcTEVSNQGV | 1.954196 | Control(minimal) | control 9 |
| ZNRF3 | SsSDSVVDcTEVSNQGV | 1.954196 | Control(minimal) | control 9 |
| ZNRF3 | SsSDSVVDcTEVSNQGV | 1.945849 | Control(minimal) | control 9 |
| ZNRF3 | SSsDSVVDcTEVSNQGV | 1.954196 | Control(minimal) | control 11 |
| ZNRF3 | SSsDSVVDcTEVSNQGV | 1.954196 | Control(minimal) | control 11 |
| ZNRF3 | SSsDSVVDcTEVSNQGV | 1.954196 | Control(minimal) | control 11 |
| ZNRF3 | SSsDSVVDcTEVSNQGV | 1.954196 | Control(minimal) | control 11 |
| ZNRF3 | SSsDSVVDcTEVSNQGV | 1.954196 | Control(minimal) | control 11 |
| ZNRF3 | SSsDSVVDcTEVSNQGV | 1.954196 | Control(minimal) | control 11 |
| ZNRF3 | SsSDSVVDcTEVSNQGV | 1.954196 | Control(minimal) | control 11 |
| ZNRF3 | SsSDSVVDcTEVSNQGV | 1.954196 | Control(minimal) | control 11 |
| ZNRF3 | SsSDSVVDcTEVSNQGV | 1.954196 | Control(minimal) | control 11 |
| ZNRF3 | SsSDSVVDcTEVSNQGV | 1.954196 | Control(minimal) | control 11 |
| ZNRF3 | SsSDSVVDcTEVSNQGV | 1.954196 | Control(minimal) | control 11 |
| ZNRF3 | SsSDSVVDcTEVSNQGV | 1.945849 | Control(minimal) | control 11 |
| ZNRF3 | SsSDSVVDcTEVSNQGV | 2.148132 | Control(minimal) | control 13 |
| ZNRF3 | SsSDSVVDcTEVSNQGV | 2.148132 | Control(minimal) | control 13 |
| ZNRF3 | SsSDSVVDcTEVSNQGV | 2.148132 | Control(minimal) | control 13 |
| ZNRF3 | SsSDSVVDcTEVSNQGV | 2.148132 | Control(minimal) | control 13 |
| ZNRF3 | SsSDSVVDcTEVSNQGV | 2.148132 | Control(minimal) | control 13 |
| ZNRF3 | SsSDSVVDcTEVSNQGV | 2.148132 | Control(minimal) | control 13 |
| ZNRF3 | SSsDSVVDcTEVSNQGV | 2.148132 | Control(minimal) | control 13 |
| ZNRF3 | SSsDSVVDcTEVSNQGV | 2.148132 | Control(minimal) | control 13 |
| ZNRF3 | SSsDSVVDcTEVSNQGV | 2.148132 | Control(minimal) | control 13 |
| ZNRF3 | SSsDSVVDcTEVSNQGV | 2.148132 | Control(minimal) | control 13 |
| ZNRF3 | SSsDSVVDcTEVSNQGV | 2.148132 | Control(minimal) | control 13 |
| ZNRF3 | SSsDSVVDcTEVSNQGV | 2.058233 | Control(minimal) | control 13 |
