# Supplementary material for: Distinguishing and phenotype monitoring of traumatic brain injury and post-concussion syndrome including chronic migraine in serum of Iraq and Afghanistan war veterans
Source: PLoS One. 2019 Apr 26;14(4):e0215762. doi: 10.1371/journal.pone.0215762 (PMC6485717; doi:10.1371/journal.pone.0215762)
Supplement: S15 Table — (DOCX) [file pone.0215762.s041.docx]

**S15 Table. Proteins identified MSMS analysis TBI + CM (N=10) vs TBI (N=10) MS/MS results.**

| Symbol | Sequence_mod | Xcorr_mod | pathology | De-identified ID |
| --- | --- | --- | --- | --- |
| IGFN1 | EDSGILGKGNSTEWGNAL | 2.4123 | TBI | TBI 11 |
| IGFN1 | KtGPGGPGDPRGCEGV | 2.4123 | TBI | TBI 11 |
| DNAH9 | TEVGmAFARLEEGyESA | 1.95 | TBI | TBI 11 |
| DNAH9 | TEVGmAFARLEEGYEsA | 1.95 | TBI | TBI 11 |
| DNAH9 | TEVGmAFARLEEGyESA | 1.95 | TBI | TBI 11 |
| DNAH9 | TEVGmAFARLEEGYEsA | 1.95 | TBI | TBI 11 |
| DNAH9 | TEVGmAFARLEEGyESA | 1.95 | TBI | TBI 11 |
| DNAH9 | TEVGmAFARLEEGYEsA | 1.95 | TBI | TBI 11 |
| DNAH9 | TEVGmAFARLEEGyESA | 1.95 | TBI | TBI 11 |
| DNAH9 | TEVGmAFARLEEGYEsA | 1.95 | TBI | TBI 11 |
| DNAH9 | TEVGmAFARLEEGyESA | 1.95 | TBI | TBI 11 |
| DNAH9 | TEVGmAFARLEEGyESA | 1.95 | TBI | TBI 11 |
| DNAH9 | TEVGmAFARLEEGYEsA | 1.95 | TBI | TBI 11 |
| DNAH9 | TEVGmAFARLEEGYEsA | 1.9421 | TBI | TBI 11 |
| EBF4 | sLPSPTTAVPLLVPFTLN | 1.9177 | TBI | TBI 11 |
| EBF4 | sLPSPTTAVPLLVPFTLN | 1.9177 | TBI | TBI 11 |
| EBF4 | sLPSPTTAVPLLVPFTLN | 1.9177 | TBI | TBI 11 |
| FBN1 | ASNIEDQSEtEANVSLAS | 1.8922 | TBI | TBI 11 |
| FBN1 | ASNIEDQSEtEANVSLAS | 1.8922 | TBI | TBI 11 |
| FBN1 | ASNIEDQSEtEANVSLAS | 1.8922 | TBI | TBI 11 |
| FBN1 | ASNIEDQSEtEANVSLAS | 1.8922 | TBI | TBI 11 |
| FBN1 | ASNIEDQSEtEANVSLAS | 1.8922 | TBI | TBI 11 |
| SH2D7 | WRQEFPKLsQEAQPc | 1.879 | TBI | TBI 11 |
| SH2D7 | WRQEFPKLsQEAQPc | 1.879 | TBI | TBI 11 |
| SMYD3 | DCAMDAcINLGLLEEALF | 1.8228 | TBI | TBI 11 |
| SMYD3 | DCAMDAcINLGLLEEALF | 1.8228 | TBI | TBI 11 |
| SMYD3 | DCAMDAcINLGLLEEALF | 1.8228 | TBI | TBI 11 |
| SMYD3 | DCAMDAcINLGLLEEALF | 1.8228 | TBI | TBI 11 |
| SMYD3 | DCAMDAcINLGLLEEALF | 1.8228 | TBI | TBI 11 |
| SMYD3 | DCAMDAcINLGLLEEALF | 1.8228 | TBI | TBI 11 |
| SMYD3 | DCAMDAcINLGLLEEALF | 1.8228 | TBI | TBI 11 |
| SMYD3 | DCAMDAcINLGLLEEALF | 1.8228 | TBI | TBI 11 |
| SMYD3 | DCAMDAcINLGLLEEALF | 1.8228 | TBI | TBI 11 |
| SMYD3 | DCAMDAcINLGLLEEALF | 1.8228 | TBI | TBI 11 |
| SMYD3 | DCAMDAcINLGLLEEALF | 1.8228 | TBI | TBI 11 |
| SMYD3 | DCAMDAcINLGLLEEALF | 1.8228 | TBI | TBI 11 |
| ZBED4 | ALVGSsPHLPALHYDEP | 1.7561 | TBI | TBI 11 |
| ZBED4 | ALVGSsPHLPALHYDEP | 1.7561 | TBI | TBI 11 |
| ZBED4 | ALVGSsPHLPALHYDEP | 1.7561 | TBI | TBI 11 |
| ZBED4 | ALVGSsPHLPALHYDEP | 1.7561 | TBI | TBI 11 |
| ZBED4 | ALVGSsPHLPALHYDEP | 1.7561 | TBI | TBI 11 |
| ZBED4 | ALVGSsPHLPALHYDEP | 1.7561 | TBI | TBI 11 |
| ZBED4 | ALVGSsPHLPALHYDEP | 1.7561 | TBI | TBI 11 |
| EBF4 | FGVPTSATtEVSMTEIM | 1.8635 | TBI+CM | TBI 19 |
| EBF4 | FGVPTSATtEVSMTEIM | 1.8635 | TBI+CM | TBI 19 |
| EBF4 | FGVPTSATtEVSMTEIM | 1.8635 | TBI+CM | TBI 19 |
| EBF4 | FGVPTSATtEVSMTEIM | 1.8635 | TBI+CM | TBI 19 |
| EBF4 | FGVPTSATtEVSMTEIM | 1.8635 | TBI+CM | TBI 19 |
| EBF4 | FGVPTSATtEVSMTEIM | 1.8635 | TBI+CM | TBI 19 |
| EBF4 | FGVPTSATtEVSMTEIM | 1.8635 | TBI+CM | TBI 19 |
| EBF4 | FGVPTSATtEVSMTEIM | 1.8635 | TBI+CM | TBI 19 |
| EBF4 | FGVPTSAtTEVSMTEIM | 1.8635 | TBI+CM | TBI 19 |
| EBF4 | FGVPTSAtTEVSMTEIM | 1.8635 | TBI+CM | TBI 19 |
| EBF4 | FGVPTSAtTEVSMTEIM | 1.8635 | TBI+CM | TBI 19 |
| EBF4 | FGVPTSAtTEVSMTEIM | 1.8635 | TBI+CM | TBI 19 |
| EBF4 | FGVPTSAtTEVSMTEIM | 1.8635 | TBI+CM | TBI 19 |
| EBF4 | FGVPTSAtTEVSMTEIM | 1.8635 | TBI+CM | TBI 19 |
| EBF4 | FGVPTSAtTEVSMTEIM | 1.8635 | TBI+CM | TBI 19 |
| EBF4 | FGVPTSATtEVSMTEIM | 1.8635 | TBI+CM | TBI 19 |
| EBF4 | FGVPTSAtTEVSMTEIM | 1.8635 | TBI+CM | TBI 19 |
| EBF4 | FGVPTSAtTEVSMTEIM | 1.8635 | TBI+CM | TBI 19 |
| EBF4 | FGVPTSAtTEVSMTEIM | 1.8635 | TBI+CM | TBI 19 |
| EBF4 | FGVPTSAtTEVSMTEIM | 1.8635 | TBI+CM | TBI 19 |
| EBF4 | FGVPTSAtTEVSMTEIM | 1.8635 | TBI+CM | TBI 19 |
| EBF4 | FGVPTSATtEVSMTEIM | 1.8408 | TBI+CM | TBI 19 |
| EBF4 | FGVPTSATtEVSMTEIM | 1.8408 | TBI+CM | TBI 19 |
| EBF4 | FGVPTSATtEVSMTEIM | 1.8408 | TBI+CM | TBI 19 |
| EBF4 | FGVPTSATtEVSMTEIM | 1.8408 | TBI+CM | TBI 19 |
| EBF4 | FGVPTSATtEVSMTEIM | 1.8408 | TBI+CM | TBI 19 |
| EBF4 | FGVPTSATtEVSMTEIM | 1.8408 | TBI+CM | TBI 19 |
| EBF4 | FGVPTSATtEVSMTEIM | 1.8408 | TBI+CM | TBI 19 |
| EBF4 | FGVPTSATtEVSMTEIM | 1.8408 | TBI+CM | TBI 19 |
| EBF4 | FGVPTSATtEVSMTEIM | 1.8408 | TBI+CM | TBI 19 |
| EBF4 | FGVPTSATtEVSMTEIM | 1.8408 | TBI+CM | TBI 19 |
| EBF4 | FGVPTSATtEVSMTEIM | 1.8408 | TBI+CM | TBI 19 |
| ATP9B | VcHHIHHVPsVLLSAGPG | 1.8379 | TBI+CM | TBI 19 |
| ATP9B | VcHHIHHVPsVLLSAGPG | 1.8379 | TBI+CM | TBI 19 |
| ATP9B | VcHHIHHVPsVLLSAGPG | 1.8379 | TBI+CM | TBI 19 |
| EGF | cAFWNHGctLGCKNTP | 1.8214 | TBI+CM | TBI 19 |
| EGF | cAFWNHGctLGCKNTP | 1.8214 | TBI+CM | TBI 19 |
| EGF | cAFWNHGctLGCKNTP | 1.8214 | TBI+CM | TBI 19 |
| EGF | cAFWNHGctLGCKNTP | 1.8214 | TBI+CM | TBI 19 |
| EGF | cAFWNHGctLGCKNTP | 1.8214 | TBI+CM | TBI 19 |
| EGF | cAFWNHGctLGCKNTP | 1.8214 | TBI+CM | TBI 19 |
| EGF | cAFWNHGctLGCKNTP | 1.8214 | TBI+CM | TBI 19 |
| EGF | cAFWNHGctLGCKNTP | 1.8214 | TBI+CM | TBI 19 |
| EGF | cAFWNHGctLGCKNTP | 1.8214 | TBI+CM | TBI 19 |
| ERBB3 | QGNLGEScQEsAVSGSSE | 1.816 | TBI+CM | TBI 19 |
| ERBB3 | QGNLGEScQEsAVSGSSE | 1.816 | TBI+CM | TBI 19 |
| ERBB3 | QGNLGEScQEsAVSGSSE | 1.816 | TBI+CM | TBI 19 |
| ERBB3 | QGNLGEScQEsAVSGSSE | 1.816 | TBI+CM | TBI 19 |
| ERBB3 | QGNLGEScQEsAVSGSSE | 1.816 | TBI+CM | TBI 19 |
| ERBB3 | QGNLGEScQEsAVSGSSE | 1.816 | TBI+CM | TBI 19 |
| ERBB3 | QGNLGEScQEsAVSGSSE | 1.816 | TBI+CM | TBI 19 |
| ERBB3 | QGNLGEScQEsAVSGSSE | 1.816 | TBI+CM | TBI 19 |
| ERBB3 | QGNLGEScQEsAVSGSSE | 1.816 | TBI+CM | TBI 19 |
| ERBB3 | QGNLGEScQEsAVSGSSE | 1.816 | TBI+CM | TBI 19 |
| ERBB3 | QGNLGEScQEsAVSGSSE | 1.816 | TBI+CM | TBI 19 |
| ERBB3 | QGNLGEScQEsAVSGSSE | 1.816 | TBI+CM | TBI 19 |
| ERBB3 | QGNLGEScQEsAVSGSSE | 1.816 | TBI+CM | TBI 19 |
| ERBB3 | QGNLGEScQEsAVSGSSE | 1.816 | TBI+CM | TBI 19 |
| ERBB3 | QGNLGEScQEsAVSGSSE | 1.816 | TBI+CM | TBI 19 |
| SLC39A14 | SLCSLLGASVVPFMKKt | 1.7947 | TBI+CM | TBI 19 |
| SLC39A14 | SLCSLLGASVVPFMKKt | 1.7947 | TBI+CM | TBI 19 |
| SLC39A14 | SLCSLLGASVVPFMKKt | 1.7947 | TBI+CM | TBI 19 |
| SLC39A14 | SLCSLLGASVVPFMKKt | 1.7947 | TBI+CM | TBI 19 |
| SLC39A14 | SLCSLLGASVVPFMKKt | 1.7947 | TBI+CM | TBI 19 |
| SLC39A14 | SLCSLLGASVVPFMKKt | 1.7947 | TBI+CM | TBI 19 |
| SLC39A14 | SLCSLLGASVVPFMKKt | 1.7947 | TBI+CM | TBI 19 |
| SLC39A14 | SLCSLLGASVVPFMKKt | 1.7947 | TBI+CM | TBI 19 |
| SLC39A14 | SLCSLLGASVVPFMKKt | 1.7947 | TBI+CM | TBI 19 |
| ZNF91 | TCKRIHtGEKPcKCEE | 1.779 | TBI+CM | TBI 19 |
| ZNF91 | TCKRIHtGEKPcKCEE | 1.779 | TBI+CM | TBI 19 |
| ZNF91 | TCKRIHtGEKPcKCEE | 1.779 | TBI+CM | TBI 19 |
| ZNF91 | TCKRIHtGEKPCKcEE | 1.779 | TBI+CM | TBI 19 |
| ZNF91 | TCKRIHtGEKPcKCEE | 1.779 | TBI+CM | TBI 19 |
| ZNF91 | TCKRIHtGEKPCKcEE | 1.7344 | TBI+CM | TBI 19 |
| ZNF91 | TCKRIHtGEKPCKcEE | 1.7344 | TBI+CM | TBI 19 |
| ZNF91 | TCKRIHtGEKPCKcEE | 1.7344 | TBI+CM | TBI 19 |
| MYO15A | AKAcEQNLQKtLRF | 1.7158 | TBI+CM | TBI 19 |
| MYO15A | AKAcEQNLQKtLRF | 1.7158 | TBI+CM | TBI 19 |
| MYO15A | AKAcEQNLQKtLRF | 1.7158 | TBI+CM | TBI 19 |
| MYO15A | AKAcEQNLQKtLRF | 1.7158 | TBI+CM | TBI 19 |
| MYO15A | AKAcEQNLQKtLRF | 1.7158 | TBI+CM | TBI 19 |
| MYO15A | AKAcEQNLQKtLRF | 1.7158 | TBI+CM | TBI 19 |
| MYO15A | AKAcEQNLQKtLRF | 1.7158 | TBI+CM | TBI 19 |
| MYO15A | AKAcEQNLQKtLRF | 1.7158 | TBI+CM | TBI 19 |
| MYO15A | AKAcEQNLQKtLRF | 1.7158 | TBI+CM | TBI 19 |
| MYO15A | AKAcEQNLQKtLRF | 1.7158 | TBI+CM | TBI 19 |
| MYO15A | AKAcEQNLQKtLRF | 1.7158 | TBI+CM | TBI 19 |
| MYO15A | AKAcEQNLQKtLRF | 1.7158 | TBI+CM | TBI 19 |
| MYO15A | AKAcEQNLQKtLRF | 1.7158 | TBI+CM | TBI 19 |
| MYO15A | AKAcEQNLQKtLRF | 1.7158 | TBI+CM | TBI 19 |
| MYO15A | AKAcEQNLQKtLRF | 1.7158 | TBI+CM | TBI 19 |
| MYO15A | AKAcEQNLQKtLRF | 1.7158 | TBI+CM | TBI 19 |
| MYO15A | AKAcEQNLQKtLRF | 1.7158 | TBI+CM | TBI 19 |
| MYO15A | AKAcEQNLQKtLRF | 1.7158 | TBI+CM | TBI 19 |
| MYO15A | AKAcEQNLQKtLRF | 1.7158 | TBI+CM | TBI 19 |
| MYO15A | AKAcEQNLQKtLRF | 1.7158 | TBI+CM | TBI 19 |
| MYO15A | AKAcEQNLQKtLRF | 1.7158 | TBI+CM | TBI 19 |
| FBN2 | ctTTNmcLNGMCINEDG | 3.3564 | TBI | TBI 20 |
| FBN2 | ctTTNmcLNGMCINEDG | 3.3564 | TBI | TBI 20 |
| FBN2 | ctTTNmcLNGMCINEDG | 3.3564 | TBI | TBI 20 |
| FBN2 | ctTTNmcLNGMCINEDG | 3.3564 | TBI | TBI 20 |
| FBN2 | ctTTNmcLNGMCINEDG | 3.3564 | TBI | TBI 20 |
| FBN2 | ctTTNmcLNGMCINEDG | 3.3564 | TBI | TBI 20 |
| FBN2 | ctTTNmcLNGMCINEDG | 3.3564 | TBI | TBI 20 |
| FBN2 | ctTTNmcLNGMCINEDG | 3.3564 | TBI | TBI 20 |
| FBN2 | ctTTNmcLNGMCINEDG | 3.3564 | TBI | TBI 20 |
| FBN2 | ctTTNmcLNGMCINEDG | 3.3564 | TBI | TBI 20 |
| FBN2 | ctTTNmcLNGMCINEDG | 3.3564 | TBI | TBI 20 |
| FBN2 | ctTTNmcLNGMCINEDG | 3.3564 | TBI | TBI 20 |
| FBN2 | ctTTNmcLNGMCINEDG | 3.3564 | TBI | TBI 20 |
| FBN2 | ctTTNmcLNGMCINEDG | 3.3564 | TBI | TBI 20 |
| FBN2 | ctTTNmcLNGMCINEDG | 3.3564 | TBI | TBI 20 |
| FBN2 | cDMHASCLNIPGSFKCSC | 3.3564 | TBI | TBI 20 |
| FBN2 | cTtTNmcLNGMCINEDG | 3.3564 | TBI | TBI 20 |
| FBN2 | ctTTNmcLNGMCINEDG | 3.3564 | TBI | TBI 20 |
| FREM2 | DPPLYGEILVNG | 3.1027 | TBI | TBI 20 |
| FREM2 | tLGmNFTQDEVDRNL | 3.1027 | TBI | TBI 20 |
| FREM2 | DPPLYGEILVNG | 3.1027 | TBI | TBI 20 |
| FREM2 | tLGmNFTQDEVDRNL | 3.1027 | TBI | TBI 20 |
| FREM2 | DPPLYGEILVNG | 3.1027 | TBI | TBI 20 |
| FREM2 | tLGmNFTQDEVDRNL | 3.1027 | TBI | TBI 20 |
| FREM2 | DPPLYGEILVNG | 3.1027 | TBI | TBI 20 |
| FREM2 | tLGmNFTQDEVDRNL | 3.1027 | TBI | TBI 20 |
| CACNA2D1 | RDPcAGPVcDCKRN | 2.9126 | TBI | TBI 20 |
| CACNA2D1 | RDPcAGPVcDCKRN | 2.9126 | TBI | TBI 20 |
| CACNA2D1 | RDPcAGPVcDCKRN | 2.9126 | TBI | TBI 20 |
| CACNA2D1 | RDPcAGPVcDCKRN | 2.9126 | TBI | TBI 20 |
| CACNA2D1 | RDPcAGPVcDCKRN | 2.9126 | TBI | TBI 20 |
| CACNA2D1 | TDcGGVsGLNPSLWYIIG | 2.9126 | TBI | TBI 20 |
| VLDLR | DEVNcPSRTc | 2.442 | TBI | TBI 20 |
| VLDLR | DISKVcNQEQDCR | 2.442 | TBI | TBI 20 |
| VLDLR | DEVNcPSRTc | 2.442 | TBI | TBI 20 |
| VLDLR | DEVNcPSRTc | 2.442 | TBI | TBI 20 |
| VLDLR | DEVNcPSRTc | 2.442 | TBI | TBI 20 |
| VLDLR | DEVNcPSRTc | 2.442 | TBI | TBI 20 |
| MUC6 | LCPSQPQSVPGSNIEGCY | 2.2642 | TBI | TBI 20 |
| MUC6 | LCPSQPQSVPGSNIEGCY | 2.2642 | TBI | TBI 20 |
| MUC6 | LCPSQPQSVPGSNIEGCY | 2.2642 | TBI | TBI 20 |
| CCDC18 | ADLKRQKVIELtGTAR | 2.2437 | TBI | TBI 20 |
| CCDC18 | ADLKRQKVIELtGTAR | 2.2437 | TBI | TBI 20 |
| CCDC18 | ADLKRQKVIELTGtAR | 2.2437 | TBI | TBI 20 |
| CCDC18 | ADLKRQKVIELtGTAR | 2.2437 | TBI | TBI 20 |
| CCDC18 | ADLKRQKVIELTGtAR | 2.2437 | TBI | TBI 20 |
| CCDC18 | ADLKRQKVIELTGtAR | 2.2437 | TBI | TBI 20 |
| CCDC18 | ADLKRQKVIELTGtAR | 2.2437 | TBI | TBI 20 |
| CCDC18 | ADLKRQKVIELtGTAR | 2.2437 | TBI | TBI 20 |
| CCDC18 | ADLKRQKVIELTGtAR | 2.2437 | TBI | TBI 20 |
| HUWE1 | PTTATTTVSISPtTKGSKS | 2.243 | TBI | TBI 20 |
| HUWE1 | PTTATTTVSISPTtKGSKS | 2.243 | TBI | TBI 20 |
| HUWE1 | PTTATTTVSISPTtKGSKS | 2.243 | TBI | TBI 20 |
| HUWE1 | PTTATTTVSISPTtKGSKS | 2.243 | TBI | TBI 20 |
| HUWE1 | PTTATTTVSISPtTKGSKS | 2.243 | TBI | TBI 20 |
| HUWE1 | PTTATTTVSISPtTKGSKS | 2.243 | TBI | TBI 20 |
| HUWE1 | PTTATTTVSISPtTKGSKS | 2.243 | TBI | TBI 20 |
| HUWE1 | PTTATTTVSISPTtKGSKS | 2.243 | TBI | TBI 20 |
| HUWE1 | PTTATTTVSISPtTKGSKS | 2.243 | TBI | TBI 20 |
| TBX22 | RSSAAGKSEPLEKQPKTE | 2.1815 | TBI | TBI 20 |
| TBX22 | RSSAAGKSEPLEKQPKTE | 2.1815 | TBI | TBI 20 |
| TBX22 | RSSAAGKSEPLEKQPKTE | 2.1815 | TBI | TBI 20 |
| MUC4 | PVTSPSSASTGHAtPLPVTG | 2.1461 | TBI | TBI 20 |
| MUC4 | PVTSPSSASTGHAtPLPVTG | 2.1461 | TBI | TBI 20 |
| MUC4 | PVTSPSSASTGHAtPLPVTG | 2.1461 | TBI | TBI 20 |
| PADI3 | SSyDAKRAQVFHIc | 2.1434 | TBI | TBI 20 |
| PADI3 | SSyDAKRAQVFHIc | 2.1434 | TBI | TBI 20 |
| PADI3 | SsYDAKRAQVFHIc | 2.1434 | TBI | TBI 20 |
| PADI3 | SsYDAKRAQVFHIc | 2.1434 | TBI | TBI 20 |
| PADI3 | SsYDAKRAQVFHIc | 2.1434 | TBI | TBI 20 |
| PADI3 | SSyDAKRAQVFHIc | 2.1434 | TBI | TBI 20 |
| PADI3 | SsYDAKRAQVFHIc | 2.1434 | TBI | TBI 20 |
| PADI3 | SsYDAKRAQVFHIc | 2.1434 | TBI | TBI 20 |
| STAT1 | LETTSLPVVVIsNVSQL | 2.134 | TBI | TBI 20 |
| STAT1 | LETTSLPVVVIsNVSQL | 2.134 | TBI | TBI 20 |
| STAT1 | LETTSLPVVVIsNVSQL | 2.134 | TBI | TBI 20 |
| STAT1 | LETTSLPVVVIsNVSQL | 2.134 | TBI | TBI 20 |
| STAT1 | LETTSLPVVVIsNVSQL | 2.134 | TBI | TBI 20 |
| STAT1 | LETTSLPVVVIsNVSQL | 2.134 | TBI | TBI 20 |
| STAT1 | LETTSLPVVVIsNVSQL | 2.134 | TBI | TBI 20 |
| RUNX2 | LQPGKmSDVSPQQQQQQ | 2.1286 | TBI | TBI 20 |
| RUNX2 | LQPGKmSDVSPQQQQQQ | 2.1286 | TBI | TBI 20 |
| RUNX2 | LQPGKmSDVSPQQQQQQ | 2.1286 | TBI | TBI 20 |
| SLC25A40 | QMLAsCTGAILTSVIVTP | 2.1166 | TBI | TBI 20 |
| SLC25A40 | QMLAsCTGAILTSVIVTP | 2.1166 | TBI | TBI 20 |
| SLC25A40 | QMLAsCTGAILTSVIVTP | 2.1166 | TBI | TBI 20 |
| SLC25A40 | QMLAsCTGAILTSVIVTP | 2.1166 | TBI | TBI 20 |
| SLC25A40 | QMLAsCTGAILTSVIVTP | 2.1166 | TBI | TBI 20 |
| SLC25A40 | QMLAsCTGAILTSVIVTP | 2.1166 | TBI | TBI 20 |
| DKC1 | sESAKKEVVAEVVKAPQ | 2.1141 | TBI | TBI 20 |
| DKC1 | sESAKKEVVAEVVKAPQ | 2.1141 | TBI | TBI 20 |
| DKC1 | SEsAKKEVVAEVVKAPQ | 2.1141 | TBI | TBI 20 |
| DKC1 | sESAKKEVVAEVVKAPQ | 2.1141 | TBI | TBI 20 |
| DKC1 | sESAKKEVVAEVVKAPQ | 2.1141 | TBI | TBI 20 |
| DKC1 | sESAKKEVVAEVVKAPQ | 2.1141 | TBI | TBI 20 |
| DKC1 | sESAKKEVVAEVVKAPQ | 2.1141 | TBI | TBI 20 |
| DKC1 | sESAKKEVVAEVVKAPQ | 2.1141 | TBI | TBI 20 |
| DKC1 | SEsAKKEVVAEVVKAPQ | 2.1141 | TBI | TBI 20 |
| DKC1 | SEsAKKEVVAEVVKAPQ | 2.1141 | TBI | TBI 20 |
| DKC1 | SEsAKKEVVAEVVKAPQ | 2.1141 | TBI | TBI 20 |
| DKC1 | sESAKKEVVAEVVKAPQ | 2.1141 | TBI | TBI 20 |
| DKC1 | SEsAKKEVVAEVVKAPQ | 2.1141 | TBI | TBI 20 |
| REEP6 | QRNLVTEVLGALEAKtG | 2.1121 | TBI | TBI 20 |
| REEP6 | QRNLVTEVLGALEAKtG | 2.1121 | TBI | TBI 20 |
| REEP6 | QRNLVTEVLGALEAKtG | 2.1121 | TBI | TBI 20 |
| REEP6 | QRNLVTEVLGALEAKtG | 2.1121 | TBI | TBI 20 |
| REEP6 | QRNLVTEVLGALEAKtG | 2.1121 | TBI | TBI 20 |
| REEP6 | QRNLVTEVLGALEAKtG | 2.1121 | TBI | TBI 20 |
| DKC1 | sESAKKEVVAEVVKAPQ | 2.0919 | TBI | TBI 20 |
| DKC1 | sESAKKEVVAEVVKAPQ | 2.0919 | TBI | TBI 20 |
| DKC1 | sESAKKEVVAEVVKAPQ | 2.0919 | TBI | TBI 20 |
| GLRB | EDIEYGNcTKyYKGT | 2.0804 | TBI | TBI 20 |
| GLRB | EDIEYGNcTKyYKGT | 2.0804 | TBI | TBI 20 |
| GLRB | EDIEYGNcTKyYKGT | 2.0804 | TBI | TBI 20 |
| GLRB | EDIEYGNcTKyYKGT | 2.0804 | TBI | TBI 20 |
| HUWE1 | PTTATTTVSISPTtKGSKS | 2.0698 | TBI | TBI 20 |
| HUWE1 | PTTATTTVSISPTtKGSKS | 2.0698 | TBI | TBI 20 |
| HUWE1 | PTTATTTVSISPTtKGSKS | 2.0698 | TBI | TBI 20 |
| CCDC18 | ADLKRQKVIELtGTAR | 2.0571 | TBI | TBI 20 |
| CCDC18 | ADLKRQKVIELtGTAR | 2.0571 | TBI | TBI 20 |
| CCDC18 | ADLKRQKVIELtGTAR | 2.0571 | TBI | TBI 20 |
| NFASC | EQSIWNVtVLPNSKW | 2.0535 | TBI | TBI 20 |
| NFASC | EQSIWNVtVLPNSKW | 2.0535 | TBI | TBI 20 |
| NFASC | EQSIWNVtVLPNSKW | 2.0535 | TBI | TBI 20 |
| NFASC | EQSIWNVtVLPNSKW | 2.0535 | TBI | TBI 20 |
| MUC19 | TGLPGESAEVtGTIGSPAGV | 2.0475 | TBI | TBI 20 |
| MUC19 | TGLPGESAEVtGTIGSPAGV | 2.0475 | TBI | TBI 20 |
| MUC19 | TGLPGESAEVtGTIGSPAGV | 2.0475 | TBI | TBI 20 |
| MUC19 | TGLPGESAEVtGTIGSPAGV | 2.0475 | TBI | TBI 20 |
| FBXO34 | PFAcGIEHCsVHYVSDS | 2.0397 | TBI | TBI 20 |
| FBXO34 | PFAcGIEHCsVHYVSDS | 2.0397 | TBI | TBI 20 |
| FBXO34 | PFAcGIEHCsVHYVSDS | 2.0397 | TBI | TBI 20 |
| FBXO34 | PFAcGIEHCsVHYVSDS | 2.0397 | TBI | TBI 20 |
| MCRS1 | SLcPcccLAGETLVSQAPT | 2.0225 | TBI | TBI 20 |
| MCRS1 | SLcPcccLAGETLVSQAPT | 2.0225 | TBI | TBI 20 |
| MCRS1 | SLcPcccLAGETLVSQAPT | 2.0225 | TBI | TBI 20 |
| MCRS1 | SLcPcccLAGETLVSQAPT | 2.0225 | TBI | TBI 20 |
| CA3 | TKGKEAPFTKFDPsCL | 2.0204 | TBI | TBI 20 |
| CA3 | TKGKEAPFTKFDPsCL | 2.0204 | TBI | TBI 20 |
| CA3 | TKGKEAPFTKFDPsCL | 2.0204 | TBI | TBI 20 |
| CA3 | TKGKEAPFTKFDPsCL | 2.0204 | TBI | TBI 20 |
| CA3 | TKGKEAPFTKFDPsCL | 2.0204 | TBI | TBI 20 |
| MCRS1 | SLcPcccLAGETLVSQAPT | 2.0045 | TBI | TBI 20 |
| SLC12A1 | EmNsGmAKKQAWLIK | 1.9917 | TBI | TBI 20 |
| SLC12A1 | EmNsGmAKKQAWLIK | 1.9917 | TBI | TBI 20 |
| SLC12A1 | EmNsGmAKKQAWLIK | 1.9917 | TBI | TBI 20 |
| SLC12A1 | EmNsGmAKKQAWLIK | 1.9917 | TBI | TBI 20 |
| SLC12A1 | EmNsGmAKKQAWLIK | 1.9917 | TBI | TBI 20 |
| SLC12A1 | EmNsGmAKKQAWLIK | 1.9917 | TBI | TBI 20 |
| SLC12A1 | EmNsGmAKKQAWLIK | 1.9917 | TBI | TBI 20 |
| SLC12A1 | EmNsGmAKKQAWLIK | 1.9917 | TBI | TBI 20 |
| PKD1 | mLILQAETTAGTVTPTAI | 1.9855 | TBI | TBI 20 |
| PKD1 | mLILQAETTAGTVTPTAI | 1.9855 | TBI | TBI 20 |
| PKD1 | mLILQAETTAGTVTPTAI | 1.9855 | TBI | TBI 20 |
| PKD1 | mLILQAETTAGTVTPTAI | 1.9855 | TBI | TBI 20 |
| MAPRE2 | sGSTQcAGSVPTGAAYcQF | 1.9851 | TBI | TBI 20 |
| MAPRE2 | SGStQcAGSVPTGAAYcQF | 1.9851 | TBI | TBI 20 |
| MAPRE2 | SGsTQcAGSVPTGAAYcQF | 1.9851 | TBI | TBI 20 |
| MAPRE2 | sGSTQcAGSVPTGAAYcQF | 1.9851 | TBI | TBI 20 |
| MAPRE2 | sGSTQcAGSVPTGAAYcQF | 1.9851 | TBI | TBI 20 |
| MAPRE2 | sGSTQcAGSVPTGAAYcQF | 1.9851 | TBI | TBI 20 |
| MAPRE2 | SGsTQcAGSVPTGAAYcQF | 1.9851 | TBI | TBI 20 |
| MAPRE2 | sGSTQcAGSVPTGAAYcQF | 1.9851 | TBI | TBI 20 |
| MAPRE2 | sGSTQcAGSVPTGAAYcQF | 1.9851 | TBI | TBI 20 |
| MAPRE2 | SGsTQcAGSVPTGAAYcQF | 1.9851 | TBI | TBI 20 |
| MAPRE2 | sGSTQcAGSVPTGAAYcQF | 1.9851 | TBI | TBI 20 |
| SLAMF7 | YGLLHcGNTEKDGKSPL | 1.9619 | TBI | TBI 20 |
| SLAMF7 | YGLLHcGNTEKDGKSPL | 1.9619 | TBI | TBI 20 |
| SLAMF7 | YGLLHcGNTEKDGKSPL | 1.9619 | TBI | TBI 20 |
| SLAMF7 | YGLLHcGNTEKDGKSPL | 1.9619 | TBI | TBI 20 |
| SLAMF7 | YGLLHcGNTEKDGKSPL | 1.9619 | TBI | TBI 20 |
| EPS8 | EGARVySQITVQKAALE | 1.9618 | TBI | TBI 20 |
| EPS8 | EGARVySQITVQKAALE | 1.9618 | TBI | TBI 20 |
| EPS8 | EGARVySQITVQKAALE | 1.9618 | TBI | TBI 20 |
| EPS8 | EGARVySQITVQKAALE | 1.9618 | TBI | TBI 20 |
| EPS8 | EGARVySQITVQKAALE | 1.9618 | TBI | TBI 20 |
| MUC17 | EGSTPLTRMPVSTtMVAS | 1.9555 | TBI | TBI 20 |
| MUC17 | EGSTPLTRMPVSTtMVAS | 1.9555 | TBI | TBI 20 |
| MUC17 | EGSTPLTRMPVSTtMVAS | 1.9555 | TBI | TBI 20 |
| NFKB2 | cSTKVKTLLLNAAQNtM | 1.9533 | TBI | TBI 20 |
| NFKB2 | cSTKVKTLLLNAAQNtM | 1.9533 | TBI | TBI 20 |
| NFKB2 | cSTKVKTLLLNAAQNtM | 1.9533 | TBI | TBI 20 |
| NSD3 | LPmIPsSSASKKKcEKGG | 1.9465 | TBI | TBI 20 |
| NSD3 | LPmIPsSSASKKKcEKGG | 1.9465 | TBI | TBI 20 |
| NSD3 | LPmIPsSSASKKKcEKGG | 1.9465 | TBI | TBI 20 |
| NSD3 | LPmIPsSSASKKKcEKGG | 1.9465 | TBI | TBI 20 |
| SLC23A1 | PcQWGLPtVTAAAVLGMF | 1.9359 | TBI | TBI 20 |
| SLC23A1 | PcQWGLPtVTAAAVLGMF | 1.9359 | TBI | TBI 20 |
| SLC23A1 | PcQWGLPtVTAAAVLGMF | 1.9359 | TBI | TBI 20 |
| SLC23A1 | PcQWGLPtVTAAAVLGMF | 1.9359 | TBI | TBI 20 |
| SLC23A1 | PcQWGLPtVTAAAVLGMF | 1.9359 | TBI | TBI 20 |
| MAN1B1 | RTPSKIPYSDVNIGtGV | 1.9252 | TBI | TBI 20 |
| MAN1B1 | RTPSKIPYsDVNIGTGV | 1.9252 | TBI | TBI 20 |
| MAN1B1 | RTPSKIPYSDVNIGtGV | 1.9252 | TBI | TBI 20 |
| MAN1B1 | RTPSKIPYsDVNIGTGV | 1.9252 | TBI | TBI 20 |
| MAN1B1 | RTPSKIPYSDVNIGtGV | 1.9252 | TBI | TBI 20 |
| MAN1B1 | RTPSKIPYsDVNIGTGV | 1.9252 | TBI | TBI 20 |
| MAN1B1 | RTPSKIPYSDVNIGtGV | 1.9252 | TBI | TBI 20 |
| MAN1B1 | RTPSKIPYsDVNIGTGV | 1.9252 | TBI | TBI 20 |
| MAN1B1 | RTPSKIPYSDVNIGtGV | 1.9252 | TBI | TBI 20 |
| MAN1B1 | RTPSKIPYsDVNIGTGV | 1.9252 | TBI | TBI 20 |
| MAN1B1 | RTPSKIPYSDVNIGtGV | 1.9252 | TBI | TBI 20 |
| MAN1B1 | RTPSKIPYsDVNIGTGV | 1.9252 | TBI | TBI 20 |
| MAN1B1 | RTPSKIPYSDVNIGtGV | 1.9252 | TBI | TBI 20 |
| MAN1B1 | RTPSKIPYsDVNIGTGV | 1.9252 | TBI | TBI 20 |
| MAN1B1 | RTPSKIPYSDVNIGtGV | 1.9252 | TBI | TBI 20 |
| MAN1B1 | RTPSKIPYSDVNIGtGV | 1.9252 | TBI | TBI 20 |
| MAN1B1 | RTPSKIPYsDVNIGTGV | 1.9252 | TBI | TBI 20 |
| ABCG5 | VESGQIMcILGSsGSGKT | 1.9203 | TBI | TBI 20 |
| ABCG5 | VESGQIMcILGSSGsGKT | 1.9203 | TBI | TBI 20 |
| ABCG5 | VESGQIMcILGSsGSGKT | 1.9203 | TBI | TBI 20 |
| ABCG5 | VESGQIMcILGSsGSGKT | 1.9203 | TBI | TBI 20 |
| ABCG5 | VESGQIMcILGSSGsGKT | 1.9203 | TBI | TBI 20 |
| AFF3 | QPNcRTSVPsSKGSSSS | 1.9111 | TBI | TBI 20 |
| AFF3 | QPNcRTSVPSSKGSsSS | 1.9111 | TBI | TBI 20 |
| AFF3 | QPNcRTSVPSSKGSsSS | 1.9111 | TBI | TBI 20 |
| AFF3 | QPNcRTSVPSSKGSsSS | 1.9111 | TBI | TBI 20 |
| AFF3 | QPNcRTSVPsSKGSSSS | 1.9111 | TBI | TBI 20 |
| AFF3 | QPNcRTSVPSSKGSsSS | 1.9111 | TBI | TBI 20 |
| AFF3 | QPNcRTSVPSSKGSsSS | 1.9111 | TBI | TBI 20 |
| AFF3 | QPNcRTSVPSSKGSsSS | 1.9111 | TBI | TBI 20 |
| AFF3 | QPNcRTSVPsSKGSSSS | 1.9111 | TBI | TBI 20 |
| AFF3 | QPNcRTSVPSSKGsSSS | 1.9111 | TBI | TBI 20 |
| AFF3 | QPNcRTSVPSsKGSSSS | 1.9111 | TBI | TBI 20 |
| AFF3 | QPNcRTSVPSSKGSsSS | 1.9111 | TBI | TBI 20 |
| AFF3 | QPNcRTSVPsSKGSSSS | 1.9111 | TBI | TBI 20 |
| AFF3 | QPNcRTSVPSSKGSsSS | 1.9111 | TBI | TBI 20 |
| AFF3 | QPNcRTSVPSSKGSsSS | 1.9111 | TBI | TBI 20 |
| AFF3 | QPNcRTSVPSSKGSsSS | 1.9111 | TBI | TBI 20 |
| AFF3 | QPNcRTSVPsSKGSSSS | 1.9111 | TBI | TBI 20 |
| AFF3 | QPNcRTSVPSSKGSsSS | 1.9111 | TBI | TBI 20 |
| AFF3 | QPNcRTSVPSSKGSsSS | 1.9111 | TBI | TBI 20 |
| AFF3 | QPNcRTSVPSSKGSsSS | 1.9111 | TBI | TBI 20 |
| AFF3 | QPNcRTSVPsSKGSSSS | 1.9111 | TBI | TBI 20 |
| AFF3 | QPNcRTSVPsSKGSSSS | 1.9111 | TBI | TBI 20 |
| AFF3 | QPNcRTSVPsSKGSSSS | 1.9111 | TBI | TBI 20 |
| AFF3 | QPNcRTSVPsSKGSSSS | 1.9111 | TBI | TBI 20 |
| AFF3 | QPNcRTSVPSSKGSsSS | 1.9111 | TBI | TBI 20 |
| AFF3 | QPNcRTSVPsSKGSSSS | 1.9111 | TBI | TBI 20 |
| AFF3 | QPNcRTSVPsSKGSSSS | 1.9111 | TBI | TBI 20 |
| AFF3 | QPNcRTSVPsSKGSSSS | 1.9111 | TBI | TBI 20 |
| AFF3 | QPNcRTSVPsSKGSSSS | 1.9111 | TBI | TBI 20 |
| AFF3 | QPNcRTSVPsSKGSSSS | 1.9111 | TBI | TBI 20 |
| AFF3 | QPNcRTSVPsSKGSSSS | 1.9111 | TBI | TBI 20 |
| AFF3 | QPNcRTSVPSSKGSsSS | 1.9111 | TBI | TBI 20 |
| AFF3 | QPNcRTSVPsSKGSSSS | 1.9111 | TBI | TBI 20 |
| AFF3 | QPNcRTSVPsSKGSSSS | 1.9111 | TBI | TBI 20 |
| AFF3 | QPNcRTSVPsSKGSSSS | 1.9111 | TBI | TBI 20 |
| AFF3 | QPNcRTSVPsSKGSSSS | 1.9111 | TBI | TBI 20 |
| AFF3 | QPNcRTSVPsSKGSSSS | 1.9111 | TBI | TBI 20 |
| AFF3 | QPNcRTSVPsSKGSSSS | 1.9111 | TBI | TBI 20 |
| AFF3 | QPNcRTSVPSSKGSsSS | 1.9111 | TBI | TBI 20 |
| AFF3 | QPNcRTSVPsSKGSSSS | 1.9111 | TBI | TBI 20 |
| AFF3 | QPNcRTSVPsSKGSSSS | 1.9111 | TBI | TBI 20 |
| AFF3 | QPNcRTSVPsSKGSSSS | 1.9111 | TBI | TBI 20 |
| AFF3 | QPNcRTSVPsSKGSSSS | 1.9111 | TBI | TBI 20 |
| MAN1B1 | RTPSKIPYsDVNIGTGV | 1.9101 | TBI | TBI 20 |
| TECPR1 | KVQGRPSPQAIWSITCKG | 1.9087 | TBI | TBI 20 |
| TECPR1 | KVQGRPSPQAIWSITCKG | 1.9087 | TBI | TBI 20 |
| TECPR1 | KVQGRPSPQAIWSITCKG | 1.9087 | TBI | TBI 20 |
| TECPR1 | KVQGRPSPQAIWSITCKG | 1.9087 | TBI | TBI 20 |
| TECPR1 | KVQGRPSPQAIWSITCKG | 1.9087 | TBI | TBI 20 |
| UBR7 | ELENEAcAVLGGSDsEK | 1.9043 | TBI | TBI 20 |
| UBR7 | ELENEAcAVLGGSDsEK | 1.9043 | TBI | TBI 20 |
| UBR7 | ELENEAcAVLGGSDsEK | 1.9043 | TBI | TBI 20 |
| UBR7 | ELENEAcAVLGGSDsEK | 1.9043 | TBI | TBI 20 |
| UBR7 | ELENEAcAVLGGSDsEK | 1.9043 | TBI | TBI 20 |
| MIPOL1 | YKtKEcKMRITAEEM | 1.9024 | TBI | TBI 20 |
| MIPOL1 | YKtKEcKMRITAEEM | 1.9024 | TBI | TBI 20 |
| MIPOL1 | YKtKEcKMRITAEEM | 1.9024 | TBI | TBI 20 |
| MIPOL1 | YKtKEcKMRITAEEM | 1.9024 | TBI | TBI 20 |
| MIPOL1 | YKtKEcKMRITAEEM | 1.9024 | TBI | TBI 20 |
| MIPOL1 | YKtKEcKMRITAEEM | 1.9024 | TBI | TBI 20 |
| PCDH8 | AIIAIATTCNRRKKEVR | 1.9005 | TBI | TBI 20 |
| PCDH8 | AIIAIATTCNRRKKEVR | 1.9005 | TBI | TBI 20 |
| PCDH8 | AIIAIATTCNRRKKEVR | 1.9005 | TBI | TBI 20 |
| PCDH8 | AIIAIATTCNRRKKEVR | 1.9005 | TBI | TBI 20 |
| PCDH8 | AIIAIATTCNRRKKEVR | 1.9005 | TBI | TBI 20 |
| AFF3 | QPNcRTSVPSSKGsSSS | 1.8915 | TBI | TBI 20 |
| AFF3 | QPNcRTSVPSSKGsSSS | 1.8915 | TBI | TBI 20 |
| AFF3 | QPNcRTSVPSSKGsSSS | 1.8915 | TBI | TBI 20 |
| AFF3 | QPNcRTSVPSSKGsSSS | 1.8915 | TBI | TBI 20 |
| AFF3 | QPNcRTSVPSSKGsSSS | 1.8915 | TBI | TBI 20 |
| AFF3 | QPNcRTSVPSSKGsSSS | 1.8915 | TBI | TBI 20 |
| AFF3 | QPNcRTSVPSSKGsSSS | 1.8915 | TBI | TBI 20 |
| AFF3 | QPNcRTSVPSSKGsSSS | 1.8915 | TBI | TBI 20 |
| AFF3 | QPNcRTSVPSSKGsSSS | 1.8915 | TBI | TBI 20 |
| AFF3 | QPNcRTSVPSSKGsSSS | 1.8915 | TBI | TBI 20 |
| AFF3 | QPNcRTSVPSSKGsSSS | 1.8915 | TBI | TBI 20 |
| AFF3 | QPNcRTSVPSSKGsSSS | 1.8915 | TBI | TBI 20 |
| AFF3 | QPNcRTSVPSSKGsSSS | 1.8915 | TBI | TBI 20 |
| AFF3 | QPNcRTSVPSSKGsSSS | 1.8915 | TBI | TBI 20 |
| AFF3 | QPNcRTSVPSSKGsSSS | 1.8915 | TBI | TBI 20 |
| AFF3 | QPNcRTSVPSSKGsSSS | 1.8915 | TBI | TBI 20 |
| AFF3 | QPNcRTSVPSSKGsSSS | 1.8915 | TBI | TBI 20 |
| AFF3 | QPNcRTSVPSSKGsSSS | 1.8915 | TBI | TBI 20 |
| AFF3 | QPNcRTSVPSSKGsSSS | 1.8915 | TBI | TBI 20 |
| AFF3 | QPNcRTSVPSsKGSSSS | 1.89 | TBI | TBI 20 |
| AFF3 | QPNcRTSVPSsKGSSSS | 1.89 | TBI | TBI 20 |
| AFF3 | QPNcRTSVPSsKGSSSS | 1.89 | TBI | TBI 20 |
| AFF3 | QPNcRTSVPSsKGSSSS | 1.89 | TBI | TBI 20 |
| AFF3 | QPNcRTSVPSsKGSSSS | 1.89 | TBI | TBI 20 |
| AFF3 | QPNcRTSVPSsKGSSSS | 1.89 | TBI | TBI 20 |
| AFF3 | QPNcRTSVPSsKGSSSS | 1.89 | TBI | TBI 20 |
| AFF3 | QPNcRTSVPSsKGSSSS | 1.89 | TBI | TBI 20 |
| AFF3 | QPNcRTSVPSsKGSSSS | 1.89 | TBI | TBI 20 |
| AFF3 | QPNcRTSVPSsKGSSSS | 1.89 | TBI | TBI 20 |
| AFF3 | QPNcRTSVPSsKGSSSS | 1.89 | TBI | TBI 20 |
| AFF3 | QPNcRTSVPSsKGSSSS | 1.89 | TBI | TBI 20 |
| AFF3 | QPNcRTSVPSsKGSSSS | 1.89 | TBI | TBI 20 |
| AFF3 | QPNcRTSVPSsKGSSSS | 1.89 | TBI | TBI 20 |
| AFF3 | QPNcRTSVPSsKGSSSS | 1.89 | TBI | TBI 20 |
| AFF3 | QPNcRTSVPSsKGSSSS | 1.89 | TBI | TBI 20 |
| AFF3 | QPNcRTSVPSsKGSSSS | 1.89 | TBI | TBI 20 |
| AFF3 | QPNcRTSVPSsKGSSSS | 1.89 | TBI | TBI 20 |
| AFF3 | QPNcRTSVPSsKGSSSS | 1.89 | TBI | TBI 20 |
| LOC646383 | SGTECGcILPFLcFLsR | 1.8882 | TBI | TBI 20 |
| LOC646383 | SGTECGcILPFLcFLsR | 1.8882 | TBI | TBI 20 |
| LOC646383 | SGTECGcILPFLcFLsR | 1.8882 | TBI | TBI 20 |
| DNAH14 | PtKCHYMFNLRDMF | 1.8804 | TBI | TBI 20 |
| DNAH14 | PtKCHYMFNLRDMF | 1.8804 | TBI | TBI 20 |
| DNAH14 | PtKCHYMFNLRDMF | 1.8804 | TBI | TBI 20 |
| DNAH14 | PtKCHYMFNLRDMF | 1.8804 | TBI | TBI 20 |
| DNAH14 | PtKCHYMFNLRDMF | 1.8804 | TBI | TBI 20 |
| ING2 | ScVSLTyKPKGKWYCP | 1.8742 | TBI | TBI 20 |
| ING2 | ScVSLTyKPKGKWYCP | 1.8742 | TBI | TBI 20 |
| ING2 | ScVsLTYKPKGKWYCP | 1.8742 | TBI | TBI 20 |
| ING2 | ScVSLTyKPKGKWYCP | 1.8742 | TBI | TBI 20 |
| ING2 | ScVsLTYKPKGKWYCP | 1.8742 | TBI | TBI 20 |
| ING2 | ScVsLTYKPKGKWYCP | 1.8742 | TBI | TBI 20 |
| ING2 | ScVsLTYKPKGKWYCP | 1.8742 | TBI | TBI 20 |
| ING2 | ScVSLTyKPKGKWYCP | 1.8742 | TBI | TBI 20 |
| ING2 | ScVsLTYKPKGKWYCP | 1.8742 | TBI | TBI 20 |
| ING2 | ScVsLTYKPKGKWYCP | 1.8742 | TBI | TBI 20 |
| ING2 | ScVsLTYKPKGKWYCP | 1.8742 | TBI | TBI 20 |
| ING2 | ScVSLTyKPKGKWYCP | 1.8742 | TBI | TBI 20 |
| ING2 | ScVsLTYKPKGKWYCP | 1.8742 | TBI | TBI 20 |
| PADI3 | SSyDAKRAQVFHIc | 1.8706 | TBI | TBI 20 |
| PADI3 | SSyDAKRAQVFHIc | 1.8706 | TBI | TBI 20 |
| PADI3 | SSyDAKRAQVFHIc | 1.8706 | TBI | TBI 20 |
| PADI3 | SSyDAKRAQVFHIc | 1.8706 | TBI | TBI 20 |
| ABCG5 | VESGQIMcILGSSGsGKT | 1.867 | TBI | TBI 20 |
| TRB | SATYLcAVQDLGTSGSRLT | 1.8589 | TBI | TBI 20 |
| TRB | SATYLcAVQDLGTSGSRLT | 1.8589 | TBI | TBI 20 |
| TRB | SATYLcAVQDLGTSGSRLT | 1.8589 | TBI | TBI 20 |
| TRB | SATYLcAVQDLGTSGSRLT | 1.8589 | TBI | TBI 20 |
| CCER1 | RYSPKtEYGPPRKQP | 1.8379 | TBI | TBI 20 |
| CCER1 | RYSPKtEYGPPRKQP | 1.8379 | TBI | TBI 20 |
| CCER1 | RYSPKtEYGPPRKQP | 1.8379 | TBI | TBI 20 |
| MT-ND5 | CLLVWFCFLCcVsLC | 1.8353 | TBI | TBI 20 |
| MT-ND5 | CLLVWFCFLCcVsLC | 1.8353 | TBI | TBI 20 |
| SBF1 | PKLLRPRLLPGEECVLD | 1.8308 | TBI | TBI 20 |
| SBF1 | PKLLRPRLLPGEECVLD | 1.8308 | TBI | TBI 20 |
| SBF1 | PKLLRPRLLPGEECVLD | 1.8308 | TBI | TBI 20 |
| SBF1 | PKLLRPRLLPGEECVLD | 1.8308 | TBI | TBI 20 |
| SBF1 | PKLLRPRLLPGEECVLD | 1.8308 | TBI | TBI 20 |
| SBF1 | PKLLRPRLLPGEECVLD | 1.8308 | TBI | TBI 20 |
| SBF1 | PKLLRPRLLPGEECVLD | 1.8308 | TBI | TBI 20 |
| SBF1 | PKLLRPRLLPGEECVLD | 1.8308 | TBI | TBI 20 |
| SBF1 | PKLLRPRLLPGEECVLD | 1.8308 | TBI | TBI 20 |
| AFF3 | QPNcRTSVPSSKGSsSS | 1.8282 | TBI | TBI 20 |
| AFF3 | QPNcRTSVPSSKGSsSS | 1.8282 | TBI | TBI 20 |
| AFF3 | QPNcRTSVPSSKGSsSS | 1.8282 | TBI | TBI 20 |
| AFF3 | QPNcRTSVPSSKGSsSS | 1.8282 | TBI | TBI 20 |
| AFF3 | QPNcRTSVPSSKGSsSS | 1.8282 | TBI | TBI 20 |
| AFF3 | QPNcRTSVPSSKGSsSS | 1.8282 | TBI | TBI 20 |
| AFF3 | QPNcRTSVPSSKGSsSS | 1.8282 | TBI | TBI 20 |
| AFF3 | QPNcRTSVPSSKGSsSS | 1.8282 | TBI | TBI 20 |
| AFF3 | QPNcRTSVPSSKGSsSS | 1.8282 | TBI | TBI 20 |
| AFF3 | QPNcRTSVPSSKGSsSS | 1.8282 | TBI | TBI 20 |
| AFF3 | QPNcRTSVPSSKGSsSS | 1.8282 | TBI | TBI 20 |
| AFF3 | QPNcRTSVPSSKGSsSS | 1.8282 | TBI | TBI 20 |
| AFF3 | QPNcRTSVPSSKGSsSS | 1.8282 | TBI | TBI 20 |
| AFF3 | QPNcRTSVPSSKGSsSS | 1.8282 | TBI | TBI 20 |
| AFF3 | QPNcRTSVPSSKGSsSS | 1.8282 | TBI | TBI 20 |
| AFF3 | QPNcRTSVPSSKGSsSS | 1.8282 | TBI | TBI 20 |
| AFF3 | QPNcRTSVPSSKGSsSS | 1.8282 | TBI | TBI 20 |
| AFF3 | QPNcRTSVPSSKGSsSS | 1.8282 | TBI | TBI 20 |
| AFF3 | QPNcRTSVPSSKGSsSS | 1.8282 | TBI | TBI 20 |
| FREM2 | tLGmNFTQDEVDRNL | 1.8275 | TBI | TBI 20 |
| FREM2 | tLGmNFTQDEVDRNL | 1.8275 | TBI | TBI 20 |
| FREM2 | tLGmNFTQDEVDRNL | 1.8275 | TBI | TBI 20 |
| FREM2 | tLGmNFTQDEVDRNL | 1.8275 | TBI | TBI 20 |
| FREM2 | tLGmNFTQDEVDRNL | 1.8275 | TBI | TBI 20 |
| MCRS1 | SLcPcccLAGETLVSQAPT | 1.825 | TBI | TBI 20 |
| ARID1B | NsSmQDMYNQSPSGAMS | 1.8094 | TBI | TBI 20 |
| ARID1B | NSsmQDMYNQSPSGAMS | 1.8094 | TBI | TBI 20 |
| ARID1B | NsSmQDMYNQSPSGAMS | 1.8094 | TBI | TBI 20 |
| ARID1B | NSsmQDMYNQSPSGAMS | 1.8094 | TBI | TBI 20 |
| ARID1B | NsSmQDMYNQSPSGAMS | 1.8094 | TBI | TBI 20 |
| ARID1B | NSsmQDMYNQSPSGAMS | 1.8094 | TBI | TBI 20 |
| ARID1B | NsSmQDMYNQSPSGAMS | 1.8094 | TBI | TBI 20 |
| ARID1B | NSsmQDMYNQSPSGAMS | 1.8094 | TBI | TBI 20 |
| ARID1B | NsSmQDMYNQSPSGAMS | 1.8094 | TBI | TBI 20 |
| ARID1B | NSsmQDMYNQSPSGAMS | 1.8094 | TBI | TBI 20 |
| ARID1B | NsSmQDMYNQSPSGAMS | 1.8094 | TBI | TBI 20 |
| ARID1B | NsSmQDMYNQSPSGAMS | 1.8094 | TBI | TBI 20 |
| ARID1B | NSsmQDMYNQSPSGAMS | 1.8094 | TBI | TBI 20 |
| MDFIC | CNCPcDMDCGIMDAccES | 1.8034 | TBI | TBI 20 |
| MDFIC | CNCPcDMDCGIMDAccES | 1.8034 | TBI | TBI 20 |
| MDFIC | CNCPcDMDCGIMDAccES | 1.8034 | TBI | TBI 20 |
| PSD3 | GTSsGTFSPVRLDESGE | 1.8006 | TBI | TBI 20 |
| PSD3 | GTSsGTFSPVRLDESGE | 1.8006 | TBI | TBI 20 |
| PSD3 | GTSsGTFSPVRLDESGE | 1.8006 | TBI | TBI 20 |
| PSD3 | GTSsGTFSPVRLDESGE | 1.8006 | TBI | TBI 20 |
| PSD3 | GTSsGTFSPVRLDESGE | 1.8006 | TBI | TBI 20 |
| PSD3 | GTSsGTFSPVRLDESGE | 1.8006 | TBI | TBI 20 |
| PSD3 | GTSsGTFSPVRLDESGE | 1.8006 | TBI | TBI 20 |
| PSD3 | GTSsGTFSPVRLDESGE | 1.8006 | TBI | TBI 20 |
| PSD3 | GTSsGTFSPVRLDESGE | 1.8006 | TBI | TBI 20 |
| PSD3 | GTSsGTFSPVRLDESGE | 1.8006 | TBI | TBI 20 |
| PSD3 | GTSsGTFSPVRLDESGE | 1.8006 | TBI | TBI 20 |
| ARID1B | NSsmQDMYNQSPSGAMS | 1.7994 | TBI | TBI 20 |
| LSM12 | VVITPPyQVENcKGKEG | 1.7867 | TBI | TBI 20 |
| LSM12 | VVITPPyQVENcKGKEG | 1.7867 | TBI | TBI 20 |
| LSM12 | VVITPPyQVENcKGKEG | 1.7867 | TBI | TBI 20 |
| FBN2 | cDMHASCLNIPGSFKCSC | 1.7722 | TBI | TBI 20 |
| FBN2 | cDMHASCLNIPGSFKCSC | 1.7722 | TBI | TBI 20 |
| FBN2 | cDMHASCLNIPGSFKCSC | 1.7722 | TBI | TBI 20 |
| FBN2 | cDMHASCLNIPGSFKCSC | 1.7722 | TBI | TBI 20 |
| FBN2 | cDMHASCLNIPGSFKCSC | 1.7722 | TBI | TBI 20 |
| FBN2 | cDMHASCLNIPGSFKCSC | 1.7722 | TBI | TBI 20 |
| FBN2 | cDMHASCLNIPGSFKCSC | 1.7722 | TBI | TBI 20 |
| FBN2 | cDMHASCLNIPGSFKCSC | 1.7722 | TBI | TBI 20 |
| FBN2 | cDMHASCLNIPGSFKCSC | 1.7722 | TBI | TBI 20 |
| FBN2 | cDMHASCLNIPGSFKCSC | 1.7722 | TBI | TBI 20 |
| FBN2 | cDMHASCLNIPGSFKCSC | 1.7722 | TBI | TBI 20 |
| FBN2 | cDMHASCLNIPGSFKCSC | 1.7722 | TBI | TBI 20 |
| FBN2 | cDMHASCLNIPGSFKCSC | 1.7722 | TBI | TBI 20 |
| FBN2 | cDMHASCLNIPGSFKCSC | 1.7722 | TBI | TBI 20 |
| CCDC148 | HmKNEMRNIKyKPV | 1.7611 | TBI | TBI 20 |
| CCDC148 | HmKNEMRNIKyKPV | 1.7611 | TBI | TBI 20 |
| CCDC148 | HmKNEMRNIKyKPV | 1.7611 | TBI | TBI 20 |
| CCDC148 | HmKNEMRNIKyKPV | 1.7611 | TBI | TBI 20 |
| CCDC148 | HmKNEMRNIKyKPV | 1.7611 | TBI | TBI 20 |
| CCDC148 | HmKNEMRNIKyKPV | 1.7611 | TBI | TBI 20 |
| CCDC148 | HmKNEMRNIKyKPV | 1.7611 | TBI | TBI 20 |
| CCDC148 | HmKNEMRNIKyKPV | 1.7611 | TBI | TBI 20 |
| CCDC148 | HmKNEMRNIKyKPV | 1.7611 | TBI | TBI 20 |
| CCDC148 | HmKNEMRNIKyKPV | 1.7611 | TBI | TBI 20 |
| CCDC148 | HmKNEMRNIKyKPV | 1.7611 | TBI | TBI 20 |
| MAPRE2 | SGStQcAGSVPTGAAYcQF | 1.7559 | TBI | TBI 20 |
| MAPRE2 | SGStQcAGSVPTGAAYcQF | 1.7559 | TBI | TBI 20 |
| MAPRE2 | SGStQcAGSVPTGAAYcQF | 1.7559 | TBI | TBI 20 |
| MAPRE2 | SGStQcAGSVPTGAAYcQF | 1.7559 | TBI | TBI 20 |
| MAPRE2 | SGStQcAGSVPTGAAYcQF | 1.7559 | TBI | TBI 20 |
| EBF4 | QsLHGPMFKNTSVGPLY | 1.7553 | TBI | TBI 20 |
| EBF4 | QsLHGPMFKNTSVGPLY | 1.7553 | TBI | TBI 20 |
| EBF4 | QsLHGPMFKNTSVGPLY | 1.7553 | TBI | TBI 20 |
| MAPRE2 | SGsTQcAGSVPTGAAYcQF | 1.7367 | TBI | TBI 20 |
| MAPRE2 | SGsTQcAGSVPTGAAYcQF | 1.7367 | TBI | TBI 20 |
| MAPRE2 | SGsTQcAGSVPTGAAYcQF | 1.7367 | TBI | TBI 20 |
| MAPRE2 | SGsTQcAGSVPTGAAYcQF | 1.7367 | TBI | TBI 20 |
| MAPRE2 | SGsTQcAGSVPTGAAYcQF | 1.7367 | TBI | TBI 20 |
| TENM1 | yVACDNTGtPLAVFSS | 1.7228 | TBI | TBI 20 |
| TENM1 | yVACDNTGtPLAVFSS | 1.7228 | TBI | TBI 20 |
| TENM1 | yVACDNTGtPLAVFSS | 1.7228 | TBI | TBI 20 |
| TENM1 | yVACDNTGtPLAVFSS | 1.7228 | TBI | TBI 20 |
| TENM1 | yVACDNTGtPLAVFSS | 1.7228 | TBI | TBI 20 |
| TENM1 | yVACDNTGtPLAVFSS | 1.7228 | TBI | TBI 20 |
| TENM1 | yVACDNTGtPLAVFSS | 1.7228 | TBI | TBI 20 |
| C7 | SLVCNGDSDcDEDs | 1.7174 | TBI | TBI 20 |
| C7 | SLVCNGDSDcDEDs | 1.7174 | TBI | TBI 20 |
| C7 | SLVCNGDSDcDEDs | 1.7174 | TBI | TBI 20 |
| C7 | SLVCNGDSDcDEDs | 1.7174 | TBI | TBI 20 |
| C7 | SLVCNGDSDcDEDs | 1.7174 | TBI | TBI 20 |
| TNFRSF14 | APPRPTPSPFLVtCLQ | 1.7171 | TBI | TBI 20 |
| TNFRSF14 | APPRPTPSPFLVtCLQ | 1.7171 | TBI | TBI 20 |
| TNFRSF14 | APPRPTPSPFLVtCLQ | 1.7171 | TBI | TBI 20 |
| ING2 | ScVSLTyKPKGKWYCP | 1.7029 | TBI | TBI 20 |
| ING2 | ScVSLTyKPKGKWYCP | 1.7029 | TBI | TBI 20 |
| ING2 | ScVSLTyKPKGKWYCP | 1.7029 | TBI | TBI 20 |
| ING2 | ScVSLTyKPKGKWYCP | 1.7029 | TBI | TBI 20 |
| ING2 | ScVSLTyKPKGKWYCP | 1.7029 | TBI | TBI 20 |
| ING2 | ScVSLTyKPKGKWYCP | 1.7029 | TBI | TBI 20 |
| ING2 | ScVSLTyKPKGKWYCP | 1.7029 | TBI | TBI 20 |
| FBN2 | ctTTNmcLNGMCINEDG | 3.3564 | TBI | TBI 21 |
| FBN2 | ctTTNmcLNGMCINEDG | 3.3564 | TBI | TBI 21 |
| FBN2 | ctTTNmcLNGMCINEDG | 3.3564 | TBI | TBI 21 |
| FBN2 | ctTTNmcLNGMCINEDG | 3.3564 | TBI | TBI 21 |
| FBN2 | ctTTNmcLNGMCINEDG | 3.3564 | TBI | TBI 21 |
| FBN2 | ctTTNmcLNGMCINEDG | 3.3564 | TBI | TBI 21 |
| FBN2 | ctTTNmcLNGMCINEDG | 3.3564 | TBI | TBI 21 |
| FBN2 | ctTTNmcLNGMCINEDG | 3.3564 | TBI | TBI 21 |
| FBN2 | ctTTNmcLNGMCINEDG | 3.3564 | TBI | TBI 21 |
| FBN2 | ctTTNmcLNGMCINEDG | 3.3564 | TBI | TBI 21 |
| FBN2 | ctTTNmcLNGMCINEDG | 3.3564 | TBI | TBI 21 |
| FBN2 | ctTTNmcLNGMCINEDG | 3.3564 | TBI | TBI 21 |
| FBN2 | ctTTNmcLNGMCINEDG | 3.3564 | TBI | TBI 21 |
| FBN2 | ctTTNmcLNGMCINEDG | 3.3564 | TBI | TBI 21 |
| FBN2 | ctTTNmcLNGMCINEDG | 3.3564 | TBI | TBI 21 |
| FBN2 | cDMHASCLNIPGSFKCSC | 3.3564 | TBI | TBI 21 |
| FBN2 | cTtTNmcLNGMCINEDG | 3.3564 | TBI | TBI 21 |
| FBN2 | ctTTNmcLNGMCINEDG | 3.3564 | TBI | TBI 21 |
| FREM2 | DPPLYGEILVNG | 3.1027 | TBI | TBI 21 |
| FREM2 | tLGmNFTQDEVDRNL | 3.1027 | TBI | TBI 21 |
| FREM2 | DPPLYGEILVNG | 3.1027 | TBI | TBI 21 |
| FREM2 | tLGmNFTQDEVDRNL | 3.1027 | TBI | TBI 21 |
| FREM2 | DPPLYGEILVNG | 3.1027 | TBI | TBI 21 |
| FREM2 | tLGmNFTQDEVDRNL | 3.1027 | TBI | TBI 21 |
| FREM2 | DPPLYGEILVNG | 3.1027 | TBI | TBI 21 |
| FREM2 | tLGmNFTQDEVDRNL | 3.1027 | TBI | TBI 21 |
| CACNA2D1 | RDPcAGPVcDCKRN | 2.9126 | TBI | TBI 21 |
| CACNA2D1 | RDPcAGPVcDCKRN | 2.9126 | TBI | TBI 21 |
| CACNA2D1 | RDPcAGPVcDCKRN | 2.9126 | TBI | TBI 21 |
| CACNA2D1 | RDPcAGPVcDCKRN | 2.9126 | TBI | TBI 21 |
| CACNA2D1 | RDPcAGPVcDCKRN | 2.9126 | TBI | TBI 21 |
| CACNA2D1 | TDcGGVsGLNPSLWYIIG | 2.9126 | TBI | TBI 21 |
| VLDLR | DEVNcPSRTc | 2.442 | TBI | TBI 21 |
| VLDLR | DISKVcNQEQDCR | 2.442 | TBI | TBI 21 |
| VLDLR | DEVNcPSRTc | 2.442 | TBI | TBI 21 |
| VLDLR | DEVNcPSRTc | 2.442 | TBI | TBI 21 |
| VLDLR | DEVNcPSRTc | 2.442 | TBI | TBI 21 |
| VLDLR | DEVNcPSRTc | 2.442 | TBI | TBI 21 |
| MUC6 | LCPSQPQSVPGSNIEGCY | 2.2642 | TBI | TBI 21 |
| MUC6 | LCPSQPQSVPGSNIEGCY | 2.2642 | TBI | TBI 21 |
| MUC6 | LCPSQPQSVPGSNIEGCY | 2.2642 | TBI | TBI 21 |
| CCDC18 | ADLKRQKVIELtGTAR | 2.2437 | TBI | TBI 21 |
| CCDC18 | ADLKRQKVIELtGTAR | 2.2437 | TBI | TBI 21 |
| CCDC18 | ADLKRQKVIELTGtAR | 2.2437 | TBI | TBI 21 |
| CCDC18 | ADLKRQKVIELtGTAR | 2.2437 | TBI | TBI 21 |
| CCDC18 | ADLKRQKVIELTGtAR | 2.2437 | TBI | TBI 21 |
| CCDC18 | ADLKRQKVIELTGtAR | 2.2437 | TBI | TBI 21 |
| CCDC18 | ADLKRQKVIELTGtAR | 2.2437 | TBI | TBI 21 |
| CCDC18 | ADLKRQKVIELtGTAR | 2.2437 | TBI | TBI 21 |
| CCDC18 | ADLKRQKVIELTGtAR | 2.2437 | TBI | TBI 21 |
| HUWE1 | PTTATTTVSISPtTKGSKS | 2.243 | TBI | TBI 21 |
| HUWE1 | PTTATTTVSISPTtKGSKS | 2.243 | TBI | TBI 21 |
| HUWE1 | PTTATTTVSISPTtKGSKS | 2.243 | TBI | TBI 21 |
| HUWE1 | PTTATTTVSISPTtKGSKS | 2.243 | TBI | TBI 21 |
| HUWE1 | PTTATTTVSISPtTKGSKS | 2.243 | TBI | TBI 21 |
| HUWE1 | PTTATTTVSISPtTKGSKS | 2.243 | TBI | TBI 21 |
| HUWE1 | PTTATTTVSISPtTKGSKS | 2.243 | TBI | TBI 21 |
| HUWE1 | PTTATTTVSISPTtKGSKS | 2.243 | TBI | TBI 21 |
| HUWE1 | PTTATTTVSISPtTKGSKS | 2.243 | TBI | TBI 21 |
| TBX22 | RSSAAGKSEPLEKQPKTE | 2.1815 | TBI | TBI 21 |
| TBX22 | RSSAAGKSEPLEKQPKTE | 2.1815 | TBI | TBI 21 |
| TBX22 | RSSAAGKSEPLEKQPKTE | 2.1815 | TBI | TBI 21 |
| MUC4 | PVTSPSSASTGHAtPLPVTG | 2.1461 | TBI | TBI 21 |
| MUC4 | PVTSPSSASTGHAtPLPVTG | 2.1461 | TBI | TBI 21 |
| MUC4 | PVTSPSSASTGHAtPLPVTG | 2.1461 | TBI | TBI 21 |
| PADI3 | SSyDAKRAQVFHIc | 2.1434 | TBI | TBI 21 |
| PADI3 | SSyDAKRAQVFHIc | 2.1434 | TBI | TBI 21 |
| PADI3 | SsYDAKRAQVFHIc | 2.1434 | TBI | TBI 21 |
| PADI3 | SsYDAKRAQVFHIc | 2.1434 | TBI | TBI 21 |
| PADI3 | SsYDAKRAQVFHIc | 2.1434 | TBI | TBI 21 |
| PADI3 | SSyDAKRAQVFHIc | 2.1434 | TBI | TBI 21 |
| PADI3 | SsYDAKRAQVFHIc | 2.1434 | TBI | TBI 21 |
| PADI3 | SsYDAKRAQVFHIc | 2.1434 | TBI | TBI 21 |
| STAT1 | LETTSLPVVVIsNVSQL | 2.134 | TBI | TBI 21 |
| STAT1 | LETTSLPVVVIsNVSQL | 2.134 | TBI | TBI 21 |
| STAT1 | LETTSLPVVVIsNVSQL | 2.134 | TBI | TBI 21 |
| STAT1 | LETTSLPVVVIsNVSQL | 2.134 | TBI | TBI 21 |
| STAT1 | LETTSLPVVVIsNVSQL | 2.134 | TBI | TBI 21 |
| STAT1 | LETTSLPVVVIsNVSQL | 2.134 | TBI | TBI 21 |
| STAT1 | LETTSLPVVVIsNVSQL | 2.134 | TBI | TBI 21 |
| RUNX2 | LQPGKmSDVSPQQQQQQ | 2.1286 | TBI | TBI 21 |
| RUNX2 | LQPGKmSDVSPQQQQQQ | 2.1286 | TBI | TBI 21 |
| RUNX2 | LQPGKmSDVSPQQQQQQ | 2.1286 | TBI | TBI 21 |
| SLC25A40 | QMLAsCTGAILTSVIVTP | 2.1166 | TBI | TBI 21 |
| SLC25A40 | QMLAsCTGAILTSVIVTP | 2.1166 | TBI | TBI 21 |
| SLC25A40 | QMLAsCTGAILTSVIVTP | 2.1166 | TBI | TBI 21 |
| SLC25A40 | QMLAsCTGAILTSVIVTP | 2.1166 | TBI | TBI 21 |
| SLC25A40 | QMLAsCTGAILTSVIVTP | 2.1166 | TBI | TBI 21 |
| SLC25A40 | QMLAsCTGAILTSVIVTP | 2.1166 | TBI | TBI 21 |
| DKC1 | sESAKKEVVAEVVKAPQ | 2.1141 | TBI | TBI 21 |
| DKC1 | sESAKKEVVAEVVKAPQ | 2.1141 | TBI | TBI 21 |
| DKC1 | SEsAKKEVVAEVVKAPQ | 2.1141 | TBI | TBI 21 |
| DKC1 | sESAKKEVVAEVVKAPQ | 2.1141 | TBI | TBI 21 |
| DKC1 | sESAKKEVVAEVVKAPQ | 2.1141 | TBI | TBI 21 |
| DKC1 | sESAKKEVVAEVVKAPQ | 2.1141 | TBI | TBI 21 |
| DKC1 | sESAKKEVVAEVVKAPQ | 2.1141 | TBI | TBI 21 |
| DKC1 | sESAKKEVVAEVVKAPQ | 2.1141 | TBI | TBI 21 |
| DKC1 | SEsAKKEVVAEVVKAPQ | 2.1141 | TBI | TBI 21 |
| DKC1 | SEsAKKEVVAEVVKAPQ | 2.1141 | TBI | TBI 21 |
| DKC1 | SEsAKKEVVAEVVKAPQ | 2.1141 | TBI | TBI 21 |
| DKC1 | sESAKKEVVAEVVKAPQ | 2.1141 | TBI | TBI 21 |
| DKC1 | SEsAKKEVVAEVVKAPQ | 2.1141 | TBI | TBI 21 |
| REEP6 | QRNLVTEVLGALEAKtG | 2.1121 | TBI | TBI 21 |
| REEP6 | QRNLVTEVLGALEAKtG | 2.1121 | TBI | TBI 21 |
| REEP6 | QRNLVTEVLGALEAKtG | 2.1121 | TBI | TBI 21 |
| REEP6 | QRNLVTEVLGALEAKtG | 2.1121 | TBI | TBI 21 |
| REEP6 | QRNLVTEVLGALEAKtG | 2.1121 | TBI | TBI 21 |
| REEP6 | QRNLVTEVLGALEAKtG | 2.1121 | TBI | TBI 21 |
| DKC1 | sESAKKEVVAEVVKAPQ | 2.0919 | TBI | TBI 21 |
| DKC1 | sESAKKEVVAEVVKAPQ | 2.0919 | TBI | TBI 21 |
| DKC1 | sESAKKEVVAEVVKAPQ | 2.0919 | TBI | TBI 21 |
| GLRB | EDIEYGNcTKyYKGT | 2.0804 | TBI | TBI 21 |
| GLRB | EDIEYGNcTKyYKGT | 2.0804 | TBI | TBI 21 |
| GLRB | EDIEYGNcTKyYKGT | 2.0804 | TBI | TBI 21 |
| GLRB | EDIEYGNcTKyYKGT | 2.0804 | TBI | TBI 21 |
| HUWE1 | PTTATTTVSISPTtKGSKS | 2.0698 | TBI | TBI 21 |
| HUWE1 | PTTATTTVSISPTtKGSKS | 2.0698 | TBI | TBI 21 |
| HUWE1 | PTTATTTVSISPTtKGSKS | 2.0698 | TBI | TBI 21 |
| CCDC18 | ADLKRQKVIELtGTAR | 2.0571 | TBI | TBI 21 |
| CCDC18 | ADLKRQKVIELtGTAR | 2.0571 | TBI | TBI 21 |
| CCDC18 | ADLKRQKVIELtGTAR | 2.0571 | TBI | TBI 21 |
| NFASC | EQSIWNVtVLPNSKW | 2.0535 | TBI | TBI 21 |
| NFASC | EQSIWNVtVLPNSKW | 2.0535 | TBI | TBI 21 |
| NFASC | EQSIWNVtVLPNSKW | 2.0535 | TBI | TBI 21 |
| NFASC | EQSIWNVtVLPNSKW | 2.0535 | TBI | TBI 21 |
| MUC19 | TGLPGESAEVtGTIGSPAGV | 2.0475 | TBI | TBI 21 |
| MUC19 | TGLPGESAEVtGTIGSPAGV | 2.0475 | TBI | TBI 21 |
| MUC19 | TGLPGESAEVtGTIGSPAGV | 2.0475 | TBI | TBI 21 |
| MUC19 | TGLPGESAEVtGTIGSPAGV | 2.0475 | TBI | TBI 21 |
| FBXO34 | PFAcGIEHCsVHYVSDS | 2.0397 | TBI | TBI 21 |
| FBXO34 | PFAcGIEHCsVHYVSDS | 2.0397 | TBI | TBI 21 |
| FBXO34 | PFAcGIEHCsVHYVSDS | 2.0397 | TBI | TBI 21 |
| FBXO34 | PFAcGIEHCsVHYVSDS | 2.0397 | TBI | TBI 21 |
| MCRS1 | SLcPcccLAGETLVSQAPT | 2.0225 | TBI | TBI 21 |
| MCRS1 | SLcPcccLAGETLVSQAPT | 2.0225 | TBI | TBI 21 |
| MCRS1 | SLcPcccLAGETLVSQAPT | 2.0225 | TBI | TBI 21 |
| MCRS1 | SLcPcccLAGETLVSQAPT | 2.0225 | TBI | TBI 21 |
| CA3 | TKGKEAPFTKFDPsCL | 2.0204 | TBI | TBI 21 |
| CA3 | TKGKEAPFTKFDPsCL | 2.0204 | TBI | TBI 21 |
| CA3 | TKGKEAPFTKFDPsCL | 2.0204 | TBI | TBI 21 |
| CA3 | TKGKEAPFTKFDPsCL | 2.0204 | TBI | TBI 21 |
| CA3 | TKGKEAPFTKFDPsCL | 2.0204 | TBI | TBI 21 |
| MCRS1 | SLcPcccLAGETLVSQAPT | 2.0045 | TBI | TBI 21 |
| SLC12A1 | EmNsGmAKKQAWLIK | 1.9917 | TBI | TBI 21 |
| SLC12A1 | EmNsGmAKKQAWLIK | 1.9917 | TBI | TBI 21 |
| SLC12A1 | EmNsGmAKKQAWLIK | 1.9917 | TBI | TBI 21 |
| SLC12A1 | EmNsGmAKKQAWLIK | 1.9917 | TBI | TBI 21 |
| SLC12A1 | EmNsGmAKKQAWLIK | 1.9917 | TBI | TBI 21 |
| SLC12A1 | EmNsGmAKKQAWLIK | 1.9917 | TBI | TBI 21 |
| SLC12A1 | EmNsGmAKKQAWLIK | 1.9917 | TBI | TBI 21 |
| SLC12A1 | EmNsGmAKKQAWLIK | 1.9917 | TBI | TBI 21 |
| PKD1 | mLILQAETTAGTVTPTAI | 1.9855 | TBI | TBI 21 |
| PKD1 | mLILQAETTAGTVTPTAI | 1.9855 | TBI | TBI 21 |
| PKD1 | mLILQAETTAGTVTPTAI | 1.9855 | TBI | TBI 21 |
| PKD1 | mLILQAETTAGTVTPTAI | 1.9855 | TBI | TBI 21 |
| MAPRE2 | sGSTQcAGSVPTGAAYcQF | 1.9851 | TBI | TBI 21 |
| MAPRE2 | SGStQcAGSVPTGAAYcQF | 1.9851 | TBI | TBI 21 |
| MAPRE2 | SGsTQcAGSVPTGAAYcQF | 1.9851 | TBI | TBI 21 |
| MAPRE2 | sGSTQcAGSVPTGAAYcQF | 1.9851 | TBI | TBI 21 |
| MAPRE2 | sGSTQcAGSVPTGAAYcQF | 1.9851 | TBI | TBI 21 |
| MAPRE2 | sGSTQcAGSVPTGAAYcQF | 1.9851 | TBI | TBI 21 |
| MAPRE2 | SGsTQcAGSVPTGAAYcQF | 1.9851 | TBI | TBI 21 |
| MAPRE2 | sGSTQcAGSVPTGAAYcQF | 1.9851 | TBI | TBI 21 |
| MAPRE2 | sGSTQcAGSVPTGAAYcQF | 1.9851 | TBI | TBI 21 |
| MAPRE2 | SGsTQcAGSVPTGAAYcQF | 1.9851 | TBI | TBI 21 |
| MAPRE2 | sGSTQcAGSVPTGAAYcQF | 1.9851 | TBI | TBI 21 |
| SLAMF7 | YGLLHcGNTEKDGKSPL | 1.9619 | TBI | TBI 21 |
| SLAMF7 | YGLLHcGNTEKDGKSPL | 1.9619 | TBI | TBI 21 |
| SLAMF7 | YGLLHcGNTEKDGKSPL | 1.9619 | TBI | TBI 21 |
| SLAMF7 | YGLLHcGNTEKDGKSPL | 1.9619 | TBI | TBI 21 |
| SLAMF7 | YGLLHcGNTEKDGKSPL | 1.9619 | TBI | TBI 21 |
| EPS8 | EGARVySQITVQKAALE | 1.9618 | TBI | TBI 21 |
| EPS8 | EGARVySQITVQKAALE | 1.9618 | TBI | TBI 21 |
| EPS8 | EGARVySQITVQKAALE | 1.9618 | TBI | TBI 21 |
| EPS8 | EGARVySQITVQKAALE | 1.9618 | TBI | TBI 21 |
| EPS8 | EGARVySQITVQKAALE | 1.9618 | TBI | TBI 21 |
| MUC17 | EGSTPLTRMPVSTtMVAS | 1.9555 | TBI | TBI 21 |
| MUC17 | EGSTPLTRMPVSTtMVAS | 1.9555 | TBI | TBI 21 |
| MUC17 | EGSTPLTRMPVSTtMVAS | 1.9555 | TBI | TBI 21 |
| NFKB2 | cSTKVKTLLLNAAQNtM | 1.9533 | TBI | TBI 21 |
| NFKB2 | cSTKVKTLLLNAAQNtM | 1.9533 | TBI | TBI 21 |
| NFKB2 | cSTKVKTLLLNAAQNtM | 1.9533 | TBI | TBI 21 |
| NSD3 | LPmIPsSSASKKKcEKGG | 1.9465 | TBI | TBI 21 |
| NSD3 | LPmIPsSSASKKKcEKGG | 1.9465 | TBI | TBI 21 |
| NSD3 | LPmIPsSSASKKKcEKGG | 1.9465 | TBI | TBI 21 |
| NSD3 | LPmIPsSSASKKKcEKGG | 1.9465 | TBI | TBI 21 |
| SLC23A1 | PcQWGLPtVTAAAVLGMF | 1.9359 | TBI | TBI 21 |
| SLC23A1 | PcQWGLPtVTAAAVLGMF | 1.9359 | TBI | TBI 21 |
| SLC23A1 | PcQWGLPtVTAAAVLGMF | 1.9359 | TBI | TBI 21 |
| SLC23A1 | PcQWGLPtVTAAAVLGMF | 1.9359 | TBI | TBI 21 |
| SLC23A1 | PcQWGLPtVTAAAVLGMF | 1.9359 | TBI | TBI 21 |
| MAN1B1 | RTPSKIPYSDVNIGtGV | 1.9252 | TBI | TBI 21 |
| MAN1B1 | RTPSKIPYsDVNIGTGV | 1.9252 | TBI | TBI 21 |
| MAN1B1 | RTPSKIPYSDVNIGtGV | 1.9252 | TBI | TBI 21 |
| MAN1B1 | RTPSKIPYsDVNIGTGV | 1.9252 | TBI | TBI 21 |
| MAN1B1 | RTPSKIPYSDVNIGtGV | 1.9252 | TBI | TBI 21 |
| MAN1B1 | RTPSKIPYsDVNIGTGV | 1.9252 | TBI | TBI 21 |
| MAN1B1 | RTPSKIPYSDVNIGtGV | 1.9252 | TBI | TBI 21 |
| MAN1B1 | RTPSKIPYsDVNIGTGV | 1.9252 | TBI | TBI 21 |
| MAN1B1 | RTPSKIPYSDVNIGtGV | 1.9252 | TBI | TBI 21 |
| MAN1B1 | RTPSKIPYsDVNIGTGV | 1.9252 | TBI | TBI 21 |
| MAN1B1 | RTPSKIPYSDVNIGtGV | 1.9252 | TBI | TBI 21 |
| MAN1B1 | RTPSKIPYsDVNIGTGV | 1.9252 | TBI | TBI 21 |
| MAN1B1 | RTPSKIPYSDVNIGtGV | 1.9252 | TBI | TBI 21 |
| MAN1B1 | RTPSKIPYsDVNIGTGV | 1.9252 | TBI | TBI 21 |
| MAN1B1 | RTPSKIPYSDVNIGtGV | 1.9252 | TBI | TBI 21 |
| MAN1B1 | RTPSKIPYSDVNIGtGV | 1.9252 | TBI | TBI 21 |
| MAN1B1 | RTPSKIPYsDVNIGTGV | 1.9252 | TBI | TBI 21 |
| ABCG5 | VESGQIMcILGSsGSGKT | 1.9203 | TBI | TBI 21 |
| ABCG5 | VESGQIMcILGSSGsGKT | 1.9203 | TBI | TBI 21 |
| ABCG5 | VESGQIMcILGSsGSGKT | 1.9203 | TBI | TBI 21 |
| ABCG5 | VESGQIMcILGSsGSGKT | 1.9203 | TBI | TBI 21 |
| ABCG5 | VESGQIMcILGSSGsGKT | 1.9203 | TBI | TBI 21 |
| AFF3 | QPNcRTSVPsSKGSSSS | 1.9111 | TBI | TBI 21 |
| AFF3 | QPNcRTSVPSSKGSsSS | 1.9111 | TBI | TBI 21 |
| AFF3 | QPNcRTSVPSSKGSsSS | 1.9111 | TBI | TBI 21 |
| AFF3 | QPNcRTSVPSSKGSsSS | 1.9111 | TBI | TBI 21 |
| AFF3 | QPNcRTSVPsSKGSSSS | 1.9111 | TBI | TBI 21 |
| AFF3 | QPNcRTSVPSSKGSsSS | 1.9111 | TBI | TBI 21 |
| AFF3 | QPNcRTSVPSSKGSsSS | 1.9111 | TBI | TBI 21 |
| AFF3 | QPNcRTSVPSSKGSsSS | 1.9111 | TBI | TBI 21 |
| AFF3 | QPNcRTSVPsSKGSSSS | 1.9111 | TBI | TBI 21 |
| AFF3 | QPNcRTSVPSSKGsSSS | 1.9111 | TBI | TBI 21 |
| AFF3 | QPNcRTSVPSsKGSSSS | 1.9111 | TBI | TBI 21 |
| AFF3 | QPNcRTSVPSSKGSsSS | 1.9111 | TBI | TBI 21 |
| AFF3 | QPNcRTSVPsSKGSSSS | 1.9111 | TBI | TBI 21 |
| AFF3 | QPNcRTSVPSSKGSsSS | 1.9111 | TBI | TBI 21 |
| AFF3 | QPNcRTSVPSSKGSsSS | 1.9111 | TBI | TBI 21 |
| AFF3 | QPNcRTSVPSSKGSsSS | 1.9111 | TBI | TBI 21 |
| AFF3 | QPNcRTSVPsSKGSSSS | 1.9111 | TBI | TBI 21 |
| AFF3 | QPNcRTSVPSSKGSsSS | 1.9111 | TBI | TBI 21 |
| AFF3 | QPNcRTSVPSSKGSsSS | 1.9111 | TBI | TBI 21 |
| AFF3 | QPNcRTSVPSSKGSsSS | 1.9111 | TBI | TBI 21 |
| AFF3 | QPNcRTSVPsSKGSSSS | 1.9111 | TBI | TBI 21 |
| AFF3 | QPNcRTSVPsSKGSSSS | 1.9111 | TBI | TBI 21 |
| AFF3 | QPNcRTSVPsSKGSSSS | 1.9111 | TBI | TBI 21 |
| AFF3 | QPNcRTSVPsSKGSSSS | 1.9111 | TBI | TBI 21 |
| AFF3 | QPNcRTSVPSSKGSsSS | 1.9111 | TBI | TBI 21 |
| AFF3 | QPNcRTSVPsSKGSSSS | 1.9111 | TBI | TBI 21 |
| AFF3 | QPNcRTSVPsSKGSSSS | 1.9111 | TBI | TBI 21 |
| AFF3 | QPNcRTSVPsSKGSSSS | 1.9111 | TBI | TBI 21 |
| AFF3 | QPNcRTSVPsSKGSSSS | 1.9111 | TBI | TBI 21 |
| AFF3 | QPNcRTSVPsSKGSSSS | 1.9111 | TBI | TBI 21 |
| AFF3 | QPNcRTSVPsSKGSSSS | 1.9111 | TBI | TBI 21 |
| AFF3 | QPNcRTSVPSSKGSsSS | 1.9111 | TBI | TBI 21 |
| AFF3 | QPNcRTSVPsSKGSSSS | 1.9111 | TBI | TBI 21 |
| AFF3 | QPNcRTSVPsSKGSSSS | 1.9111 | TBI | TBI 21 |
| AFF3 | QPNcRTSVPsSKGSSSS | 1.9111 | TBI | TBI 21 |
| AFF3 | QPNcRTSVPsSKGSSSS | 1.9111 | TBI | TBI 21 |
| AFF3 | QPNcRTSVPsSKGSSSS | 1.9111 | TBI | TBI 21 |
| AFF3 | QPNcRTSVPsSKGSSSS | 1.9111 | TBI | TBI 21 |
| AFF3 | QPNcRTSVPSSKGSsSS | 1.9111 | TBI | TBI 21 |
| AFF3 | QPNcRTSVPsSKGSSSS | 1.9111 | TBI | TBI 21 |
| AFF3 | QPNcRTSVPsSKGSSSS | 1.9111 | TBI | TBI 21 |
| AFF3 | QPNcRTSVPsSKGSSSS | 1.9111 | TBI | TBI 21 |
| AFF3 | QPNcRTSVPsSKGSSSS | 1.9111 | TBI | TBI 21 |
| MAN1B1 | RTPSKIPYsDVNIGTGV | 1.9101 | TBI | TBI 21 |
| TECPR1 | KVQGRPSPQAIWSITCKG | 1.9087 | TBI | TBI 21 |
| TECPR1 | KVQGRPSPQAIWSITCKG | 1.9087 | TBI | TBI 21 |
| TECPR1 | KVQGRPSPQAIWSITCKG | 1.9087 | TBI | TBI 21 |
| TECPR1 | KVQGRPSPQAIWSITCKG | 1.9087 | TBI | TBI 21 |
| TECPR1 | KVQGRPSPQAIWSITCKG | 1.9087 | TBI | TBI 21 |
| UBR7 | ELENEAcAVLGGSDsEK | 1.9043 | TBI | TBI 21 |
| UBR7 | ELENEAcAVLGGSDsEK | 1.9043 | TBI | TBI 21 |
| UBR7 | ELENEAcAVLGGSDsEK | 1.9043 | TBI | TBI 21 |
| UBR7 | ELENEAcAVLGGSDsEK | 1.9043 | TBI | TBI 21 |
| UBR7 | ELENEAcAVLGGSDsEK | 1.9043 | TBI | TBI 21 |
| MIPOL1 | YKtKEcKMRITAEEM | 1.9024 | TBI | TBI 21 |
| MIPOL1 | YKtKEcKMRITAEEM | 1.9024 | TBI | TBI 21 |
| MIPOL1 | YKtKEcKMRITAEEM | 1.9024 | TBI | TBI 21 |
| MIPOL1 | YKtKEcKMRITAEEM | 1.9024 | TBI | TBI 21 |
| MIPOL1 | YKtKEcKMRITAEEM | 1.9024 | TBI | TBI 21 |
| MIPOL1 | YKtKEcKMRITAEEM | 1.9024 | TBI | TBI 21 |
| PCDH8 | AIIAIATTCNRRKKEVR | 1.9005 | TBI | TBI 21 |
| PCDH8 | AIIAIATTCNRRKKEVR | 1.9005 | TBI | TBI 21 |
| PCDH8 | AIIAIATTCNRRKKEVR | 1.9005 | TBI | TBI 21 |
| PCDH8 | AIIAIATTCNRRKKEVR | 1.9005 | TBI | TBI 21 |
| PCDH8 | AIIAIATTCNRRKKEVR | 1.9005 | TBI | TBI 21 |
| AFF3 | QPNcRTSVPSSKGsSSS | 1.8915 | TBI | TBI 21 |
| AFF3 | QPNcRTSVPSSKGsSSS | 1.8915 | TBI | TBI 21 |
| AFF3 | QPNcRTSVPSSKGsSSS | 1.8915 | TBI | TBI 21 |
| AFF3 | QPNcRTSVPSSKGsSSS | 1.8915 | TBI | TBI 21 |
| AFF3 | QPNcRTSVPSSKGsSSS | 1.8915 | TBI | TBI 21 |
| AFF3 | QPNcRTSVPSSKGsSSS | 1.8915 | TBI | TBI 21 |
| AFF3 | QPNcRTSVPSSKGsSSS | 1.8915 | TBI | TBI 21 |
| AFF3 | QPNcRTSVPSSKGsSSS | 1.8915 | TBI | TBI 21 |
| AFF3 | QPNcRTSVPSSKGsSSS | 1.8915 | TBI | TBI 21 |
| AFF3 | QPNcRTSVPSSKGsSSS | 1.8915 | TBI | TBI 21 |
| AFF3 | QPNcRTSVPSSKGsSSS | 1.8915 | TBI | TBI 21 |
| AFF3 | QPNcRTSVPSSKGsSSS | 1.8915 | TBI | TBI 21 |
| AFF3 | QPNcRTSVPSSKGsSSS | 1.8915 | TBI | TBI 21 |
| AFF3 | QPNcRTSVPSSKGsSSS | 1.8915 | TBI | TBI 21 |
| AFF3 | QPNcRTSVPSSKGsSSS | 1.8915 | TBI | TBI 21 |
| AFF3 | QPNcRTSVPSSKGsSSS | 1.8915 | TBI | TBI 21 |
| AFF3 | QPNcRTSVPSSKGsSSS | 1.8915 | TBI | TBI 21 |
| AFF3 | QPNcRTSVPSSKGsSSS | 1.8915 | TBI | TBI 21 |
| AFF3 | QPNcRTSVPSSKGsSSS | 1.8915 | TBI | TBI 21 |
| AFF3 | QPNcRTSVPSsKGSSSS | 1.89 | TBI | TBI 21 |
| AFF3 | QPNcRTSVPSsKGSSSS | 1.89 | TBI | TBI 21 |
| AFF3 | QPNcRTSVPSsKGSSSS | 1.89 | TBI | TBI 21 |
| AFF3 | QPNcRTSVPSsKGSSSS | 1.89 | TBI | TBI 21 |
| AFF3 | QPNcRTSVPSsKGSSSS | 1.89 | TBI | TBI 21 |
| AFF3 | QPNcRTSVPSsKGSSSS | 1.89 | TBI | TBI 21 |
| AFF3 | QPNcRTSVPSsKGSSSS | 1.89 | TBI | TBI 21 |
| AFF3 | QPNcRTSVPSsKGSSSS | 1.89 | TBI | TBI 21 |
| AFF3 | QPNcRTSVPSsKGSSSS | 1.89 | TBI | TBI 21 |
| AFF3 | QPNcRTSVPSsKGSSSS | 1.89 | TBI | TBI 21 |
| AFF3 | QPNcRTSVPSsKGSSSS | 1.89 | TBI | TBI 21 |
| AFF3 | QPNcRTSVPSsKGSSSS | 1.89 | TBI | TBI 21 |
| AFF3 | QPNcRTSVPSsKGSSSS | 1.89 | TBI | TBI 21 |
| AFF3 | QPNcRTSVPSsKGSSSS | 1.89 | TBI | TBI 21 |
| AFF3 | QPNcRTSVPSsKGSSSS | 1.89 | TBI | TBI 21 |
| AFF3 | QPNcRTSVPSsKGSSSS | 1.89 | TBI | TBI 21 |
| AFF3 | QPNcRTSVPSsKGSSSS | 1.89 | TBI | TBI 21 |
| AFF3 | QPNcRTSVPSsKGSSSS | 1.89 | TBI | TBI 21 |
| AFF3 | QPNcRTSVPSsKGSSSS | 1.89 | TBI | TBI 21 |
| LOC646383 | SGTECGcILPFLcFLsR | 1.8882 | TBI | TBI 21 |
| LOC646383 | SGTECGcILPFLcFLsR | 1.8882 | TBI | TBI 21 |
| LOC646383 | SGTECGcILPFLcFLsR | 1.8882 | TBI | TBI 21 |
| DNAH14 | PtKCHYMFNLRDMF | 1.8804 | TBI | TBI 21 |
| DNAH14 | PtKCHYMFNLRDMF | 1.8804 | TBI | TBI 21 |
| DNAH14 | PtKCHYMFNLRDMF | 1.8804 | TBI | TBI 21 |
| DNAH14 | PtKCHYMFNLRDMF | 1.8804 | TBI | TBI 21 |
| DNAH14 | PtKCHYMFNLRDMF | 1.8804 | TBI | TBI 21 |
| ING2 | ScVSLTyKPKGKWYCP | 1.8742 | TBI | TBI 21 |
| ING2 | ScVSLTyKPKGKWYCP | 1.8742 | TBI | TBI 21 |
| ING2 | ScVsLTYKPKGKWYCP | 1.8742 | TBI | TBI 21 |
| ING2 | ScVSLTyKPKGKWYCP | 1.8742 | TBI | TBI 21 |
| ING2 | ScVsLTYKPKGKWYCP | 1.8742 | TBI | TBI 21 |
| ING2 | ScVsLTYKPKGKWYCP | 1.8742 | TBI | TBI 21 |
| ING2 | ScVsLTYKPKGKWYCP | 1.8742 | TBI | TBI 21 |
| ING2 | ScVSLTyKPKGKWYCP | 1.8742 | TBI | TBI 21 |
| ING2 | ScVsLTYKPKGKWYCP | 1.8742 | TBI | TBI 21 |
| ING2 | ScVsLTYKPKGKWYCP | 1.8742 | TBI | TBI 21 |
| ING2 | ScVsLTYKPKGKWYCP | 1.8742 | TBI | TBI 21 |
| ING2 | ScVSLTyKPKGKWYCP | 1.8742 | TBI | TBI 21 |
| ING2 | ScVsLTYKPKGKWYCP | 1.8742 | TBI | TBI 21 |
| PADI3 | SSyDAKRAQVFHIc | 1.8706 | TBI | TBI 21 |
| PADI3 | SSyDAKRAQVFHIc | 1.8706 | TBI | TBI 21 |
| PADI3 | SSyDAKRAQVFHIc | 1.8706 | TBI | TBI 21 |
| PADI3 | SSyDAKRAQVFHIc | 1.8706 | TBI | TBI 21 |
| ABCG5 | VESGQIMcILGSSGsGKT | 1.867 | TBI | TBI 21 |
| TRB | SATYLcAVQDLGTSGSRLT | 1.8589 | TBI | TBI 21 |
| TRB | SATYLcAVQDLGTSGSRLT | 1.8589 | TBI | TBI 21 |
| TRB | SATYLcAVQDLGTSGSRLT | 1.8589 | TBI | TBI 21 |
| TRB | SATYLcAVQDLGTSGSRLT | 1.8589 | TBI | TBI 21 |
| CCER1 | RYSPKtEYGPPRKQP | 1.8379 | TBI | TBI 21 |
| CCER1 | RYSPKtEYGPPRKQP | 1.8379 | TBI | TBI 21 |
| CCER1 | RYSPKtEYGPPRKQP | 1.8379 | TBI | TBI 21 |
| MT-ND5 | CLLVWFCFLCcVsLC | 1.8353 | TBI | TBI 21 |
| MT-ND5 | CLLVWFCFLCcVsLC | 1.8353 | TBI | TBI 21 |
| SBF1 | PKLLRPRLLPGEECVLD | 1.8308 | TBI | TBI 21 |
| SBF1 | PKLLRPRLLPGEECVLD | 1.8308 | TBI | TBI 21 |
| SBF1 | PKLLRPRLLPGEECVLD | 1.8308 | TBI | TBI 21 |
| SBF1 | PKLLRPRLLPGEECVLD | 1.8308 | TBI | TBI 21 |
| SBF1 | PKLLRPRLLPGEECVLD | 1.8308 | TBI | TBI 21 |
| SBF1 | PKLLRPRLLPGEECVLD | 1.8308 | TBI | TBI 21 |
| SBF1 | PKLLRPRLLPGEECVLD | 1.8308 | TBI | TBI 21 |
| SBF1 | PKLLRPRLLPGEECVLD | 1.8308 | TBI | TBI 21 |
| SBF1 | PKLLRPRLLPGEECVLD | 1.8308 | TBI | TBI 21 |
| AFF3 | QPNcRTSVPSSKGSsSS | 1.8282 | TBI | TBI 21 |
| AFF3 | QPNcRTSVPSSKGSsSS | 1.8282 | TBI | TBI 21 |
| AFF3 | QPNcRTSVPSSKGSsSS | 1.8282 | TBI | TBI 21 |
| AFF3 | QPNcRTSVPSSKGSsSS | 1.8282 | TBI | TBI 21 |
| AFF3 | QPNcRTSVPSSKGSsSS | 1.8282 | TBI | TBI 21 |
| AFF3 | QPNcRTSVPSSKGSsSS | 1.8282 | TBI | TBI 21 |
| AFF3 | QPNcRTSVPSSKGSsSS | 1.8282 | TBI | TBI 21 |
| AFF3 | QPNcRTSVPSSKGSsSS | 1.8282 | TBI | TBI 21 |
| AFF3 | QPNcRTSVPSSKGSsSS | 1.8282 | TBI | TBI 21 |
| AFF3 | QPNcRTSVPSSKGSsSS | 1.8282 | TBI | TBI 21 |
| AFF3 | QPNcRTSVPSSKGSsSS | 1.8282 | TBI | TBI 21 |
| AFF3 | QPNcRTSVPSSKGSsSS | 1.8282 | TBI | TBI 21 |
| AFF3 | QPNcRTSVPSSKGSsSS | 1.8282 | TBI | TBI 21 |
| AFF3 | QPNcRTSVPSSKGSsSS | 1.8282 | TBI | TBI 21 |
| AFF3 | QPNcRTSVPSSKGSsSS | 1.8282 | TBI | TBI 21 |
| AFF3 | QPNcRTSVPSSKGSsSS | 1.8282 | TBI | TBI 21 |
| AFF3 | QPNcRTSVPSSKGSsSS | 1.8282 | TBI | TBI 21 |
| AFF3 | QPNcRTSVPSSKGSsSS | 1.8282 | TBI | TBI 21 |
| AFF3 | QPNcRTSVPSSKGSsSS | 1.8282 | TBI | TBI 21 |
| FREM2 | tLGmNFTQDEVDRNL | 1.8275 | TBI | TBI 21 |
| FREM2 | tLGmNFTQDEVDRNL | 1.8275 | TBI | TBI 21 |
| FREM2 | tLGmNFTQDEVDRNL | 1.8275 | TBI | TBI 21 |
| FREM2 | tLGmNFTQDEVDRNL | 1.8275 | TBI | TBI 21 |
| FREM2 | tLGmNFTQDEVDRNL | 1.8275 | TBI | TBI 21 |
| MCRS1 | SLcPcccLAGETLVSQAPT | 1.825 | TBI | TBI 21 |
| ARID1B | NsSmQDMYNQSPSGAMS | 1.8094 | TBI | TBI 21 |
| ARID1B | NSsmQDMYNQSPSGAMS | 1.8094 | TBI | TBI 21 |
| ARID1B | NsSmQDMYNQSPSGAMS | 1.8094 | TBI | TBI 21 |
| ARID1B | NSsmQDMYNQSPSGAMS | 1.8094 | TBI | TBI 21 |
| ARID1B | NsSmQDMYNQSPSGAMS | 1.8094 | TBI | TBI 21 |
| ARID1B | NSsmQDMYNQSPSGAMS | 1.8094 | TBI | TBI 21 |
| ARID1B | NsSmQDMYNQSPSGAMS | 1.8094 | TBI | TBI 21 |
| ARID1B | NSsmQDMYNQSPSGAMS | 1.8094 | TBI | TBI 21 |
| ARID1B | NsSmQDMYNQSPSGAMS | 1.8094 | TBI | TBI 21 |
| ARID1B | NSsmQDMYNQSPSGAMS | 1.8094 | TBI | TBI 21 |
| ARID1B | NsSmQDMYNQSPSGAMS | 1.8094 | TBI | TBI 21 |
| ARID1B | NsSmQDMYNQSPSGAMS | 1.8094 | TBI | TBI 21 |
| ARID1B | NSsmQDMYNQSPSGAMS | 1.8094 | TBI | TBI 21 |
| MDFIC | CNCPcDMDCGIMDAccES | 1.8034 | TBI | TBI 21 |
| MDFIC | CNCPcDMDCGIMDAccES | 1.8034 | TBI | TBI 21 |
| MDFIC | CNCPcDMDCGIMDAccES | 1.8034 | TBI | TBI 21 |
| PSD3 | GTSsGTFSPVRLDESGE | 1.8006 | TBI | TBI 21 |
| PSD3 | GTSsGTFSPVRLDESGE | 1.8006 | TBI | TBI 21 |
| PSD3 | GTSsGTFSPVRLDESGE | 1.8006 | TBI | TBI 21 |
| PSD3 | GTSsGTFSPVRLDESGE | 1.8006 | TBI | TBI 21 |
| PSD3 | GTSsGTFSPVRLDESGE | 1.8006 | TBI | TBI 21 |
| PSD3 | GTSsGTFSPVRLDESGE | 1.8006 | TBI | TBI 21 |
| PSD3 | GTSsGTFSPVRLDESGE | 1.8006 | TBI | TBI 21 |
| PSD3 | GTSsGTFSPVRLDESGE | 1.8006 | TBI | TBI 21 |
| PSD3 | GTSsGTFSPVRLDESGE | 1.8006 | TBI | TBI 21 |
| PSD3 | GTSsGTFSPVRLDESGE | 1.8006 | TBI | TBI 21 |
| PSD3 | GTSsGTFSPVRLDESGE | 1.8006 | TBI | TBI 21 |
| ARID1B | NSsmQDMYNQSPSGAMS | 1.7994 | TBI | TBI 21 |
| LSM12 | VVITPPyQVENcKGKEG | 1.7867 | TBI | TBI 21 |
| LSM12 | VVITPPyQVENcKGKEG | 1.7867 | TBI | TBI 21 |
| LSM12 | VVITPPyQVENcKGKEG | 1.7867 | TBI | TBI 21 |
| FBN2 | cDMHASCLNIPGSFKCSC | 1.7722 | TBI | TBI 21 |
| FBN2 | cDMHASCLNIPGSFKCSC | 1.7722 | TBI | TBI 21 |
| FBN2 | cDMHASCLNIPGSFKCSC | 1.7722 | TBI | TBI 21 |
| FBN2 | cDMHASCLNIPGSFKCSC | 1.7722 | TBI | TBI 21 |
| FBN2 | cDMHASCLNIPGSFKCSC | 1.7722 | TBI | TBI 21 |
| FBN2 | cDMHASCLNIPGSFKCSC | 1.7722 | TBI | TBI 21 |
| FBN2 | cDMHASCLNIPGSFKCSC | 1.7722 | TBI | TBI 21 |
| FBN2 | cDMHASCLNIPGSFKCSC | 1.7722 | TBI | TBI 21 |
| FBN2 | cDMHASCLNIPGSFKCSC | 1.7722 | TBI | TBI 21 |
| FBN2 | cDMHASCLNIPGSFKCSC | 1.7722 | TBI | TBI 21 |
| FBN2 | cDMHASCLNIPGSFKCSC | 1.7722 | TBI | TBI 21 |
| FBN2 | cDMHASCLNIPGSFKCSC | 1.7722 | TBI | TBI 21 |
| FBN2 | cDMHASCLNIPGSFKCSC | 1.7722 | TBI | TBI 21 |
| FBN2 | cDMHASCLNIPGSFKCSC | 1.7722 | TBI | TBI 21 |
| CCDC148 | HmKNEMRNIKyKPV | 1.7611 | TBI | TBI 21 |
| CCDC148 | HmKNEMRNIKyKPV | 1.7611 | TBI | TBI 21 |
| CCDC148 | HmKNEMRNIKyKPV | 1.7611 | TBI | TBI 21 |
| CCDC148 | HmKNEMRNIKyKPV | 1.7611 | TBI | TBI 21 |
| CCDC148 | HmKNEMRNIKyKPV | 1.7611 | TBI | TBI 21 |
| CCDC148 | HmKNEMRNIKyKPV | 1.7611 | TBI | TBI 21 |
| CCDC148 | HmKNEMRNIKyKPV | 1.7611 | TBI | TBI 21 |
| CCDC148 | HmKNEMRNIKyKPV | 1.7611 | TBI | TBI 21 |
| CCDC148 | HmKNEMRNIKyKPV | 1.7611 | TBI | TBI 21 |
| CCDC148 | HmKNEMRNIKyKPV | 1.7611 | TBI | TBI 21 |
| CCDC148 | HmKNEMRNIKyKPV | 1.7611 | TBI | TBI 21 |
| MAPRE2 | SGStQcAGSVPTGAAYcQF | 1.7559 | TBI | TBI 21 |
| MAPRE2 | SGStQcAGSVPTGAAYcQF | 1.7559 | TBI | TBI 21 |
| MAPRE2 | SGStQcAGSVPTGAAYcQF | 1.7559 | TBI | TBI 21 |
| MAPRE2 | SGStQcAGSVPTGAAYcQF | 1.7559 | TBI | TBI 21 |
| MAPRE2 | SGStQcAGSVPTGAAYcQF | 1.7559 | TBI | TBI 21 |
| EBF4 | QsLHGPMFKNTSVGPLY | 1.7553 | TBI | TBI 21 |
| EBF4 | QsLHGPMFKNTSVGPLY | 1.7553 | TBI | TBI 21 |
| EBF4 | QsLHGPMFKNTSVGPLY | 1.7553 | TBI | TBI 21 |
| MAPRE2 | SGsTQcAGSVPTGAAYcQF | 1.7367 | TBI | TBI 21 |
| MAPRE2 | SGsTQcAGSVPTGAAYcQF | 1.7367 | TBI | TBI 21 |
| MAPRE2 | SGsTQcAGSVPTGAAYcQF | 1.7367 | TBI | TBI 21 |
| MAPRE2 | SGsTQcAGSVPTGAAYcQF | 1.7367 | TBI | TBI 21 |
| MAPRE2 | SGsTQcAGSVPTGAAYcQF | 1.7367 | TBI | TBI 21 |
| TENM1 | yVACDNTGtPLAVFSS | 1.7228 | TBI | TBI 21 |
| TENM1 | yVACDNTGtPLAVFSS | 1.7228 | TBI | TBI 21 |
| TENM1 | yVACDNTGtPLAVFSS | 1.7228 | TBI | TBI 21 |
| TENM1 | yVACDNTGtPLAVFSS | 1.7228 | TBI | TBI 21 |
| TENM1 | yVACDNTGtPLAVFSS | 1.7228 | TBI | TBI 21 |
| TENM1 | yVACDNTGtPLAVFSS | 1.7228 | TBI | TBI 21 |
| TENM1 | yVACDNTGtPLAVFSS | 1.7228 | TBI | TBI 21 |
| C7 | SLVCNGDSDcDEDs | 1.7174 | TBI | TBI 21 |
| C7 | SLVCNGDSDcDEDs | 1.7174 | TBI | TBI 21 |
| C7 | SLVCNGDSDcDEDs | 1.7174 | TBI | TBI 21 |
| C7 | SLVCNGDSDcDEDs | 1.7174 | TBI | TBI 21 |
| C7 | SLVCNGDSDcDEDs | 1.7174 | TBI | TBI 21 |
| TNFRSF14 | APPRPTPSPFLVtCLQ | 1.7171 | TBI | TBI 21 |
| TNFRSF14 | APPRPTPSPFLVtCLQ | 1.7171 | TBI | TBI 21 |
| TNFRSF14 | APPRPTPSPFLVtCLQ | 1.7171 | TBI | TBI 21 |
| ING2 | ScVSLTyKPKGKWYCP | 1.7029 | TBI | TBI 21 |
| ING2 | ScVSLTyKPKGKWYCP | 1.7029 | TBI | TBI 21 |
| ING2 | ScVSLTyKPKGKWYCP | 1.7029 | TBI | TBI 21 |
| ING2 | ScVSLTyKPKGKWYCP | 1.7029 | TBI | TBI 21 |
| ING2 | ScVSLTyKPKGKWYCP | 1.7029 | TBI | TBI 21 |
| ING2 | ScVSLTyKPKGKWYCP | 1.7029 | TBI | TBI 21 |
| ING2 | ScVSLTyKPKGKWYCP | 1.7029 | TBI | TBI 21 |
| KCNK18 | ARRccPEALGK | 2.4503 | TBI | TBI 22 |
| KCNK18 | VERLDIPLPIIALIVFA | 2.4503 | TBI | TBI 22 |
| WDR46 | EsDFLDNVSGSScGPVP | 2.2491 | TBI | TBI 22 |
| WDR46 | EsDFLDNVSGSScGPVP | 2.2491 | TBI | TBI 22 |
| WDR46 | EsDFLDNVSGSScGPVP | 2.2491 | TBI | TBI 22 |
| WDR46 | EsDFLDNVSGSScGPVP | 2.2491 | TBI | TBI 22 |
| MEGF10 | CGcKNDAVcSPVDGs | 2.1841 | TBI | TBI 22 |
| MEGF10 | CGcKNDAVcSPVDGs | 2.1841 | TBI | TBI 22 |
| MEGF10 | CGcKNDAVcSPVDGs | 2.1841 | TBI | TBI 22 |
| MEGF10 | cVHGRCIAPNtCQC | 2.1841 | TBI | TBI 22 |
| ZFHX3 | QSAAHsNDSPPPPSAAAPSS | 1.971 | TBI | TBI 22 |
| ZFHX3 | QSAAHsNDSPPPPSAAAPSS | 1.971 | TBI | TBI 22 |
| ZFHX3 | QSAAHsNDSPPPPSAAAPSS | 1.971 | TBI | TBI 22 |
| ZFHX3 | QSAAHsNDSPPPPSAAAPSS | 1.971 | TBI | TBI 22 |
| MT-ND5 | IVNVIVCLLVcLcMFmS | 1.9481 | TBI | TBI 22 |
| MT-ND5 | IVNVIVCLLVcLcMFmS | 1.9481 | TBI | TBI 22 |
| MT-ND5 | IVNVIVCLLVcLcMFmS | 1.9481 | TBI | TBI 22 |
| MT-ND5 | IVNVIVCLLVcLcMFmS | 1.9234 | TBI | TBI 22 |
| MT-ND5 | IVNVVVcLVIcLcMFmS | 1.8905 | TBI | TBI 22 |
| MT-ND5 | IVNVVVcLVIcLcMFmS | 1.8905 | TBI | TBI 22 |
| MT-ND5 | IVNVVVcLVIcLcMFmS | 1.8905 | TBI | TBI 22 |
| MT-ND5 | IVNVVVcLVIcLcMFmS | 1.8658 | TBI | TBI 22 |
| IGL | PGTSPKLWIySTSNLASG | 1.8472 | TBI | TBI 22 |
| IGL | PGTSPKLWIySTSNLASG | 1.8472 | TBI | TBI 22 |
| IGL | PGTSPKLWIySTSNLASG | 1.8472 | TBI | TBI 22 |
| IGL | PGTSPKLWIYsTSNLASG | 1.8472 | TBI | TBI 22 |
| IGL | PGTSPKLWIySTSNLASG | 1.8472 | TBI | TBI 22 |
| TNRC6B | HmcVLGNTtILAEFATD | 1.8333 | TBI | TBI 22 |
| TNRC6B | HmcVLGNTtILAEFATD | 1.8333 | TBI | TBI 22 |
| TNRC6B | HmcVLGNTtILAEFATD | 1.8333 | TBI | TBI 22 |
| TNRC6B | HmcVLGNTtILAEFATD | 1.8333 | TBI | TBI 22 |
| TNRC6B | HmcVLGNTtILAEFATD | 1.8333 | TBI | TBI 22 |
| TNRC6B | HmcVLGNTtILAEFATD | 1.8333 | TBI | TBI 22 |
| SH2D7 | DQEGSTyEQIPAcW | 1.8303 | TBI | TBI 22 |
| SH2D7 | DQEGSTyEQIPAcW | 1.8303 | TBI | TBI 22 |
| SH2D7 | DQEGSTyEQIPAcW | 1.8303 | TBI | TBI 22 |
| SH2D7 | DQEGStYEQIPAcW | 1.8303 | TBI | TBI 22 |
| SH2D7 | DQEGSTyEQIPAcW | 1.8303 | TBI | TBI 22 |
| SH2D7 | DQEGStYEQIPAcW | 1.8256 | TBI | TBI 22 |
| SH2D7 | DQEGStYEQIPAcW | 1.8256 | TBI | TBI 22 |
| SH2D7 | DQEGStYEQIPAcW | 1.8256 | TBI | TBI 22 |
| ZNF646 | DSVLEDIMNsVSGEGGD | 1.7816 | TBI | TBI 22 |
| ZNF646 | DSVLEDIMNsVSGEGGD | 1.7816 | TBI | TBI 22 |
| ZNF646 | DSVLEDIMNsVSGEGGD | 1.7816 | TBI | TBI 22 |
| SVEP1 | AAGsVVSFKcMEGFVLNT | 2.1534 | TBI+CM | TBI 24 |
| SVEP1 | AAGsVVSFKCmEGFVLNT | 2.1534 | TBI+CM | TBI 24 |
| SVEP1 | AAGsVVSFKcMEGFVLNT | 2.1534 | TBI+CM | TBI 24 |
| SVEP1 | AAGsVVSFKCmEGFVLNT | 2.1534 | TBI+CM | TBI 24 |
| SVEP1 | AAGsVVSFKcMEGFVLNT | 2.1534 | TBI+CM | TBI 24 |
| SVEP1 | AAGsVVSFKcMEGFVLNT | 2.1534 | TBI+CM | TBI 24 |
| SVEP1 | AAGsVVSFKCmEGFVLNT | 2.1534 | TBI+CM | TBI 24 |
| MEGF10 | CPPGYtGAFcEDLCPPGK | 2.0359 | TBI+CM | TBI 24 |
| MEGF10 | CPPGYtGAFcEDLCPPGK | 2.0359 | TBI+CM | TBI 24 |
| MEGF10 | CPPGYtGAFcEDLCPPGK | 2.0359 | TBI+CM | TBI 24 |
| MEGF10 | CPPGYtGAFcEDLCPPGK | 2.0359 | TBI+CM | TBI 24 |
| MEGF10 | CPPGyTGAFCEDLcPPGK | 2.0359 | TBI+CM | TBI 24 |
| MEGF10 | CPPGyTGAFCEDLcPPGK | 2.0359 | TBI+CM | TBI 24 |
| MEGF10 | CPPGyTGAFCEDLcPPGK | 2.0359 | TBI+CM | TBI 24 |
| MEGF10 | CPPGyTGAFcEDLCPPGK | 2.0359 | TBI+CM | TBI 24 |
| MEGF10 | CPPGyTGAFCEDLcPPGK | 2.0359 | TBI+CM | TBI 24 |
| MEGF10 | CPPGYtGAFCEDLcPPGK | 2.0359 | TBI+CM | TBI 24 |
| MEGF10 | CPPGyTGAFCEDLcPPGK | 2.0359 | TBI+CM | TBI 24 |
| MEGF10 | CPPGYtGAFcEDLCPPGK | 2.0359 | TBI+CM | TBI 24 |
| MEGF10 | CPPGyTGAFCEDLcPPGK | 2.0359 | TBI+CM | TBI 24 |
| MEGF10 | CPPGyTGAFCEDLcPPGK | 2.0359 | TBI+CM | TBI 24 |
| MEGF10 | CPPGyTGAFcEDLCPPGK | 2.0179 | TBI+CM | TBI 24 |
| MEGF10 | CPPGyTGAFcEDLCPPGK | 2.0179 | TBI+CM | TBI 24 |
| MEGF10 | CPPGyTGAFcEDLCPPGK | 2.0179 | TBI+CM | TBI 24 |
| MEGF10 | CPPGyTGAFcEDLCPPGK | 2.0179 | TBI+CM | TBI 24 |
| MEGF10 | CPPGyTGAFcEDLCPPGK | 2.0179 | TBI+CM | TBI 24 |
| MEGF10 | CPPGyTGAFcEDLCPPGK | 2.0179 | TBI+CM | TBI 24 |
| RXFP1 | RSENKLyAMSIISLcc | 2.0105 | TBI+CM | TBI 24 |
| RXFP1 | RSENKLyAMSIISLcc | 2.0105 | TBI+CM | TBI 24 |
| RXFP1 | RSENKLyAMSIISLcc | 2.0105 | TBI+CM | TBI 24 |
| RXFP1 | RSENKLyAMSIISLcc | 2.0105 | TBI+CM | TBI 24 |
| RXFP1 | RSENKLyAMSIISLcc | 2.0105 | TBI+CM | TBI 24 |
| RXFP1 | RSENKLyAMSIISLcc | 2.0105 | TBI+CM | TBI 24 |
| RXFP1 | RSENKLyAMSIISLcc | 2.0105 | TBI+CM | TBI 24 |
| RXFP1 | RSENKLyAMSIISLcc | 2.0105 | TBI+CM | TBI 24 |
| RXFP1 | RSENKLyAMSIISLcc | 2.0105 | TBI+CM | TBI 24 |
| RXFP1 | RSENKLyAMSIISLcc | 2.0105 | TBI+CM | TBI 24 |
| RXFP1 | RSENKLyAMSIISLcc | 2.0105 | TBI+CM | TBI 24 |
| SVEP1 | AAGsVVSFKCmEGFVLNT | 1.9865 | TBI+CM | TBI 24 |
| SMYD3 | AFAKVIcNSFTICNAEm | 1.9388 | TBI+CM | TBI 24 |
| SMYD3 | AFAKVIcNSFTICNAEm | 1.9388 | TBI+CM | TBI 24 |
| SMYD3 | AFAKVIcNSFTICNAEm | 1.9388 | TBI+CM | TBI 24 |
| SMYD3 | AFAKVIcNSFTICNAEm | 1.9388 | TBI+CM | TBI 24 |
| SMYD3 | AFAKVIcNSFTICNAEm | 1.9388 | TBI+CM | TBI 24 |
| SMYD3 | AFAKVIcNSFTICNAEm | 1.9388 | TBI+CM | TBI 24 |
| SMYD3 | AFAKVIcNSFTICNAEm | 1.9388 | TBI+CM | TBI 24 |
| SMYD3 | AFAKVIcNSFTICNAEm | 1.9388 | TBI+CM | TBI 24 |
| SMYD3 | AFAKVIcNSFTICNAEm | 1.9388 | TBI+CM | TBI 24 |
| POU4F3 | HFSmEFcsCcPGWSAM | 1.8912 | TBI+CM | TBI 24 |
| POU4F3 | HFSmEFCsccPGWSAM | 1.8912 | TBI+CM | TBI 24 |
| POU4F3 | HFSmEFcsCcPGWSAM | 1.8912 | TBI+CM | TBI 24 |
| POU4F3 | HFSmEFcsCcPGWSAM | 1.8912 | TBI+CM | TBI 24 |
| POU4F3 | HFSmEFCsccPGWSAM | 1.8912 | TBI+CM | TBI 24 |
| POU4F3 | HFSmEFcsCcPGWSAM | 1.8912 | TBI+CM | TBI 24 |
| POU4F3 | HFSmEFcsCcPGWSAM | 1.8912 | TBI+CM | TBI 24 |
| FBN2 | KRMCccTY | 1.8895 | TBI+CM | TBI 24 |
| FBN2 | KRMCccTY | 1.8895 | TBI+CM | TBI 24 |
| FBN2 | KRMCccTY | 1.8895 | TBI+CM | TBI 24 |
| FBN2 | KRMCccTY | 1.8895 | TBI+CM | TBI 24 |
| FBN2 | KRMCccTY | 1.8895 | TBI+CM | TBI 24 |
| FBN2 | KRMCccTY | 1.8895 | TBI+CM | TBI 24 |
| FBN2 | KRMCccTY | 1.8895 | TBI+CM | TBI 24 |
| FBN2 | KRMCccTY | 1.8895 | TBI+CM | TBI 24 |
| FBN2 | KRMCccTY | 1.8895 | TBI+CM | TBI 24 |
| FBN2 | cECPEGLTLDG | 1.8895 | TBI+CM | TBI 24 |
| FBN2 | KRMCccTY | 1.8895 | TBI+CM | TBI 24 |
| FBN2 | KRMCccTY | 1.8895 | TBI+CM | TBI 24 |
| MEGF10 | CPPGYtGAFCEDLcPPGK | 1.8748 | TBI+CM | TBI 24 |
| MEGF10 | CPPGYtGAFCEDLcPPGK | 1.8748 | TBI+CM | TBI 24 |
| MEGF10 | CPPGYtGAFCEDLcPPGK | 1.8748 | TBI+CM | TBI 24 |
| MEGF10 | CPPGYtGAFCEDLcPPGK | 1.8748 | TBI+CM | TBI 24 |
| MEGF10 | CPPGYtGAFCEDLcPPGK | 1.8748 | TBI+CM | TBI 24 |
| MEGF10 | CPPGYtGAFCEDLcPPGK | 1.8748 | TBI+CM | TBI 24 |
| MEGF10 | CPPGYtGAFcEDLCPPGK | 1.8571 | TBI+CM | TBI 24 |
| MEGF10 | CPPGYtGAFcEDLCPPGK | 1.8571 | TBI+CM | TBI 24 |
| MEGF10 | CPPGYtGAFcEDLCPPGK | 1.8571 | TBI+CM | TBI 24 |
| MEGF10 | CPPGYtGAFcEDLCPPGK | 1.8571 | TBI+CM | TBI 24 |
| MEGF10 | CPPGYtGAFcEDLCPPGK | 1.8571 | TBI+CM | TBI 24 |
| MEGF10 | CPPGYtGAFcEDLCPPGK | 1.8571 | TBI+CM | TBI 24 |
| PLOD1 | KFLLEYIAPmTEKLy | 1.8254 | TBI+CM | TBI 24 |
| PLOD1 | KFLLEYIAPmTEKLy | 1.8254 | TBI+CM | TBI 24 |
| PLOD1 | KFLLEYIAPmTEKLy | 1.8254 | TBI+CM | TBI 24 |
| PLOD1 | KFLLEYIAPmTEKLy | 1.8254 | TBI+CM | TBI 24 |
| PLOD1 | KFLLEYIAPmTEKLy | 1.8254 | TBI+CM | TBI 24 |
| PLOD1 | KFLLEYIAPmTEKLy | 1.8254 | TBI+CM | TBI 24 |
| PLOD1 | KFLLEYIAPmTEKLy | 1.8254 | TBI+CM | TBI 24 |
| PLOD1 | KFLLEYIAPmTEKLy | 1.8254 | TBI+CM | TBI 24 |
| PLOD1 | KFLLEYIAPmTEKLy | 1.8254 | TBI+CM | TBI 24 |
| PLOD1 | KFLLEYIAPmTEKLy | 1.8254 | TBI+CM | TBI 24 |
| PLOD1 | KFLLEYIAPmTEKLy | 1.8254 | TBI+CM | TBI 24 |
| MUC5B | VVTTmAtMPTATASTVPS | 1.7964 | TBI+CM | TBI 24 |
| MUC5B | VVTTmAtMPTATASTVPS | 1.7964 | TBI+CM | TBI 24 |
| MUC5B | VVTTmAtMPTATASTVPS | 1.7964 | TBI+CM | TBI 24 |
| MUC5B | VVTTmAtMPTATASTVPS | 1.7964 | TBI+CM | TBI 24 |
| MUC5B | VVTTmAtMPTATASTVPS | 1.7964 | TBI+CM | TBI 24 |
| CTSW | SVQELLDcGRCGDGC | 1.7716 | TBI+CM | TBI 24 |
| CTSW | SVQELLDcGRCGDGC | 1.7716 | TBI+CM | TBI 24 |
| CTSW | SVQELLDcGRCGDGC | 1.7716 | TBI+CM | TBI 24 |
| CTSW | SVQELLDcGRCGDGC | 1.7716 | TBI+CM | TBI 24 |
| CTSW | SVQELLDcGRCGDGC | 1.7716 | TBI+CM | TBI 24 |
| CTSW | SVQELLDcGRCGDGC | 1.7716 | TBI+CM | TBI 24 |
| CTSW | SVQELLDcGRCGDGC | 1.7716 | TBI+CM | TBI 24 |
| POU4F3 | HFSmEFCsccPGWSAM | 1.7714 | TBI+CM | TBI 24 |
| POU4F3 | HFSmEFCsccPGWSAM | 1.7714 | TBI+CM | TBI 24 |
| POU4F3 | HFSmEFCsccPGWSAM | 1.7714 | TBI+CM | TBI 24 |
| TNRC6B | GmcAGGYSHISHcR | 1.7149 | TBI+CM | TBI 24 |
| TNRC6B | GmcAGGYSHISHcR | 1.7149 | TBI+CM | TBI 24 |
| TNRC6B | GmcAGGYSHISHcR | 1.7149 | TBI+CM | TBI 24 |
| RP1 | CLQPDGSctGVG | 2.8581 | TBI | TBI 25 |
| RP1 | IKDGRcDGTGtGDVDC | 2.8581 | TBI | TBI 25 |
| RP1 | CLQPDGSctGVG | 2.8581 | TBI | TBI 25 |
| RP1 | IKDGRcDGTGtGDVDC | 2.8581 | TBI | TBI 25 |
| RP1 | CLQPDGSctGVG | 2.8581 | TBI | TBI 25 |
| RP1 | IKDGRcDGTGtGDVDC | 2.8581 | TBI | TBI 25 |
| RP1 | CLQPDGSctGVG | 2.8581 | TBI | TBI 25 |
| RP1 | IKDGRcDGTGtGDVDC | 2.8581 | TBI | TBI 25 |
| RP1 | CLQPDGSctGVG | 2.8581 | TBI | TBI 25 |
| RP1 | IKDGRcDGTGtGDVDC | 2.8581 | TBI | TBI 25 |
| RP1 | CLQPDGSctGVG | 2.8581 | TBI | TBI 25 |
| RP1 | IKDGRcDGTGtGDVDC | 2.8581 | TBI | TBI 25 |
| CACHD1 | RtVTLTARVRCFILEV | 2.3201 | TBI | TBI 25 |
| CACHD1 | RtVTLTARVRCFILEV | 2.3201 | TBI | TBI 25 |
| CACHD1 | RtVTLTARVRCFILEV | 2.3201 | TBI | TBI 25 |
| TRB | SATYLcAVQDLGTSGSRLT | 2.0498 | TBI | TBI 25 |
| TRB | SATYLcAVQDLGTSGSRLT | 2.0498 | TBI | TBI 25 |
| TRB | SATYLcAVQDLGTSGSRLT | 2.0498 | TBI | TBI 25 |
| TRB | SATYLcAVQDLGTSGSRLT | 2.0498 | TBI | TBI 25 |
| CCDC148 | cLcDLTNFEQELsEQ | 2.0244 | TBI | TBI 25 |
| CCDC148 | cLcDLTNFEQELsEQ | 2.0244 | TBI | TBI 25 |
| CCDC148 | cLcDLTNFEQELsEQ | 2.0244 | TBI | TBI 25 |
| CCDC148 | cLcDLTNFEQELsEQ | 2.0244 | TBI | TBI 25 |
| CCDC148 | cLcDLTNFEQELsEQ | 2.0244 | TBI | TBI 25 |
| CCDC148 | cLcDLTNFEQELsEQ | 2.0244 | TBI | TBI 25 |
| CCDC148 | cLcDLTNFEQELsEQ | 2.0244 | TBI | TBI 25 |
| TRB | ALYLcASSLSPLAGGFNEQ | 1.9981 | TBI | TBI 25 |
| TRB | ALYLcASSLSPLAGGFNEQ | 1.9981 | TBI | TBI 25 |
| LRP1 | CSsGRcVAEALLCNGQ | 1.9969 | TBI | TBI 25 |
| LRP1 | CsSGRcVAEALLCNGQ | 1.9969 | TBI | TBI 25 |
| LRP1 | CSsGRcVAEALLCNGQ | 1.9969 | TBI | TBI 25 |
| LRP1 | CsSGRcVAEALLCNGQ | 1.9969 | TBI | TBI 25 |
| LRP1 | CSsGRcVAEALLCNGQ | 1.9969 | TBI | TBI 25 |
| LRP1 | CSsGRcVAEALLCNGQ | 1.9969 | TBI | TBI 25 |
| LRP1 | CsSGRcVAEALLCNGQ | 1.9969 | TBI | TBI 25 |
| LRP1 | CsSGRcVAEALLCNGQ | 1.9918 | TBI | TBI 25 |
| IGFN1 | QGCEccmScAVQGSPRPH | 1.9893 | TBI | TBI 25 |
| IGFN1 | QGCEccmScAVQGSPRPH | 1.9893 | TBI | TBI 25 |
| IGFN1 | QGCEccmScAVQGSPRPH | 1.9893 | TBI | TBI 25 |
| IGFN1 | QGCEccmScAVQGSPRPH | 1.9893 | TBI | TBI 25 |
| IGH | KIScKGSGFSFTNY | 1.8999 | TBI | TBI 25 |
| IGH | KIScKGSGFSFTNY | 1.8999 | TBI | TBI 25 |
| IGH | KIScKGSGFSFTNY | 1.8999 | TBI | TBI 25 |
| IGH | KIScKGSGFSFTNY | 1.8999 | TBI | TBI 25 |
| IGH | KIScKGSGFSFTNY | 1.8999 | TBI | TBI 25 |
| IGH | KIScKGSGFSFTNY | 1.8999 | TBI | TBI 25 |
| IGH | KIScKGSGFSFTNY | 1.8999 | TBI | TBI 25 |
| IGH | KIScKGSGFSFTNY | 1.8999 | TBI | TBI 25 |
| IGH | KIScKGSGFSFTNY | 1.8999 | TBI | TBI 25 |
| IGH | KIScKGSGFSFTNY | 1.8999 | TBI | TBI 25 |
| RP1 | IKDGRcDGTGtGDVDC | 1.8917 | TBI | TBI 25 |
| RP1 | IKDGRcDGTGtGDVDC | 1.8917 | TBI | TBI 25 |
| RP1 | IKDGRcDGTGtGDVDC | 1.8917 | TBI | TBI 25 |
| RP1 | IKDGRcDGTGtGDVDC | 1.8917 | TBI | TBI 25 |
| RP1 | IKDGRcDGTGtGDVDC | 1.8917 | TBI | TBI 25 |
| RP1 | IKDGRcDGTGtGDVDC | 1.8917 | TBI | TBI 25 |
| RP1 | IKDGRcDGTGtGDVDC | 1.8917 | TBI | TBI 25 |
| NOTCH4 | EIDPCHsQPcFHGGT | 1.8633 | TBI | TBI 25 |
| NOTCH4 | EIDPCHsQPcFHGGT | 1.8633 | TBI | TBI 25 |
| NOTCH4 | EIDPCHsQPcFHGGT | 1.8633 | TBI | TBI 25 |
| NOTCH4 | EIDPCHsQPcFHGGT | 1.8633 | TBI | TBI 25 |
| NOTCH4 | EIDPCHsQPcFHGGT | 1.8633 | TBI | TBI 25 |
| SYNE2 | CTPGLEDEKEASENEtD | 1.8455 | TBI | TBI 25 |
| SYNE2 | CTPGLEDEKEASENEtD | 1.8455 | TBI | TBI 25 |
| SYNE2 | CTPGLEDEKEASENEtD | 1.8455 | TBI | TBI 25 |
| SYNE2 | CTPGLEDEKEASENEtD | 1.8455 | TBI | TBI 25 |
| SYNE2 | CTPGLEDEKEASENEtD | 1.8455 | TBI | TBI 25 |
| SYNE2 | CTPGLEDEKEASENEtD | 1.8455 | TBI | TBI 25 |
| SYNE2 | CTPGLEDEKEASENEtD | 1.8455 | TBI | TBI 25 |
| SYNE2 | CTPGLEDEKEASENEtD | 1.8455 | TBI | TBI 25 |
| SYNE2 | CTPGLEDEKEASENEtD | 1.8455 | TBI | TBI 25 |
| SYNE2 | CTPGLEDEKEASENEtD | 1.8455 | TBI | TBI 25 |
| SYNE2 | CTPGLEDEKEASENEtD | 1.8455 | TBI | TBI 25 |
| SYNE2 | CTPGLEDEKEASENEtD | 1.8455 | TBI | TBI 25 |
| TF | FFSGSCAPCADGtDFPQL | 1.8422 | TBI | TBI 25 |
| TF | FFSGSCAPCADGtDFPQL | 1.8422 | TBI | TBI 25 |
| TF | FFSGSCAPCADGtDFPQL | 1.8422 | TBI | TBI 25 |
| TF | FFSGSCAPCADGtDFPQL | 1.8422 | TBI | TBI 25 |
| TF | FFSGSCAPCADGtDFPQL | 1.8422 | TBI | TBI 25 |
| TF | FFSGSCAPCADGtDFPQL | 1.8422 | TBI | TBI 25 |
| TF | FFSGSCAPCADGtDFPQL | 1.8422 | TBI | TBI 25 |
| TF | FFSGSCAPCADGtDFPQL | 1.8422 | TBI | TBI 25 |
| TF | FFSGSCAPCADGtDFPQL | 1.8422 | TBI | TBI 25 |
| TF | FFSGSCAPCADGtDFPQL | 1.8422 | TBI | TBI 25 |
| TF | FFSGSCAPCADGtDFPQL | 1.8422 | TBI | TBI 25 |
| TF | FFSGSCAPCADGtDFPQL | 1.8422 | TBI | TBI 25 |
| TF | FFSGSCAPCADGtDFPQL | 1.8422 | TBI | TBI 25 |
| TF | FFSGSCAPCADGtDFPQL | 1.8422 | TBI | TBI 25 |
| TF | FFSGSCAPCADGtDFPQL | 1.8422 | TBI | TBI 25 |
| TF | FFSGSCAPCADGtDFPQL | 1.8422 | TBI | TBI 25 |
| TF | FFSGSCAPCADGtDFPQL | 1.8422 | TBI | TBI 25 |
| TF | FFSGSCAPCADGtDFPQL | 1.8422 | TBI | TBI 25 |
| TF | FFSGSCAPCADGtDFPQL | 1.8422 | TBI | TBI 25 |
| TF | FFSGSCAPCADGtDFPQL | 1.8422 | TBI | TBI 25 |
| TF | FFSGSCAPCADGtDFPQL | 1.8422 | TBI | TBI 25 |
| TF | FFSGSCAPCADGtDFPQL | 1.8422 | TBI | TBI 25 |
| TF | FFSGSCAPCADGtDFPQL | 1.8422 | TBI | TBI 25 |
| TF | FFSGSCAPCADGtDFPQL | 1.8422 | TBI | TBI 25 |
| TF | FFSGSCAPCADGtDFPQL | 1.8422 | TBI | TBI 25 |
| TF | FFSGSCAPCADGtDFPQL | 1.8422 | TBI | TBI 25 |
| TF | FFSGSCAPCADGtDFPQL | 1.8422 | TBI | TBI 25 |
| TF | FFSGSCAPCADGtDFPQL | 1.8422 | TBI | TBI 25 |
| TF | FFSGSCAPCADGtDFPQL | 1.8422 | TBI | TBI 25 |
| TF | FFSGSCAPCADGtDFPQL | 1.8422 | TBI | TBI 25 |
| TF | FFSGSCAPCADGtDFPQL | 1.8422 | TBI | TBI 25 |
| TF | FFSGSCAPCADGtDFPQL | 1.8422 | TBI | TBI 25 |
| TF | FFSGSCAPCADGtDFPQL | 1.8422 | TBI | TBI 25 |
| TF | FFSGSCAPCADGtDFPQL | 1.8422 | TBI | TBI 25 |
| TF | FFSGSCAPCADGtDFPQL | 1.8422 | TBI | TBI 25 |
| TF | FFSGSCAPCADGtDFPQL | 1.8422 | TBI | TBI 25 |
| TF | FFSGSCAPCADGtDFPQL | 1.8422 | TBI | TBI 25 |
| TF | FFSGSCAPCADGtDFPQL | 1.8422 | TBI | TBI 25 |
| TF | FFSGSCAPCADGtDFPQL | 1.8422 | TBI | TBI 25 |
| TF | FFSGSCAPCADGtDFPQL | 1.8422 | TBI | TBI 25 |
| TF | FFSGSCAPCADGtDFPQL | 1.8422 | TBI | TBI 25 |
| TF | FFSGSCAPCADGtDFPQL | 1.8422 | TBI | TBI 25 |
| TF | FFSGSCAPCADGtDFPQL | 1.8422 | TBI | TBI 25 |
| TF | FFSGSCAPCADGtDFPQL | 1.8422 | TBI | TBI 25 |
| TF | FFSGSCAPCADGtDFPQL | 1.8422 | TBI | TBI 25 |
| TF | FFSGSCAPCADGtDFPQL | 1.8422 | TBI | TBI 25 |
| TF | FFSGSCAPCADGtDFPQL | 1.8422 | TBI | TBI 25 |
| TF | FFSGSCAPCADGtDFPQL | 1.8422 | TBI | TBI 25 |
| TF | FFSGSCAPCADGtDFPQL | 1.8422 | TBI | TBI 25 |
| TF | FFSGSCAPCADGtDFPQL | 1.8422 | TBI | TBI 25 |
| TF | FFSGSCAPCADGtDFPQL | 1.8422 | TBI | TBI 25 |
| TF | FFSGSCAPCADGtDFPQL | 1.8422 | TBI | TBI 25 |
| TF | FFSGSCAPCADGtDFPQL | 1.8422 | TBI | TBI 25 |
| TF | FFSGSCAPCADGtDFPQL | 1.8422 | TBI | TBI 25 |
| TF | FFSGSCAPCADGtDFPQL | 1.8422 | TBI | TBI 25 |
| TF | FFSGSCAPCADGtDFPQL | 1.8422 | TBI | TBI 25 |
| TF | FFSGSCAPCADGtDFPQL | 1.8422 | TBI | TBI 25 |
| TF | FFSGSCAPCADGtDFPQL | 1.8422 | TBI | TBI 25 |
| TF | FFSGSCAPCADGtDFPQL | 1.8422 | TBI | TBI 25 |
| TF | FFSGSCAPCADGtDFPQL | 1.8422 | TBI | TBI 25 |
| TF | FFSGSCAPCADGtDFPQL | 1.8422 | TBI | TBI 25 |
| TF | FFSGSCAPCADGtDFPQL | 1.8422 | TBI | TBI 25 |
| TF | FFSGSCAPCADGtDFPQL | 1.8422 | TBI | TBI 25 |
| TF | FFSGSCAPCADGtDFPQL | 1.8422 | TBI | TBI 25 |
| TF | FFSGSCAPCADGtDFPQL | 1.8422 | TBI | TBI 25 |
| ZBED4 | PIKLVQKVAsKIPSPDR | 1.8203 | TBI | TBI 25 |
| ZBED4 | PIKLVQKVAsKIPSPDR | 1.8203 | TBI | TBI 25 |
| ZBED4 | PIKLVQKVAsKIPSPDR | 1.8203 | TBI | TBI 25 |
| ZBED4 | PIKLVQKVAsKIPSPDR | 1.8203 | TBI | TBI 25 |
| ZBED4 | PIKLVQKVAsKIPSPDR | 1.8203 | TBI | TBI 25 |
| ZBED4 | PIKLVQKVAsKIPSPDR | 1.8203 | TBI | TBI 25 |
| ZBED4 | PIKLVQKVAsKIPSPDR | 1.8203 | TBI | TBI 25 |
| TANC1 | tYTcLVPEFVHSIAA | 1.8096 | TBI | TBI 25 |
| TANC1 | tYTcLVPEFVHSIAA | 1.8096 | TBI | TBI 25 |
| TANC1 | tYTcLVPEFVHSIAA | 1.8096 | TBI | TBI 25 |
| TANC1 | tYTcLVPEFVHSIAA | 1.8096 | TBI | TBI 25 |
| TANC1 | tYTcLVPEFVHSIAA | 1.8096 | TBI | TBI 25 |
| TANC1 | tYTcLVPEFVHSIAA | 1.8096 | TBI | TBI 25 |
| TANC1 | tYTcLVPEFVHSIAA | 1.8096 | TBI | TBI 25 |
| TANC1 | tYTcLVPEFVHSIAA | 1.8096 | TBI | TBI 25 |
| TANC1 | tYTcLVPEFVHSIAA | 1.8096 | TBI | TBI 25 |
| IGH | KIScKGSGFSFTNY | 1.7576 | TBI | TBI 25 |
| IGH | KIScKGSGFSFTNY | 1.7576 | TBI | TBI 25 |
| IGH | KIScKGSGFSFTNY | 1.7576 | TBI | TBI 25 |
| IGH | KIScKGSGFSFTNY | 1.7576 | TBI | TBI 25 |
| ZNF571 | YEcKDcGKAFI | 2.5415 | TBI+CM | TBI 28 |
| ZNF571 | YEcKDcGKAFI | 2.5415 | TBI+CM | TBI 28 |
| ZNF571 | YEcKDcGKAFI | 2.5415 | TBI+CM | TBI 28 |
| ZNF571 | YEcKDcGKAFI | 2.5415 | TBI+CM | TBI 28 |
| ZNF571 | YEcKDcGKAFI | 2.5415 | TBI+CM | TBI 28 |
| ZNF571 | YEcKDcGKAFI | 2.5415 | TBI+CM | TBI 28 |
| ZNF571 | PYEcKDCGKAFIL | 2.5415 | TBI+CM | TBI 28 |
| ZNF571 | YEcKDcGKAFI | 2.5415 | TBI+CM | TBI 28 |
| PXN | CGGCARAILENyISALNT | 2.0464 | TBI+CM | TBI 28 |
| PXN | CGGCARAILENyISALNT | 2.0464 | TBI+CM | TBI 28 |
| PXN | CGGCARAILENyISALNT | 2.0464 | TBI+CM | TBI 28 |
| PXN | CGGCARAILENyISALNT | 2.0464 | TBI+CM | TBI 28 |
| PXN | CGGCARAILENyISALNT | 2.0464 | TBI+CM | TBI 28 |
| PXN | CGGCARAILENyISALNT | 2.0464 | TBI+CM | TBI 28 |
| PXN | CGGCARAILENyISALNT | 2.0464 | TBI+CM | TBI 28 |
| PXN | CGGCARAILENyISALNT | 2.0464 | TBI+CM | TBI 28 |
| PXN | CGGCARAILENyISALNT | 2.0464 | TBI+CM | TBI 28 |
| RNF219 | DccsTSYAQNLDFESS | 1.9988 | TBI+CM | TBI 28 |
| RNF219 | DccsTSYAQNLDFESS | 1.9988 | TBI+CM | TBI 28 |
| RNF219 | DccsTSYAQNLDFESS | 1.9988 | TBI+CM | TBI 28 |
| RNF219 | DccsTSYAQNLDFESS | 1.9988 | TBI+CM | TBI 28 |
| RNF219 | DccsTSYAQNLDFESS | 1.9988 | TBI+CM | TBI 28 |
| RNF219 | DccsTSYAQNLDFESS | 1.9988 | TBI+CM | TBI 28 |
| RNF219 | DccsTSYAQNLDFESS | 1.9988 | TBI+CM | TBI 28 |
| RNF219 | DccsTSYAQNLDFESS | 1.9894 | TBI+CM | TBI 28 |
| VWDE | PKCLYGGRCIFPNVcsC | 1.9311 | TBI+CM | TBI 28 |
| VWDE | PKCLYGGRCIFPNVcsC | 1.9311 | TBI+CM | TBI 28 |
| VWDE | PKCLYGGRCIFPNVcsC | 1.9311 | TBI+CM | TBI 28 |
| VWDE | PKCLYGGRCIFPNVcsC | 1.9311 | TBI+CM | TBI 28 |
| LRP1 | NGGTcAASPSGMPtCRc | 1.9193 | TBI+CM | TBI 28 |
| LRP1 | NGGTcAASPSGMPtCRc | 1.9193 | TBI+CM | TBI 28 |
| LRP1 | NGGTcAASPSGMPtCRc | 1.9193 | TBI+CM | TBI 28 |
| LRP1 | NGGTcAASPSGMPtCRc | 1.9193 | TBI+CM | TBI 28 |
| MUC17 | TmLLSSTYVtSSEASTPS | 1.8819 | TBI+CM | TBI 28 |
| MUC17 | TmLLSSTYVtSSEASTPS | 1.8819 | TBI+CM | TBI 28 |
| MUC17 | TmLLSSTYVtSSEASTPS | 1.8819 | TBI+CM | TBI 28 |
| MUC17 | TmLLSSTYVtSSEASTPS | 1.8819 | TBI+CM | TBI 28 |
| MUC17 | TmLLSSTYVtSSEASTPS | 1.8819 | TBI+CM | TBI 28 |
| MUC17 | TmLLSSTYVtSSEASTPS | 1.8819 | TBI+CM | TBI 28 |
| MUC17 | TmLLSSTYVtSSEASTPS | 1.8819 | TBI+CM | TBI 28 |
| EBF4 | TStAQTTSLVSLPSGDQGI | 1.8762 | TBI+CM | TBI 28 |
| EBF4 | tSTAQTTSLVSLPSGDQGI | 1.8762 | TBI+CM | TBI 28 |
| EBF4 | TsTAQTTSLVSLPSGDQGI | 1.8762 | TBI+CM | TBI 28 |
| EBF4 | TsTAQTTSLVSLPSGDQGI | 1.8762 | TBI+CM | TBI 28 |
| EBF4 | TsTAQTTSLVSLPSGDQGI | 1.8762 | TBI+CM | TBI 28 |
| EBF4 | TsTAQTTSLVSLPSGDQGI | 1.8762 | TBI+CM | TBI 28 |
| EBF4 | TsTAQTTSLVSLPSGDQGI | 1.8762 | TBI+CM | TBI 28 |
| EBF4 | TsTAQTTSLVSLPSGDQGI | 1.8762 | TBI+CM | TBI 28 |
| EBF4 | TsTAQTTSLVSLPSGDQGI | 1.8762 | TBI+CM | TBI 28 |
| EBF4 | TsTAQTTSLVSLPSGDQGI | 1.8762 | TBI+CM | TBI 28 |
| EBF4 | TsTAQTTSLVSLPSGDQGI | 1.8762 | TBI+CM | TBI 28 |
| EBF4 | TsTAQTTSLVSLPSGDQGI | 1.8762 | TBI+CM | TBI 28 |
| EBF4 | TsTAQTTSLVSLPSGDQGI | 1.8762 | TBI+CM | TBI 28 |
| EBF4 | TsTAQTTSLVSLPSGDQGI | 1.8762 | TBI+CM | TBI 28 |
| EBF4 | TsTAQTTSLVSLPSGDQGI | 1.8762 | TBI+CM | TBI 28 |
| EBF4 | TStAQTTSLVSLPSGDQGI | 1.8762 | TBI+CM | TBI 28 |
| EBF4 | TStAQTTSLVSLPSGDQGI | 1.8762 | TBI+CM | TBI 28 |
| EBF4 | TsTAQTTSLVSLPSGDQGI | 1.8762 | TBI+CM | TBI 28 |
| EBF4 | TStAQTTSLVSLPSGDQGI | 1.8762 | TBI+CM | TBI 28 |
| EBF4 | TStAQTTSLVSLPSGDQGI | 1.8762 | TBI+CM | TBI 28 |
| EBF4 | TStAQTTSLVSLPSGDQGI | 1.8762 | TBI+CM | TBI 28 |
| EBF4 | TStAQTTSLVSLPSGDQGI | 1.8762 | TBI+CM | TBI 28 |
| EBF4 | TStAQTTSLVSLPSGDQGI | 1.8762 | TBI+CM | TBI 28 |
| EBF4 | TStAQTTSLVSLPSGDQGI | 1.8762 | TBI+CM | TBI 28 |
| EBF4 | TsTAQTTSLVSLPSGDQGI | 1.8762 | TBI+CM | TBI 28 |
| EBF4 | TStAQTTSLVSLPSGDQGI | 1.8762 | TBI+CM | TBI 28 |
| EBF4 | TStAQTTSLVSLPSGDQGI | 1.8762 | TBI+CM | TBI 28 |
| EBF4 | TStAQTTSLVSLPSGDQGI | 1.8762 | TBI+CM | TBI 28 |
| EBF4 | TStAQTTSLVSLPSGDQGI | 1.8762 | TBI+CM | TBI 28 |
| EBF4 | TStAQTTSLVSLPSGDQGI | 1.8762 | TBI+CM | TBI 28 |
| EBF4 | TStAQTTSLVSLPSGDQGI | 1.8762 | TBI+CM | TBI 28 |
| EBF4 | TsTAQTTSLVSLPSGDQGI | 1.8762 | TBI+CM | TBI 28 |
| EBF4 | TStAQTTSLVSLPSGDQGI | 1.8762 | TBI+CM | TBI 28 |
| EBF4 | TStAQTTSLVSLPSGDQGI | 1.8762 | TBI+CM | TBI 28 |
| EBF4 | TStAQTTSLVSLPSGDQGI | 1.8762 | TBI+CM | TBI 28 |
| EBF4 | TStAQTTSLVSLPSGDQGI | 1.8762 | TBI+CM | TBI 28 |
| EBF4 | TStAQTTSLVSLPSGDQGI | 1.8762 | TBI+CM | TBI 28 |
| EBF4 | tSTAQTTSLVSLPSGDQGI | 1.8659 | TBI+CM | TBI 28 |
| EBF4 | tSTAQTTSLVSLPSGDQGI | 1.8659 | TBI+CM | TBI 28 |
| EBF4 | tSTAQTTSLVSLPSGDQGI | 1.8659 | TBI+CM | TBI 28 |
| EBF4 | tSTAQTTSLVSLPSGDQGI | 1.8659 | TBI+CM | TBI 28 |
| EBF4 | tSTAQTTSLVSLPSGDQGI | 1.8659 | TBI+CM | TBI 28 |
| EBF4 | tSTAQTTSLVSLPSGDQGI | 1.8659 | TBI+CM | TBI 28 |
| EBF4 | tSTAQTTSLVSLPSGDQGI | 1.8659 | TBI+CM | TBI 28 |
| EBF4 | tSTAQTTSLVSLPSGDQGI | 1.8659 | TBI+CM | TBI 28 |
| EBF4 | tSTAQTTSLVSLPSGDQGI | 1.8659 | TBI+CM | TBI 28 |
| EBF4 | tSTAQTTSLVSLPSGDQGI | 1.8659 | TBI+CM | TBI 28 |
| EBF4 | tSTAQTTSLVSLPSGDQGI | 1.8659 | TBI+CM | TBI 28 |
| EBF4 | tSTAQTTSLVSLPSGDQGI | 1.8659 | TBI+CM | TBI 28 |
| EBF4 | tSTAQTTSLVSLPSGDQGI | 1.8659 | TBI+CM | TBI 28 |
| EBF4 | tSTAQTTSLVSLPSGDQGI | 1.8659 | TBI+CM | TBI 28 |
| EBF4 | tSTAQTTSLVSLPSGDQGI | 1.8659 | TBI+CM | TBI 28 |
| EBF4 | tSTAQTTSLVSLPSGDQGI | 1.8659 | TBI+CM | TBI 28 |
| EBF4 | tSTAQTTSLVSLPSGDQGI | 1.8659 | TBI+CM | TBI 28 |
| EBF4 | tSTAQTTSLVSLPSGDQGI | 1.8659 | TBI+CM | TBI 28 |
| EBF4 | tSTAQTTSLVSLPSGDQGI | 1.8659 | TBI+CM | TBI 28 |
| MTO1 | CQWGCFGLECsGmITAR | 1.8596 | TBI+CM | TBI 28 |
| MTO1 | CQWGCFGLECsGmITAR | 1.8596 | TBI+CM | TBI 28 |
| MTO1 | CQWGCFGLECsGmITAR | 1.8596 | TBI+CM | TBI 28 |
| EBF4 | TsTAQTTSLVSLPSGDQGI | 1.8155 | TBI+CM | TBI 28 |
| EBF4 | TsTAQTTSLVSLPSGDQGI | 1.8155 | TBI+CM | TBI 28 |
| EBF4 | TsTAQTTSLVSLPSGDQGI | 1.8155 | TBI+CM | TBI 28 |
| EBF4 | TsTAQTTSLVSLPSGDQGI | 1.8155 | TBI+CM | TBI 28 |
| EBF4 | TsTAQTTSLVSLPSGDQGI | 1.8155 | TBI+CM | TBI 28 |
| EBF4 | TsTAQTTSLVSLPSGDQGI | 1.8155 | TBI+CM | TBI 28 |
| EBF4 | TsTAQTTSLVSLPSGDQGI | 1.8155 | TBI+CM | TBI 28 |
| EBF4 | TsTAQTTSLVSLPSGDQGI | 1.8155 | TBI+CM | TBI 28 |
| EBF4 | TsTAQTTSLVSLPSGDQGI | 1.8155 | TBI+CM | TBI 28 |
| EBF4 | TsTAQTTSLVSLPSGDQGI | 1.8155 | TBI+CM | TBI 28 |
| EBF4 | TsTAQTTSLVSLPSGDQGI | 1.8155 | TBI+CM | TBI 28 |
| EBF4 | TsTAQTTSLVSLPSGDQGI | 1.8155 | TBI+CM | TBI 28 |
| EBF4 | TsTAQTTSLVSLPSGDQGI | 1.8155 | TBI+CM | TBI 28 |
| EBF4 | TsTAQTTSLVSLPSGDQGI | 1.8155 | TBI+CM | TBI 28 |
| EBF4 | TsTAQTTSLVSLPSGDQGI | 1.8155 | TBI+CM | TBI 28 |
| EBF4 | TsTAQTTSLVSLPSGDQGI | 1.8155 | TBI+CM | TBI 28 |
| EBF4 | TsTAQTTSLVSLPSGDQGI | 1.8155 | TBI+CM | TBI 28 |
| EBF4 | TsTAQTTSLVSLPSGDQGI | 1.8155 | TBI+CM | TBI 28 |
| EBF4 | TsTAQTTSLVSLPSGDQGI | 1.8155 | TBI+CM | TBI 28 |
| SYNE2 | KCLKmLDmSFKDAER | 1.7208 | TBI+CM | TBI 28 |
| SYNE2 | KcLKMLDmSFKDAER | 1.7208 | TBI+CM | TBI 28 |
| SYNE2 | KCLKmLDmSFKDAER | 1.7208 | TBI+CM | TBI 28 |
| SYNE2 | KcLKMLDmSFKDAER | 1.7208 | TBI+CM | TBI 28 |
| SYNE2 | KCLKmLDmSFKDAER | 1.7208 | TBI+CM | TBI 28 |
| SYNE2 | KcLKMLDmSFKDAER | 1.7208 | TBI+CM | TBI 28 |
| SYNE2 | KCLKmLDmSFKDAER | 1.7208 | TBI+CM | TBI 28 |
| SYNE2 | KcLKMLDmSFKDAER | 1.7208 | TBI+CM | TBI 28 |
| SYNE2 | KCLKmLDmSFKDAER | 1.7208 | TBI+CM | TBI 28 |
| SYNE2 | KcLKMLDmSFKDAER | 1.7208 | TBI+CM | TBI 28 |
| SYNE2 | KCLKmLDmSFKDAER | 1.7208 | TBI+CM | TBI 28 |
| SYNE2 | KCLKmLDmSFKDAER | 1.7208 | TBI+CM | TBI 28 |
| SYNE2 | KcLKMLDmSFKDAER | 1.7208 | TBI+CM | TBI 28 |
| PRRC2A | KQEQSDPKSSDAsTAQP | 3.1617 | TBI+CM | TBI 3 |
| PRRC2A | KQEQSDPKSSDAsTAQP | 3.1617 | TBI+CM | TBI 3 |
| PRRC2A | KQEQSDPKSSDAsTAQP | 3.1617 | TBI+CM | TBI 3 |
| PRRC2A | KQEQSDPKSSDAsTAQP | 3.1617 | TBI+CM | TBI 3 |
| PRRC2A | KQEQSDPKSSDAsTAQP | 3.1617 | TBI+CM | TBI 3 |
| PRRC2A | KQEQSDPKSSDAsTAQP | 3.1617 | TBI+CM | TBI 3 |
| PRRC2A | KQEQSDPKSSDAsTAQP | 3.1617 | TBI+CM | TBI 3 |
| PRRC2A | KQEQSDPKSSDAsTAQP | 3.1617 | TBI+CM | TBI 3 |
| PRRC2A | KQEQSDPKSSDAsTAQP | 3.1617 | TBI+CM | TBI 3 |
| PRRC2A | KQEQSDPKSSDAsTAQP | 3.1617 | TBI+CM | TBI 3 |
| PRRC2A | KQEQSDPKSSDAsTAQP | 3.1617 | TBI+CM | TBI 3 |
| PRRC2A | KQEQSDPKSSDAsTAQP | 3.1617 | TBI+CM | TBI 3 |
| PRRC2A | KQEQSDPKSSDAsTAQP | 3.1617 | TBI+CM | TBI 3 |
| PRRC2A | KQEQSDPKSSDAsTAQP | 3.1617 | TBI+CM | TBI 3 |
| PRRC2A | KQEQSDPKSSDAsTAQP | 3.1617 | TBI+CM | TBI 3 |
| PRRC2A | KLKFSDEEDGRDSDEEG | 3.1617 | TBI+CM | TBI 3 |
| FBN1 | IPtPGScRCECNK | 2.9791 | TBI+CM | TBI 3 |
| FBN1 | QHCNIRcMNGGScsDD | 2.9791 | TBI+CM | TBI 3 |
| MT-ND5 | ctHAFFKAMLFMcSG | 2.0189 | TBI+CM | TBI 3 |
| MT-ND5 | ctHAFFKAMLFMcSG | 2.0189 | TBI+CM | TBI 3 |
| MT-ND5 | ctHAFFKAMLFMcSG | 2.0189 | TBI+CM | TBI 3 |
| MT-ND5 | ctHAFFKAMLFMcSG | 2.0189 | TBI+CM | TBI 3 |
| MT-ND5 | ctHAFFKAMLFMcSG | 2.0189 | TBI+CM | TBI 3 |
| MT-ND5 | ctHAFFKAMLFMcSG | 2.0189 | TBI+CM | TBI 3 |
| MT-ND5 | ctHAFFKAMLFMcSG | 2.0189 | TBI+CM | TBI 3 |
| MT-ND5 | ctHAFFKAMLFMcSG | 2.0189 | TBI+CM | TBI 3 |
| MT-ND5 | ctHAFFKAMLFMcSG | 2.0189 | TBI+CM | TBI 3 |
| MT-ND5 | ctHAFFKAMLFMcSG | 2.0189 | TBI+CM | TBI 3 |
| MT-ND5 | ctHAFFKAMLFMcSG | 2.0189 | TBI+CM | TBI 3 |
| MT-ND5 | ctHAFFKAMLFMcSG | 2.0189 | TBI+CM | TBI 3 |
| MT-ND5 | ctHAFFKAMLFMcSG | 2.0189 | TBI+CM | TBI 3 |
| MT-ND5 | ctHAFFKAMLFMcSG | 2.0189 | TBI+CM | TBI 3 |
| MT-ND5 | ctHAFFKAMLFMcSG | 2.0189 | TBI+CM | TBI 3 |
| MT-ND5 | ctHAFFKAMLFMcSG | 2.0189 | TBI+CM | TBI 3 |
| MT-ND5 | ctHAFFKAMLFMcSG | 2.0189 | TBI+CM | TBI 3 |
| MT-ND5 | ctHAFFKAMLFMcSG | 2.0189 | TBI+CM | TBI 3 |
| MT-ND5 | ctHAFFKAMLFMcSG | 2.0189 | TBI+CM | TBI 3 |
| MT-ND5 | ctHAFFKAMLFMcSG | 2.0189 | TBI+CM | TBI 3 |
| MT-ND5 | ctHAFFKAMLFMcSG | 2.0189 | TBI+CM | TBI 3 |
| MT-ND5 | ctHAFFKAMLFMcSG | 2.0189 | TBI+CM | TBI 3 |
| MT-ND5 | ctHAFFKAMLFMcSG | 2.0189 | TBI+CM | TBI 3 |
| MT-ND5 | ctHAFFKAMLFMcSG | 2.0189 | TBI+CM | TBI 3 |
| MT-ND5 | ctHAFFKAMLFMcSG | 2.0189 | TBI+CM | TBI 3 |
| MT-ND5 | ctHAFFKAMLFMcSG | 2.0189 | TBI+CM | TBI 3 |
| MT-ND5 | ctHAFFKAMLFMcSG | 2.0189 | TBI+CM | TBI 3 |
| MT-ND5 | ctHAFFKAMLFMcSG | 2.0189 | TBI+CM | TBI 3 |
| MT-ND5 | ctHAFFKAMLFMcSG | 2.0189 | TBI+CM | TBI 3 |
| MT-ND5 | ctHAFFKAMLFMcSG | 2.0189 | TBI+CM | TBI 3 |
| MT-ND5 | ctHAFFKAMLFMcSG | 2.0189 | TBI+CM | TBI 3 |
| MT-ND5 | ctHAFFKAMLFMcSG | 2.0189 | TBI+CM | TBI 3 |
| MT-ND5 | ctHAFFKAMLFMcSG | 2.0189 | TBI+CM | TBI 3 |
| MT-ND5 | ctHAFFKAMLFMcSG | 2.0189 | TBI+CM | TBI 3 |
| MT-ND5 | ctHAFFKAMLFMcSG | 2.0189 | TBI+CM | TBI 3 |
| MT-ND5 | ctHAFFKAMLFMcSG | 2.0189 | TBI+CM | TBI 3 |
| MT-ND5 | ctHAFFKAMLFMcSG | 2.0189 | TBI+CM | TBI 3 |
| MT-ND5 | ctHAFFKAMLFMcSG | 2.0189 | TBI+CM | TBI 3 |
| MT-ND5 | ctHAFFKAMLFMcSG | 2.0189 | TBI+CM | TBI 3 |
| MT-ND5 | ctHAFFKAMLFMcSG | 2.0189 | TBI+CM | TBI 3 |
| MT-ND5 | ctHAFFKAMLFMcSG | 2.0189 | TBI+CM | TBI 3 |
| MT-ND5 | ctHAFFKAMLFMcSG | 2.0189 | TBI+CM | TBI 3 |
| MT-ND5 | ctHAFFKAMLFMcSG | 2.0189 | TBI+CM | TBI 3 |
| MT-ND5 | ctHAFFKAMLFMcSG | 2.0189 | TBI+CM | TBI 3 |
| MT-ND5 | ctHAFFKAMLFMcSG | 2.0189 | TBI+CM | TBI 3 |
| MT-ND5 | ctHAFFKAMLFMcSG | 2.0189 | TBI+CM | TBI 3 |
| MT-ND5 | ctHAFFKAMLFMcSG | 2.0189 | TBI+CM | TBI 3 |
| MT-ND5 | ctHAFFKAMLFMcSG | 2.0189 | TBI+CM | TBI 3 |
| MT-ND5 | ctHAFFKAMLFMcSG | 2.0189 | TBI+CM | TBI 3 |
| MT-ND5 | ctHAFFKAMLFMcSG | 2.0189 | TBI+CM | TBI 3 |
| MT-ND5 | ctHAFFKAMLFMcSG | 2.0189 | TBI+CM | TBI 3 |
| MT-ND5 | ctHAFFKAMLFMcSG | 2.0189 | TBI+CM | TBI 3 |
| MT-ND5 | ctHAFFKAMLFMcSG | 2.0189 | TBI+CM | TBI 3 |
| MT-ND5 | ctHAFFKAMLFMcSG | 2.0189 | TBI+CM | TBI 3 |
| MT-ND5 | ctHAFFKAMLFMcSG | 2.0189 | TBI+CM | TBI 3 |
| MT-ND5 | ctHAFFKAMLFMcSG | 2.0189 | TBI+CM | TBI 3 |
| MT-ND5 | ctHAFFKAMLFMcSG | 2.0189 | TBI+CM | TBI 3 |
| MT-ND5 | ctHAFFKAMLFMcSG | 2.0189 | TBI+CM | TBI 3 |
| MT-ND5 | ctHAFFKAMLFMcSG | 2.0189 | TBI+CM | TBI 3 |
| MT-ND5 | ctHAFFKAMLFMcSG | 2.0189 | TBI+CM | TBI 3 |
| MT-ND5 | ctHAFFKAMLFMcSG | 2.0189 | TBI+CM | TBI 3 |
| MT-ND5 | ctHAFFKAMLFMcSG | 2.0189 | TBI+CM | TBI 3 |
| MT-ND5 | ctHAFFKAMLFMcSG | 2.0189 | TBI+CM | TBI 3 |
| MT-ND5 | ctHAFFKAMLFMcSG | 2.0189 | TBI+CM | TBI 3 |
| MT-ND5 | ctHAFFKAMLFMcSG | 2.0189 | TBI+CM | TBI 3 |
| MT-ND5 | ctHAFFKAMLFMcSG | 2.0189 | TBI+CM | TBI 3 |
| MT-ND5 | ctHAFFKAMLFMcSG | 2.0189 | TBI+CM | TBI 3 |
| MT-ND5 | ctHAFFKAMLFMcSG | 2.0189 | TBI+CM | TBI 3 |
| MT-ND5 | ctHAFFKAMLFMcSG | 2.0189 | TBI+CM | TBI 3 |
| MT-ND5 | ctHAFFKAMLFMcSG | 2.0189 | TBI+CM | TBI 3 |
| MT-ND5 | ctHAFFKAMLFMcSG | 2.0189 | TBI+CM | TBI 3 |
| MT-ND5 | ctHAFFKAMLFMcSG | 2.0189 | TBI+CM | TBI 3 |
| MT-ND5 | ctHAFFKAMLFMcSG | 2.0189 | TBI+CM | TBI 3 |
| MT-ND5 | ctHAFFKAMLFMcSG | 2.0189 | TBI+CM | TBI 3 |
| MT-ND5 | ctHAFFKAMLFMcSG | 2.0189 | TBI+CM | TBI 3 |
| SYNE2 | yTTQLEDLRQEASNL | 1.9589 | TBI+CM | TBI 3 |
| SYNE2 | YtTQLEDLRQEASNL | 1.9589 | TBI+CM | TBI 3 |
| SYNE2 | YTtQLEDLRQEASNL | 1.9589 | TBI+CM | TBI 3 |
| SYNE2 | yTTQLEDLRQEASNL | 1.9589 | TBI+CM | TBI 3 |
| SYNE2 | YtTQLEDLRQEASNL | 1.9589 | TBI+CM | TBI 3 |
| SYNE2 | YTtQLEDLRQEASNL | 1.9589 | TBI+CM | TBI 3 |
| SYNE2 | yTTQLEDLRQEASNL | 1.9589 | TBI+CM | TBI 3 |
| SYNE2 | YtTQLEDLRQEASNL | 1.9589 | TBI+CM | TBI 3 |
| SYNE2 | YTtQLEDLRQEASNL | 1.9589 | TBI+CM | TBI 3 |
| SYNE2 | yTTQLEDLRQEASNL | 1.9589 | TBI+CM | TBI 3 |
| SYNE2 | YtTQLEDLRQEASNL | 1.9589 | TBI+CM | TBI 3 |
| SYNE2 | YTtQLEDLRQEASNL | 1.9589 | TBI+CM | TBI 3 |
| SYNE2 | yTTQLEDLRQEASNL | 1.9589 | TBI+CM | TBI 3 |
| SYNE2 | YtTQLEDLRQEASNL | 1.9589 | TBI+CM | TBI 3 |
| SYNE2 | YTtQLEDLRQEASNL | 1.9589 | TBI+CM | TBI 3 |
| SYNE2 | yTTQLEDLRQEASNL | 1.9589 | TBI+CM | TBI 3 |
| SYNE2 | yTTQLEDLRQEASNL | 1.9589 | TBI+CM | TBI 3 |
| SYNE2 | YtTQLEDLRQEASNL | 1.9589 | TBI+CM | TBI 3 |
| SYNE2 | yTTQLEDLRQEASNL | 1.9589 | TBI+CM | TBI 3 |
| SYNE2 | YTtQLEDLRQEASNL | 1.9589 | TBI+CM | TBI 3 |
| SYNE2 | yTTQLEDLRQEASNL | 1.9589 | TBI+CM | TBI 3 |
| SYNE2 | yTTQLEDLRQEASNL | 1.9589 | TBI+CM | TBI 3 |
| SYNE2 | yTTQLEDLRQEASNL | 1.9589 | TBI+CM | TBI 3 |
| SYNE2 | yTTQLEDLRQEASNL | 1.9589 | TBI+CM | TBI 3 |
| SYNE2 | yTTQLEDLRQEASNL | 1.9589 | TBI+CM | TBI 3 |
| TTN | CPLENGGRSSIsHLKKAA | 1.9512 | TBI+CM | TBI 3 |
| TTN | CPLENGGRSSIsHLKKAA | 1.9512 | TBI+CM | TBI 3 |
| TTN | CPLENGGRSSIsHLKKAA | 1.9512 | TBI+CM | TBI 3 |
| TTN | CPLENGGRSSIsHLKKAA | 1.9512 | TBI+CM | TBI 3 |
| TTN | CPLENGGRSSIsHLKKAA | 1.9512 | TBI+CM | TBI 3 |
| MUC19 | PTRPQVsQPETTVVATR | 1.9496 | TBI+CM | TBI 3 |
| MUC19 | PTRPQVsQPETTVVATR | 1.9496 | TBI+CM | TBI 3 |
| MUC19 | PTRPQVsQPETTVVATR | 1.9496 | TBI+CM | TBI 3 |
| SYNE2 | YtTQLEDLRQEASNL | 1.9438 | TBI+CM | TBI 3 |
| SYNE2 | YtTQLEDLRQEASNL | 1.9438 | TBI+CM | TBI 3 |
| SYNE2 | YtTQLEDLRQEASNL | 1.9438 | TBI+CM | TBI 3 |
| SYNE2 | YtTQLEDLRQEASNL | 1.9438 | TBI+CM | TBI 3 |
| SYNE2 | YtTQLEDLRQEASNL | 1.9438 | TBI+CM | TBI 3 |
| SYNE2 | YtTQLEDLRQEASNL | 1.9438 | TBI+CM | TBI 3 |
| SYNE2 | YtTQLEDLRQEASNL | 1.9438 | TBI+CM | TBI 3 |
| PAPPA | mHLDDDcTDsFTPN | 1.8226 | TBI+CM | TBI 3 |
| PAPPA | mHLDDDcTDsFTPN | 1.8226 | TBI+CM | TBI 3 |
| C7 | YAPWSECNGcTKt | 1.8091 | TBI+CM | TBI 3 |
| C7 | YAPWSECNGcTKt | 1.8091 | TBI+CM | TBI 3 |
| C7 | YAPWSECNGcTKt | 1.8091 | TBI+CM | TBI 3 |
| C7 | YAPWSECNGcTKt | 1.8091 | TBI+CM | TBI 3 |
| C7 | YAPWSECNGcTKt | 1.8091 | TBI+CM | TBI 3 |
| C7 | YAPWSECNGcTKt | 1.8091 | TBI+CM | TBI 3 |
| C7 | YAPWSECNGcTKt | 1.8091 | TBI+CM | TBI 3 |
| WDR46 | GSVScLyPHPIPCGcCG | 1.7668 | TBI+CM | TBI 3 |
| WDR46 | GSVScLyPHPIPCGcCG | 1.7668 | TBI+CM | TBI 3 |
| WDR46 | GSVScLyPHPIPCGcCG | 1.7668 | TBI+CM | TBI 3 |
| WDR46 | GSVScLyPHPIPCGcCG | 1.7668 | TBI+CM | TBI 3 |
| WDR46 | GSVScLyPHPIPCGcCG | 1.7668 | TBI+CM | TBI 3 |
| NOTCH4 | GcEGRSGDGACDAGcs | 1.7535 | TBI+CM | TBI 3 |
| NOTCH4 | GcEGRSGDGACDAGcs | 1.7535 | TBI+CM | TBI 3 |
| NOTCH4 | GcEGRSGDGACDAGcs | 1.7535 | TBI+CM | TBI 3 |
| NOTCH4 | GcEGRSGDGACDAGcs | 1.7535 | TBI+CM | TBI 3 |
| NOTCH4 | GcEGRSGDGACDAGcs | 1.7535 | TBI+CM | TBI 3 |
| NOTCH4 | GcEGRSGDGACDAGcs | 1.7535 | TBI+CM | TBI 3 |
| SYNE2 | YTtQLEDLRQEASNL | 1.7468 | TBI+CM | TBI 3 |
| SYNE2 | YTtQLEDLRQEASNL | 1.7468 | TBI+CM | TBI 3 |
| SYNE2 | YTtQLEDLRQEASNL | 1.7468 | TBI+CM | TBI 3 |
| SYNE2 | YTtQLEDLRQEASNL | 1.7468 | TBI+CM | TBI 3 |
| SYNE2 | YTtQLEDLRQEASNL | 1.7468 | TBI+CM | TBI 3 |
| SYNE2 | YTtQLEDLRQEASNL | 1.7468 | TBI+CM | TBI 3 |
| SYNE2 | YTtQLEDLRQEASNL | 1.7468 | TBI+CM | TBI 3 |
| MT-ND1 | ALTtALLLWTPLPmPNP | 2.2514 | TBI+CM | TBI 31 |
| MT-ND1 | ALTtALLLWTPLPmPNP | 2.2514 | TBI+CM | TBI 31 |
| MT-ND1 | ALTtALLLWTPLPmPNP | 2.2514 | TBI+CM | TBI 31 |
| MT-ND1 | ALtTALLLWTPLPmPNP | 2.2514 | TBI+CM | TBI 31 |
| MT-ND1 | ALTtALLLWTPLPmPNP | 2.2514 | TBI+CM | TBI 31 |
| MT-ND1 | ALtTALLLWTPLPmPNP | 2.2295 | TBI+CM | TBI 31 |
| MT-ND1 | ALtTALLLWTPLPmPNP | 2.2295 | TBI+CM | TBI 31 |
| MT-ND1 | ALtTALLLWTPLPmPNP | 2.2295 | TBI+CM | TBI 31 |
| CRCT1 | RRQRSSGccccGGGSQRS | 2.135 | TBI+CM | TBI 31 |
| CRCT1 | RRQRSSGccccGGGSQRS | 2.135 | TBI+CM | TBI 31 |
| CRCT1 | RRQRSSGccccGGGSQRS | 2.135 | TBI+CM | TBI 31 |
| CRCT1 | RRQRSSGccccGGGSQRS | 2.135 | TBI+CM | TBI 31 |
| CRCT1 | RRQRSSGccccGGGSQRS | 2.135 | TBI+CM | TBI 31 |
| ZKSCAN7 | PYKcNEcAKAFTQSS | 2.0607 | TBI+CM | TBI 31 |
| ZKSCAN7 | PYKcNEcAKAFTQSS | 2.0607 | TBI+CM | TBI 31 |
| ZKSCAN7 | PYKcNEcAKAFTQSS | 2.0607 | TBI+CM | TBI 31 |
| ZKSCAN7 | PYKcNEcAKAFTQSS | 2.0607 | TBI+CM | TBI 31 |
| ZKSCAN7 | PYKcNEcAKAFTQSS | 2.0607 | TBI+CM | TBI 31 |
| ZKSCAN7 | PYKcNEcAKAFTQSS | 2.0607 | TBI+CM | TBI 31 |
| MT1M | DPNcSCtTGVSCAcTGSCK | 2.0282 | TBI+CM | TBI 31 |
| MT1M | DPNcSCtTGVSCAcTGSCK | 2.0282 | TBI+CM | TBI 31 |
| MT1M | DPNcSCtTGVSCAcTGSCK | 2.0282 | TBI+CM | TBI 31 |
| MT1M | DPNcSCtTGVScACTGSCK | 2.0282 | TBI+CM | TBI 31 |
| MT1M | DPNcSCtTGVSCAcTGSCK | 2.0282 | TBI+CM | TBI 31 |
| NOTCH4 | YcQCLPGHTGQWc | 2.0177 | TBI+CM | TBI 31 |
| NOTCH4 | YcQCLPGHTGQWc | 2.0177 | TBI+CM | TBI 31 |
| NOTCH4 | YcQCLPGHTGQWc | 2.0177 | TBI+CM | TBI 31 |
| NOTCH4 | YcQCLPGHTGQWc | 2.0177 | TBI+CM | TBI 31 |
| NOTCH4 | YcQCLPGHTGQWc | 2.0177 | TBI+CM | TBI 31 |
| CRCT1 | RRQRSSGccccGGGSQRS | 2.0154 | TBI+CM | TBI 31 |
| CRCT1 | RRQRSSGccccGGGSQRS | 2.0154 | TBI+CM | TBI 31 |
| IGFN1 | QGcECcmScAVQGSPRPH | 1.9467 | TBI+CM | TBI 31 |
| IGFN1 | QGcECcmScAVQGSPRPH | 1.9467 | TBI+CM | TBI 31 |
| IGFN1 | QGcECcmScAVQGSPRPH | 1.9467 | TBI+CM | TBI 31 |
| IGFN1 | QGcECcmScAVQGSPRPH | 1.9467 | TBI+CM | TBI 31 |
| IGFN1 | QGcECcmScAVQGSPRPH | 1.9467 | TBI+CM | TBI 31 |
| IGFN1 | QGcECcmScAVQGSPRPH | 1.9467 | TBI+CM | TBI 31 |
| IGFN1 | QGcECcmScAVQGSPRPH | 1.9467 | TBI+CM | TBI 31 |
| IGFN1 | QGcECcmScAVQGSPRPH | 1.9467 | TBI+CM | TBI 31 |
| IGFN1 | QGcECcmScAVQGSPRPH | 1.9467 | TBI+CM | TBI 31 |
| IGFN1 | QGcECcmScAVQGSPRPH | 1.9467 | TBI+CM | TBI 31 |
| IGFN1 | QGcECcmScAVQGSPRPH | 1.9467 | TBI+CM | TBI 31 |
| IGFN1 | QGcECcmScAVQGSPRPH | 1.9467 | TBI+CM | TBI 31 |
| IGFN1 | QGcECcmScAVQGSPRPH | 1.9467 | TBI+CM | TBI 31 |
| CRCT1 | RRQRSSGccccGGGSQRS | 1.9379 | TBI+CM | TBI 31 |
| CRCT1 | RRQRSSGccccGGGSQRS | 1.9379 | TBI+CM | TBI 31 |
| IGFN1 | QGcECcmScAVQGSPRPH | 1.9114 | TBI+CM | TBI 31 |
| IGFN1 | QGcECcmScAVQGSPRPH | 1.9114 | TBI+CM | TBI 31 |
| IGFN1 | QGcECcmScAVQGSPRPH | 1.9114 | TBI+CM | TBI 31 |
| IGFN1 | QGcECcmScAVQGSPRPH | 1.9114 | TBI+CM | TBI 31 |
| IGFN1 | QGcECcmScAVQGSPRPH | 1.9114 | TBI+CM | TBI 31 |
| IGFN1 | QGcECcmScAVQGSPRPH | 1.9114 | TBI+CM | TBI 31 |
| IGFN1 | QGcECcmScAVQGSPRPH | 1.9114 | TBI+CM | TBI 31 |
| MT-ND1 | YLIcVLCETNRt | 1.8693 | TBI+CM | TBI 31 |
| MT-ND1 | YLIcVLCETNRt | 1.8693 | TBI+CM | TBI 31 |
| MT-ND1 | YLIcVLCETNRt | 1.8693 | TBI+CM | TBI 31 |
| MT-ND1 | YLIcVLCETNRt | 1.8693 | TBI+CM | TBI 31 |
| MT-ND1 | YLIcVLCETNRt | 1.8693 | TBI+CM | TBI 31 |
| MT-ND1 | YLIcVLCETNRt | 1.8693 | TBI+CM | TBI 31 |
| MT-ND1 | YLIcVLCETNRt | 1.8693 | TBI+CM | TBI 31 |
| MT-ND1 | YLIcVLCETNRt | 1.8693 | TBI+CM | TBI 31 |
| MT-ND1 | YLIcVLCETNRt | 1.8693 | TBI+CM | TBI 31 |
| MT-ND1 | YLIcVLCETNRt | 1.8693 | TBI+CM | TBI 31 |
| MT-ND1 | YLIcVLCETNRt | 1.8693 | TBI+CM | TBI 31 |
| MT-ND1 | YLIcVLCETNRt | 1.8693 | TBI+CM | TBI 31 |
| MT-ND1 | YLIcVLCETNRt | 1.8693 | TBI+CM | TBI 31 |
| MT-ND1 | YLIcVLCETNRt | 1.8693 | TBI+CM | TBI 31 |
| MT-ND1 | YLIcVLCETNRt | 1.8693 | TBI+CM | TBI 31 |
| MT-ND1 | YLIcVLCETNRt | 1.8693 | TBI+CM | TBI 31 |
| MT-ND1 | YLIcVLCETNRt | 1.8693 | TBI+CM | TBI 31 |
| MT-ND1 | YLIcVLCETNRt | 1.8693 | TBI+CM | TBI 31 |
| MT-ND1 | YLIcVLCETNRt | 1.8693 | TBI+CM | TBI 31 |
| MT-ND1 | YLIcVLCETNRt | 1.8693 | TBI+CM | TBI 31 |
| MT-ND1 | YLIcVLCETNRt | 1.8693 | TBI+CM | TBI 31 |
| MT-ND1 | YLIcVLCETNRt | 1.8693 | TBI+CM | TBI 31 |
| MT-ND1 | YLIcVLCETNRt | 1.8693 | TBI+CM | TBI 31 |
| MT-ND1 | YLIcVLCETNRt | 1.8693 | TBI+CM | TBI 31 |
| MT-ND1 | YLIcVLCETNRt | 1.8693 | TBI+CM | TBI 31 |
| MT-ND1 | YLIcVLCETNRt | 1.8693 | TBI+CM | TBI 31 |
| MT-ND1 | YLIcVLCETNRt | 1.8693 | TBI+CM | TBI 31 |
| MT-ND1 | YLIcVLCETNRt | 1.8693 | TBI+CM | TBI 31 |
| MT-ND1 | YLIcVLCETNRt | 1.8693 | TBI+CM | TBI 31 |
| MT-ND1 | YLIcVLCETNRt | 1.8693 | TBI+CM | TBI 31 |
| MT-ND1 | YLIcVLCETNRt | 1.8693 | TBI+CM | TBI 31 |
| MT-ND1 | YLIcVLCETNRt | 1.8693 | TBI+CM | TBI 31 |
| MT-ND1 | YLIcVLCETNRt | 1.8693 | TBI+CM | TBI 31 |
| MT-ND1 | YLIcVLCETNRt | 1.8693 | TBI+CM | TBI 31 |
| MT-ND1 | YLIcVLCETNRt | 1.8693 | TBI+CM | TBI 31 |
| MT-ND1 | YLIcVLCETNRt | 1.8693 | TBI+CM | TBI 31 |
| MT-ND1 | YLIcVLCETNRt | 1.8693 | TBI+CM | TBI 31 |
| MT-ND1 | YLIcVLCETNRt | 1.8693 | TBI+CM | TBI 31 |
| MT-ND1 | YLIcVLCETNRt | 1.8693 | TBI+CM | TBI 31 |
| MT-ND1 | YLIcVLCETNRt | 1.8693 | TBI+CM | TBI 31 |
| MT-ND1 | YLIcVLCETNRt | 1.8693 | TBI+CM | TBI 31 |
| MT-ND1 | YLIcVLCETNRt | 1.8693 | TBI+CM | TBI 31 |
| MT-ND1 | YLIcVLCETNRt | 1.8693 | TBI+CM | TBI 31 |
| MT-ND1 | YLIcVLCETNRt | 1.8693 | TBI+CM | TBI 31 |
| MT-ND1 | YLIcVLCETNRt | 1.8693 | TBI+CM | TBI 31 |
| MT-ND1 | YLIcVLCETNRt | 1.8693 | TBI+CM | TBI 31 |
| MT-ND1 | YLIcVLCETNRt | 1.8693 | TBI+CM | TBI 31 |
| MT-ND1 | YLIcVLCETNRt | 1.8693 | TBI+CM | TBI 31 |
| MT-ND1 | YLIcVLCETNRt | 1.8693 | TBI+CM | TBI 31 |
| MT-ND1 | YLIcVLCETNRt | 1.8693 | TBI+CM | TBI 31 |
| MT-ND1 | YLIcVLCETNRt | 1.8693 | TBI+CM | TBI 31 |
| MT-ND1 | YLIcVLCETNRt | 1.8693 | TBI+CM | TBI 31 |
| MT-ND1 | YLIcVLCETNRt | 1.8693 | TBI+CM | TBI 31 |
| MT-ND1 | YLIcVLCETNRt | 1.8693 | TBI+CM | TBI 31 |
| MT-ND1 | YLIcVLCETNRt | 1.8693 | TBI+CM | TBI 31 |
| MT-ND1 | YLIcVLCETNRt | 1.8693 | TBI+CM | TBI 31 |
| MT-ND1 | YLIcVLCETNRt | 1.8693 | TBI+CM | TBI 31 |
| MT-ND1 | YLIcVLCETNRt | 1.8693 | TBI+CM | TBI 31 |
| MT-ND1 | YLIcVLCETNRt | 1.8693 | TBI+CM | TBI 31 |
| MT-ND1 | YLIcVLCETNRt | 1.8693 | TBI+CM | TBI 31 |
| MT-ND1 | YLIcVLCETNRt | 1.8693 | TBI+CM | TBI 31 |
| MT-ND1 | YLIcVLCETNRt | 1.8693 | TBI+CM | TBI 31 |
| MT-ND1 | YLIcVLCETNRt | 1.8693 | TBI+CM | TBI 31 |
| MT-ND1 | YLIcVLCETNRt | 1.8693 | TBI+CM | TBI 31 |
| MT-ND1 | YLIcVLCETNRt | 1.8693 | TBI+CM | TBI 31 |
| MT-ND1 | YLIcVLCETNRt | 1.8693 | TBI+CM | TBI 31 |
| MT-ND1 | YLIcVLCETNRt | 1.8693 | TBI+CM | TBI 31 |
| MT-ND1 | YLIcVLCETNRt | 1.8693 | TBI+CM | TBI 31 |
| MT-ND1 | YLIcVLCETNRt | 1.8693 | TBI+CM | TBI 31 |
| MT-ND1 | YLIcVLCETNRt | 1.8693 | TBI+CM | TBI 31 |
| MT-ND1 | YLIcVLCETNRt | 1.8693 | TBI+CM | TBI 31 |
| MT-ND1 | YIIcVLCETNRt | 1.8693 | TBI+CM | TBI 31 |
| MT-ND1 | YIIcVLCETNRt | 1.8693 | TBI+CM | TBI 31 |
| MT-ND1 | YIIcVLCETNRt | 1.8693 | TBI+CM | TBI 31 |
| TRB | FcASSSGVStDtQy | 1.8437 | TBI+CM | TBI 31 |
| TRB | FcASSSGVStDtQy | 1.8437 | TBI+CM | TBI 31 |
| TRB | FcASSSGVStDtQy | 1.8437 | TBI+CM | TBI 31 |
| CTSW | VsVHELLDCGRCGDGcH | 1.8419 | TBI+CM | TBI 31 |
| CTSW | VsVHELLDCGRCGDGcH | 1.8419 | TBI+CM | TBI 31 |
| CTSW | VsVHELLDCGRCGDGcH | 1.8419 | TBI+CM | TBI 31 |
| MTO1 | CSGmITARcsLDHL | 1.8179 | TBI+CM | TBI 31 |
| MTO1 | CSGmITARcsLDHL | 1.8179 | TBI+CM | TBI 31 |
| MTO1 | CSGmITARcsLDHL | 1.8179 | TBI+CM | TBI 31 |
| KIAA2026 | EKGTActSTRRRSTP | 1.7192 | TBI+CM | TBI 31 |
| KIAA2026 | EKGTActSTRRRSTP | 1.7192 | TBI+CM | TBI 31 |
| KIAA2026 | EKGTActSTRRRSTP | 1.7192 | TBI+CM | TBI 31 |
| KIAA2026 | EKGTActSTRRRSTP | 1.7192 | TBI+CM | TBI 31 |
| KIAA2026 | EKGTActSTRRRSTP | 1.7192 | TBI+CM | TBI 31 |
| TTN | TFINKVASLKIPCAEms | 3.9765 | TBI+CM | TBI 35 |
| TTN | TFINKVASLKIPCAEms | 3.9765 | TBI+CM | TBI 35 |
| TTN | TFINKVASLKIPCAEms | 3.9765 | TBI+CM | TBI 35 |
| TTN | IPPsFTKKLKKmDSIK | 3.9765 | TBI+CM | TBI 35 |
| TTN | TFINKVASLKIPcAEMs | 3.9765 | TBI+CM | TBI 35 |
| TTN | TFINKVASLKIPCAEms | 3.9765 | TBI+CM | TBI 35 |
| SYNE2 | AFQEItSMKERc | 3.0739 | TBI+CM | TBI 35 |
| SYNE2 | KcLKMLDmSFKDAER | 3.0739 | TBI+CM | TBI 35 |
| SYNE2 | AFQEItSMKERc | 3.0739 | TBI+CM | TBI 35 |
| SYNE2 | AFQEItSMKERc | 3.0739 | TBI+CM | TBI 35 |
| SYNE2 | AFQEItSMKERc | 3.0739 | TBI+CM | TBI 35 |
| SYNE2 | AFQEItSMKERc | 3.0739 | TBI+CM | TBI 35 |
| SYNE2 | AFQEItSMKERc | 3.0739 | TBI+CM | TBI 35 |
| SYNE2 | AFQEItSMKERc | 3.0739 | TBI+CM | TBI 35 |
| SYNE2 | AFQEItSMKERc | 3.0739 | TBI+CM | TBI 35 |
| SYNE2 | AFQEItSMKERc | 3.0739 | TBI+CM | TBI 35 |
| PCLO | PIQESRDLEPDYSsY | 2.3353 | TBI+CM | TBI 35 |
| PCLO | PIQESRDLEPDYSSy | 2.3353 | TBI+CM | TBI 35 |
| PCLO | PIQESRDLEPDYSsY | 2.3353 | TBI+CM | TBI 35 |
| PCLO | PIQESRDLEPDYSSy | 2.3353 | TBI+CM | TBI 35 |
| PCLO | PIQESRDLEPDYSsY | 2.3353 | TBI+CM | TBI 35 |
| PCLO | PIQESRDLEPDYSSy | 2.3353 | TBI+CM | TBI 35 |
| PCLO | PIQESRDLEPDYSsY | 2.3353 | TBI+CM | TBI 35 |
| PCLO | PIQESRDLEPDYSSy | 2.3353 | TBI+CM | TBI 35 |
| PCLO | PIQESRDLEPDYSsY | 2.3353 | TBI+CM | TBI 35 |
| PCLO | PIQESRDLEPDYSSy | 2.3353 | TBI+CM | TBI 35 |
| PCLO | PIQESRDLEPDYSsY | 2.3353 | TBI+CM | TBI 35 |
| PCLO | PIQESRDLEPDYSsY | 2.3353 | TBI+CM | TBI 35 |
| PCLO | PIQESRDLEPDYSsY | 2.3353 | TBI+CM | TBI 35 |
| PCLO | PIQESRDLEPDYSSy | 2.3353 | TBI+CM | TBI 35 |
| PCLO | PIQESRDLEPDYSsY | 2.3353 | TBI+CM | TBI 35 |
| MT1M | cKCtSCKKSCcSCCPVGC | 2.3194 | TBI+CM | TBI 35 |
| MT1M | cKCtSCKKSCcSCCPVGC | 2.3194 | TBI+CM | TBI 35 |
| MT1M | cKCtSCKKSCcSCCPVGC | 2.3194 | TBI+CM | TBI 35 |
| MT1M | cKCtSCKKSCcSCCPVGC | 2.3194 | TBI+CM | TBI 35 |
| MT1M | cKCtSCKKSCcSCCPVGC | 2.3194 | TBI+CM | TBI 35 |
| MT1M | cKCtSCKKSCcSCCPVGC | 2.3194 | TBI+CM | TBI 35 |
| MT1M | cKCtSCKKSCcSCCPVGC | 2.3194 | TBI+CM | TBI 35 |
| PCLO | PIQESRDLEPDYSSy | 2.1534 | TBI+CM | TBI 35 |
| PCLO | PIQESRDLEPDYSSy | 2.1534 | TBI+CM | TBI 35 |
| PCLO | PIQESRDLEPDYSSy | 2.1534 | TBI+CM | TBI 35 |
| TTN | TFINKVASLKIPcAEMs | 2.1129 | TBI+CM | TBI 35 |
| TTN | TFINKVASLKIPcAEMs | 2.1129 | TBI+CM | TBI 35 |
| TTN | TFINKVASLKIPcAEMs | 2.1129 | TBI+CM | TBI 35 |
| TTN | TFINKVASLKIPcAEMs | 2.1129 | TBI+CM | TBI 35 |
| TTN | TFINKVASLKIPcAEMs | 2.1129 | TBI+CM | TBI 35 |
| TTN | TFINKVASLKIPcAEMs | 2.1129 | TBI+CM | TBI 35 |
| CRCT1 | GccccGGGSQRSQRSNNR | 2.0405 | TBI+CM | TBI 35 |
| CRCT1 | GccccGGGSQRSQRSNNR | 2.0405 | TBI+CM | TBI 35 |
| LRP2 | EVNNNPCLENNGGcSHLc | 2.0398 | TBI+CM | TBI 35 |
| LRP2 | EVNNNPCLENNGGcSHLc | 2.0398 | TBI+CM | TBI 35 |
| LRP2 | EVNNNPCLENNGGcSHLc | 2.0398 | TBI+CM | TBI 35 |
| POU4F3 | DLLQHFSMEFcsccPG | 1.8821 | TBI+CM | TBI 35 |
| POU4F3 | DLLQHFSMEFcsccPG | 1.8821 | TBI+CM | TBI 35 |
| POU4F3 | DLLQHFSMEFcsccPG | 1.8821 | TBI+CM | TBI 35 |
| POU4F3 | DLLQHFSMEFcsccPG | 1.8821 | TBI+CM | TBI 35 |
| POU4F3 | DLLQHFSMEFcsccPG | 1.8821 | TBI+CM | TBI 35 |
| TTN | IPPsFTKKLKKmDSIK | 1.8636 | TBI+CM | TBI 35 |
| TTN | IPPsFTKKLKKmDSIK | 1.8636 | TBI+CM | TBI 35 |
| TTN | IPPsFTKKLKKmDSIK | 1.8636 | TBI+CM | TBI 35 |
| TTN | IPPsFTKKLKKmDSIK | 1.8636 | TBI+CM | TBI 35 |
| TTN | IPPsFTKKLKKmDSIK | 1.8636 | TBI+CM | TBI 35 |
| TTN | IPPsFTKKLKKmDSIK | 1.8636 | TBI+CM | TBI 35 |
| IGH | TcDVSGVPAPtVTWLKD | 1.8589 | TBI+CM | TBI 35 |
| IGH | TcDVSGVPAPtVTWLKD | 1.8589 | TBI+CM | TBI 35 |
| IGH | TcDVSGVPAPtVTWLKD | 1.8589 | TBI+CM | TBI 35 |
| ZNF879 | cNECGRAFSQcsS | 1.8256 | TBI+CM | TBI 35 |
| ZNF879 | cNECGRAFSQcsS | 1.8256 | TBI+CM | TBI 35 |
| ZNF879 | cNECGRAFSQcsS | 1.8256 | TBI+CM | TBI 35 |
| ZNF879 | cNECGRAFSQcsS | 1.8256 | TBI+CM | TBI 35 |
| IGL | LLLtLLIHcTGAQTQ | 1.8235 | TBI+CM | TBI 35 |
| IGL | LLLtLLIHcTGAQTQ | 1.8235 | TBI+CM | TBI 35 |
| IGL | LLLtLLIHcTGAQTQ | 1.8235 | TBI+CM | TBI 35 |
| ZKSCAN7 | SLTLSQRALQWNmm | 1.8059 | TBI+CM | TBI 35 |
| ZKSCAN7 | SLTLSQRALQWNmm | 1.8059 | TBI+CM | TBI 35 |
| ZKSCAN7 | SLTLSQRALQWNmm | 1.8059 | TBI+CM | TBI 35 |
| ZKSCAN7 | SLTLSQRALQWNmm | 1.8059 | TBI+CM | TBI 35 |
| ZKSCAN7 | SLTLSQRALQWNmm | 1.8059 | TBI+CM | TBI 35 |
| ZKSCAN7 | SLTLSQRALQWNmm | 1.8059 | TBI+CM | TBI 35 |
| ZKSCAN7 | SLTLSQRALQWNmm | 1.8059 | TBI+CM | TBI 35 |
| ZKSCAN7 | SLTLSQRALQWNmm | 1.8059 | TBI+CM | TBI 35 |
| ZKSCAN7 | SLTLSQRALQWNmm | 1.8059 | TBI+CM | TBI 35 |
| ZKSCAN7 | SLTLSQRALQWNmm | 1.8059 | TBI+CM | TBI 35 |
| ZKSCAN7 | SLTLSQRALQWNmm | 1.8059 | TBI+CM | TBI 35 |
| ZKSCAN7 | SLTLSQRALQWNmm | 1.8059 | TBI+CM | TBI 35 |
| ZKSCAN7 | SLTLSQRALQWNmm | 1.8059 | TBI+CM | TBI 35 |
| ZKSCAN7 | SLTLSQRALQWNmm | 1.8059 | TBI+CM | TBI 35 |
| ZKSCAN7 | SLTLSQRALQWNmm | 1.8059 | TBI+CM | TBI 35 |
| ZKSCAN7 | SLTLSQRALQWNmm | 1.8059 | TBI+CM | TBI 35 |
| ZKSCAN7 | SLTLSQRALQWNmm | 1.8059 | TBI+CM | TBI 35 |
| TTN | TFINKVASLKIPCAEms | 1.7992 | TBI+CM | TBI 35 |
| TTN | TFINKVASLKIPCAEms | 1.7992 | TBI+CM | TBI 35 |
| TTN | TFINKVASLKIPCAEms | 1.7992 | TBI+CM | TBI 35 |
| TTN | TFINKVASLKIPCAEms | 1.7992 | TBI+CM | TBI 35 |
| TTN | TFINKVASLKIPCAEms | 1.7992 | TBI+CM | TBI 35 |
| TTN | TFINKVASLKIPCAEms | 1.7992 | TBI+CM | TBI 35 |
| SYNE2 | AFQEItSMKERc | 1.7892 | TBI+CM | TBI 35 |
| SYNE2 | KcLKMLDmSFKDAER | 1.7892 | TBI+CM | TBI 35 |
| SYNE2 | KcLKMLDmSFKDAER | 1.7892 | TBI+CM | TBI 35 |
| SYNE2 | KcLKMLDmSFKDAER | 1.7892 | TBI+CM | TBI 35 |
| SYNE2 | KcLKMLDmSFKDAER | 1.7892 | TBI+CM | TBI 35 |
| SYNE2 | KcLKMLDmSFKDAER | 1.7892 | TBI+CM | TBI 35 |
| SYNE2 | KcLKMLDmSFKDAER | 1.7892 | TBI+CM | TBI 35 |
| SYNE2 | KcLKMLDmSFKDAER | 1.7892 | TBI+CM | TBI 35 |
| SYNE2 | KcLKMLDmSFKDAER | 1.7892 | TBI+CM | TBI 35 |
| SYNE2 | KcLKMLDmSFKDAER | 1.7892 | TBI+CM | TBI 35 |
| SYNE2 | KcLKMLDmSFKDAER | 1.7892 | TBI+CM | TBI 35 |
| SYNE2 | KcLKMLDmSFKDAER | 1.7892 | TBI+CM | TBI 35 |
| SYNE2 | KcLKMLDmSFKDAER | 1.7892 | TBI+CM | TBI 35 |
| FRAS1 | FCmCDHGQVTcQT | 1.7462 | TBI+CM | TBI 35 |
| FRAS1 | FCmCDHGQVTcQT | 1.7462 | TBI+CM | TBI 35 |
| FRAS1 | FCmCDHGQVTcQT | 1.7462 | TBI+CM | TBI 35 |
| FRAS1 | FCmCDHGQVTcQT | 1.7462 | TBI+CM | TBI 35 |
| FRAS1 | FCmCDHGQVTcQT | 1.7462 | TBI+CM | TBI 35 |
| FRAS1 | FCmCDHGQVTcQT | 1.7462 | TBI+CM | TBI 35 |
| FRAS1 | FCmCDHGQVTcQT | 1.7462 | TBI+CM | TBI 35 |
| FRAS1 | FCmCDHGQVTcQT | 1.7462 | TBI+CM | TBI 35 |
| FRAS1 | FCmCDHGQVTcQT | 1.7462 | TBI+CM | TBI 35 |
| FRAS1 | FCmCDHGQVTcQT | 1.7462 | TBI+CM | TBI 35 |
| FRAS1 | FCmCDHGQVTcQT | 1.7462 | TBI+CM | TBI 35 |
| MT-ND1 | AFLmLTERKILAYMQL | 1.7347 | TBI+CM | TBI 35 |
| MT-ND1 | AFLmLTERKILAYMQL | 1.7347 | TBI+CM | TBI 35 |
| MT-ND1 | AFLmLTERKILAYMQL | 1.7347 | TBI+CM | TBI 35 |
| PCLO | IIKHISAPEKtYKGG | 3.8774 | TBI+CM | TBI 39 |
| PCLO | PAPsGPKASPmPVPTES | 3.8774 | TBI+CM | TBI 39 |
| PCLO | IIKHISAPEKtYKGG | 3.8774 | TBI+CM | TBI 39 |
| PCLO | PAPsGPKASPmPVPTES | 3.8774 | TBI+CM | TBI 39 |
| PCLO | IIKHISAPEKtYKGG | 3.8774 | TBI+CM | TBI 39 |
| PCLO | PAPsGPKASPmPVPTES | 3.8774 | TBI+CM | TBI 39 |
| PCLO | IIKHISAPEKtYKGG | 3.8774 | TBI+CM | TBI 39 |
| PCLO | PAPsGPKASPmPVPTES | 3.8774 | TBI+CM | TBI 39 |
| PCLO | IIKHISAPEKtYKGG | 3.8774 | TBI+CM | TBI 39 |
| PCLO | PAPsGPKASPmPVPTES | 3.8774 | TBI+CM | TBI 39 |
| PCLO | IIKHISAPEKtYKGG | 3.8774 | TBI+CM | TBI 39 |
| PCLO | PAPsGPKASPmPVPTES | 3.8774 | TBI+CM | TBI 39 |
| PCLO | PAPsGPKASPmPVPTES | 2.2895 | TBI+CM | TBI 39 |
| PCLO | PAPsGPKASPmPVPTES | 2.2895 | TBI+CM | TBI 39 |
| PCLO | PAPsGPKASPmPVPTES | 2.2895 | TBI+CM | TBI 39 |
| PCLO | PAPsGPKASPmPVPTES | 2.2895 | TBI+CM | TBI 39 |
| PCLO | PAPsGPKASPmPVPTES | 2.2895 | TBI+CM | TBI 39 |
| PCLO | PAPsGPKASPmPVPTES | 2.2895 | TBI+CM | TBI 39 |
| PCLO | PAPsGPKASPmPVPTES | 2.2895 | TBI+CM | TBI 39 |
| PCLO | PAPsGPKASPmPVPTES | 2.2895 | TBI+CM | TBI 39 |
| DUOX1 | VIATYQNIAVYEWLPs | 2.0971 | TBI+CM | TBI 39 |
| DUOX1 | VIATYQNIAVYEWLPs | 2.0971 | TBI+CM | TBI 39 |
| DUOX1 | VIATYQNIAVYEWLPs | 2.0971 | TBI+CM | TBI 39 |
| DUOX1 | VIATYQNIAVYEWLPs | 2.0971 | TBI+CM | TBI 39 |
| DUOX1 | VIATYQNIAVYEWLPs | 2.0971 | TBI+CM | TBI 39 |
| TENM1 | NEmDAVsESLSFGERGc | 2.0593 | TBI+CM | TBI 39 |
| TENM1 | NEmDAVsESLSFGERGc | 2.0593 | TBI+CM | TBI 39 |
| TENM1 | NEmDAVsESLSFGERGc | 2.0593 | TBI+CM | TBI 39 |
| IGH | VSGFTsSNDWISWVRQ | 1.9519 | TBI+CM | TBI 39 |
| IGH | VSGFTsSNDWISWVRQ | 1.9519 | TBI+CM | TBI 39 |
| TNFRSF14 | PLGQPGCPRtLHPLSMAT | 1.9405 | TBI+CM | TBI 39 |
| TNFRSF14 | PLGQPGCPRtLHPLSMAT | 1.9405 | TBI+CM | TBI 39 |
| MYO15A | FIGFLQEEKyG | 1.8733 | TBI+CM | TBI 39 |
| MYO15A | FIGFLQEEKyG | 1.8733 | TBI+CM | TBI 39 |
| PLOD1 | PVLIHGNGPtKLQLNYL | 1.837 | TBI+CM | TBI 39 |
| PLOD1 | PVLIHGNGPtKLQLNYL | 1.837 | TBI+CM | TBI 39 |
| PLOD1 | PVLIHGNGPtKLQLNYL | 1.837 | TBI+CM | TBI 39 |
| PLOD1 | PVLIHGNGPtKLQLNYL | 1.837 | TBI+CM | TBI 39 |
| PLOD1 | PVLIHGNGPtKLQLNYL | 1.837 | TBI+CM | TBI 39 |
| CCDC148 | IELEsLEcPYPDL | 1.7944 | TBI+CM | TBI 39 |
| CCDC148 | IELEsLEcPYPDL | 1.7944 | TBI+CM | TBI 39 |
| CCDC148 | IELEsLEcPYPDL | 1.7944 | TBI+CM | TBI 39 |
| CCDC148 | IELEsLEcPYPDL | 1.7944 | TBI+CM | TBI 39 |
| CCDC148 | IELEsLEcPYPDL | 1.7944 | TBI+CM | TBI 39 |
| CCDC148 | IELEsLEcPYPDL | 1.7944 | TBI+CM | TBI 39 |
| CCDC148 | IELEsLEcPYPDL | 1.7944 | TBI+CM | TBI 39 |
| CCDC148 | IELEsLEcPYPDL | 1.7944 | TBI+CM | TBI 39 |
| CCDC148 | IELEsLEcPYPDL | 1.7944 | TBI+CM | TBI 39 |
| CCDC148 | IELEsLEcPYPDL | 1.7944 | TBI+CM | TBI 39 |
| CCDC148 | IELEsLEcPYPDL | 1.7944 | TBI+CM | TBI 39 |
| CCDC148 | IELEsLEcPYPDL | 1.7944 | TBI+CM | TBI 39 |
| CCDC148 | IELEsLEcPYPDL | 1.7944 | TBI+CM | TBI 39 |
| CCDC148 | IELEsLEcPYPDL | 1.7944 | TBI+CM | TBI 39 |
| CCDC148 | IELEsLEcPYPDL | 1.7944 | TBI+CM | TBI 39 |
| MSI2 | CmVmRDPTTKRs | 1.7539 | TBI+CM | TBI 39 |
| MSI2 | CmVmRDPTTKRs | 1.7539 | TBI+CM | TBI 39 |
| MSI2 | CmVmRDPTTKRs | 1.7539 | TBI+CM | TBI 39 |
| MSI2 | CmVmRDPTTKRs | 1.7539 | TBI+CM | TBI 39 |
| MSI2 | CmVmRDPTTKRs | 1.7539 | TBI+CM | TBI 39 |
| MSI2 | CmVmRDPTTKRs | 1.7539 | TBI+CM | TBI 39 |
| MSI2 | CmVmRDPTTKRs | 1.7539 | TBI+CM | TBI 39 |
| MSI2 | CmVmRDPTTKRs | 1.7539 | TBI+CM | TBI 39 |
| MSI2 | CmVmRDPTTKRs | 1.7539 | TBI+CM | TBI 39 |
| MSI2 | CmVmRDPTTKRs | 1.7539 | TBI+CM | TBI 39 |
| MSI2 | CmVmRDPTTKRs | 1.7539 | TBI+CM | TBI 39 |
| MSI2 | CmVmRDPTTKRs | 1.7539 | TBI+CM | TBI 39 |
| MSI2 | CmVmRDPTtKRS | 1.7539 | TBI+CM | TBI 39 |
| MSI2 | CmVmRDPTTKRs | 1.7539 | TBI+CM | TBI 39 |
| MSI2 | CmVmRDPTtKRS | 1.7539 | TBI+CM | TBI 39 |
| MSI2 | CmVmRDPTtKRS | 1.7539 | TBI+CM | TBI 39 |
| MSI2 | CmVmRDPTtKRS | 1.7539 | TBI+CM | TBI 39 |
| MSI2 | CmVmRDPTtKRS | 1.7539 | TBI+CM | TBI 39 |
| MSI2 | CmVmRDPTtKRS | 1.7539 | TBI+CM | TBI 39 |
| MSI2 | CmVmRDPTtKRS | 1.7539 | TBI+CM | TBI 39 |
| MSI2 | CmVmRDPTtKRS | 1.7539 | TBI+CM | TBI 39 |
| MSI2 | CmVmRDPTTKRs | 1.7539 | TBI+CM | TBI 39 |
| MSI2 | CmVmRDPTtKRS | 1.7539 | TBI+CM | TBI 39 |
| MSI2 | CmVmRDPTtKRS | 1.7539 | TBI+CM | TBI 39 |
| MSI2 | CmVmRDPTtKRS | 1.7539 | TBI+CM | TBI 39 |
| MSI2 | CmVmRDPTtKRS | 1.7539 | TBI+CM | TBI 39 |
| MSI2 | CmVmRDPTtKRS | 1.7539 | TBI+CM | TBI 39 |
| MSI2 | CmVmRDPTtKRS | 1.7539 | TBI+CM | TBI 39 |
| MSI2 | CmVmRDPTtKRS | 1.7539 | TBI+CM | TBI 39 |
| MSI2 | CmVmRDPTtKRS | 1.7539 | TBI+CM | TBI 39 |
| MSI2 | CmVmRDPTTKRs | 1.7539 | TBI+CM | TBI 39 |
| MSI2 | CmVmRDPTtKRS | 1.7539 | TBI+CM | TBI 39 |
| MSI2 | CmVmRDPTtKRS | 1.7539 | TBI+CM | TBI 39 |
| PRAG1 | DcCPGSPVAKAAsQTAGS | 1.7469 | TBI+CM | TBI 39 |
| PRAG1 | DcCPGSPVAKAAsQTAGS | 1.7469 | TBI+CM | TBI 39 |
| PRAG1 | DcCPGSPVAKAAsQTAGS | 1.7469 | TBI+CM | TBI 39 |
| PRAG1 | DcCPGSPVAKAAsQTAGS | 1.7469 | TBI+CM | TBI 39 |
| PRAG1 | DcCPGSPVAKAAsQTAGS | 1.7469 | TBI+CM | TBI 39 |
| PRAG1 | DcCPGSPVAKAAsQTAGS | 1.7469 | TBI+CM | TBI 39 |
| PRAG1 | DcCPGSPVAKAAsQTAGS | 1.7469 | TBI+CM | TBI 39 |
| PRAG1 | DcCPGSPVAKAAsQTAGS | 1.7469 | TBI+CM | TBI 39 |
| PRAG1 | DcCPGSPVAKAAsQTAGS | 1.7469 | TBI+CM | TBI 39 |
| ZNF571 | YECKEcGKAFI | 4.3334 | TBI+CM | TBI 40 |
| ZNF571 | YECKEcGKAFI | 4.3334 | TBI+CM | TBI 40 |
| ZNF571 | YECKEcGKAFI | 4.3334 | TBI+CM | TBI 40 |
| ZNF571 | YECKEcGKAFI | 4.3334 | TBI+CM | TBI 40 |
| ZNF571 | YECKEcGKAFI | 4.3334 | TBI+CM | TBI 40 |
| ZNF571 | YECKEcGKAFI | 4.3334 | TBI+CM | TBI 40 |
| ZNF571 | YECKEcGKAFI | 4.3334 | TBI+CM | TBI 40 |
| ZNF571 | YECKEcGKAFI | 4.3334 | TBI+CM | TBI 40 |
| ZNF571 | YECKEcGKAFI | 4.3334 | TBI+CM | TBI 40 |
| ZNF571 | FEcKEcGKAFI | 4.3334 | TBI+CM | TBI 40 |
| ZNF571 | YECKEcGKAFI | 4.3334 | TBI+CM | TBI 40 |
| ZNF571 | YIcKEcGKAFL | 4.3334 | TBI+CM | TBI 40 |
| ZNF571 | YECKEcGKAFI | 4.3334 | TBI+CM | TBI 40 |
| ZNF571 | YECKEcGKAFI | 4.3334 | TBI+CM | TBI 40 |
| ZNF571 | YECKEcGKAFI | 4.3334 | TBI+CM | TBI 40 |
| TTN | HKGSIKETHymV | 3.3197 | TBI+CM | TBI 40 |
| TTN | HKGSIKETHymV | 3.3197 | TBI+CM | TBI 40 |
| TTN | HKGSIKETHymV | 3.3197 | TBI+CM | TBI 40 |
| TTN | HKGSIKETHymV | 3.3197 | TBI+CM | TBI 40 |
| TTN | YtCQATNDVGKDM | 3.3197 | TBI+CM | TBI 40 |
| TTN | yTCQATNDVGKDM | 3.3197 | TBI+CM | TBI 40 |
| FRAS1 | GQccPDctS | 2.8885 | TBI+CM | TBI 40 |
| FRAS1 | GQccPDctS | 2.8885 | TBI+CM | TBI 40 |
| FRAS1 | GQccPDctS | 2.8885 | TBI+CM | TBI 40 |
| FRAS1 | GQccPDctS | 2.8885 | TBI+CM | TBI 40 |
| FRAS1 | GQccPDctS | 2.8885 | TBI+CM | TBI 40 |
| FRAS1 | GQccPDctS | 2.8885 | TBI+CM | TBI 40 |
| FRAS1 | GQccPDctS | 2.8885 | TBI+CM | TBI 40 |
| FRAS1 | GQccPDctS | 2.8885 | TBI+CM | TBI 40 |
| FRAS1 | GQccPDctS | 2.8885 | TBI+CM | TBI 40 |
| FRAS1 | CNTHcGSCDSQASCTScR | 2.8885 | TBI+CM | TBI 40 |
| FRAS1 | GQccPDcTs | 2.8885 | TBI+CM | TBI 40 |
| FRAS1 | GQccPDctS | 2.8885 | TBI+CM | TBI 40 |
| PRAG1 | ADLSDGScGGSSIGPQPPSQG | 2.6953 | TBI+CM | TBI 40 |
| PRAG1 | ADLSDGScGGSSIGPQPPSQG | 2.6953 | TBI+CM | TBI 40 |
| PRAG1 | ADLSDGScGGSSIGPQPPSQG | 2.6953 | TBI+CM | TBI 40 |
| ZNF91 | YKCEEcGKAFI | 2.6287 | TBI+CM | TBI 40 |
| ZNF91 | YKCEEcGKAFI | 2.6287 | TBI+CM | TBI 40 |
| ZNF91 | YKCEEcGKAFI | 2.6287 | TBI+CM | TBI 40 |
| ZNF91 | YKCEEcGKAFI | 2.6287 | TBI+CM | TBI 40 |
| ZNF91 | YKCEEcGKAFI | 2.6287 | TBI+CM | TBI 40 |
| ZNF91 | YKCEEcGKAFI | 2.6287 | TBI+CM | TBI 40 |
| ZNF91 | YKCEEcGKAFI | 2.6287 | TBI+CM | TBI 40 |
| ZNF91 | YKCEEcGKAFL | 2.6287 | TBI+CM | TBI 40 |
| ZNF91 | YKCEEcGKAFI | 2.6287 | TBI+CM | TBI 40 |
| ZNF91 | YKCEEcGKAFI | 2.6287 | TBI+CM | TBI 40 |
| DUOX1 | tPCScGRRPTPPPGRTGP | 2.366 | TBI+CM | TBI 40 |
| DUOX1 | tPCScGRRPTPPPGRTGP | 2.366 | TBI+CM | TBI 40 |
| DUOX1 | tPcSCGRRPTPPPGRTGP | 2.366 | TBI+CM | TBI 40 |
| DUOX1 | tPcSCGRRPTPPPGRTGP | 2.366 | TBI+CM | TBI 40 |
| DUOX1 | tPcSCGRRPTPPPGRTGP | 2.366 | TBI+CM | TBI 40 |
| DUOX1 | tPCScGRRPTPPPGRTGP | 2.366 | TBI+CM | TBI 40 |
| DUOX1 | tPcSCGRRPTPPPGRTGP | 2.366 | TBI+CM | TBI 40 |
| DUOX1 | tPCScGRRPTPPPGRTGP | 2.2142 | TBI+CM | TBI 40 |
| DUOX1 | tPCScGRRPTPPPGRTGP | 2.2142 | TBI+CM | TBI 40 |
| DUOX1 | tPCScGRRPTPPPGRTGP | 2.2142 | TBI+CM | TBI 40 |
| RXFP1 | VKCsLGYFPcGNITKcL | 2.1295 | TBI+CM | TBI 40 |
| RXFP1 | VKCsLGYFPcGNITKcL | 2.1295 | TBI+CM | TBI 40 |
| RXFP1 | VKCsLGYFPcGNITKcL | 2.1295 | TBI+CM | TBI 40 |
| RXFP1 | VKCsLGYFPcGNITKcL | 2.1295 | TBI+CM | TBI 40 |
| RXFP1 | VKCsLGYFPcGNITKcL | 2.1295 | TBI+CM | TBI 40 |
| RXFP1 | VKCsLGYFPcGNITKcL | 2.1295 | TBI+CM | TBI 40 |
| RXFP1 | VKCsLGYFPcGNITKcL | 2.1295 | TBI+CM | TBI 40 |
| RXFP1 | VKCsLGYFPcGNITKcL | 2.1295 | TBI+CM | TBI 40 |
| VWDE | cKcKPGYIGSNcQTALCD | 2.1005 | TBI+CM | TBI 40 |
| VWDE | cKcKPGYIGSNcQTALCD | 2.1005 | TBI+CM | TBI 40 |
| VWDE | cKcKPGYIGSNcQTALCD | 2.1005 | TBI+CM | TBI 40 |
| VWDE | cKcKPGYIGSNcQTALCD | 2.1005 | TBI+CM | TBI 40 |
| VWDE | cKcKPGYIGSNcQTALCD | 2.1005 | TBI+CM | TBI 40 |
| SLC39A14 | SGcLLcCtDWSAVVPG | 1.9757 | TBI+CM | TBI 40 |
| SLC39A14 | SGcLLCctDWSAVVPG | 1.9757 | TBI+CM | TBI 40 |
| SLC39A14 | SGcLLcCtDWSAVVPG | 1.9757 | TBI+CM | TBI 40 |
| SLC39A14 | SGcLLCctDWSAVVPG | 1.9757 | TBI+CM | TBI 40 |
| SLC39A14 | SGcLLcCtDWSAVVPG | 1.9757 | TBI+CM | TBI 40 |
| SLC39A14 | SGcLLcCtDWSAVVPG | 1.9757 | TBI+CM | TBI 40 |
| SLC39A14 | SGcLLCctDWSAVVPG | 1.9757 | TBI+CM | TBI 40 |
| SLC39A14 | SGcLLcCtDWSAVVPG | 1.9757 | TBI+CM | TBI 40 |
| SLC39A14 | SGcLLcCtDWSAVVPG | 1.9757 | TBI+CM | TBI 40 |
| SLC39A14 | SGcLLcCtDWSAVVPG | 1.9757 | TBI+CM | TBI 40 |
| IQCN | TSRMSPSRAHAsmTCMV | 1.9369 | TBI+CM | TBI 40 |
| IQCN | TSRMSPSRAHAsmTCMV | 1.9369 | TBI+CM | TBI 40 |
| IQCN | TSRMSPSRAHAsmTCMV | 1.9369 | TBI+CM | TBI 40 |
| TTN | YtCQATNDVGKDM | 1.8679 | TBI+CM | TBI 40 |
| TTN | YtCQATNDVGKDM | 1.8679 | TBI+CM | TBI 40 |
| TTN | YtCQATNDVGKDM | 1.8679 | TBI+CM | TBI 40 |
| TTN | YtCQATNDVGKDM | 1.8679 | TBI+CM | TBI 40 |
| TTN | YtCQATNDVGKDM | 1.8679 | TBI+CM | TBI 40 |
| TTN | YtCQATNDVGKDM | 1.8679 | TBI+CM | TBI 40 |
| SLC39A14 | SGcLLCctDWSAVVPG | 1.8215 | TBI+CM | TBI 40 |
| SLC39A14 | SGcLLCctDWSAVVPG | 1.8215 | TBI+CM | TBI 40 |
| SLC39A14 | SGcLLCctDWSAVVPG | 1.8215 | TBI+CM | TBI 40 |
| SLC39A14 | SGcLLCctDWSAVVPG | 1.8215 | TBI+CM | TBI 40 |
| TNRC6B | KRVAVPNGQPPSAARYmP | 1.7957 | TBI+CM | TBI 40 |
| TNRC6B | KRVAVPNGQPPSAARYmP | 1.7957 | TBI+CM | TBI 40 |
| TNRC6B | KRVAVPNGQPPSAARYmP | 1.7957 | TBI+CM | TBI 40 |
| TNRC6B | KRVAVPNGQPPSAARYmP | 1.7957 | TBI+CM | TBI 40 |
| TNRC6B | KRVAVPNGQPPSAARYmP | 1.7957 | TBI+CM | TBI 40 |
| TNRC6B | KRVAVPNGQPPSAARYmP | 1.7957 | TBI+CM | TBI 40 |
| TNRC6B | KRVAVPNGQPPSAARYmP | 1.7957 | TBI+CM | TBI 40 |
| TNRC6B | KRVAVPNGQPPSAARYmP | 1.7957 | TBI+CM | TBI 40 |
| TNRC6B | KRVAVPNGQPPSAARYmP | 1.7957 | TBI+CM | TBI 40 |
| TNRC6B | KRVAVPNGQPPSAARYmP | 1.7957 | TBI+CM | TBI 40 |
| TNRC6B | KRVAVPNGQPPSAARYmP | 1.7957 | TBI+CM | TBI 40 |
| ZFHX3 | SPGSLLQQYQQyQQSL | 2.3417 | TBI | TBI 41 |
| ZFHX3 | SPGSLLQQYQQyQQSL | 2.3417 | TBI | TBI 41 |
| ZFHX3 | SPGSLLQQYQQyQQSL | 2.3417 | TBI | TBI 41 |
| ZFHX3 | SPGSLLQQYQQyQQSL | 2.3417 | TBI | TBI 41 |
| ZFHX3 | SPGSLLQQYQQyQQSL | 2.3417 | TBI | TBI 41 |
| TANC1 | GISPCSTLtSSTASPSTDS | 2.1608 | TBI | TBI 41 |
| TANC1 | GISPCSTLtSSTASPSTDS | 2.1608 | TBI | TBI 41 |
| TANC1 | GISPCSTLtSSTASPSTDS | 2.1608 | TBI | TBI 41 |
| TANC1 | GISPCSTLtSSTASPSTDS | 2.1608 | TBI | TBI 41 |
| TANC1 | GISPCSTLtSSTASPSTDS | 2.1608 | TBI | TBI 41 |
| TANC1 | GISPCSTLtSSTASPSTDS | 2.1608 | TBI | TBI 41 |
| WDHD1 | QISDQTcAISWPLLQKC | 2.0941 | TBI | TBI 41 |
| WDHD1 | QISDQTcAISWPLLQKC | 2.0941 | TBI | TBI 41 |
| WDHD1 | QISDQTcAISWPLLQKC | 2.0941 | TBI | TBI 41 |
| WDHD1 | QISDQTcAISWPLLQKC | 2.0941 | TBI | TBI 41 |
| WDHD1 | QISDQTcAISWPLLQKC | 2.0941 | TBI | TBI 41 |
| LSM12 | MEEVVItPPYQVENCK | 2.0408 | TBI | TBI 41 |
| LSM12 | MEEVVItPPYQVENCK | 2.0408 | TBI | TBI 41 |
| LSM12 | MEEVVItPPYQVENCK | 2.0408 | TBI | TBI 41 |
| IGH | mCLKASDTWASGIRsQP | 1.9382 | TBI | TBI 41 |
| IGH | mCLKASDTWASGIRsQP | 1.9382 | TBI | TBI 41 |
| IGH | McLKASDTWASGIRsQP | 1.9382 | TBI | TBI 41 |
| IGH | McLKASDTWASGIRsQP | 1.9357 | TBI | TBI 41 |
| PCDH8 | mQsGLWACTAECKILGH | 1.9068 | TBI | TBI 41 |
| PCDH8 | mQsGLWACTAECKILGH | 1.9068 | TBI | TBI 41 |
| PCDH8 | mQsGLWACTAECKILGH | 1.9068 | TBI | TBI 41 |
| PCDH8 | mQsGLWACTAECKILGH | 1.9068 | TBI | TBI 41 |
| PCDH8 | mQsGLWACTAECKILGH | 1.9068 | TBI | TBI 41 |
| PCDH8 | mQsGLWACTAECKILGH | 1.9068 | TBI | TBI 41 |
| HUWE1 | KEVQTPKWItPVLLLI | 1.847 | TBI | TBI 41 |
| HUWE1 | KEVQTPKWItPVLLLI | 1.847 | TBI | TBI 41 |
| HUWE1 | KEVQTPKWItPVLLLI | 1.847 | TBI | TBI 41 |
| HUWE1 | KEVQTPKWItPVLLLI | 1.847 | TBI | TBI 41 |
| HUWE1 | KEVQTPKWItPVLLLI | 1.847 | TBI | TBI 41 |
| HUWE1 | KEVQTPKWItPVLLLI | 1.847 | TBI | TBI 41 |
| HUWE1 | KEVQTPKWItPVLLLI | 1.847 | TBI | TBI 41 |
| HUWE1 | KEVQTPKWItPVLLLI | 1.847 | TBI | TBI 41 |
| HUWE1 | KEVQTPKWItPVLLLI | 1.847 | TBI | TBI 41 |
| TTN | GTDHTsAtLIVKDEKS | 1.7747 | TBI | TBI 41 |
| TTN | GTDHTsAtLIVKDEKS | 1.7747 | TBI | TBI 41 |
| TTN | GTDHTsAtLIVKDEKS | 1.7747 | TBI | TBI 41 |
| TTN | GTDHTsAtLIVKDEKS | 1.7747 | TBI | TBI 41 |
| TTN | GTDHTsAtLIVKDEKS | 1.7747 | TBI | TBI 41 |
| TTN | GTDHTsAtLIVKDEKS | 1.7747 | TBI | TBI 41 |
| TTN | GTDHTsAtLIVKDEKS | 1.7747 | TBI | TBI 41 |
| TTN | GTDHTsAtLIVKDEKS | 1.7747 | TBI | TBI 41 |
| TTN | GTDHTsAtLIVKDEKS | 1.7747 | TBI | TBI 41 |
| TTN | GTDHTsAtLIVKDEKS | 1.7747 | TBI | TBI 41 |
| TTN | GTDHTsAtLIVKDEKS | 1.7747 | TBI | TBI 41 |
| TTN | GTDHTsAtLIVKDEKS | 1.7747 | TBI | TBI 41 |
| TTN | GTDHTsAtLIVKDEKS | 1.7747 | TBI | TBI 41 |
| TNRC6B | PPGLTNPKPSsPWSSTA | 1.7481 | TBI | TBI 41 |
| TNRC6B | PPGLTNPKPSsPWSSTA | 1.7481 | TBI | TBI 41 |
| TNRC6B | PPGLTNPKPSsPWSSTA | 1.7481 | TBI | TBI 41 |
| TNRC6B | PPGLTNPKPSsPWSSTA | 1.7481 | TBI | TBI 41 |
| TNRC6B | PPGLTNPKPSsPWSSTA | 1.7481 | TBI | TBI 41 |
| TNRC6B | PPGLTNPKPSsPWSSTA | 1.7481 | TBI | TBI 41 |
| TNRC6B | PPGLTNPKPSsPWSSTA | 1.7481 | TBI | TBI 41 |
| TNRC6B | PPGLTNPKPSsPWSSTA | 1.7481 | TBI | TBI 41 |
| TNRC6B | PPGLTNPKPSsPWSSTA | 1.7481 | TBI | TBI 41 |
| TNRC6B | PPGLTNPKPSsPWSSTA | 1.7481 | TBI | TBI 41 |
| TNRC6B | PPGLTNPKPsSPWSSTA | 1.7481 | TBI | TBI 41 |
| TNRC6B | PPGLTNPKPsSPWSSTA | 1.7481 | TBI | TBI 41 |
| TNRC6B | PPGLTNPKPsSPWSSTA | 1.7481 | TBI | TBI 41 |
| TNRC6B | PPGLTNPKPsSPWSSTA | 1.7481 | TBI | TBI 41 |
| TNRC6B | PPGLTNPKPsSPWSSTA | 1.7481 | TBI | TBI 41 |
| TNRC6B | PPGLTNPKPsSPWSSTA | 1.7481 | TBI | TBI 41 |
| TNRC6B | PPGLTNPKPsSPWSSTA | 1.7481 | TBI | TBI 41 |
| TNRC6B | PPGLTNPKPsSPWSSTA | 1.7481 | TBI | TBI 41 |
| TNRC6B | PPGLTNPKPSsPWSSTA | 1.7481 | TBI | TBI 41 |
| TNRC6B | PPGLTNPKPsSPWSSTA | 1.7481 | TBI | TBI 41 |
| TNRC6B | PPGLTNPKPsSPWSSTA | 1.7481 | TBI | TBI 41 |
| TNRC6B | PPGLTNPKPsSPWSSTA | 1.7481 | TBI | TBI 41 |
| TNRC6B | PPGLTNPKPsSPWSSTA | 1.7481 | TBI | TBI 41 |
| TNRC6B | PPGLTNPKPsSPWSSTA | 1.7481 | TBI | TBI 41 |
| TNRC6B | PPGLTNPKPsSPWSSTA | 1.7481 | TBI | TBI 41 |
| ZNF646 | AGDcQLNGPTLSHmDsW | 2.4338 | TBI | TBI 42 |
| ZNF646 | AGDcQLNGPTLSHmDsW | 2.4338 | TBI | TBI 42 |
| ZNF646 | AGDcQLNGPTLSHmDsW | 2.4338 | TBI | TBI 42 |
| ZNF646 | AGDcQLNGPTLSHmDsW | 2.4338 | TBI | TBI 42 |
| ZNF646 | AGDcQLNGPTLSHmDsW | 2.4338 | TBI | TBI 42 |
| SVEP1 | PVLIcQEDGTRNGSAPsC | 2.057 | TBI | TBI 42 |
| SVEP1 | PVLIcQEDGTRNGSAPsC | 2.057 | TBI | TBI 42 |
| SVEP1 | PVLIcQEDGTRNGSAPsC | 2.057 | TBI | TBI 42 |
| IGH | QSGGGVFKPGGSLRLscEA | 2.0388 | TBI | TBI 42 |
| IGH | QSGGGVFKPGGSLRLscEA | 2.0388 | TBI | TBI 42 |
| IGH | QSGGGVFKPGGSLRLscEA | 2.0388 | TBI | TBI 42 |
| ITGB3 | CEKCPtCPDACTFKKE | 1.7964 | TBI | TBI 42 |
| ITGB3 | CEKCPtCPDACTFKKE | 1.7964 | TBI | TBI 42 |
| ITGB3 | CEKCPtCPDACTFKKE | 1.7964 | TBI | TBI 42 |
| ITGB3 | CEKCPtCPDACTFKKE | 1.7964 | TBI | TBI 42 |
| ITGB3 | CEKCPtCPDACTFKKE | 1.7964 | TBI | TBI 42 |
| ITGB3 | CEKCPtCPDACTFKKE | 1.7964 | TBI | TBI 42 |
| ITGB3 | CEKCPtCPDACTFKKE | 1.7964 | TBI | TBI 42 |
| ITGB3 | CEKCPtCPDACTFKKE | 1.7964 | TBI | TBI 42 |
| ITGB3 | CEKCPtCPDACTFKKE | 1.7964 | TBI | TBI 42 |
| ITGB3 | CEKCPtCPDACTFKKE | 1.7964 | TBI | TBI 42 |
| ITGB3 | CEKCPtCPDACTFKKE | 1.7964 | TBI | TBI 42 |
| MSI2 | EQFGKAPPSsTGFLNSH | 2.1676 | TBI+CM | TBI 43 |
| MSI2 | EQFGKAPPSsTGFLNSH | 2.1676 | TBI+CM | TBI 43 |
| MSI2 | EQFGKAPPSsTGFLNSH | 2.1676 | TBI+CM | TBI 43 |
| MSI2 | EQFGKAPPSStGFLNSH | 2.1676 | TBI+CM | TBI 43 |
| MSI2 | EQFGKAPPSsTGFLNSH | 2.1676 | TBI+CM | TBI 43 |
| MSI2 | EQFGKAPPSStGFLNSH | 2.0774 | TBI+CM | TBI 43 |
| MSI2 | EQFGKAPPSStGFLNSH | 2.0774 | TBI+CM | TBI 43 |
| MSI2 | EQFGKAPPSStGFLNSH | 2.0774 | TBI+CM | TBI 43 |
| PXN | APKCGGcARAILENYIs | 2.0658 | TBI+CM | TBI 43 |
| PXN | APKcGGCARAILENYIs | 2.0658 | TBI+CM | TBI 43 |
| PXN | APKCGGcARAILENYIs | 2.0658 | TBI+CM | TBI 43 |
| PXN | APKcGGCARAILENYIs | 2.0658 | TBI+CM | TBI 43 |
| PXN | APKCGGcARAILENYIs | 2.0658 | TBI+CM | TBI 43 |
| PXN | APKcGGCARAILENYIs | 2.0658 | TBI+CM | TBI 43 |
| PXN | APKCGGcARAILENYIs | 2.0658 | TBI+CM | TBI 43 |
| PXN | APKcGGCARAILENYIs | 2.0658 | TBI+CM | TBI 43 |
| PXN | APKCGGcARAILENYIs | 2.0658 | TBI+CM | TBI 43 |
| PXN | APKcGGCARAILENYIs | 2.0658 | TBI+CM | TBI 43 |
| PXN | APKcGGCARAILENYIs | 2.0658 | TBI+CM | TBI 43 |
| PXN | APKcGGCARAILENYIs | 2.0658 | TBI+CM | TBI 43 |
| PXN | APKCGGcARAILENYIs | 2.0658 | TBI+CM | TBI 43 |
| PXN | APKcGGCARAILENYIs | 2.0658 | TBI+CM | TBI 43 |
| PXN | APKCGGcARAILENYIs | 2.0658 | TBI+CM | TBI 43 |
| PXN | APKCGGcARAILENYIs | 2.0658 | TBI+CM | TBI 43 |
| PXN | APKCGGcARAILENYIs | 2.0658 | TBI+CM | TBI 43 |
| PXN | APKcGGCARAILENYIs | 2.0658 | TBI+CM | TBI 43 |
| PXN | APKCGGcARAILENYIs | 2.0658 | TBI+CM | TBI 43 |
| PXN | APKCGGcARAILENYIs | 2.0658 | TBI+CM | TBI 43 |
| PXN | APKCGGcARAILENYIs | 2.0658 | TBI+CM | TBI 43 |
| PXN | APKCGGcARAILENYIs | 2.0658 | TBI+CM | TBI 43 |
| PXN | APKCGGcARAILENYIs | 2.0658 | TBI+CM | TBI 43 |
| PXN | APKCGGcARAILENYIs | 2.0658 | TBI+CM | TBI 43 |
| PXN | APKCGGcARAILENYIs | 2.0658 | TBI+CM | TBI 43 |
| PXN | APKCGGcARAILENYIs | 2.0658 | TBI+CM | TBI 43 |
| RXFP1 | IVsHGGGQDVKcSLGYF | 1.9982 | TBI+CM | TBI 43 |
| RXFP1 | IVsHGGGQDVKcSLGYF | 1.9982 | TBI+CM | TBI 43 |
| PAPPA | AIGSEcATScLDHNSEsI | 1.9714 | TBI+CM | TBI 43 |
| PAPPA | AIGSEcATScLDHNSEsI | 1.9714 | TBI+CM | TBI 43 |
| PAPPA | AIGSEcATScLDHNSEsI | 1.9714 | TBI+CM | TBI 43 |
| PAPPA | AIGSEcATScLDHNSEsI | 1.9714 | TBI+CM | TBI 43 |
| PAPPA | AIGSEcATScLDHNSEsI | 1.9714 | TBI+CM | TBI 43 |
| PAPPA | AIGSEcATScLDHNSEsI | 1.9714 | TBI+CM | TBI 43 |
| PAPPA | AIGSEcATScLDHNSEsI | 1.9714 | TBI+CM | TBI 43 |
| RNF219 | PGRMcYTccSsSYALD | 1.9568 | TBI+CM | TBI 43 |
| RNF219 | PGRMcYTccSsSYALD | 1.9568 | TBI+CM | TBI 43 |
| RNF219 | PGRMcYTccsSSYALD | 1.9568 | TBI+CM | TBI 43 |
| RNF219 | PGRMcYTccSsSYALD | 1.9568 | TBI+CM | TBI 43 |
| RNF219 | PGRMcYTccSSsYALD | 1.9568 | TBI+CM | TBI 43 |
| RNF219 | PGRMcYTccSsSYALD | 1.9568 | TBI+CM | TBI 43 |
| RNF219 | PGRMcYTccSSSyALD | 1.9568 | TBI+CM | TBI 43 |
| RNF219 | PGRMcYTccSsSYALD | 1.9568 | TBI+CM | TBI 43 |
| RNF219 | PGRMcYTccsSSYALD | 1.9512 | TBI+CM | TBI 43 |
| RNF219 | PGRMcYTccsSSYALD | 1.9512 | TBI+CM | TBI 43 |
| RNF219 | PGRMcYTccsSSYALD | 1.9512 | TBI+CM | TBI 43 |
| RNF219 | PGRMcYTccsSSYALD | 1.9512 | TBI+CM | TBI 43 |
| RNF219 | PGRMcYTccSSsYALD | 1.9436 | TBI+CM | TBI 43 |
| RNF219 | PGRMcYTccSSsYALD | 1.9436 | TBI+CM | TBI 43 |
| RNF219 | PGRMcYTccSSsYALD | 1.9436 | TBI+CM | TBI 43 |
| RNF219 | PGRMcYTccSSsYALD | 1.9436 | TBI+CM | TBI 43 |
| RNF219 | PGRMcYTccSSSyALD | 1.9361 | TBI+CM | TBI 43 |
| RNF219 | PGRMcYTccSSSyALD | 1.9361 | TBI+CM | TBI 43 |
| RNF219 | PGRMcYTccSSSyALD | 1.9361 | TBI+CM | TBI 43 |
| RNF219 | PGRMcYTccSSSyALD | 1.9361 | TBI+CM | TBI 43 |
| LRP2 | LcHTPEPtcPPHEFKC | 1.9236 | TBI+CM | TBI 43 |
| LRP2 | LcHTPEPtcPPHEFKC | 1.9236 | TBI+CM | TBI 43 |
| LRP2 | LcHTPEPtcPPHEFKC | 1.9236 | TBI+CM | TBI 43 |
| LRP2 | LcHTPEPtcPPHEFKC | 1.9236 | TBI+CM | TBI 43 |
| LRP2 | LcHTPEPtcPPHEFKC | 1.9236 | TBI+CM | TBI 43 |
| PLP1 | GLLEccARcLVGAPFAsL | 1.8813 | TBI+CM | TBI 43 |
| PLP1 | GLLEccARcLVGAPFAsL | 1.8813 | TBI+CM | TBI 43 |
| PLP1 | GLLEccARcLVGAPFAsL | 1.8813 | TBI+CM | TBI 43 |
| PLP1 | GLLEccARcLVGAPFAsL | 1.8813 | TBI+CM | TBI 43 |
| PLP1 | GLLEccARcLVGAPFAsL | 1.8813 | TBI+CM | TBI 43 |
| PLP1 | GLLEccARcLVGAPFAsL | 1.8813 | TBI+CM | TBI 43 |
| PLP1 | GLLEccARcLVGAPFAsL | 1.8813 | TBI+CM | TBI 43 |
| IGL | cDIQmTQSPSsLSASVGG | 1.8782 | TBI+CM | TBI 43 |
| IGL | cDIQmTQSPSsLSASVGG | 1.8782 | TBI+CM | TBI 43 |
| IGL | cDIQmTQSPSsLSASVGG | 1.8782 | TBI+CM | TBI 43 |
| IGL | cDIQmTQSPSsLSASVGG | 1.8782 | TBI+CM | TBI 43 |
| IGL | cDIQmTQSPSsLSASVGG | 1.8782 | TBI+CM | TBI 43 |
| IQCN | PSQAPGVTSNLAQPsQAF | 1.8724 | TBI+CM | TBI 43 |
| IQCN | PSQAPGVTSNLAQPsQAF | 1.8724 | TBI+CM | TBI 43 |
| IQCN | PSQAPGVTSNLAQPsQAF | 1.8724 | TBI+CM | TBI 43 |
| PXN | APKcGGCARAILENYIs | 1.8577 | TBI+CM | TBI 43 |
| PXN | APKcGGCARAILENYIs | 1.8577 | TBI+CM | TBI 43 |
| PXN | APKcGGCARAILENYIs | 1.8577 | TBI+CM | TBI 43 |
| PXN | APKcGGCARAILENYIs | 1.8577 | TBI+CM | TBI 43 |
| PXN | APKcGGCARAILENYIs | 1.8577 | TBI+CM | TBI 43 |
| PXN | APKcGGCARAILENYIs | 1.8577 | TBI+CM | TBI 43 |
| PXN | APKcGGCARAILENYIs | 1.8577 | TBI+CM | TBI 43 |
| PXN | APKcGGCARAILENYIs | 1.8577 | TBI+CM | TBI 43 |
| PXN | APKcGGCARAILENYIs | 1.8577 | TBI+CM | TBI 43 |
| PXN | APKcGGCARAILENYIs | 1.8577 | TBI+CM | TBI 43 |
| KIAA2026 | VIGGGcCKEQELtYE | 1.8301 | TBI+CM | TBI 43 |
| KIAA2026 | VIGGGcCKEQELTyE | 1.8301 | TBI+CM | TBI 43 |
| KIAA2026 | VIGGGCcKEQELtYE | 1.8301 | TBI+CM | TBI 43 |
| KIAA2026 | VIGGGcCKEQELtYE | 1.8301 | TBI+CM | TBI 43 |
| KIAA2026 | VIGGGcCKEQELtYE | 1.8301 | TBI+CM | TBI 43 |
| KIAA2026 | VIGGGCcKEQELtYE | 1.8301 | TBI+CM | TBI 43 |
| KIAA2026 | VIGGGcCKEQELtYE | 1.8301 | TBI+CM | TBI 43 |
| KIAA2026 | VIGGGcCKEQELtYE | 1.8301 | TBI+CM | TBI 43 |
| KIAA2026 | VIGGGCcKEQELtYE | 1.8301 | TBI+CM | TBI 43 |
| KIAA2026 | VIGGGcCKEQELtYE | 1.8301 | TBI+CM | TBI 43 |
| KIAA2026 | VIGGGcCKEQELtYE | 1.8301 | TBI+CM | TBI 43 |
| KIAA2026 | VIGGGCcKEQELtYE | 1.8301 | TBI+CM | TBI 43 |
| KIAA2026 | VIGGGcCKEQELtYE | 1.8301 | TBI+CM | TBI 43 |
| ATP9B | VcHHIHHVPSVLLsAGPG | 1.8224 | TBI+CM | TBI 43 |
| ATP9B | VcHHIHHVPSVLLsAGPG | 1.8224 | TBI+CM | TBI 43 |
| ATP9B | VcHHIHHVPSVLLsAGPG | 1.8224 | TBI+CM | TBI 43 |
| WDHD1 | KEVFsLAGPVVSMAGH | 1.7877 | TBI+CM | TBI 43 |
| WDHD1 | KEVFsLAGPVVSMAGH | 1.7877 | TBI+CM | TBI 43 |
| WDHD1 | KEVFsLAGPVVSMAGH | 1.7877 | TBI+CM | TBI 43 |
| WDHD1 | KEVFsLAGPVVSMAGH | 1.7877 | TBI+CM | TBI 43 |
| WDHD1 | KEVFsLAGPVVSMAGH | 1.7877 | TBI+CM | TBI 43 |
| WDHD1 | KEVFsLAGPVVSMAGH | 1.7877 | TBI+CM | TBI 43 |
| WDHD1 | KEVFsLAGPVVSMAGH | 1.7877 | TBI+CM | TBI 43 |
| WDHD1 | KEVFsLAGPVVSMAGH | 1.7877 | TBI+CM | TBI 43 |
| WDHD1 | KEVFsLAGPVVSMAGH | 1.7877 | TBI+CM | TBI 43 |
| WDHD1 | KEVFsLAGPVVSMAGH | 1.7877 | TBI+CM | TBI 43 |
| WDHD1 | KEVFsLAGPVVSMAGH | 1.7877 | TBI+CM | TBI 43 |
| TRB | LGPRDLSAGAsGSSQH | 1.7543 | TBI+CM | TBI 43 |
| TRB | LGPRDLSAGAsGSSQH | 1.7543 | TBI+CM | TBI 43 |
| TRB | LGPRDLSAGAsGSSQH | 1.7543 | TBI+CM | TBI 43 |
| KIAA2026 | VIGGGcCKEQELTyE | 1.7486 | TBI+CM | TBI 43 |
| KIAA2026 | VIGGGcCKEQELTyE | 1.7486 | TBI+CM | TBI 43 |
| KIAA2026 | VIGGGcCKEQELTyE | 1.7486 | TBI+CM | TBI 43 |
| KIAA2026 | VIGGGcCKEQELTyE | 1.7486 | TBI+CM | TBI 43 |
| KIAA2026 | VIGGGcCKEQELTyE | 1.7486 | TBI+CM | TBI 43 |
| KIAA2026 | VIGGGcCKEQELTyE | 1.7486 | TBI+CM | TBI 43 |
| KIAA2026 | VIGGGcCKEQELTyE | 1.7486 | TBI+CM | TBI 43 |
| MUC5B | ASATAAAARCPRALsIH | 1.7291 | TBI+CM | TBI 43 |
| MUC5B | ASATAAAARCPRALsIH | 1.7291 | TBI+CM | TBI 43 |
| MUC5B | ASATAAAARCPRALsIH | 1.7291 | TBI+CM | TBI 43 |
| MUC5B | ASATAAAARCPRALsIH | 1.7291 | TBI+CM | TBI 43 |
| MUC5B | ASATAAAARCPRALsIH | 1.7291 | TBI+CM | TBI 43 |
| MUC5B | ASATAAAARCPRALsIH | 1.7291 | TBI+CM | TBI 43 |
| MUC5B | ASATAAAARCPRALsIH | 1.7291 | TBI+CM | TBI 43 |
| KIAA2026 | VIGGGCcKEQELtYE | 1.7105 | TBI+CM | TBI 43 |
| KIAA2026 | VIGGGCcKEQELtYE | 1.7105 | TBI+CM | TBI 43 |
| KIAA2026 | VIGGGCcKEQELtYE | 1.7105 | TBI+CM | TBI 43 |
| KIAA2026 | VIGGGCcKEQELtYE | 1.7105 | TBI+CM | TBI 43 |
| KIAA2026 | VIGGGCcKEQELtYE | 1.7105 | TBI+CM | TBI 43 |
| KIAA2026 | VIGGGCcKEQELtYE | 1.7105 | TBI+CM | TBI 43 |
| KIAA2026 | VIGGGCcKEQELtYE | 1.7105 | TBI+CM | TBI 43 |
| PRRC2A | QLPVVNFGsLPPAPPP | 1.7062 | TBI+CM | TBI 43 |
| PRRC2A | QLPVVNFGsLPPAPPP | 1.7062 | TBI+CM | TBI 43 |
| PRRC2A | QLPVVNFGsLPPAPPP | 1.7062 | TBI+CM | TBI 43 |
| PRRC2A | QLPVVNFGsLPPAPPP | 1.7062 | TBI+CM | TBI 43 |
| PRRC2A | QLPVVNFGsLPPAPPP | 1.7062 | TBI+CM | TBI 43 |
| PRRC2A | QLPVVNFGsLPPAPPP | 1.7062 | TBI+CM | TBI 43 |
| PRRC2A | QLPVVNFGsLPPAPPP | 1.7062 | TBI+CM | TBI 43 |
| PRRC2A | QLPVVNFGsLPPAPPP | 1.7062 | TBI+CM | TBI 43 |
| PRRC2A | QLPVVNFGsLPPAPPP | 1.7062 | TBI+CM | TBI 43 |
| PRRC2A | QLPVVNFGsLPPAPPP | 1.7062 | TBI+CM | TBI 43 |
| PRRC2A | QLPVVNFGsLPPAPPP | 1.7062 | TBI+CM | TBI 43 |
| PRRC2A | QLPVVNFGsLPPAPPP | 1.7062 | TBI+CM | TBI 43 |
| PRRC2A | QLPVVNFGsLPPAPPP | 1.7062 | TBI+CM | TBI 43 |
| PRRC2A | QLPVVNFGsLPPAPPP | 1.7062 | TBI+CM | TBI 43 |
| PRRC2A | QLPVVNFGsLPPAPPP | 1.7062 | TBI+CM | TBI 43 |
| PRRC2A | QLPVVNFGsLPPAPPP | 1.7062 | TBI+CM | TBI 43 |
| PRRC2A | QLPVVNFGsLPPAPPP | 1.7062 | TBI+CM | TBI 43 |
| IGH | MYIsNDGtNcHFII | 2.114 | TBI | TBI 44 |
| IGH | MYIsNDGtNcHFII | 2.114 | TBI | TBI 44 |
| IGH | MYIsNDGtNcHFII | 2.114 | TBI | TBI 44 |
| IGH | ATGyFPEPVmVTcDTGS | 1.9726 | TBI | TBI 44 |
| IGH | ATGyFPEPVmVTcDTGS | 1.9726 | TBI | TBI 44 |
| IGH | AtGYFPEPVmVTcDTGS | 1.9726 | TBI | TBI 44 |
| MT-ND1 | cMLmIWsVFLLGVTLc | 1.9273 | TBI | TBI 44 |
| MT-ND1 | cmLMIWsVFLLGVTLc | 1.9273 | TBI | TBI 44 |
| MT-ND1 | CmLmIWsVFLLGVTLc | 1.9273 | TBI | TBI 44 |
| MT-ND1 | cMLmIWsVFLLGVTLc | 1.9273 | TBI | TBI 44 |
| MT-ND1 | cMLmIWsVFLLGVTLc | 1.9273 | TBI | TBI 44 |
| MT-ND1 | cmLMIWsVFLLGVTLc | 1.9273 | TBI | TBI 44 |
| MT-ND1 | CmLmIWsVFLLGVTLc | 1.9273 | TBI | TBI 44 |
| MT-ND1 | cMLmIWsVFLLGVTLc | 1.9273 | TBI | TBI 44 |
| MT-ND1 | cMLmIWsVFLLGVTLc | 1.9273 | TBI | TBI 44 |
| MUC19 | TGPFTGLtGTSAQSAGVTm | 1.9029 | TBI | TBI 44 |
| MUC19 | TGPFTGLtGTSAQSAGVTm | 1.9029 | TBI | TBI 44 |
| MUC19 | TGPFTGLtGTSAQSAGVTm | 1.9029 | TBI | TBI 44 |
| MT-ND1 | cmLMIWsVFLLGVTLc | 1.8843 | TBI | TBI 44 |
| MT-ND1 | cmLMIWsVFLLGVTLc | 1.8843 | TBI | TBI 44 |
| MT-ND1 | cmLMIWsVFLLGVTLc | 1.8843 | TBI | TBI 44 |
| IGH | AtGYFPEPVmVTcDTGS | 1.8817 | TBI | TBI 44 |
| MT-ND1 | CmLmIWsVFLLGVTLc | 1.872 | TBI | TBI 44 |
| MT-ND1 | CmLmIWsVFLLGVTLc | 1.872 | TBI | TBI 44 |
| MT-ND1 | CmLmIWsVFLLGVTLc | 1.872 | TBI | TBI 44 |
| WDR46 | AAPsCLCLCPcQPATSHS | 1.8452 | TBI | TBI 44 |
| WDR46 | AAPsCLCLCPcQPATSHS | 1.8452 | TBI | TBI 44 |
| KCNK18 | VTRLGKYLcMLYALFG | 1.8162 | TBI | TBI 44 |
| KCNK18 | VTRLGKYLcMLYALFG | 1.8162 | TBI | TBI 44 |
| KCNK18 | VTRLGKYLcMLYALFG | 1.8162 | TBI | TBI 44 |
| NOTCH4 | CPPGFQGSLcQDHVNPCE | 1.7024 | TBI | TBI 44 |
| NOTCH4 | CPPGFQGSLcQDHVNPCE | 1.7024 | TBI | TBI 44 |
| NOTCH4 | CPPGFQGSLcQDHVNPCE | 1.7024 | TBI | TBI 44 |
| NOTCH4 | CPPGFQGSLcQDHVNPCE | 1.7024 | TBI | TBI 44 |
| NOTCH4 | CPPGFQGSLcQDHVNPCE | 1.7024 | TBI | TBI 44 |
| NOTCH4 | CPPGFQGSLcQDHVNPCE | 1.7024 | TBI | TBI 44 |
| NOTCH4 | CPPGFQGSLcQDHVNPCE | 1.7024 | TBI | TBI 44 |
| NOTCH4 | CPPGFQGSLcQDHVNPCE | 1.7024 | TBI | TBI 44 |
| NOTCH4 | CPPGFQGSLcQDHVNPCE | 1.7024 | TBI | TBI 44 |
| NOTCH4 | CPPGFQGSLcQDHVNPCE | 1.7024 | TBI | TBI 44 |
| NOTCH4 | CPPGFQGSLcQDHVNPCE | 1.7024 | TBI | TBI 44 |
| MUC19 | IsVELRPCPSGQTG | 2.5408 | TBI | TBI 45 |
| MUC19 | yGSSSSGDSDKK | 2.5408 | TBI | TBI 45 |
| SVEP1 | AAGsVVSFKcMEGFVLNT | 2.1534 | TBI | TBI 45 |
| SVEP1 | AAGsVVSFKCmEGFVLNT | 2.1534 | TBI | TBI 45 |
| SVEP1 | AAGsVVSFKcMEGFVLNT | 2.1534 | TBI | TBI 45 |
| SVEP1 | AAGsVVSFKCmEGFVLNT | 2.1534 | TBI | TBI 45 |
| SVEP1 | AAGsVVSFKcMEGFVLNT | 2.1534 | TBI | TBI 45 |
| SVEP1 | AAGsVVSFKcMEGFVLNT | 2.1534 | TBI | TBI 45 |
| SVEP1 | AAGsVVSFKCmEGFVLNT | 2.1534 | TBI | TBI 45 |
| MUC6 | ACGCDSGGDCEcLcDAVAAY | 2.1138 | TBI | TBI 45 |
| MUC6 | ACGCDSGGDCEcLcDAVAAY | 2.1138 | TBI | TBI 45 |
| MUC6 | ACGCDSGGDcECLcDAVAAY | 2.1138 | TBI | TBI 45 |
| MUC6 | ACGCDSGGDcECLcDAVAAY | 2.0341 | TBI | TBI 45 |
| FRAS1 | GGcESSCGKGFYNRQGTCS | 2.0286 | TBI | TBI 45 |
| FRAS1 | GGcESSCGKGFYNRQGTCS | 2.0286 | TBI | TBI 45 |
| FRAS1 | GGcESSCGKGFYNRQGTCS | 2.0286 | TBI | TBI 45 |
| FRAS1 | GGcESSCGKGFYNRQGTCS | 2.0286 | TBI | TBI 45 |
| FRAS1 | GGcESSCGKGFYNRQGTCS | 2.0286 | TBI | TBI 45 |
| FRAS1 | GGcESSCGKGFYNRQGTCS | 2.0286 | TBI | TBI 45 |
| FRAS1 | GGcESSCGKGFYNRQGTCS | 2.0286 | TBI | TBI 45 |
| SVEP1 | AAGsVVSFKCmEGFVLNT | 1.9865 | TBI | TBI 45 |
| SMYD3 | AFAKVIcNSFTICNAEm | 1.9388 | TBI | TBI 45 |
| SMYD3 | AFAKVIcNSFTICNAEm | 1.9388 | TBI | TBI 45 |
| SMYD3 | AFAKVIcNSFTICNAEm | 1.9388 | TBI | TBI 45 |
| SMYD3 | AFAKVIcNSFTICNAEm | 1.9388 | TBI | TBI 45 |
| SMYD3 | AFAKVIcNSFTICNAEm | 1.9388 | TBI | TBI 45 |
| SMYD3 | AFAKVIcNSFTICNAEm | 1.9388 | TBI | TBI 45 |
| SMYD3 | AFAKVIcNSFTICNAEm | 1.9388 | TBI | TBI 45 |
| SMYD3 | AFAKVIcNSFTICNAEm | 1.9388 | TBI | TBI 45 |
| SMYD3 | AFAKVIcNSFTICNAEm | 1.9388 | TBI | TBI 45 |
| POU4F3 | HFSmEFcsCcPGWSAM | 1.8912 | TBI | TBI 45 |
| POU4F3 | HFSmEFCsccPGWSAM | 1.8912 | TBI | TBI 45 |
| POU4F3 | HFSmEFcsCcPGWSAM | 1.8912 | TBI | TBI 45 |
| POU4F3 | HFSmEFcsCcPGWSAM | 1.8912 | TBI | TBI 45 |
| POU4F3 | HFSmEFCsccPGWSAM | 1.8912 | TBI | TBI 45 |
| POU4F3 | HFSmEFcsCcPGWSAM | 1.8912 | TBI | TBI 45 |
| POU4F3 | HFSmEFcsCcPGWSAM | 1.8912 | TBI | TBI 45 |
| FBN2 | KRMCccTY | 1.8895 | TBI | TBI 45 |
| FBN2 | KRMCccTY | 1.8895 | TBI | TBI 45 |
| FBN2 | KRMCccTY | 1.8895 | TBI | TBI 45 |
| FBN2 | KRMCccTY | 1.8895 | TBI | TBI 45 |
| FBN2 | KRMCccTY | 1.8895 | TBI | TBI 45 |
| FBN2 | KRMCccTY | 1.8895 | TBI | TBI 45 |
| FBN2 | KRMCccTY | 1.8895 | TBI | TBI 45 |
| FBN2 | KRMCccTY | 1.8895 | TBI | TBI 45 |
| FBN2 | KRMCccTY | 1.8895 | TBI | TBI 45 |
| FBN2 | cECPEGLTLDG | 1.8895 | TBI | TBI 45 |
| FBN2 | KRMCccTY | 1.8895 | TBI | TBI 45 |
| FBN2 | KRMCccTY | 1.8895 | TBI | TBI 45 |
| CACHD1 | VGPVAGGIMGCIMV | 1.8155 | TBI | TBI 45 |
| CACHD1 | VGPVAGGIMGCIMV | 1.8155 | TBI | TBI 45 |
| CACHD1 | VGPVAGGIMGCIMV | 1.8155 | TBI | TBI 45 |
| CACHD1 | VGPVAGGIMGCIMV | 1.8155 | TBI | TBI 45 |
| CACHD1 | VGPVAGGIMGCIMV | 1.8155 | TBI | TBI 45 |
| CACHD1 | VGPVAGGIMGCIMV | 1.8155 | TBI | TBI 45 |
| CACHD1 | VGPVAGGIMGCIMV | 1.8155 | TBI | TBI 45 |
| CACHD1 | VGPVAGGIMGCIMV | 1.8155 | TBI | TBI 45 |
| CACHD1 | VGPVAGGIMGCIMV | 1.8155 | TBI | TBI 45 |
| CTSW | SVQELLDcGRCGDGC | 1.7716 | TBI | TBI 45 |
| CTSW | SVQELLDcGRCGDGC | 1.7716 | TBI | TBI 45 |
| CTSW | SVQELLDcGRCGDGC | 1.7716 | TBI | TBI 45 |
| CTSW | SVQELLDcGRCGDGC | 1.7716 | TBI | TBI 45 |
| CTSW | SVQELLDcGRCGDGC | 1.7716 | TBI | TBI 45 |
| CTSW | SVQELLDcGRCGDGC | 1.7716 | TBI | TBI 45 |
| CTSW | SVQELLDcGRCGDGC | 1.7716 | TBI | TBI 45 |
| POU4F3 | HFSmEFCsccPGWSAM | 1.7714 | TBI | TBI 45 |
| POU4F3 | HFSmEFCsccPGWSAM | 1.7714 | TBI | TBI 45 |
| POU4F3 | HFSmEFCsccPGWSAM | 1.7714 | TBI | TBI 45 |
| TNRC6B | GmcAGGYSHISHcR | 1.7149 | TBI | TBI 45 |
| TNRC6B | GmcAGGYSHISHcR | 1.7149 | TBI | TBI 45 |
| TNRC6B | GmcAGGYSHISHcR | 1.7149 | TBI | TBI 45 |
| LRP1 | CSVYGtcSQLcTNTD | 2.385 | TBI | TBI 46 |
| LRP1 | CSVYGtcSQLcTNTD | 2.385 | TBI | TBI 46 |
| LRP1 | CSVYGtcSQLcTNTD | 2.385 | TBI | TBI 46 |
| LRP1 | CSVYGtcSQLcTNTD | 2.385 | TBI | TBI 46 |
| LRP1 | CSVYGtcSQLcTNTD | 2.385 | TBI | TBI 46 |
| LRP1 | LDQCWEHcRNGGtCAAS | 2.385 | TBI | TBI 46 |
| DNAH9 | IKKGRFIKIGDKEcEY | 2.2563 | TBI | TBI 46 |
| DNAH9 | IKKGRFIKIGDKEcEY | 2.2563 | TBI | TBI 46 |
| DNAH9 | IKKGRFIKIGDKEcEY | 2.2563 | TBI | TBI 46 |
| DNAH9 | IKKGRFIKIGDKEcEY | 2.2563 | TBI | TBI 46 |
| DNAH9 | IKKGRFIKIGDKEcEY | 2.2563 | TBI | TBI 46 |
| DNAH9 | IKKGRFIKIGDKEcEY | 2.2563 | TBI | TBI 46 |
| DNAH9 | IKKGRFIKIGDKEcEY | 2.2563 | TBI | TBI 46 |
| DNAH9 | IKKGRFIKIGDKEcEY | 2.2563 | TBI | TBI 46 |
| ITGB3 | MAsCSAELLPPPRccG | 2.0723 | TBI | TBI 46 |
| ITGB3 | MAsCSAELLPPPRccG | 2.0723 | TBI | TBI 46 |
| ITGB3 | MAsCSAELLPPPRccG | 2.0723 | TBI | TBI 46 |
| ITGB3 | MASCsAELLPPPRccG | 2.0723 | TBI | TBI 46 |
| ITGB3 | MAsCSAELLPPPRccG | 2.0723 | TBI | TBI 46 |
| ITGB3 | MASCsAELLPPPRccG | 2.0121 | TBI | TBI 46 |
| ITGB3 | MASCsAELLPPPRccG | 2.0121 | TBI | TBI 46 |
| ITGB3 | MASCsAELLPPPRccG | 2.0121 | TBI | TBI 46 |
| SYNE2 | QTEEGTTPPIEADtLDS | 1.9504 | TBI | TBI 46 |
| SYNE2 | QTEEGTTPPIEADtLDS | 1.9504 | TBI | TBI 46 |
| SYNE2 | QTEEGTTPPIEADtLDS | 1.9504 | TBI | TBI 46 |
| SYNE2 | QTEEGTTPPIEADtLDS | 1.9504 | TBI | TBI 46 |
| SYNE2 | QTEEGTTPPIEADtLDS | 1.9504 | TBI | TBI 46 |
| SYNE2 | QTEEGTTPPIEADtLDS | 1.9504 | TBI | TBI 46 |
| SYNE2 | QTEEGTTPPIEADtLDS | 1.9504 | TBI | TBI 46 |
| MT-ND1 | CFmcILVFCGLSYLSY | 1.824 | TBI | TBI 46 |
| MT-ND1 | CFmcILVFCGLSYLSY | 1.824 | TBI | TBI 46 |
| MT-ND1 | CFmcILVFCGLSYLSY | 1.824 | TBI | TBI 46 |
| MT-ND1 | CFmcILVFCGLSYLSY | 1.824 | TBI | TBI 46 |
| MT-ND1 | CFmcILVFCGLSYLSY | 1.824 | TBI | TBI 46 |
| FBN1 | GTCRNTIGsFNcRcNH | 1.8104 | TBI | TBI 46 |
| FBN1 | GTCRNTIGsFNcRcNH | 1.8104 | TBI | TBI 46 |
| FBN1 | GTCRNTIGsFNcRcNH | 1.8104 | TBI | TBI 46 |
| FBN1 | GTCRNTIGsFNcRcNH | 1.8104 | TBI | TBI 46 |
| FBN1 | GTCRNTIGsFNcRcNH | 1.8104 | TBI | TBI 46 |
| FBN1 | GTCRNTIGsFNcRcNH | 1.8104 | TBI | TBI 46 |
| IGL | FcSLYTTTtTLVFGGGT | 1.8053 | TBI | TBI 46 |
| IGL | FcSLYTTTtTLVFGGGT | 1.8053 | TBI | TBI 46 |
| IGL | FcSLYTTTtTLVFGGGT | 1.8053 | TBI | TBI 46 |
| IGH | FLVAAATGAQSVTtIPPH | 1.7861 | TBI | TBI 46 |
| IGH | FLVAAATGAQSVTtIPPH | 1.7861 | TBI | TBI 46 |
| IGH | FLVAAATGAQSVTtIPPH | 1.7861 | TBI | TBI 46 |
| MUC5B | IRVFccNYGHcPStPA | 2.2233 | TBI+CM | TBI 7 |
| MUC5B | IRVFccNYGHcPStPA | 2.2233 | TBI+CM | TBI 7 |
| MUC5B | IRVFccNYGHcPsTPA | 2.2233 | TBI+CM | TBI 7 |
| MUC5B | IRVFccNYGHcPsTPA | 2.0944 | TBI+CM | TBI 7 |
| PLP1 | AHsLERVCHcLGKWL | 1.8521 | TBI+CM | TBI 7 |
| PLP1 | AHsLERVCHcLGKWL | 1.8521 | TBI+CM | TBI 7 |
| PLP1 | AHsLERVCHcLGKWL | 1.8521 | TBI+CM | TBI 7 |
| PLP1 | AHsLERVCHcLGKWL | 1.8521 | TBI+CM | TBI 7 |
| PLP1 | AHsLERVCHcLGKWL | 1.8521 | TBI+CM | TBI 7 |
| PLP1 | AHsLERVCHcLGKWL | 1.8521 | TBI+CM | TBI 7 |
| PLP1 | AHsLERVCHcLGKWL | 1.8521 | TBI+CM | TBI 7 |
| LAMA1 | GYYGNPtVPGEScVPCDC | 1.8391 | TBI+CM | TBI 7 |
| LAMA1 | GYYGNPtVPGEScVPCDC | 1.8391 | TBI+CM | TBI 7 |
| LAMA1 | GYYGNPtVPGEScVPCDC | 1.8391 | TBI+CM | TBI 7 |
| LAMA1 | GYYGNPtVPGEScVPCDC | 1.8391 | TBI+CM | TBI 7 |
| LAMA1 | GYYGNPtVPGEScVPCDC | 1.8391 | TBI+CM | TBI 7 |
| LAMA1 | GYYGNPtVPGEScVPCDC | 1.8391 | TBI+CM | TBI 7 |
| RELN | QcEEMCNGQGScINGTKc | 1.8183 | TBI+CM | TBI 7 |
| RELN | QCEEMcNGQGScINGTKc | 1.8183 | TBI+CM | TBI 7 |
| RELN | QcEEMCNGQGScINGTKc | 1.8183 | TBI+CM | TBI 7 |
| RELN | QCEEMcNGQGScINGTKc | 1.8183 | TBI+CM | TBI 7 |
| RELN | QcEEMCNGQGScINGTKc | 1.8183 | TBI+CM | TBI 7 |
| RELN | QCEEMcNGQGScINGTKc | 1.8183 | TBI+CM | TBI 7 |
| RELN | QcEEMCNGQGScINGTKc | 1.8183 | TBI+CM | TBI 7 |
| RELN | QCEEMcNGQGScINGTKc | 1.8183 | TBI+CM | TBI 7 |
| RELN | QcEEMCNGQGScINGTKc | 1.8183 | TBI+CM | TBI 7 |
| RELN | QcEEMCNGQGScINGTKc | 1.8183 | TBI+CM | TBI 7 |
| RELN | QCEEMcNGQGScINGTKc | 1.8183 | TBI+CM | TBI 7 |
| EGF | DFLTDKLYWcDAKQSV | 1.8181 | TBI+CM | TBI 7 |
| EGF | DFLTDKLYWcDAKQSV | 1.8181 | TBI+CM | TBI 7 |
| EGF | DFLTDKLYWcDAKQSV | 1.8181 | TBI+CM | TBI 7 |
| EGF | DFLTDKLYWcDAKQSV | 1.8181 | TBI+CM | TBI 7 |
| EGF | DFLTDKLYWcDAKQSV | 1.8181 | TBI+CM | TBI 7 |
| RELN | QCEEMcNGQGScINGTKc | 1.8085 | TBI+CM | TBI 7 |
| ERBB3 | NEcRPcHENctQGCK | 1.7584 | TBI+CM | TBI 7 |
| ERBB3 | NEcRPcHENctQGCK | 1.7584 | TBI+CM | TBI 7 |
| ERBB3 | NEcRPcHENctQGCK | 1.7584 | TBI+CM | TBI 7 |
| ERBB3 | NEcRPcHENctQGCK | 1.7584 | TBI+CM | TBI 7 |
| ERBB3 | NEcRPcHENctQGCK | 1.7584 | TBI+CM | TBI 7 |
| ERBB3 | NEcRPcHENctQGCK | 1.7584 | TBI+CM | TBI 7 |
| ERBB3 | NEcRPcHENctQGCK | 1.7584 | TBI+CM | TBI 7 |
| ZNF879 | VcAFSQcSSFQFGsDCG | 1.723 | TBI+CM | TBI 7 |
| ZNF879 | VcAFSQcSSFQFGsDCG | 1.723 | TBI+CM | TBI 7 |
| ZNF879 | VcAFSQcSSFQFGsDCG | 1.723 | TBI+CM | TBI 7 |
| MEGF10 | CEQGTyGNDcHQRcQC | 1.704 | TBI+CM | TBI 7 |
| MEGF10 | CEQGTyGNDcHQRcQC | 1.704 | TBI+CM | TBI 7 |
| MEGF10 | CEQGTyGNDcHQRcQC | 1.704 | TBI+CM | TBI 7 |
| MEGF10 | CEQGTyGNDcHQRcQC | 1.704 | TBI+CM | TBI 7 |
| MEGF10 | CEQGTyGNDcHQRcQC | 1.704 | TBI+CM | TBI 7 |
| MUC19 | SNPATCSNVAPFQDsE | 1.7005 | TBI+CM | TBI 7 |
| MUC19 | SNPATCSNVAPFQDsE | 1.7005 | TBI+CM | TBI 7 |
| MUC19 | SNPATCSNVAPFQDsE | 1.7005 | TBI+CM | TBI 7 |
| MUC19 | SNPATCSNVAPFQDsE | 1.7005 | TBI+CM | TBI 7 |
| MUC19 | SNPATCSNVAPFQDsE | 1.7005 | TBI+CM | TBI 7 |
| MUC19 | SNPATCSNVAPFQDsE | 1.7005 | TBI+CM | TBI 7 |
| MUC19 | SNPATCSNVAPFQDsE | 1.7005 | TBI+CM | TBI 7 |
| MUC19 | SNPATCSNVAPFQDsE | 1.7005 | TBI+CM | TBI 7 |
| MUC19 | SNPATCSNVAPFQDsE | 1.7005 | TBI+CM | TBI 7 |
